# PIM kinases mediate resistance of glioblastoma cells to TRAIL by a p62/SQSTM1-dependent mechanism

**DOI:** 10.1038/s41419-018-1293-3

**Published:** 2019-01-18

**Authors:** Santiago Serrano-Saenz, Carmen Palacios, Daniel Delgado-Bellido, Laura López-Jiménez, Angel Garcia-Diaz, Yolanda Soto-Serrano, J. Ignacio Casal, Rubén A. Bartolomé, José Luis Fernández-Luna, Abelardo López-Rivas, F. Javier Oliver

**Affiliations:** 1 Instituto de Parasitología y Biomedicina López-Neyra, CSIC, CIBERONC, Parque Tecnológico Ciencias de la Salud, Avenida del Conocimiento, s/n, 18100 Armilla, Granada Spain; 2 Centro de Investigación Biomédica en Red-Oncología (CIBERONC), Carlos III Health Institute, Madrid, Spain; 3 Centro Andaluz de Biología Molecular y Medicina Regenerativa-CABIMER, CSIC-Universidad de Sevilla-Universidad Pablo de Olavide, CIBERONC, Avda Américo Vespucio 24, 41092 Sevilla, Spain; 4 Department of Molecular Biomedicine, Centro de Investigaciones Biológicas, CSIC, Ramiro de Maeztu 9, 28039 Madrid, Spain; 5 HUMV—Hospital Universitario Marqués de Valdecilla Avenida Valdecilla, 25, 39008 Santander, Cantabria Spain

## Abstract

Glioblastoma (GBM) is the most common and aggressive brain tumor and is associated with poor prognosis. GBM cells are frequently resistant to tumor necrosis factor-related apoptosis-inducing ligand (TRAIL) and finding new combinatorial therapies to sensitize glioma cells to TRAIL remains an important challenge. PIM kinases are serine/threonine kinases that promote cell survival and proliferation and are highly expressed in different tumors. In this work, we studied the role of PIM kinases as regulators of TRAIL sensitivity in GBM cells. Remarkably, PIM inhibition or knockdown facilitated activation by TRAIL of a TRAIL-R2/DR5-mediated and mitochondria-operated apoptotic pathway in TRAIL-resistant GBM cells. The sensitizing effect of PIM knockdown on TRAIL-induced apoptosis was mediated by enhanced caspase-8 recruitment to and activation at the death-inducing signaling complex (DISC). Interestingly, TRAIL-induced internalization of TRAIL-R2/DR5 was significantly reduced in PIM knockdown cells. Phospho-proteome profiling revealed a decreased phosphorylation of p62/SQSTM1 after PIM knockdown. Our results also showed an interaction between p62/SQSTM1 and the DISC that was reverted after PIM knockdown. In line with this, p62/SQSTM1 ablation increased TRAIL-R2/DR5 levels and facilitated TRAIL-induced caspase-8 activation, revealing an inhibitory role of p62/SQSTM1 in TRAIL-mediated apoptosis in GBM. Conversely, upregulation of TRAIL-R2/DR5 upon PIM inhibition and apoptosis induced by the combination of PIM inhibitor and TRAIL were abrogated by a constitutively phosphorylated p62/SQSTM1^S332E^ mutant. Globally, our data represent the first evidence that PIM kinases regulate TRAIL-induced apoptosis in GBM and identify a specific role of p62/SQSTM1^Ser332^ phosphorylation in the regulation of the extrinsic apoptosis pathway activated by TRAIL.

## Introduction

Glioblastoma multiforme, classified by World Health Organization (WHO) as grade IV astrocytoma, is the most common and aggressive brain tumor in adults. Median survival of GBM patients is 14.6 months^1^. Current therapy involves surgery, followed by radiation and adjuvant alkylating chemotherapy with temozolomide^2,3^. Despite improvement, GBM is still a challenge for medical research and new therapies are urgently required.

TRAIL/Apo2L is a cytokine of the tumor necrosis factor (TNF) gene superfamily that selectively induces apoptosis in many tumor cells while leaving normal cells intact and remains an attractive candidate for antitumor therapies^4^. TRAIL induces apoptosis upon binding to death domain (DD)-containing receptors TRAIL-R1/DR4 and TRAIL-R2/DR5. This interaction activates the recruitment of the intracellular adaptor molecule FAS-associated death domain protein (FADD), which concurrently engages procaspase-8 at the death-inducing signaling protein complex (DISC)^5^. Within the DISC, caspase-8 is activated by transcatalytic and autocatalytic cleavage and released into the cytoplasm, initiating the protease cascade. Caspase-8 activation at the DISC subsequently leads to effector caspases activation, thereby triggering the execution of the extrinsic apoptotic pathway. In addition, activated caspase-8 is able to cleave Bid, a BH3-only pro-apoptotic member of the Bcl-2 family protein, releasing a truncated protein (tBid) that translocates to the mitochondrial outer-membrane and, in concert with other pro-apoptotic Bcl-2 family proteins, induces the release of apoptogenic factors, thereby amplifying caspase activation^6^. However, most of GBM cells are resistant to TRAIL treatment and new therapeutic targets must be found to sensitize these tumor cells to TRAIL^7^.

PIM kinases belong to a family of three highly conserved serine/threonine kinases proteins with short half-life^8^. They share high homology at the amino acid sequences and have functional redundancy. PIM kinases also present overlapping function with Akt, suggesting cross-talk between them in the control of survival signaling pathways^9–11^. Over-expression of PIM kinases correlate with poor prognosis in several hematological^12–15^ and solid tumors^16–18^, including GBM^19^. PIM overexpression in cancer increases malignancy by direct regulation of several processes as apoptosis, cell cycle progression, or migration^8^. In addition, mice lacking all three PIM kinases are viable and fertile, which suggests that pharmacological PIM inhibition might have low toxicity^20^. For these reasons, PIM inhibition, alone or in combination, has been proposed as an encouraging treatment against cancer and several inhibitors have been developed^8^.

P62/SQSTM1 is a multifunctional scaffold protein involved in different cellular processes including selective autophagy, antioxidant response, endosomal trafficking, inflammation, and apoptosis^21^. Aberrant amplification and phosphorylation of p62/SQSTM1 have been implicated in tumor development and resistance to therapy^22,23^.

In the current study, we have investigated the role of PIM kinases in the control of TRAIL resistance in GBM cells. Our results represent the first evidence that abrogating PIM function sensitizes GBM cells to TRAIL-induced cell death. Disabling PIM kinases upregulates TRAIL-R2/DR5 expression and inhibits TRAIL-induced internalization of this receptor, thus facilitating TRAIL-induced apoptosis. In addition, we identified p62/SQSTM1 phosphorylation as a key event involved in the regulation of TRAIL-induced cell death by PIM kinases. Altogether, these results suggest that targeting PIM kinases in combination with pro-apoptotic TRAIL receptor agonists may represent new therapeutic strategies against gliomas.

## Results

### Disabling PIM kinases function sensitizes GBM cells to TRAIL-induced apoptosis

**Fig. 1:**
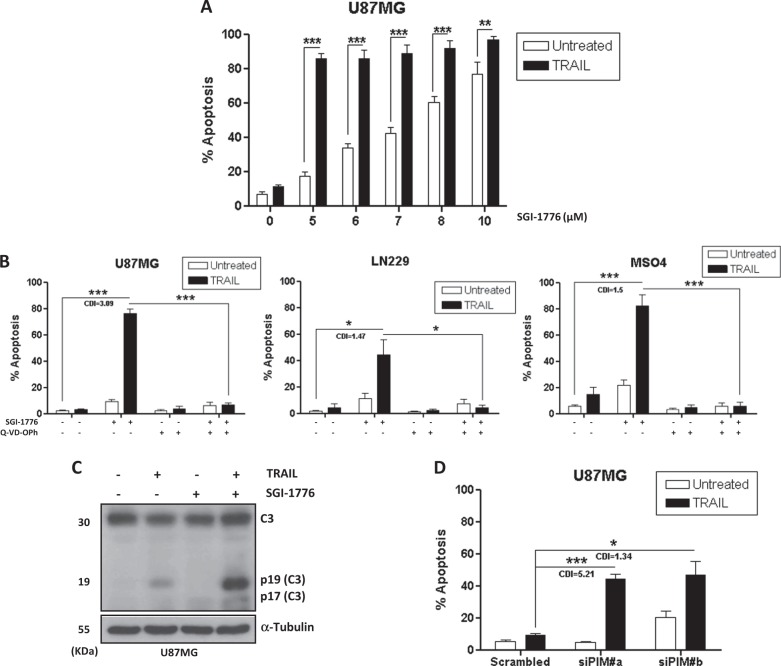
PIM kinases disabling sensitizes GBM cells to TRAIL-induced apoptosis. **A** U87MG cells were treated for 48 h with the indicated concentrations of SGI-1776 in the presence or absence of TRAIL (500 ng/mL). Apoptosis was determined by flow cytometry (hypodiploid cells). **B** GBM cell lines (U87MG and LN-229) and primary cultures of GBM cells (MSO4) were treated as indicated and apoptosis was assessed after 48 h. SGI-1776 (5 μM), TRAIL (500 ng/mL), and Q-VD-OPh (20 μM) were used in these experiments. **C** Caspase-3 activation was determined by western blot after 22 h of treatment with TRAIL (500 ng/mL) and SGI-1776 (5 μM). **D** GBM cells were incubated in the presence of small interfering RNA (siRNAs) for 48 h to knockdown PIM2 and PIM3 expression prior to treatment with TRAIL for 48 h. Apoptosis was assessed by flow cytometry. PIM proteins knockdown was determined by western blot (Fig. S2B). Two alternatives siRNAs sequences were used to reduce the risk of off-target effect (siPIM#a and siPIM#b)

To examine the role of PIM kinases in the regulation of TRAIL resistance in GBM cells, we initially determined the effect of the PIM kinases inhibitor SGI-1776 in apoptosis induced by TRAIL in the human GBM cell line U87MG. U87MG cells showed a marked resistance to TRAIL even at saturating concentrations of this death ligand (Fig. S1A). A dose-dependent apoptotic response of U87MG cells to SGI-1776 in combination with TRAIL was found at doses of the PIM kinases inhibitor between 5 and 10 μM (Fig. 1A and S1B). Similar results were obtained in another GBM cell line (LN-229) and in patient-derived primary cultures of GBM cells (MSO4)^24^ (Fig. S1C). Pan-caspases inhibitor Q-VD-OPh completely inhibited the generation of hypodiploid cells by the combination treatment confirming a caspase-dependent cell death mechanism in all three human GBM cell models tested (Fig. 1B). Moreover, effector caspase-3 activation, which is considered the point-of-no-return during apoptosis^25^, was also observed in cells treated with a combination of TRAIL and SGI-1776 (Fig. 1C). Importantly, inhibition of caspases with Q-VD-OPh promoted long-term survival as determined in clonogenicity assays (Fig. S2A).

Interestingly, PIM kinases inhibitor did not sensitize normal mouse astrocytes to a high dose of TRAIL (Fig. S1D, left panel). Owing to the difficulty of obtaining cultures of human astrocytes, we used normal human endothelial cells (HUVEC) as a non-tumor primary human cell model to determine the potential toxicity of the combination treatment in normal cells. In this model, the combination of TRAIL + SGI-1776 has no synergic effect in the range of PIM inhibitor concentrations that results in a marked sensitization to TRAIL in GBM cells (Fig. S1D, right panel).

To verify the target specificity of the observed sensitizing effects of SGI-1776 on TRAIL-induced apoptosis in GBM cells, we first checked the expression of PIM kinase family members in U87MG cells. As shown in Fig. S2B U87MG cells expressed PIM2 and PIM3, but we did not detect PIM1 (Fig. S2B). Next, we silenced the expression of PIM kinases with specific siRNAs prior to the addition of TRAIL. Knockdown of either PIM2 or PIM3 did not result in sensitization of GBM cells to TRAIL-induced apoptosis (data not shown). Interestingly, simultaneous knockdown of PIM2 and PIM3 strongly sensitized U87MG to TRAIL (Fig. 1D and S2B), which suggested a redundant function of PIM kinases in the regulation of TRAIL-induced apoptosis. Similar results were obtained in LN-229 cells and in patient-derived primary cultures of GBM cells MSO4 (Fig. S2C). The effect of the combined treatment was synergic as determined by calculating the coefficient of drug interaction (CDI > 1).

**Fig. 2:**
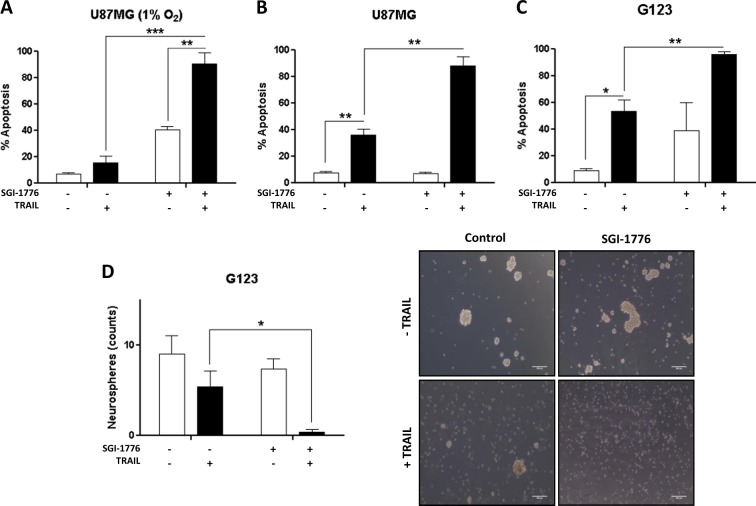
Targeting PIM kinases abrogates resistance of GBM cells to TRAIL under hypoxic and stemness culture conditions. **A** U87MG treated with SGI-1776 (5 μM) and TRAIL (500 ng/mL) for 48 h under hypoxic conditions (1% O_2_). Apoptotic cell death was measured by flow cytometry. **B** U87MG cells were cultured in stemness medium to enrich the culture in GSCs. U87MG GSCs were treated with SGI-1776 (5 μM) and TRAIL (500 ng/mL) 24 h and cell death was measured by flow cytometry. **C** G123 primary glioma cells cultured in stemness medium were treated with SGI-1776 (5 μM) and TRAIL (500 ng/μL) 24 h. Apoptosis was determined by flow cytometry. **D** G123 primary glioma cells cultured in stemness medium were treated with SGI-1776 (5 μM) and TRAIL (500 ng/mL) for 24 h. Neurospheres were counted as indicated in materials and methods. Scale bar (500 μm)

GBM is a highly aggressive tumor, due in part to a fast proliferation that renders large hypoxic and anoxic areas^26^. Hypoxia in turn shapes the phenotype of glioma cells through the activation of the expression of a variety of genes that allow adaptation to hostile low oxygen conditions and increases resistance to different treatments^27^. In this line, we further investigated the effect of SGI-1776 and TRAIL combination in GBM cells under hypoxic conditions that may be present in the tumor microenvironment. Results shown in Fig. 2A indicated that SGI-1776 induced apoptosis in U87MG cells and markedly sensitized these cells to TRAIL under low O_2_ conditions.

Another important feature of GBM is the presence of glioma stem cells (GSCs), a tumor subpopulation with high plasticity and pluripotency. GSCs show high resistance to conventional treatment and the capacity to regenerate the tumor^28–30^. Under specific cell culture conditions (as described in methods), GSCs exhibit neurospheres formation capacity^31^. To examine the effect of the combinatorial treatment over GSCs, we enriched U87MG stem cells by culturing them in stemness medium prior to treatment with SGI-1776 and TRAIL. As shown in Fig. 2B neurosphere-forming cells showed partial sensitivity to TRAIL that was strongly enhanced by SGI-1776 treatment. Likewise, treatment with PIM inhibitor and TRAIL significantly induced apoptosis in GSCs from primary GBM G123 cultures (Fig. 2C) and completely abrogated neurospheres formation (Fig. 2D). Collectively, these results suggest that inhibiting PIM kinases markedly sensitizes GSCs to TRAIL-induced apoptosis.

**Fig. 3:**
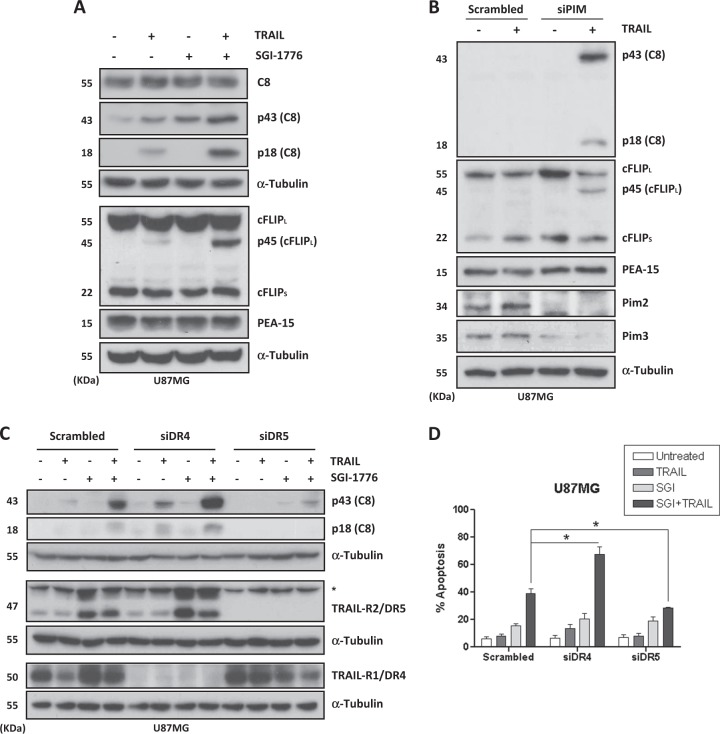
Involvement of TRAIL-R2/DR5 in TRAIL-induced caspase-8 activation and apoptosis upon inhibition of PIM kinases function. Caspase-8 activation, cFLIP_**L**_ cleavage, and PEA-15 levels were assessed by western blot in U87MG cells extracts after overnight treatment with 5 μM SGI-1776 (**A**) or PIM silencing with siRNAs (**B**) followed by 6-h treatment with TRAIL (500 ng/mL). **C**, **D** U87MG cells were transfected for 48 h either with a scrambled oligonucleotide or with the indicated small interfering RNAs (siRNAs) targeting TRAIL-R1/DR4 or TRAIL-R2/DR5. In **C** cells were then treated with SGI-1776 (5 μM) overnight prior to incubation for 6 h in medium with TRAIL (500 ng/mL). Caspase-8 activation was determined by western blot. In **D** cells were treated with SGI-1776 (5 μM) and TRAIL (500 ng/mL) for 48 h. Hypodiploid apoptotic cells were determined by flow cytometry

To get further insight into the mechanism underlying the observed sensitization to TRAIL-induced apoptosis by SGI-1776 we initially determined procaspase-8 processing by western blot as a measurement of potential caspase-8 activation at the DISC. As shown in Fig. 3A, TRAIL-induced procaspase-8 processing was mainly observed in U87MG cells pretreated with SGI-1776. Likewise, cleavage of cFLIP_L_, a known caspase-8 substrate^32^, was strongly induced in cells treated with the combination of SGI-1776 and TRAIL (Fig. 3A). Importantly, these results were confirmed by silencing PIM kinases expression with siRNAs prior to TRAIL addition (Fig. 3B). Similar results were obtained in GBM primary cells MSO4 (Fig. S3A).

We also studied the role of pro-apoptotic receptors TRAIL-R1/DR4 and TRAIL-R2/DR5 in caspase-8 activation after treatment with SGI-1776 and TRAIL. Results shown in Fig. 3C demonstrate that silencing of TRAIL-R2/DR5 markedly abrogated caspase-8 processing in U87MG cells treated with PIM inhibitor and TRAIL. TRAIL-R2/DR5 knockdown also significantly attenuated TRAIL-induced apoptosis in cells treated with the PIM kinase inhibitor (Fig. 3D and S3B). On the contrary, silencing of TRAIL-R1/DR4 not only did not prevent the activation of caspase-8 and the generation of hypodiploid cells but it also increased both parameters in U87MG cells incubated with SGI-1776 and TRAIL (Fig. 3C, D). Altogether, these data demonstrated that pro-apoptotic TRAIL-R2/DR5 was responsible for caspase-8 activation and apoptosis induced by the combination of PIM kinase inhibitor and TRAIL in U87MG cells.

### PIM kinases regulate caspase-8 activation at the TRAIL DISC

**Fig. 4:**
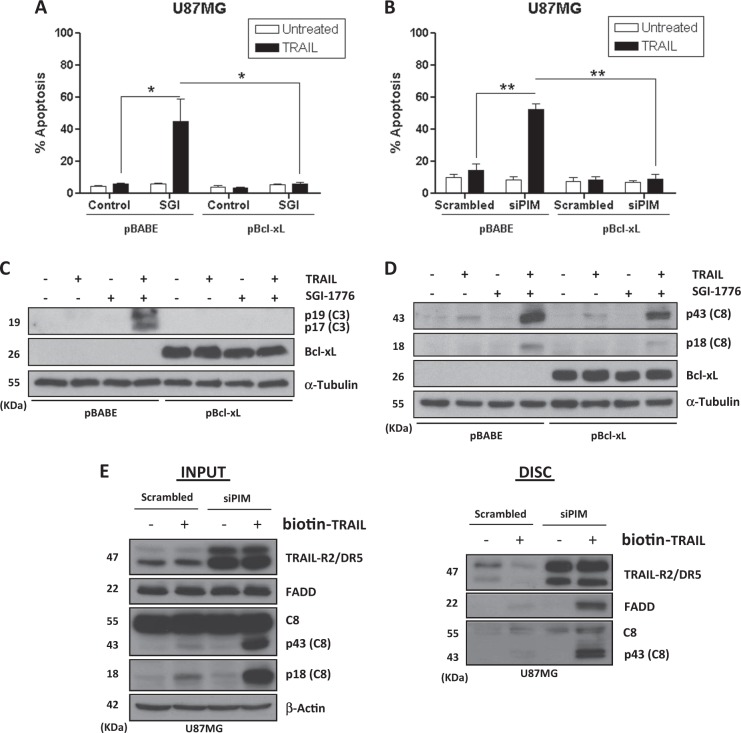
PIM kinases inhibition facilitates TRAIL-induced caspase-8 activation at the death-inducing signaling complex (DISC) and apoptosis through a mitochondria-operated pathway. **A** Mock or Bcl-xL-overexpressing U87MG cells were treated with SGI-1776 (5 μM) and TRAIL (500 ng/mL) for 48 h. Apoptosis was measured by flow cytometry. **B** Mock or Bcl-xL-overexpressing U87MG cells were transfected for 48 h either with a scrambled oligonucleotide or with the indicated small interfering RNAs (siRNAs) targeting PIM2 and PIM3 prior to treatment with TRAIL (500 ng/mL) for 48 h. Hypodiploid cells were determined by flow cytometry. **C** Caspase-3 activation in mock and Bcl-xL cells treated with SGI-1776 (5 μM) and TRAIL (500 ng/mL) for 22 h. **D** Caspase-8 activation in mock and Bcl-xL U87MG cells after incubation with SGI-1776 (5 μM) overnight followed by treatment with TRAIL (500 ng/mL) for 6 h. **E** U87MG cells were transfected with siRNAs for PIM2 and PIM3 kinases and then treated with biotin-TRAIL (1000 ng/mL) for 90 min. DISC complexes were pulled down with streptavidin-agarose beads as indicated in the materials and methods section and analyzed by western blot

Cells are classified in two types regarding the apoptotic mechanism activated upon death receptor ligation by TRAIL. In type I cells, caspase-8 activation at the DISC is sufficient to stimulate effector caspases and induce apoptosis. In contrast, in type II cells, Bid cleavage by caspase-8 and the subsequent activation of the mitochondrial-operated apoptotic pathway are required to elicit a cell death program^33^. Overexpression of the anti-apoptotic Bcl-xL protein blocks the mitochondrial pathway of apoptosis and has been frequently used to distinguish between both cell types in apoptosis induced by death ligands^33^. To this end, we generated U87MG stably overexpressing Bcl-xL by retroviral infection (Fig. S4A). Strikingly, TRAIL-induced apoptosis was completely inhibited in U87MG-Bcl-xL cells treated with SGI-1776 (Fig. 4A and S4B) or after PIM kinases knockdown (Fig. 4B). Accordingly, Bcl-xL overexpression inhibited caspase-3 activation in GBM cells treated with TRAIL and PIM kinases inhibitor for 22 h (Fig. 4C) confirming that U87MG GBM cells are type II cells that require amplification of apoptosis signaling through the mitochondria. Importantly, although slightly reduced, TRAIL-induced caspase-8 activation was also observed in Bcl-xL cells treated with PIM inhibitor (Fig. 4D). Mechanistically, these results suggest that the sensitizing effect of PIM kinases disabling in TRAIL-induced apoptosis in GBM cells must be positioned upstream of the mitochondria. To further confirm that PIM kinases modulate apical caspase-8 activation by TRAIL, we performed a DISC precipitation assay with Biotin-TRAIL^34^. As shown in Fig. 4E, TRAIL DISC formation and caspase-8 activation were clearly enhanced in PIM knockdown U87MG cells (Fig. S4C), which demonstrated that PIM kinases modulate early events in the pathway leading to TRAIL-induced cell death in GBM cells.

### Inhibition of PIM function increases TRAIL-R2/DR5 protein levels and prevents TRAIL-induced internalization favoring caspase-8 activation

**Fig. 5:**
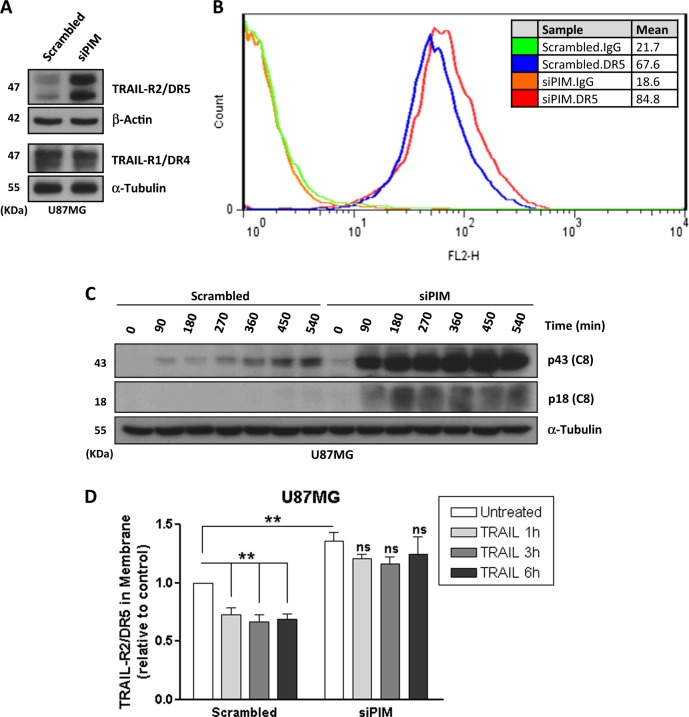
PIM kinases knockdown abolishes TRAIL-induced TRAIL-R2/DR5 internalization, favoring caspase-8 activation. **A** Expression of death receptors (TRAIL-R1/DR4 and TRAIL-R2/DR5) were determined by western blot in U87MG cells after PIM2/3 knockdown. **B** Plasma membrane levels of TRAIL-R2/DR5 following PIM knockdown were analyzed in U87MG by flow cytometry as described in the materials and methods section. **C** U87MG cells were transfected with a scrambled oligonucleotide or siRNAs targeting PIM2 and PIM3 and treated with TRAIL (500 ng/mL) for the indicated times. Caspase-8 activation was determined by western blot. **D** U87MG transfected either with a scrambled oligonucleotide or small interfering RNAs (siRNAs) targeting PIM2/3 were treated with TRAIL (500 ng/mL) for the indicated times. Cell surface TRAIL-R2/DR5 levels were assessed by flow cytometry as described under materials and methods

To further explore the mechanism underlying sensitization to TRAIL upon PIM inhibition in GBM cells, we determined by quantitative PCR pathway array the expression of genes involved in the signaling pathway activated upon TRAIL binding to pro-apoptotic TRAIL-R2/DR5. Results of these experiments did not show significant differences in mRNA expression of key proteins of the TRAIL-activated signaling pathway (Fig. S5A). To further investigate possible changes in the levels of DISC proteins, we determined by western blot the levels of canonical components of this complex. As shown in Fig. 5A, TRAIL-R2/DR5 levels were significantly upregulated after PIM knockdown, whereas those of other relevant DISC proteins like TRAIL-R1/DR4, FADD, caspase-8, and FLIP remained unaltered (Fig. 5A and S5B). Collectively, these results suggested a post-transcriptional regulation TRAIL-R2/DR5 expression by PIM kinases, confirming results obtained in GBM cells treated with PIM kinases inhibitor (Fig. 3C). Moreover, TRAIL-R2/DR5 protein upregulation upon PIM kinases disabling was also paralleled by an increase in TRAIL-R2/DR5 at the cell surface (Fig. 5B). To get further insight into the mechanism leading to sensitization to TRAIL in GBM cells we performed time-course experiments of caspase-8 activation in control and siPIM cells incubated with TRAIL. Results shown in Fig. 5C demonstrate sustained caspase-8 activation by TRAIL in PIM knockdown cells as compared to control cells. From these results, we reasoned that changes in TRAIL-R2/DR5 protein dynamics may underlie the sustained activation of caspase-8 by TRAIL in GBM cells in which PIM kinases have been either chemically or genetically inhibited. To this end, we determined TRAIL-R2/DR5 levels at the cell surface by flow cytometry after different times of TRAIL treatment in control and PIM knockdown cells. Interestingly, while control cells showed a significant internalization of TRAIL-R2/DR5 after treatment with TRAIL, TRAIL-R2/DR5 membrane levels remained elevated in PIM knockdown cells for up-to 6 h in the presence of TRAIL (Fig. 5D). These results suggested that the internalization machinery might be compromised after PIM inhibition thus allowing the sustained activation of caspase-8, which in turn may further inhibit TRAIL-R2/DR5 internalization as reported^35^

### Control of p62/sequestosome-1 protein phosphorylation by PIM kinases and its involvement in the regulation of TRAIL sensitivity in GBM cells

**Fig. 6:**
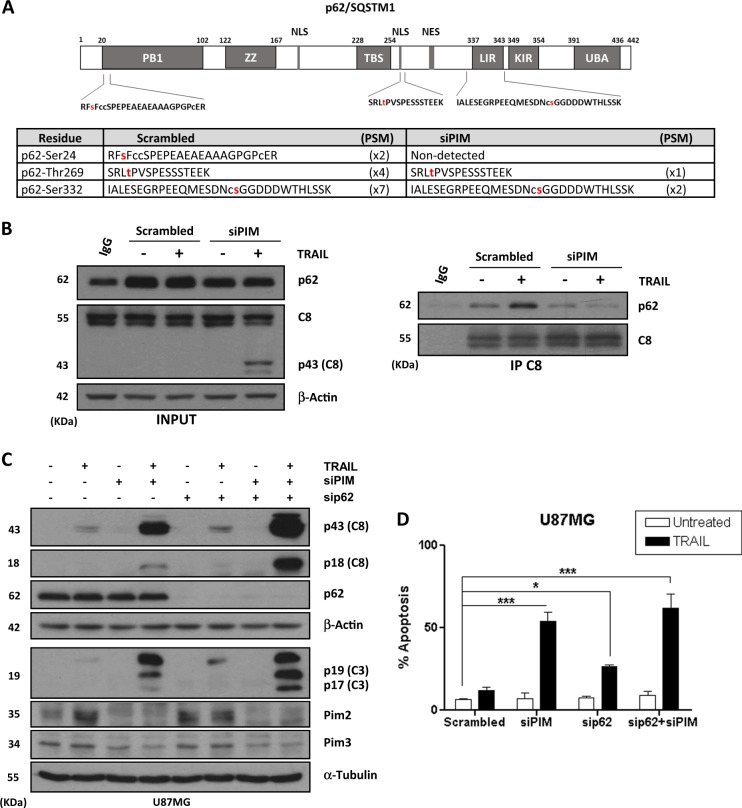
p62/SQSTM1 involvement in the control of TRAIL resistance by PIM kinases in GBM cells. **A** Phosphopeptides detected in p62/SQSTM1 protein by mass spectrometry. The phosphorylation site is marked in red. **B** U87MG cells were transfected with a scrambled oligonucleotide or siRNAs targeting PIM2 and PIM3 and treated with TRAIL (500 ng/mL) for 6 h. Caspase-8 was then immunoprecipitated as described under materials and methods and interaction with p62/SQSTM1 was analyzed by western blot. U87MG cells were transfected with the indicated small interfering RNAs (siRNAs) for 48 h prior to treatment with TRAIL for 6 (**C**) or 48 (**D**) hours. Activation of initiator and effector caspases was assessed by western blot, using antibodies that recognize the cleavage fragments (**C**). Apoptosis was determined by flow cytometry (**D**)

In order to identify protein targets that may be responsible for the sensitization of GBM cells to TRAIL following PIM kinases inhibition, we performed omics assays by mass spectrometry (MS-MS) to analyze the global phosphoproteomic signature after PIM knockdown. We identified 1456 phosphoproteins and observed a decrease in the phosphorylation status of 569 proteins (Fig. S6A and Table S1), including several proteins related with caspase-8 function (Fig. S6C)^36–39^. Among these proteins, we observed changes in the phosphorylation status of the autophagy adaptor protein p62/SQSTM1^40^. Thus, phosphorylation in Ser24, Thr269 and Ser332 residues were detected in control (scrambled RNA oligonucleotide) but not in PIM knockdown cells (Fig. 6A and Tables S2 and S3). To further investigate the role of p62/SQSTM1 in the sensitization to TRAIL observed in GBM cells after PIM disabling, we performed co-immunoprecipitation assays to test whether p62/SQSTM1 interacts with caspase-8 upon TRAIL treatment as reported in other tumor cells^36^. Results shown in Fig. 6B, demonstrated enhanced p62/SQSTM1-caspase-8 interaction after TRAIL treatment in control (scrambled) cells. Intriguingly, under conditions of PIM knockdown-mediated sensitization to TRAIL, interaction between caspase-8 and p62/SQSTM1 was markedly reduced (Fig. 6B), suggesting that in GBM cells p62/SQSTM1 is playing a negative role in caspase-8 activation after TRAIL treatment. To demonstrate this inhibitory interaction, we silenced p62/SQSTM1 expression and measured caspases activation and apoptosis after TRAIL treatment. Strikingly, silencing p62/SQSTM1 expression partially sensitized GBM cells to TRAIL and further increased TRAIL-induced activation of initiator and effector caspases in PIM knockdown cells (Fig. 6C, D).

**Fig. 7:**
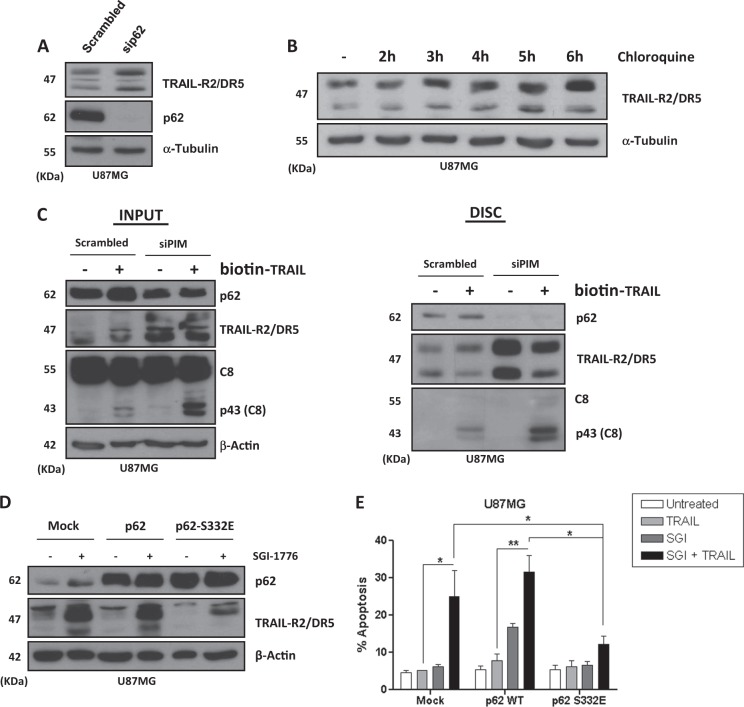
PIM kinases-dependent phosphorylation of p62/SQSTM1 in Ser332 controls TRAIL-R2/DR5 levels in GBM cells. **A** U87MG cells were transfected with a scrambled oligonucleotide or p62/SQSTM1 small interfering RNAs (siRNAs) for 48 h and then TRAIL-R2/DR5 levels were analyzed by western blot. **B** Western blot analysis of TRAIL-R2/DR5 levels in U87MG cells treated with chloroquine for the indicated times. **C** U87MG cells were transfected with the indicated siRNAs for 48 h prior to treatment with biotin-TRAIL (1000 ng/mL) for 90 min. TRAIL DISC was pulled down with streptavidin-agarose beads as indicated in the materials and methods section and proteins associated to DISC were determined by western blot. **D** U87MG cells were transiently transfected with the indicated plasmids using jetPRIME reagent. After transfection, cells were treated overnight with or without SGI-1776 (5 μM) and protein expression was analyzed by western blot. **E** U87MG cells were transfected with plasmids p62/SQSTM1 wild-type and p62/SQSTM1^S233E^ mutant as described. After transfection, cells were treated for 48 h with SGI-1776 (5 μM), TRAIL (500 ng/mL), or both. Apoptosis was determined by flow cytometry

p62/SQSTM1 plays an important role in selective lysosomal degradation of proteins by autophagy^41^. To get further insight into the role of p62/SQSTM1 in TRAIL-R2/DR5 up-regulation and TRAIL DISC formation in PIM knockdown GBM cells, we first determined TRAIL-R2/DR5 levels in GBM cells depleted of p62/SQSTM1 by siRNA. As shown in Fig. 7A, p62/SQSTM1 knockdown significantly increased TRAIL-R2/DR5 levels in U87MG cells. In addition, inhibition of lysosome-dependent protein degradation with chloroquine increased TRAIL-R2/DR5 protein levels (Fig. 7B). Altogether, these results suggest that TRAIL-R2 levels in GBM cells are subject to a p62-dependent degradation in lysosomes that may be regulated by PIM kinases. In this respect, results shown in Fig. 7C, right panel, demonstrate a basal interaction between TRAIL-R2/DR5 and p62/SQSTM1 at the TRAIL DISC where this cargo receptor may interact with caspase-8 (Fig. 6B). Interestingly, PIM kinases knockdown abolished interaction of p62/SQSTM1 with TRAIL-R2/DR5 at the TRAIL DISC (Fig. 7C, right panel) as well as with caspase-8 (Fig. 6B).

Phosphoproteomic signature generated in GBM cells after PIM knockdown allowed us to identify changes in the phosphorylation status of p62/SQSTM1 (Fig. 6A). Among these changes, loss of phosphorylation in Ser332 residue could be of interest as this residue is located within the LIR domain that is crucial in selective degradation of target proteins via autophagy^42^. These results prompted us to investigate its possible role in mediating TRAIL-R2/DR5 up-regulation after PIM inhibition. To this end, we generated by site-directed mutagenesis a mutant form of p62/SQSTM1 protein with a serine to glutamic acid substitution (p62/SQSTM1^S332E^) to mimic constitutively phosphorylated p62/SQSTM1 (Fig. S7). Whereas ectopic expression of wild type p62/SQSTM1 did not prevent TRAIL-R2/DR5 upregulation following SGI-1776 treatment, overexpression of the p62/SQSTM1^S332E^ mutant form markedly inhibited TRAIL-R2/DR5 upregulation upon PIM inhibition (Fig. 7D). Importantly, overexpression of the p62/SQSTM1^S332E^ mutant significantly attenuated apoptosis induced by the combination of PIM kinase inhibitor and TRAIL, confirming that the reduction of phosphorylation in p62/SQSTM1-Ser332 after PIM inhibition detected by MS-MS is implicated in TRAIL-R2/DR5 stabilization and mediates sensitization to TRAIL in GBM cells (Fig. 7E).

## Discussion

Despite an intensive research, GBM patients still need efficient and safe therapeutic options. GBM tumors are characterized by extensive hypoxia and recurrent disease arising from therapy resistant cancer cells that very often leads to elevated mortality. In addition, therapy resistant GBM cells have been found to exhibit stem cell-like properties. TRAIL is a promising treatment against tumors, showing high sensitivity and selectivity to induce apoptosis in tumor cells. The ability of TRAIL to induce apoptosis in tumor cells has prompted researches to further investigate its potential as an antitumor agent^43,44^. Nevertheless, many primary tumors are resistant to TRAIL or can acquire resistance during therapy^4^. In these cases, the use of TRAIL in combination with other treatments can result in additive or synergistic antitumor effects. GBM cells are frequently resistant to TRAIL^7,45,46^. Our results show for the first time clear evidences that PIM inhibition is an effective target to overcome the resistance to TRAIL-mediated apoptosis in GBM cells. Notably, our data demonstrated that the combination of PIM kinases disabling and TRAIL was completely effective in inducing apoptosis in patient-derived primary cultures of GBM cells and neurospheres, with enriched stem-like phenotype and closer resemblance to the clinical situation.

Regarding GBM, a potential therapeutic opportunity was suggested through the knockdown of proteins like cFLIP, PEA-15, and RIPK1 that negatively regulate caspase-8 activation at the DISC, as silencing their expression sensitized GBM cells to TRAIL treatment^47^. These results suggested that an effective activation of caspase-8 might be sufficient to induce apoptosis in GBM cells^7^. However, in spite of effective apical caspase-8 activation by TRAIL after PIM kinases inhibition, our data clearly demonstrate that DISC inhibitors such as FLIP and PEA-15 were not downregulated under these conditions. Death receptor-induced apoptosis can proceed through the mitochondria in type II cells^33^. Our data also demonstrate that GBM cells belong to the type II group as apoptosis was completely blocked in cells overexpressing anti-apoptotic Bcl-xL protein of the Bcl-2 family. However, synergistic activation of caspase-8 by PIM kinases inhibition was still observed in Bcl-xL cells suggesting that the anti-apoptotic action of PIM kinases was located up-stream of mitochondria, probably affecting the early events of TRAIL signaling^5^.

TRAIL-R1/DR4 and TRAIL-R/DR5 are pro-apoptotic receptors due to the presence of a cytoplasmic death domain which allows triggering of apoptosis upon TRAIL binding^48^. Interestingly, in the present report we have demonstrated that TRAIL-R2/DR5 mediates TRAIL-induced cell death upon PIM kinases inhibition. Intriguingly, our data suggest that TRAIL-R1/DR4 may act as an inhibitory receptor for TRAIL in U87MG cells treated with PIM inhibitor, counteracting the pro-apoptotic role of TRAIL-R2/DR5. Although both TRAIL-R1/DR4 and TRAIL-R2/DR5 are involved in TRAIL-induced apoptosis in different tumor cells, both receptors may also activate survival pathways^49,50^. Whether TRAIL-R1/DR4 is preferentially activating survival pathways in GBM cells upon TRAIL binding is an issue that warrants further investigation.

Unlike TNF-R1 and CD95, TRAIL receptor-triggered caspase activity has been demonstrated to halt clathrin-dependent endocytosis of TRAIL pro-apoptotic receptors, leading to amplification of apoptosis signaling by TRAIL^35,51,52^. Interestingly, our data demonstrate that silencing PIM expression elevates TRAIL-R2/DR5 levels at the plasma membrane and prevents TRAIL-induced TRAIL-R2/DR5 internalization which results in sustained DISC formation and caspase-8 activation, overcoming the action of the anti-apoptotic machinery in the DISC.

**Fig. 8:**
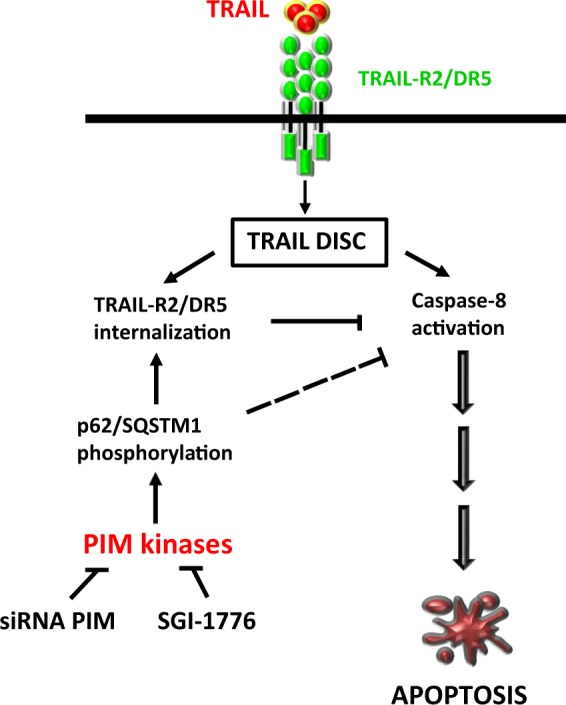
Schematic overview of the proposed mechanism of PIM kinases-mediated resistance to TRAIL-induced apoptosis in GBM cells. PIM kinases promote resistance to TRAIL in GBM cells through a mechanism involving p62/SQSTM1 phosphorylation

Interestingly, we have also found that after PIM disabling, p62/SQSTM1 phosphorylation is affected as part of the whole phosphoproteomic signature induced by PIM knockdown. Despite other data indicating a pro-apoptotic role of p62/SQSTM1 in some tumor cells facilitating full activation of ubiquitinated caspase-8 upon TRAIL receptor engagement by TRAIL^36^, our results demonstrate that in GBM cells p62/SQSTM1 interacts with the TRAIL DISC and plays a negative role in the activation of apoptosis by TRAIL. Interestingly, p62/SQSTM1 interaction with the TRAIL DISC does not take place in PIM knockdown cells. Although we lack a complete picture of the molecular mechanism responsible for the sensitization process, the fact that p62/SQSTM1 is involved in internalization of some membrane receptors^53^ suggests that the phosphorylation of this scaffold protein by PIM kinases may lead to recruitment of TRAIL-R2/DR5 to regions of the plasma membrane more accessible to the endocytic machinery, thus facilitating its internalization and termination of apoptosis signaling. In addition, autophagic degradation of active caspase-8 has been reported to underlie the resistance of some tumor cells to TRAIL^54^. Interestingly, GBM cells display high cytoprotective autophagy in response to chemotherapy^55^ and TRAIL sensitivity is restored after autophagy inhibition in different tumor settings^55,56^. It is well known that p62/SQSTM1 functions as a selective autophagy receptor linking ubiquitinated proteins with LC3 to promote lysosomal degradation^57^. LC3-interacting regions (LIRs) are short linear motifs within selective autophagy cargo receptors that mediate binding to LC3^42^. Phosphorylation of the LC3-interacting region (LIR) motif has been implicated in the regulation of cargo receptor function^58^. Our data demonstrate that phosphorylation of Ser332 residue of p62/SQSTM1 is important in the regulation of TRAIL sensitivity by PIM kinases. Interestingly, this residue is located within the LIR domain of p62/SQSTM1, which suggests that direct or indirect phosphorylation by PIM kinases may promote interaction of this cargo receptor with LC3 for autophagosome formation leading to cargo degradation after fusion with lysosomes. Altogether, our results suggest that p62/SQSTM1 plays a complex and cell type-dependent role in the activation of caspase-8 upon TRAIL-R2/DR5 activation by TRAIL (Fig. 8), regulated by PIM kinases.

In summary, our results represent the first evidence that PIM kinases are involved in the regulation of TRAIL-induced apoptosis in GBM. Although PIM inhibitor SGI-1776 was withdrawn from clinical trials due to cardiac toxicity, we have used this small molecule as proof-of-concept to demonstrate the important role of PIM kinases as mediators of TRAIL resistance in GBM cells. Therefore, targeting PIM kinases with inhibitors that might exhibit a more favorable therapeutic profile may provide novel therapeutic approaches to treat GBM in combination with agonists of TRAIL receptors.

## Materials and methods

### Cell culture and reagents

U87MG cell line was purchased from the European Collection of Authenticated Cell Cultures (Cat. 89081402) through the University of Granada Cell Lines Service. LN929 cell line was a donation from Dr. Joan Seoane (Vall d’Hebron Institute of Oncology, Barcelona). Patient-derived primary cultures of glioblastoma cells MSO4 were a gift of Dr. Victor Yuste (Autonomous University of Barcelona). GBM cell lines and mouse astrocytes were cultured in Dulbecco's Modified Eagle Medium (DMEM) high glucose medium supplemented with 10% of inactivated fetal bovine serum (FBS) and gentamicin (50 μg/mL). Neurospheres cultures of patient-derived G123 glioblastoma cells were maintained in DMEM-F12 medium without FBS but supplemented with N2 (Invitrogen), B27 (Invitrogen), G5 (Invitrogen), Amphotericin B 150 μg/mL (Gibco), and Streptomycin–Penicillin (1:1) 50 μg/mL. U87MG neurospheres were also enriched in this medium. Human umbilical endothelial cells (HUVEC) were grown in endothelial growth medium-2 (EGM-2) (Lonza) supplemented with EGM-2 Bulletkit (Catalog#CC-3162). HeLa cells were cultured in DMEM low glucose medium supplemented with 10% of inactivated fetal bovine serum (FBS) and gentamicin (50 μg/mL). All cell lines were tested for mycoplasma contamination and maintained at 37 °C in a 5% CO_2_-humidified incubator. For hypoxic conditions, U87MG were incubated in a hypoxic camera with 1% oxygen. Soluble human His-tagged recombinant TRAIL was produced in our laboratory as described^34^. PIM kinases inhibitor SGI-1776 was purchased from Selleckchem. Pan-Caspase inhibitor Q-VD-OPh was from Apexbio (Hsinchu, Taiwan). Lysosomal degradation inhibitor Chloroquine was purchased from Sigma-Aldrich (C6628–25G).

### Determination of apoptosis

Cells (1.5 × 10^5^/well) were treated in 6-well plates as indicated in the figure legends. After treatment, hypodiploid apoptotic cells were detected by flow cytometry according to published procedures^59^. Briefly, cells were washed with cold phosphate-buffered saline (PBS), fixed in 70% cold ethanol and then stained with propidium iodide while treating with RNAse. Quantitative analysis of the cell cycle and hypodiploid cells was carried out in a FACSCalibur cytometer using the FlowJO7.6.3 software (Tree Star).

### Immunoblot analysis of proteins

After treatment cells were washed with cold PBS and lysed in TR3 lysis buffer (3% SDS, 10% glycerol, 10 mM Na_2_HPO_4_). Protein concentration was determined by DC Protein Assay (BioRad). Loading buffer (10 × ) was added and samples were boiled for 5 min. Proteins were resolved on polyacrylamide gel electrophoresis (PAGE) and detected as described previously^59^. Antibodies used were: Caspase-3 polyclonal antibody (Enzo Life Sciences), Caspase-8 (1C12), Cleaved Caspase-8 (Asp391) (18C8), cleaved Caspase-3 (Asp175), PIM3 (D17C9), TRAIL-R2/DR5 and p61/SQSTM1 (Cell Signaling); PIM2 (F-11), PEA-15 (H-3), and GAPDH (Santa Cruz Biotechnology); cFlip (NF6) (AdipoGen); Bcl-xL and FADD (BD Pharmingen); TRAIL-R1/DR4 (R&D Systems); α-Tubulin and anti-β-Actin clone AC-74 (Sigma).

### Clonogenic assay

Cells (5 × 10^2^) were cultured in 12-wells plates, treated as indicated and incubated for 2 weeks. Colonies were fixed and stained with a mixture of 0.5% crystal violet in 50/50 methanol/water. Proliferation was determined after solubilization in 1% SDS by measuring of absorbance at 590 nm.

### Neurosphere formation assay

Neurospheres cultured under stemness conditions^60^ were dissociated and plated in 12-well plates. After 24-h treatment, the ability to form neurospheres was determined. Results are presented as the mean of blindly counts of ten fields. Later, cells were dissociated and apoptosis was measured by flow cytometry.

### **Reverse transcription polymerase chain reaction** (RT-PCR)

Total RNA was isolated by RNeasy Mini Kit (Qiagen) according to the manufacturer’s recommendations. One microgram of isolated RNA was treated with DNAse I and RNAsin Ribonuclease inhibitors (Invitrogen) and reverse-transcribed using iScriptcDNA synthesis kit (BioRad). cDNA was amplified using Q5® Hot Start High-Fidelity 2X Master Mix (M0494S, New England Biolabs) in CFX96 real-time PCR detection systems. Primer sequences for the targets are the following: 36B4 (Housekeeping gene): Forward 5′-CAGATTGGCTACCCAACTGTT-3′, Reverse 5′-GGCCAGGACTCGTTTGTACC-3′; PIM1: Forward 5′-CAGAGTGGATCCGCTACCAT-3′, Reverse 5′-TGGATTTCTTCGAAGGTTGG-3′; PIM2: Forward 5′-TGGGCATCCTCCTCTATGAC-3′, Reverse 5′-GTACTACCTCGGCTGGTGTT-3′; PIM3: Forward 5′-AAGCTCATCGACTTCGGTTC-3′, Reverse 5′-AGGATCTCCTCGTCCTGCTC-3′. cDNA amplifications were resolved on agarose gel electrophoresis and visualized with Gel Red nucleic acid stain (BIOTUM) in a Gel Doc EZ Imager (BioRad).

### RNA interference

Cells were transfected in 6-well plates with jetPRIME (Polyplus) according to manufacturer’s instructions. siRNAs were used at 50 nM. For PIM knockdown, a pool of siPIM2 and siPIM3 (1:1) was applied. siRNA employed were: scrambled (5′-CCUACAUCCCGAUCGAUGAUG[dT][dT]-3′); PIM2#a (5´-GUGGAGUUGUCCAUCGUGACA[dT][dT]-3´); PIM2#b (5′-ACCUUCUUCCCGACCCUCA[dT][dT]-3′); PIM3#a (5´-GGCGUGCUUCUCUACGAUA[dT][dT]-3´); PIM3#b (5′-GCACGUGGUGAAGGAGCGG[dT][dT]-3´); TRAIL-R1/DR4 (5´-GGAACUUUCCGGAAUGAC[dT][dT]-3´); TRAIL-R2/DR5 (5´-GACCCUUGUGCUCGUUGUC[dT][dT]-3´); TRAIL-R2/DR5#b (5′-UCAUGUAUCUAGAAGGUAA-[dT][dT]-3´); and p62/SQSTM1 (5′-GCAUUGAAGUUGAUAUCGA[dT][dT]-3´ All siRNAs were synthesized by Sigma-Aldrich (St. Louis, MO).

### Generation of Bcl-xL-overexpressing GBM cells

pBabe and pBabe-Bcl-xL retroviral vectors for stable gene protein expression were provided by Dr. Cristina Muñoz-Pinedo (IDIBELL, Barcelona, Spain). Retroviruses were produced by transfection of HEK293-T cells by the calcium phosphate method with the corresponding plasmids. Retrovirus-containing supernatants were collected 48 h after transfection and concentrated by ultracentrifugation at 22,000 rpm for 90 min at 4 °C.

Stable populations of U87MG cells infected with retroviruses were obtained after selection in culture medium containing puromycin (1.5 µg/mL) during 48 h.

### DISC isolation

DISC precipitation was performed using biotin-labeled recombinant TRAIL (biotin-TRAIL)^34^. Cells were treated with bio-TRAIL for the times indicated in the figure legends. DISC formation was then stopped and unbound TRAIL was removed by washing the cells three times in ice-cold PBS. Cells were lysed in 3 mL of lysis buffer (30 mM Tris/HCl, pH 7.5, 150 mM NaCl, 10% glycerol, 1% Triton X-100, 0.1% of sodium deoxycholate) containing Complete mini protease inhibitors cocktail (Roche Molecular Biochemicals)) for 30 min on ice followed by centrifugation at 13 K for 30 min at 4 °C. To provide an unstimulated receptor control, biotin-TRAIL was added to lysates from untreated cells. The TRAIL DISC was collected from the lysates with 30 μL of streptavidin-agarose beads after overnight incubation at 4 °C. Beads were collected by centrifugation, washed six times with lysis buffer and receptor complexes were eluted with 30 μL of sample buffer. Proteins were analyzed by western blotting.

### Analysis of TRAIL receptor internalization by flow cytometry

After TRAIL treatment cells were washed with cold PBS and cell surface-bound TRAIL was removed by three acid washes in cold-high glucose DMEM medium containing 0.2% BSA, pH 3.5 with HCl. Cells were detached with trypsin solution and resuspended in growth media. After incubation for 15 min under cell culture conditions (37 °C in a 5% CO_2_-humidified, 95% air incubator), cells were washed with ice-cold PBS and resuspended in PBS. Cells were then labeled either with 5 µg/mL of anti-TRAIL-R2-PE antibody or an IgG-PE control (BD Bioscience) for 30 min on ice and darkness. Analysis of the cell surface receptor expression was carried out in a FACSCalibur cytometer using the FlowJO7.6.3 software (Tree Star).

### Co-Immunoprecipitation

A total of 5 × 10^6^ cells were lysed with GST-FISH lysis buffer (NP40 1:100, Glycerol 1:10 in Tris-HCl 10 mM with NaCl 5 mM, supplemented with proteases and phosphatases inhibitors) on ice during 30 min and centrifuged at 13,000 rpm (30 min, 4 °C). Lysates were then incubated with capture antibody O/N at 4 °C. Capture antibody was collected with Dynabeads Protein G (Invitrogen) during 6 h at 4 °C. Beads were washed six times with lysis buffer and resuspended in loading buffer. Beads were boiled for 5 min and supernatant proteins were resolved by electrophoresis and detected by western blot using TrueBlot ULTRA secondary antibody (Tebu-bio).

### Analysis of apoptosis gene expression profile by pathway array

Cells (5 × 10^5^) were treated overnight with SGI-1776 (5 μM) and RNA was obtained using the RNeasy kit (Qiagen). Apoptosis gene expression was determined with human RT^2^ Profiler^TM^ PCR Arrays for apoptosis (PAHS-012Z, QUIAGEN) in a CFX96 thermocycler (BioRad). Data were analyzed according to manufacture instructions in QIAGEN web site.

### Phosphoproteins detection by mass spectrometry (MS-MS)

Cells were lysed and 300 μg of protein extract were precipitated with acetone and resuspended in 50 mM ammonium bicarbonate solution containing 8 M urea. After reduction and alkylation, samples were digested with trypsin and desalted with Clean-Up. Samples were enriched in phosphopeptides by affinity chromatography on TiO_2_ columns. Enriched samples were analyzed on nano-LC-MS in Easy-nLC 1000 (Proxeon) coupled to an ionic source with nanoelectrospray (ThermoScientific). MS-MS were acquired by LTQ-OrbitrapVelos (ThermoScientific). Raw files were analyzed in Uniprot database using Sequest in Proteome Discoverer (ThermoScientific). Peptides identification was validated by Percolator using *q*-value ≤ 0.01

### Analysis of phosphoproteins from MS-MS data

Selection of proteins showing reduced phosphorylation after PIM knockdown was performed after defining a 1.6 times decrease threshold in phosphopeptides detected by MS-MS. The resulting list was compared with the list of annotated interacting proteins in Biogrid Database for caspase-8. The coincidence between both lists was analyzed in STRING database to define the interaction.

### Site-directed mutagenesis

A constitutively phosphorylated mutant in Ser332 of p62/SQSTM1^(S→E)^ was generated using plasmid HA-p62/SQSTM1 (Plasmid #28027, Addgene) as a template. Mutation was introduced with Q5 Site-Directed Mutagenesis Kit (E0554S), (New England BioLabs) according to manufacture instructions. Specific primers were online designed with the program NEBaseChanger software: Sense (5´-GGATAACTGTGAAGGAGGAGATG-3´); antisense (5´-GACTCCATCTGTTCCTCAG-3´) and synthesized by Sigma-Aldrich (St. Louis, MO).

### Data analysis

For determination of synergy between PIM kinases disabling and TRAIL in the activation of apoptosis we calculated the coefficient of drug interaction (CDI):

CDI = *AB*/(*A* x *B*)

Where *A* is the effect of TRAIL, *B* is the effect of PIM inhibition and *AB* is the effect of combine treatment. If CDI > 1 the effect of combination is considered synergy.

Results are shown as the mean and standard error of mean ( ± SEM) of three independents experiments. Statistical significance was determined by ANOVA test and *p*-values below 0.05 were considered significant (*p < 0.05; **p < 0.01; ***p < 0.001).

## Supplementary information

Supplemental data
