# Supplementary material for: PIM kinases mediate resistance of glioblastoma cells to TRAIL by a p62/SQSTM1-dependent mechanism
Source: Cell Death Dis. 2019 Jan 18;10(2):51. doi: 10.1038/s41419-018-1293-3 (PMC6362213; doi:10.1038/s41419-018-1293-3)
Supplement: Supplementary file 1 — Supplemental data [file 41419_2018_1293_MOESM1_ESM.pdf]

Supplementary figures Legend

Figure S1. (A) U87MG cells were treated for 48 hours with different doses of TRAIL. Apoptosis was determined by flow cytometry. (B, C and D) Cells were treated for 48 hours with the indicated concentrations of SGI-1776 in combination with TRAIL 500ng/ml. Apoptosis was measured by flow cytometry.

Figure S2. (A) Clonogenicity assay in U87MG treated as indicated with SGI-1776 (5μM), TRAIL (500ng/ml) and Q-VD-Oph (20μM). Cells were treated for 48 hours, washed and cultured for 2 weeks. Clonogenic growth was determined as described under materials and methods by measuring absorbance at 590nm. (B) Expression of mRNAs of PIM kinases in U87MG. PIM 1 mRNA expression in HeLa cells was also determined as a control. PIM 2 and 3 knockdown was confirmed by western blot. (C) TRAIL-induced apoptosis was determined in LN-229 and MSO4 GBM cells after PIM 2 and 3 knockdown.

Figure S3. (A) MSO4 cells were transfected for 48 hours with a scrambled oligonucleotide or siRNAs targeting PIM2 and PIM3 and subsequently treated with TRAIL (500ng/mL) for 6 hours. Caspase-8 activation was determined by western blot. (B) U87MG cells were transfected for 48 hours either with a scrambled oligonucleotide or a siRNA targeting TRAIL-R2/DR5 prior to treatment for 48 hours with the indicated additions. Apoptotic cells were determined by flow cytometry.

Figure S4. (A) Analysis by western blot of Bcl-xL expression in pBabe and pBabe-Bcl-xL U87MG cells. (B) Time-course of apoptosis induction in pBabe and pBabe-Bcl-xL U87MG cells by the indicated treatments. (C) Analysis by western blot of PIM 2 and 3 knockdown in samples from the experiment shown in Figure 4E.

Figure S5. (A) Heat map and scattering plot of apoptosis human pathway array (RT2 ProfilerTM PCR Array). Threshold=2. (B) Analysis by western blot of intracellular proteins of the extrinsic apoptosis pathway after knockdown of PIM2/3 in U87MG cells.

Figure S6. Summary of phosphorylated proteins detected by MS-MS (A) in samples from PIM2/3 knockdown U87MG cells as analyzed by western blot (B). (C) Proteins that interact with caspase 8 (BioGrid) show decreased phosphorylation upon PIM2/3 knockdown as detected in by MS-MS.

Figure S7. (A) Coding sequence of p62/SQSTM1 wild type and p6/SQSTM12-S332E mutant. (B) Protein alignment by ClustalW2 between wild type and S332E mutant p62/SQSTM1.

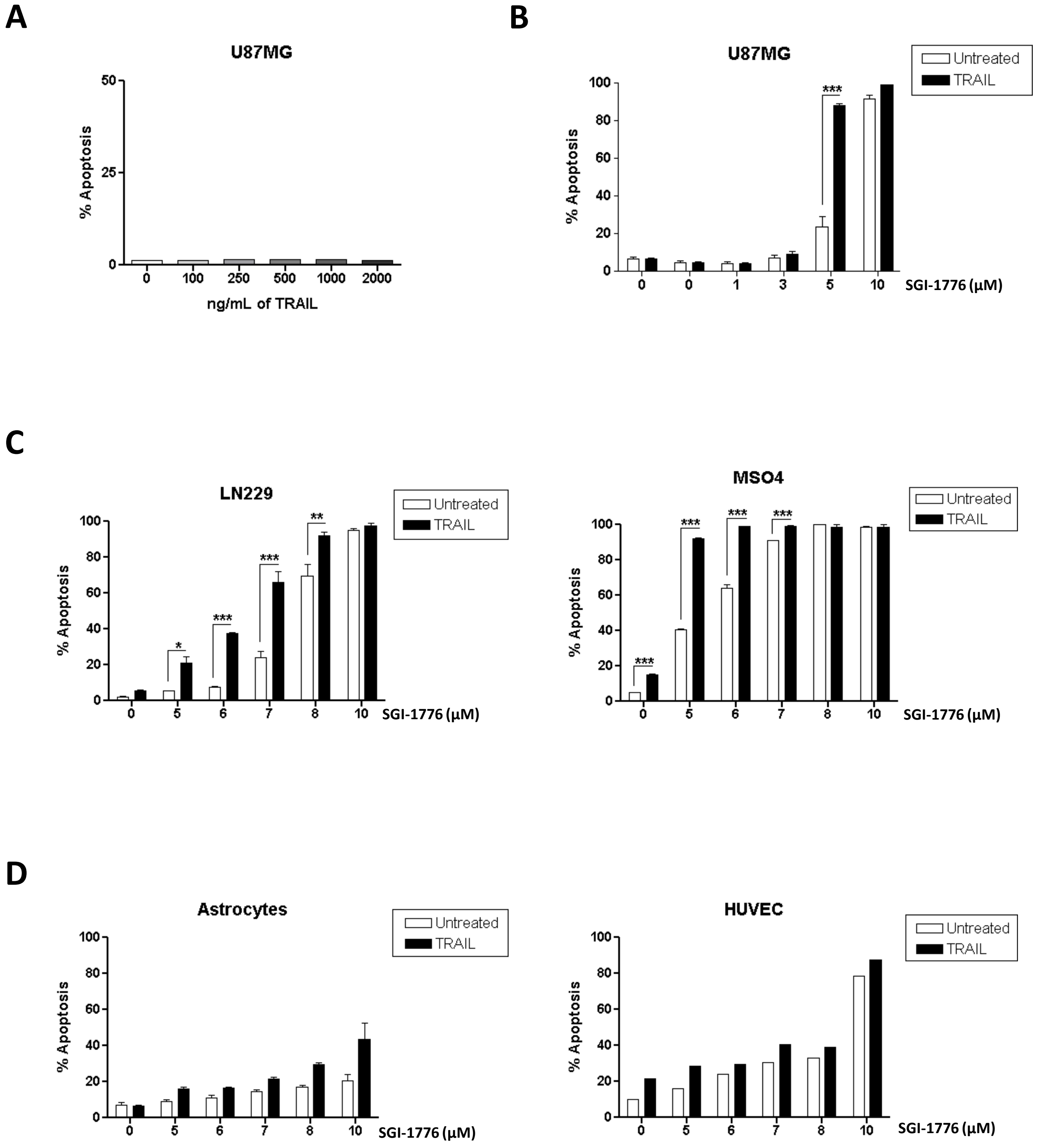
A
U87MG
% Apoptosis
50
25
0
0 100 250 500 1000 2000
ng/mL of TRAIL
B
U87MG
Untreated
TRAIL
% Apoptosis
100
80
60
40
20
0
***
0 0 1 3 5 10
SGI-1776 (μM)
C
LN229
Untreated
TRAIL
% Apoptosis
100
80
60
40
20
0
* *** *** **
0 5 6 7 8 10
SGI-1776 (μM)
MSO4
Untreated
TRAIL
% Apoptosis
100
80
60
40
20
0
*** *** *** ***
0 5 6 7 8 10
SGI-1776 (μM)
D
Astrocytes
Untreated
TRAIL
% Apoptosis
100
80
60
40
20
0
0 5 6 7 8 10
SGI-1776 (μM)
HUVEC
Untreated
TRAIL
% Apoptosis
100
80
60
40
20
0
0 5 6 7 8 10
SGI-1776 (μM)

Figure S1

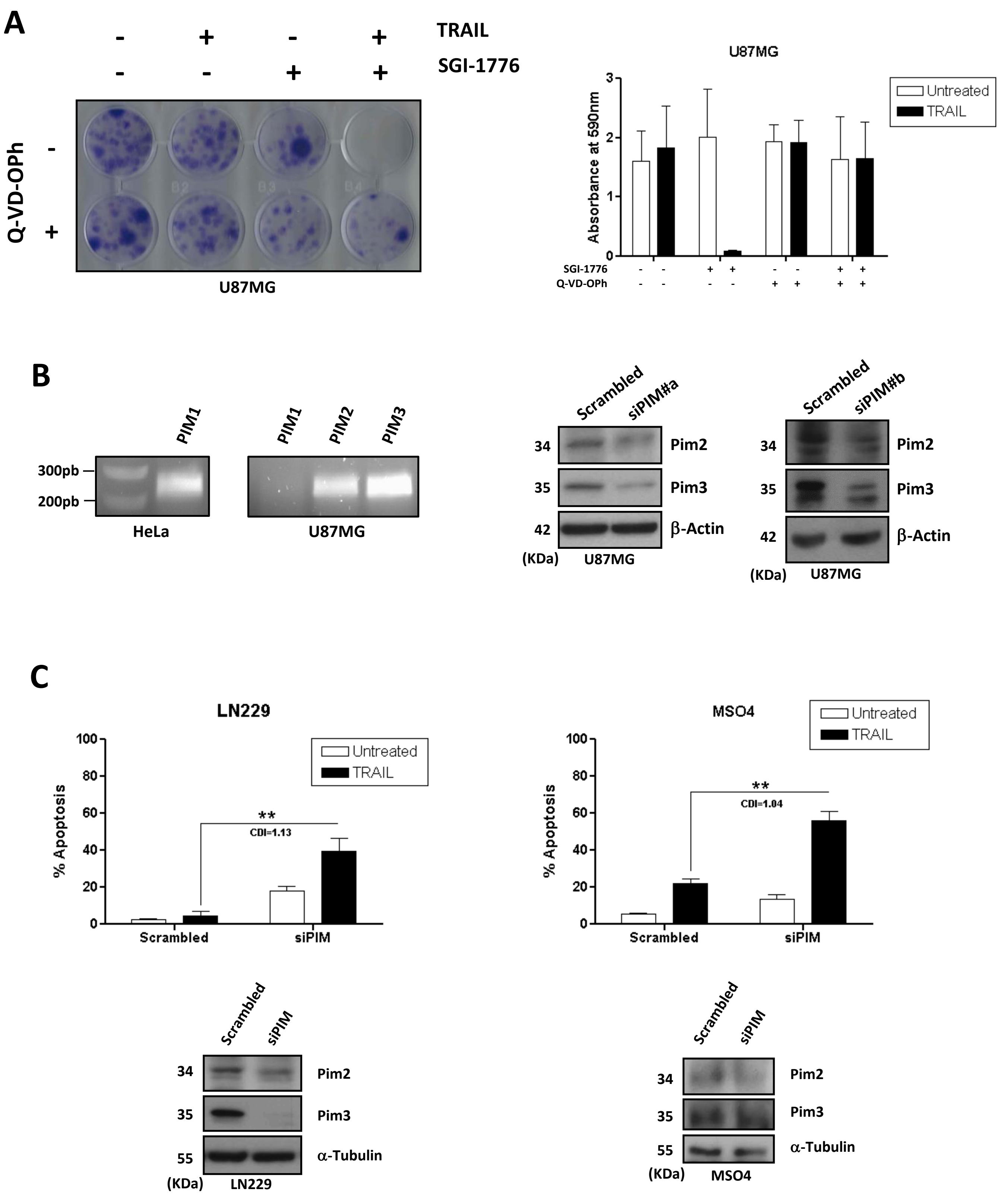
A
TRAIL
SGI-1776
Q-VD-OPh
U87MG
U87MG
Absorbance at 590nm
Untreated
TRAIL
SGI-1776
Q-VD-OPh
B
PIM1
PIM1
PIM2
PIM3
300pb
200pb
HeLa
U87MG
Scrambled
siPIM#a
Pim2
Pim3
β-Actin
(KDa)
U87MG
Scrambled
siPIM#b
Pim2
Pim3
β-Actin
(KDa)
U87MG
C
LN229
% Apoptosis
Untreated
TRAIL
CDI=1.13
Scrambled
siPIM
MSO4
% Apoptosis
Untreated
TRAIL
CDI=1.04
Scrambled
siPIM
Scrambled
siPIM
Pim2
Pim3
α-Tubulin
(KDa)
LN229
Scrambled
siPIM
Pim2
Pim3
α-Tubulin
(KDa)
MSO4

Figure S2

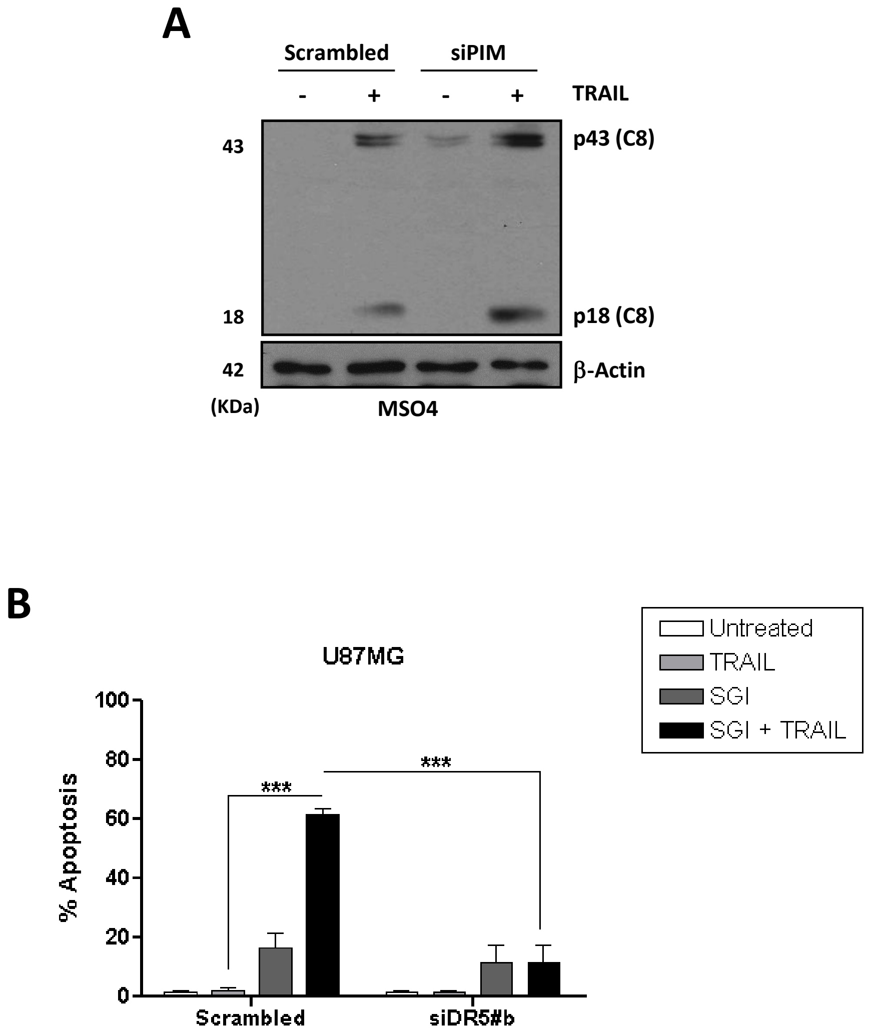
A
Scrambled
siPIM
- + - + TRAIL
43
p43 (C8)
18
p18 (C8)
42
β-Actin
(KDa)
MSO4
B
U87MG
Untreated
TRAIL
SGI
SGI + TRAIL
% Apoptosis
100
80
60
40
20
0
***
***
Scrambled
siDR5#b

Figure S3

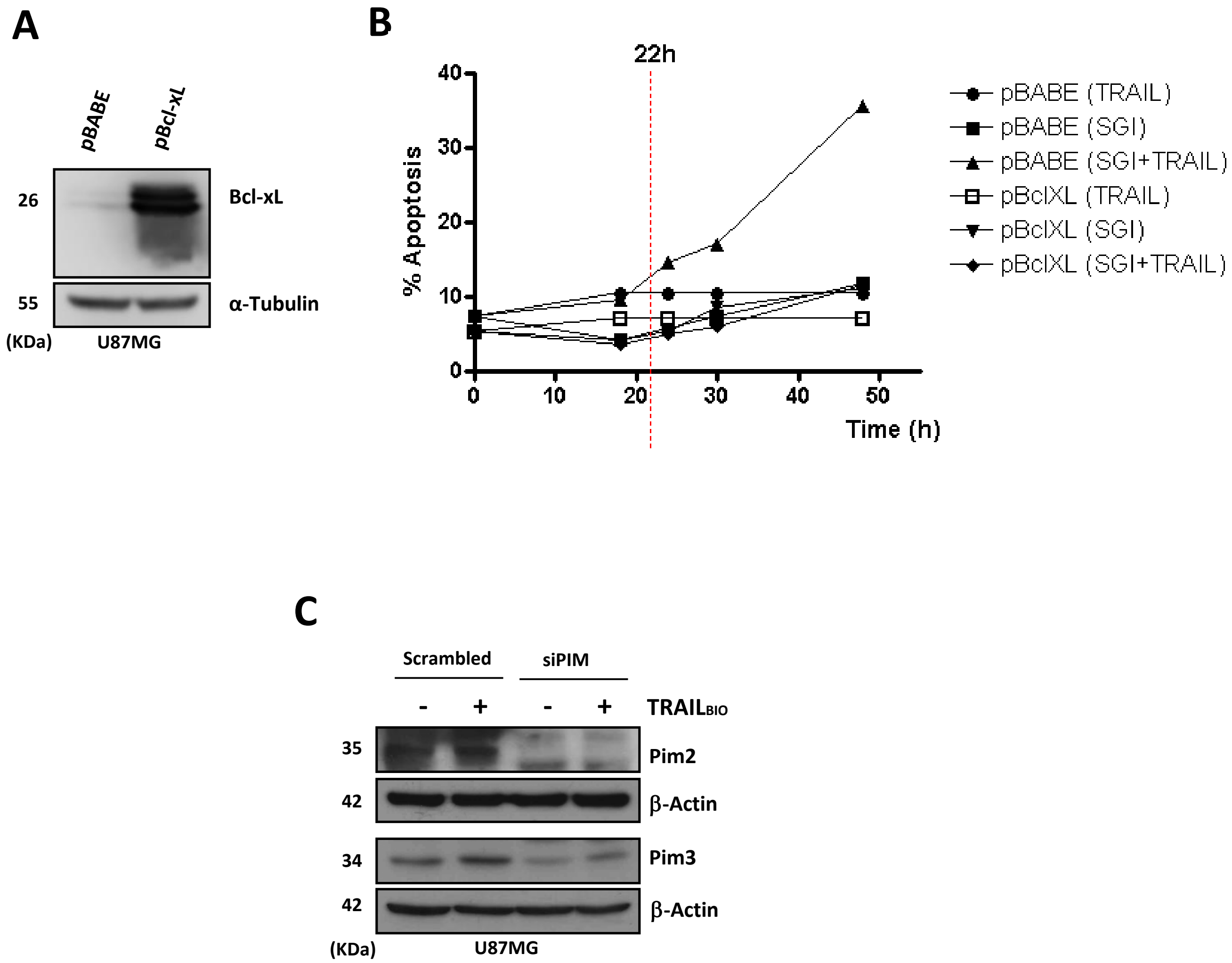
A
pBABE
pBcl-xL
26
Bcl-xL
55
α-Tubulin
(KDa)
U87MG
B
22h
40
30
20
10
0
% Apoptosis
0
10
20
30
40
50
Time (h)
pBABE (TRAIL)
pBABE (SGI)
pBABE (SGI+TRAIL)
pBclXL (TRAIL)
pBclXL (SGI)
pBclXL (SGI+TRAIL)
C
Scrambled
siPIM
- + - +
TRAILBIO
35
Pim2
42
β-Actin
34
Pim3
42
β-Actin
(KDa)
U87MG

Figure S4

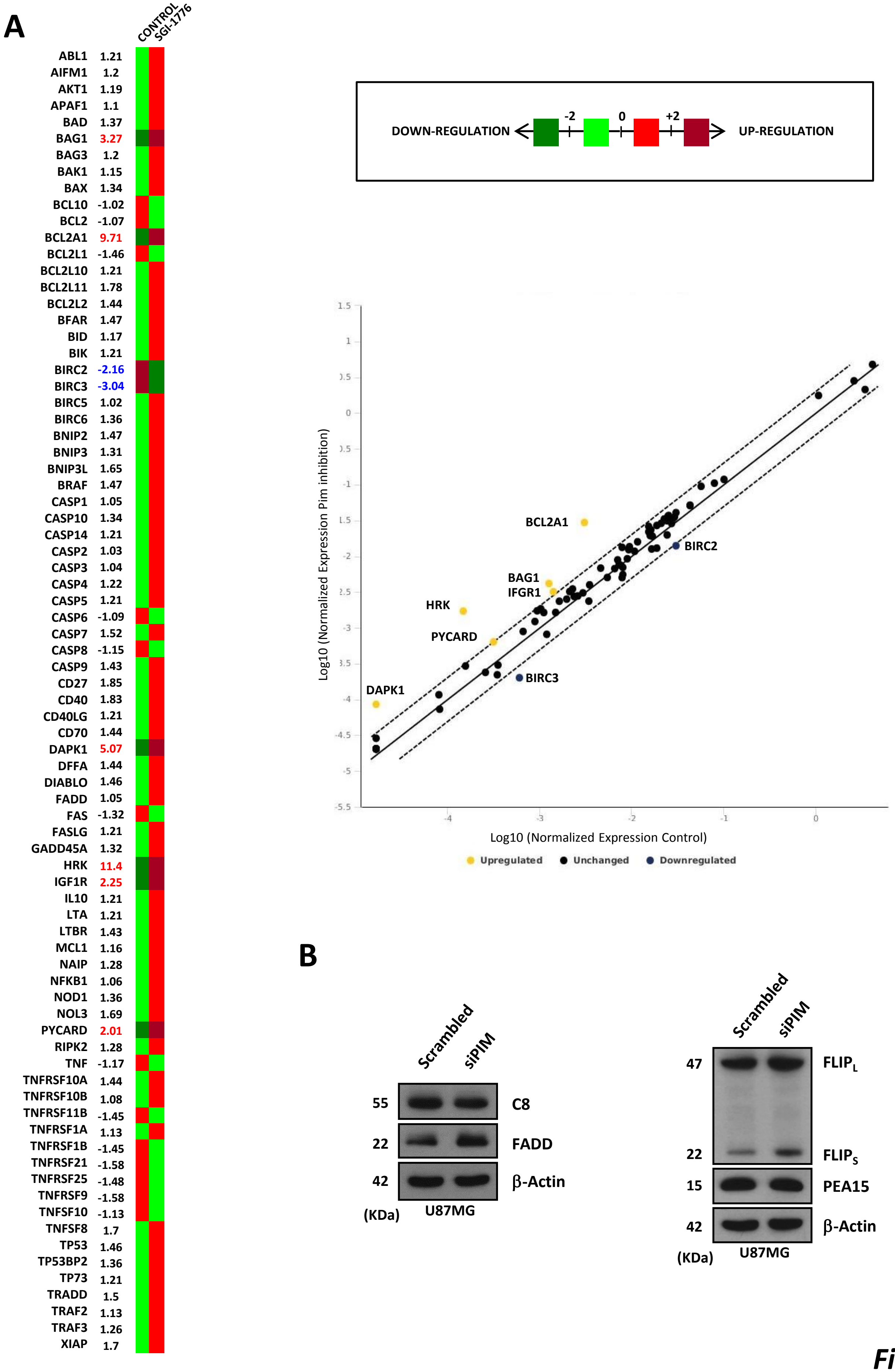
A
CONTROL
SGI-1776
ABL1 1.21
AIFM1 1.2
AKT1 1.19
APAF1 1.1
BAD 1.37
BAG1 3.27
BAG3 1.2
BAK1 1.15
BAX 1.34
BCL10 -1.02
BCL2 -1.07
BCL2A1 9.71
BCL2L1 -1.46
BCL2L10 1.21
BCL2L11 1.78
BCL2L2 1.44
BFAR 1.47
BID 1.17
BIK 1.21
BIRC2 -2.16
BIRC3 -3.04
BIRC5 1.02
BIRC6 1.36
BNIP2 1.47
BNIP3 1.31
BNIP3L 1.65
BRAF 1.47
CASP1 1.05
CASP10 1.34
CASP14 1.21
CASP2 1.03
CASP3 1.04
CASP4 1.22
CASP5 1.21
CASP6 -1.09
CASP7 1.52
CASP8 -1.15
CASP9 1.43
CD27 1.85
CD40 1.83
CD40LG 1.21
CD70 1.44
DAPK1 5.07
DFFA 1.44
DIABLO 1.46
FADD 1.05
FAS -1.32
FASLG 1.21
GADD45A 1.32
HRK 11.4
IGF1R 2.25
IL10 1.21
LTA 1.21
LTBR 1.43
MCL1 1.16
NAIP 1.28
NFKB1 1.06
NOD1 1.36
NOL3 1.69
PYCARD 2.01
RIPK2 1.28
TNF -1.17
TNFRSF10A 1.44
TNFRSF10B 1.08
TNFRSF11B -1.45
TNFRSF1A 1.13
TNFRSF1B -1.45
TNFRSF21 -1.58
TNFRSF25 -1.48
TNFRSF9 -1.58
TNFSF10 -1.13
TNFSF8 1.7
TP53 1.46
TP53BP2 1.36
TP73 1.21
TRADD 1.5
TRAF2 1.13
TRAF3 1.26
XIAP 1.7
DOWN-REGULATION
-2
0
+2
UP-REGULATION
Log10 (Normalized Expression Pim inhibition)
Log10 (Normalized Expression Control)
BCL2A1
BIRC2
BAG1
IFGR1
HRK
PYCARD
BIRC3
DAPK1
Upregulated
Unchanged
Downregulated
B
Scrambled
siPIM
55
C8
22
FADD
42
β-Actin
(KDa)
U87MG
47
FLIP_L
22
FLIP_S
15
PEA15
42
β-Actin
(KDa)
U87MG

Figure S5

A

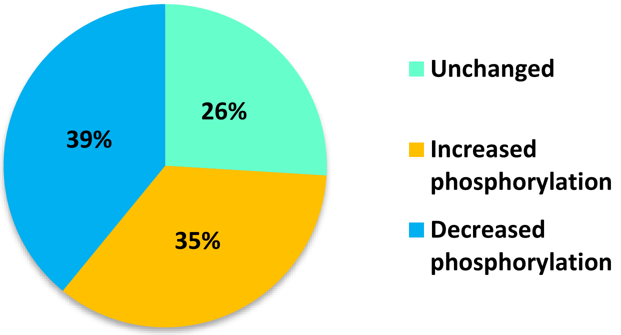

Summary

Total proteins detected: 1456 (Tabla S1)
Up-phosporylated proteins: 509 (35%)
Down-phosporylated proteins: 569 (39%)
Unchanged proteins: 378 (26%)

ΔPSM Threshold: 1.6

$$\Delta PSM_{DOWN\text{-}PHOSPHORYLATED} = PSM_{Scrambled} / PSM_{siPIM}$$

$$\Delta PSM_{UP\text{-}PHOSPHORYLATED} = PSM_{siPIM} / PSM_{Scrambled}$$

B

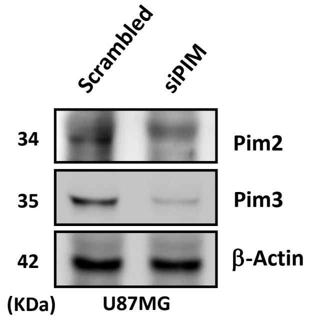

C

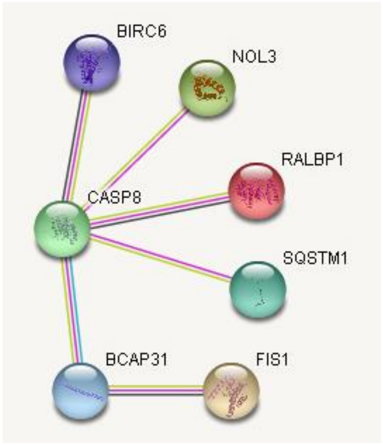

Figure S6

A

>SQSTM1/p62 Wild Type

ATGGCGTCGCTCACCGTGAAGGCCTACCTTCTGGGCAAGGAGGACGCGGCGCGCGAGATTCGCCGCTTCAGCTTCTGCTGCAGCCCCGAGCCTG
AGGCGGAAGCCGAGGCTGCGGCGGGTCCGGGACCCTGCGAGCGGCTGCTGAGCCGGGTGGCCGCCCTGTTCCCCGCGCTGCGGCCTGGCGGC
TTCCAGGCGCACTACCGCGATGAGGACGGGGACTTGGTTGCCTTTTCCAGTGACGAGGAATTGACAATGGCCATGTCCTACGTGAAGGATGACA
TCTTCCGAATCTACATTAAAGAGAAAAAAGAGTGCCGGCGGGACCACCGCCCACCGTGTGCTCAGGAGGCGCCCCGCAACATGGTGCACCCCAA
TGTGATCTGCGATGGCTGCAATGGGCCTGTGGTAGGAACCCGCTACAAGTGCAGCGTCTGCCCAGACTACGACTTGTGTAGCGTCTGCGAGGGA
AAGGGCTTGCACCGGGGGCACACCAAGCTCGCATTCCCCAGCCCCTTCGGGCACCTGTCTGAGGGCTTCTCGCACAGCCGCTGGCTCCGGAAGG
TGAAACACGGACACTTCGGGTGGCCAGGATGGGAAATGGGTCCACCAGGAAACTGGAGCCCACGTCCTCCTCGTGCAGGGGAGGCCCGCCCTG
GCCCCACGGCAGAATCAGCTTCTGGTCCATCGGAGGATCCGAGTGTGAATTTCCTGAAGAACGTTGGGGAGAGTGTGGCAGCTGCCCTTAGCCC
TCTGGGCATTGAAGTTGATATCGATGTGGAGCACGGAGGGAAAAGAAGCCGCCTGACCCCCGTCTCTCCAGAGAGTTCCAGCACAGAGGAGAA
GAGCAGCTCACAGCCAAGCAGCTGCTGCTCTGACCCCAGCAAGCCGGGTGGGAATGTTGAGGGCGCCACGCAGTCTCTGGCGGAGCAGATGAG
GAAGATCGCCTTGGAGTCCGAGGGGCGCCCTGAGGAACAGATGGAGTCGGATAACTGT**TCA**GGAGGAGATGATGACTGGACCCATCTGTCTTC
AAAAGAAGTGGACCCGTCTACAGGTGAACTCCAGTCCCTACAGATGCCAGAATCCGAAGGGCCAAGCTCTCTGGACCCCTCCCAGGAGGGACCC
ACAGGGCTGAAGGAAGCTGCCTTGTACCCACATCTCCCGCCAGAGGCTGACCCGCGGCTGATTGAGTCCCTCTCCCAGATGCTGTCCATGGGCTT
CTCTGATGAAGGCGGCTGGCTCACCAGGCTCCTGCAGACCAAGAACTATGACATCGGAGCGGCTCTGGACACCATCCAGTATTCAAAGCATCCC
CCGCCGTTGTGA

>SQSTM1/p62(S332E)

ATGGCGTCGCTCACCGTGAAGGCCTACCTTCTGGGCAAGGAGGACGCGGCGCGCGAGATTCGCCGCTTCAGCTTCTGCTGCAGCCCCGAGCCTG
AGGCGGAAGCCGAGGCTGCGGCGGGTCCGGGACCCTGCGAGCGGCTGCTGAGCCGGGTGGCCGCCCTGTTCCCCGCGCTGCGGCCTGGCGGC
TTCCAGGCGCACTACCGCGATGAGGACGGGGACTTGGTTGCCTTTTCCAGTGACGAGGAATTGACAATGGCCATGTCCTACGTGAAGGATGACA
TCTTCCGAATCTACATTAAAGAGAAAAAAGAGTGCCGGCGGGACCACCGCCCACCGTGTGCTCAGGAGGCGCCCCGCAACATGGTGCACCCCAA
TGTGATCTGCGATGGCTGCAATGGGCCTGTGGTAGGAACCCGCTACAAGTGCAGCGTCTGCCCAGACTACGACTTGTGTAGCGTCTGCGAGGGA
AAGGGCTTGCACCGGGGGCACACCAAGCTCGCATTCCCCAGCCCCTTCGGGCACCTGTCTGAGGGCTTCTCGCACAGCCGCTGGCTCCGGAAGG
TGAAACACGGACACTTCGGGTGGCCAGGATGGGAAATGGGTCCACCAGGAAACTGGAGCCCACGTCCTCCTCGTGCAGGGGAGGCCCGCCCTG
GCCCCACGGCAGAATCAGCTTCTGGTCCATCGGAGGATCCGAGTGTGAATTTCCTGAAGAACGTTGGGGAGAGTGTGGCAGCTGCCCTTAGCCC
TCTGGGCATTGAAGTTGATATCGATGTGGAGCACGGAGGGAAAAGAAGCCGCCTGACCCCCGTCTCTCCAGAGAGTTCCAGCACAGAGGAGAA
GAGCAGCTCACAGCCAAGCAGCTGCTGCTCTGACCCCAGCAAGCCGGGTGGGAATGTTGAGGGCGCCACGCAGTCTCTGGCGGAGCAGATGAG
GAAGATCGCCTTGGAGTCCGAGGGGCGCCCTGAGGAACAGATGGAGTCGGATAACTGT**GAA**GGAGGAGATGATGACTGGACCCATCTGTCTTC
AAAAGAAGTGGACCCGTCTACAGGTGAACTCCAGTCCCTACAGATGCCAGAATCCGAAGGGCCAAGCTCTCTGGACCCCTCCCAGGAGGGACCC
ACAGGGCTGAAGGAAGCTGCCTTGTACCCACATCTCCCGCCAGAGGCTGACCCGCGGCTGATTGAGTCCCTCTCCCAGATGCTGTCCATGGGCTT
CTCTGATGAAGGCGGCTGGCTCACCAGGCTCCTGCAGACCAAGAACTATGACATCGGAGCGGCTCTGGACACCATCCAGTATTCAAAGCATCCC
CCGCCGTTGTGA

B

```
p62-wt          MASLTVKAYLLGKEDAAREIRRFSFCCSPEPEAEAEAAAGPGPCERLLSRVAALFPALRP      60
p62-S332E       MASLTVKAYLLGKEDAAREIRRFSFCCSPEPEAEAEAAAGPGPCERLLSRVAALFPALRP      60
                ************************************************************

p62-wt          GGFQAHYRDEDGDLVAFSSDEELTMAMSYVKDDIFRIYIKEKKECRRDHRPPCAQEAPRN      120
p62-S332E       GGFQAHYRDEDGDLVAFSSDEELTMAMSYVKDDIFRIYIKEKKECRRDHRPPCAQEAPRN      120
                ************************************************************

p62-wt          MVHPNVICDGCNGPVVGTRYKCSVCPDYDLCSVCEGKGLHRGHTKLAFPSPFGHLSEGFS      180
p62-S332E       MVHPNVICDGCNGPVVGTRYKCSVCPDYDLCSVCEGKGLHRGHTKLAFPSPFGHLSEGFS      180
                ************************************************************

p62-wt          HSRWLRKVKHGHFGWPGWEMGPPGNWSPRPPRAGEARPGPTAESASGPSEDPSVNFLKNV      240
p62-S332E       HSRWLRKVKHGHFGWPGWEMGPPGNWSPRPPRAGEARPGPTAESASGPSEDPSVNFLKNV      240
                ************************************************************

p62-wt          GESVAAALSPLGIEVDIDVEHGGKRSRLTPVSPESSSTEEKSSSQPSSCCSDPSKPGGNV      300
p62-S332E       GESVAAALSPLGIEVDIDVEHGGKRSRLTPVSPESSSTEEKSSSQPSSCCSDPSKPGGNV      300
                ************************************************************

p62-wt          EGATQSLAEQMRKIALESEGRPEEQMESDNCSGGDDDWTHLSSKEVDPSTGELQSLQMPE      360
p62-S332E       EGATQSLAEQMRKIALESEGRPEEQMESDNCEGGDDDWTHLSSKEVDPSTGELQSLQMPE      360
                *******************************.****************************

p62-wt          SEGPSSLDPSQEGPTGLKEAALYPHLPPEADPRLIESLSQMLSMGFSDEGGWLTRLLQTK      420
p62-S332E       SEGPSSLDPSQEGPTGLKEAALYPHLPPEADPRLIESLSQMLSMGFSDEGGWLTRLLQTK      420
                ************************************************************

p62-wt          NYDIGAALDTIQYSKHPPPL          440
p62-S332E       NYDIGAALDTIQYSKHPPPL          440
                ********************
```

Figure S7

		Scrambled			siPIM		
Name	Accession	Coverage	Peptides	PSM	Coverage	Peptides	PSM
1433B	P31946	12,30	2	7	12,30	2	5
1433E	P62258	16,31	3	4	8,15	2	2
1433F	Q04917	15,04	3	3	10,98	2	4
1433G	P61981	11,34	2	8	11,34	2	8
1433T	P27348	12,65	2	13	18,37	3	6
1433Z	P63104	8,98	2	2	18,37	3	5
1A68	P01891	13,70	3	4	13,70	3	6
1B07	P01889	5,52	1	1	5,52	1	1
2A5G	Q13362				2,47	1	1
2ABA	P63151	3,36	1	4	3,36	1	3
3BP5L	Q7L8J4	8,86	2	4	8,86	2	3
4EBP1	Q13541	30,51	2	3			
4EBP2	Q13542	25,83	1	1			
4ET	Q9NRA8	3,21	1	1	4,32	2	4
6PGD	P52209	2,34	1	1			
AAK1	Q2M2I8	1,27	1	4	1,62	2	7
AAKB1	Q9Y478	10,00	1	2	10,00	1	5
AAKB2	O43741	9,47	1	3			
AAPK1	Q13131	3,22	1	5	3,22	1	3
AB1IP	Q7Z5R6	2,10	1	5	2,10	1	4
ABCA1	O95477	1,11	1	2	1,55	2	3
ABCA4	P78363	1,06	1	1			
ABCF1	Q8NE71	12,19	6	18	10,06	5	35
ABI1	Q8IZP0	3,14	1	2	3,14	1	1
ABI2	Q9NYB9				2,74	1	1
ABL1	P00519	2,83	2	3	1,06	1	1
ABL2	P42684	1,15	1	1	1,15	1	1
ABLM1	O14639	10,72	3	6	9,23	3	5
ABLM3	O94929	4,96	2	3	10,29	5	7
ACAP2	Q15057				2,06	1	2
ACBD5	Q5T8D3				7,21	2	3
ACINU	Q9UKV3	1,28	1	1	2,33	2	2
ACLY	P53396	2,05	2	4	2,05	3	11
ACOD	O00767				4,18	1	2
ACPH	P13798	1,78	1	1	1,78	1	1
ACSA	Q9NR19	1,43	1	3	1,43	1	4
ACTB	P60709	23,20	8	11	12,00	5	11
ACTH	P63267	16,49	6	7	5,85	3	8
ACTN1	P12814	1,69	1	5	1,24	1	1
ACTN4	O43707	3,76	2	6	1,59	1	1
ADA17	P78536	1,46	1	1			
ADAM9	Q13443	1,10	1	1			
ADDA	P35611	8,08	5	6	4,75	4	6
ADDG	Q9UEY8	3,12	2	7	3,12	2	8
ADNP	Q9H2P0	1,45	1	1	1,72	1	2
ADRM1	Q16186				2,95	1	1
AFAD	P55196	1,09	1	1	3,44	4	5
AFF4	Q9UHB7				4,53	1	2
AGM1	O95394	3,69	1	2	3,69	1	1
AGRG1	Q9Y653				3,86	2	2
AHNK	Q09666	3,06	21	77	3,06	21	80
AHNK2	Q8IVF2	1,16	5	19	0,54	2	27
AIFM1	O95831				4,98	1	1
AIM1	Q9Y4K1	1,74	1	4	1,28	1	1
AKA11	Q9UKA4	4,00	5	12	2,10	3	3
AKA12	Q02952	16,88	16	52	12,64	12	37
AKAP2	Q9Y2D5	9,39	4	7	5,38	3	4
AKAP8	O43823				2,60	1	1
AKIB1	Q9P2G1				1,19	1	1
AKNA	Q7Z591	1,44	1	1			
AKP13	Q12802	0,39	1	2	0,39	1	1
AKT1	P31749				5,02	1	4
AKTS1	Q96B36	23,02	3	3	23,02	4	8
ALBU	P02768	6,47	2	2			
ALDOA	P04075	4,12	2	4	3,85	1	1
ALG3	Q92685	2,74	1	2	2,74	1	4
ALKB5	Q6P6C2	4,70	1	2	10,97	2	7
AMPB	Q9H4A4	1,38	1	1			
AMPD2	Q01433	1,59	1	2	1,59	1	3
AMRP	P30533	2,24	1	1	4,76	2	3

AN34A	Q69YU3	5,24	2	4	5,24	2	3
ANCHR	Q96K21	3,97	1	3	6,70	2	3
ANK2	Q01484	2,20	2	3	1,66	2	2
ANKY2	Q8IV38	8,16	1	2			
ANR40	Q6AI12				1,90	1	1
ANR52	Q8NB46	2,04	1	1			
ANS1A	Q92625	1,15	1	1	1,15	1	6
ANX11	P50995				2,54	1	1
ANXA1	P04083	15,03	5	5	14,45	5	8
ANXA2	P07355	22,71	9	14	7,67	3	5
ANXA4	P09525	2,19	1	1	2,19	1	1
ANXA7	P20073	3,65	1	1			
AP2A2	O94973	1,37	1	1			
AP2B1	P63010				1,25	1	1
AP3B1	O00203	2,11	2	2	2,11	2	3
AP3B2	Q13367	2,48	1	2	2,48	1	3
AP3D1	O14617	4,77	3	10	4,77	3	8
APC4	Q9UJX5	3,47	1	2			
APEX1	P27695	6,60	1	2	6,60	1	1
API5	Q9BZZ5	3,20	1	2			
AR6P4	Q66PJ3	7,08	1	1	7,08	1	1
ARF3	P61204	6,94	1	1	12,50	2	2
ARFG1	Q8N6T3	10,24	3	5	9,90	2	3
ARFG2	Q8N6H7	5,16	1	2			
ARFG3	Q9NP61	3,60	1	3	3,60	1	1
ARH40	Q8TER5	1,49	1	1	1,49	1	2
ARHG2	Q92974	5,32	2	10	5,74	3	13
ARHG6	Q15052	7,56	5	7	7,56	6	9
ARHG7	Q14155	6,81	5	7	7,28	5	8
ARHGA	O15013	2,83	2	3	2,83	2	4
ARHGB	O15085	1,71	2	3	3,15	3	5
ARHGC	Q9NZN5				1,31	1	1
ARI3A	Q99856				4,89	1	3
ARM10	Q8N2F6	10,67	1	5	10,67	1	5
ARMX3	Q9UH62	3,96	1	1	3,96	1	1
ARNT	P27540	1,94	1	1	1,94	1	1
ARP19	P56211	11,46	1	1	11,46	1	2
ARP21	Q9UBL0	1,60	1	1			
ARP3	P61158	3,83	1	1			
ARPC5	O15511	23,18	1	1			
ASAP2	O43150	2,39	1	2	2,39	1	2
ASML	O95671	4,62	1	2	4,62	1	2
ASND1	Q9NWL6				3,42	1	1
ASPP2	Q13625	1,00	2	2	1,00	2	3
ASTN1	O14525	1,89	1	1			
AT1A1	P05023	2,64	3	5	1,47	2	3
AT2B1	P20020	2,03	2	6	2,03	1	5
AT2B4	P23634	4,90	3	4	3,48	2	4
ATAD1	Q8NBU5				7,20	1	1
ATG2A	Q2TAZ0	0,57	1	1			
ATG2B	Q96BY7	2,21	2	2			
ATG9A	Q7Z3C6	5,36	3	7	2,50	2	8
ATIF1	Q9UII2	11,32	3	8	9,43	1	1
ATLA1	Q8WXF7	4,52	2	3	1,99	1	2
ATLA3	Q6DD88	3,88	2	2	3,88	2	2
ATN1	P54259				3,70	2	3
ATX2	Q99700	3,42	1	1			
ATX2L	Q8WWM7	8,16	4	6	1,34	1	1
AUXI	O75061				1,44	2	2
AXDN1	Q5T1B0	3,39	1	1			
B3A2	P04920	2,53	1	1	2,53	1	3
BABA1	Q9NWV8	21,64	2	4	15,20	1	2
BAD	Q92934	20,24	3	4	20,24	3	5
BAG3	O95817	6,09	2	6	8,70	3	10
BAG6	P46379	4,18	2	2	2,23	1	2
BAIP2	Q9UQB8	1,95	1	2	1,95	1	2
BAP29	Q9UHQ4	6,64	1	7	6,64	1	2
BAP31	P51572	6,10	1	1			
BASI	P35613	10,23	1	2	15,34	3	4
BASP1	P80723	29,48	2	2	39,88	3	6
BCL7B	Q9BQE9				18,31	1	2
BCL9L	Q86UU0	0,86	1	1			

BCLF1	Q9NYF8	5,98	3	3	7,48	4	4
BCR	P11274	3,50	2	5	6,28	3	6
BD1L1	Q8NFC6				1,61	3	3
BET1L	Q9NYM9	11,71	1	1	11,71	1	1
BICC1	Q9H694	1,93	1	1	1,93	1	1
BICD2	Q8TD16	1,46	1	2	2,43	2	3
BIG2	Q9Y6D5	0,78	1	1	1,96	2	5
BIG3	Q5TH69	0,60	1	1	2,48	3	4
BIN1	O00499	4,65	2	13	4,65	2	16
BIRC6	Q9NR09	0,39	1	3	0,39	1	1
BL1S3	Q6QNY0	11,39	1	2	11,39	1	4
BNI3L	O60238	12,29	2	10	12,29	2	14
BNIP2	Q12982	4,14	1	1	5,41	2	4
BNIP3	Q12983	15,46	2	2	21,65	3	3
BOLA2	Q9H3K6	27,59	1	2	27,59	1	1
BOP1	Q14137	2,52	1	1	2,52	1	1
BORG4	Q9H3Q1	10,14	1	1			
BORG5	Q00587	4,95	2	2	2,08	1	1
BRAF	P15056	2,48	1	7	3,66	2	4
BRE1A	Q5VTR2	1,85	2	4	5,64	3	11
BROX	Q5VW32	4,22	1	1	6,33	1	1
BYST	Q13895	4,12	1	3	4,12	1	6
C170B	Q9Y4F5	1,18	1	1			
C2CD5	Q86YS7				2,80	2	4
C2D1A	Q6P1N0	1,37	1	1	3,16	2	2
C2D1B	Q5T0F9	2,44	1	1			
C43BP	Q9Y5P4	2,88	1	6	2,88	1	1
CAD11	P55287				1,88	1	1
CAH9	Q16790	2,40	1	1	2,40	1	1
CALD1	Q05682	9,86	3	3	6,81	3	5
CALD1	Q05682	10,53	4	7	7,33	4	6
CALD1	Q05682	10,84	4	5	3,28	2	2
CALM	P62158	8,72	1	1	16,78	2	3
CALR	P27797	12,95	5	26	12,95	5	10
CALU	O43852	27,30	6	20	14,92	3	8
CALX	P27824	15,03	9	29	13,85	8	36
CAMP1	Q5T5Y3	0,83	1	1			
CAMP2	Q08AD1	1,92	2	4	3,21	3	6
CAND1	Q86VP6	1,13	1	1	1,13	2	2
CAP1	Q01518	2,32	1	1			
CAPS1	Q9ULU8	5,25	2	4	5,25	2	3
CAPSL	Q8WWF8	5,29	1	1			
CAPZB	P47756	3,31	1	1	3,31	1	1
CARL1	Q5VZK9	1,13	1	1	3,09	4	7
CASC3	O15234	3,13	1	1	8,53	3	7
CASC4	Q6P4E1	5,53	1	2			
CASP2	P42575				3,76	1	3
CASP9	P55211	12,03	1	7			
CATD	P07339	4,37	1	1	4,37	1	1
CAV1	Q03135	9,55	1	1	9,55	1	1
CBARP	Q8N350	2,65	1	1	2,65	1	1
CBLB	Q13191				2,08	1	2
CBPD	O75976	1,94	1	2	1,94	1	2
CBX3	Q13185	7,10	1	3	8,20	3	8
CBX4	O00257				2,68	1	1
CC120	Q96HB5	3,81	1	1			
CCAR1	Q8IX12	2,29	1	1			
CCD18	Q5T9S5				0,54	1	1
CCD22	O60826	1,75	1	1			
CCD25	Q86WR0	12,14	1	2	12,14	1	3
CCD43	Q96MW1	15,63	1	1			
CCD61	Q9Y6R9				4,82	1	1
CCD86	Q9H6F5	6,39	1	2	5,83	1	1
CCD97	Q96F63				6,71	1	2
CCDC6	Q16204	2,53	1	3	5,70	2	4
CCNK	O75909	3,08	1	1	3,08	1	1
CCNL1	Q9UK58				2,80	1	2
CCNY	Q8ND76				3,83	1	2
CCPG1	Q9ULG6				4,95	1	3
CD276	Q5ZPR3	4,11	1	1	12,97	2	3
CD2AP	Q9Y5K6	3,60	2	2	4,85	3	5
CD2B2	O95400	3,23	1	2	5,28	1	1

CD44	P16070	2,94	1	1	13,24	3	5
CD97	P48960				1,21	1	1
CDC26	Q8NHZ8	28,24	1	1	28,24	1	1
CDC37	Q16543	5,56	1	2	5,56	1	1
CDC6	Q99741				3,57	1	1
CDK1	P06493	4,58	1	1	4,58	1	1
CDK12	Q9NYV4	0,79	1	1	1,02	1	1
CDK13	Q14004				0,76	1	1
CDK7	P50613	2,60	1	1	4,62	2	2
CDS1	Q92903				5,21	1	2
CDS2	O95674	11,24	2	10	11,24	2	7
CDV3	Q9UKY7	7,04	1	1			
CE170	Q5SW79	8,40	8	20	8,02	9	16
CE350	Q5VT06				0,48	1	1
CETN2	P41208	9,88	1	1			
CF203	Q9P0P8				12,50	1	1
CH60	P10809	2,09	1	1			
CHAP1	Q96JM3	7,02	3	4	7,14	3	3
CHD4	Q14839	1,67	1	1			
CHIP	Q9UNE7	9,90	2	5	5,94	1	3
CHM2B	Q9UQN3	5,81	1	1	5,81	1	1
CHMP7	Q8WUX9	2,65	1	3	2,65	1	1
CHSP1	Q9Y2V2	19,05	3	5	19,05	3	6
CI016	Q9BUW7	19,28	1	2			
CI040	Q8IXQ3	7,73	1	1			
CI078	Q9NZ63	5,54	1	4	11,76	2	4
CIC	Q96RK0				1,06	1	2
CIP4	Q15642	5,28	1	5	5,28	1	9
CK084	Q9BUA3	6,56	1	9	13,65	2	2
CK096	Q7Z7L8				2,07	1	1
CKAP5	Q14008	1,42	1	7	1,42	1	8
CLAP1	Q7Z460	2,11	2	8	2,11	2	12
CLAP2	O75122	3,23	3	3	4,95	5	9
CLCB	P09497	3,32	1	1	3,32	1	2
CLCN7	P51798	3,23	1	2			
CLIC1	O00299	4,98	1	1	4,98	1	1
CLIC4	Q9Y696	13,44	2	5	4,74	1	1
CLIP1	P30622	2,37	3	5	2,37	3	5
CLK3	P49761				19,74	2	3
CLN3	Q13286				6,32	2	3
CLN6	Q9NWW5	3,54	1	1	3,54	1	1
CLSPN	Q9HAW4	2,35	1	1			
CLUA1	Q96AJ1				4,86	1	1
CMBL	Q96DG6	6,94	1	2			
CND3	Q9BPX3	4,83	3	7	4,83	3	11
CNPY2	Q9Y2B0	7,14	1	2	7,14	1	1
COBL1	Q53SF7				1,24	1	1
COF1	P23528	8,43	1	4	15,06	2	6
COPA	P53621	2,04	2	2	0,98	1	2
COPB	P53618	1,47	1	1	1,47	1	1
COPB2	P35606	4,68	2	3	5,93	3	8
COPE	O14579	3,52	1	1			
CPNE7	Q9UBL6				2,69	1	2
CPSF7	Q8N684	3,46	1	1			
CQ059	Q96GS4	7,28	1	1			
CR025	Q96B23	9,65	2	3	9,94	3	6
CRBG3	Q68DQ2	0,67	1	1	0,81	1	2
CRCM	P23508	2,05	1	2			
CREL2	Q6UXH1				3,87	1	2
CRIP2	P52943	7,69	1	2			
CRK	P46108	17,16	2	9	17,16	2	7
CSF1	P09603	9,77	1	1	9,77	1	1
CSKI2	Q8WXE0				2,14	1	2
CSKP	O14936				3,09	1	2
CSN1	Q13098				3,90	1	1
CSN2	P61201	3,84	1	1			
CSRP1	P21291	8,81	1	1			
CSTF3	Q12996	3,35	1	1			
CT027	Q9GZN8				12,07	1	1
CT2NL	Q9P2B4	2,03	1	1	2,03	1	1
CTDP1	Q9Y5B0				1,27	1	1
CTGE5	O15320				2,68	1	1

CTIF	O43310				1,51	1	1
CTNA1	P35221	3,54	1	4	6,90	2	8
CTND1	O60716	4,58	2	3	4,58	2	4
CTR9	Q6PD62	1,79	2	2	3,50	3	4
CTRO	O14578				0,84	1	1
CTU2	Q2VPK5				3,27	1	2
CX038	Q8TB03	6,50	1	1			
CXA1	P17302				4,19	1	1
CYTB	P04080	12,24	1	1	9,18	1	1
CYTSA	Q69YQ0	2,88	2	5	2,88	2	4
DAB2	P98082	1,60	1	1			
DAP1	P51397	27,45	1	1			
DAXX	Q9UER7	2,41	1	2	2,41	1	1
DBNL	Q9UJU6	7,03	2	3	3,67	1	3
DC1I2	Q13409	2,29	1	2	2,29	1	1
DC1L1	Q9Y6G9	3,63	2	7	7,27	2	4
DC1L2	O43237	4,58	2	7	4,58	2	9
DCAF6	Q58WW2				2,44	1	4
DCAF8	Q5TAQ9				7,69	1	1
DCBD2	Q96PD2	4,77	2	4	1,94	1	3
DCP1A	Q9NPI6	3,13	1	1	3,13	1	2
DDB2	Q92466	8,33	1	1	8,33	2	6
DDX20	Q9UHI6	2,67	1	1	2,67	1	2
DDX3X	O00571	1,55	1	1			
DDX42	Q86XP3	4,03	1	3			
DDX55	Q8NHQ9	16,43	2	2	6,28	1	2
DDX58	O95786	1,70	1	2	1,70	1	2
DEN1A	Q8TEH3	1,09	1	1	1,39	1	2
DEN4C	Q5VZ89	1,75	1	1	2,01	1	2
DENR	O43583	13,13	1	4	14,14	2	3
DEST	P60981				8,78	1	1
DHE3	P00367	2,15	1	1	2,15	1	1
DHX16	O60231	1,44	1	1	2,79	2	3
DHX34	Q14147	2,80	1	2	2,80	1	2
DHX57	Q6P158	7,16	2	2	7,16	2	2
DHX9	Q08211	2,13	1	1	2,13	1	2
DI3L2	Q8IYB7	3,05	1	3	3,05	1	5
DIAP1	O60610				1,36	1	1
DIDO1	Q9BTC0	1,07	1	1	3,39	3	4
DJC17	Q9NVM6				4,61	1	2
DKC1	O60832				3,89	1	3
DLG1	Q12959	3,14	1	3	3,14	1	2
DLG5	Q8TDM6	1,93	1	1	1,93	1	1
DLGP4	Q9Y2H0	4,64	1	4	4,64	1	5
DMXL1	Q9Y485				1,16	2	6
DMXL2	Q8TDJ6	0,92	1	2	0,88	1	1
DNJA1	P31689	3,78	1	1			
DNJB1	P25685	4,12	1	2	4,12	1	1
DNJB6	O75190				6,14	1	2
DNJC1	Q96KC8	8,48	2	3	8,84	3	5
DNJC2	Q99543	2,11	1	1	2,11	1	2
DNJC5	Q9H3Z4	10,18	1	2			
DNJC7	Q99615	3,42	1	3			
DNLI1	P18858	2,12	1	1	2,12	1	2
DNMBP	Q6XZF7				2,79	1	4
DNMT1	P26358	1,48	1	1	1,48	1	1
DOC10	Q96BY6	1,88	2	2	0,69	1	1
DOC11	Q5JSL3	1,25	1	3	1,25	1	3
DOCK5	Q9H7D0	0,43	1	1	1,02	2	2
DOCK7	Q96N67	2,02	4	19	2,68	5	21
DPOE3	Q9NRF9	17,69	1	2			
DPYL2	Q16555	5,41	2	2	6,72	3	5
DPYL3	Q14195	4,97	2	3			
DREB	Q16643	1,23	1	1	4,01	2	3
DSCR3	O14972				10,74	1	1
DTD1	Q8TEA8	7,66	1	3	7,66	1	3
DTNA	Q9Y4J8	3,58	1	1	3,58	1	1
DTX3L	Q8TDB6	2,30	1	1	2,30	1	1
DUS3L	Q96G46	4,98	1	2	4,98	1	4
DYHC1	Q14204	0,28	1	1	0,28	1	1
DYL1	P63167	24,72	1	2			
DYN1	Q05193	1,29	1	2	1,41	3	4

DYST	Q03001	0,89	4	5	0,52	3	4
DYST	Q03001	17,41	2	2	8,04	1	2
E41L1	Q9H4G0	11,14	5	5	8,94	3	9
E41L2	O43491				4,90	1	2
EAF1	Q96JC9	11,38	1	2	11,38	1	2
ECD	O95905	3,99	1	2			
EDC3	Q96F86	5,91	4	4	5,71	3	4
EDC4	Q6P2E9	2,84	1	9	2,84	1	3
EEPD1	Q7L9B9	2,99	1	1			
EF1A1	P68104	2,49	1	1	2,49	1	1
EF1B	P24534	17,33	4	20	17,33	4	22
EF1D	P29692	18,68	3	25	18,68	4	15
EF2	P13639	7,11	6	15	7,11	5	12
EF2K	O00418	3,31	1	2	5,38	3	6
EFHC1	Q5JVL4				6,12	1	1
EFHD2	Q96C19	12,08	3	4	11,67	2	2
EFTU	P49411	3,76	1	2	2,65	1	1
EGFR	P00533	1,57	1	1			
EGLN1	Q9GZT9	3,71	1	1			
EH1L1	Q8N3D4	2,76	2	3	1,25	1	1
EHBP1	Q8NDI1	1,55	1	5	1,55	1	5
EHD1	Q9H4M9				3,37	1	1
EHD2	Q9NZN4	5,65	1	2	5,65	1	3
EIF2A	Q9BY44	4,85	1	3	4,85	1	3
EIF3A	Q14152	1,78	2	2			
EIF3B	P55884	7,86	2	9	7,86	2	7
EIF3C	Q99613	1,55	1	2	1,55	1	3
EIF3F	O00303				1,96	1	1
ELF4	Q99607				2,26	1	2
EMAL1	O00423				1,60	2	2
EMAL4	Q9HC35	1,63	1	1	3,26	1	1
ENAH	Q8N8S7	4,13	1	1	4,13	1	1
ENL	Q03111				2,68	1	1
ENOA	P06733	15,44	6	11	7,37	3	4
ENOG	P09104	8,70	4	8	4,09	3	4
ENPL	P14625	5,85	3	3	1,49	1	1
EP300	Q09472	1,04	1	2	1,04	1	6
EPHB2	P29323	1,83	1	3	1,83	1	3
EPN2	O95208				3,30	1	2
EPS15	P42566	6,01	2	3	2,06	1	1
EPS8	Q12929	2,67	1	1	2,49	1	3
ERC6L	Q2NKX8				0,64	1	1
ERCC5	P28715	2,28	2	3	3,54	3	6
ERF	P50548	5,29	1	1	4,65	1	4
ERIC1	Q86X53	5,87	1	1	5,87	1	1
ERRFI	Q9UJM3	4,11	1	1	4,11	1	1
ESYT2	A0FGR8				1,12	1	1
EVA1A	Q9H8M9	7,24	1	2	7,24	1	2
EXOC1	Q9NV70	1,71	1	1			
EYA4	O95677	2,88	1	2	2,88	1	2
EZRI	P15311	2,05	1	1	2,05	1	2
F102A	Q5T9C2	4,13	1	1	4,13	1	1
F10A1	P50502	10,03	2	8	5,96	1	4
F10C1	Q70Z53	7,30	1	2	7,30	1	1
F1142	Q9NRY5				5,35	1	1
F117A	Q9C073	2,87	1	1			
F122A	Q96E09				3,14	1	1
F122B	Q7Z309	8,82	1	1	8,24	1	1
F134A	Q8NC44	2,76	1	1	2,76	1	1
F134C	Q86VR2	9,59	1	3	18,45	2	2
F195B	C9JLW8				23,71	1	1
F219A	Q8IW50	18,59	1	2			
F234A	Q9H0X4	3,15	1	1	3,15	1	1
F234B	A2RU67	6,75	1	2			
F91A1	Q658Y4				4,42	1	1
FA13A	O94988	3,14	1	3	3,14	1	7
FA20A	Q96MK3				3,33	1	1
FA21A	Q641Q2	9,47	8	17	4,02	5	17
FA53C	Q9NYF3	2,81	1	2	2,81	1	1
FA83G	A6ND36	1,89	1	1	1,89	1	1
FBN1	P35555	0,63	1	1			
FBP1L	Q5T0N5	5,48	1	1			

FBXW9	Q5XUX1				4,15	1	2
FCHO2	Q0JRZ9	2,19	2	2	1,16	1	1
FER	P16591	3,09	1	2	3,09	1	2
FETUA	P02765	3,54	2	5	3,54	2	6
FGD1	P98174	1,98	1	2			
FGD3	Q5JSP0	3,63	1	2			
FHOD1	Q9Y613	0,86	1	1	0,86	1	1
FIL1L	Q4L180				2,11	1	1
FIP1	Q6UN15	2,88	1	3	3,65	3	8
FIS1	Q9Y3D6	10,53	1	1			
FKB15	Q5T1M5	1,08	1	3	1,08	1	6
FKBP4	Q02790	4,58	1	1			
FL2D	Q15007	9,60	1	2			
FLCN	Q8NFG4				9,14	1	1
FLII	Q13045				1,15	1	1
FLNA	P21333	0,91	2	2	2,24	6	7
FLNC	Q14315	2,01	5	7	1,93	6	7
FLOT1	O75955	3,69	1	1			
FMNL2	Q96PY5				0,64	1	1
FNBP1	Q96RU3	3,45	1	1	2,00	1	1
FNBP4	Q8N3X1				3,83	2	2
FOSL1	P15407	5,17	1	1			
FOXC2	Q99958	2,99	1	1	2,99	1	3
FOXK1	P85037	8,77	2	2			
FOXP4	Q8IVH2	3,30	1	1			
FPRP	Q9P2B2				0,80	1	1
FR1OP	O95684	6,86	1	6	6,86	2	5
FRIH	P02794	13,66	1	3			
FRM4A	Q9P2Q2				1,06	1	1
FRMD6	Q96NE9				3,41	1	1
FRYL	O94915	0,43	1	2	0,33	1	1
FUBP2	Q92945				3,09	2	3
FUND2	Q9BWH2				4,76	1	1
FXR1	P51114	4,66	1	1	4,66	1	2
FXR2	P51116	2,97	1	2	2,97	1	3
FYN	P06241	2,07	1	1	2,07	1	1
FYV1	Q9Y2I7	0,62	1	1	0,62	1	1
G3BP1	Q13283	9,66	2	22	3,86	1	12
G3BP2	Q9UN86	6,24	1	3			
G3P	P04406	13,99	3	3	5,12	1	1
G6PD	P11413	2,57	1	1	2,57	1	1
GA2L1	Q99501				2,20	1	1
GAK	O14976	2,52	1	2	5,28	2	8
GALT7	Q86SF2				3,04	1	3
GANAB	Q14697	5,69	3	4	6,42	4	5
GANAB	Q14697	6,04	3	5	6,78	4	6
GAPD1	Q14C86	0,77	1	1			
GBF1	Q92538	2,32	2	6	4,15	4	12
GCFC2	P16383	3,59	1	1	2,05	1	2
GDIR1	P52565	10,63	1	1			
GELS	P06396	2,05	1	3	2,05	1	3
GEM	P55040	6,08	2	2	3,38	1	2
GEPH	Q9NQX3	3,80	2	3			
GFPT1	Q06210	1,91	1	3	1,91	1	3
GFPT2	O94808	2,05	2	7	2,05	2	5
GGN	Q86UU5				4,60	1	1
GIT1	Q9Y2X7	8,54	4	15	6,70	3	13
GIT2	Q14161				3,96	1	10
GLCI1	Q86VQ1	3,84	1	2	5,85	2	4
GLU2B	P14314				2,10	1	2
GNL1	P36915				2,97	2	4
GOGA1	Q92805				3,39	1	2
GOGA2	Q08379	2,00	1	1			
GOGA4	Q13439	1,92	3	10	2,41	5	14
GOGB1	Q14789	0,38	1	1			
GOLI4	O00461	4,74	2	6	4,74	2	7
GOLM1	Q8NBJ4				4,60	1	1
GORS2	Q9H8Y8	6,25	1	3	6,25	1	1
GPN1	Q9HCN4	7,17	1	2	7,17	1	2
GPT2L	Q9NWQ4				5,13	1	2
GPTC2	Q9NW75				3,46	1	1
GPTC4	Q5T3I0	3,59	1	1			

GPTC8	Q9UKJ3	0,98	1	1	0,98	1	1
GRAP1	Q4V328	5,80	2	6			
GRB10	Q13322	1,68	1	1	1,68	1	1
GRDN	Q3V6T2	0,72	1	8	1,95	2	9
GREM1	O60565	11,96	1	2			
GRIN1	Q7Z2K8	2,26	1	2	2,26	1	2
GRIP1	Q9Y3R0				1,77	2	2
GRN	P28799	4,36	1	1			
GRP75	P38646	2,21	1	1			
GSK3A	P49840	8,90	2	8	8,90	2	9
GSLG1	Q92896	1,19	1	1			
GSTM1	P09488				6,63	1	1
GTPB1	O00178	4,04	1	2	4,04	1	2
GYS1	P13807	2,08	1	2	2,08	1	2
HACD3	Q9P035	3,31	1	2	3,31	1	1
HAP28	Q13442	10,50	4	6	10,50	4	9
HBS1L	Q9Y450	5,54	1	1			
HD	P42858				0,54	1	1
HDAC1	Q13547				3,73	1	1
HDAC2	Q92769	11,35	2	6	11,35	2	4
HDGF	P51858	15,88	6	18	20,17	6	28
HDGR2	Q7Z4V5	4,48	2	3	1,49	1	1
HDGR3	Q9Y3E1				20,20	1	1
HEAT3	Q7Z4Q2	5,29	1	2			
HECAM	Q14CZ8				4,63	1	1
HECD1	Q9ULT8	1,15	1	2	1,15	1	4
HECW1	Q76N89				0,83	1	2
HECW2	Q9P2P5	0,99	1	1			
HERC1	Q15751	0,51	1	1	0,70	2	4
HG2A	P04233	10,00	1	1	10,00	2	2
HIRP3	Q9BW71	8,09	3	6	11,69	5	7
HMGA1	P17096	19,79	1	4	19,79	1	9
HMGA2	P52926	15,12	1	3	15,12	1	3
HMGN1	P05114	14,00	1	1	14,00	1	1
HMGN2	P05204				21,11	1	1
HMOX1	P09601	3,82	1	1	3,82	1	1
HN1	Q9UK76	13,89	2	6	13,89	2	11
HNRH1	P31943	3,56	1	1	3,56	1	1
HNRL2	Q1KMD3	3,08	2	2	6,16	3	3
HNRLL	Q8WVV9	6,91	1	1			
HNRPD	Q14103	5,88	1	6	5,88	1	10
HNRPK	P61978	1,82	1	2	1,82	1	2
HNRPQ	O60506	1,90	1	1	1,90	1	1
HNRPU	Q00839	3,97	1	1			
HPS4	Q9NQG7				2,27	1	3
HPS5	Q9UPZ3	2,17	1	1	2,17	1	2
HRH1	P35367	2,26	1	2	2,26	1	2
HS105	Q92598	0,98	1	1	0,98	1	1
HS90A	P07900	17,62	12	23	11,75	9	15
HS90B	P08238	13,81	9	27	10,64	9	28
HSF1	Q00613	6,75	2	6	6,75	2	4
HSP74	P34932	11,31	4	9	6,19	2	6
HSP77	P48741	7,36	2	2	7,36	2	2
HSP7C	P11142	10,37	6	12	8,82	5	8
HSPB1	P04792	11,22	2	6	11,22	2	7
HSPB8	Q9UJY1	7,65	1	1			
HTSF1	O43719	9,54	4	9	17,35	6	12
HUWE1	Q7Z6Z7	1,33	3	3	1,77	4	10
HYEP	P07099	5,05	1	3			
HYPK	Q9NX55				12,94	1	1
I2BP1	Q8IU81	5,48	2	2	2,40	2	4
I2BP2	Q7Z5L9	2,63	2	4	5,25	4	6
I2BPL	Q9H1B7	1,88	1	2	5,40	3	5
IAH1	Q2TAA2	12,59	1	2	12,59	1	1
IBP3	P17936				7,22	1	2
ICAL	P20810	12,71	5	6			
ICLN	P54105	11,81	1	4	11,81	1	3
IF16	Q16666				4,01	1	1
IF2B	P20042	5,11	2	3			
IF2G	P41091				2,33	1	2
IF2P	O60841	5,66	6	16	7,38	10	29
IF3M	Q9H2K0	6,47	2	2			

IF4B	P23588	13,46	6	13	14,34	5	23
IF4G1	Q04637	2,28	3	4	3,14	5	7
IF4G3	O43432	2,27	3	4	2,97	4	7
IF4H	Q15056	3,51	1	2			
IF5	P55010	5,57	1	2	5,57	1	4
IF5A1	P63241	11,04	1	2			
IFT43	Q96FT9	10,09	1	1	10,09	2	3
IGBP1	P78318	6,78	2	3			
IL1AP	Q9NPH3				2,63	1	2
ILF3	Q12906	8,60	3	11	2,15	2	7
IMA1	P52292				3,21	1	2
IMA3	O00629	3,45	1	5	3,45	1	2
IMA4	O00505	3,45	1	4	3,45	1	6
IMDH2	P12268	1,95	1	1	1,95	1	1
INAR1	P17181	2,66	1	1	2,66	1	1
INF2	Q27J81				2,58	1	2
IPO5	O00410	1,54	1	1			
IPP2	P41236	27,80	2	4	28,78	3	10
IQEC2	Q5JU85	1,79	1	3			
IQGA1	P46940	1,93	2	4	1,93	3	5
IRS1	P35568	1,13	1	1	2,82	3	3
IRS2	Q9Y4H2				0,90	1	1
ITA3	P26006	1,24	1	3	1,24	1	4
ITA5	P08648	1,72	1	5	1,72	1	13
ITAV	P06756				1,50	1	2
ITPR1	Q14643	0,33	1	1	0,33	1	1
ITSN1	Q15811	2,17	1	2	2,17	1	2
IWS1	Q96ST2	4,03	2	2	18,07	10	12
JIP4	O60271	3,28	4	13	9,63	6	15
JMY	Q8N9B5	2,46	1	1	2,25	1	2
K0556	O60303	0,74	1	1	1,05	1	1
K0754	O94854	1,43	1	1	1,43	1	1
K0930	Q6ICG6	7,30	1	1			
K1143	Q96AT1	36,36	4	6	26,62	3	7
K1468	Q9P260	2,14	1	2			
K1522	Q9P206	1,93	1	3	3,00	2	4
K1671	Q9BY89				4,79	1	1
K1C10	P13645	4,45	3	3	1,20	1	1
K2C8	P05787	3,73	2	3	3,73	2	4
KAP0	P10644	5,34	1	15	11,87	3	18
KAP2	P13861	11,52	3	8	11,52	3	13
KAP3	P31323	5,02	1	1	5,02	1	2
KCC2D	Q13557	6,69	2	8	5,02	2	19
KCC2G	Q13555				1,62	1	1
KCC4	Q16566	6,77	1	1			
KDM5C	P41229				1,31	1	1
KHDR1	Q07666	7,92	3	5			
KI13A	Q9H1H9				0,74	1	2
KI13B	Q9NQT8	2,30	3	5	2,74	4	5
KI21A	Q7Z4S6	1,54	1	3	3,27	2	5
KIF15	Q9NS87				0,92	1	1
KIF1B	O60333	1,30	2	2	0,51	1	2
KIF1C	O43896				1,27	1	1
KIF23	Q02241	3,42	1	3	3,42	1	6
KIF24	Q5T7B8	1,81	1	1			
KINH	P33176	3,32	2	2	1,14	1	1
KLC1	Q07866	3,77	2	3	3,77	2	4
KLC2	Q9H0B6	4,40	2	2	4,40	2	2
KLC4	Q9NSK0				2,40	1	1
KLDC4	Q8TBB5				7,13	1	1
KPBB	Q93100				0,83	1	1
KPCA	P17252	3,13	1	1	1,19	1	1
KPCD	Q05655	3,11	3	12	3,11	3	17
KPYM	P14618	21,66	8	9	11,86	7	8
KS6A1	Q15418				1,09	1	1
KS6A3	P51812	3,11	2	3	3,11	2	2
KTN1	Q86UP2	1,31	1	2	1,31	1	1
LA	P05455	5,64	4	6	5,64	4	9
LAMC1	P11047	1,37	1	2	1,37	1	3
LAP2	Q96RT1	3,30	2	5	1,92	1	2
LAP2B	P42167	13,71	2	12	19,35	2	12
LAR4B	Q92615	2,17	2	5	2,17	2	4

LARP1	Q6PKG0	11,95	7	19	11,95	9	16
LARP4	Q71RC2	1,65	1	1			
LARP6	Q9BRS8	16,70	4	4	7,13	2	3
LARP7	Q4G0J3	5,33	2	6	8,08	3	6
LASP1	Q14847	11,22	1	2	11,22	1	4
LC7L3	O95232				5,56	1	1
LDHA	P00338	22,89	6	8	11,45	4	5
LDLR	P01130	4,40	1	1			
LEG1	P09382	16,30	2	2	16,30	2	2
LEO1	Q8WVC0	2,31	2	3	4,46	3	5
LFG3	Q969X1	4,50	1	1	4,50	1	2
LIMA1	Q9UHB6	13,69	5	8	12,69	4	5
LIMC1	Q9UPQ0				12,32	1	1
LIMD1	Q9UGP4	3,40	1	2	3,40	1	1
LIMK1	P53667	3,26	1	1			
LIMK2	P53671				3,24	1	1
LIPA1	Q13136	1,27	1	2	1,27	1	3
LIPB1	Q86W92	6,18	5	20	7,81	6	22
LMAN2	Q12907				3,09	1	1
LMNA	P02545	13,99	5	12	8,92	5	9
LMNA	P02545	16,27	6	9	7,68	5	9
LMNB2	Q03252				3,23	2	2
LMO7	Q8WWI1	4,60	7	9	5,78	7	20
LPIN2	Q92539				3,35	1	1
LPXN	O60711	5,70	1	4	5,70	1	3
LRC47	Q8N1G4	6,00	3	7			
LRP1	Q07954	1,50	4	5	0,86	2	3
LRRF1	Q32MZ4	10,77	4	15	12,37	5	9
LRRF2	Q9Y608	8,75	2	5	8,75	2	11
LRWD1	Q9UFC0	6,49	2	4	6,49	2	3
LS14A	Q8ND56				2,13	1	2
LUC7L	Q9NQ29	2,82	1	1			
LUZP1	Q86V48	1,75	1	4	2,83	2	4
LYRIC	Q86UE4	5,50	2	2	3,09	1	1
LYSM2	Q8IV50	7,44	1	2			
M3K2	Q9Y2U5	3,39	1	4			
M3K7	O43318	6,11	2	2	3,67	1	1
M4K4	O95819	1,46	1	1			
MA7D1	Q3KQU3	5,73	3	13	10,09	5	13
MA7D3	Q8IWC1	1,80	1	1	1,92	1	1
MACF1	Q9UPN3	2,23	7	14	1,09	4	10
MAF1	Q9H063	6,25	1	1	6,25	1	1
MAGD2	Q9UNF1	3,74	1	2	3,74	1	1
MAGI3	Q5TCQ9				0,81	1	1
MAN1	Q9Y2U8				1,87	1	1
MAP1A	P78559	20,87	40	127	15,66	34	110
MAP1B	P46821	29,29	48	174	22,81	38	162
MAP1S	Q66K74	2,03	2	8	1,94	1	3
MAP4	P27816	14,41	10	26	9,46	8	19
MAP9	Q49MG5	4,24	1	1	4,24	1	1
MARCS	P29966	10,84	3	4	23,49	5	13
MARH7	Q9H992	2,85	1	1			
MARK2	Q7KZI7				2,62	2	2
MARK3	P27448	2,88	1	4	2,88	1	3
MAST4	O15021				0,54	1	1
MATR3	P43243	2,60	1	1	1,65	1	1
MAX	P61244	12,62	1	1			
MB12A	Q96EY5	5,84	1	1	5,84	1	1
MCAF1	Q6VMQ6	2,17	1	1	3,26	2	2
MCFD2	Q8NI22	18,09	1	3	18,09	1	3
MCM2	P49736	4,42	5	10	4,42	5	11
MCM3	P25205	2,97	1	3	5,69	2	10
MED1	Q15648	0,89	1	2	1,90	2	2
MEI1	Q5TIA1	4,75	1	1			
MELK	Q14680	3,06	1	1	3,06	1	1
MELPH	Q9BV36	15,79	4	14	12,89	5	19
MEP50	Q9BQA1	4,32	1	2			
MEPCE	Q7L2J0	2,03	1	2	4,21	2	3
MET	P08581				1,22	1	3
MFAP1	P55081	4,78	1	2	9,57	2	2
MFF	Q9GZY8	3,36	1	1	7,56	2	2
MFF	Q9GZY8				7,90	2	2

MFS11	O43934	7,81	1	1	7,81	1	4
MFSD5	Q6N075	4,22	1	1			
MFSD8	Q8NHS3	4,05	1	1			
MIA3	Q5JRA6	0,81	1	1	2,54	2	7
MIC19	Q9NX63				7,93	1	2
MICA2	O94851	2,55	1	1			
MICA3	Q7RTP6	1,37	1	2	1,37	2	4
MIEAP	Q8TC71	1,98	1	1	1,98	1	1
MILK1	Q8N3F8	1,27	1	2	1,51	1	2
MINT	Q96T58				0,22	1	1
MK01	P28482	6,01	1	10	6,01	1	11
MK03	P27361	5,67	1	5	5,67	1	4
MK07	Q13164	2,36	1	1			
MKL1	Q969V6	1,83	1	1			
ML12A	P19105				6,43	1	1
MLEC	Q14165	8,90	1	3			
MLH1	P40692	3,11	1	1			
MLTK	Q9NYL2				1,38	1	1
MMTA2	Q9BU76	6,08	1	1	6,08	1	3
MOES	P26038	1,56	1	1	1,56	1	1
MON1A	Q86VX9				3,42	1	2
MON1B	Q7L1V2	8,23	1	2			
MORC2	Q9Y6X9				3,92	3	3
MOT1	P53985	4,20	2	8	4,20	2	6
MP2K2	P36507	3,25	1	1			
MPP8	Q99549	2,33	1	1			
MPRD	P20645	7,58	1	1			
MPRI	P11717	1,53	2	2	0,92	1	2
MPRIP	Q6WCQ1	6,73	4	5	5,56	3	6
MRCKA	Q5VT25	0,55	1	1	0,55	1	1
MRCKB	Q9Y5S2	1,23	1	1	1,23	1	1
MRP1	P33527	0,74	1	2	1,04	1	1
MRP4	O15439	2,30	1	2	2,17	1	1
MRP5	O15440				1,29	1	2
MTAP2	P11137	1,15	2	4	2,96	4	8
MTCL1	Q9Y4B5	1,10	1	2	1,94	2	3
MTCL1	Q9Y4B5	1,33	1	1	4,92	2	2
MTMR3	Q13615				0,86	1	1
MTMR4	Q9NYA4				1,26	1	3
MTMR6	Q9Y217	2,25	1	1			
MTMR9	Q96QG7	3,45	1	1	3,23	1	1
MTNB	Q96GX9				4,90	1	1
MTPN	P58546	16,10	1	1			
MTSSL	Q765P7	3,08	1	1			
MVD1	P53602	4,00	1	1	4,00	1	1
MXRA7	P84157	20,00	3	6	20,00	2	6
MY18A	Q92614	4,76	6	16	5,80	8	20
MYCB2	O75592	0,91	3	5	0,86	3	12
MYCPP	Q7Z401				0,81	1	2
MYCT	Q96QE2				2,31	1	1
MYH1	P12882				0,77	1	1
MYH9	P35579	7,65	14	34	7,30	12	36
MYL6	P60660	9,93	2	3	9,93	2	3
MYO5A	Q9Y4I1	0,93	1	2			
MYO9B	Q13459	4,06	5	8	2,97	4	8
MYPN	Q86TC9	1,82	1	1	1,82	1	2
MYPT1	O14974	4,77	2	6	4,77	2	6
NAA10	P41227	12,27	1	3	19,09	2	3
NAA15	Q9BXJ9	2,66	1	3			
NAA30	Q147X3	7,73	1	1	11,33	2	4
NAB2	Q15742				9,11	3	4
NACA	Q13765	16,28	1	1	16,28	1	1
NADAP	Q9BWU0				2,26	2	4
NAF1	Q96HR8				5,66	1	1
NAMPT	P43490	3,05	1	2	3,05	1	1
NASP	P49321	13,14	2	5			
NAV1	Q8NEY1				1,01	1	1
NBAS	A2RRP1				0,89	1	2
NCBP1	Q09161	2,41	2	8	2,28	1	2
NCBP2	P52298				12,62	1	1
NCK1	P16333	7,03	1	1	7,03	2	2
NCK5L	Q9HCH0				1,09	1	1

NCOA7	Q8NI08				6,02		1	3
NCOR1	O75376				1,97		2	4
NDE1	Q9NXR1	3,58	1	2				
NDKA	P15531	9,21	1	1	9,21		1	1
NDRG1	Q92597	13,74	4	32	15,34		6	52
NDRG3	Q9UGV2	19,58	3	5	14,34		3	23
NED4L	Q96PU5	2,04	1	2	2,64		1	1
NEK1	Q96PY6				0,76		1	1
NEK4	P51957	1,46	1	2	1,60		1	1
NELFE	P18615	3,16	1	2	11,32		3	6
NEMF	O60524	2,71	2	2				
NEMO	Q9Y6K9	4,38	1	1				
NEST	P48681				1,91		1	2
NEUM	P17677	8,82	2	2	7,14		1	1
NF1	P21359	0,71	1	2				
NFIC	P08651	8,37	2	4	8,37		3	7
NFKB2	Q00653	2,78	1	2				
NFX1	Q12986	1,44	1	1				
NGRN	Q9NPE2	35,94	1	3	35,94		1	1
NHRF1	O14745	8,91	1	3	8,91		1	6
NHSL2	Q5HYW2	2,26	1	1				
NIBL1	Q96TA1	2,73	1	5	2,73		1	4
NIPA	Q86WB0	4,78	1	2	4,78		1	3
NMD3	Q96D46	3,38	1	4	3,38		1	5
NOB1	Q9ULX3	4,37	1	3	8,98		2	3
NOC2L	Q9Y3T9	2,27	1	1				
NOG1	Q9BZE4				2,51		1	3
NOL3	O60936	16,35	1	1				
NOP58	Q9Y2X3	4,35	1	2	4,54		2	3
NP1L4	Q99733	8,27	2	5	6,67		1	3
NPAS3	Q8IXF0	1,55	1	1				
NPM	P06748	41,70	5	23	31,27		3	5
NRBP	Q9UHY1				2,24		1	1
NRBP2	Q9NSY0	3,99	1	2	4,39		2	4
NRDC	O43847	4,26	3	8	2,17		2	5
NSF1C	Q9UNZ2	5,01	1	1	5,01		1	1
NSRP1	Q9H0G5	4,48	1	2	4,48		1	3
NSUN2	Q08J23	5,65	1	9	5,65		1	6
NU214	P35658	0,82	1	1	0,82		1	3
NUB1	Q9Y5A7	2,66	1	1				
NUCB1	Q02818				2,82		1	2
NUCKS	Q9H1E3	34,16	9	28	35,39		12	53
NUCL	P19338	3,94	2	5	1,13		1	1
NUDC	Q9Y266	3,02	1	1				
NUDT6	P53370	13,61	1	1				
NUFP2	Q7Z417	3,17	1	1	3,88		2	3
NUP98	P52948	1,85	1	1	3,59		2	3
NXP20	Q8IWE2	7,99	1	2	7,99		1	2
OCAD1	Q9NX40	5,88	1	1	5,88		1	1
ODBA	P12694	3,15	1	2	3,15		1	5
ODPA	P08559	3,90	1	3	6,41		2	7
ODPB	P11177	4,69	1	1				
OGA	O60502	3,40	1	4				
OGFR	Q9NZT2	6,09	2	4	7,91		3	5
OPA1	O60313	1,04	1	1				
OPTN	Q96CV9	5,58	3	5	6,15		3	11
OSBL1	Q9BXW6	2,11	1	1	2,11		1	1
OSBL3	Q9H4L5	4,87	2	8	4,87		2	6
OSBL8	Q9BZF1				2,95		1	2
OSBP1	P22059	3,35	1	2	3,35		2	3
OSBP2	Q969R2	3,87	1	1	3,87		1	1
OSTF1	Q92882	7,01	1	6	7,01		1	20
OSTM1	Q86WC4				3,89		1	1
OSTP	P10451	20,21	3	3	18,49		3	6
OTU7B	Q6GQQ9				2,95		1	1
OTUB1	Q96FW1	9,59	1	3				
OTUD4	Q01804				1,43		1	1
OXR1	Q8N573	2,98	1	4	2,98		1	3
P2R3A	Q06190	1,04	1	1	1,83		2	4
P2RX4	Q99571				5,41		1	1
P3C2A	O00443				1,45		1	1
P4K2A	Q9BTU6	9,60	4	12	9,60		4	12

P66A	Q86YP4	3,29	1	2	3,29	1	3
PA24A	P47712	2,40	1	2	2,40	1	1
PABP1	P11940				2,19	1	1
PACN3	Q9UKS6	6,84	2	5	6,84	2	4
PACS1	Q6VY07	2,08	1	5	2,08	1	5
PAGR1	Q9BTK6	7,09	1	1			
PAIRB	Q8NC51	15,76	3	7	10,08	2	4
PAK1	Q13153	6,61	1	1	1,28	1	1
PAK2	Q13177	7,63	2	4	7,63	2	2
PALLD	Q8WX93	2,38	2	2	4,02	2	2
PALMD	Q9NP74	17,82	3	7	9,04	2	6
PANK2	Q9BZ23				4,03	1	1
PARD3	Q8TEW0	3,78	2	3	1,26	2	2
PARG	Q86W56	3,00	1	1	3,00	1	4
PARP4	Q9UKK3	0,99	1	1	2,03	2	4
PARVA	Q9NVD7	13,19	2	5	5,49	1	1
PATL1	Q86TB9	1,91	1	2	2,07	2	8
PAXI	P49023	10,32	4	13	6,26	3	14
PBDC1	Q9BVG4	7,30	2	2	7,30	2	4
PBIP1	Q96AQ6	2,11	1	2	5,29	2	4
PCF11	O94913				0,64	1	1
PCLI1	Q7Z2X4				11,31	1	1
PCM1	Q15154	4,06	4	6	4,06	4	11
PCNP	Q8WW12	13,48	1	1			
PCY1A	P49585	11,17	3	3	11,17	3	6
PDC6I	Q8WUM4	1,15	1	1			
PDCD4	Q53EL6				3,71	1	3
PDCD5	O14737	8,80	1	1	8,80	2	2
PDE12	Q6L8Q7	6,36	1	9			
PDIA1	P07237	3,94	3	4	3,94	2	2
PDIA4	P13667	3,41	1	2			
PDIA6	Q15084	2,29	1	1	2,29	1	1
PDRG1	Q9NUG6	8,27	1	1	8,27	1	1
PDS5B	Q9NTI5	1,29	1	2	3,52	3	3
PDXD1	Q6P996	2,73	1	2			
PDZD8	Q8NEN9	1,47	1	1	1,47	1	2
PEA15	Q15121	11,54	2	4	11,54	2	5
PEAK1	Q9H792	1,26	2	3			
PEBB	Q13951				9,63	1	1
PEBP1	P30086	8,02	1	2			
PERQ2	Q6Y7W6	1,31	2	4	1,31	2	2
PEX19	P40855	6,51	1	5	6,51	1	6
PFKAL	P17858				1,03	1	1
PFKAP	Q01813	1,29	1	2	1,29	1	1
PGAM1	P18669	4,33	1	2	4,33	1	3
PGAM5	Q96HS1	5,49	1	3			
PGK1	P00558	2,31	1	1	2,31	1	2
PGRC1	O00264	32,87	5	23	32,87	5	25
PGRC2	O15173	10,76	1	4	10,76	1	2
PHAG1	Q9NWQ8				3,24	1	1
PHAR4	Q8IZ21	4,08	2	4	4,08	2	5
PHAX	Q9H814	3,55	1	1			
PHF6	Q8IWS0	12,82	2	2	4,81	1	2
PHF6	Q8IWS0	17,52	4	9	17,22	3	6
PHF8	Q9UPP1	2,16	1	1	2,16	1	2
PHLB1	Q86UU1	3,62	3	5			
PHLP	Q13371				6,88	1	1
PHRF1	Q9P1Y6	1,67	1	2	3,21	2	2
PI4KB	Q9UBF8	3,75	2	4	2,25	2	4
PIGB	Q92521	1,81	1	2	1,81	1	2
PJA1	Q8NG27				2,89	1	1
PJA2	O43164	1,89	1	2	1,89	1	1
PKHA3	Q9HB20	8,00	1	6	8,00	1	10
PKHA6	Q9Y2H5				1,53	1	2
PKHM2	Q8IWE5	3,14	1	1			
PKHO1	Q53GL0	6,40	2	2	6,40	2	2
PKHO2	Q8TD55	2,95	1	1	2,95	1	1
PKN1	Q16512	3,61	2	3	1,38	1	1
PKN2	Q16513				2,13	1	2
PLEC	Q15149	0,68	2	2	1,08	3	6
PLOD2	O00469	2,87	1	1			
PLPP6	Q8IY26				6,44	1	1

PML	P29590	4,29	1	1			
PNKP	Q96T60				4,36	1	1
PNPO	Q9NVS9	11,45	2	3	10,24	1	1
PP12C	Q9BZL4				2,12	1	3
PP1R7	Q15435	23,93	3	4	5,00	1	3
PP4R2	Q9NY27	13,06	3	5	13,06	4	8
PP6R3	Q5H9R7	4,61	2	5	2,08	1	4
PPIA	P62937	8,57	1	1	8,57	1	1
PPIB	P23284	6,48	1	1	6,48	1	1
PPID	Q08752	6,22	1	6			
PPIG	Q13427				1,46	1	1
PPM1B	O75688	5,73	1	1			
PR38B	Q5VTL8	2,38	1	2	2,38	2	3
PR40A	O75400				1,51	1	1
PRC2A	P48634	0,97	1	2	2,46	3	7
PRC2B	Q5JSZ5	2,87	3	4	2,87	3	4
PRC2C	Q9Y520	0,94	1	1	1,92	3	6
PRCC	Q92733	8,96	2	4	4,28	2	3
PRDX1	Q06830	15,08	2	3	8,04	2	2
PRDX6	P30041	7,59	1	1			
PREX1	Q8TCU6	1,05	1	1	1,05	1	2
PRIC2	Q7Z3G6	1,54	1	1	2,73	2	2
PRIPO	Q96LW4				2,50	1	2
PRKRA	O75569	5,75	1	1			
PROF1	P07737	30,00	3	6	8,57	1	1
PRPF3	O43395	3,37	1	1	3,37	1	1
PRPS1	P60891				2,79	1	1
PRR11	Q96HE9	4,72	1	1			
PRR12	Q9ULL5	3,76	2	2	2,19	1	1
PRRT3	Q5FWE3	1,34	1	1	1,34	1	1
PRS4	P62191	4,36	1	1	4,36	1	1
PRS7	P35998	2,77	1	3			
PRUN2	Q8WUY3	1,37	3	8	1,80	4	5
PSA	P55786	1,07	1	1	1,07	1	1
PSA3	P25788	5,65	1	3	5,65	1	5
PSA4	P25789	5,79	1	5	5,79	1	1
PSD10	O75832	6,62	1	1	6,62	1	1
PSD3	Q9NYI0	3,31	1	1	3,31	1	3
PSF2	Q9Y248				7,57	1	1
PSIP1	O75475	3,95	1	7	8,21	1	1
PSMD1	Q99460	2,06	1	1			
PSMD2	Q13200	4,52	3	4	4,52	3	3
PSN1	P49768	4,38	1	1	4,38	1	3
PSN2	P49810				2,90	1	1
PTBP1	P26599				2,26	1	3
PTN12	Q05209	9,85	3	4	8,77	3	4
PTN14	Q15678	2,11	2	3	2,11	2	3
PTOV1	Q86YD1				2,64	1	1
PTSS1	P48651	12,23	3	7	11,62	2	13
PTSS2	Q9BVG9	3,29	2	2	3,29	2	3
PUF60	Q9UHX1				3,21	1	1
PUM1	Q14671	1,15	2	2	1,15	1	2
PUM2	Q8TB72	1,22	1	1	2,23	2	3
PURB	Q96QR8				6,09	1	2
PWP1	Q13610				18,37	1	1
PYR1	P27708	0,76	2	5	0,76	2	4
PYRG1	P17812	5,83	2	25	7,50	3	8
PYRG2	Q9NRF8				3,24	1	3
QSOX2	Q6ZRP7	3,15	1	5	3,15	1	10
R113A	O15541				7,00	1	1
R3HD2	Q9Y2K5	2,24	1	2	2,24	1	3
RAB10	P61026	6,00	1	1			
RAB8A	P61006	7,25	1	1			
RABE1	Q15276				2,05	2	4
RABL6	Q3YEC7	9,74	4	6	3,98	2	6
RABX5	Q9UJ41				1,63	1	1
RAD18	Q9NS91				2,22	1	1
RAD9A	Q99638				4,35	1	1
RADI	P35241	4,67	1	1	4,67	1	1
RAE1	P24386				1,99	1	3
RAF1	P04049	2,01	1	2	2,01	1	2
RAI14	Q9P0K7	6,31	3	3	1,26	1	1

RANB3	Q9H6Z4	5,21	1	3	20,64	4	12
RANG	P43487	17,00	1	6	17,00	1	3
RAPH1	Q70E73	2,08	1	2	3,36	2	3
RB11A	P62491				5,16	1	1
RB27A	P51159				7,51	1	1
RB6I2	Q8IUD2	1,81	1	2	1,81	1	1
RBCC1	Q8TDY2	0,75	1	2	1,32	2	7
RBG1L	Q5R372				7,60	1	5
RBGP1	Q9Y3P9	1,40	1	1	3,27	2	3
RBL2	Q08999	1,23	1	1	1,23	1	1
RBM12	Q9NTZ6	1,50	1	1			
RBM23	Q86U06				5,33	1	1
RBM26	Q5T8P6				1,12	1	2
RBM33	Q96EV2	0,94	1	1	1,71	1	1
RBM39	Q14498	4,96	2	5	4,96	2	6
RBMX	P38159				3,70	2	2
RBP1	Q15311	1,83	1	2	5,04	2	2
RBP2	P49792	1,67	2	3	0,65	1	2
RCN1	Q15293	20,54	4	16	10,57	3	6
RCOR1	Q9UKL0	5,39	1	1			
RD23A	P54725	9,74	1	7	9,74	1	5
RD23B	P54727	8,61	1	16	8,61	1	8
RECQ5	O94762	2,39	1	1			
REEP4	Q9H6H4	11,67	2	4	11,67	2	7
RENT1	Q92900	1,52	1	1	1,52	1	2
REPS1	Q96D71				2,47	2	4
REQU	Q92785	7,25	1	1	7,25	1	2
RER1	O15258	9,18	1	1			
RERE	Q9P2R6				1,19	1	1
RFC1	P35251	1,39	1	2	1,39	1	3
RFFL	Q8WZ73	5,63	1	1	5,63	1	4
RFIP5	Q9BXF6	4,44	2	6	6,58	3	12
RFTN1	Q14699	7,09	2	2	9,00	3	3
RGPA1	Q6GYQ0	3,53	2	13			
RGPS2	Q86X27	3,77	1	3	3,77	1	2
RGRF1	Q13972	6,34	1	1			
RHG01	Q07960	4,56	2	3	5,69	3	6
RHG05	Q13017	3,40	3	3	2,27	3	5
RHG07	Q96QB1				1,67	1	3
RHG12	Q8IWW6	5,29	2	4	5,29	2	4
RHG21	Q5T5U3	4,29	6	11	1,89	3	7
RHG22	Q7Z5H3	6,48	1	3			
RHG23	Q9P227	2,62	2	2	2,62	2	3
RHG29	Q52LW3				4,83	1	2
RHG32	A7KAX9	1,09	1	3	1,09	1	2
RHG35	Q9NRY4	1,00	1	6	1,87	2	7
RIC8A	Q9NPQ8	5,14	1	3	5,14	1	3
RICTR	Q6R327	2,34	5	6	1,93	3	3
RIF1	Q5UIP0				0,53	1	1
RIMS3	Q9UJD0	8,44	1	1	5,19	1	1
RIN1	Q13671	1,53	1	1	2,91	1	1
RIN2	Q8WYP3				1,34	1	1
RINI	P13489				2,17	1	1
RIOK2	Q9BVS4	8,86	2	3	3,16	1	5
RIOK3	O14730				4,07	1	2
RL15	P61313				6,21	1	1
RL1D1	O76021				5,93	1	2
RL22L	Q6P5R6	12,30	1	4	12,30	1	8
RL34	P49207	8,55	1	1			
RL6	Q02878	6,94	2	8	6,94	1	2
RL7	P18124	7,66	1	2			
RLA0	P05388	7,84	1	1	7,84	1	4
RLA1	P05386	19,10	1	12	19,10	1	19
RMD3	Q96TC7	3,83	1	4	3,83	1	2
RMP	O94763				5,88	1	3
RN139	Q8WU17				2,86	1	1
RN213	Q63HN8	0,54	1	2	0,54	1	2
RNF13	O43567				3,05	1	1
RNF4	P78317	11,05	1	2	11,05	1	4
RNH1	O60930	7,69	1	4	7,69	1	9
ROA1	P09651	7,12	2	5	4,49	2	5
ROCK1	Q13464	1,11	1	2			

RP1L1	Q8IWN7				1,09	1	1
RPB3	P19387	6,91	1	1			
RPC3	Q9BUI4				2,43	1	1
RPGF1	Q13905				1,25	1	1
RPGF6	Q8TEU7	3,26	1	2	3,26	1	2
RPP30	P78346	5,97	1	1	5,97	1	1
RPRD2	Q5VT52	1,71	1	2	1,71	1	2
RPTOR	Q8N122	1,53	1	1	1,53	1	1
RRAGC	Q9HB90	3,51	1	2	3,51	1	2
RRAS2	P62070	12,60	1	2	12,60	2	3
RRBP1	Q9P2E9				2,97	1	3
RRN3	Q9NYV6				8,20	1	1
RRP12	Q5JTH9				1,84	1	2
RS12	P25398	6,06	1	1			
RS27	P42677	15,48	2	3	15,48	1	1
RS27L	Q71UM5	15,48	1	2	15,48	1	1
RS28	P62857	15,94	1	1	15,94	1	2
RS3	P23396	5,35	1	1	5,35	1	1
RS4X	P62701				2,66	1	1
RS6	P62753	4,42	2	3	6,83	2	2
RSRC2	Q7L4I2	2,53	1	1	5,99	4	6
RTKN	Q9BST9	2,14	1	2	2,14	1	2
RTN1	Q16799	3,87	2	6	1,68	1	2
RTN4	Q9NQC3	5,62	3	14	2,01	1	1
RU17	P08621	3,81	1	2			
RUBIC	Q92622	1,09	1	1			
RUVB1	Q9Y265	4,17	1	4			
S10A6	P06703	16,67	2	2	8,89	1	2
S10AD	Q99584				10,20	1	1
S10AG	Q96FQ6				10,68	1	2
S11IP	Q8N1F8	2,46	2	4	1,36	1	2
S20A1	Q8WUM9	3,83	2	8	2,06	1	2
S20A2	Q08357	3,68	1	1			
S30BP	Q9UHR5	5,14	1	1	5,14	1	1
S35A5	Q9BS91				3,30	1	1
S35F3	Q8IY50				3,06	1	1
S35G2	Q8TBE7	3,16	1	1			
S38A1	Q9H2H9	2,05	1	2	1,85	1	1
S38A2	Q96QD8	4,15	1	2			
S39A7	Q92504				2,99	1	2
S4A7	Q9Y6M7	6,26	2	3	5,83	2	5
S7A6O	Q96CW6	4,21	1	1	4,21	1	2
SAGE1	Q9NXZ1				0,88	1	1
SAP	P07602	1,91	1	1			
SASH1	O94885	1,28	1	1	4,57	4	9
SC16A	O15027	2,06	2	4	2,06	2	7
SC22B	O75396	6,51	1	2	6,51	1	1
SC31A	O94979	4,25	3	8	4,25	3	8
SCAF8	Q9UPN6	2,44	2	2	1,57	1	2
SCAM2	O15127				3,95	1	2
SCAM3	O14828	8,07	1	3	2,31	1	1
SCAP	Q12770				1,92	1	1
SCRIB	Q14160	0,71	1	1	2,07	2	3
SDCG3	Q96C92	6,63	1	1	6,63	1	1
SDS3	Q9H7L9	6,71	1	1	6,71	1	3
SEC62	Q99442				6,27	1	1
SEM4B	Q9NPR2	1,20	1	1			
sept-02	Q15019	5,54	1	7	5,54	1	6
sept-06	Q14141	3,28	1	2			
sept-07	Q16181	3,67	1	1			
sept-09	Q9UHD8	4,76	2	5	4,76	2	6
SERC1	Q9NRX5	2,87	1	1	2,87	1	1
SET	Q01105	11,91	1	1			
SF01	Q15637	4,93	2	13	4,93	2	16
SF3A1	Q15459				3,02	1	1
SF3A3	Q12874	2,20	1	1	2,20	1	1
SF3B2	Q13435	2,91	1	1	5,14	3	4
SFPQ	P23246	2,99	1	3			
SFSWA	Q12872	2,84	1	2	7,05	2	3
SFXN2	Q96NB2	3,42	1	1			
SGPP1	Q9BX95	2,95	2	4	2,95	2	3
SGTA	O43765	11,82	2	2	11,82	2	4

SH24A	Q9H788	2,93	1	1	2,93	1	1
SH2B3	Q9UQQ2	4,17	1	4			
SH3B4	Q9P0V3	3,99	1	1			
SH3G1	Q99961				2,63	1	1
SH3K1	Q96B97	2,23	1	1	4,30	2	2
SH3L1	O75368				8,77	1	1
SHB	Q15464	2,55	1	1			
SHCBP	Q8NEM2				2,23	1	1
SI1L3	O60292				1,07	1	3
SIM13	P0DJ93				32,97	1	2
SIPA1	Q96FS4	1,54	1	1	1,54	1	2
SIR1	Q96EB6	3,57	1	1	3,57	1	1
SIR2	Q8IXJ6	12,60	2	10	6,68	1	11
SIR6	Q8N6T7				5,18	1	1
SKIV2	Q15477	0,80	1	1	0,80	1	2
SKP1	P63208	21,47	2	4	7,36	1	1
SL9A1	P19634	3,07	1	3	3,07	1	3
SLAI2	Q9P270	2,75	1	1	2,75	2	2
SLIRP	Q9GZT3	11,21	1	1	11,21	1	1
SLK	Q9H2G2	1,91	1	1	1,91	1	2
SMAP	O00193	26,23	4	13	13,66	4	22
SMC3	Q9UQE7	0,82	1	1	0,82	1	1
SMC4	Q9NTJ3	3,58	2	21	3,58	2	27
SMN	Q16637	7,60	2	12	7,60	2	8
SMRC1	Q92922				1,36	1	3
SMRC2	Q8TAQ2	2,21	2	2	1,15	1	5
SNAA	P54920	8,81	1	1			
SND1	Q7KZF4	1,32	1	1	1,43	1	1
SNP23	O00161	8,06	1	1			
SNRK	Q9NRH2				3,79	1	2
SNTA1	Q13424	4,65	1	2	6,98	2	4
SNTB1	Q13884				4,97	1	1
SNX14	Q9Y5W7	1,01	1	1	1,01	1	1
SNX29	Q8TEQ0	5,78	2	2	4,18	2	2
SNX3	O60493	10,77	1	1			
SNX9	Q9Y5X1				3,53	1	4
SODC	P00441	15,58	2	6	9,09	1	2
SOSSC	Q9NRY2	12,50	1	1			
SP100	P23497	5,17	1	1			
SPAT2	Q9UM82	2,31	1	1	2,31	1	1
SPB1	Q8IY81				2,95	1	4
SPB6	P35237				3,99	1	1
SPD2A	Q5TCZ1				5,00	3	5
SPD2B	A1X283	1,54	1	1	1,54	1	1
SPF45	Q96I25	3,99	1	2	3,99	1	2
SPG21	Q9NZD8	3,56	1	1	3,56	1	1
SPIR1	Q08AE8	2,57	1	2	2,57	1	1
SPRE1	Q7Z699	2,70	1	1	2,70	1	1
SPRE2	Q7Z698	3,13	1	1	3,13	1	1
SPS2	Q99611	3,35	1	1			
SPTB2	Q01082	1,40	3	5	2,50	5	13
SPTCS	Q96JI7				0,53	1	1
SPTN1	Q13813	2,08	6	15	1,51	3	4
SQSTM	Q13501	19,32	5	16	10,91	3	3
SRBS1	Q9BX66	3,03	1	2			
SRC8	Q14247	6,24	5	17	6,24	5	14
SRCAP	Q6ZRS2	0,65	1	1			
SRGP1	Q7Z6B7				1,51	1	1
SRPK2	P78362	1,74	1	1			
SRPRA	P08240	5,25	1	1	5,25	1	7
SRRM1	Q8IYB3	20,91	19	44	19,58	19	56
SRRM2	Q9UQ35	4,88	6	14	4,58	7	9
SRS11	Q05519	6,42	2	2	9,52	3	5
SRSF2	Q01130	8,13	1	2			
SRSF4	Q08170				3,44	1	3
SRSF6	Q13247	2,91	1	1	6,10	3	4
SSFA2	P28290				5,02	4	8
SSH1	Q8WYL5	1,49	1	3	4,21	2	8
SSH2	Q76I76				0,91	1	2
SSH3	Q8TE77	7,11	1	1	7,11	1	3
SSRG	Q9UNL2	7,57	1	1			
SSRP1	Q08945	3,39	1	1	3,39	1	1

ST32C	Q86UX6	3,29	1	1	3,29	1	2
STA13	Q9Y3M8				2,33	1	1
STAM1	Q92783	3,97	1	1			
STAT3	P40763				3,05	1	1
STIM1	Q13586	8,32	3	5	5,11	2	6
STIP1	P31948	3,28	1	4	5,01	2	2
STK10	O94804	4,96	3	9	4,13	3	4
STK11	Q15831				2,72	1	1
STK33	Q9BYT3	3,13	1	3	3,13	1	3
STK39	Q9UEW8	3,99	1	1	3,99	2	2
STK4	Q13043				4,76	1	1
STMN1	P16949	40,27	5	6	16,11	2	6
STRN	O43815	3,15	2	2	3,15	2	2
STRP1	Q5VSL9	3,86	1	1	3,86	1	1
STRP2	Q9ULQ0				1,32	1	1
STT3B	Q8TCJ2	2,78	2	7	2,78	2	11
STX18	Q9P2W9	6,27	1	3			
STX1A	Q16623	7,57	2	3	7,57	2	3
STX4	Q12846	6,44	1	2	6,44	1	2
STXB1	P61764				1,85	2	3
STXB5	Q5T5C0				1,09	1	1
SUCB2	Q96I99				2,31	1	1
SUFU	Q9UMX1	5,31	1	1	2,54	1	1
SUGP1	Q8IWZ8	4,19	1	1			
SURF2	Q15527	8,59	1	1	8,59	1	2
SVIL	O95425	2,46	3	7	3,36	4	7
SWAHC	Q53LP3				2,10	1	1
SYEP	P07814	2,25	2	4	2,12	2	8
SYFB	Q9NSD9	2,45	1	2	2,45	1	1
SYNC	O43776				1,09	1	1
SYNRG	Q9UMZ2	3,25	2	7	1,71	1	1
T11L1	Q9NUJ3	1,96	1	1			
T2FA	P35269	7,74	2	7	10,25	3	4
TACC1	O75410	4,90	1	1	4,90	1	1
TACC2	O95359	3,85	1	3	3,85	1	1
TAGL2	P37802	5,53	1	2	5,53	1	1
TANC1	Q9C0D5	1,31	1	2	1,31	1	1
TANC2	Q9HCD6	1,75	1	1	1,75	1	2
TAOK3	Q9H2K8	2,45	1	1	2,45	1	1
TAXB1	Q86VP1	2,49	1	1			
TB10B	Q4KMP7	5,07	2	4			
TB182	Q9C0C2	10,01	15	31	10,01	14	40
TBA1A	Q71U36	15,63	4	6	7,21	2	3
TBA1C	Q9BQE3	9,80	3	5	6,24	2	4
TBB5	P07437	2,70	1	1	2,70	1	1
TBC15	Q8TC07	4,30	2	4	4,30	2	3
TBC25	Q3MII6	1,60	1	1	1,60	1	1
TBC9B	Q66K14				2,60	1	1
TBCA	O75347	16,67	1	1			
TBCD4	O60343	3,64	4	14	3,64	4	12
TBCD5	Q92609	2,01	2	6	6,29	4	12
TBD2A	Q9BYX2	3,63	1	1	3,63	1	2
TBD2B	Q9UPU7	1,77	1	2	1,97	2	2
TBX2	Q13207	2,53	1	1			
TBX3	O15119				4,67	1	1
TCAL1	Q15170	8,81	1	1	8,81	1	1
TCAL3	Q969E4	52,50	6	15	57,00	9	27
TCAL4	Q96EI5	9,77	1	1	9,77	1	1
TCAM2	Q86XR7	6,81	1	1	6,81	1	2
TCEA1	P23193	5,36	1	5	5,36	1	5
TCF25	Q9BQ70				3,70	1	1
TCOF	Q13428	3,05	3	4	4,18	5	7
TCPA	P17987	3,24	1	5	3,24	1	5
TCPD	P50991	2,36	1	2	2,36	1	1
TCPG	P49368	3,55	1	1			
TCTP	P13693	13,04	2	2	13,04	2	2
TEBP	Q15185	11,81	1	9	11,81	1	24
TERA	P55072	1,12	1	1			
TEX2	Q8IWB9				3,99	2	2
TF3C3	Q9Y5Q9	5,81	1	1	5,81	1	1
TFE3	P19532				4,52	2	3
TFP11	Q9UBB9	5,85	3	3	3,11	2	2

TFPI2	P48307	4,91	1	1	4,91	1	1
TFR1	P02786				1,84	1	1
TGFR2	P37173				3,00	1	4
TGM2	P21980	5,82	4	6	4,37	3	3
TGON2	O43493	10,03	2	10	11,87	4	15
TGON2	O43493	17,39	4	17	11,44	5	19
TGS1	Q96RS0	2,81	1	3	2,81	1	1
THUM1	Q9NXG2	6,80	1	2	6,80	2	6
THYN1	Q9P016	9,04	1	1			
TIAM1	Q13009				0,72	1	1
TIF1A	O15164				1,97	1	1
TIF1B	Q13263	6,77	2	7	1,73	2	4
TISB	Q07352				2,37	1	1
TJAP1	Q5JTD0	3,11	1	1	6,02	2	2
TLE3	Q04726				3,16	1	2
TLE4	Q04727				1,56	1	2
TLN1	Q9Y490	1,50	5	16	0,91	2	3
TM1L1	O75674	6,94	1	1			
TM230	Q96A57				11,67	1	2
TM237	Q96Q45				2,75	1	1
TM45A	Q9NWC5				4,36	1	1
TM9S2	Q99805	2,87	1	1			
TMCC1	O94876	1,48	1	1	1,48	1	1
TMF1	P82094	1,28	1	1	1,28	1	2
TMM51	Q9NW97	14,23	1	2			
TMOD3	Q9NYL9	5,40	1	4	5,40	1	2
TMX1	Q9H3N1	7,50	1	1	9,29	3	3
TMX2	Q9Y320	10,47	1	3			
TNIK	Q9UKE5	1,18	1	1	1,18	1	1
TNPO1	Q92973	1,18	1	1	1,18	1	1
TNPO3	Q9Y5L0	2,21	1	2			
TOIP1	Q5JTV8	13,19	3	5	13,72	5	16
TOIP2	Q8NFQ8	10,21	2	2	10,21	2	3
TOM34	Q15785	3,56	1	1			
TOM70	O94826	6,41	2	3	6,41	2	2
TOP2A	P11388	0,78	1	1			
TOP2B	Q02880	1,05	1	2	1,11	2	4
TOX4	O94842	3,53	1	2			
TP53B	Q12888	2,79	3	5	2,28	3	4
TPBG	Q13641	3,10	1	1			
TPC12	Q8WVT3				6,12	1	2
TPD52	P55327	5,98	1	1	5,98	1	1
TPD54	O43399	13,98	2	5	13,98	2	6
TPIS	P60174				7,78	1	1
TPM3	P06753	20,00	4	8	9,82	2	2
TPM4	P67936	26,21	5	9	21,37	4	4
TPMT	P51580	6,12	1	1	6,12	1	1
TPPC3	O43617				7,78	1	1
TR112	Q9UI30	11,67	1	5			
TR150	Q9Y2W1				4,29	2	2
TRAD1	O14545	2,41	1	2	2,41	1	2
TRADD	Q15628				3,97	1	1
TRAM2	Q15035				3,24	1	1
TRI16	O95361	5,85	1	4	3,55	1	2
TRI18	O15344				2,54	1	3
TRI22	Q8IYM9	2,83	1	2	2,83	1	2
TRIM3	O75382	2,11	1	2	2,11	1	3
TRIO	O75962	0,74	2	7	1,76	3	8
TRIPC	Q14669	0,60	1	2	1,15	2	4
TRM1	Q9NXH9	2,22	1	1	2,22	1	1
TRPM7	Q96QT4				0,70	1	1
TRPT1	Q86TN4	8,82	1	5	8,82	1	3
TRUA	Q9Y606				3,76	1	1
TSC1	Q92574	4,67	3	4	2,70	2	3
TSC2	P49815	1,77	2	4	2,30	3	6
TTC1	Q99614	13,01	1	5			
TTC33	Q6PID6	4,96	1	1			
TTF2	Q9UNY4	2,96	1	1			
TXLNA	P40222	2,56	1	5	2,56	1	5
TYB4	P62328	54,55	3	3	54,55	3	5
U2AF2	P26368	2,97	1	1	2,97	1	2
U520	O75643				2,56	1	1

U5S1	Q15029	3,43	1	1			
UB2E3	Q969T4	15,94	2	7	15,94	1	1
UB2V1	Q13404	11,65	1	1	11,65	1	1
UBA1	P22314				1,38	1	1
UBC	P0CG48	38,10	2	8	38,10	2	14
UBE2O	Q9C0C9				2,32	2	2
UBE2Z	Q9H832				10,57	1	2
UBE4B	O95155	1,11	1	1	1,11	1	1
UBP10	Q14694	3,26	1	1	3,26	1	2
UBP15	Q9Y4E8	5,15	2	5	3,36	1	5
UBP16	Q9Y5T5	3,31	1	2			
UBP20	Q9Y2K6	2,41	1	1	4,27	2	9
UBP24	Q9UPU5	0,42	1	1	0,42	1	1
UBP2L	Q14157	4,41	2	3	2,56	1	5
UBP32	Q8NFA0	0,75	1	1	0,75	1	2
UBP47	Q96K76	1,32	1	1	2,80	2	4
UBP54	Q70EL1	2,24	1	1	2,24	1	2
UBP7	Q93009				2,18	1	3
UBP8	P40818	1,61	2	7	1,52	2	5
UBR4	Q5T4S7	0,58	2	6	0,41	1	12
UCHL3	P15374	6,09	1	1			
UN93B	Q9H1C4	6,70	1	1	6,70	1	3
USO1	O60763	6,24	2	2			
USP9X	Q93008	1,25	1	3	1,25	1	4
VAMP3	Q15836	17,00	1	1			
VAMP4	O75379	12,06	1	2	11,35	1	2
VAPB	O95292	5,35	1	3	5,35	1	3
VASP	P50552	4,21	1	2	4,21	1	2
VATC1	P21283	4,71	1	1	4,71	1	1
VAV2	P52735				1,31	1	1
VCIP1	Q96JH7	2,21	1	1			
VGF	O15240	4,72	1	1			
VIGLN	Q00341	1,30	1	1			
VIME	P08670	9,23	4	7	12,02	5	10
VINC	P18206				1,59	2	2
VINEX	O60504	3,95	1	2	11,85	3	4
VP13B	Q7Z7G8				1,05	1	1
VP13D	Q5THJ4	0,48	1	2	0,48	1	2
VPP2	Q9Y487	3,15	1	2	3,15	1	3
VPS35	Q96QK1	1,63	1	2			
VPS4A	Q9UN37	5,49	1	1	5,72	2	8
VPS4B	O75351	5,86	2	3	5,86	2	4
VPS50	Q96JG6	1,93	1	3	1,93	1	2
WAC	Q9BTA9				6,19	3	4
WAPL	Q7Z5K2	2,52	2	3	3,19	2	4
WASH7	Q2M389	3,07	2	4	2,13	1	1
WASL	O00401	1,58	1	1			
WBP11	Q9Y2W2	2,34	1	1	2,34	1	1
WDHD1	O75717	4,77	2	3	3,08	1	5
WDR26	Q9H7D7	5,09	1	1			
WDR4	P57081				4,89	1	1
WDR44	Q5JSH3	9,83	5	17	4,85	3	5
WDR47	O94967				1,68	1	1
WDR55	Q9H6Y2	6,27	1	3	6,27	1	1
WDR59	Q6PJI9	4,30	1	2			
WDTC1	Q8N5D0	2,01	1	1	2,01	1	3
WNK1	Q9H4A3	2,23	3	8	0,91	1	9
WWC3	Q9ULE0				2,01	1	1
XPC	Q01831				3,32	1	1
XPO1	O14980				1,40	1	1
XRCC1	P18887	5,53	2	3	5,53	2	2
XRCC5	P13010	3,01	2	2			
XRN2	Q9H0D6				1,83	2	3
XRP2	O75695	4,57	1	4			
YAF2	Q8IY57				11,59	1	3
YAP1	P46937				4,89	1	2
YBOX1	P67809	21,30	3	6	16,36	3	15
YBOX3	P16989	11,99	2	3	7,02	1	1
YIPF1	Q9Y548	8,50	1	1			
YJ005	Q6ZSR9	5,35	1	3	5,35	1	5
YTDC2	Q9H6S0				1,19	1	2
ZBT7A	O95365	7,53	2	3	7,53	3	5

ZC3H4	Q9UPT8	1,92	1	1	3,45	2	3
ZCCHV	Q7Z2W4	2,08	1	1	4,01	2	2
ZCH18	Q86VM9				7,40	3	4
ZFPL1	O95159	5,81	1	3	5,81	1	3
ZFR	Q96KR1	1,40	1	1	1,40	1	1
ZFY26	Q68DK2				2,02	1	1
ZHX3	Q9H4I2	2,93	1	1	4,81	2	2
ZN316	A6NFI3	1,20	1	1	1,20	1	1
ZN318	Q5VUA4	0,90	1	1			
ZN513	Q8N8E2	5,43	1	2			
ZN609	O15014				0,85	1	2
ZNRF2	Q8NHG8	4,96	1	1	4,96	1	2
ZNT1	Q9Y6M5				2,17	1	1
ZO1	Q07157	4,50	4	10	3,78	3	5
ZO2	Q9UDY2	7,80	6	12	11,91	13	15
ZRAB2	O95218	12,50	2	3	16,88	3	5
ZZEF1	O43149	0,68	1	1			

Sample: Scrambled

Accessions	Sequence	PSMs	Modifications	phosphoRS Site Probabilities
Q9UPN3	RGsDASDFDLLETQSAcSDTSESSAAGGQGNSR	4	S3(Phospho); C17(Carbamidomethyl)	S(3): 98.9; S(6): 1.1; T(13): 0.0; S(15): 0.0; S(18): 0.0; T(20): 0.0; S(21): 0.0; S(23): 0.0; S(24): 0.0; S(32): 0.0
O00193	sASPDDDLGSSNWEAADLGNEER	6	S1(Phospho)	S(1): 50.0; S(3): 50.0; S(10): 0.0; S(11): 0.0
Q9UHB6	SKEGHSLEMENENLVENGADsDEDDNSFLK	2	S21(Phospho)	S(1): 0.0; S(6): 0.0; S(21): 100.0; S(27): 0.0
Q92900	SQIDVALsQDSTYQGER	1	S8(Phospho)	S(1): 0.0; S(8): 99.3; S(11): 0.7; T(12): 0.0; Y(13): 0.0
P55211	DHGFEVASTsPEDESPGSNPEPDATPFQEGLR	7	S10(Phospho)	S(8): 1.0; T(9): 6.3; S(10): 43.2; S(15): 43.2; S(18): 6.3; T(25): 0.0
Q9NY27	NHSDSSTSESEVSSVsPLK	2	S16(Phospho)	S(3): 0.0; S(5): 0.0; S(6): 0.0; T(7): 0.0; S(8): 0.0; S(10): 0.0; S(13): 0.0; S(14): 0.0; S(16): 100.0
O60763	LKDLGHPVEEEDELEsGDQEDEDDESEDPGKDLDHI	1	S16(Phospho)	S(16): 100.0; S(26): 0.0
P34932	MQVDQEEPHVEEQQQTPAENKAESEEMETSQAGSK	2		
P07900;P08238	HNDDEQYAWESSAGGSFTVR	7		
Q9Y490	SKDHFGLEGDEESTMLEDSVsPK	6	S21(Phospho)	S(1): 0.0; S(13): 0.0; T(14): 0.0; S(19): 0.5; S(21): 99.5
Q02952	EVSsLEGsPPPcLGQEEAVcTK	3	S4(Phospho); S8(Phospho); C12(Carbamidomethyl); C20(Carbamidomethyl)	S(3): 9.4; S(4): 90.6; S(8): 100.0; T(21): 0.0
Q14147	LQEEQDGGssDEDRAGPAPPGASDGVDIQDVK	2	S9(Phospho); S10(Phospho)	S(9): 100.0; S(10): 100.0; S(23): 0.0
P27348	DNLTLWTSDsAGEEcDAAEGAEN	12	S10(Phospho); C15(Carbamidomethyl)	T(4): 0.0; T(7): 7.7; S(8): 0.7; S(10): 91.7
Q99614	LLRDDEAHLQEDQGEEEcFHDcSASFEEEPGADKVENK	3	C18(Carbamidomethyl); C22(Carbamidomethyl)	
Q9Y487	KDsEEEVSLLGSQDIEEGNHQVEDGcR	2	S3(Phospho); C26(Carbamidomethyl)	S(3): 100.0; S(8): 0.0; S(12): 0.0
Q96K21	LPDsDDDEDEETAIQR	3	S4(Phospho)	S(4): 100.0; T(12): 0.0
P63167	NADMSEEMQQDSVEcATQALEK	2	C15(Carbamidomethyl)	
Q7Z4S6	KLSsSDAPAQDTGSSAAAVETDASR	3	S4(Phospho)	S(3): 46.8; S(4): 46.8; S(5): 5.7; T(12): 0.7; S(14): 0.0; S(15): 0.0; T(21): 0.0; S(24): 0.0
Q8IVF2	DAHDVsPTsTDTEAQLTVER	6	S6(Phospho); S9(Phospho)	S(6): 93.5; T(8): 35.5; S(9): 35.5; T(10): 35.5; T(12): 0.0; T(17): 0.0
Q9C0C2	SFGTRPLSsGFSPEEAQQQDEEFEK	5	S9(Phospho)	S(1): 8.8; T(4): 0.1; S(8): 8.8; S(9): 81.2; S(12): 1.0
Q14694	NHSVNEEEQEEQGEGsEDEWEQVGPR	1	S16(Phospho)	S(3): 0.0; S(16): 100.0
Q13765	VQGEAVSNIQENTQTPTVQEEsEEEEVDETGVEVK	1	S22(Phospho)	S(7): 0.0; T(13): 0.0; T(15): 0.0; T(17): 0.4; S(22): 99.6; T(30): 0.0
Q5T0N5	RHSsDINHLVTQGREsPEGSYTDDANQEVR	1	S4(Phospho); S16(Phospho)	S(3): 50.0; S(4): 50.0; T(11): 0.7; S(16): 99.1; S(20): 0.1; Y(21): 0.1; T(22): 0.0
Q15185	DWEDDsDEDMSNFDR	7	S6(Phospho)	S(6): 100.0; S(11): 0.0
Q9H3N1	KVEEEQEADEEDVsEEEAESK	1	S14(Phospho)	S(14): 100.0; S(20): 0.0
A2RU67	SPLGEAPEPDsDAEVAEAAKPHLSEVTTEGYPSEPLGGLEQK	2	S11(Phospho)	S(1): 0.3; S(11): 99.7; S(24): 0.0; T(27): 0.0; T(28): 0.0; Y(31): 0.0; S(33): 0.0
Q5JSH3	EYVSNDAAQsDDEEKLQSQPTDTDGGR	3	S10(Phospho)	Y(2): 0.0; S(4): 0.0; S(10): 100.0; S(18): 0.0; T(21): 0.0; T(23): 0.0
Q7Z5H3	SLDLDHSMDEAGAGASNSEPSEPDsPTREHAR	3	S25(Phospho)	S(1): 0.0; S(7): 0.0; S(16): 0.0; S(18): 0.0; S(21): 1.2; S(25): 88.8; T(27): 10.0
Q03001	cSMSSSADFsDEDDFSQK	1	C1(Carbamidomethyl); S10(Phospho)	S(2): 0.0; S(4): 0.0; S(5): 0.0; S(6): 0.0; S(10): 100.0; S(16): 0.0
O43583	LtVENSPKQEAGISEGQGTAGEEEEK	4	T2(Phospho)	T(2): 50.0; S(6): 50.0; S(14): 0.0; T(19): 0.0
O43815	FLESAAADFsDEDEDDDVDGREK	1	S10(Phospho)	S(4): 0.0; S(10): 100.0
Q9H1E3	KVVDYSQFQEsDDADEDYGR	4	S11(Phospho)	Y(5): 0.0; S(6): 0.0; S(11): 100.0; Y(18): 0.0
P51858	GNAEGssDEEGKLVIDEPAK	4	S6(Phospho); S7(Phospho)	S(6): 100.0; S(7): 100.0
P49792	NHETDGGsAHGDDDDDGPHFEPVVPLPDKIEVK	1	S8(Phospho)	T(4): 3.5; S(8): 96.5
P06748	cGSGPVHISGQHLVAVEEDAEsEDEEEEDVK	16	C1(Carbamidomethyl); S22(Phospho)	S(3): 0.0; S(9): 0.0; S(22): 100.0
Q9BPX3	TLHcEGTEINsDDEQESKEVEETATAK	4	C4(Carbamidomethyl); S11(Phospho)	T(1): 0.0; T(7): 0.0; S(11): 100.0; S(17): 0.0; T(23): 0.0; T(25): 0.0
Q16623	TAKDsDDDDDVAVTVDR	1	S5(Phospho)	T(1): 0.0; S(5): 100.0; T(14): 0.0
P78559	SLsPEDAESLSVLSVPSPDTANQEPTPK	7	S3(Phospho)	S(1): 50.0; S(3): 50.0; S(9): 0.0; S(11): 0.0; S(14): 0.0; S(17): 0.0; T(20): 0.0; T(26): 0.0
P46821	VSAEAEVAPVsPEVTQEVVEEHcAsPEDK	5	S11(Phospho); C23(Carbamidomethyl); S25(Phospho)	S(2): 0.0; S(11): 99.5; T(15): 1.1; S(25): 99.5
Q9UGV2	SVTSNQSDGtQEScESPDVLDR	3	T10(Phospho); C14(Carbamidomethyl)	S(1): 0.0; T(3): 0.0; S(4): 0.0; S(7): 0.5; T(10): 99.5; S(13): 0.0; S(16): 0.0
Q96GS4	sLDGLSEAcGGAGSSGSAESGAGGGR	1	S1(Phospho); C9(Carbamidomethyl)	S(1): 100.0; S(6): 0.0; S(14): 0.0; S(15): 0.0; S(17): 0.0; S(20): 0.0
Q16637	RGTGQsDDsDIWDDTALIK	7	S6(Phospho); S9(Phospho)	T(3): 1.4; S(6): 98.6; S(9): 100.0; T(15): 0.0
Q9UEY8	SPEKIEEVLSPEGSPSKsPSK	4	S18(Phospho)	S(1): 0.0; S(10): 0.0; S(14): 0.0; S(16): 0.0; S(18): 8.4; S(20): 91.6
P46821	KIAELEEEQSQGSTTNsDWMK	8	S17(Phospho)	S(10): 0.0; S(13): 0.0; T(14): 0.0; T(15): 0.0; S(17): 100.0
Q13501	IALESEGRPEEQMESDNcsGGDDDWTHLSSK	3	C18(Carbamidomethyl); S19(Phospho)	S(5): 0.0; S(15): 0.4; S(19): 99.6; T(26): 0.0; S(29): 0.0; S(30): 0.0
P09603	SHQEPQRADsPLEQPEGSPLTQDDR	1	S10(Phospho)	S(1): 0.0; S(10): 100.0; S(18): 0.0; T(21): 0.0
Q969E4	mEKPYNKNEGNLENEGKPEDEVEPDDEGKsDEEEKPDVEGK	1	N-Term(Acetyl); S30(Phospho)	Y(5): 0.0; S(30): 100.0
P05386	KEEsEEsDDDMGFGLFD	5	S4(Phospho); S7(Phospho)	S(4): 100.0; S(7): 100.0
Q93008	NGILAIEGTGSDVDDDMsGDEKQDNESNVDPR	3	S18(Phospho)	T(9): 0.2; S(11): 17.3; S(18): 82.3; S(27): 0.2
Q02241	RSsTVAPAQPDGAESEWTDVETR	3	S3(Phospho)	S(2): 4.2; S(3): 47.9; T(4): 47.9; S(15): 0.0; T(18): 0.0; T(22): 0.0
Q8NE71	KAEQGsEEEGEGEEEEEEGGESKADDPYAHLSK	1	S6(Phospho)	S(6): 100.0; S(22): 0.0; Y(28): 0.0; S(32): 0.0
Q6PKG0	ESPRPLQLPGAEGPAIsDGEEGGGEPGAGGGAAGAAGAGR	2	S17(Phospho)	S(2): 0.0; S(17): 100.0
Q9UEY8	SPEKIEEVLSPEGSPsKsPSK	1	S16(Phospho); S18(Phospho)	S(1): 0.0; S(10): 0.0; S(14): 98.9; S(16): 10.7; S(18): 89.3; S(20): 1.1
Q9C0C2	RDsLGAYASQDANEQGQDLGK	2	S3(Phospho)	S(3): 99.6; Y(7): 0.4; S(9): 0.0
P78559	SHWDDsTsDSELEK	1	S6(Phospho); S8(Phospho)	S(1): 0.0; S(6): 94.2; T(7): 11.1; S(8): 88.9; S(10): 5.8
P46821	AAEAGGAEEQYGFLTtPTK	2	T16(Phospho)	Y(11): 0.0; T(15): 0.5; T(16): 92.6; T(18): 6.9
Q14498	DKsPVREPIDNLTPEER	4	S3(Phospho)	S(3): 100.0; T(13): 0.0
Q9ULX3	KDDsDDDGGGWITPSNIK	3	S4(Phospho)	S(4): 100.0; T(13): 0.0; S(15): 0.0
Q9Y2X7	HGsGADsDYENTQSGDPLLGLEGK	4	S3(Phospho); S7(Phospho)	S(3): 10.5; S(7): 81.3; Y(9): 97.6; T(12): 10.5; S(14): 0.2
P46821	ASVSPMDEPVPDSEsPIEK	5	S15(Phospho)	S(2): 0.0; S(4): 0.0; S(13): 50.0; S(15): 50.0
Q6PKG0	KNTFTAWsDEESDYEIDDRDVNK	3	S8(Phospho)	T(3): 0.0; T(5): 0.1; S(8): 99.9; S(12): 0.0; Y(14): 0.0
Q86XP3	YMAENPTAGVVQEEEEDNLEYDsDGNPIAPTKK	3	S23(Phospho)	Y(1): 0.0; T(7): 0.0; Y(21): 3.7; S(23): 96.2; T(31): 0.0
Q5T5U3	RNsEGSELScTEGSLTSSLDSR	5	S3(Phospho); C10(Carbamidomethyl)	S(3): 89.7; S(6): 10.2; S(9): 0.2; T(11): 0.0; S(14): 0.0; T(16): 0.0; S(17): 0.0; S(18): 0.0; S(21): 0.0
Q8N1G4	YTLENKEEGSLsDTEADAVSGQLPDPTTNPSAGK	2	S12(Phospho)	Y(1): 0.0; T(2): 0.0; S(10): 12.9; S(12): 74.1; T(14): 12.9; S(20): 0.0; T(27): 0.0; T(28): 0.0; S(31): 0.0
P31946	DNLTLWTSENQGDEGDAGEGEN	6		
Q01105	ELNSNHDGADETSEKEQQEAIEHIDEVQNEIDR	1		
Q9H8Y8	VGDSTPVSEKPVSAAVDANASEsP	3	S23(Phospho)	S(4): 0.0; T(5): 0.0; S(8): 0.0; S(13): 0.0; S(21): 7.6; S(23): 92.4
Q14697	VSQGSKDPAEGDGAQPEETPRDGDKPEETQGK	2		
Q08211	DKDDDGGEDDDANcNLIcGDEYGPETR	1	C14(Carbamidomethyl); C18(Carbamidomethyl)	
Q9UHD8	sFEVEEVETPNSTPPR	3	S1(Phospho)	S(1): 100.0; T(9): 0.0; S(12): 0.0; T(13): 0.0
Q09666	SSKAsLGsLEGEAEAEASSPK	8	S5(Phospho); S8(Phospho)	S(1): 9.0; S(2): 9.0; S(5): 91.0; S(8): 91.0; S(18): 0.0; S(19): 0.0
Q96N67	SLSNsNPDISGTPTSPDDEVR	7	S5(Phospho)	S(1): 0.9; S(3): 8.6; S(5): 90.5; S(10): 0.0; T(12): 0.0; T(14): 0.0; S(15): 0.0
P78559	GQDVVQEWQETsPTREEPAGEQK	4	S12(Phospho)	T(11): 0.1; S(12): 99.7; T(14): 0.1
Q3KQU3	RSsQPsPTAVPASDSPPTK	5	S3(Phospho); S6(Phospho)	S(2): 9.7; S(3): 90.3; S(6): 90.3; T(8): 9.7; S(13): 0.0; S(15): 0.0; T(18): 0.0
Q9UBB9	KGAAEEAELEDsDDEEKPVKQDDFPK	1	S12(Phospho)	S(12): 100.0
Q9Y2X7	HGsGADSDYENTQSGDPLLGLEGK	6	S3(Phospho)	S(3): 98.5; S(7): 0.7; Y(9): 0.7; T(12): 0.0; S(14): 0.0
Q3YEC7	RADDFPVRDDPsDVtDEDEGPAEPPPPPK	2	S12(Phospho); T15(Phospho)	S(12): 100.0; T(15): 100.0
Q02952	LSAEyEKVELPSEEQVSGSQGPSEEKPAPLATEVFDEK	2	Y5(Phospho)	S(2): 50.0; Y(5): 50.0; S(12): 0.1; S(17): 0.0; S(19): 0.0; S(23): 0.0; T(32): 0.0
Q71U36	DYEEVGVDSVEGEGEEEGEEY	1		
Q969E4	REDEGEPGDEGQLEDEGsQEKQGR	2	S18(Phospho)	S(18): 100.0
P46940	DSLHEKFPDAGEDELLK	3		
Q14137	IGDEYAEDssDEEDIR	1	S9(Phospho); S10(Phospho)	Y(5): 0.0; S(9): 100.0; S(10): 100.0
Q09666	AsLGsLEGEAEAEASSPK	4	S2(Phospho); S5(Phospho)	S(2): 100.0; S(5): 100.0; S(15): 0.0; S(16): 0.0
Q7Z6Z7	REESPMDVDQPsPSAQDTQSIASDGTPQGEK	1	S12(Phospho)	S(4): 0.0; S(12): 88.3; S(14): 11.7; T(18): 0.0; S(20): 0.0; S(23): 0.0; T(26): 0.0
Q15435	DGEERGEEDPEEEHELPVDMETINLDRDAEDVDLNHYR	1		
P54105	FEEESKEPVADEEEEDsDDDVEPITEFR	4	S17(Phospho)	S(5): 0.0; S(17): 100.0; T(25): 0.0

P23528	HELQANcYEEVKDR	4	C7(Carbamidomethyl)	
P31323	RAsVcAEAYNPDEEEDDAESR	1	S3(Phospho); C5(Carbamidomethyl)	S(3): 91.0; Y(9): 9.0; S(20): 0.0
Q8TE77	APSEEELHGDQTDFGQGSQsPQKQEEQR	1	S20(Phospho)	S(3): 0.0; T(12): 0.0; S(18): 50.0; S(20): 50.0
P50502	KVEEDLKADEPssEEsDLEIDK	1	S12(Phospho); S13(Phospho); S16(Phospho)	S(12): 100.0; S(13): 100.0; S(16): 100.0
P42167	GPPDFssDEEREPTPVLGSGAAAAGR	5	S6(Phospho); S7(Phospho)	S(6): 100.0; S(7): 100.0; T(14): 0.0; S(19): 0.0
P31948	KETKPEPMEEDLPENKK	4		
Q99733	EFITGDVEPTDAESEWHsENEEEEKLAGDMk	3	S18(Phospho); C-Term(Oxidation)	T(4): 0.0; T(10): 0.1; S(14): 0.2; S(18): 99.7
O75717	SHILEDDENsVDISMLK	2	S10(Phospho)	S(1): 0.0; S(10): 100.0; S(14): 0.0
Q13442	SLDsDEsEDEEDDYQQK	1	S4(Phospho); S7(Phospho)	S(1): 0.5; S(4): 99.5; S(7): 100.0; Y(14): 0.0
O00203	EGDELEDNGKNFYEsDDDQKEK	1	S15(Phospho)	Y(13): 0.4; S(15): 99.6
Q8NEN9	AQNEFKDEAQSLSHsPK	1	S15(Phospho)	S(11): 0.0; S(13): 0.4; S(15): 99.6
O60231	LLEDsEEsSEETVSR	1	S5(Phospho); S8(Phospho)	S(5): 99.2; S(8): 91.8; S(9): 9.0; T(12): 0.0; S(14): 0.0
O95365	HFKDEDEDEDVAsPDGLGR	2	S13(Phospho)	S(13): 100.0
Q5SW79	NQATSAtSEKDNDDDQSDKGTYTIELENPNSEEVEAR	7	T7(Phospho)	T(4): 0.5; S(5): 13.2; T(7): 72.8; S(8): 13.2; S(17): 0.1; T(21): 0.1; Y(22): 0.0; T(23): 0.0; S(31): 0.0
P27797	AKIDDPTDSKPEDWDKPEHIPDPDAK	14		
Q07954	cDNDNDcGDNSDEAGcSHScSSTQFK	1	C1(Carbamidomethyl); C7(Carbamidomethyl); C16(Carbamidomethyl); C20(Carbamidomethyl)	
O43934	KPDSENVLGEDESsDDQDMEVNESAQNNLTK	1	S14(Phospho)	S(4): 0.2; S(13): 70.9; S(14): 14.5; S(24): 14.5; T(30): 0.0
Q9NTI5	AEsPESSAIESTQSTPQK	2	S3(Phospho)	S(3): 100.0; S(6): 0.0; S(7): 0.0; S(11): 0.0; T(12): 0.0; S(14): 0.0; T(15): 0.0
O43719	LFEEsDDKEDEDADGKEVEDADEK	1	S5(Phospho)	S(5): 100.0
Q8IYB3	KETEsEAEDNLDDLEK	3	S5(Phospho)	T(3): 50.0; S(5): 50.0
P51114	RGPNYTSGYGTNSELSNPsETESER	1	S19(Phospho)	Y(5): 0.0; T(6): 0.0; S(7): 0.0; Y(9): 0.0; T(11): 0.0; S(13): 0.0; S(16): 0.0; S(19): 98.4; T(21): 1.6; S(23): 0.0
P78559	DRWPEVSPEDTQSLSLSEEsPSKETSLDVSSK	13	S20(Phospho)	S(7): 0.0; T(11): 0.0; S(13): 0.0; S(15): 0.0; S(17): 49.6; S(20): 49.6; S(22): 0.7; T(25): 0.0; S(26): 0.0; S(30): 0.0; S(31): 0.0
Q9H0G5	VEENPDADsDFDAKssADDEIEETR	2	S9(Phospho); S15(Phospho); S16(Phospho)	S(9): 100.0; S(15): 100.0; S(16): 100.0; T(24): 0.0
O95674	VDGETAsDSESRAESAPLPVSADDTPEVLNR	5	S7(Phospho)	T(5): 0.0; S(7): 10.8; S(9): 1.4; S(11): 87.6; S(15): 0.2; S(21): 0.0; T(25): 0.0
O00264	LLKEGEEPTVYsDEEEPKDESAR	6	S12(Phospho)	T(9): 0.0; Y(11): 7.7; S(12): 92.3; S(21): 0.0
Q15154	NVRsDIsDQEEDEESEGcPVSINLSK	2	S4(Phospho); S7(Phospho); C18(Carbamidomethyl)	S(4): 100.0; S(7): 100.0; S(15): 0.0; S(21): 0.0; S(25): 0.0
P78559	DRGLDsGAETEEEKDTWEEK	1	S6(Phospho)	S(6): 98.6; T(10): 1.4; T(16): 0.0
Q9H1E3	VVDYSQFQEsDDADEDYGRDSGPPTKK	2	S10(Phospho)	Y(4): 0.1; S(5): 0.4; S(10): 99.6; Y(17): 0.0; S(21): 0.0; T(25): 0.0
P25788	ESLKEEDEsDDDNm	1	S9(Phospho); C-Term(Oxidation)	S(2): 0.0; S(9): 100.0
P06733	DATNVGDEGGFAPNILENK	3		
Q09666	LKsEDGVEGDLGETQSR	4	S3(Phospho)	S(3): 100.0; T(14): 0.0; S(16): 0.0
Q12906	DSSKGEDSAEEtEAKPAVVAPAPVVEAVSTPSAAFPSDATAENVK	6	T12(Phospho)	S(2): 6.9; S(3): 6.9; S(8): 6.9; T(12): 79.4; S(29): 0.0; T(30): 0.0; S(32): 0.0; S(37): 0.0; T(40): 0.0
Q8TEQ0	LDVKsIDDEDVDENEDDVYGNSSGR	1	S5(Phospho)	S(5): 100.0; Y(19): 0.0; S(22): 0.0; S(23): 0.0
Q9BXF6	SNsSSEAVLGQEELSAQAK	5	S3(Phospho)	S(1): 0.2; S(3): 33.3; S(4): 33.3; S(5): 33.3; S(15): 0.0
P43487	DTHEDHDTSTENTDESNHDPQFEPIVSLPEQEIK	5		
P25788	ESLKEEDEsDDDNM	2	S9(Phospho)	S(2): 0.0; S(9): 100.0
P67809	NYQQNYQNsESGEKNEGSESAPEGQAQQR	4	S9(Phospho)	Y(2): 0.0; Y(6): 0.8; S(9): 49.6; S(11): 49.6; S(18): 0.0; S(20): 0.0
O95817	SQsPAASDcSSSSSSASLPSSGR	5	S3(Phospho); C9(Carbamidomethyl)	S(1): 8.3; S(3): 91.7; S(7): 0.0; S(10): 0.0; S(11): 0.0; S(12): 0.0; S(13): 0.0; S(14): 0.0; S(15): 0.0; S(17): 0.0; S(20): 0.0; S(21): 0.0
Q9Y2X3	HIKEEPLsEEEPcTSTAIASPEK	1	S8(Phospho); C13(Carbamidomethyl)	S(8): 100.0; T(14): 0.0; S(15): 0.0; T(16): 0.0; S(20): 0.0
Q8IYB3	KEtEsEAEDNLDDLEK	3	T3(Phospho); S5(Phospho)	T(3): 100.0; S(5): 100.0
O00193	RSAsPDDDLGSSNWEAADLGNEER	2	S4(Phospho)	S(2): 8.2; S(4): 82.8; S(11): 8.2; S(12): 0.8
P23588	SLENETLNKEEDcHsPTSKPPKPDQPLK	4	C13(Carbamidomethyl); S15(Phospho)	S(1): 0.0; T(6): 0.0; S(15): 94.9; T(17): 2.5; S(18): 2.5
P78559	AELEEMEEVHPsDEEEEDATKAEGFYQK	1	S12(Phospho)	S(12): 100.0; T(20): 0.0; Y(26): 0.0
Q66K74	sASPHDVDLcLVSPcEFEHR	4	S1(Phospho); C10(Carbamidomethyl); C15(Carbamidomethyl)	S(1): 50.0; S(3): 50.0; S(13): 0.0
Q9UH62	YNDWsDDDDDSNESK	1	S5(Phospho)	Y(1): 0.0; S(5): 100.0; S(11): 0.0; S(14): 0.0
Q96F86	SQDVAVsPQQQQcSK	1	S7(Phospho); C13(Carbamidomethyl)	S(1): 0.0; S(7): 100.0; S(14): 0.0
Q9UBB9	GAAEEAELEDsDDEEKPVKQDDFPK	1	S11(Phospho)	S(11): 100.0
Q05209	HNIAGTTHSGAEKDVDVSEDsPPPLPER	2	S21(Phospho)	T(6): 0.0; T(7): 0.0; S(9): 0.0; S(18): 2.0; S(21): 98.0
P55010	EAEEEssGGEEEDEDENIEVVYSK	2	S6(Phospho); S7(Phospho)	S(6): 100.0; S(7): 100.0; Y(22): 0.0; S(23): 0.0
Q9NP74	SEHQNSsPTcQEDEEDVR	5	S7(Phospho); C10(Carbamidomethyl)	S(1): 0.0; S(6): 92.4; S(7): 7.0; T(9): 0.6
P49815	SQsGTLDGESAAWSASGEDSR	3	S3(Phospho)	S(1): 6.8; S(3): 86.4; T(5): 6.8; S(10): 0.0; S(14): 0.0; S(16): 0.0; S(20): 0.0
Q9UQ35	HGGSPQPLAtTPLSQEPVNPPSEAsPTR	3	T10(Phospho); S25(Phospho)	S(4): 100.0; T(10): 0.0; T(11): 0.0; S(14): 0.0; S(22): 0.1; S(25): 90.3; T(27): 9.6
Q9Y2X7	SQSDLDDQHDYDSVAsDEDTDQEPLR	2	S16(Phospho)	S(1): 0.0; S(3): 0.0; Y(11): 0.0; S(13): 0.0; S(16): 100.0; T(20): 0.0
Q9C0C2	VPssDEEVVEEPQSR	3	S3(Phospho); S4(Phospho)	S(3): 100.0; S(4): 100.0; S(14): 0.0
Q96KC8	EDAEGVAAEEEQEGDsGEQETGATDARPR	1	S16(Phospho)	S(16): 100.0; T(21): 0.0; T(24): 0.0
P20020	IEDsEPHIPLIDDTDAEDDAPTK	2	S4(Phospho)	S(4): 100.0; T(14): 0.0; T(22): 0.0
Q5VSL9	AAsPPASASDLIEQQQK	1	S3(Phospho)	S(3): 100.0; S(7): 0.0; S(9): 0.0
Q32MZ4	cTLPEHESPsQDISDAcEAESTER	2	C1(Carbamidomethyl); S10(Phospho); C17(Carbamidomethyl)	T(2): 0.0; S(8): 1.2; S(10): 98.8; S(14): 0.0; S(21): 0.0; T(22): 0.0
Q6L8Q7	EAKPGAAEPEVGVPSSLSPSSPsSSWTETDVEER	9	S23(Phospho)	S(15): 0.0; S(16): 0.0; S(18): 0.0; S(20): 5.9; S(21): 5.9; S(23): 40.7; S(24): 40.7; S(25): 5.9; T(27): 0.9; T(29): 0.1
P78559	GFKsPPcEDFSVTGESEK	2	S4(Phospho); C7(Carbamidomethyl)	S(4): 100.0; S(11): 0.0; T(13): 0.0; S(16): 0.0
P06748	LAADEDDDDDDEEDDDEDDDDDDFDDEEAEEKAPVKK	3		
Q8N2F6	SSKSAEDLTDGsYDDVLNAEQLQK	5	S12(Phospho)	S(1): 1.5; S(2): 1.5; S(4): 1.5; T(9): 0.2; S(12): 84.3; Y(13): 11.0
Q9UPN3	EVEEELATSGGQsPTGEQIPQFQQR	1	S13(Phospho)	T(8): 0.0; S(9): 0.0; S(13): 50.0; T(15): 50.0
Q969E4	NEGNLENEGKPEDEVEPDDEGKsDEEEKPDVEGK	3	S23(Phospho)	S(23): 100.0
Q9NXG2	FTDKDQQPsGsEGEDDDAEAALKK	2	S9(Phospho); S11(Phospho)	T(2): 0.0; S(9): 100.0; S(11): 100.0
Q8IVF2	DAHDVsPTSTDTEAQLTVER	7	S6(Phospho)	S(6): 92.3; T(8): 7.6; S(9): 0.1; T(10): 0.0; T(12): 0.0; T(17): 0.0
P47712	HIVSNDSSDsDDESHEPK	2	S10(Phospho)	S(4): 0.0; S(7): 0.0; S(8): 0.0; S(10): 100.0; S(14): 0.0
P07355	RAEDGSVIDYELIDQDAR	1		
P29692	KPATPAEDDEDDDIDLFGsDNEEEDKEAAQLREER	10	S19(Phospho)	T(4): 0.0; S(19): 100.0
P78559	TEATQGLDYVPSAGTIsPTSSLEEDKGFK	1	S17(Phospho)	T(1): 0.0; T(4): 0.0; Y(9): 0.0; S(12): 0.0; T(15): 0.0; S(17): 98.5; T(19): 1.4; S(20): 0.0; S(21): 0.0
P11274	GRsSESScGVDGDYEDAELNPR	4	S3(Phospho); C8(Carbamidomethyl)	S(3): 25.0; S(4): 25.0; S(6): 25.0; S(7): 25.0; Y(14): 0.0
Q9H6Y2	TcEERPAEDGsDEEDPDSMEAPTR	3	C2(Carbamidomethyl); S11(Phospho)	T(1): 0.0; S(11): 100.0; S(18): 0.0; T(23): 0.0
Q8TEW0	RSsDPALIGLSTSVSDSNFSSEEPSR	2	S3(Phospho)	S(2): 50.0; S(3): 50.0; S(11): 0.0; T(12): 0.0; S(13): 0.0; S(15): 0.0; S(17): 0.0; S(20): 0.0; S(21): 0.0; S(25): 0.0
P08648	LLESSLSsSEGEEPVEYK	5	S8(Phospho)	S(4): 0.0; S(5): 0.0; S(7): 0.0; S(8): 94.6; S(9): 5.4; Y(17): 0.0
Q13501	IALESEGRPEEQMEsDNcsGGDDDWTHLSSK	1	S15(Phospho); C18(Carbamidomethyl); S19(Phospho)	S(5): 0.0; S(15): 100.0; S(19): 99.9; T(26): 0.0; S(29): 0.0; S(30): 0.0
Q9UHB6	EGHSLEMENENLVENGADsDEDDNSFLK	2	S19(Phospho)	S(4): 0.0; S(19): 99.5; S(25): 0.5
P78559	GLDsGAEtEEEKDTWEEK	4	S4(Phospho); T8(Phospho)	S(4): 100.0; T(8): 100.0; T(14): 0.0
O60524	NPYLLsEEEDDDVDGDVNVEKNETEPPK	1	S6(Phospho)	Y(3): 0.0; S(6): 100.0; T(24): 0.0
Q9C0C2	RFsEGVLQSPSQDQEK	3	S3(Phospho)	S(3): 100.0; S(9): 0.0; S(11): 0.0
Q02952	GLAEVQQDGEAEEGATsDGEKKR	8	S17(Phospho)	T(16): 50.0; S(17): 50.0
P63208	VIQWcTHHKDDPPPPEDDENKEK	3	C5(Carbamidomethyl)	
P78559	KVAELEEEQSQGsSSYSDWVK	6	S13(Phospho)	S(10): 0.0; S(13): 99.4; S(14): 0.6; S(15): 0.0; Y(16): 0.0; S(17): 0.0
Q9NP74	EQEESLGsPVHHSPFDAQTTGDGTEDPSLTALR	1	S8(Phospho)	S(5): 18.3; S(8): 80.2; S(13): 1.1; T(19): 0.3; T(20): 0.1; T(24): 0.0; S(28): 0.0; T(30): 0.0
Q63HN8	EAAEPLSEPKEDQEAAELLsEPEEESER	2	S20(Phospho)	S(7): 0.0; S(20): 99.7; S(26): 0.3
Q9Y2D5	GQKsPGALETPSAAGSQGNTASQGK	2	S4(Phospho)	S(4): 100.0; T(10): 0.0; S(12): 0.0; S(16): 0.0; T(20): 0.0; S(22): 0.0
P28715	FDSSLLssDDETK	2	S7(Phospho); S8(Phospho)	S(3): 0.0; S(4): 0.0; S(7): 100.0; S(8): 100.0; T(12): 0.0
P28482	VADPDHDHTGFLTEyVATR	4	Y15(Phospho)	T(9): 0.0; T(13): 0.0; Y(15): 100.0; T(18): 0.0
P40855	NATDLQNSsMSEEELTK	5	S9(Phospho)	T(3): 0.0; S(8): 0.3; S(9): 49.8; S(11): 49.8; T(16): 0.0

Q9H2K8	NGPLNEsQEDEEDSEHGTSLNR	1	S7(Phospho)	S(7): 100.0; S(14): 0.0; T(18): 0.0; S(19): 0.0
P84157	QEEEQDLDGEKGPSsEGPEEEDGEGFSFK	2	S15(Phospho)	S(14): 0.2; S(15): 99.8; S(27): 0.0
P78346	KPRPsEGDEDcLPASK	1	S5(Phospho); C11(Carbamidomethyl)	S(5): 100.0; S(15): 0.0
Q99733	EFITGDVEPTDAESEWHsENEEEEKLAGDMK	1	S18(Phospho)	T(4): 0.0; T(10): 0.1; S(14): 12.7; S(18): 87.3
P21359	KVsVSESNVLLDEEVLTDPK	2	S3(Phospho)	S(3): 84.9; S(5): 7.6; S(7): 7.6; T(17): 0.0
P46821	DVMsDETNNEETESPSQEFVNITK	2	S4(Phospho)	S(4): 90.4; T(7): 9.6; T(12): 0.0; S(14): 0.0; S(16): 0.0; T(23): 0.0
P35579	IAQLEEELEEEQGNTELINDR	2		
Q9NQC3	RGSsGsVDETLFALPAASEPVIR	6	S4(Phospho); S6(Phospho)	S(3): 52.6; S(4): 52.6; S(6): 93.5; T(10): 1.3; S(18): 0.0
Q05209	TPLSFTNPLHsDDsDSDERNSDGAVTQNK	1	S11(Phospho); S14(Phospho)	T(1): 0.0; S(4): 0.0; T(6): 0.1; S(11): 99.9; S(14): 97.5; S(16): 2.5; S(21): 0.0; T(26): 0.0
P46821	QGsPDQVsPVSEMTSTSLYQDK	3	S3(Phospho); S8(Phospho)	S(3): 100.0; S(8): 100.0; S(11): 0.0; T(14): 0.0; S(15): 0.0; T(16): 0.0; S(17): 0.0; Y(19): 0.0
Q5VT52	DVEDMELsDVEDDGSK	2	S8(Phospho)	S(8): 100.0; S(15): 0.0
O43395	WDEQTSNTKGDDDEEsDEEAVKK	1	S16(Phospho)	T(5): 0.0; S(6): 0.0; T(8): 0.0; S(16): 100.0
O00505	NVPQEESLEDsDVDADFK	4	S11(Phospho)	S(7): 0.0; S(11): 100.0
Q9UHB6	HEVEKSEISENTDASGKIEK	1		
Q86VR2	AMDNHsDsEEELAAFcPQLDDSTVAR	3	S6(Phospho); S8(Phospho); C16(Carbamidomethyl)	S(6): 100.0; S(8): 100.0; S(22): 0.0; T(23): 0.0
Q9UKA4	sLSEEVESSESGELPEVDVK	4	S1(Phospho)	S(1): 90.9; S(3): 9.1; S(8): 0.0; S(9): 0.0; S(11): 0.0
P46821	sPSEARQDVDLcLVSScEYK	2	S1(Phospho); C12(Carbamidomethyl); C17(Carbamidomethyl)	S(1): 50.0; S(3): 50.0; S(15): 0.0; S(16): 0.0; Y(19): 0.0
O75396	NLGsINTELQDVQR	2	S4(Phospho)	S(4): 100.0; T(7): 0.0
Q9BV36	AEGLEEADTGASGcHSHPEEQPTsIsPSR	2	C14(Carbamidomethyl); S24(Phospho); S26(Phospho)	T(9): 0.0; S(12): 0.0; S(16): 0.0; T(23): 86.1; S(24): 13.9; S(26): 13.9; S(28): 86.1
Q7Z3C6	HPEPVPEEGsEDELPPQVHKV	4	S10(Phospho)	S(10): 100.0
Q6ZRP7	DNLLDTYSADQGDSsEGGTLAR	5	S15(Phospho)	T(6): 0.0; Y(7): 0.0; S(8): 0.0; S(14): 50.0; S(15): 50.0; T(19): 0.0
Q9UEW8	TEDGDWEWsDDEMDEKSEEGK	1	S9(Phospho)	T(1): 0.0; S(9): 100.0; S(17): 0.0
Q96AT1	NSSLLsFDNEDENE	2	S6(Phospho)	S(2): 0.0; S(3): 0.0; S(6): 100.0
P78559	GLDsGAETEEEKDTWEEK	4	S4(Phospho)	S(4): 99.8; T(8): 0.2; T(14): 0.0
O14745	SAsSDTSEELNSQDSPPK	3	S3(Phospho)	S(1): 50.0; S(3): 50.0; S(4): 0.0; T(6): 0.0; S(7): 0.0; S(12): 0.0; S(15): 0.0
P54725	EDKsPSEESAPTTSPESVSGSVPSSGSSGR	7	S4(Phospho)	S(4): 98.5; S(6): 0.2; S(9): 1.3; T(12): 0.0; T(13): 0.0; S(14): 0.0; S(17): 0.0; S(19): 0.0; S(21): 0.0; S(24): 0.0; S(25): 0.0; S(27): 0.0; S(28): 0.0
Q9NPQ8	GLMAGGRPEGQYsEDEDtDTDEYKEAK	1	S13(Phospho); T18(Phospho)	Y(12): 50.0; S(13): 50.0; T(18): 97.7; T(20): 2.2; Y(23): 0.1
Q09666	GKGGVTGsPEASISGSK	3	S8(Phospho)	T(6): 0.1; S(8): 99.9; S(12): 0.0; S(14): 0.0; S(16): 0.0
O94979	DSDQVAQsDGEESPAAEEQLLGEHIK	3	S8(Phospho)	S(2): 0.0; S(8): 100.0; S(13): 0.0
P46821	TDATDGKDYNASASTIsPPSSMEEDKFSR	3	S17(Phospho)	T(1): 0.0; T(4): 0.0; Y(9): 0.0; S(12): 0.0; S(14): 0.0; T(15): 0.0; S(17): 2.7; S(20): 94.5; S(21): 2.7; S(28): 0.1
O00193	TGEEDKKINEELESQYQQSMDSK	3		
Q13557	KPDGVKEsTESSNTTIEDEDVK	6	S8(Phospho)	S(8): 92.9; T(9): 7.0; S(11): 0.0; S(12): 0.0; T(14): 0.0; T(15): 0.0
Q7L1V2	EGGGVHAVPPDPEDEGLEETGSKDKDQPPSPsPPPQSEALSSTSR	2	S32(Phospho)	T(20): 0.0; S(22): 0.0; S(30): 5.7; S(32): 92.1; S(37): 0.4; S(41): 0.4; S(42): 0.4; T(43): 0.4; S(44): 0.4
Q9NZN4	GPDEAMEDGEEGsDDEAEWVVTK	2	S13(Phospho)	S(13): 100.0; T(22): 0.0
P35579	GAGDGsDEEVDGKADGAEAKPAE	2	S6(Phospho)	S(6): 100.0
O00264	GDQPAASGDsDDDEPPPLPR	9	S10(Phospho)	S(7): 0.1; S(10): 99.9
Q9C0C2	NRsAEEGELAESK	2	S3(Phospho)	S(3): 100.0; S(12): 0.0
P55196	ADHRSsPNVANQPPsPGGK	1	S6(Phospho); S15(Phospho)	S(5): 50.0; S(6): 50.0; S(15): 100.0
Q09666	LPSGsGAAsPTGSAVDIR	7	S5(Phospho); S9(Phospho)	S(3): 8.7; S(5): 91.3; S(9): 99.9; T(11): 0.1; S(13): 0.0
Q92769	MLPHAPGVQMQAIPEDAVHEDsGDEDGEDPDKR	2	S22(Phospho)	S(22): 100.0
Q14008	HsTSGTDEGEDGDEPDDGSNDVVDLLPR	7	S2(Phospho)	S(2): 85.6; T(3): 11.3; S(4): 1.6; T(6): 1.6; S(19): 0.0
O95671	HDSIPAADTFEDLsDVEGGGSEPTQR	2	S14(Phospho)	S(3): 2.5; T(9): 2.5; S(14): 95.0; S(21): 0.0; T(24): 0.0
P67809	EDGNEEDKENQGDETQGQQPPQR	1		
Q8N122	VLDTSSLTQsAPAsPTNK	1	S10(Phospho); S14(Phospho)	T(4): 0.0; S(5): 0.0; S(6): 0.0; T(8): 10.6; S(10): 89.4; S(14): 89.4; T(16): 10.6
P46821	DIKPQLELIEDEEKLKETEPVEAYVIQK	2		
Q9H1E3	VVDYSQFQEsDDADEDYGRDSGPPTK	1	S10(Phospho)	Y(4): 0.0; S(5): 0.0; S(10): 100.0; Y(17): 0.0; S(21): 0.0; T(25): 0.0
P08651	NWTEDMEGGISsPVKK	2	S12(Phospho)	T(3): 0.0; S(11): 0.5; S(12): 99.5
Q13177	GTEAPAVVtEEEDDDEETAPPVIAPRPDHTK	3	T9(Phospho)	T(2): 0.0; T(9): 100.0; T(18): 0.0; T(30): 0.0
P08238	IEDVGsDEEDDSGK	1	S6(Phospho)	S(6): 100.0; S(12): 0.0
P07900	NPDDITNEEYGEFYK	1		
P60660	NKDQGTYEDYVEGLR	2		
Q4G0J3	SRPTsEGsDIESTEPQK	1	S5(Phospho); S8(Phospho)	S(1): 2.7; T(4): 97.3; S(5): 97.3; S(8): 2.7; S(12): 0.0; T(13): 0.0
O43493	LSPHAFKTEsGEETDLISPPQEEVK	5	S10(Phospho)	S(2): 0.0; T(8): 0.0; S(10): 99.8; T(14): 0.2; S(18): 0.0
O00629	NVPHEDIcEDsDIDGDYR	4	C8(Carbamidomethyl); S11(Phospho)	S(11): 100.0; Y(17): 0.0
P46821	KsPSEARQDVDLcLVSScEYK	2	S2(Phospho); C13(Carbamidomethyl); C18(Carbamidomethyl)	S(2): 99.9; S(4): 0.1; S(16): 0.0; S(17): 0.0; Y(20): 0.0
P24534	YGPADVEDTTGSGATDSKDDDDIDLFGsDDEEESEEAK	3	S28(Phospho)	Y(1): 0.0; T(9): 0.0; T(10): 0.0; S(12): 0.0; T(15): 0.0; S(17): 0.0; S(28): 81.0; S(34): 19.0
Q7Z460	RQsSGSATNVASTPDNR	5	S3(Phospho)	S(3): 99.2; S(4): 0.8; S(6): 0.0; T(8): 0.0; S(12): 0.0; T(13): 0.0
P00533	GSHQIsLDNPDYQQDFFPK	1	S6(Phospho)	S(2): 0.1; S(6): 99.9; Y(12): 0.0
P67936	KIQALQQQADEAEDR	1		
Q13501	RFsFccSPEPEAEAEAAAGPGPcER	2	S3(Phospho); C5(Carbamidomethyl); C6(Carbamidomethyl); C23(Carbamidomethyl)	S(3): 90.5; S(7): 9.5
Q5JTV8	GLRDsHsSEEDEASSQTDLSQTISK	2	S5(Phospho); S7(Phospho)	S(5): 91.4; S(7): 91.4; S(8): 17.2; S(14): 0.0; S(15): 0.0; T(17): 0.0; S(20): 0.0; T(22): 0.0; S(24): 0.0
Q147X3	cPFPAGAALAccsEDEEDDEEHEGGGSR	1	C1(Carbamidomethyl); C11(Carbamidomethyl); C12(Carbamidomethyl); S13(Phospho)	S(13): 100.0; S(27): 0.0
P34932	NKEDQYDHLDAADMTK	3		
Q8NHQ9	KREEGsDIEDEDMEELLNDTR	1	S6(Phospho)	S(6): 100.0; T(20): 0.0
P61981	DNLTLWTSDQQDDDGGEGNN	7		
Q96RT1	TEPHDSDcSVDLGIsKSTEDLSPQK	3	C8(Carbamidomethyl); S15(Phospho)	T(1): 0.0; S(6): 0.0; S(9): 0.0; S(15): 46.7; S(17): 46.7; T(18): 6.5; S(22): 0.0
P84157	QEEEQDLDGEKGPSSEGPEEEDGEGFSFKYsPGK	2	S31(Phospho)	S(14): 0.0; S(15): 0.0; S(27): 4.1; Y(30): 17.4; S(31): 78.5
Q00839	AKsPQPPVEEEDEHFDDTVVcLDTYNcDLHFK	1	S3(Phospho); C21(Carbamidomethyl); C27(Carbamidomethyl)	S(3): 99.9; T(18): 0.1; T(24): 0.0; Y(25): 0.0
Q9Y696	DEFTNTcPSDKEVEIAYSDVAK	3	C7(Carbamidomethyl)	
Q8NFQ8	DHQEVETEGPESADTGDKSESPDEANVGKHPK	1		
Q7Z3C6	HPEPVPEEGsEDELPPQVHK	2	S10(Phospho)	S(10): 100.0
Q9Y265	DSIEKEHVEEISELFYDAK	4		
P35579	KLEEEQIILEDQNcK	1	C14(Carbamidomethyl)	
P13861	GDsEsEEDEDLEVPVPSR	2	S3(Phospho); S5(Phospho)	S(3): 100.0; S(5): 100.0; S(17): 0.0
Q8NE71	KAEQGsEEEGEGEEEEEEGGESK	1	S6(Phospho)	S(6): 100.0; S(22): 0.0
Q9Y4K1	MPLLELGGETtPPLStERSPEAVGSEcPSR	4	T11(Phospho); T16(Phospho); C27(Carbamidomethyl)	T(10): 50.0; T(11): 50.0; S(15): 3.3; T(16): 15.7; S(19): 80.9; S(25): 0.2; S(29): 0.0
P30622	TASESISNLSEAGsIK	2	S14(Phospho)	T(1): 0.0; S(3): 0.0; S(5): 0.0; S(7): 0.0; S(10): 0.0; S(14): 100.0
O75351	EGQPSPADEKGNDsDGEGESDDPEKK	2	S14(Phospho)	S(5): 0.0; S(14): 100.0; S(20): 0.0
Q92614	YSHSYLsDsDTEAKLTETNA	2	S7(Phospho); S9(Phospho)	Y(1): 0.0; S(2): 0.0; S(4): 0.0; Y(5): 0.3; S(7): 97.1; S(9): 85.5; T(11): 16.9; T(16): 0.0; T(18): 0.0
Q68DQ2	YEDKPEPEVDALGsPPALLK	1	S14(Phospho)	Y(1): 0.0; S(14): 100.0
Q8TEU7	LPEGPVDsEDDEEEDEEIDRTDPLQGR	2	S8(Phospho)	S(8): 100.0; T(21): 0.0
P51858	KGNAEGssDEEGKLVIDEPAK	2	S7(Phospho); S8(Phospho)	S(7): 100.0; S(8): 100.0
P23193	KKEPAITSQNsPEAR	5	S11(Phospho)	T(7): 0.0; S(8): 0.0; S(11): 100.0
Q13283	SSsPAPADIAQTVQEDLR	20	S3(Phospho)	S(1): 4.1; S(2): 4.1; S(3): 91.8; T(12): 0.0
P16591	VQENDGKEPPPVVNyEEDAR	2	Y15(Phospho)	Y(15): 100.0
Q70E73	mEQLsDEEIDHGAEEDsDKEDQDLDK	2	N-Term(Acetyl); S5(Phospho); S17(Phospho)	S(5): 100.0; S(17): 100.0
P27816	KcsLPAEEDSVLEK	1	C2(Carbamidomethyl); S3(Phospho)	S(3): 100.0; S(10): 0.0
Q14839	FGTEELFKDEATDGGGDNKEGEDSSVIHYDDK	1		

Q9UDY2	SILKPSTPIPPQEGEEVGEsSEEQDNAPK	4	S20(Phospho)	S(1): 0.0; S(6): 0.0; T(7): 0.0; S(20): 50.0; S(21): 50.0
Q05655	RSDSASsEPVGIYQGFEK	5	S7(Phospho)	S(2): 1.2; S(4): 32.9; S(6): 32.9; S(7): 32.9; Y(13): 0.0
Q9UGP4	DYHVEcYHcEDcGLELNDEDGHR	2	C6(Carbamidomethyl); C9(Carbamidomethyl); C12(Carbamidomethyl)	
P63104	SVTEQGAELSNEER	1		
Q08752	DGSGDSHPDFPEDADIDLKDVDK	5		
Q9NYL9	YKDLDEDELLGNLsETELK	4	S14(Phospho)	Y(1): 0.0; S(14): 99.5; T(16): 0.5
P49792	NRPDYVsEEEEDDEDFETAVK	2	S7(Phospho)	Y(5): 0.1; S(7): 99.9; T(18): 0.0
Q969X1	AVsDSFGPGEWDDR	1	S3(Phospho)	S(3): 100.0; S(5): 0.0
P11142	NQTAEKEEFEHQQK	4		
Q9HCD6	DcSYGAVTsPTSTLESR	1	C2(Carbamidomethyl); S9(Phospho)	S(3): 0.0; Y(4): 0.0; T(8): 6.2; S(9): 87.1; T(11): 6.2; S(12): 0.5; T(13): 0.0; S(16): 0.0
Q15019	IYHLPDAEsDEDEDFKEQTR	7	S9(Phospho)	Y(2): 0.0; S(9): 100.0; T(19): 0.0
P78318	KAAQQQEEQEEKEEEDDEQTLHR	2		
Q7Z2K8	HRsPSGAGEGAScSDGPR	2	S3(Phospho); C13(Carbamidomethyl)	S(3): 99.2; S(5): 0.8; S(12): 0.0; S(14): 0.0
Q9H6F5	ALVEFESNPEETREPGsPPSVQR	2	S17(Phospho)	S(7): 0.0; T(12): 0.0; S(17): 99.9; S(20): 0.1
Q32MZ4	ALDSNsLENDDLSAPGREPGHFNPESR	3	S6(Phospho)	S(4): 12.0; S(6): 88.0; S(13): 0.0; S(26): 0.0
Q9C0C2	RDsLGAYASQDANEQGQDLGKR	1	S3(Phospho)	S(3): 100.0; Y(7): 0.0; S(9): 0.0
Q7Z6Z7	GSGTAsDDEFENLR	1	S6(Phospho)	S(2): 0.0; T(4): 0.0; S(6): 100.0
Q12959	NKDQSEQETSDADQHVTSNAsDSESSYR	2	S21(Phospho)	S(5): 0.0; T(9): 0.0; S(10): 0.0; T(17): 0.4; S(18): 13.8; S(21): 85.3; S(23): 0.4; S(25): 0.0; S(26): 0.0; Y(27): 0.0
Q5H9R7	IQQFDDGGsDEEDIWEEK	3	S9(Phospho)	S(9): 100.0
P07355	TDLEKDIISDTSGDFRK	1		
Q2M389	EKEEETKtSNGDLSDSTVSADPVVK	3	T8(Phospho)	T(6): 33.3; T(8): 33.3; S(9): 33.3; S(14): 0.0; S(16): 0.0; T(17): 0.0; S(19): 0.0
Q5JSH3	TKEYVSNDAAQsDDEEKLQSQPTDTDGGR	2	S12(Phospho)	T(1): 0.0; Y(4): 0.0; S(6): 0.0; S(12): 100.0; S(20): 0.0; T(23): 0.0; T(25): 0.0
Q969E4	GTDDsPKDSQEDLQER	6	S5(Phospho)	T(2): 0.0; S(5): 93.8; S(9): 6.2
Q08J23	AGEPNsPDAEEANSPDVTAGcDPAGVHPPR	5	S6(Phospho); C21(Carbamidomethyl)	S(6): 100.0; S(14): 0.0; T(18): 0.0
Q96EY5	DMQGLsLDAASQPSK	1	S6(Phospho)	S(6): 100.0; S(11): 0.0; S(14): 0.0
Q9UPN6	SSEPVKETVQTTQsPTPVEK	1	S14(Phospho)	S(1): 0.0; S(2): 0.0; T(8): 0.0; T(11): 0.0; T(12): 0.0; S(14): 7.1; T(16): 92.9
O60238	DHSSQSEEEVVEGEKEVEALKK	4		
Q1KMD3	REEDEPEERsGDETPGSEVPGDK	1	S10(Phospho)	S(10): 100.0; T(14): 0.0; S(17): 0.0
P35579	NKHEAMITDLEER	4		
Q9H4A3	EKPELSEPSHLNGPSsDPEAAFLSR	2	S16(Phospho)	S(6): 0.0; S(9): 0.0; S(15): 14.4; S(16): 85.5; S(24): 0.0
Q9C0C2	DsLGAYASQDANEQGQDLGK	2	S2(Phospho)	S(2): 99.6; Y(6): 0.3; S(8): 0.1
P46821	TTKtPEDGDYSYEIIEK	2	T4(Phospho)	T(1): 0.0; T(2): 0.1; T(4): 99.9; Y(10): 0.0; S(11): 0.0; Y(12): 0.0
Q86WR0	VENMSSNQDGNDsDEFM	2	S13(Phospho)	S(5): 0.0; S(6): 0.0; S(13): 100.0
O95365	AGAAAGDsDEESRADDKGVMDYYLK	1	S8(Phospho)	S(8): 98.6; S(12): 1.4; Y(22): 0.0; Y(23): 0.0
Q70Z53	KSEDSLLRNsDEEESASESELWK	2	S10(Phospho)	S(2): 0.0; S(5): 0.0; S(10): 100.0; S(15): 0.0; S(17): 0.0; S(19): 0.0
Q96N67	SRsLsNSNPDISGTPTSPDDEVR	5	S3(Phospho); S5(Phospho)	S(1): 19.1; S(3): 80.9; S(5): 80.9; S(7): 19.1; S(12): 0.0; T(14): 0.0; T(16): 0.0; S(17): 0.0
P42167	GPPDFsSDEEREPTPVLGSGAAAAGR	4	S6(Phospho)	S(6): 88.5; S(7): 11.3; T(14): 0.2; S(19): 0.0
Q14680	SNNWsLEDVTASDK	1	S5(Phospho)	S(1): 0.0; S(5): 100.0; T(10): 0.0; S(12): 0.0
Q09666	sSKASLGSLEGEAEAEASSPK	4	S1(Phospho)	S(1): 47.0; S(2): 47.0; S(5): 5.4; S(8): 0.7; S(18): 0.0; S(19): 0.0
P13010	KKDQVTAQEIFQDNHEDGPTAK	1		
P41236	IQEQEssGEEDSDLSPEER	3	S6(Phospho); S7(Phospho)	S(6): 100.0; S(7): 100.0; S(12): 0.0; S(15): 0.0
Q9ULT8	VSTLAGPSsDDENEEESKPEKEDEPQEDAK	2	S9(Phospho)	S(2): 0.0; T(3): 0.0; S(8): 14.6; S(9): 85.3; S(17): 0.0
P78559	TPGVGKEDAAEETVKPGPEEGTLEKEEK	1		
P02545	SVTVVEDDEDEDGDDLLHHHHVSGSR	6		
P01891	KGGSYSQAASSDSAQGsDVsLTAcKV	1	S17(Phospho); S20(Phospho); C24(Carbamidomethyl)	S(4): 0.0; Y(5): 0.0; S(6): 0.0; S(10): 0.0; S(11): 0.0; S(13): 0.0; S(17): 100.0; S(20): 100.0; T(22): 0.0
Q86W92	SQsTTFNPDDMSEPEFK	8	S3(Phospho)	S(1): 49.7; S(3): 49.7; T(4): 0.5; T(5): 0.0; S(12): 0.0
Q9NRF9	SRDEDNDEDEERLEEEEQNEEEEVDN	2		
O94885	scETLEGPQTVDTWPR	1	S1(Phospho); C2(Carbamidomethyl)	S(1): 99.8; T(4): 0.2; T(10): 0.0; T(13): 0.0
O14617	HRPsEADEEELAR	1	S4(Phospho)	S(4): 100.0
P46821	KLGDVSPtQIDVSQFGsFKEDTK	1	T8(Phospho); S17(Phospho)	S(6): 45.9; T(8): 45.9; S(13): 8.4; S(17): 96.5; T(22): 3.4
Q9UMX1	APsRKDsLESDSSTAIIPHELIR	1	S3(Phospho); S7(Phospho)	S(3): 98.4; S(7): 31.0; S(10): 31.0; S(12): 31.0; S(13): 6.8; T(14): 1.7
Q14315	VEAAEIVEGEDSAYSVR	2		
Q9NTJ3	RREEGPPPPsPDGAsSDAEPEPPSGR	4	S10(Phospho); S15(Phospho)	S(10): 100.0; S(15): 87.9; S(16): 12.1; S(24): 0.0
P46821	DIKPQLELIEDEEK	1		
Q8IV38	DSNPEDSGEGKKESLESEAELEGLQDAPAGPQVSEE	2		
P78559	TEATQGLDYVPSAGTIsPTsSLEEDKGFK	1	S17(Phospho); S20(Phospho)	T(1): 0.0; T(4): 0.0; Y(9): 0.0; S(12): 2.5; T(15): 0.4; S(17): 97.0; T(19): 84.7; S(20): 13.2; S(21): 2.2
O15258	VDPSLMEDsDDGPSLPTK	1	S9(Phospho)	S(4): 0.0; S(9): 100.0; S(14): 0.0; T(17): 0.0
Q01484	KTsLVIVESADNQPETcER	2	S3(Phospho); C17(Carbamidomethyl)	T(2): 7.0; S(3): 93.0; S(9): 0.0; T(16): 0.0
Q07666	GDSKKDDEENYLDLFSHK	2		
Q9HB20	TYsDTDScSDIPLEDPDRPVHcSK	6	S3(Phospho); C8(Carbamidomethyl); C22(Carbamidomethyl)	T(1): 33.2; Y(2): 33.2; S(3): 33.2; T(5): 0.3; S(7): 0.0; S(9): 0.0; S(23): 0.0
P17096	KLEKEEEEGISQEssEEEQ	2	S14(Phospho); S15(Phospho)	S(11): 0.0; S(14): 100.0; S(15): 100.0
Q8NC44	QALDsEEEEEDVAAK	1	S5(Phospho)	S(5): 100.0
Q9NP61	RKPDYEPVENTDEAQKK	3		
P46821	SLMssPEDLTK	3	S4(Phospho); S5(Phospho)	S(1): 0.0; S(4): 100.0; S(5): 100.0; T(10): 0.0
Q8TDB6	LKEGHETPMDIDsDDSK	1	S13(Phospho)	T(7): 0.0; S(13): 99.9; S(16): 0.1
O95218	EVEDKEsEGEEEDEDEDLSK	2	S7(Phospho)	S(7): 100.0; S(19): 0.0
O94804	QVAEQGGDLsPAANR	2	S10(Phospho)	S(10): 100.0
Q9BV36	AEGLEEADTGASGcHSHPEEQPTSIsPSR	5	C14(Carbamidomethyl); S26(Phospho)	T(9): 0.0; S(12): 0.0; S(16): 0.0; T(23): 0.3; S(24): 11.9; S(26): 75.9; S(28): 11.9
Q9Y4E8	GASAATGIPLEsDEDSNDNDNDIENENcMHTN	4	S12(Phospho); C28(Carbamidomethyl)	S(3): 0.0; T(6): 0.0; S(12): 95.5; S(16): 4.5; T(31): 0.0
Q14247	TQtPPVSPAPQPTEER	1	T3(Phospho)	T(1): 0.3; T(3): 99.7; S(7): 0.0; T(13): 0.0
P17812	SGSssPDSEITELKFPSINHD	2	S4(Phospho); S5(Phospho)	S(1): 10.2; S(3): 49.9; S(4): 49.9; S(5): 88.7; S(8): 0.7; T(11): 0.7; S(17): 0.0
Q8N3D4	KGsDALRPPVPQGEDEVPK	1	S3(Phospho)	S(3): 100.0
Q13367	AFyGSEEDEAKGAGSEETAAAAAPSR	2	Y3(Phospho)	Y(3): 88.5; S(5): 11.5; S(15): 0.0; T(18): 0.0; S(25): 0.0
Q9UNZ2	DLIHDQDEDEEEEEGQR	1		
Q9H1E3	NSQEDsEDSEDKDVK	1	S6(Phospho)	S(2): 0.0; S(6): 100.0; S(9): 0.0
Q13435	GFEEEHKDsDDDssDDEQEKKPEAPK	1	S9(Phospho); S13(Phospho); S14(Phospho)	S(9): 100.0; S(13): 100.0; S(14): 100.0
P04075	GILAADEsTGSIAK	1	S8(Phospho)	S(8): 93.3; T(9): 6.7; S(11): 0.0
Q86X53	ARQEEGADAsEEDPTPAGEEDVKDAR	1	S10(Phospho)	S(10): 100.0; T(15): 0.0
P46821	DVMSDETNNEETESPSQEFVNITK	1		
P78559	WPEVSPEDTQSLSLSEESPsKETSLDVSSK	3	S20(Phospho)	S(5): 0.0; T(9): 0.0; S(11): 0.0; S(13): 0.0; S(15): 0.0; S(18): 16.5; S(20): 82.5; T(23): 0.8; S(24): 0.2; S(28): 0.0; S(29): 0.0
Q86UU1	KNsITEISDNEDDLLEYHR	2	S3(Phospho)	S(3): 99.6; T(5): 0.4; S(8): 0.0; Y(17): 0.0
P82094	SVSEINsDDELSGK	1	S7(Phospho)	S(1): 0.0; S(3): 0.0; S(7): 100.0; S(12): 0.0
P54727	QEKPAEKPAETPVATsPTATDSTSGDSSR	15	S16(Phospho)	T(11): 0.0; T(15): 10.0; S(16): 79.7; T(18): 10.0; T(20): 0.2; S(22): 0.0; T(23): 0.0; S(24): 0.0; S(27): 0.0; S(28): 0.0
Q13542	TVAISDAAQLPHDYcTtPGGtLFSTTPGGTR	1	C15(Carbamidomethyl); T17(Phospho); T21(Phospho)	T(1): 0.0; S(5): 0.0; Y(14): 3.0; T(16): 15.1; T(17): 81.3; T(21): 0.7; S(24): 13.2; T(25): 71.0; T(26): 13.2; T(30): 2.6
P27361	IADPEHDHTGFLTEyVATR	2	Y15(Phospho)	T(9): 0.0; T(13): 0.0; Y(15): 100.0; T(18): 0.0
P30622	DGHDQHVLELEAK	1		
Q13017	GGIDNPAITsDQELDDKK	1	S10(Phospho)	T(9): 0.0; S(10): 100.0
Q92597	tASGSSVTsLDGTR	5	T1(Phospho); S9(Phospho)	T(1): 7.7; S(3): 92.3; S(5): 0.1; S(6): 0.1; T(8): 7.7; S(9): 92.3; T(13): 0.0

Q9NTJ3	TESPAtAAETASEELDNR	9	T6(Phospho)	T(1): 0.9; S(3): 8.6; T(6): 90.5; T(10): 0.0; S(12): 0.0
Q13153	AVSETPAVPPVsEDEDDDDDATPPPVIAPRPEHTK	1	S12(Phospho)	S(3): 0.0; T(5): 0.0; S(12): 99.7; T(23): 0.3; T(35): 0.0
Q96B36	RTEARSsDEENGPPsSPDLDR	1	S7(Phospho); S15(Phospho)	T(2): 0.9; S(6): 8.7; S(7): 90.4; S(15): 91.2; S(16): 8.8
Q7Z4Q2	RPQFsPTGDcQAEAAAAANGTGGEEDDGPAAELLEK	2	S5(Phospho); C10(Carbamidomethyl)	S(5): 78.6; T(7): 21.4; T(21): 0.1
Q05682	RGsIGENQVEVMVEEK	2	S3(Phospho)	S(3): 100.0
P16989	NYAGEEEEEGSGsSEGFDPPATDR	2	S13(Phospho)	Y(2): 0.0; S(11): 0.3; S(13): 97.5; S(14): 2.1; T(22): 0.0
Q15836	ADALQAGASQFETsAAK	1	S14(Phospho)	S(9): 0.0; T(13): 1.2; S(14): 98.7
P06396	DSQEEEKTEALTSAK	3		
Q9HB90	MSPNETLFLEsTNK	2	S11(Phospho)	S(2): 0.0; T(6): 0.0; S(11): 93.3; T(12): 6.7
Q15052	sTAALEEDAQILK	2	S1(Phospho)	S(1): 50.0; T(2): 50.0
Q14157	STSAPQMSPGsSDNQSSSPQPAQQK	2	S11(Phospho)	S(1): 0.0; T(2): 0.0; S(3): 0.0; S(8): 33.3; S(11): 33.3; S(12): 33.3; S(16): 0.0; S(17): 0.0; S(18): 0.0
Q13200	DKAPVQPQQsPAAAPGGTDEKPSGK	2	S10(Phospho)	S(10): 100.0; T(18): 0.0; S(23): 0.0
O95674	VAHEPVAPPEDKESESEAKVDGETAsDSESR	5	S26(Phospho)	S(14): 0.0; S(16): 0.0; T(24): 49.8; S(26): 49.8; S(28): 0.3; S(30): 0.1
O94979	DSDQVAQsDGEESPAAEEQLLGEHIKEEK	2	S8(Phospho)	S(2): 0.0; S(8): 100.0; S(13): 0.0
P46821	GPAEsPDEGITTTEGEGEcEQTPEELEPVEK	1	S5(Phospho); C19(Carbamidomethyl)	S(5): 100.0; T(11): 0.0; T(12): 0.0; T(13): 0.0; T(22): 0.0
Q9Y6D5	GQsQLsNPTDDSWK	1	S3(Phospho); S6(Phospho)	S(3): 100.0; S(6): 88.8; T(9): 11.2; S(12): 0.0
Q13459	GsDEENLDSETSASTESLLEER	1	S2(Phospho)	S(2): 100.0; S(9): 0.0; T(11): 0.0; S(12): 0.0; S(14): 0.0; T(15): 0.0; S(17): 0.0
Q13895	MPQDGsDDEDEEWPTLEK	3	S6(Phospho)	S(6): 100.0; T(15): 0.0
Q9UEY8	IEEVLSPEGSPSKsPSK	1	S14(Phospho)	S(6): 0.0; S(10): 0.0; S(12): 0.5; S(14): 99.1; S(16): 0.5
Q8IWS0	TAHNSEADLEESFNEHELEPSsPK	4	S22(Phospho)	T(1): 0.0; S(5): 0.0; S(12): 0.0; S(21): 12.2; S(22): 87.8
P41236	DLHDDDEDEEMLETADGESMNTEESNQGSTPSDQQQNK	1		
Q02952	EVSSLEGsPPPcLGQEEAVcTK	3	S8(Phospho); C12(Carbamidomethyl); C20(Carbamidomethyl)	S(3): 0.0; S(4): 0.1; S(8): 99.8; T(21): 0.0
Q9H1E3	TPSPKEEDEEPEsPPEKK	3	S13(Phospho)	T(1): 0.0; S(3): 0.0; S(13): 100.0
Q9BY44	SDKsPDLAPTPAPQSTPR	3	S4(Phospho)	S(1): 0.6; S(4): 99.4; T(10): 0.0; S(15): 0.0; T(16): 0.0
Q12982	KGsITEYTAAEEK	1	S3(Phospho)	S(3): 100.0; T(5): 0.0; Y(7): 0.0; T(8): 0.0
Q6VMQ6	NKQDDDLNcEPLsPHNITPEPVSK	1	C9(Carbamidomethyl); S13(Phospho)	S(13): 85.1; T(18): 14.8; S(23): 0.0
P29966	GEAAAERPGEAAVAsSPSK	2	S15(Phospho)	S(15): 91.5; S(16): 8.4; S(18): 0.1
Q9UHQ4	SHGKDEEcVLEAENKK	7	C8(Carbamidomethyl)	
Q96K76	STETSDFENIEsPLNER	1	S12(Phospho)	S(1): 0.0; T(2): 0.0; T(4): 0.0; S(5): 0.0; S(12): 100.0
Q3YEC7	ADDFPVRDDPsDVtDEDEGPAEPPPPPK	2	S11(Phospho); T14(Phospho)	S(11): 100.0; T(14): 100.0
P04083	GDRSEDFGVNEDLADSDAR	1		
P13861	RVsVcAETYNPDEEEEDTDPR	2	S3(Phospho); C5(Carbamidomethyl)	S(3): 8.1; T(8): 8.1; Y(9): 83.9; T(18): 0.0
P49023	DGGRsSPGGQDEGGFMAQGK	6	S5(Phospho)	S(5): 94.3; S(6): 5.7
O43318	sIQDLTVTGTEPGQVSSR	1	S1(Phospho)	S(1): 100.0; T(6): 0.0; T(8): 0.0; T(10): 0.0; S(16): 0.0; S(17): 0.0
Q9C0C2	sQEADVQDWEFR	1	S1(Phospho)	S(1): 100.0
Q8NI22	DDDKNNDGYIDYAEFAK	3		
O75382	REDsPGPEVQPMDK	2	S4(Phospho)	S(4): 100.0
P35579	KGAGDGsDEEVDGK	3	S7(Phospho)	S(7): 100.0
Q92733	IAAPELHKGDsDsEEDEPTK	1	S11(Phospho); S13(Phospho)	S(11): 100.0; S(13): 100.0; T(19): 0.0
Q9UN37	KPVKENQSEGKGSDsDSEGDNPEK	1	S15(Phospho)	S(8): 84.7; S(13): 2.1; S(15): 13.1; S(17): 0.1
Q5T1M5	SsLsGDEEDELFK	1	S2(Phospho); S4(Phospho)	S(1): 50.0; S(2): 50.0; S(4): 100.0
P78559	SHWDDSTsDSELEK	6	S8(Phospho)	S(1): 0.0; S(6): 0.3; T(7): 5.4; S(8): 93.9; S(10): 0.3
Q96CV9	KNsAIPSELNEK	1	S3(Phospho)	S(3): 99.9; S(7): 0.1
Q02878	HQEGEIFDTEKEKYEITEQR	7		
Q12996	RPNEDsDEDEEKGAVVPPVHDIYR	1	S6(Phospho)	S(6): 100.0; Y(23): 0.0
Q9UHI6	SYLEGSsDNQLKDSESTPVDDR	1	S7(Phospho)	S(1): 0.0; Y(2): 0.0; S(6): 50.0; S(7): 50.0; S(14): 0.0; S(16): 0.0; T(17): 0.0
O14976	ESESALMEDRDEsEVsDEGGSPISSEGQEPR	2	S13(Phospho); S16(Phospho)	S(2): 0.2; S(4): 0.2; S(13): 99.7; S(16): 100.0; S(21): 0.0; S(24): 0.0; S(25): 0.0
Q09666	DDGVFVQEVTQNsPAAR	2	S13(Phospho)	T(10): 0.0; S(13): 100.0
P0CG48	TITLEVEPSDTIENVK	1		
Q96QD8	SHyADVDPENQNFLLESNLGK	2	Y3(Phospho)	S(1): 50.0; Y(3): 50.0; S(17): 0.0
Q9Y2X7	HGsGADSDYENTQSGDPLLGLEGKR	1	S3(Phospho)	S(3): 99.8; S(7): 0.0; Y(9): 0.2; T(12): 0.0; S(14): 0.0
Q9C0C2	ASRVPssDEEVVEEPQSR	2	S6(Phospho); S7(Phospho)	S(2): 12.4; S(6): 93.8; S(7): 93.8; S(17): 0.0
Q7Z5K2	HKKEEEDEELDLNK	2		
Q66PJ3	sAGEEEDGPVLTDEQK	1	S1(Phospho)	S(1): 100.0; T(12): 0.0
Q7Z460	SRsDIDVNAAASAK	3	S3(Phospho)	S(1): 50.0; S(3): 50.0; S(12): 0.0
O15234	STVTGERQsGDGQESTEPVENK	1	S9(Phospho)	S(1): 0.0; T(2): 0.0; T(4): 0.0; S(9): 100.0; S(15): 0.0; T(16): 0.0
Q66K74	RSAsPHDVDLcLVSPcEFEHR	2	S4(Phospho); C11(Carbamidomethyl); C16(Carbamidomethyl)	S(2): 50.0; S(4): 50.0; S(14): 0.0
P55884	TEPAAEAEAASGPSESPsPPAAEELPGSHAEPPVPAQGEAPGEQAR	7	S18(Phospho)	T(1): 0.0; S(11): 2.2; S(14): 6.6; S(16): 20.9; S(18): 69.6; S(28): 0.7
P20020	IEDsEPHIPLIDDTDAEDDAPTKR	4	S4(Phospho)	S(4): 100.0; T(14): 0.0; T(22): 0.0
O60841	KQSFDDNDsEELEDKDSK	3	S9(Phospho)	S(3): 0.0; S(9): 100.0; S(17): 0.0
Q9ULL5	KQEtAAVcGETDEEAGEsGGEGIFR	1	T4(Phospho); C8(Carbamidomethyl); S18(Phospho)	T(4): 0.0; T(11): 100.0; S(18): 100.0
P28715	NAPAAVDEGSIsPR	1	S12(Phospho)	S(10): 0.0; S(12): 100.0
Q8N1G4	EEGSLsDTEADAVSGQLPDPTTNPSAGK	3	S6(Phospho)	S(4): 49.4; S(6): 49.4; T(8): 1.3; S(14): 0.0; T(21): 0.0; T(22): 0.0; S(25): 0.0
O95684	EKGPTTGEGALDLSDVHsPPKsPEGK	5	S18(Phospho); S22(Phospho)	T(5): 0.0; T(6): 0.0; S(14): 5.3; S(18): 97.3; S(22): 97.3
O60841	NKPGPNIEsGNEDDDASFK	4	S9(Phospho)	S(9): 100.0; S(17): 0.0
O43707	KHEAFESDLAAHQDR	5		
Q9H1B7	KAsPEPPDSAEGALK	2	S3(Phospho)	S(3): 100.0; S(9): 0.0
P05023	DKYEPAAVSEQGDKK	3		
Q09666	ASLGSLEGEAEAEASsPK	7	S16(Phospho)	S(2): 0.0; S(5): 0.0; S(15): 0.8; S(16): 99.2
Q8N573	VVsSTSEEEEAFTEK	4	S3(Phospho)	S(3): 47.8; S(4): 47.8; T(5): 4.0; S(6): 0.4; T(13): 0.0
Q9Y4B5	REGPVGGEsDSEEMFEK	2	S9(Phospho)	S(9): 100.0; S(11): 0.0
P04083	SEDFGVNEDLADSDAR	1		
P78559	DEALEQNIQALEENHQTQEQESLVQEDK	1		
P24534	YGPADVEDTTGSGATDSKDDDDIDLFGsDDEEESEEAKR	11	S28(Phospho)	Y(1): 0.0; T(9): 0.0; T(10): 0.0; S(12): 0.0; T(15): 0.0; S(17): 0.0; S(28): 100.0; S(34): 0.0
O75962	KGAANAsGSSPDAPAK	5	S7(Phospho)	S(7): 98.4; S(9): 0.8; S(10): 0.8
Q12888	NsPEDLGLSLTGDScK	2	S2(Phospho); C15(Carbamidomethyl)	S(2): 100.0; S(9): 0.0; T(11): 0.0; S(14): 0.0
O60524	NPYLLsEEEDDDVDGDVNVEK	1	S6(Phospho)	Y(3): 0.0; S(6): 100.0
Q13283	YQDEVFGGFVTEPQEEsEEEVEEPEER	2	S17(Phospho)	Y(1): 0.0; T(11): 0.0; S(17): 100.0
Q96BY6	ASLAsLDsNPSTNEK	1	S5(Phospho); S8(Phospho)	S(2): 9.8; S(5): 90.2; S(8): 100.0; S(11): 0.0; T(12): 0.0
Q9UKA4	KPEsPYGNLcDAPDsPRPVK	2	S4(Phospho); C10(Carbamidomethyl); S15(Phospho)	S(4): 98.8; Y(6): 1.2; S(15): 100.0
P27816	DMsPLSETEMALGK	5	S3(Phospho)	S(3): 100.0; S(6): 0.0; T(8): 0.0
O15511	KVDVDEYDENKFVDEEDGGDGQAGPDEGEVDScLR	1	C33(Carbamidomethyl)	
P78317	RLPQDHADScVVssDDEELSR	2	C10(Carbamidomethyl); S13(Phospho); S14(Phospho)	S(9): 0.0; S(13): 100.0; S(14): 100.0; S(20): 0.0
Q92597	tASGSsVTsLDGTR	1	T1(Phospho); S6(Phospho); S9(Phospho)	T(1): 49.9; S(3): 49.9; S(5): 0.3; S(6): 99.7; T(8): 50.0; S(9): 50.0; T(13): 0.1
P21980	DEREDITHTYKYPEGSSEER	2		
P35579	QAQQERDELADEIANSSGK	1		
P29692	KPATPAEDDEDDDIDLFGsDNEEEDKEAAQLR	10	S19(Phospho)	T(4): 0.0; S(19): 100.0
O43847	LGADESEEEGRRGsLSNAGDPEIVK	4	S14(Phospho)	S(6): 0.6; S(14): 84.1; S(16): 15.3

Q96AT1	RIQPQPPDEDGDHsDKEDEQPQVVVLK	1	S14(Phospho)	S(14): 100.0
Q8N1G4	KYTLENKEEGSLsDTEADAVSGQLPDPTTNPSAGK	2	S13(Phospho)	Y(2): 81.2; T(3): 18.4; S(11): 0.0; S(13): 0.3; T(15): 0.0; S(21): 0.0; T(28): 0.0; T(29): 0.0; S(32): 0.0
O60238	DHSsQSEEEVVEGEKEVEALKK	5	S4(Phospho)	S(3): 49.6; S(4): 49.6; S(6): 0.7
Q8N8S7	GSTIETEQKEDKGEDSEPVTSK	1		
Q3KQU3	RSsQPsPTAVPASDSPPTKQEVK	4	S3(Phospho); S6(Phospho)	S(2): 1.8; S(3): 98.2; S(6): 87.2; T(8): 11.3; S(13): 1.5; S(15): 0.0; T(18): 0.0
Q92614	NKLEGDsDVDSELEDRVDGVK	2	S7(Phospho)	S(7): 100.0; S(11): 0.0
P52943	AsSVTTFTGEPNTcPR	2	S2(Phospho); C14(Carbamidomethyl)	S(2): 92.5; S(3): 6.9; T(5): 0.6; T(6): 0.0; T(8): 0.0; T(13): 0.0
Q9HCN4	GTLDEEDEEADsDTDDIDHR	2	S12(Phospho)	T(2): 0.0; S(12): 99.9; T(14): 0.1
Q5JSH3	LTQTSsTEQLNVLETETEVLNK	8	S6(Phospho)	T(2): 0.5; T(4): 0.5; S(5): 4.6; S(6): 47.2; T(7): 47.2; T(15): 0.0; T(17): 0.0
P35568	HSsETFSSTPSATR	1	S3(Phospho)	S(2): 1.2; S(3): 98.7; T(5): 0.2; S(7): 0.0; S(8): 0.0; T(9): 0.0; S(11): 0.0; T(13): 0.0
Q09666	GHYEVtGSDDETGKLQGSGVSLASK	5	T6(Phospho)	Y(3): 0.0; T(6): 89.1; S(8): 10.8; T(12): 0.0; S(18): 0.0; S(21): 0.0; S(24): 0.0
P16333	RKPsVPDSASPADDSFVDPGER	1	S4(Phospho)	S(4): 99.8; S(8): 0.2; S(10): 0.0; S(15): 0.0
Q09666	GKGGVtGSPEASISGsKGDLK	2	T6(Phospho); S16(Phospho)	T(6): 1.7; S(8): 98.3; S(12): 0.0; S(14): 88.7; S(16): 11.3
P80723	KTEAPAAPAAQETKsDGAPASDSKPGSSEAAPSSK	1	S15(Phospho)	T(2): 0.0; T(13): 50.0; S(15): 50.0; S(21): 0.0; S(23): 0.0; S(27): 0.0; S(28): 0.0; S(33): 0.0; S(34): 0.0
Q9H1E3	VVDYSQFQEsDDADEDYGR	4	S10(Phospho)	Y(4): 0.0; S(5): 0.0; S(10): 100.0; Y(17): 0.0
Q16637	GTGQsDDSDIWDDTALIK	3	S5(Phospho)	T(2): 11.1; S(5): 88.9; S(8): 0.0; T(14): 0.0
Q13541	NSPVtKtPPRDLPTIPGVTSPSSDEPPMEASQSHLR	2	T5(Phospho); T7(Phospho)	S(2): 19.0; T(5): 84.1; T(7): 96.6; T(14): 0.3; T(19): 0.0; S(20): 0.0; S(22): 0.0; S(23): 0.0; S(31): 0.0; S(33): 0.0
Q99543	NAsAsFQELEDK	1	S3(Phospho); S5(Phospho)	S(3): 100.0; S(5): 100.0
Q04917	DNLTLWTSDQQDEEAGEGN	1		
O95155	SQsMDIDGVScEK	1	S3(Phospho); C11(Carbamidomethyl)	S(1): 50.0; S(3): 50.0; S(10): 0.0
Q5SW79	ERsESLDPDSSMDTTLILK	3	S3(Phospho)	S(3): 50.0; S(5): 50.0; S(10): 0.0; S(11): 0.0; T(14): 0.0; T(15): 0.0
P05386	KEESEEsDDDMGFGLFD	5	S7(Phospho)	S(4): 0.0; S(7): 100.0
Q8IV50	SGsESEEAELSLSLAR	2	S3(Phospho)	S(1): 8.3; S(3): 90.9; S(5): 0.8; S(11): 0.0; S(13): 0.0
P46821	TTRtPEEGGYSYDISEK	1	T4(Phospho)	T(1): 33.3; T(2): 33.3; T(4): 33.3; Y(10): 0.0; S(11): 0.0; Y(12): 0.0; S(15): 0.0
Q7L8J4	GLSDHVsLDGQELGTR	3	S7(Phospho)	S(3): 9.2; S(7): 90.8; T(15): 0.0
Q86W92	DFAARSPsAsITDEDSNV	2	S8(Phospho); S10(Phospho)	S(6): 98.4; S(8): 2.4; S(10): 49.6; T(12): 49.6; S(16): 0.0
Q05682;Q05682	QKQEEESLGQVTDQVEVNAQNSVPDEEAK	1		
O14974	RStQGVTLTDLQEAEK	2	T3(Phospho)	S(2): 0.4; T(3): 99.6; T(7): 0.0; T(9): 0.0
P62328	TETQEKNPLPSKETIEQEK	1		
O60504	LcDDGPQLPTsPR	2	C2(Carbamidomethyl); S11(Phospho)	T(10): 0.5; S(11): 99.5
Q16799	GSIsEDELITAIK	2	S4(Phospho)	S(2): 0.0; S(4): 100.0; T(10): 0.0
Q5H9R7	NTVDLVTTcHIHSsSDDEIDFK	2	C9(Carbamidomethyl); S14(Phospho)	T(2): 0.0; T(7): 1.1; T(8): 1.1; S(13): 9.2; S(14): 79.2; S(15): 9.2
Q13586	KAVAEEDNGSIGEETDSsPGRK	2	S18(Phospho)	S(10): 0.0; T(15): 0.2; S(17): 49.9; S(18): 49.9
P41227	GLAAEDsGGDSKDLSEVSETTESTDVK	3	S7(Phospho)	S(7): 99.6; S(11): 0.4; S(15): 0.0; S(18): 0.0; T(20): 0.0; T(21): 0.0; S(23): 0.0; T(24): 0.0
P46821	TLEVVsPSQSVTGSAGHTPYYQSPTDEK	3	S6(Phospho)	T(1): 0.0; S(6): 90.4; S(8): 3.3; S(10): 3.3; T(12): 0.7; S(14): 0.2; T(18): 0.7; Y(20): 0.7; Y(21): 0.7; S(23): 0.0; T(25): 0.0
P62070	KFQEQEcPPsPEPTRK	2	C7(Carbamidomethyl); S10(Phospho)	S(10): 100.0; T(14): 0.0
Q13439	EENPEsDGEPVVEDGTSVK	1	S6(Phospho)	S(6): 100.0; T(16): 0.0; S(17): 0.0
Q5VZ89	RSsLPLDHGSPAQENPESEK	1	S3(Phospho)	S(2): 3.0; S(3): 96.9; S(10): 0.1; S(18): 0.0
Q9NPQ8	GLMAGGRPEGQYsEDEDTDTDEYKEAK	2	S13(Phospho)	Y(12): 50.0; S(13): 50.0; T(18): 0.0; T(20): 0.0; Y(23): 0.0
Q9BTU6	SSsESYTQSFQSR	3	S3(Phospho)	S(1): 3.1; S(2): 3.1; S(3): 45.3; S(5): 45.3; Y(6): 3.1; T(7): 0.0; S(9): 0.0; S(12): 0.0
Q9BRS8	DKNHDEEPTASIHLNK	1		
Q6PKG0	EGTGQQERESPRPLQLPGAEGPAIsDGEEGGGEPGAGGGAAGAAGAGR	1	S10(Phospho); S25(Phospho)	T(3): 57.7; S(10): 57.7; S(25): 84.6
P78559	DRDLEQTDKAPEQK	2		
Q9Y490	DHFGLEGDEESTMLEDSVsPKK	2	S19(Phospho)	S(11): 0.0; T(12): 0.0; S(17): 10.3; S(19): 89.7
Q6P2E9	DSQDASAEQsDHDDEVASLASASGGFGTK	5	S10(Phospho)	S(2): 0.0; S(6): 10.6; S(10): 89.4; S(18): 0.0; S(21): 0.0; S(23): 0.0; T(28): 0.0
Q05655	sDSASSEPVGIYQGFEK	6	S1(Phospho)	S(1): 88.1; S(3): 10.5; S(5): 1.3; S(6): 0.0; Y(12): 0.0
O60841	KWDGsEEDEDNSK	1	S5(Phospho)	S(5): 100.0; S(12): 0.0
P43490	DVYKEHFQDDVFNEK	2		
Q13442	KSLDsDEsEDEEDDYQQK	1	S5(Phospho); S8(Phospho)	S(2): 0.5; S(5): 99.5; S(8): 100.0; Y(15): 0.0
P46821	SPsDSGYSYETIGK	3	S3(Phospho)	S(1): 8.8; S(3): 90.2; S(5): 1.0; Y(7): 0.0; S(8): 0.0; Y(9): 0.0; T(11): 0.0
Q6P158	DLQEQDADAGsER	1	S11(Phospho)	S(11): 100.0
Q6P4E1	VAENVADKNEEPSsNHIPHGK	2	S14(Phospho)	S(13): 14.3; S(14): 85.7
Q6ZSR9	HySPEDEPSPEAQPIAAYK	3	Y2(Phospho)	Y(2): 91.1; S(3): 8.9; S(9): 0.0; Y(18): 0.0
Q9NPE2	GVAGPGPIGREPDPDsDWEPEER	3	S16(Phospho)	S(16): 100.0
Q6PKG0	EGTGQQERESPRPLQLPGAEGPAIsDGEEGGGEPGAGGGAAGAAGAGR	1	S25(Phospho)	T(3): 23.9; S(10): 67.3; S(25): 8.8
Q8IWE2	IHGVNSGSSEGAQPNTENGVPEITDAATDQGPAEsPPTsPSSASR	2	S35(Phospho); S39(Phospho)	S(6): 0.0; S(8): 0.0; S(9): 0.0; T(16): 0.0; T(24): 0.0; T(28): 0.0; S(35): 100.0; T(38): 84.4; S(39): 14.6; S(41): 0.5; S(42): 0.5; S(44): 0.1
Q9UPN3	RGSDAsDFDLLETQsAcSDTSESSAAGGQGNSR	3	S6(Phospho); S15(Phospho); C17(Carbamidomethyl)	S(3): 50.0; S(6): 50.0; T(13): 0.1; S(15): 0.2; S(18): 16.7; T(20): 79.1; S(21): 3.7; S(23): 0.2; S(24): 0.1; S(32): 0.0
P49023	DGGRSsPGGQDEGGFMAQGk	2	S6(Phospho); C-Term(Oxidation)	S(5): 8.4; S(6): 91.6
Q08357	EGALSRVsDEsLSKVQEAESPVFK	1	S8(Phospho); S11(Phospho)	S(5): 0.3; S(8): 99.7; S(11): 98.3; S(13): 1.7; S(20): 0.0
Q8TDJ6	VESDLLDQEEMVDKPDIGsYER	2	S19(Phospho)	S(3): 0.0; S(19): 86.4; Y(20): 13.6
Q9NYF8	KAEGEPQEEsPLKSK	1	S10(Phospho)	S(10): 99.8; S(14): 0.2
Q6PKG0	NTFTAWsDEESDYEIDDRDVNK	3	S7(Phospho)	T(2): 0.0; T(4): 0.0; S(7): 49.9; S(11): 49.9; Y(13): 0.1
Q92685	SGsAAQAEGLcK	2	S3(Phospho); C11(Carbamidomethyl)	S(1): 6.7; S(3): 93.3
Q16637	RGTGQsDDSDIWDDTALIK	2	S6(Phospho)	T(3): 7.8; S(6): 91.5; S(9): 0.7; T(15): 0.0
P51858	GNAEGssDEEGKLVIDEPAKEK	2	S6(Phospho); S7(Phospho)	S(6): 100.0; S(7): 100.0
P20042	KKDAsDDLDDLNFFNQK	1	S5(Phospho)	S(5): 100.0
P52948	DSENLAsPSEYPENGER	1	S7(Phospho)	S(2): 0.0; S(7): 99.3; S(9): 0.7; Y(11): 0.0
Q641Q2	AGNsDsEEDDANGRVELILEPK	2	S4(Phospho); S6(Phospho)	S(4): 100.0; S(6): 100.0
Q92597	sRsHTSEGAHLDITPNSGAAGNSAGPK	5	S1(Phospho); S3(Phospho)	S(1): 98.4; S(3): 3.2; T(5): 96.8; S(6): 1.6; T(14): 0.0; S(17): 0.0; S(23): 0.0
Q8IWW6	AtTPPNQGRPDsPVYANLQELK	2	T2(Phospho); S12(Phospho)	T(2): 50.0; T(3): 50.0; S(12): 99.6; Y(15): 0.4
Q13136	RSsDGsLSHEEDLAK	1	S3(Phospho); S6(Phospho)	S(2): 11.0; S(3): 11.0; S(6): 96.7; S(8): 81.3
Q09666	GHYEVTGsDDETGK	2	S8(Phospho)	Y(3): 0.0; T(6): 0.0; S(8): 99.3; T(12): 0.7
Q14247	AKTQtPPVSPAPQPtEERLPSSPVyEDAASFK	1	T5(Phospho); T15(Phospho); Y25(Phospho)	T(3): 81.6; T(5): 18.4; S(9): 94.8; T(15): 5.7; S(21): 44.6; S(22): 44.6; Y(25): 10.1; S(30): 0.2
Q92974	EPALPLEPDSGGNTsPGVTANGEAR	1	S15(Phospho)	S(10): 0.0; T(14): 1.4; S(15): 98.6; T(19): 0.0
Q9UEY8	IEEVLSPEGSPsKsPSK	1	S12(Phospho); S14(Phospho)	S(6): 0.0; S(10): 50.0; S(12): 50.0; S(14): 99.3; S(16): 0.7
Q9H1C4	YLEEDNsDEsDAEGEHGDGAEEEAPPAGPRPGPEPAGLGR	1	S7(Phospho); S10(Phospho)	Y(1): 1.8; S(7): 99.1; S(10): 99.1
Q86W92	RRPsDENTIAPSEVQK	1	S4(Phospho)	S(4): 100.0; T(8): 0.0; S(12): 0.0
P78559	ELAPAWEDTsPEQDNR	7	S10(Phospho)	T(9): 0.3; S(10): 99.7
O00499	GNKsPSPPDGSPAATPEIR	4	S4(Phospho)	S(4): 88.6; S(6): 11.3; S(11): 0.0; T(15): 0.0
O94929	RFssGGEEDDFDR	1	S3(Phospho); S4(Phospho)	S(3): 100.0; S(4): 100.0
Q13586	SHsPSSPDPDTPSPVGDSR	2	S3(Phospho)	S(1): 10.2; S(3): 89.8; S(5): 0.0; S(6): 0.0; T(11): 0.0; S(13): 0.0; S(18): 0.0
Q96CV9	tSDSDQQAYLVQR	2	T1(Phospho)	T(1): 49.8; S(2): 49.8; S(4): 0.3; Y(9): 0.0
Q6VY07	TNSsDsERSPDLGHSTQIPR	1	S4(Phospho); S6(Phospho)	T(1): 3.9; S(3): 32.2; S(4): 32.2; S(6): 32.8; S(9): 98.9; S(15): 0.0; T(16): 0.0
P01891	KGGSYSQAASSDsAQGsDVsLTAcKV	1	S13(Phospho); S17(Phospho); S20(Phospho); C24(Carbamidomethyl)	S(4): 0.0; Y(5): 0.0; S(6): 0.0; S(10): 50.1; S(11): 50.1; S(13): 87.7; S(17): 87.8; S(20): 24.1; T(22): 0.2
P46821	IAELEEEQSQGSTTNsDWMK	1	S16(Phospho)	S(9): 10.5; S(12): 37.9; T(13): 10.5; T(14): 3.3; S(16): 37.9
P27816	DMEsPTKLDVTLAK	4	S4(Phospho)	S(4): 100.0; T(6): 0.0; T(11): 0.0
Q15052	KDsIPQVLLPEEEK	2	S3(Phospho)	S(3): 100.0
Q07666	KDDEENYLDLFSHK	1		

Q02952	KDEGEGAAGAGDHKDPSLGAGEAASK	4		
Q13641	DAcRDHMEGYHYR	1	C3(Carbamidomethyl)	
Q6P5R6	YFQISQDEDEsESED	4	S11(Phospho)	Y(1): 0.0; S(5): 0.0; S(11): 100.0; S(13): 0.0
P35579	KGAGDGsDEEVDGKADGAEAKPAE	9	S7(Phospho)	S(7): 100.0
Q13017	HRGsEEDPLLSPVETWK	1	S4(Phospho)	S(4): 100.0; S(11): 0.0; T(15): 0.0
P62258	DNLTLWTSDMQGDGEEQNKEALQDVEDENQ	2		
Q92574	NKsESQcDEDGMTSSLSESLK	1	S3(Phospho); C7(Carbamidomethyl)	S(3): 11.1; S(5): 88.9; T(13): 0.0; S(14): 0.0; S(15): 0.0; S(17): 0.0; S(19): 0.0
Q96C19	LSEIDVSSEGVK	1		
P35613	RKPEDVLDDDDAGsAPLK	2	S14(Phospho)	S(14): 100.0
Q6PJI9	EAEHLSSPWGEsSPEELR	2	S12(Phospho)	S(6): 0.0; S(7): 0.0; S(12): 91.1; S(13): 8.9
P78559	DRWPEVsPEDTQSLSLSEESPSK	3	S7(Phospho)	S(7): 99.9; T(11): 0.1; S(13): 0.0; S(15): 0.0; S(17): 0.0; S(20): 0.0; S(22): 0.0
Q9UJM3	RWsAEVTSSTYSDEDRPPK	1	S3(Phospho)	S(3): 100.0; T(7): 0.0; S(8): 0.0; S(9): 0.0; T(10): 0.0; Y(11): 0.0; S(12): 0.0
Q92882	TLSNAEDYLDDEDsD	4	S14(Phospho)	T(1): 0.0; S(3): 0.0; Y(8): 0.0; S(14): 100.0
Q7Z699	HVsFQDEDEIVR	1	S3(Phospho)	S(3): 100.0
O43707	RDHALLEEQSK	1		
Q8WUY3	NLSLTDFVGDESPsPER	4	S14(Phospho)	S(3): 0.0; T(5): 0.0; S(12): 0.0; S(14): 100.0
Q09666	ASLGSLEGEAEAEASsPKGK	4	S16(Phospho)	S(2): 0.0; S(5): 0.0; S(15): 9.4; S(16): 90.6
Q9BV36	ASsESQIFELNK	2	S3(Phospho)	S(2): 0.5; S(3): 99.0; S(5): 0.5
Q5JSL3	EDSRGsLIPEGATGFPDQGNTGENTR	3	S6(Phospho)	S(3): 49.9; S(6): 49.9; T(13): 0.2; T(21): 0.0; T(25): 0.0
Q9Y490	DHFGLEGDEESTMLEDSVsPK	4	S19(Phospho)	S(11): 0.0; T(12): 0.0; S(17): 8.5; S(19): 91.5
Q6WCQ1	DQPDGSSLsPAQsPSQSQPPAASSLR	2	S9(Phospho); S13(Phospho)	S(6): 0.3; S(7): 1.9; S(9): 97.8; S(13): 86.7; S(15): 11.6; S(17): 1.6; S(23): 0.0; S(24): 0.0
P49368	KGESQTDIEITREEDFTR	1		
P27816	DMsPLsETEMALGK	2	S3(Phospho); S6(Phospho)	S(3): 100.0; S(6): 92.2; T(8): 7.8
O43852	VHHEPQLSDKVHNDAQSFDYDHDAFLGAEEAK	1		
Q06210	VDsTTcLFPVEEK	3	S3(Phospho); C6(Carbamidomethyl)	S(3): 88.8; T(4): 5.6; T(5): 5.6
P17812	SGSsSPDSEITELKFPSINHD	21	S4(Phospho)	S(1): 0.9; S(3): 6.1; S(4): 46.4; S(5): 46.4; S(8): 0.1; T(11): 0.0; S(17): 0.0
O00499	GNKsPsPPDGSPAATPEIR	3	S4(Phospho); S6(Phospho)	S(4): 100.0; S(6): 100.0; S(11): 0.0; T(15): 0.0
Q9Y6G9	DFQEYVEPGEDFPAsPQR	2	S15(Phospho)	Y(5): 0.0; S(15): 100.0
P17181	NLLLSTsEEQIEK	1	S7(Phospho)	S(5): 0.3; T(6): 5.0; S(7): 94.7
Q9Y2K6	AVPIAVADEGEsEsEDDDLKPR	1	S12(Phospho); S14(Phospho)	S(12): 100.0; S(14): 100.0
Q6ICG6	SHsANDSEEFFREDDGGADLHNATNLR	1	S3(Phospho)	S(1): 48.7; S(3): 48.7; S(7): 2.5; T(24): 0.0
Q969E4	SEGEGKPQGEGKPASQAKPESQPR	2		
O43815	FLESAAADFsDEDEDDDVDGR	1	S10(Phospho)	S(4): 0.0; S(10): 100.0
Q09666	GGVTGsPEASISGSK	1	S6(Phospho)	T(4): 0.0; S(6): 98.7; S(10): 1.1; S(12): 0.0; S(14): 0.1
P46108	DKPEEQWWNAEDSEGKR	2		
Q9BX95	RNSLtGEEGQLAR	3	T5(Phospho)	S(3): 4.8; T(5): 95.2
Q92614	STsFRQGPEESGLGDGTGPK	3	S3(Phospho)	S(1): 8.1; T(2): 8.1; S(3): 83.7; S(11): 0.0; T(17): 0.0
Q9UPN3	DAYRPTTDADKIEDEVTR	2		
P78559	ELSSEPQtPPAQK	1	T8(Phospho)	S(3): 0.0; S(4): 0.0; T(8): 100.0
Q92597	SHtSEGAHLDITPNSGAAGNSAGPK	12	T3(Phospho)	S(1): 8.4; T(3): 90.7; S(4): 0.8; T(12): 0.0; S(15): 0.0; S(21): 0.0
Q9UN86	NLEELEEKSTtPPPAEPVSLPQEPPKPR	3	T11(Phospho)	S(9): 0.6; T(10): 15.3; T(11): 84.1; S(19): 0.0
Q14697;Q14697	DAQHYGGWEHR	2		
Q9UKS6	DGTAPPPQSPGSPGTGQDEEWsDEESPR	1	S22(Phospho)	T(3): 0.0; S(9): 0.0; S(12): 0.0; T(15): 0.0; S(22): 100.0; S(26): 0.0
Q66K74	sAsPHDVDLcLVSPcEFEHR	2	S1(Phospho); S3(Phospho); C10(Carbamidomethyl); C15(Carbamidomethyl)	S(1): 100.0; S(3): 100.0; S(13): 0.0
Q02952	KPKEDEVEASEK	2		
Q12846	AIEPQKEEADENYNsVNTR	2	S15(Phospho)	Y(13): 0.1; S(15): 99.9; T(18): 0.0
Q8IVF2	DAHDVSPTSTDTEAQLTVER	1		
Q92625	SPsFASEWDEIEK	1	S3(Phospho)	S(1): 5.6; S(3): 94.4; S(6): 0.0
Q15678	IcTEQSNsPPPIR	2	C2(Carbamidomethyl); S8(Phospho)	T(3): 0.0; S(6): 6.4; S(8): 93.6
P17096	KLEKEEEEGIsQEssEEEQ	2	S11(Phospho); S14(Phospho); S15(Phospho)	S(11): 100.0; S(14): 100.0; S(15): 100.0
Q9C0C2	TEAQDLcRAsPEPPGPESSSR	1	C7(Carbamidomethyl); S10(Phospho)	T(1): 0.0; S(10): 100.0; S(18): 0.0; S(19): 0.0; S(20): 0.0
Q86X27	SAAsREDLVGPEVGASPQSGR	3	S4(Phospho)	S(1): 50.0; S(4): 50.0; S(16): 0.0; S(19): 0.0
P46821	DIKPQLELIEDEEKLK	1		
O14974	STGVSFWTQDSDENEQEQQsDTEEGSNKK	4	S20(Phospho)	S(1): 0.0; T(2): 0.0; S(5): 0.0; T(8): 0.1; S(11): 0.0; S(20): 82.5; T(22): 17.2; S(26): 0.2
P78559	WLAEsPVGLPPEEEDKLTR	4	S5(Phospho)	S(5): 100.0; T(18): 0.0
Q9NYI0	SHSSPsLNPDTsPITAK	1	S6(Phospho); S12(Phospho)	S(1): 47.5; S(3): 47.5; S(4): 5.1; S(6): 0.0; T(11): 1.2; S(12): 98.8; T(15): 0.0
Q92597	sRTAsGSSVTSLDGTR	4	S1(Phospho); S5(Phospho)	S(1): 0.7; T(3): 99.3; S(5): 99.9; S(7): 0.1; S(8): 0.0; T(10): 0.0; S(11): 0.0; T(15): 0.0
Q03001	DAYKPITDADKIEDEVTR	2		
Q16555	GLYDGPVcEVSVtPK	1	C8(Carbamidomethyl); T13(Phospho)	Y(3): 0.0; S(11): 0.6; T(13): 99.4
Q08J23	AGEPNsPDAEEANsPDVTAGcDPAGVHPPR	4	S6(Phospho); S14(Phospho); C21(Carbamidomethyl)	S(6): 100.0; S(14): 100.0; T(18): 0.0
P46821	DYNASASTIsPPSSMEEDKFSR	7	S10(Phospho)	Y(2): 0.0; S(5): 0.1; S(7): 33.0; T(8): 33.0; S(10): 33.0; S(13): 0.5; S(14): 0.5; S(21): 0.0
O00193	sASPDDDLGSSNWEAADLGNEERK	2	S1(Phospho)	S(1): 50.0; S(3): 50.0; S(10): 0.0; S(11): 0.0
O15085	TRNsGIWESPELDR	1	S4(Phospho)	T(1): 5.6; S(4): 94.4; S(9): 0.0
P49023	TSSVSNPQDSVGsPcSR	2	S13(Phospho); C15(Carbamidomethyl)	T(1): 0.0; S(2): 0.0; S(3): 0.0; S(5): 0.0; S(10): 0.0; S(13): 99.4; S(16): 0.6
Q14195	EESREPAPAsPAPAGVEIR	1	S10(Phospho)	S(3): 0.0; S(10): 100.0
P27708	IHRAsDPGLPAEEPK	1	S5(Phospho)	S(5): 100.0
P50991	DIEREDIEFIcK	2	C11(Carbamidomethyl)	
P07737	DSLLQDGEFSMDLR	1		
P46821	KEsKEETPEVTK	2	S3(Phospho)	S(3): 99.9; T(7): 0.1; T(11): 0.0
Q9NVD7	DLAEDLYDGQVLQK	1		
Q32MZ4	RGSGDTsISIDTEASIR	9	S7(Phospho)	S(3): 0.0; T(6): 0.5; S(7): 99.4; S(9): 0.0; T(12): 0.0; S(15): 0.0
Q9UMZ2	SGSLDDsFSDFQELPASSK	5	S7(Phospho)	S(1): 0.0; S(3): 0.0; S(7): 98.7; S(9): 1.3; S(17): 0.0; S(18): 0.0
P23634	NEKGEVEQEKLDK	2		
Q8IWZ8	AVQQHQHGYDsDEEVDSELGTWEHQLR	1	S11(Phospho)	Y(9): 0.0; S(11): 99.7; S(17): 0.0; T(21): 0.3
O43852	MDKEETKDWILPSDYDHAEAEAR	4		
Q9Y5Q9	GKLsAEENPDDSEVPSSSGINSTK	1	S4(Phospho)	S(4): 100.0; S(12): 0.0; S(16): 0.0; S(17): 0.0; S(18): 0.0; S(22): 0.0; T(23): 0.0
P07355	AEDGSVIDYELIDQDAR	2		
O95359	LDNTPAsPPRsPAEPNDIPIAK	3	S7(Phospho); S11(Phospho)	T(4): 0.0; S(7): 100.0; S(11): 100.0
Q9UKA4	SVsPTFLNPSDENLK	4	S3(Phospho)	S(1): 9.4; S(3): 90.6; T(5): 0.0; S(10): 0.0
P49736	GLLYDsDEEDEERPAR	4	S6(Phospho)	Y(4): 0.0; S(6): 100.0
O95477	QScLRPFTEDDAADPNDsDIDPESR	2	C3(Carbamidomethyl); S18(Phospho)	S(2): 0.0; T(8): 0.0; S(18): 97.4; S(24): 2.6
P46821	VQSLEGEKLsPKsDIsPLTPR	2	S10(Phospho); S13(Phospho); S16(Phospho)	S(3): 2.6; S(10): 96.2; S(13): 50.7; S(16): 50.7; T(19): 99.8
Q7L2J0	RNscNVGGGGGGFK	2	S3(Phospho); C4(Carbamidomethyl)	S(3): 100.0
Q86TN4	KPLSLAGDEETEcQSsPK	5	C13(Carbamidomethyl); S16(Phospho)	S(4): 0.0; T(11): 0.0; S(15): 8.0; S(16): 92.0
Q1KMD3	EEDEPEERsGDETPGSEVPGDK	1	S9(Phospho)	S(9): 100.0; T(13): 0.0; S(16): 0.0
Q5T4S7	TGSTsSKEDDYESDAATIVQK	5	S5(Phospho)	T(1): 5.0; S(3): 5.0; T(4): 42.5; S(5): 42.5; S(6): 5.0; Y(11): 0.0; S(13): 0.0; T(17): 0.0
P13798	ALDVSAsDDEIAR	1	S7(Phospho)	S(5): 0.0; S(7): 100.0
P14618	NTGIIcTIGPASR	1	C6(Carbamidomethyl)	

P30622	TASESISNLsEAGsIK	1	S10(Phospho); S14(Phospho)	T(1): 0.0; S(3): 0.0; S(5): 0.0; S(7): 0.4; S(10): 99.6; S(14): 100.0
Q9UK76	NsSEASSGDFLDLK	1	S2(Phospho)	S(2): 99.5; S(3): 0.5; S(6): 0.0; S(7): 0.0
P23246	DKLESEMEDAYHEHQANLLR	3		
P06748	ADKDYHFKVDNDENEHQLSLR	2		
Q86WB0	SQDATFSPGsEQAEKsPGPIVSR	1	S10(Phospho); S16(Phospho)	S(1): 0.0; T(5): 0.0; S(7): 99.8; S(10): 0.2; S(16): 100.0; S(22): 0.0
Q13439	TSsFTEQLDEGTPNR	3	S3(Phospho)	T(1): 4.4; S(2): 47.6; S(3): 47.6; T(5): 0.4; T(12): 0.0
Q07866	SREsLNVDVVK	2	S4(Phospho)	S(1): 0.0; S(4): 100.0
Q99733	EFITGDVEPTDAESEWHsENEEEEK	1	S18(Phospho)	T(4): 0.0; T(10): 0.0; S(14): 0.0; S(18): 100.0
Q09666	EVFSScSSEVVLsGDDEEYQR	3	C6(Carbamidomethyl); S13(Phospho)	S(4): 0.0; S(5): 0.0; S(7): 0.0; S(8): 0.0; S(13): 100.0; Y(19): 0.0
P18669	HGEsAWNLENR	1	S4(Phospho)	S(4): 100.0
P56211	YFDsGDYNMAK	1	S4(Phospho)	Y(1): 0.0; S(4): 100.0; Y(7): 0.0
P46821	VSPSKSPSLsPsPPsPLEK	10	S10(Phospho); S12(Phospho); S15(Phospho)	S(2): 0.2; S(4): 1.2; S(6): 98.5; S(8): 0.3; S(10): 98.6; S(12): 1.2; S(15): 100.0
Q9UK76	RNsSEASSGDFLDLK	5	S3(Phospho)	S(3): 50.0; S(4): 50.0; S(7): 0.0; S(8): 0.0
Q5T1M5	SSLsGDEEDELFK	2	S4(Phospho)	S(1): 7.9; S(2): 7.9; S(4): 84.3
Q92974	LQDSSDPDTGSEEEGSSRLsPPHsPR	6	S20(Phospho); S24(Phospho)	S(4): 0.0; S(5): 0.0; T(9): 0.0; S(11): 0.0; S(16): 0.3; S(17): 5.6; S(20): 97.0; S(24): 97.0
P11137	VDHGAEIITQsPGR	1	S11(Phospho)	T(9): 0.0; S(11): 100.0
Q9UMZ2	ETSFGsSENITMTSLSK	2	S6(Phospho)	T(2): 0.0; S(3): 0.0; S(6): 89.6; S(7): 10.2; T(11): 0.2; T(13): 0.0; S(14): 0.0; S(16): 0.0
Q09472	TEIKEEEDQPSTSATQsSPAPGQSK	2	S17(Phospho)	T(1): 0.0; S(11): 0.0; T(12): 0.0; S(13): 0.0; T(15): 46.2; S(17): 46.2; S(18): 7.4; S(24): 0.2
Q14789	DHANEELDELKR	1		
A1X283	KEsIIKSEGELLER	1	S3(Phospho)	S(3): 100.0; S(7): 0.0
P23588	TGsESSQTGTSTTSSR	2	S3(Phospho)	T(1): 50.0; S(3): 50.0; S(5): 0.0; S(6): 0.0; T(8): 0.0; T(10): 0.0; S(11): 0.0; T(12): 0.0; T(13): 0.0; S(14): 0.0; S(15): 0.0
P11142	STAGDTHLGGEDFDNR	2		
Q9NV70	DAIKENPEFDLHFEK	1		
P55081	KIVEPEVVGEsDsEVEGDAWR	2	S11(Phospho); S13(Phospho)	S(11): 100.0; S(13): 100.0
O43237	DFQDYMEPEEGcQGsPQR	6	C12(Carbamidomethyl); S15(Phospho)	Y(5): 0.0; S(15): 100.0
Q9H4L5	LHsSNPNLSTLDFGEEK	7	S3(Phospho)	S(3): 93.6; S(4): 6.4; S(9): 0.0; T(10): 0.0
Q13200	APVQPQQsPAAAPGGTDEKPSGK	1	S8(Phospho)	S(8): 100.0; T(16): 0.0; S(21): 0.0
Q9H1E3	EKTPSPKEEDEEPEsPPEK	3	S15(Phospho)	T(3): 0.0; S(5): 0.0; S(15): 100.0
P13639	LDSEDKDKEGKPLLK	6		
O60936	ASDPDEAGGPEGSEAVQSGtPEEPEPELEAEASK	1	T20(Phospho)	S(2): 0.0; S(13): 0.0; S(18): 2.6; T(20): 97.4; S(33): 0.0
Q6R327	HIEDTGsTPsIGENDLK	1	S7(Phospho); S10(Phospho)	T(5): 88.4; S(7): 10.5; T(8): 2.8; S(10): 98.4
P08238	IEDVGsDEEDDSGKDK	5	S6(Phospho)	S(6): 100.0; S(12): 0.0
P24534	DDDDIDLFGsDDEEESEEAK	3	S10(Phospho)	S(10): 100.0; S(16): 0.0
Q5VTR2	KALVVPEPEPDSDsNQER	2	S14(Phospho)	S(12): 8.0; S(14): 92.0
P46821	ESsPLYsPTFsDSTSAVK	4	S3(Phospho); S7(Phospho); S11(Phospho)	S(2): 90.1; S(3): 9.9; Y(6): 9.8; S(7): 90.0; T(9): 0.3; S(11): 99.8; S(13): 0.0; T(14): 0.0; S(15): 0.0
P29692	KPAtPAEDDEDDDIDLFGsDNEEEDKEAAQLR	2	T4(Phospho); S19(Phospho)	T(4): 100.0; S(19): 100.0
O43432	GGsSKDLLDNQSQEEQR	2	S3(Phospho)	S(3): 50.0; S(4): 50.0; S(12): 0.0
P10451	KANDEsNEHSDVIDSQELSK	1	S6(Phospho)	S(6): 99.9; S(10): 0.1; S(15): 0.0; S(19): 0.0
Q86YP4	RPPsPDVIVLsDNEQPsSPR	2	S4(Phospho); S11(Phospho); S17(Phospho)	S(4): 100.0; S(11): 99.4; S(17): 15.3; S(18): 85.3
P07099	EDDSIRPFKVETSDEEIHDLHQR	3		
P78559	DFQESsPQKGLEVER	1	S6(Phospho)	S(5): 0.0; S(6): 100.0
P60660	DQGTYEDYVEGLR	1		
Q5T3I0	NDADEKHPEHAEQNIR	1		
Q9NY27	NKHPDEDAVEAEGHEVKR	2		
Q9BPX3	TLHcEGTEINsDDEQESK	1	C4(Carbamidomethyl); S11(Phospho)	T(1): 0.0; T(7): 0.0; S(11): 100.0; S(17): 0.0
Q9UQ35	RGEGDAPFSEPGTTSTQRPssPETATK	1	S20(Phospho); S21(Phospho)	S(9): 0.0; T(13): 0.1; T(14): 0.3; S(15): 0.3; T(16): 0.3; S(20): 99.0; S(21): 99.4; T(24): 0.3; T(26): 0.1
Q00341	VATLNsEEESDPPTYK	1	S6(Phospho)	T(3): 0.1; S(6): 99.9; S(10): 0.1; T(14): 0.0; Y(15): 0.0
Q9UQQ2	GLESTEAEMHIPSALEPSTSSsPR	4	S22(Phospho)	S(4): 0.0; T(5): 0.0; S(13): 0.0; S(18): 0.0; T(19): 0.0; S(20): 0.2; S(21): 1.4; S(22): 98.3
P08240	GTGSGGQLQDLDcsssDDEGAAQNSTKPSATK	1	C13(Carbamidomethyl); S14(Phospho); S15(Phospho); S16(Phospho)	T(2): 0.0; S(4): 0.0; S(14): 100.0; S(15): 100.0; S(16): 100.0; S(25): 0.0; T(26): 0.0; S(29): 0.0; T(31): 0.0
O00203	NFYEsDDDQKEK	1	S5(Phospho)	Y(3): 0.0; S(5): 100.0
Q15293	HLVYESDKNKDEK	13		
Q9BW71	RPPtPcsDPER	3	T4(Phospho); C6(Carbamidomethyl); S7(Phospho)	T(4): 100.0; S(7): 100.0
P20810	DTSQSDKDLDDALDKLsDSLGQR	2	S17(Phospho)	T(2): 0.0; S(3): 0.0; S(5): 0.0; S(17): 100.0; S(19): 0.0
Q92597	TAsGSSVTSLDGTR	1	S3(Phospho)	T(1): 0.4; S(3): 99.6; S(5): 0.0; S(6): 0.0; T(8): 0.0; S(9): 0.0; T(13): 0.0
Q6PKG0	DFQEETVKDYEAGQLYGLEK	1		
Q9BPX3	cQTAEADsEsDHEVPEPESEMK	2	C1(Carbamidomethyl); S8(Phospho); S10(Phospho)	T(3): 0.0; S(8): 100.0; S(10): 100.0; S(19): 0.0
P58546	NGDLDEVKDYVAKGEDVNR	1		
Q09666	GGVTGSPEASISGsKGDLK	1	S14(Phospho)	T(4): 0.0; S(6): 0.0; S(10): 33.3; S(12): 33.3; S(14): 33.3
Q9Y2D5	DALGDSLQVPVsPSSTTSSR	2	S12(Phospho)	S(6): 0.0; S(12): 6.6; S(14): 0.7; S(15): 6.6; T(16): 6.6; T(17): 6.6; S(18): 6.6; S(19): 66.4
P16949	NKESKDPADETEAD	1		
P28799	DVEcGEGHFcHDNQTccR	1	C4(Carbamidomethyl); C10(Carbamidomethyl); C16(Carbamidomethyl); C17(Carbamidomethyl)	
Q9Y608	RGsGDTSSLIDPDTSLSELR	3	S3(Phospho)	S(3): 97.0; T(6): 1.0; S(7): 1.0; S(8): 1.0; T(14): 0.0; S(15): 0.0; S(17): 0.0
P63151	DKRPEGYNLKEEDGR	4		
Q03001	FcGVNETENEDNTNRDsPIFDYSPR	1	C2(Carbamidomethyl); S17(Phospho)	T(7): 0.0; T(13): 0.0; S(17): 99.8; Y(22): 0.2; S(23): 0.0
P14618	DPVQEAWAEDVDLR	1		
P18858	VLGsEGEEEDEALsPAK	1	S4(Phospho); S14(Phospho)	S(4): 100.0; S(14): 100.0
P42677	DLLHPSPEEEKRK	2		
P07355	TDLEKDIISDTSGDFR	1		
P08651	TEMDKsPFNSPSPQDSPR	1	S6(Phospho)	T(1): 0.1; S(6): 99.9; S(10): 0.0; S(12): 0.0; S(16): 0.0
O95218	ENVEYIEREEsDGEYDEFGR	1	S11(Phospho)	Y(5): 1.1; S(11): 98.9; Y(15): 0.0
Q01130	DAEDAMDAMDGAVLDGR	2		
P27824	QKSDAEEDGGTVSQEEEDRKPK	2		
Q9BYX2	RAVSEGcAsEDEVEGEA	1	C7(Carbamidomethyl); S9(Phospho)	S(4): 1.1; S(9): 98.9
Q69YQ0	KGSSGNAsEVSVAcLTER	1	S8(Phospho); C14(Carbamidomethyl)	S(3): 33.3; S(4): 33.3; S(8): 33.3; S(11): 0.0; T(16): 0.0
Q92614	LEGDsDVDsELEDRVDGVK	2	S5(Phospho); S9(Phospho)	S(5): 100.0; S(9): 100.0
P17987	DDKHGSYEDAVHSGALND	4		
Q8TEQ0	KVtNIISFDDEEDEQNSGDVFK	1	T3(Phospho)	T(3): 50.0; S(7): 50.0; S(17): 0.0
Q16204	LDQPVsAPPsPR	3	S6(Phospho); S10(Phospho)	S(6): 100.0; S(10): 100.0
Q86V48	EKPDsDDDLDIASLVTAK	4	S5(Phospho)	S(5): 100.0; S(13): 0.0; T(16): 0.0
O60711	STLQDsDEYSNPAPLPLDQHSR	4	S6(Phospho)	S(1): 0.0; T(2): 0.0; S(6): 100.0; Y(9): 0.0; S(10): 0.0; S(21): 0.0
Q09161	KTsDANETEDHLESLIcK	2	S3(Phospho); C17(Carbamidomethyl)	T(2): 0.7; S(3): 99.3; T(8): 0.0; S(14): 0.0
Q9UKA4	sFSEDVFQSVK	1	S1(Phospho)	S(1): 99.9; S(3): 0.1; S(9): 0.0
Q71U36	TIGGGDDsFNTFFSETGAGK	2	S8(Phospho)	T(1): 0.0; S(8): 49.9; T(11): 49.9; S(14): 0.2; T(16): 0.0
Q9Y2K5	sASTDLGTADVVLGR	2	S1(Phospho)	S(1): 47.7; S(3): 47.7; T(4): 4.6; T(8): 0.0
P27797	IDDPTDSKPEDWDKPEHIPDPDAK	4		
P13639	ETVSEESNVLcLSK	1	C11(Carbamidomethyl)	
Q9NQC3	mEDLDQsPLVsSSDSPPRPQPAFK	4	N-Term(Acetyl); S7(Phospho); S11(Phospho)	S(7): 100.0; S(11): 0.0; S(12): 0.0; S(13): 0.2; S(15): 99.8
P17677	NDDDQKIEQDGIKPEDK	1		

Q8TB72	GKAsPFEEDQNR	1	S4(Phospho)	S(4): 100.0
Q9UII2	HHEEEIVHHK	5		
Q8TD55	cSsLGDLLGEGPR	1	C1(Carbamidomethyl); S3(Phospho)	S(2): 50.0; S(3): 50.0
P31689	HYNGEAYEDDEHHPR	1		
P63267;P60709	SYELPDGQVITIGNER	1		
Q6UN15	DHsPTPSVFNSDEER	3	S3(Phospho)	S(3): 99.4; T(5): 0.6; S(7): 0.0; S(11): 0.0
P35611	WLNSGRGDEASEEGQNGSsPK	1	S19(Phospho)	S(4): 0.0; S(11): 0.0; S(18): 10.8; S(19): 89.2
P05023	DKYEPAAVSEQGDK	1		
Q92614	NKLEGDsDVDsELEDRVDGVK	3	S7(Phospho); S11(Phospho)	S(7): 100.0; S(11): 100.0
Q6PD62	GGEFDEFVNDDtDDDLPISK	1	T12(Phospho)	T(12): 100.0; S(19): 0.0
Q9Y5T5	NINMDNDLEVLTSSPtR	2	T16(Phospho)	T(12): 0.0; S(13): 0.1; S(14): 8.7; T(16): 91.2
P01891	DGEDQTQDTELVETRPAGDGTFQK	1		
P13861	VADAKGDsEsEEDEDLEVPVPSR	4	S8(Phospho); S10(Phospho)	S(8): 100.0; S(10): 100.0; S(22): 0.0
Q969T4	QRSDDESPsTSSGSSDADQRDPAAPEPEEQEER	5	S9(Phospho)	S(3): 2.6; S(7): 2.6; S(9): 66.4; T(10): 12.9; S(11): 12.9; S(12): 2.6; S(14): 0.0; S(15): 0.0
P49768	AAVQELSSsILAGEDPEER	1	S9(Phospho)	S(7): 1.5; S(8): 11.0; S(9): 87.5
O95159	DDDRTPGLHGDcDDDKYR	3	C12(Carbamidomethyl)	
Q8NC51	DELTDLDQSNVTEETPEGEEHHPVADTENKENEVEEVKEEGPK	2		
P46821	DSISAVSSEKVSPsKsPsLSPSPPSPLEK	4	S14(Phospho); S16(Phospho); S18(Phospho)	S(2): 0.0; S(4): 0.0; S(7): 0.0; S(8): 0.0; S(12): 0.3; S(14): 99.7; S(16): 99.7; S(18): 0.5; S(20): 1.8; S(22): 98.0; S(25): 0.0
Q8TCJ2	ENPPVEDssDEDDKR	2	S8(Phospho); S9(Phospho)	S(8): 100.0; S(9): 100.0
Q92538	ADAPDAGAQsDSELPSYHQNDVSLDR	1	S10(Phospho)	S(10): 87.1; S(12): 12.9; S(16): 0.0; Y(17): 0.0; S(23): 0.0
Q92614	NKLEGDsDVDSELEDR	1	S7(Phospho)	S(7): 100.0; S(11): 0.0
P35579	KQELEEIcHDLEAR	2	C8(Carbamidomethyl)	
Q96QK1	NTDKNGEELHGGK	2		
Q92785	VDDDsLGEFPVTNSR	1	S5(Phospho)	S(5): 100.0; T(12): 0.0; S(14): 0.0
Q6GYQ0	HFSQSEEtGNEVFGALNEEQPLPR	3	T8(Phospho)	S(3): 1.9; S(5): 12.5; T(8): 85.6
Q9H0B6	TLSSsSMDLSR	1	S5(Phospho)	T(1): 0.0; S(3): 0.0; S(4): 0.4; S(5): 94.2; S(6): 5.5; S(10): 0.0
O43852	GHDLNEDGLVSWEEYK	1		
Q6PD62	KGGEFDEFVNDDtDDDLPISK	1	T13(Phospho)	T(13): 100.0; S(20): 0.0
Q9ULU8	AGGGRPSsPSPSVVSEK	3	S8(Phospho)	S(7): 46.8; S(8): 46.8; S(10): 5.6; S(12): 0.7; S(15): 0.0
P35251	IIYDsDsESEETLQVK	2	S5(Phospho); S7(Phospho)	Y(3): 0.0; S(5): 99.0; S(7): 91.0; S(9): 9.9; T(12): 0.0
Q9H6Z4	SAGGSSPEGGEDsDREDGNYcPPVKR	1	S13(Phospho); C21(Carbamidomethyl)	S(1): 0.0; S(5): 0.0; S(6): 0.0; S(13): 100.0; Y(20): 0.0
Q69YQ0	RSStSSEPTPTVK	1	T4(Phospho)	S(2): 0.7; S(3): 41.5; T(4): 41.5; S(5): 5.2; S(6): 5.2; T(9): 5.2; T(11): 0.7
P46821	QGsPDQVSPVSEMTSTSLYQDK	3	S3(Phospho)	S(3): 88.0; S(8): 12.0; S(11): 0.0; T(14): 0.0; S(15): 0.0; T(16): 0.0; S(17): 0.0; Y(19): 0.0
P07900	ESEDKPEIEDVGsDEEEKK	3	S13(Phospho)	S(2): 0.0; S(13): 100.0
P16949	AsGQAFELILsPR	2	S2(Phospho); S11(Phospho)	S(2): 100.0; S(11): 100.0
O15240	QNALLFAEEEDGEAGAEDKRsQEETPGHR	1	S21(Phospho)	S(21): 77.1; T(25): 22.9
P09104	DGKYDLDFKsPTDPSR	1	S10(Phospho)	Y(4): 0.0; S(10): 100.0; T(12): 0.0; S(15): 0.0
Q01433	RAsLQASTAAPEAR	2	S3(Phospho)	S(3): 100.0; S(7): 0.0; T(8): 0.0
Q8WUX9	IsDAELEAELEK	3	S2(Phospho)	S(2): 100.0
Q13459	VSPPAPGsAPEtPEDK	2	S8(Phospho); T12(Phospho)	S(2): 0.0; S(8): 100.0; T(12): 100.0
P46821	VSAEAEVAPVSPEVTQEVVEEHcAsPEDK	6	C23(Carbamidomethyl); S25(Phospho)	S(2): 0.0; S(11): 0.0; T(15): 0.0; S(25): 100.0
P26006	SQPsETERLTDDY	3	S4(Phospho)	S(1): 0.4; S(4): 99.6; T(6): 0.0; T(10): 0.0; Y(13): 0.0
P07814	EYIPGQPPLSQSSDSsPTR	2	S16(Phospho)	Y(2): 0.0; S(10): 0.0; S(12): 0.0; S(13): 0.1; S(15): 0.8; S(16): 90.9; T(18): 8.2
Q9H063	LSKsQGGEEEGPLSDK	1	S4(Phospho)	S(2): 0.9; S(4): 99.1; S(14): 0.0
Q15751	DREEGHPEPEDEEEEREHEVMTAGK	1		
P00367	DDGSWEVIEGYR	1		
Q14155	KEsAPQVLLPEEEK	1	S3(Phospho)	S(3): 100.0
O00264	IVRGDQPAASGDsDDDEPPPLPR	1	S13(Phospho)	S(10): 0.0; S(13): 100.0
P46108	DsSTSPGDYVLSVSENSR	7	S2(Phospho)	S(2): 46.8; S(3): 46.8; T(4): 5.7; S(5): 0.7; Y(9): 0.0; S(12): 0.0; S(14): 0.0; S(17): 0.0
P37802	NFSDNQLQEGK	2		
O75351	EGQPSPADEKGNDsDGEGESDDPEK	1	S14(Phospho)	S(5): 0.0; S(14): 100.0; S(20): 0.0
P46821	TPQASTYSYETSDLcYTAEKKsPSEAR	2	C15(Carbamidomethyl); S22(Phospho)	T(1): 0.0; S(5): 0.0; T(6): 0.0; Y(7): 0.0; S(8): 0.0; Y(9): 0.0; T(11): 0.0; S(12): 0.0; Y(16): 0.6; T(17): 0.6; S(22): 83.7; S(24): 15.1
P27824	QKsDAEEDGGTVsQEEEDR	1	S3(Phospho); S13(Phospho)	S(3): 100.0; T(11): 0.0; S(13): 100.0
Q8NE71	KLSVPtsDEEDEVPAPKPR	1	T6(Phospho); S7(Phospho)	S(3): 0.0; T(6): 100.0; S(7): 100.0
Q9H6Z4	SAGGssPEGGEDsDREDGNYcPPVKR	2	S5(Phospho); S6(Phospho); S13(Phospho); C21(Carbamidomethyl)	S(1): 87.2; S(5): 25.6; S(6): 87.2; S(13): 100.0; Y(20): 0.0
P05129	tFcGTPDYIAPEIIAYQPYGK	1	T1(Phospho); C3(Carbamidomethyl)	T(1): 97.3; T(5): 2.3; Y(8): 0.4; Y(16): 0.0; Y(19): 0.0
O60271	SASQSsLDKLDQELK	2	S6(Phospho)	S(1): 0.0; S(3): 0.0; S(5): 6.4; S(6): 93.6
P02765	cDSSPDsAEDVRK	4	C1(Carbamidomethyl); S7(Phospho)	S(3): 0.0; S(4): 0.0; S(7): 100.0
Q12959	NKDQSEQETSDADQHVTSNASDSESSYR	1		
P05455	TKFAsDDEHDEHDENGATGPVK	1	S5(Phospho)	T(1): 0.0; S(5): 100.0; T(18): 0.0
Q9NRY4	TSFSVGsDDELGPIR	1	S7(Phospho)	T(1): 0.0; S(2): 0.0; S(4): 0.0; S(7): 100.0
O75475	KDEEGQKEEDKPR	7		
Q8TEW0	SKsMDLGIADETK	1	S3(Phospho)	S(1): 0.5; S(3): 99.5; T(12): 0.0
Q96TA1	AAPEASsPPAsPLQHLLPGK	5	S7(Phospho); S11(Phospho)	S(6): 0.7; S(7): 99.3; S(11): 100.0
P27824	SDAEEDGGTVsQEEEDRKPK	2	S11(Phospho)	S(1): 0.0; T(9): 50.0; S(11): 50.0
P61026	NIDEHANEDVER	1		
Q92733	QITQEEDDsDEEVAPENFFSLPEK	3	S9(Phospho)	T(3): 1.4; S(9): 98.6; S(20): 0.0
Q13813	WRsLQQLAEER	1	S3(Phospho)	S(3): 100.0
Q99615	HSGASAEVQKEEEKK	3		
Q07157	VQIPVSRPDPEPVsDNEEDsYDEEIHDPR	3	S14(Phospho); S20(Phospho)	S(6): 0.0; S(14): 100.0; S(20): 50.0; Y(21): 50.0
P29590	KAsPEAASTPRDPIDVDLPEEAER	1	S3(Phospho)	S(3): 99.9; S(8): 0.0; T(9): 0.0
Q86W92	RsQSTTFNPDDMSEPEFK	3	S2(Phospho)	S(2): 97.3; S(4): 0.9; T(5): 0.9; T(6): 0.9; S(13): 0.0
Q9NZT2	SQGDEAGGHGEDRPEPLsPK	3	S18(Phospho)	S(1): 0.0; S(18): 100.0
Q8NE71	KLSVPTsDEEDEVPAPKPR	2	S7(Phospho)	S(3): 0.0; T(6): 7.9; S(7): 92.1
Q6P2E9	DSQDAsAEQsDHDDEVASLASASGGFGTK	4	S6(Phospho); S10(Phospho)	S(2): 97.8; S(6): 86.8; S(10): 15.4; S(18): 0.0; S(21): 0.0; S(23): 0.0; T(28): 0.0
Q13501	KIALESEGRPEEQMESDNcsGGDDDWTHLSSK	4	C19(Carbamidomethyl); S20(Phospho)	S(6): 0.0; S(16): 2.7; S(20): 97.3; T(27): 0.0; S(30): 0.0; S(31): 0.0
P35606	DNNQFASASLDR	1		
O75832	DHYEATAMHR	1		
Q08AE8	SKsMDKSDEELQFPK	2	S3(Phospho)	S(1): 0.5; S(3): 99.5; S(7): 0.0
P07355	TNQELQEINR	1		
Q9BX66	RVGEQDSAPTQEKPtSPGK	2	T15(Phospho)	S(7): 0.3; T(10): 33.2; T(15): 33.2; S(16): 33.2
P34932	NKEDQYDHLDAADMTk	2	C-Term(Oxidation)	
Q8N6T3	RSSDsWEVWGSASTNR	2	S5(Phospho)	S(2): 48.2; S(3): 3.6; S(5): 48.2; S(11): 0.0; S(13): 0.0; T(14): 0.0
P08670	DGQVINETsQHHDDLE	2	S9(Phospho)	T(8): 8.5; S(9): 91.5
P80723	DKKAEGAATEEEGTPK	1		
P05388	VEAKEEsEEsDEDMGFGLFD	1	S7(Phospho); S10(Phospho)	S(7): 100.0; S(10): 100.0
O60502	LENEGsDEDIETDVLYSPQMALK	4	S6(Phospho)	S(6): 98.3; T(12): 1.7; Y(16): 0.0; S(17): 0.0
Q15642	APsDsSLGTPSDGRPELR	3	S3(Phospho); S5(Phospho)	S(3): 100.0; S(5): 8.9; S(6): 91.1; T(9): 0.0; S(11): 0.0

P02768	KVPQVSTPTLVEVSR	1		
Q02952	GLAEVQQDGEAEEGATsDGEK	4	S17(Phospho)	T(16): 0.0; S(17): 100.0
Q5JSP0	VTPQEEADsDVGEEPDSENTPQK	2	S9(Phospho)	T(2): 0.0; S(9): 100.0; S(17): 0.0; T(20): 0.0
P46821	VQSLEGEKLsPK	1	S10(Phospho)	S(3): 0.0; S(10): 100.0
Q5JTV8	VNFSEEGEtEEDDQDSSHSSVTTVK	2	T9(Phospho)	S(4): 0.0; T(9): 100.0; S(16): 0.0; S(17): 0.0; S(19): 0.0; S(20): 0.0; T(22): 0.0; T(23): 0.0
Q02952	EKTEAEPTHVNEEKLEHETAVTVSEEVSK	5		
Q9Y520	SVEDVRPHHtDANNQSAcFEAPDQK	1	T10(Phospho); C18(Carbamidomethyl)	S(1): 1.2; T(10): 98.8; S(16): 0.0
A7KAX9	SAKsEESLTSLHAVDGDSK	3	S4(Phospho)	S(1): 0.0; S(4): 0.3; S(7): 97.7; T(9): 2.0; S(10): 0.0; S(18): 0.0
Q96BY7	IAAEEEEENDGHYQEEEEGGAHsLKDVcDLR	1	S23(Phospho); C28(Carbamidomethyl)	Y(13): 1.1; S(23): 98.9
Q9UER7	DGDKsPMSSLQISNEK	2	S5(Phospho)	S(5): 92.4; S(8): 7.6; S(9): 0.0; S(13): 0.0
O43765	SRTPsASNDDQQE	1	S5(Phospho)	S(1): 0.0; T(3): 0.4; S(5): 99.5; S(7): 0.0
P50502	AKSEENTKEEKPDSK	7		
Q14699	GDHAsLENEKPGTGDVcSAPAGR	1	S5(Phospho); C17(Carbamidomethyl)	S(5): 100.0; T(13): 0.0; S(18): 0.0
Q9H1E3	DDSHsAEDsEDEKEDHKNVR	2	S5(Phospho); S9(Phospho)	S(3): 0.1; S(5): 99.9; S(9): 100.0
Q8IXJ6	VQEAQDsDsDSEGGAAGGEADMDFLR	6	S7(Phospho); S9(Phospho)	S(7): 87.5; S(9): 87.5; S(11): 25.0
Q69YQ0	KGSsGNAsEVSVAcLTER	3	S4(Phospho); S8(Phospho); C14(Carbamidomethyl)	S(3): 92.8; S(4): 92.8; S(8): 14.3; S(11): 0.0; T(16): 0.0
P19387	NRDNDPNDYVEQDDILIVK	1		
P27824	DKGDEEEEGEEKLEEK	1		
P34932	NAEQNGPVDGQGDNPGPQAAEQGTDTAVPSDSDK	1		
Q9BTU6	VAAAAGSGPsPPGsPGHDR	4	S10(Phospho); S14(Phospho)	S(7): 0.0; S(10): 100.0; S(14): 100.0
Q02952	TEAEPTHVNEEKLEHETAVTVSEEVSK	1		
Q9H3K6	DLEAEHVEVEDTTLNR	2		
Q96B36	SsDEENGPPsSPDLDR	1	S2(Phospho); S10(Phospho)	S(1): 49.9; S(2): 49.9; S(10): 12.8; S(11): 87.4
O95292	SLSSsLDDTEVKK	3	S5(Phospho)	S(1): 0.0; S(3): 0.0; S(4): 9.0; S(5): 90.8; T(9): 0.1
O00629	NVPHEDIcEDSDIDGDYR	1	C8(Carbamidomethyl)	
P63267;P60709	DSYVGDEAQSK	1		
Q09666	GGVtGSPEASISGsKGDLK	2	T4(Phospho); S14(Phospho)	T(4): 50.0; S(6): 50.0; S(10): 0.3; S(12): 12.3; S(14): 87.4
P23588	YAALsVDGEDENEGEDYAE	2	S5(Phospho)	Y(1): 0.2; S(5): 99.8; Y(17): 0.0
Q86W92	SQsTTFNPDDMSEPEFKR	5	S3(Phospho)	S(1): 6.9; S(3): 79.4; T(4): 6.9; T(5): 6.9; S(12): 0.0
O43719	VFDDEsDEKEDEEYADEKGLEAADK	4	S6(Phospho)	S(6): 0.4; Y(14): 99.6
Q9H1E3	KVVDYSQFQEsDDADEDYGRDSGPPTK	4	S11(Phospho)	Y(5): 0.0; S(6): 0.0; S(11): 100.0; Y(18): 0.0; S(22): 0.0; T(26): 0.0
Q9NVS9	KKNEELEQLYQDQEVPKPK	2		
Q15642	APsDSSLGTPSDGRPELR	2	S3(Phospho)	S(3): 100.0; S(5): 0.0; S(6): 0.0; T(9): 0.0; S(11): 0.0
Q16623	TAKDsDDDDDVAVTVDRDR	2	S5(Phospho)	T(1): 0.0; S(5): 100.0; T(14): 0.0
Q9UKL0	EREEsEDELEEANGNNPIDIEVDQNK	1	S5(Phospho)	S(5): 100.0
P05455	TKFAsDDEHDEHDENGATGPVKR	2	S5(Phospho)	T(1): 0.3; S(5): 99.7; T(18): 0.0
Q4V328	GKEEELQDVRDQLEQAQEER	2		
P63208	NDFTEEEEAQVR	1		
O43399	mDSAGQDINLNsPNK	1	N-Term(Acetyl); S12(Phospho)	S(3): 0.0; S(12): 100.0
P42566	SINKLDsPDPFK	1	S7(Phospho)	S(1): 0.0; S(7): 100.0
Q7Z3C6	LEASYSDsPPGEEDLLVHVAEGSK	1	S8(Phospho)	S(4): 0.6; Y(5): 3.0; S(6): 15.0; S(8): 81.3; S(23): 0.0
Q12872	SGVSsDNEDDDDEEDGNYLHPSLFASK	2	S5(Phospho)	S(1): 2.6; S(4): 14.4; S(5): 83.0; Y(18): 0.0; S(22): 0.0; S(26): 0.0
P23588	SRTGsESSQTGTSTTSSR	3	S5(Phospho)	S(1): 5.0; T(3): 47.5; S(5): 47.5; S(7): 0.1; S(8): 0.0; T(10): 0.0; T(12): 0.0; S(13): 0.0; T(14): 0.0; T(15): 0.0; S(16): 0.0; S(17): 0.0
P27797	KIKDPDASKPEDWDER	5		
Q8IWW6	IHQDsESGDELSSSSTEQIR	2	S5(Phospho)	S(5): 50.0; S(7): 50.0; S(12): 0.0; S(13): 0.0; S(14): 0.0; S(15): 0.0; T(16): 0.0
P46821	ESsPLYsPTFSDSTSAVK	6	S3(Phospho); S7(Phospho)	S(2): 50.0; S(3): 50.0; Y(6): 9.9; S(7): 79.9; T(9): 9.9; S(11): 0.2; S(13): 0.0; T(14): 0.0; S(15): 0.0
P20810	SEDSKKPADDQDPIDALSGDLDScPSTTETSQNTAK	1	C24(Carbamidomethyl)	
O14639	STsQGSINSPVYSR	3	S3(Phospho)	S(1): 33.3; T(2): 33.3; S(3): 33.3; S(6): 0.0; S(9): 0.0; Y(12): 0.0; S(13): 0.0
P27448	RsQTSTADSDLKEDGISSR	4	S2(Phospho)	S(2): 96.3; T(4): 1.7; S(5): 0.3; T(6): 1.7; S(9): 0.0; S(17): 0.0; S(18): 0.0
Q96D46	DSAIPVEsDTDDEGAPR	2	S8(Phospho)	S(2): 0.0; S(8): 50.0; T(10): 50.0
O14639	TLsPTPSAEGYQDVR	2	S3(Phospho)	T(1): 7.7; S(3): 92.3; T(5): 0.1; S(7): 0.0; Y(11): 0.0
Q9NQT8	DKTEDDADREAQLLEMR	2		
P00338	DQLIYNLLKEEQTPQNK	1		
Q14247	LPSsPVYEDAASFK	2	S4(Phospho)	S(3): 7.3; S(4): 92.7; Y(7): 0.0; S(12): 0.0
P13639	DLEEDHAcIPIKK	4	C8(Carbamidomethyl)	
Q9BUW7	LEFQQQLGEAPSDAsP	2	S15(Phospho)	S(12): 0.0; S(15): 100.0
P35611	KQKGsEENLDEAR	2	S5(Phospho)	S(5): 100.0
Q9UDY2	GRsIDQDYER	1	S3(Phospho)	S(3): 100.0; Y(8): 0.0
P35221	SRTsVQTEDDQLIAGQSAR	4	S4(Phospho)	S(1): 0.6; T(3): 49.7; S(4): 49.7; T(7): 0.0; S(17): 0.0
Q2TAA2	DVAEAKPELSLLGDGDH	2		
Q02952	VELPsEEQVSGSQGPSEEKPAPLATEVFDEK	2	S5(Phospho)	S(5): 99.9; S(10): 0.1; S(12): 0.0; S(16): 0.0; T(25): 0.0
Q15052	KsTAALEEDAQILK	1	S2(Phospho)	S(2): 91.1; T(3): 8.9
P51812	NsIQFTDGYEVK	2	S2(Phospho)	S(2): 100.0; T(6): 0.0; Y(9): 0.0
O00461	REPEEHQVEEEHRK	4		
Q96TC7	SQsLPNSLDYTQTSDPGR	4	S3(Phospho)	S(1): 10.4; S(3): 88.2; S(7): 1.3; Y(10): 0.0; T(11): 0.0; T(13): 0.0; S(14): 0.0
O94915	RHDEDEDDSLKDR	2		
Q8IVF2	SHsSSEAYEPR	2	S3(Phospho)	S(1): 0.4; S(3): 99.1; S(4): 0.4; S(5): 0.0; Y(8): 0.0
Q8TCJ2	ENPPVEDsSDEDDKR	2	S8(Phospho)	S(8): 91.3; S(9): 8.7
P20810	KSEDSKKPADDQDPIDALSGDLDScPSTTETSQNTAK	1	C25(Carbamidomethyl)	
Q8NB46	RAEPHTPSSHDAEEDEPLKESR	1		
Q02880	VVEAVNSDsDsEFGIPK	2	S9(Phospho); S11(Phospho)	S(7): 54.6; S(9): 54.6; S(11): 90.8
P62258	DNLTLWTSDMQGDGEEQNK	1		
O94762	YTGEEDGAGGHsPAPPQTEEcLR	1	S12(Phospho); C21(Carbamidomethyl)	Y(1): 0.2; T(2): 0.2; S(12): 99.6; T(18): 0.0
P07437	EVDEQMLNVQNK	1		
P35579	RQAQQERDELADEIANSSGK	2		
O15013	YDTNNNEEEEGEQFDFDsGDEIPEADR	2	S18(Phospho)	Y(1): 0.0; T(3): 0.0; S(18): 100.0
Q8WWM7	EVDGLLTSEPMGsPVSSK	1	S13(Phospho)	T(7): 0.4; S(8): 0.4; S(13): 96.5; S(16): 2.3; S(17): 0.4
Q8WX93	SRDsGDENEPIQER	1	S4(Phospho)	S(1): 0.0; S(4): 100.0
Q96G46	QENcGAQQVPAGPGtSTPPSsPVR	2	C4(Carbamidomethyl); T15(Phospho); S21(Phospho)	T(15): 77.7; S(16): 13.3; T(17): 20.8; S(20): 76.3; S(21): 11.9
Q8N8E2	DSEGDSLGARPGLPYGLsDDESGGGR	2	S18(Phospho)	S(2): 0.0; S(6): 0.0; Y(15): 14.8; S(18): 82.3; S(22): 2.9
Q9BVG9	DAGGPRPEsPVPAGR	1	S9(Phospho)	S(9): 100.0
Q13459	VQEKPDsPGGSTQIQR	2	S7(Phospho)	S(7): 100.0; S(11): 0.0; T(12): 0.0
O94808	LDsSAcLHAVGDK	2	S3(Phospho); C6(Carbamidomethyl)	S(3): 100.0; S(4): 0.0
Q96EI5	EGKPEIEGKPEsEGEPGSETR	1	S12(Phospho)	S(12): 100.0; S(18): 0.0; T(20): 0.0
Q9NZT2	KVEEEGsPGDPDHEASTQGR	1	S7(Phospho)	S(7): 100.0; S(16): 0.0; T(17): 0.0
Q9UNE7	LGAGGGsPEKSPSAQELK	2	S7(Phospho)	S(7): 100.0; S(11): 0.0; S(13): 0.0
Q9Y2U5	AQsYPDNHQEFSDYDNPIFEK	4	S3(Phospho)	S(3): 85.8; Y(4): 14.2; S(12): 0.0; Y(14): 0.0
P46821	TTKSPsDSGYSYETIGK	5	S6(Phospho)	T(1): 0.0; T(2): 0.0; S(4): 50.0; S(6): 50.0; S(8): 0.0; Y(10): 0.0; S(11): 0.0; Y(12): 0.0; T(14): 0.0

P78559	SPFEIIsPPAsPPEMVGQR	5	S7(Phospho); S11(Phospho)	S(1): 0.0; S(7): 100.0; S(11): 100.0
O60271	RSsTLSQLPGDK	1	S3(Phospho)	S(2): 0.5; S(3): 99.0; T(4): 0.5; S(6): 0.0
O60343	tSStcSNESLSVGGTSVTPR	4	T1(Phospho); T4(Phospho); C5(Carbamidomethyl)	T(1): 47.9; S(2): 47.9; S(3): 47.9; T(4): 47.9; S(6): 8.3; S(9): 0.1; S(11): 0.0; T(15): 0.0; S(16): 0.0; T(18): 0.0
Q9BYT3	DNFDMcSSsFTSSK	3	C6(Carbamidomethyl); S9(Phospho)	S(7): 33.2; S(8): 33.2; S(9): 33.2; T(11): 0.3; S(12): 0.0; S(13): 0.0
Q8NHZ8	QKEDVEVVGGsDGEGAIGLSSDPK	1	S11(Phospho)	S(11): 100.0; S(20): 0.0; S(21): 0.0
Q9NYF3	SRsQPcDLDAR	2	S3(Phospho); C6(Carbamidomethyl)	S(1): 0.1; S(3): 99.9
Q9Y5S2	HSTPSNSSNPSGPPsPNSPHR	1	S15(Phospho)	S(2): 0.0; T(3): 0.0; S(5): 0.0; S(7): 0.0; S(8): 0.0; S(11): 0.0; S(15): 99.9; S(18): 0.1
Q9UHD8	HVDSLSQRsPK	2	S9(Phospho)	S(4): 0.0; S(6): 6.5; S(9): 93.4
Q86W56	SPQNDDHsDTDSEENRDNQQFLTTVK	1	S8(Phospho)	S(1): 0.0; S(8): 46.2; T(10): 46.2; S(12): 7.6; T(23): 0.0; T(24): 0.0
P43243	RDsFDDRGPSLNPVLDYDHGSR	1	S3(Phospho)	S(3): 0.1; S(10): 99.9; Y(17): 0.0; S(21): 0.0
Q8IYM9	SsGFAFDPSVNYSK	2	S2(Phospho)	S(1): 6.7; S(2): 93.3; S(9): 0.0; Y(12): 0.0; S(13): 0.0
Q6P1N0	KQNsPVAPTAQPK	1	S4(Phospho)	S(4): 100.0; T(9): 0.0
Q9H6H4	AGGLQDsDTEDEcWsDTEAVPR	3	S7(Phospho); C13(Carbamidomethyl); S15(Phospho)	S(7): 99.2; T(9): 0.8; S(15): 99.2; T(17): 0.8
Q9H4I2	KAEENAsQEEEEAAEDEGGEEDLASELR	1	S7(Phospho)	S(7): 100.0; S(25): 0.0
Q14247	tQTPPVsPAPQPTEER	3	T1(Phospho); S7(Phospho)	T(1): 7.2; T(3): 92.8; S(7): 100.0; T(13): 0.0
Q9NQC3	mEDLDQSPLVSSSDsPPRPQPAFK	3	N-Term(Acetyl); S15(Phospho)	S(7): 0.0; S(11): 0.0; S(12): 0.3; S(13): 12.9; S(15): 86.7
O60238	DHSSQSEEEVVEGEKEVEALK	1		
Q13263	SRsGEGEVSGLMR	3	S3(Phospho)	S(1): 0.1; S(3): 99.9; S(9): 0.0
P01889	DGEDQTQDTELVETRPAGDR	1		
O60826	DRPGDEDWVHR	1		
O60343	HAsAPSHVQPSDSEK	6	S3(Phospho)	S(3): 100.0; S(6): 0.0; S(11): 0.0; S(13): 0.0
Q9BTK6	DLFSLDSEDPSPAsPPLR	1	S14(Phospho)	S(4): 0.0; S(7): 0.0; S(11): 0.2; S(14): 99.8
Q6P158	GLsGEEEDDEPDccNDER	1	S3(Phospho); C13(Carbamidomethyl); C14(Carbamidomethyl)	S(3): 100.0
Q09161	RKtSDANETEDHLESLIcK	6	T3(Phospho); C18(Carbamidomethyl)	T(3): 99.5; S(4): 0.5; T(9): 0.0; S(15): 0.0
Q9Y6M7	ESDKEDGREsPSYDTPSQR	2	S10(Phospho)	S(2): 0.0; S(10): 99.8; S(12): 0.2; Y(13): 0.0; T(15): 0.0; S(17): 0.0
P20042	DASDDLDDLNFFNQK	2		
Q5JSZ5	LKFsDDEEEEEVVK	1	S4(Phospho)	S(4): 100.0
P78559	GEKELSSEPQtPPAQK	1	T11(Phospho)	S(6): 0.0; S(7): 0.0; T(11): 100.0
P46821	HSPTEDEESAKAEADAYIR	1		
P11413	sADSVMAEQVALSR	1	S1(Phospho)	S(1): 88.6; S(4): 11.4; S(13): 0.0
Q9P0K7	ISQDADLKtPTKPK	1	T9(Phospho)	S(2): 0.0; T(9): 99.9; T(11): 0.1
Q00613	GHTDTEGRPPsPPPTSTPEK	4	S11(Phospho)	T(3): 0.0; T(5): 0.0; S(11): 100.0; T(15): 0.0; S(16): 0.0; T(17): 0.0
Q8IYB3	KEKtPELPEPSVK	1	T4(Phospho)	T(4): 100.0; S(11): 0.0
P63241	EDLRLPEGDLGKEIEQK	2		
P46821	SPSLSPSPPsPLEK	1	S10(Phospho)	S(1): 0.0; S(3): 0.0; S(5): 0.8; S(7): 49.6; S(10): 49.6
P15531	NIIHGSDSVESAEK	1		
P53985	ESKEEETsIDVAGKPNEVTK	1	S8(Phospho)	S(2): 0.0; T(7): 0.9; S(8): 99.1; T(19): 0.0
Q5JTD0	KDsLTQAQEQGNLLN	1	S3(Phospho)	S(3): 99.1; T(5): 0.9
P24534	DDDDIDLFGsDDEEESEEAKR	2	S10(Phospho)	S(10): 100.0; S(16): 0.0
P51580	TSLDIEEYsDTEVQK	1	S9(Phospho)	T(1): 0.0; S(2): 0.0; Y(8): 0.8; S(9): 98.4; T(11): 0.8
Q9BUA3	NLDPDPEPPsPDsPTETFAAPAEVR	7	S10(Phospho); S13(Phospho)	S(10): 100.0; S(13): 89.3; T(15): 10.6; T(17): 0.2
P35579	RNAEQYKDQADK	1		
P07900	ESEDKPEIEDVGsDEEEEKKDGDK	2	S13(Phospho)	S(2): 0.0; S(13): 100.0
Q8IZP0	HNsTTSSTSSGGYR	2	S3(Phospho)	S(3): 98.6; T(4): 1.2; T(5): 0.2; S(6): 0.0; S(7): 0.0; T(8): 0.0; S(9): 0.0; S(10): 0.0; Y(13): 0.0
Q8TEA8	SASsGAEGDVSSEREP	3	S4(Phospho)	S(1): 8.6; S(3): 8.6; S(4): 82.8; S(11): 0.0; S(12): 0.0
P78559	SPWASDFKDFQESsPQK	3	S14(Phospho)	S(1): 0.0; S(5): 0.0; S(13): 9.5; S(14): 90.5
P06733;P09104	AAVPSGASTGIYEALELR	4		
O60841	VEMYsGsDDDDDFNKLPK	5	S5(Phospho); S7(Phospho)	Y(4): 1.1; S(5): 99.0; S(7): 99.9
Q96NB2	DRNENEIGHSR	1		
P14618	LDIDsPPITAR	1	S5(Phospho)	S(5): 94.7; T(9): 5.3
P19634	SKETSSPGTDDVFTPAPSDsPSSQR	3	S20(Phospho)	S(1): 0.0; T(4): 0.0; S(5): 0.0; S(6): 0.0; T(9): 0.0; T(14): 0.0; S(18): 8.9; S(20): 41.1; S(22): 41.1; S(23): 8.9
P07900	DKEVsDDEAEEK	1	S5(Phospho)	S(5): 100.0
P21980	DEREDITHTYK	2		
Q8TCJ2	ENPPVEDssDEDDKRNQGNLYDK	1	S8(Phospho); S9(Phospho)	S(8): 100.0; S(9): 100.0; Y(21): 0.0
P27361	IADPEHDHTGFLtEYVAtR	3	T13(Phospho); T18(Phospho)	T(9): 0.0; T(13): 100.0; Y(15): 100.0; T(18): 0.0
P11047	STGHGGHcTNcQDNTDGAHcER	2	C8(Carbamidomethyl); C11(Carbamidomethyl); C20(Carbamidomethyl)	
P04083	DITSDTSGDFR	1		
Q9UJU6	NRNEQESAVHPR	2		
Q14671	RDsLTGSSDLYKR	1	S3(Phospho)	S(3): 92.4; T(5): 7.6; S(7): 0.0; S(8): 0.0; Y(11): 0.0
P46821	ESsPLYSPTFSDSTSAVK	4	S3(Phospho)	S(2): 49.9; S(3): 49.9; Y(6): 0.1; S(7): 0.0; T(9): 0.0; S(11): 0.0; S(13): 0.0; T(14): 0.0; S(15): 0.0
Q96HS1	HSQYHVDGSLEKDR	3		
P53985	KESKEEEtSIDVAGKPNEVTK	7	T8(Phospho)	S(3): 0.9; T(8): 90.4; S(9): 8.7; T(20): 0.0
Q86UP2	EIQNGNLHEsDSESVPR	2	S10(Phospho)	S(10): 99.9; S(12): 0.1; S(14): 0.0
Q4G0J3	SRPTSEGsDIESTEPQK	2	S8(Phospho)	S(1): 0.0; T(4): 1.1; S(5): 9.4; S(8): 89.5; S(12): 0.0; T(13): 0.0
P46821	HMDPPPAPVQDRsPsPR	6	S13(Phospho); S15(Phospho)	S(13): 100.0; S(15): 100.0
P48307	DEGLcsANVTR	1	C5(Carbamidomethyl); S6(Phospho)	S(6): 100.0; T(10): 0.0
P46821	TTsPPEVSGYSYEK	2	S3(Phospho)	T(1): 0.5; T(2): 0.5; S(3): 99.0; S(8): 0.0; Y(10): 0.0; S(11): 0.0; Y(12): 0.0
P27708	IHRAsDPGLPAEEPKEK	4	S5(Phospho)	S(5): 100.0
O00469	SEDYVDIVQGNR	1		
O43765	EMPQDLRsPARTPPsEEDSAEAER	1	S8(Phospho); S15(Phospho)	S(8): 3.7; T(12): 96.3; S(15): 99.9; S(19): 0.0
P14618	GDLGIEIPAEK	1		
Q641Q2	TPsDDEEDNLFAPPK	1	S3(Phospho)	T(1): 10.5; S(3): 89.5
Q86UE4	HDGKEVDEGAWETK	1		
Q8TC07	KDsSSVVEWTQAPK	3	S3(Phospho)	S(3): 94.0; S(4): 5.9; S(5): 0.0; T(10): 0.0
Q9BTC0	QEAIPDLEDsPPVsDSEEQQESAR	1	S10(Phospho); S14(Phospho)	S(10): 100.0; S(14): 100.0; S(16): 0.0; S(22): 0.0
P62191	NQEQMKPLEEKQEEER	1		
P50548	RWsEDcRLEGGGGPAGGFEDEGEDK	1	S3(Phospho); C6(Carbamidomethyl)	S(3): 100.0
P01891	KGGSYSQAASSDSAQGsDVSLTAcK	1	S17(Phospho); C24(Carbamidomethyl)	S(4): 0.0; Y(5): 0.0; S(6): 0.0; S(10): 0.0; S(11): 0.0; S(13): 0.0; S(17): 96.0; S(20): 4.0; T(22): 0.1
Q8N1F8	RAsISEPSDTDPEPR	3	S3(Phospho)	S(3): 99.1; S(5): 0.9; S(8): 0.0; T(10): 0.0
Q71UM5	DLLHPSLEEEKKK	2		
O94804	ASQSRPNSsALETLGGEK	3	S9(Phospho)	S(2): 0.0; S(4): 0.0; S(8): 10.7; S(9): 89.2; T(13): 0.0
Q9BX95	NsLTGEEGQLAR	1	S2(Phospho)	S(2): 100.0; T(4): 0.0
P78559	GLDsGAETEEEKDTWEEKK	1	S4(Phospho)	S(4): 100.0; T(8): 0.0; T(14): 0.0
Q9P035	WLDEsDAEMELr	2	S5(Phospho); C-Term(Oxidation)	S(5): 100.0
Q5VW32	DDSTKPKPEEEVKPVK	1		
P06753	GTEDELDKYSEALKDAQEK	1		
P04406	GALQNIIPASTGAAK	1		
Q86VP6	DSIKLDDDSERK	1		

P13667	DLGLSESGEDVNAAILDEsGKK	1	S19(Phospho)	S(5): 0.0; S(7): 0.2; S(19): 99.8
Q96FW1	QEPLGsDSEGVNcLAYDEAIMAQQDR	3	S6(Phospho); C13(Carbamidomethyl)	S(6): 85.3; S(8): 14.7; Y(16): 0.0
O43493	DGSNKsGAEEQGPIDGPSK	2	S6(Phospho)	S(3): 50.0; S(6): 50.0; S(18): 0.0
Q15464	HGsPEFcGILGER	1	S3(Phospho); C7(Carbamidomethyl)	S(3): 100.0
Q9Y490	SKDHFGLEGDEESTMLEDSVsPKK	2	S21(Phospho)	S(1): 0.0; S(13): 0.0; T(14): 0.0; S(19): 12.4; S(21): 87.6
Q5SW79	LKGNKHDDGTQsDSENAGAHR	2	S12(Phospho)	T(10): 44.8; S(12): 44.8; S(14): 10.3
Q96N67	SNsWVNTGGPK	1	S3(Phospho)	S(1): 1.0; S(3): 99.0; T(7): 0.0
Q16512	LNLGTDSDSsPQK	2	S10(Phospho)	T(5): 0.0; S(7): 0.0; S(9): 6.4; S(10): 93.6
Q05655	AsRRsDSASSEPVGIYQGFEK	1	S2(Phospho); S5(Phospho)	S(2): 98.7; S(5): 89.5; S(7): 11.9; S(9): 0.0; S(10): 0.0; Y(16): 0.0
P67809;P16989	SVGDGETVEFDVVEGEK	1		
Q9UKY7	AVTKDEDEWKELEQK	1		
P11274	HQDGLPYIDDsPSSSPHLSSK	1	S11(Phospho)	Y(7): 0.1; S(11): 30.7; S(13): 30.7; S(14): 30.7; S(15): 6.1; S(19): 1.3; S(20): 0.3
P16070	sQEMVHLVNK	1	S1(Phospho)	S(1): 100.0
P46821	TTRtPDTSTYcYETAEK	3	T4(Phospho); C11(Carbamidomethyl)	T(1): 33.3; T(2): 33.3; T(4): 33.3; T(7): 0.0; S(8): 0.0; T(9): 0.0; Y(10): 0.0; Y(12): 0.0; T(14): 0.0
Q7Z4V5	GESAEDKEHEEGRDsEEGPR	2	S15(Phospho)	S(3): 0.0; S(15): 100.0
Q8WZ73	VPAEDETQsIDsEDSFVPGR	1	S9(Phospho); S12(Phospho)	T(7): 0.3; S(9): 99.7; S(12): 98.1; S(15): 1.9
Q9UBF8	sVENLPEcGITHEQR	2	S1(Phospho); C8(Carbamidomethyl)	S(1): 100.0; T(11): 0.0
Q9UM82	sVDAYDSYWESR	1	S1(Phospho)	S(1): 100.0; Y(5): 0.0; S(7): 0.0; Y(8): 0.0; S(11): 0.0
P15056	RDSsDDWEIPDGQITVGQR	7	S4(Phospho)	S(3): 8.4; S(4): 91.6; T(15): 0.0
Q9UII2	KHHEEEIVHHK	1		
P35579	DLEAHIDSANK	3		
P46821	HsPTEDEESAKAEADAYIR	5	S2(Phospho)	S(2): 98.8; T(4): 1.2; S(9): 0.0; Y(17): 0.0
Q13442	KSLDsDEsEDEEDDYQQKR	2	S5(Phospho); S8(Phospho)	S(2): 0.1; S(5): 99.9; S(8): 100.0; Y(15): 0.0
O14828	DGGNPFAEPSELDNPFQDPAVIQHRPsR	3	S27(Phospho)	S(10): 0.0; S(27): 100.0
O43852	VHNDAQsFDYDHDAFLGAEEAK	4	S7(Phospho)	S(7): 100.0; Y(10): 0.0
O75717	NQVEEDAEDsGEADDEEKPEIHKPGQNSFSK	1	S10(Phospho)	S(10): 100.0; S(28): 0.0; S(30): 0.0
P11142;P48741	VEIIANDQGNR	2		
P46821	QsPDHPTVGAGVLHITENGPTEVDYSPSDMQDSSLSHk	3	S2(Phospho); C-Term(Oxidation)	S(2): 49.9; T(7): 49.9; T(16): 0.1; T(21): 0.0; Y(25): 0.0; S(26): 0.0; S(28): 0.0; S(33): 0.0; S(34): 0.0; S(36): 0.0
Q69YU3	RHsTEGPEDPPPWAEK	3	S3(Phospho)	S(3): 94.3; T(4): 5.7
Q02952	RGGGDEESGEHTQVPADSPDSQEEQKGESSASSPEEPEEITcLEK	1	C42(Carbamidomethyl)	
P51798	VGHMSsVELDDELLDPDMDPPHPFPK	2	S6(Phospho)	S(5): 50.0; S(6): 50.0
P35998	TKEDEKDDKPIR	3		
Q02952	SATLSsTESTASEMQEEMK	2	S6(Phospho)	S(1): 0.1; T(3): 0.1; S(5): 8.3; S(6): 83.2; T(7): 8.3; S(9): 0.1; T(10): 0.0; S(12): 0.0
Q7Z5L9	KPsPEPEGEVGPPK	1	S3(Phospho)	S(3): 100.0
Q14C86	SRsSDIVSSVR	1	S3(Phospho)	S(1): 0.0; S(3): 99.9; S(4): 0.0; S(8): 0.0; S(9): 0.0
Q96B23	RDsSESQLASTESDKPTTGR	1	S3(Phospho)	S(3): 78.4; S(4): 10.8; S(6): 10.8; S(10): 0.0; T(11): 0.0; S(13): 0.0; T(17): 0.0; T(18): 0.0
P17987	DDKHGsYEDAVHSGALND	1	S6(Phospho)	S(6): 90.3; Y(7): 9.7; S(13): 0.0
Q13464	DRGHDSEMIGDLQAR	2		
P21283	DFQYNEEEMKADKEEMNR	1		
P55884	sDSRAQAVSEDAGGNEGR	2	S1(Phospho)	S(1): 50.0; S(3): 50.0; S(9): 0.0
P46821	AEtEEAEEPEEDGEEHVcVSASK	1	T3(Phospho); C18(Carbamidomethyl)	T(3): 100.0; S(20): 0.0; S(22): 0.0
Q9Y2H0	QNsATESADSIEIYVPEAQTR	4	S3(Phospho)	S(3): 42.5; T(5): 7.5; S(7): 42.5; S(10): 7.5; Y(14): 0.1; T(20): 0.0
P07237	NFEDVAFDEKK	2		
P27695	KNDKEAAGEGPALYEDPPDQK	2		
Q08945	EGMNPSYDEYADsDEDQHDAYLER	1	S13(Phospho)	S(6): 0.0; Y(7): 0.0; Y(10): 0.0; S(13): 98.2; Y(21): 1.8
Q02790	AKAEASSGDHPTDTEMKEEQK	1		
O60716	GSLAsLDSLR	1	S5(Phospho)	S(2): 0.0; S(5): 100.0; S(8): 0.0
O14545	LDSQPQETsPELPR	2	S9(Phospho)	S(3): 0.0; T(8): 7.4; S(9): 92.6
Q9Y608	NSASATTPLsGNSSR	1	S10(Phospho)	S(2): 0.0; S(4): 0.0; T(6): 0.0; T(7): 0.3; S(10): 78.0; S(13): 10.9; S(14): 10.9
Q53GL0	cAsLEEILSQR	1	C1(Carbamidomethyl); S3(Phospho)	S(3): 100.0; S(9): 0.0
Q5JSZ5	LKFsDDEEEEEVVKDGRPK	1	S4(Phospho)	S(4): 100.0
Q8IYB7	RPGTQGHLGPEKEEEEsDGEPEDSSTS	3	S17(Phospho)	T(4): 0.0; S(17): 100.0; S(24): 0.0; S(25): 0.0; T(26): 0.0; S(27): 0.0
Q05682	RGsIGENQGEEK	1	S3(Phospho)	S(3): 100.0
Q9Y5L0	DLIHTGVANDHEEDFELRK	2		
Q16799	HQEQHHPELEDKDLDFK	4		
P08651	TEMDKSPFNsPsPQDSPR	1	S10(Phospho); S12(Phospho)	T(1): 0.0; S(6): 99.9; S(10): 0.1; S(12): 7.8; S(16): 92.2
Q9H4A3	KEKPELSEPSHLNGPSsDPEAAFLSR	2	S17(Phospho)	S(7): 0.1; S(10): 0.3; S(16): 49.8; S(17): 49.8; S(25): 0.0
Q9P1Y6	GAVAAEGASDtEREEPTESQGLAAR	2	T11(Phospho)	S(9): 1.2; T(11): 98.8; T(17): 0.0; S(19): 0.0
Q13131	SIDDEITEAKSGtATPQR	3	T13(Phospho)	S(1): 0.0; T(7): 0.1; S(11): 90.7; T(13): 9.1; T(15): 0.0
O00264	EGEEPTVYsDEEEPKDESARK	4	S9(Phospho)	T(6): 0.1; Y(8): 12.3; S(9): 87.6; S(18): 0.0
Q4G0J3	KRsSSEDAESLAPR	3	S3(Phospho)	S(3): 33.3; S(4): 33.3; S(5): 33.3; S(10): 0.0
O43852	DRVHHEPQLSDK	7		
P07355	DIISDTSGDFR	1		
Q9Y696	NSRPEANEALER	2		
Q96N67	SRSLsNSNPDISGTPTSPDDEVR	2	S5(Phospho)	S(1): 1.1; S(3): 9.2; S(5): 9.2; S(7): 80.6; S(12): 0.0; T(14): 0.0; T(16): 0.0; S(17): 0.0
Q9H4G0	APEsDTGDEDQDQERDTVFLK	1	S4(Phospho)	S(4): 99.6; T(6): 0.4; T(17): 0.0
Q9NRY4	TSFsVGsDDELGPIR	5	S4(Phospho); S7(Phospho)	T(1): 11.7; S(2): 11.7; S(4): 77.5; S(7): 99.1
Q8WUY3	GAsPDMAPILEPVDR	2	S3(Phospho)	S(3): 100.0
Q14155	KEsAPQVLLPEEEKIIVEETK	2	S3(Phospho)	S(3): 100.0; T(20): 0.0
P27540	FARsDDEQSSADKER	1	S4(Phospho)	S(4): 100.0; S(9): 0.0; S(10): 0.0
O43847	RGSLsNAGDPEIVK	3	S5(Phospho)	S(3): 4.9; S(5): 95.1
Q9C0D5	RADNcsPVAEEETTGSAESTLPK	2	C5(Carbamidomethyl); S6(Phospho)	S(6): 100.0; T(13): 0.0; T(14): 0.0; S(16): 0.0; S(19): 0.0; T(20): 0.0
P05023	DMTSEQLDDILK	1		
Q9NYM9	AQsPGAVEEILDR	1	S3(Phospho)	S(3): 100.0
Q5JTV8	DSHssEEDEASSQTDLSQTISK	1	S4(Phospho); S5(Phospho)	S(2): 17.7; S(4): 91.2; S(5): 91.2; S(11): 0.0; S(12): 0.0; T(14): 0.0; S(17): 0.0; T(19): 0.0; S(21): 0.0
O00264	EGEEPTVYsDEEEPKDESAR	3	S9(Phospho)	T(6): 1.3; Y(8): 10.5; S(9): 88.1; S(18): 0.0
Q5JRA6	DSDYLKNDNPEEHLK	1		
P07737	DSLLQDGEFSMDLr	1	C-Term(Oxidation)	
O75592	SKSDsYTLDPDTLR	3	S5(Phospho)	S(1): 2.1; S(3): 24.5; S(5): 24.5; Y(6): 24.5; T(7): 24.5; T(12): 0.0
P55327	NSPTFKsFEEK	1	S7(Phospho)	S(2): 0.0; T(4): 7.2; S(7): 92.8
P11142	NQTAEKEEFEHQQKELEK	3		
P62857	TGsQGQcTQVR	1	S3(Phospho); C7(Carbamidomethyl)	T(1): 0.5; S(3): 99.5; T(8): 0.0
Q9P227	sAEALGPGALVsPR	1	S1(Phospho); S12(Phospho)	S(1): 100.0; S(12): 100.0
Q9BVS4	EGsEFsFSDGEVAEK	2	S3(Phospho); S6(Phospho)	S(3): 97.8; S(6): 97.8; S(8): 4.4
Q6DD88	DQHSFELDEK	1		
P25205	DGDSYDPYDFsDTEEEMPQVHTPK	2	S11(Phospho)	S(4): 0.0; Y(5): 0.0; Y(8): 0.0; S(11): 100.0; T(13): 0.0; T(22): 0.0
Q9BTU6	VAAAAGSGPsPPGsPGHDRER	4	S10(Phospho); S14(Phospho)	S(7): 0.0; S(10): 100.0; S(14): 100.0
P35269	LDTGPQSLsGKsTPQPPSGK	4	S9(Phospho); S12(Phospho)	T(3): 0.0; S(7): 0.2; S(9): 99.8; S(12): 88.2; T(13): 10.5; S(18): 1.3

P02765	cDSSPDsAEDVR	1	C1(Carbamidomethyl); S7(Phospho)	S(3): 0.0; S(4): 0.0; S(7): 100.0
Q92538	GyTSDSEVYTDHGRPGK	5	Y2(Phospho)	Y(2): 97.9; T(3): 1.1; S(4): 1.1; S(6): 0.0; Y(9): 0.0; T(10): 0.0
Q09666	DIDISsPEFK	2	S6(Phospho)	S(5): 0.0; S(6): 100.0
Q9UNF1	HLDGEEDGsSDQSQASGTTGGR	2	S9(Phospho)	S(9): 50.0; S(10): 50.0; S(13): 0.0; S(16): 0.0; T(18): 0.0; T(19): 0.0
Q9P2W9	VSQsPSKDSEENPATEERPEK	3	S4(Phospho)	S(2): 9.3; S(4): 88.5; S(6): 1.1; S(9): 1.1; T(15): 0.0
P11142	DAGTIAGLNVLR	1		
P07339	DPDAQPGGELMLGGTDSK	1		
Q14103	IDASKNEEDEGHSNsSPR	6	S15(Phospho)	S(4): 0.0; S(13): 0.6; S(15): 92.0; S(16): 7.4
Q07954	cDGDHDcADGSDEKDcTPR	1	C1(Carbamidomethyl); C7(Carbamidomethyl); C16(Carbamidomethyl)	
P46821	QDVDLcLVsScEYK	2	C6(Carbamidomethyl); S9(Phospho); C11(Carbamidomethyl)	S(9): 99.1; S(10): 0.9; Y(13): 0.0
P28482	VADPDHDHTGFLtEYVAtR	6	T13(Phospho); T18(Phospho)	T(9): 0.0; T(13): 100.0; Y(15): 100.0; T(18): 0.0
A6NFI3	GGDAKsPVLQEK	1	S6(Phospho)	S(6): 100.0
Q15527	DLGSTEDGDGtDDFLTDKEDEK	1	T11(Phospho)	S(4): 0.0; T(5): 0.0; T(11): 87.9; T(16): 12.1
P23588	DSDKTDTDWR	1		
P51858	GNAEGSsDEEGKLVIDEPAKEK	2	S7(Phospho)	S(6): 50.0; S(7): 50.0
P04080	VHVGDEDFVHLR	1		
Q14315	VGVTEGcDPTR	1	C7(Carbamidomethyl)	
Q8TC07	NDsPTQIPVSSDVcR	1	S3(Phospho); C14(Carbamidomethyl)	S(3): 99.1; T(5): 0.9; S(10): 0.0; S(11): 0.0
O60493	GDDGIFDDNFIEER	1		
O00299	NSNPALNDNLEK	1		
Q02878	HQEGEIFDTEKEK	1		
Q6PID6	SEAPAEVTHFsPK	1	S11(Phospho)	S(1): 0.0; T(8): 0.1; S(11): 99.9
Q5THJ4	EVQDKDYPLtPPPSPtVDEPK	2	T10(Phospho); T16(Phospho)	Y(7): 0.0; T(10): 100.0; S(14): 50.0; T(16): 50.0
O60841	QSFDDNDsEELEDKDSK	2	S8(Phospho)	S(2): 0.0; S(8): 100.0; S(16): 0.0
O00161	TTWGDGGENsPcNVVSK	1	S10(Phospho); C12(Carbamidomethyl)	T(1): 0.0; T(2): 0.0; S(10): 100.0; S(16): 0.0
Q5JU85	GALSSsLRDLSDAGVcY	3	S6(Phospho); C16(Carbamidomethyl)	S(4): 9.9; S(5): 9.9; S(6): 80.2; S(11): 0.0; Y(17): 0.0
P27816	EAQTLDSQIQETsI	2	S13(Phospho)	T(4): 0.0; S(7): 0.0; T(12): 1.0; S(13): 99.0
P27824	DDTDDEIAKYDGKWEVEEMK	1		
Q8IYB3	KAAsPSPQSVR	1	S4(Phospho)	S(4): 91.1; S(6): 8.8; S(9): 0.1
Q9H8M9	NVFTsAEELER	2	S5(Phospho)	T(4): 4.9; S(5): 95.1
P46940	SKsVKEDSNLTLQEK	1	S3(Phospho)	S(1): 7.2; S(3): 92.8; S(8): 0.0; T(11): 0.0
Q92934	RMsDEFVDSFK	1	S3(Phospho)	S(3): 100.0; S(9): 0.0
Q96CV9	HGARtSDSDQQAYLVQR	2	T5(Phospho)	T(5): 50.0; S(6): 50.0; S(8): 0.0; Y(13): 0.0
Q86TB9	STsPIIGsPPVR	2	S3(Phospho); S8(Phospho)	S(1): 10.9; T(2): 10.9; S(3): 78.3; S(8): 100.0
Q8N3D4	ANEAGGQVGPEAPRPPEtSPEMR	2	T18(Phospho)	T(18): 50.0; S(19): 50.0
P08559	yHGHSMSDPGVSYR	3	Y1(Phospho)	Y(1): 0.0; S(5): 99.8; S(7): 0.2; S(12): 0.0; Y(13): 0.0
O95425	EMEKsFDEQNVPK	2	S5(Phospho)	S(5): 100.0
P19338	GLSEDTTEETLKESFDGSVR	2		
O15173	LLKPGEEPSEYtDEEDTKDHNKQD	4	T12(Phospho)	S(9): 0.0; Y(11): 50.0; T(12): 50.0; T(17): 0.0
Q9UPN3	AFLAELEQNsPK	1	S10(Phospho)	S(10): 100.0
Q9BXJ9	LHDNPLTDENKEHEADTANMSDk	1	C-Term(Oxidation)	
P31943	HTGPNsPDTANDGFVR	1	S6(Phospho)	T(2): 0.0; S(6): 90.5; T(9): 9.5
Q13409	DLEDKEGEIQAGAK	2		
P35611	SPGsPVGEGTGSPPK	1	S4(Phospho)	S(1): 0.1; S(4): 99.9; T(10): 0.0; S(12): 0.0
P07900	ESEDKPEIEDVGsDEEEK	3	S13(Phospho)	S(2): 0.0; S(13): 100.0
O60716	SQsSHSYDDSTLPLIDR	2	S3(Phospho)	S(1): 0.0; S(3): 0.0; S(4): 0.0; S(6): 0.1; Y(7): 99.7; S(10): 0.1; T(11): 0.0
P04792	QLsSGVSEIR	2	S3(Phospho)	S(3): 99.0; S(4): 0.9; S(7): 0.0
Q9BVG4	GADsGEEKEEGINR	1	S4(Phospho)	S(4): 100.0
O43493	DSPSKsSAEAQTPEDTPNK	8	S6(Phospho)	S(2): 0.3; S(4): 33.2; S(6): 33.2; S(7): 33.2; T(12): 0.0; T(16): 0.0
Q8TCU6	DSVLsYTSVR	1	S5(Phospho)	S(2): 0.0; S(5): 48.5; Y(6): 48.5; T(7): 2.9; S(8): 0.2
Q8IYB3	TRHsPTPQQSNR	4	S4(Phospho)	T(1): 0.0; S(4): 99.6; T(6): 0.4; S(10): 0.0
P14618	IYVDDGLISLQVK	1		
P53621	NLsPGAVESDVR	1	S3(Phospho)	S(3): 100.0; S(9): 0.0
P07737	DSPSVWAAVPGK	1		
O60930	KSAsPEVSEGHENQHGQESEAK	4	S4(Phospho)	S(2): 50.0; S(4): 50.0; S(8): 0.0; S(19): 0.0
Q92614	SLAPDRsDDEHDPLDNTSRPR	2	S7(Phospho)	S(1): 9.3; S(7): 90.7; T(17): 0.0; S(18): 0.0
P08238	IEDVGsDEEDDSGKDKK	8	S6(Phospho)	S(6): 100.0; S(12): 0.0
P54727	QEKPAEKPAETPVATSPTATDSTSGDSSR	1		
P27824	QKSDAEEDGGTVsQEEEDRKPK	8	S13(Phospho)	S(3): 0.0; T(11): 50.0; S(13): 50.0
Q9Y2V2	ERsPsPLRGNVVPsPLPTR	3	S3(Phospho); S5(Phospho); S14(Phospho)	S(3): 100.0; S(5): 100.0; S(14): 97.9; T(18): 2.1
Q13813	HQEHKGEIDAHEDSFK	5		
Q3V6T2	SSsQENLLDEVMK	3	S3(Phospho)	S(1): 0.7; S(2): 7.6; S(3): 91.7
P51957	DLFAFQEsPPR	2	S8(Phospho)	S(8): 100.0
Q08379	QQNQEITDQLEEEKKEcHQK	1	C17(Carbamidomethyl)	
Q8IYB3	HRPsPPAtPPPK	6	S4(Phospho); T8(Phospho)	S(4): 100.0; T(8): 100.0
P02794	MGAPESGLAEYLFDKHTLGDSDNEs	3	S25(Phospho)	S(6): 1.2; Y(11): 63.5; T(17): 15.6; S(21): 15.6; S(25): 4.1
P46821	tATcHSSSSPPIDAASAEPYGFR	4	T1(Phospho); C4(Carbamidomethyl)	T(1): 83.1; T(3): 15.7; S(6): 0.7; S(7): 0.2; S(8): 0.2; S(9): 0.2; S(16): 0.0; Y(20): 0.0
P48651	GSEDsPPKHAGNNESHSSR	4	S5(Phospho)	S(2): 1.9; S(5): 98.1; S(15): 0.0; S(17): 0.0; S(18): 0.0
P20810	DKLGERDDTIPPEYR	1		
Q9Y320	AGDNIPEEQPVASTPTTVsDGENKKDK	3	S19(Phospho)	S(13): 0.0; T(14): 0.0; T(16): 2.9; T(17): 0.1; S(19): 97.0
Q12906	RPMEEDGEEKsPSK	3	S11(Phospho)	S(11): 100.0; S(13): 0.0
Q9Y5P4	SSsMSSIDLVSASDDVHR	6	S3(Phospho)	S(1): 0.8; S(2): 0.8; S(3): 98.4; S(5): 0.1; S(6): 0.0; S(11): 0.0; S(13): 0.0
Q9NTJ3	RREEGPPPPSPDGAsSDAEPEPPSGR	8	S15(Phospho)	S(10): 0.0; S(15): 88.4; S(16): 11.6; S(24): 0.0
P14618	TATESFASDPILYRPVAVALDTK	1		
O15439	KDNEESEQPPVPGtPTLR	2	T14(Phospho)	S(6): 0.0; T(14): 90.8; T(16): 9.2
P78559	MLEEKsPEKVK	2	S6(Phospho)	S(6): 100.0
P46821	KIAELEEEQSQGSTTNsDWMk	3	S17(Phospho); C-Term(Oxidation)	S(10): 0.0; S(13): 0.0; T(14): 0.1; T(15): 9.7; S(17): 90.2
Q96JM3	KPSGsPDLWKLsPDQR	2	S5(Phospho); S12(Phospho)	S(3): 50.0; S(5): 50.0; S(12): 100.0
P10809	NAGVEGSLIVEK	1		
O75410	DGHAtDEEKLASTScGQK	1	T5(Phospho); C15(Carbamidomethyl)	T(5): 100.0; S(12): 0.0; T(13): 0.0; S(14): 0.0
Q09666	SKGHYEVTGsDDETGK	1	S10(Phospho)	S(1): 0.0; Y(5): 0.0; T(8): 1.1; S(10): 98.8; T(14): 0.1
Q14699	DKQQAEENEKNLEDQSSK	1		
P53667	ScSIDRsPGAGsLGSPASQR	1	C2(Carbamidomethyl); S7(Phospho); S12(Phospho)	S(1): 0.8; S(3): 8.4; S(7): 90.8; S(12): 0.0; S(15): 99.9; S(18): 0.1
P29323	FLEDDTsDPTYTSALGGK	3	S7(Phospho)	T(6): 0.0; S(7): 1.1; T(10): 89.4; Y(11): 9.4; T(12): 0.1; S(13): 0.0
P46821	SDIsPLTPR	2	S4(Phospho)	S(1): 0.0; S(4): 100.0; T(7): 0.0
Q8TDY2	sTELVLSPDMPR	2	S1(Phospho)	S(1): 94.0; T(2): 6.0; S(7): 0.0
P51812	NQsPVLEPVGR	1	S3(Phospho)	S(3): 100.0
Q14247	TQtPPVSPAPQPtEERLPSSPVYEDAASFK	4	T3(Phospho); T13(Phospho)	T(1): 50.0; T(3): 50.0; S(7): 99.3; T(13): 0.7; S(19): 0.0; S(20): 0.0; Y(23): 0.0; S(28): 0.0

P40222	RPEGPGAQAPSsPR	5	S12(Phospho)	S(11): 0.0; S(12): 100.0
O94979	AQGEPVAGHEsPKIPYEK	3	S11(Phospho)	S(11): 100.0; Y(16): 0.0
P52209	AGQAVDDFIEK	1		
P07355	AEDGsVIDYELIDQDAR	1	S5(Phospho)	S(5): 100.0; Y(9): 0.0
Q05193	RSPtSsPTPQR	2	T4(Phospho); S6(Phospho)	S(2): 1.1; T(4): 97.8; S(5): 9.1; S(6): 83.6; T(8): 8.3
Q15293	DKDDKISWEEYK	1		
Q92614	LEGDsDVDSELEDRVDGVK	1	S5(Phospho)	S(5): 99.9; S(9): 0.1
Q6QNY0	VAGEAAEtDsEPEPEPEPTAAPR	2	T8(Phospho); S10(Phospho)	T(8): 100.0; S(10): 100.0; T(19): 0.0
P00441	KHGGPKDEER	5		
P35269	GNsRPGTPsAEGGSTSSTLR	3	S3(Phospho); S9(Phospho)	S(3): 100.0; T(7): 99.7; S(9): 0.3; S(14): 0.0; T(15): 0.0; S(16): 0.0; S(17): 0.0; T(18): 0.0
Q9UPN3	DcDVQGLEHDMEEINAR	1	C2(Carbamidomethyl)	
P23284	DKPLKDVIIADcGK	1	C12(Carbamidomethyl)	
P46821	VLsPLRsPPLIGSESAYESFLSADDK	1	S3(Phospho); S7(Phospho)	S(3): 100.0; S(7): 100.0; S(13): 0.0; S(15): 0.0; Y(17): 0.0; S(19): 0.0; S(22): 0.0
Q96B36	LNtSDFQK	1	T3(Phospho)	T(3): 99.8; S(4): 0.2
Q8WUM4	KDNDFIYHDR	1		
Q8NC51	SKSEEAHAEDSVMDHHFR	3		
Q9NYF8	AEGEWEDQEALDYFsDKESGK	1	S15(Phospho)	Y(13): 11.1; S(15): 87.4; S(19): 1.5
Q14247	AKtQTPPVsPAPQPTEER	4	T3(Phospho); S9(Phospho)	T(3): 9.6; T(5): 90.4; S(9): 100.0; T(15): 0.0
Q07954	DKSDEKPSYcNSR	2	C10(Carbamidomethyl)	
Q9BXF6	TYsDEANQMR	1	S3(Phospho)	T(1): 0.0; Y(2): 0.3; S(3): 99.7
P78559	NTSAEKELSSPIsPK	4	S13(Phospho)	T(2): 0.0; S(3): 0.0; S(9): 0.0; S(10): 0.0; S(13): 100.0
Q15311	AGKEPAKPsPSR	2	S9(Phospho)	S(9): 50.0; S(11): 50.0
Q03135	AMADELsEKQVYDAHTK	1	S7(Phospho)	S(7): 100.0; Y(12): 0.0; T(16): 0.0
P27797	EQFLDGDGWTSR	1		
P35611	SRSPGsPVGEGTGSPPK	1	S6(Phospho)	S(1): 0.0; S(3): 0.0; S(6): 100.0; T(12): 0.0; S(14): 0.0
Q9BVS4	VQGGVPAGsDEYEDEcPHLIALSSLNR	1	S9(Phospho); C16(Carbamidomethyl)	S(9): 92.9; Y(12): 7.0; S(23): 0.0; S(24): 0.0
O95425	DSSFTEVPRsPK	1	S10(Phospho)	S(2): 0.0; S(3): 0.0; T(5): 0.0; S(10): 100.0
Q02952	KDEGEGAAGAGDHKDPsLGAGEAASK	2	S17(Phospho)	S(17): 100.0; S(25): 0.0
Q9BXJ9	LHDNPLTDENKEHEADTANMSDK	2		
P25789	KHEEEEAKAER	5		
O94808	RLDsSAcLHAVGDK	5	S4(Phospho); C7(Carbamidomethyl)	S(4): 94.4; S(5): 5.6
P06733	KLNVTEQEKIDK	1		
Q8WUM9	KNsLKEDHEETK	3	S3(Phospho)	S(3): 100.0; T(11): 0.0
P29692	ATAPQTQHVsPMR	2	S10(Phospho)	T(2): 0.0; T(6): 0.0; S(10): 100.0
Q96AT1	KKPNEDEVNQDSVKK	1		
Q13428	KLGAGEGGEASVsPEKTSTTSK	1	S13(Phospho)	S(11): 1.6; S(13): 97.7; T(17): 0.2; S(18): 0.2; T(19): 0.2; T(20): 0.0; S(21): 0.0
Q9BQA1	DSVFLScSEDNR	2	C7(Carbamidomethyl)	
P52926	KQQQEPTGEPsPK	3	S11(Phospho)	T(7): 0.0; S(11): 100.0
Q969T4	QRSDDESPStSSGSSDADQR	2	T10(Phospho)	S(3): 9.2; S(7): 1.1; S(9): 9.2; T(10): 79.3; S(11): 1.1; S(12): 0.2; S(14): 0.0; S(15): 0.0
Q9Y5K6	sVDFDSLTVR	1	S1(Phospho)	S(1): 100.0; S(6): 0.0; T(8): 0.0
Q9NRX5	SDGsLEDGDDVHR	1	S4(Phospho)	S(1): 1.0; S(4): 99.0
Q6R327	NDsGEENVPLDLTR	1	S3(Phospho)	S(3): 100.0; T(13): 0.0
Q6VY07	TNSsDSERSPDLGHSTQIPR	4	S4(Phospho)	T(1): 1.0; S(3): 6.3; S(4): 43.2; S(6): 43.2; S(9): 6.3; S(15): 0.0; T(16): 0.0
P51858	AGDLLEDsPKRPK	4	S8(Phospho)	S(8): 100.0
Q92609	RTsSTLDSEGTFNSYR	4	S3(Phospho)	T(2): 94.5; S(3): 5.5; S(4): 0.0; T(5): 0.0; S(8): 0.0; T(11): 0.0; S(14): 0.0; Y(15): 0.0
Q7Z5R6	RSsDTSGSPATPLK	5	S3(Phospho)	S(2): 3.6; S(3): 48.0; T(5): 48.0; S(6): 0.3; S(8): 0.0; T(11): 0.0
Q5VTR2	ALVVPEPEPDSDsNQER	2	S13(Phospho)	S(11): 6.7; S(13): 93.3
Q92934	RMsDEFVDSFKK	1	S3(Phospho)	S(3): 100.0; S(9): 0.0
P78559	AELEEMEEVHPsDEEEEDATK	4	S12(Phospho)	S(12): 99.8; T(20): 0.2
Q53GL0	RADsDRIQPSADR	1	S4(Phospho)	S(4): 100.0; S(10): 0.0
P27824	KPEDWDERPK	1		
Q96ST2	AAVLsDsEDEEKASAK	1	S5(Phospho); S7(Phospho)	S(5): 100.0; S(7): 100.0; S(14): 0.0
Q13424	NSTGGTSVGWDsPPAsPLQR	2	S12(Phospho); S16(Phospho)	S(2): 0.0; T(3): 0.0; T(6): 0.0; S(7): 0.0; S(12): 100.0; S(16): 100.0
Q12906	RPMEEDGEEKsPSKK	2	S11(Phospho)	S(11): 90.1; S(13): 9.9
Q8IWS0	KTAHNSEADLEESFNEHELEPsSPK	2	S22(Phospho)	T(2): 0.0; S(6): 0.0; S(13): 0.3; S(22): 87.4; S(23): 12.3
O43719	VFDDEsDEKEDEEYADEK	2	S6(Phospho)	S(6): 99.8; Y(14): 0.2
Q641Q2	ETVSEAPPLLFsDEEEKEAQLGVK	1	S12(Phospho)	T(2): 0.0; S(4): 0.0; S(12): 100.0
Q04637	NHDEESLEcLcR	1	C9(Carbamidomethyl); C11(Carbamidomethyl)	
P46821	ASVsPMDEPVPDsESPIEK	3	S4(Phospho); S13(Phospho)	S(2): 9.6; S(4): 90.4; S(13): 50.0; S(15): 50.0
Q9NWV8	TRsNPEGAEDR	1	S3(Phospho)	T(1): 0.5; S(3): 99.5
P12694	IGHHsTSDDSSAYR	2	S5(Phospho)	S(5): 33.3; T(6): 33.3; S(7): 33.3; S(10): 0.0; S(11): 0.0; Y(13): 0.0
Q09666	KGDRsPEPGQTWTR	4	S5(Phospho)	S(5): 100.0; T(11): 0.0; T(13): 0.0
Q9NR09	LEGDSDDLLEDsDSEEHSR	3	S12(Phospho)	S(5): 0.0; S(12): 46.8; S(14): 46.8; S(18): 6.4
P67936	EKAEGDVAALNR	1		
Q9H792	SFLGTsGELSVK	2	S6(Phospho)	S(1): 0.0; T(5): 47.6; S(6): 47.6; S(10): 4.8
Q641Q2	RtPSDDEEDNLFAPPK	4	T2(Phospho)	T(2): 93.0; S(4): 7.0
O43318	RMsADMSEIEAR	1	S3(Phospho)	S(3): 99.0; S(7): 1.0
Q9Y478	cSDVSELSSsPPGPYHQEPYVcKPEER	2	C1(Carbamidomethyl); S10(Phospho); C22(Carbamidomethyl)	S(2): 0.0; S(5): 0.0; S(8): 4.7; S(9): 18.3; S(10): 75.7; Y(15): 1.3; Y(20): 0.0
O43399	NSATFKsFEDR	4	S7(Phospho)	S(2): 0.0; T(4): 0.0; S(7): 100.0
Q9UPN3	QGsFSEDVISHK	1	S3(Phospho)	S(3): 99.9; S(5): 0.1; S(10): 0.0
Q8TAQ2	GHREEEQEDLTK	1		
P11142	cNEIINWLDKNQTAEKEEFEHQQK	1	C1(Carbamidomethyl)	
P68104	IGGIGTVPVGR	1		
P21291	HEEAPGHRPTTNPNASK	1		
Q05519	VNGDDHHEEDMDMsD	1	S14(Phospho)	S(14): 100.0
Q13185	RKsLsDSESDDSK	3	S3(Phospho); S5(Phospho)	S(3): 98.7; S(5): 89.7; S(7): 11.4; S(9): 0.2; S(12): 0.0
Q8NE71	QQPPEPEWIGDGESTsPSDK	1	S16(Phospho)	S(14): 0.2; T(15): 11.3; S(16): 88.3; S(18): 0.2
P67936;P06753	HIAEEADRKYEEVAR	4		
O95361	ETEEQDsDSAEQGDPAGEGKEVLcDFcLDDTRR	4	S7(Phospho); C24(Carbamidomethyl); C27(Carbamidomethyl)	T(2): 16.3; S(7): 67.3; S(9): 16.3; T(31): 0.0
Q13442	SLDsDEsEDEEDDYQQKR	2	S4(Phospho); S7(Phospho)	S(1): 50.0; S(4): 50.0; S(7): 100.0; Y(14): 0.0
Q9NWV8	mEVAEPSsPTEEEEEEEEHSAEPRPR	3	N-Term(Acetyl); S8(Phospho)	S(7): 12.2; S(8): 86.0; T(10): 1.8; S(20): 0.0
Q9NWW5	HGsVSADEAAR	1	S3(Phospho)	S(3): 100.0; S(5): 0.0
Q86XR7	RHsVDTSPGYHESDSK	1	S3(Phospho)	S(3): 100.0; T(6): 0.0; S(7): 0.0; Y(10): 0.0; S(13): 0.0; S(15): 0.0
Q09666	ASLGsLEGEAEAEAsSPKGK	2	S5(Phospho); S15(Phospho)	S(2): 14.8; S(5): 85.2; S(15): 50.0; S(16): 50.0
Q5JSH3	SNSGRELtDEEILASVMIK	3	T8(Phospho)	S(1): 1.2; S(3): 1.2; T(8): 97.6; S(15): 0.0
Q14697;Q14697	DGDKPEETQGKAEKDEPGAWEETFK	1		
P18615	sLYESFVSSSDR	2	S1(Phospho)	S(1): 100.0; Y(3): 0.0; S(5): 0.0; S(8): 0.0; S(9): 0.0; S(10): 0.0
Q8NFA0	LSsSKENLDASK	1	S3(Phospho)	S(2): 0.5; S(3): 49.8; S(4): 49.8; S(11): 0.0

P78559	GELsPSFLNPPLPPSIDDRDLSTEEVR	4	S4(Phospho)	S(4): 86.6; S(6): 13.4; S(15): 0.0; S(22): 0.0; T(23): 0.0
Q9H814	DLDKELDEYMHGGK	1		
Q5SW79	DKDRNWDDIESK	1		
Q3KQU3	ESAAPAsPAPsPAPsPTPAPPQK	1	S7(Phospho); S11(Phospho); S15(Phospho)	S(2): 0.0; S(7): 100.0; S(11): 100.0; S(15): 89.5; T(17): 10.5
P26358	EADDDEEVDDNIPEMPsPK	1	S17(Phospho)	S(17): 100.0
Q15154	DQHLNNSSSsPQR	1	S10(Phospho)	S(7): 1.0; S(8): 1.0; S(9): 89.1; S(10): 8.9
P78559	NTSAEKELSsPIsPK	4	S10(Phospho); S13(Phospho)	T(2): 0.0; S(3): 0.0; S(9): 0.6; S(10): 99.4; S(13): 100.0
P0CG48	IQDKEGIPPDQQR	7		
Q5TH69	SGsTGsSLSVSVR	1	S3(Phospho); S6(Phospho)	S(1): 52.7; S(3): 52.7; T(4): 91.1; S(6): 1.7; S(7): 1.7; S(9): 0.0; S(11): 0.0
Q92609	tSSTLDSEGTFNSYR	2	T1(Phospho)	T(1): 49.7; S(2): 49.7; S(3): 0.5; T(4): 0.1; S(7): 0.0; T(10): 0.0; S(13): 0.0; Y(14): 0.0
P22059	MLAEsDEsGDEESVSQTDKTELQNTLR	2	S5(Phospho); S8(Phospho)	S(5): 100.0; S(8): 100.0; S(13): 0.0; S(15): 0.0; T(17): 0.0; T(20): 0.0; T(25): 0.0
Q8NE71	LSVPtsDEEDEVPAPKPR	2	T5(Phospho); S6(Phospho)	S(2): 0.0; T(5): 100.0; S(6): 100.0
Q07157	sREDLSAQPVQTK	3	S1(Phospho)	S(1): 50.0; S(6): 50.0; T(12): 0.0
Q14157	NQDEcVIALHDcNGDVNR	1	C5(Carbamidomethyl); C12(Carbamidomethyl)	
Q8TEH3	RPKsNIAVEGR	1	S4(Phospho)	S(4): 100.0
Q7Z3G6	SRsDNALHLASER	1	S3(Phospho)	S(1): 7.8; S(3): 92.2; S(11): 0.0
Q15154	VTNDIsPEsSPGVGR	2	S6(Phospho); S9(Phospho)	T(2): 0.0; S(6): 99.4; S(9): 7.9; S(10): 92.7
P04792	HEERQDEHGYISR	4		
Q9Y2D5	SEGDNYSATLLEPAASSLsPDHK	1	S19(Phospho)	S(1): 0.0; Y(6): 0.3; S(7): 0.3; T(9): 0.3; S(16): 7.9; S(17): 45.6; S(19): 45.6
Q12929	RKsQMEEVQDELIHR	1	S3(Phospho)	S(3): 100.0
O94929	RFsSGGEEDDFDR	1	S3(Phospho)	S(3): 90.8; S(4): 9.2
Q71U36	TIGGGDDSFNTFFSETGAGK	1		
P49840	TsSFAEPGGGGGGGGGGPGGSASGPGGTGGGK	3	S2(Phospho)	T(1): 46.4; S(2): 46.4; S(3): 7.1; S(21): 0.0; S(23): 0.0; T(28): 0.0
Q9UHB6	SEVQQPVHPKPLsPDSR	1	S13(Phospho)	S(1): 0.0; S(13): 100.0; S(16): 0.0
P27797	IKDPDASKPEDWDER	2		
P05455	FAsDDEHDEHDENGATGPVK	1	S3(Phospho)	S(3): 100.0; T(16): 0.0
P13645	DAEAWFNEK	1		
Q15154	ILEDHGsPAGEIDDEDKDKDETETVK	1	S7(Phospho)	S(7): 100.0; T(22): 0.0; T(24): 0.0
P07900	DKEVsDDEAEEKEDK	1	S5(Phospho)	S(5): 100.0
Q8IWS0	NHSGNDERDEEDEERESK	2		
Q9UPU7	DTSPDKGELVsDEEEDT	2	S11(Phospho)	T(2): 0.0; S(3): 0.0; S(11): 100.0; T(17): 0.0
P27816	DVtPPPETEVVLIK	4	T3(Phospho)	T(3): 100.0; T(8): 0.0
Q9NZ63	VGDTEKPEPERsPPNR	4	S12(Phospho)	T(4): 0.1; S(12): 99.9
Q8IZ21	SSsPVQVEEEPVR	3	S3(Phospho)	S(1): 7.0; S(2): 7.0; S(3): 85.9
Q8N6T3	sSDSWEVWGSASTNR	2	S1(Phospho)	S(1): 46.9; S(2): 46.9; S(4): 6.1; S(10): 0.1; S(12): 0.0; T(13): 0.0
Q9H4A3	DVDDGSGsPHsPHQLSSK	2	S8(Phospho); S11(Phospho)	S(6): 52.8; S(8): 52.8; S(11): 94.4; S(16): 0.0; S(17): 0.0
Q9UFC0	ANsPEKPPEAGAAHKPR	2	S3(Phospho)	S(3): 100.0
Q9NRY2	DHAEQQHIAAQQK	1		
Q13459	SPLEHsSPEKEAPSPEK	2	S6(Phospho)	S(1): 0.0; S(6): 50.0; S(7): 50.0; S(14): 0.0
P51116	TDGsIsGDRQPVTVADYISR	2	S4(Phospho); S6(Phospho)	T(1): 15.1; S(4): 87.1; S(6): 97.9; T(13): 0.0; Y(17): 0.0; S(19): 0.0
Q96ST2	MDsDEDEKEGEEEKVAK	1	S3(Phospho)	S(3): 100.0
P49023	sSPGGQDEGGFMAQGK	2	S1(Phospho)	S(1): 50.0; S(2): 50.0
Q9P0K7	sLEDVTAEYIHK	1	S1(Phospho)	S(1): 100.0; T(6): 0.0; Y(9): 0.0
Q9UQB8	SSsMAAGLER	2	S3(Phospho)	S(1): 7.4; S(2): 7.4; S(3): 85.1
Q2M2I8	VGSLtPPSsPK	4	T5(Phospho); S9(Phospho)	S(3): 0.0; T(5): 99.7; S(8): 50.2; S(9): 50.2
Q9UNE7	NHEGDEDDSHVR	2		
Q9HC35	EKKEESHSNDQSPQIR	1		
P50552	KVsKQEEASGGPTAPK	2	S3(Phospho)	S(3): 100.0; S(9): 0.0; T(13): 0.0
Q8IYB3	VPKPEPIPEPKEPsPEK	1	S14(Phospho)	S(14): 100.0
P78559	STTSQVTPAEEKDGHsPMSK	2	S16(Phospho)	S(1): 0.0; T(2): 0.0; T(3): 0.0; S(4): 0.0; T(7): 0.0; S(16): 88.8; S(19): 11.2
O60506	DSDLSHVQNK	1		
Q96KR1	RRDsDGVDGFEAEGK	1	S4(Phospho)	S(4): 100.0
Q9UKJ3	KPsVSEEVQATPNK	1	S3(Phospho)	S(3): 99.4; S(5): 0.6; T(11): 0.0
P53621	DADSQNPDAPEGK	1		
Q13131	SIDDEITEAKSGtAtPQR	2	T13(Phospho); T15(Phospho)	S(1): 0.0; T(7): 0.2; S(11): 89.8; T(13): 89.6; T(15): 20.3
P14618	LDIDSPPITAR	1		
Q9BV36	NEQLPLQYLADVDTsDEESIR	4	S15(Phospho)	Y(8): 0.0; T(14): 1.3; S(15): 88.5; S(19): 10.2
O43852	HLVYESDQNKDGK	2		
Q7Z309	RIDFTPVsPAPsPTR	1	S8(Phospho); S12(Phospho)	T(5): 0.0; S(8): 100.0; S(12): 99.1; T(14): 0.9
Q09666	SSKAsLGsLEGEAEAEAsSPK	2	S5(Phospho); S8(Phospho); S18(Phospho)	S(1): 18.8; S(2): 18.8; S(5): 73.6; S(8): 88.9; S(18): 1.7; S(19): 98.3
O14579	DSIVAELDR	1		
Q9NUG6	DQDHLDKEIEK	1		
Q01518	NSLDcEIVSAK	1	C5(Carbamidomethyl)	
O75379	RNLLEDDsDEEEDFFLR	2	S8(Phospho)	S(8): 100.0
Q7Z4V5	KRsEGFSMDR	1	S3(Phospho)	S(3): 100.0; S(7): 0.0
Q5VZK9	SNDsGEEAEKEFIFV	1	S4(Phospho)	S(1): 50.0; S(4): 50.0
P02545;P02545	LRLsPsPTSQR	2	S4(Phospho); S6(Phospho)	S(4): 91.6; S(6): 15.8; T(8): 84.2; S(9): 8.4
O00499	SPsPPDGSPAATPEIR	4	S3(Phospho)	S(1): 6.7; S(3): 93.3; S(8): 0.0; T(12): 0.0
Q9UHR5	GGLVsDAYGEDDFSR	1	S5(Phospho)	S(5): 99.7; Y(8): 0.2; S(14): 0.0
Q13813	DADETKEWIEEK	1		
P49321	KPEEEsPRKDDAK	4	S6(Phospho)	S(6): 100.0
P46821	tAtcHSSSSPPIDAASAEPYGFR	1	T1(Phospho); T3(Phospho); C4(Carbamidomethyl)	T(1): 5.2; T(3): 5.2; S(6): 77.9; S(7): 37.2; S(8): 37.2; S(9): 37.2; S(16): 0.0; Y(20): 0.0
Q15648	DRHESVGHGEDFSK	2		
P13010	DQVTAQEIFQDNHEDGPTAK	1		
Q15477	ASsLEDLVLK	1	S3(Phospho)	S(2): 50.0; S(3): 50.0
O14617	TEAQGEEDDAEGQDQDKKsPKPK	2	S19(Phospho)	T(1): 0.0; S(19): 100.0
P18887	TKPtQAAGPSSPQKPPtPEETK	2	T4(Phospho); T17(Phospho)	T(1): 0.0; T(4): 0.0; S(10): 50.0; S(11): 50.0; T(17): 100.0; T(21): 0.0
Q86UU1	TRsPsPTLGESLAPHK	1	S3(Phospho); S5(Phospho)	T(1): 7.3; S(3): 92.7; S(5): 99.9; T(7): 0.1; S(11): 0.0
Q9UII2	HHEEEIVHHKK	2		
P60709	GYSFTTTAER	1		
Q5T4S7	HVTLPSsPR	1	S7(Phospho)	T(3): 8.7; S(6): 8.7; S(7): 82.7
Q96BY6	LPSHSFEIDHEDADKDEDTTSHSSSK	1		
Q9C0C2	SPAEcREEHSKtPEER	3	C5(Carbamidomethyl); T12(Phospho)	S(1): 0.0; S(10): 11.1; T(12): 88.9
P09382	DGGAWGTEQR	1		
Q15637	TGDLGIPPNPEDRsPsPEPIYNSEGK	12	S14(Phospho); S16(Phospho)	T(1): 0.0; S(14): 100.0; S(16): 97.8; Y(21): 1.9; S(23): 0.3
Q641Q2	AGNsDSEEDDANGRVELILEPK	2	S4(Phospho)	S(4): 99.9; S(6): 0.1
Q07960	SSsPELVTHLK	2	S3(Phospho)	S(1): 0.0; S(2): 0.4; S(3): 99.5; T(8): 0.0
P07900;P08238	EKYIDQEELNK	1		

P08238;P14625	ELISNASDALDK	1		
Q9GZT9	AKPPADPAAAAsPcR	1	S12(Phospho); C14(Carbamidomethyl)	S(12): 100.0
Q641Q2	GLFSDEEDsEDLFSSQSASK	2	S9(Phospho)	S(4): 0.2; S(9): 99.8; S(14): 0.0; S(15): 0.0; S(17): 0.0; S(19): 0.0
P07737	TFVNITPAEVGVLVGK	3		
P55786	DNWEELYNR	1		
P14625	DISTNYYASQK	1		
Q07960	DAYREFDRK	1		
P02545;P02545	LQEKEDLQELNDR	1		
Q641Q2	ERRtPsDDEEDNLFAPPK	1	T4(Phospho); S6(Phospho)	T(4): 100.0; S(6): 100.0
P63267;P60709	AGFAGDDAPR	1		
O43852	VHNDAQSFDYDHDAFLGAEEAK	1		
O60271	SASQssLDKLDQELKEQQK	4	S5(Phospho); S6(Phospho)	S(1): 11.3; S(3): 87.6; S(5): 12.4; S(6): 88.7
P63267;P60709	DSYVGDEAQSKR	2		
Q96F86	HNsWSSSSR	1	S3(Phospho)	S(3): 48.0; S(5): 48.0; S(6): 4.0; S(7): 0.0; S(8): 0.0
Q14643	RDsVLAASR	1	S3(Phospho)	S(3): 100.0; S(8): 0.0
Q8WWM7	TESVSDKEDKPPLAPSGGTEGPEQPPPPcPSQTGsPPVGLIK	3	C29(Carbamidomethyl); S35(Phospho)	T(1): 0.0; S(3): 0.0; S(5): 0.0; S(16): 0.0; T(19): 0.0; S(31): 3.8; T(33): 17.1; S(35): 79.1
P53618	TNNVSEHEDTDKYR	1		
P53602	RNsRDGDPLPSSLScK	1	S3(Phospho); C15(Carbamidomethyl)	S(3): 100.0; S(11): 0.0; S(12): 0.0; S(14): 0.0
P13639	YEWDVAEAR	1		
Q92769	IAcDEEFsDsEDEGEGGRR	4	C3(Carbamidomethyl); S8(Phospho); S10(Phospho)	S(8): 100.0; S(10): 100.0
Q96EB6	RDGPGLERsPGEPGGAAPER	1	S9(Phospho)	S(9): 100.0
P06241	LIEDNEyTAR	1	Y7(Phospho)	Y(7): 94.3; T(8): 5.7
Q00587	SDsLLSFR	1	S3(Phospho)	S(1): 0.0; S(3): 100.0; S(6): 0.0
Q15170	TDEERPPVEHsPEK	1	S11(Phospho)	T(1): 0.0; S(11): 100.0
Q9BXW6	sFEEEGEHLGSR	1	S1(Phospho)	S(1): 100.0; S(11): 0.0
Q8WVC0	KLTsDEEGEPSGKR	1	S4(Phospho)	T(3): 50.0; S(4): 50.0; S(11): 0.0
Q9UKS6	DGTAPPPQSPGSPGTGQDEEWsDEESPRK	4	S22(Phospho)	T(3): 0.0; S(9): 0.1; S(12): 0.9; T(15): 3.9; S(22): 94.3; S(26): 0.9
P00441	ADDLGKGGNEESTK	1		
O94804	ASQsRPNsSALETLGGEK	3	S4(Phospho); S8(Phospho)	S(2): 0.7; S(4): 99.3; S(8): 99.3; S(9): 0.7; T(13): 0.0
P00338	VTLTSEEEAR	1		
P60709	QEYDESGPSIVHR	3		
P48741	ATAGDTHLGGEDFDNR	1		
P48651	TYsEcEDGTYSPEISWHHR	1	S3(Phospho); C5(Carbamidomethyl)	T(1): 0.7; Y(2): 0.7; S(3): 98.6; T(9): 0.0; Y(10): 0.0; S(11): 0.0; S(15): 0.0
Q86UE4	SQEPIPDDQKVsDDDKEK	1	S12(Phospho)	S(1): 0.0; S(12): 100.0
O94988	SSsLGSYDDEQEDLTPAQLTR	3	S3(Phospho)	S(1): 32.6; S(2): 32.6; S(3): 32.6; S(6): 1.7; Y(7): 0.4; T(15): 0.0; T(20): 0.0
P61158	DREVGIPPEQSLETAK	1		
P05455	FAsDDEHDEHDENGATGPVKR	2	S3(Phospho)	S(3): 100.0; T(16): 0.0
P78559	sPcGLTEQYLHK	1	S1(Phospho); C3(Carbamidomethyl)	S(1): 100.0; T(6): 0.0; Y(9): 0.0
Q99805	HTHIDKPDcSGPPMDISNK	1	C9(Carbamidomethyl)	
P10644	TDsREDEIsPPPPNPVVK	6	S3(Phospho); S9(Phospho)	T(1): 52.5; S(3): 52.5; S(9): 95.0
Q8IYB3	RYsPsPPPK	3	S3(Phospho); S5(Phospho)	Y(2): 0.5; S(3): 99.5; S(5): 100.0
P08670	NLQEAEEWYK	1		
Q96C19	RADLNQGIGEPQsPSR	2	S13(Phospho)	S(13): 50.0; S(15): 50.0
Q96DG6	KREDcSPADKPYIDEAR	2	C5(Carbamidomethyl)	
Q14847	MGPSGGEGMEPERRDsQDGSSYR	2	S16(Phospho)	S(4): 0.0; S(16): 100.0; S(20): 0.0; S(21): 0.0; Y(22): 0.0
Q8WWI1	REDsFESLDSLGSR	3	S4(Phospho)	S(4): 99.9; S(7): 0.1; S(10): 0.0; S(13): 0.0
Q9NSD9	DRYDSFIELQEK	2		
P13645	LAADDFR	1		
Q96D46	DSAIPVEsDtDDEGAPR	2	S8(Phospho); T10(Phospho)	S(2): 0.0; S(8): 100.0; T(10): 100.0
P06733	YISPDQLADLYK	1		
P35579	DLEGLSQR	1		
P13645	NHEEEMKDLR	1		
P61201	QLHQScQTDDGEDDLKK	1	C6(Carbamidomethyl)	
P05787	NKYEDEINKR	2		
P06703	LQDAEIAR	1		
Q6DD88	EHQHEEIQNVR	1		
Q8WWI1	EDsFESLDSLGSR	1	S3(Phospho)	S(3): 99.7; S(6): 0.3; S(9): 0.0; S(12): 0.0
Q9H694	SNsREHLGGGSESDNWR	1	S3(Phospho)	S(1): 0.9; S(3): 99.1; S(11): 0.0; S(13): 0.0
P61204	DAVLLVFANK	1		
P29692	ATAPQTQHVsPMr	1	S10(Phospho); C-Term(Oxidation)	T(2): 0.0; T(6): 1.1; S(10): 98.9
O75695	KADKESRPENEEERPK	4		
Q15052	MsGFIYQGK	1	S2(Phospho)	S(2): 100.0; Y(6): 0.0
Q16643	LSsPVLHR	1	S3(Phospho)	S(2): 0.0; S(3): 100.0
P13639	DLEEDHAcIPIK	2	C8(Carbamidomethyl)	
P27824	QKsDAEEDGGTVSQEEEDR	2	S3(Phospho)	S(3): 100.0; T(11): 0.0; S(13): 0.0
O43719	EsSPEKEAEEGcPEKESEEGcPK	2	S2(Phospho); C12(Carbamidomethyl); C21(Carbamidomethyl)	S(2): 50.0; S(3): 50.0; S(17): 0.0
P33527	HHNsTAELQK	2	S4(Phospho)	S(4): 92.0; T(5): 8.0
Q9UKE5	QNsDPTSENPPLPTR	1	S3(Phospho)	S(3): 100.0; T(6): 0.0; S(7): 0.0; T(14): 0.0
Q96RS0	DRPHASGTDGDEsEEDPPEHKPSK	3	S13(Phospho)	S(6): 0.0; T(8): 0.0; S(13): 100.0; S(23): 0.0
Q9Y266	DAENHEAQLK	1		
Q9Y2X7	SLSsPTDNLELSLR	2	S4(Phospho)	S(1): 0.0; S(3): 0.8; S(4): 91.0; T(6): 8.2; S(12): 0.0
P30041	DGDSVMVLPTIPEEEAK	1		
Q641Q2	sRPTsFADELAAR	1	S1(Phospho); S5(Phospho)	S(1): 91.6; T(4): 16.8; S(5): 91.6
Q14165	LQPHPGLEKKEEEEEEEEYDEGSNLK	3		
Q05682	RGsIGENQIKDEK	4	S3(Phospho)	S(3): 100.0
Q02952	RPsESDKEDELDKVK	5	S3(Phospho)	S(3): 99.9; S(5): 0.1
Q69YU3	RHsMQTEQIR	1	S3(Phospho)	S(3): 100.0; T(6): 0.0
Q9NXH9	GDQccYSHsPPTPR	1	C4(Carbamidomethyl); C5(Carbamidomethyl); S9(Phospho)	Y(6): 0.0; S(7): 0.0; S(9): 91.9; T(12): 8.0
Q13428	KLsGDQPAAR	2	S3(Phospho)	S(3): 100.0
Q8WWI1	RGEsLDNLDSPR	1	S4(Phospho)	S(4): 99.9; S(10): 0.1
Q9Y2B0	NGEsSELDLQGIR	2	S4(Phospho)	S(4): 90.5; S(5): 9.5
Q00613	VKEEPPsPPQsPR	1	S7(Phospho); S11(Phospho)	S(7): 100.0; S(11): 100.0
P35555	McKDEDEcEEGKHDcTEK	1	C2(Carbamidomethyl); C8(Carbamidomethyl); C15(Carbamidomethyl)	
Q96N67	SMsIDDTPR	2	S3(Phospho)	S(1): 0.9; S(3): 99.1; T(7): 0.0
O60271	SASQssLDKLDQELK	3	S5(Phospho); S6(Phospho)	S(1): 5.7; S(3): 49.7; S(5): 50.3; S(6): 94.3
Q8IYB3	REsPSPAPKPR	3	S3(Phospho)	S(3): 100.0; S(5): 0.0
P46821	SLMSsPEDLTK	1	S5(Phospho)	S(1): 0.0; S(4): 0.5; S(5): 99.5; T(10): 0.0
P46821	QGsPDQVSPVSEMTSTSLYQDKQEGK	1	S3(Phospho)	S(3): 100.0; S(8): 0.0; S(11): 0.0; T(14): 0.0; S(15): 0.0; T(16): 0.0; S(17): 0.0; Y(19): 0.0

Q92896	DAHSQGEVVScLEK	1	C11(Carbamidomethyl)	
P21980	NEFGEIQGDK	1		
P00338	LVIITAGAR	1		
P05386	KEESEEsDDDMGFGLFd	1	S7(Phospho); C-Term(Oxidation)	S(4): 0.0; S(7): 100.0
O94973	NADVELQQR	1		
P00338	TLHPDLGTDKDKEQWK	3		
Q8IXJ6	EHASIDAQSGAGVPNPStSASPK	4	T18(Phospho)	S(4): 0.0; S(9): 0.0; S(17): 49.1; T(18): 49.1; S(19): 1.5; S(21): 0.3
Q6GYQ0	SSStSDILEPFTVER	10	T4(Phospho)	S(1): 4.3; S(2): 30.5; S(3): 30.5; T(4): 30.5; S(5): 4.3; T(12): 0.0
Q01082	ESsPIPsPTSDR	1	S3(Phospho); S7(Phospho)	S(2): 9.5; S(3): 90.5; S(7): 100.0; T(9): 0.0; S(10): 0.0
Q9UGV2	DLEIERPILGQNDNK	1		
Q8N9B5	GAAsPVLQEDHcDSLPSVLQVEEK	1	S4(Phospho); C12(Carbamidomethyl)	S(4): 99.9; S(14): 0.1; S(17): 0.0
Q13501	DHRPPcAQEAPR	1	C6(Carbamidomethyl)	
P48651	EKtYSEcEDGTYSPEISWHHR	2	T3(Phospho); C7(Carbamidomethyl)	T(3): 45.8; Y(4): 8.5; S(5): 45.8; T(11): 0.0; Y(12): 0.0; S(13): 0.0; S(17): 0.0
P04083	GVDEATIIDILTK	1		
Q9Y6G9	DFQEYVEPGEDFPAsPQRR	5	S15(Phospho)	Y(5): 0.0; S(15): 100.0
P49585	TsPPcsPANLSR	1	S2(Phospho); C5(Carbamidomethyl); S6(Phospho)	T(1): 50.0; S(2): 50.0; S(6): 100.0; S(11): 0.0
P00338	DYNVTANSK	1		
P13667	DLGLSESGEDVNAAILDESGKK	1		
P78559	ELVLssPEDLTQDFEEMKr	1	S5(Phospho); S6(Phospho); C-Term(Oxidation)	S(5): 99.9; S(6): 99.4; T(11): 0.7
Q4KMP7	KADGPPGPHDGGDRPSAEAR	2		
Q13586	DLTHsDSESSLHMSDR	1	S5(Phospho)	T(3): 0.1; S(5): 99.8; S(7): 0.1; S(9): 0.0; S(10): 0.0; S(14): 0.0
Q9H4A4	NDHQEDFWK	1		
Q96I25	RPDPDsDEDEDYERER	2	S6(Phospho)	S(6): 100.0; Y(12): 0.0
P62937	FEDENFILK	1		
P47756	DYLLcDYNR	1	C5(Carbamidomethyl)	
Q4KMP7	TEEARPSPAPGPGtPTGTPTR	2	T14(Phospho)	T(1): 0.0; S(7): 0.0; T(14): 99.9; T(16): 0.1; T(18): 0.0; T(20): 0.0
Q8NE71	LSVPTsDEEDEVPAPKPR	7	S6(Phospho)	S(2): 0.0; T(5): 0.4; S(6): 99.6
P07237	NNFEGEVTK	1		
P10644	TDSREDEIsPPPPNPVVK	9	S9(Phospho)	T(1): 0.0; S(3): 0.0; S(9): 100.0
Q92615	SPsPAHLPDDPKVAEK	2	S3(Phospho)	S(1): 1.6; S(3): 98.4
P53396	TAsFSESRADEVAPAKK	1	S3(Phospho)	T(1): 10.2; S(3): 89.6; S(5): 0.2; S(7): 0.0
P08621	YDERPGPsPLPHR	2	S8(Phospho)	Y(1): 0.0; S(8): 100.0
P07355	DALNIETAIK	1		
P50613	AYtHQVVTR	1	T3(Phospho)	Y(2): 0.5; T(3): 99.5; T(8): 0.0
P27824	QKsDAEEDGGTVsQEEEDRKPK	5	S3(Phospho); S13(Phospho)	S(3): 98.9; T(11): 50.6; S(13): 50.6
Q5FWE3	sIDAALFR	1	S1(Phospho)	S(1): 100.0
Q8IWC1	DSNLHSSTDKEQAER	1		
Q9UKV3	SSSISEEKGDsDDEKPR	1	S11(Phospho)	S(1): 0.0; S(2): 0.0; S(3): 0.0; S(5): 0.0; S(11): 100.0
O75592	VNsGDTEVGSSLLR	1	S3(Phospho)	S(3): 99.9; T(6): 0.1; S(10): 0.0; S(11): 0.0
P09651	SEsPKEPEQLR	4	S3(Phospho)	S(1): 7.0; S(3): 93.0
Q641Q2	ASALLFsSDEEDQWNIPASQTHLASDSR	3	S7(Phospho)	S(2): 0.1; S(7): 49.9; S(8): 49.9; S(19): 0.0; T(21): 0.0; S(25): 0.0; S(27): 0.0
Q15785	NRVPsAGDVEK	1	S5(Phospho)	S(5): 100.0
Q9H7D0	sLQLMDNR	1	S1(Phospho)	S(1): 100.0
P62158	DTDSEEEIREAFR	1		
Q8WX93	sRsRDsGDENEPIQER	1	S1(Phospho); S3(Phospho); S6(Phospho)	S(1): 100.0; S(3): 100.0; S(6): 100.0
Q9UHB6	ETPHsPGVEDAPIAK	2	S5(Phospho)	T(2): 0.0; S(5): 100.0
Q09666	SSKAsLGsLEGEAEAEAsSPKGK	2	S5(Phospho); S8(Phospho); S18(Phospho)	S(1): 0.1; S(2): 0.1; S(5): 99.7; S(8): 100.0; S(18): 50.0; S(19): 50.0
Q02952	LTASEQAHPQEPAESAHEPRLSAEyEK	2	Y25(Phospho)	T(2): 0.0; S(4): 0.0; S(15): 1.4; S(22): 19.3; Y(25): 79.3
P00519	GQGESDPLDHEPAVsPLLPR	2	S15(Phospho)	S(5): 0.0; S(15): 100.0
Q01484	GGsPIIQEPEEPSEHREESSPR	1	S3(Phospho)	S(3): 100.0; S(13): 0.0; S(19): 0.0; S(20): 0.0
P27816	ETERAsPIKMDLAPSK	2	S6(Phospho)	T(2): 0.0; S(6): 100.0; S(15): 0.0
Q05682;Q05682	EDKEKEEEEEKPK	1		
P67936;P06753	IQLVEEELDR	1		
Q9H0B6	RSsRDMAGGAGPR	1	S3(Phospho)	S(2): 0.7; S(3): 99.3
P14618	APIIAVTR	1		
Q15084	NRPEDYQGGR	1		
Q9Y5K6	FNGGHsPTHSPEK	1	S6(Phospho)	S(6): 99.9; T(8): 0.1; S(10): 0.0
P25685	YHPDKNKEPGAEEK	2		
P19338	KVVVsPTK	3	S5(Phospho)	S(5): 99.4; T(7): 0.6
Q14141	EAELKEAEKELHEK	2		
O94826	SDEDKDKEGEALEVK	2		
O95394	STIGVMVTAsHNPEEDNGVK	2	S10(Phospho)	S(1): 0.0; T(2): 0.0; T(8): 1.8; S(10): 98.2
Q8WYL5	SSsSDSIHSVR	3	S3(Phospho)	S(1): 0.0; S(2): 0.4; S(3): 99.2; S(4): 0.4; S(6): 0.0; S(9): 0.0
P49840	GEPNVSyIcSR	5	Y7(Phospho); C9(Carbamidomethyl)	S(6): 4.5; Y(7): 95.5; S(10): 0.0
Q14315	ITGDDSMR	1		
Q9NR19	SWsPPPEVSR	3	S3(Phospho)	S(1): 0.0; S(3): 100.0; S(9): 0.0
Q06830	ADEGISFR	1		
Q96RT1	DLKPHEDQQDINKDVGVK	2		
P40818	SYsSPDITQAIQEEEKR	4	S3(Phospho)	S(1): 6.1; Y(2): 6.1; S(3): 87.3; S(4): 0.5; T(8): 0.0
P20073	DENQSINHQMAQEDAQR	1		
P35241	KKEEEATEWQHK	1		
Q6Y7W6	WRPHsPDGPR	3	S5(Phospho)	S(5): 100.0
P35611	QKGsEENLDEAR	1	S4(Phospho)	S(4): 100.0
Q13428	AALAPAKEsPR	1	S9(Phospho)	S(9): 100.0
Q15435	RVEsEEsGDEEGKK	1	S4(Phospho); S7(Phospho)	S(4): 100.0; S(7): 100.0
Q16181	GQLTKsPLAQMEEERR	1	S6(Phospho)	T(4): 0.1; S(6): 99.9
P63104;P31946	NLLSVAYK	1		
O95400	KGPGQPsSPQR	2	S7(Phospho)	S(7): 99.4; S(8): 0.6
O95425	cTsHSETPTVDDEEKVDER	4	C1(Carbamidomethyl); S3(Phospho)	T(2): 6.5; S(3): 6.5; S(5): 1.1; T(7): 42.9; T(9): 42.9
P49736	RTDALTSsPGR	3	S8(Phospho)	T(2): 0.0; T(6): 0.0; S(7): 0.5; S(8): 99.5
Q9NVD7	SPTPKsPPSR	4	S6(Phospho)	S(1): 0.0; T(3): 0.0; S(6): 93.5; S(9): 6.5
Q9C0C2	RPsQGPPAR	1	S3(Phospho)	S(3): 100.0
Q9H1E3	NSQEDsEDsEDKDVK	1	S6(Phospho); S9(Phospho)	S(2): 0.0; S(6): 100.0; S(9): 100.0
Q8IW50	GYSSLDQSPDEKPLVALDTDsDDDFDMSr	2	S21(Phospho); C-Term(Oxidation)	Y(2): 0.0; S(3): 0.0; S(4): 0.0; S(8): 0.0; T(19): 48.3; S(21): 48.3; S(28): 3.4
P78559	EESEPEVKEDVIEKAELEEMEEVHPsDEEEEDATK	2	S26(Phospho)	S(3): 0.0; S(26): 50.0; T(34): 50.0
Q3KQU3	RSsQPsPTAVPAsDSPPTKQEVK	1	S3(Phospho); S6(Phospho); S13(Phospho)	S(2): 10.8; S(3): 89.2; S(6): 10.8; T(8): 89.2; S(13): 0.2; S(15): 98.3; T(18): 1.5
Q9P260	AGSISTLDSLDFARYsDDGNRETDEK	2	S16(Phospho)	S(3): 0.0; S(5): 0.0; T(6): 0.0; S(9): 0.1; Y(15): 48.9; S(16): 48.9; T(23): 2.1
P46821	TLEVVsPsQsVTGSAGHTPYYQSPTDEK	4	S6(Phospho); S8(Phospho); S10(Phospho)	T(1): 0.1; S(6): 98.1; S(8): 75.5; S(10): 24.4; T(12): 24.4; S(14): 74.4; T(18): 0.9; Y(20): 1.1; Y(21): 1.1; S(23): 0.0; T(25): 0.0

Q12983	ASETDTHsIGEK	1	S8(Phospho)	S(2): 0.0; T(4): 0.0; T(6): 50.0; S(8): 50.0
Q8N5D0	KDsISEDEMVLR	1	S3(Phospho)	S(3): 99.9; S(5): 0.1
Q5SW79	NQATSAtSEKDNDDDQSDKGtYTIELENPNSEEVEAR	1	T7(Phospho); T21(Phospho)	T(4): 8.0; S(5): 8.0; T(7): 41.7; S(8): 41.7; S(17): 2.1; T(21): 8.9; Y(22): 44.8; T(23): 44.8; S(31): 0.0
O43150	LLHEDLDEsDDDMDEKLQPSPNR	2	S9(Phospho)	S(9): 100.0; S(20): 0.0
Q06830	HGEVcPAGWKPGSDTIKPDVQK	2	C5(Carbamidomethyl)	
Q9Y2W2	RRDEDMLYsPELAQR	1	S9(Phospho)	Y(8): 1.5; S(9): 98.5
P46821	TLEVVSPSQSVtGSAGHtPYYQSPTDEK	1	T12(Phospho); T18(Phospho)	T(1): 0.0; S(6): 7.2; S(8): 42.6; S(10): 42.6; T(12): 7.2; S(14): 0.3; T(18): 0.1; Y(20): 1.4; Y(21): 7.7; S(23): 45.4; T(25): 45.4
Q92466	SRsPLELEPEAKK	1	S3(Phospho)	S(1): 5.7; S(3): 94.3
Q8NDI1	DLStSPKPsPIPsPVLGR	5	T4(Phospho); S9(Phospho); S13(Phospho)	S(3): 5.5; T(4): 47.2; S(5): 47.2; S(9): 100.0; S(13): 100.0
P35658	SPGsTPTTPTsSQAPQK	1	S4(Phospho); S11(Phospho)	S(1): 88.8; S(4): 10.0; T(5): 1.2; T(7): 44.9; T(8): 44.9; T(10): 5.0; S(11): 5.0; S(12): 0.1
O60343	TSsTcsNEsLSVGGTSVTPR	2	S3(Phospho); C5(Carbamidomethyl); S6(Phospho); S9(Phospho)	T(1): 32.7; S(2): 32.7; S(3): 32.7; T(4): 18.7; S(6): 81.1; S(9): 17.3; S(11): 84.8; T(15): 0.0; S(16): 0.0; T(18): 0.0
Q99614	LLRDDEAHLQEDQGEEEcFHDcSAsFEEEPGADKVENK	2	C18(Carbamidomethyl); C22(Carbamidomethyl); S25(Phospho)	S(23): 50.0; S(25): 50.0
Q9NQX3	VKEVHDELEDLPsPPPPLsPPPTTSPHK	2	S13(Phospho); S19(Phospho)	S(13): 100.0; S(19): 100.0; T(23): 0.0; T(24): 0.0; S(25): 0.0
Q9NW97	QGEDLAHVQHPTGAGPHAQEEDsQEEEEEDEEAASR	2	S23(Phospho)	T(12): 50.0; S(23): 50.0; S(35): 0.0
Q96B97	ANsPSLFGTEGKPK	1	S3(Phospho)	S(3): 99.3; S(5): 0.7; T(9): 0.0
Q96C92	VSPAsPAGsPSADFAVHGESLGDR	1	S5(Phospho); S9(Phospho)	S(2): 4.7; S(5): 96.0; S(9): 98.3; S(11): 1.1; S(20): 0.0
Q15293	VVRPDSELGERPPEDNQSFQYDHEAFLGKEDSK	1		
Q71U36	AVFVDLEPTVIDEVR	1		
Q9Y3P9	EVLDEDtDEEKETLK	1	T7(Phospho)	T(7): 100.0; T(13): 0.0
Q5ZPR3	HSDsKEDDGQEIA	1	S4(Phospho)	S(2): 0.0; S(4): 100.0
P23634	TQDGVALEIQPLNsQEGIDNEEKDKK	1	S14(Phospho)	T(1): 0.0; S(14): 100.0
Q8IWE5	DQPsPcLSSAEDSGVDEGQGSPSEMVHSSEFR	1	S4(Phospho); C6(Carbamidomethyl)	S(4): 98.0; S(8): 0.7; S(9): 0.7; S(13): 0.7; S(21): 0.0; S(23): 0.0; S(28): 0.0; S(29): 0.0
Q9UKK3	NYDPYKPLDItPPPDQK	1	T11(Phospho)	Y(2): 0.0; Y(5): 0.0; T(11): 100.0
Q9BZZ5	RASEDTTSGsPPKK	2	S10(Phospho)	S(3): 0.0; T(6): 0.0; T(7): 0.0; S(8): 0.0; S(10): 100.0
O75909	AVVVsPKEENK	1	S5(Phospho)	S(5): 100.0
Q8WUM9	EIKcSPsEsPLMEK	2	C4(Carbamidomethyl); S7(Phospho); S9(Phospho)	S(5): 9.5; S(7): 90.5; S(9): 100.0
P02545;P02545	GRAsSHSSQTQGGGSVTK	2	S4(Phospho)	S(4): 89.4; S(5): 10.6; S(7): 0.0; S(8): 0.0; T(10): 0.0; S(15): 0.0; T(17): 0.0
Q6R327	GRNDsGEENVPLDLTREPSDNLR	2	S5(Phospho)	S(5): 100.0; T(15): 0.0; S(19): 0.0
P51397	EEKDKDDQEWEsPSPPKPTVFISGVIAR	1	S12(Phospho)	S(12): 0.7; S(14): 98.7; T(19): 0.7; S(23): 0.0
Q9UQ35	RGEGDAPFSEPGTTSTQRPSsPETATK	5	S21(Phospho)	S(9): 0.0; T(13): 0.0; T(14): 0.0; S(15): 0.0; T(16): 0.0; S(20): 14.0; S(21): 85.6; T(24): 0.4; T(26): 0.0
Q86WB0	SQDATFSPGSEQAEKsPGPIVSR	1	S16(Phospho)	S(1): 0.0; T(5): 0.0; S(7): 0.0; S(10): 0.5; S(16): 97.0; S(22): 2.6
P30086	NRPtSISWDGLDSGK	2	T4(Phospho)	T(4): 49.8; S(5): 49.8; S(7): 0.4; S(13): 0.0
Q13557	KPDGVKEStESSNtTIEDEDVK	1	T9(Phospho); T14(Phospho)	S(8): 89.9; T(9): 10.0; S(11): 0.3; S(12): 9.0; T(14): 9.0; T(15): 81.8
O15027	QIDSsPVGGETDETTVSQNYR	2	S5(Phospho)	S(4): 0.0; S(5): 2.2; T(11): 95.6; T(14): 2.2; T(15): 0.0; S(17): 0.0; Y(20): 0.0
O75976	SLLSHEFQDEtDtEEETLYSSK	2	T11(Phospho); T13(Phospho)	S(1): 0.8; S(4): 4.6; T(11): 95.2; T(13): 83.4; T(17): 15.7; Y(19): 0.2; S(20): 0.0; S(21): 0.0
P49585	MLQAIsPKQsPsSSPTR	1	S6(Phospho); S10(Phospho); S12(Phospho)	S(6): 99.8; S(10): 99.8; S(12): 0.4; S(13): 9.5; S(14): 90.3; T(16): 0.0
P05114	KVsSAEGAAKEEPK	1	S3(Phospho)	S(3): 50.0; S(4): 50.0
Q07866	ALSAsHTDLAH	1	S5(Phospho)	S(3): 0.0; S(5): 99.4; T(7): 0.5
Q15435	RVEsEESGDEEGKK	1	S4(Phospho)	S(4): 99.8; S(7): 0.2
P42684	DKsPSSLLEDAK	1	S3(Phospho)	S(3): 98.3; S(5): 0.9; S(6): 0.9
Q9Y4J8	TQFEDLVPsPTSEK	1	S9(Phospho)	T(1): 0.0; S(9): 98.6; T(11): 1.3; S(12): 0.2
Q15121	DIIRQPsEEEIIK	3	S7(Phospho)	S(7): 100.0
Q9C0C2	DAELQDQEFGKRDsLGTYSSR	2	S14(Phospho)	S(14): 99.8; T(17): 0.1; Y(18): 0.0; S(19): 0.0; S(20): 0.0
Q5SW79	VVTQRsEIGEKQDTELQEK	2	S6(Phospho)	T(3): 1.5; S(6): 98.5; T(14): 0.0
Q8TCJ2	ENPPVEDsSDEDDKRNQGNLYDK	2	S8(Phospho)	S(8): 50.0; S(9): 50.0; Y(21): 0.0
P60709	VAPEEHPVLLTEAPLNPK	1		
O60303	KDsLSQLEEYLR	1	S3(Phospho)	S(3): 98.5; S(5): 1.5; Y(10): 0.0
Q9UPP1	DAEYIYPSLEsDDDDPALK	1	S11(Phospho)	Y(4): 0.0; Y(6): 0.0; S(8): 0.0; S(11): 100.0
Q9UJX5	IKEEVLsESEAENQQAGAAALAPEIVIK	2	S7(Phospho)	S(7): 80.2; S(9): 19.8
Q7RTP6	RKTsQSEEEEAPR	2	S4(Phospho)	T(3): 7.5; S(4): 7.5; S(6): 84.9
P07355	RAEDGsVIDYELIDQDAR	3	S6(Phospho)	S(6): 86.3; Y(10): 13.7
Q15678	YVSGssPDLVTR	1	S5(Phospho); S6(Phospho)	Y(1): 0.0; S(3): 1.8; S(5): 98.5; S(6): 99.8; T(11): 0.0
Q8WUM9	EIKcsPSESPLMEK	3	C4(Carbamidomethyl); S5(Phospho)	S(5): 11.1; S(7): 77.7; S(9): 11.1
O75955	DIHDDQDYLHSLGK	1		
Q9Y613	sLEGGGcPAR	1	S1(Phospho); C7(Carbamidomethyl)	S(1): 100.0
Q9H4A3	DVDDGSGSPHsPHQLSSK	2	S11(Phospho)	S(6): 0.1; S(8): 1.1; S(11): 98.7; S(16): 0.0; S(17): 0.0
Q9BRS8	YLSPGWGsAsEEEPSR	1	S8(Phospho); S10(Phospho)	Y(1): 0.0; S(3): 0.0; S(8): 100.0; S(10): 100.0; S(15): 0.0
Q12888	IDEDGENTQIEDTEPMsPVLNSK	1	S17(Phospho)	T(8): 0.0; T(13): 0.0; S(17): 99.8; S(22): 0.2
Q9UFC0	AcAsPSAQVEGSPVAGSDGSQPAVK	2	C2(Carbamidomethyl); S4(Phospho)	S(4): 99.7; S(6): 0.3; S(12): 0.0; S(17): 0.0; S(20): 0.0
Q86TC9	TPVDEsDDEIQHDEIPTGK	1	S6(Phospho)	T(1): 0.0; S(6): 100.0; T(17): 0.0
O00461	DNQHQDEAEGDPGNRHEPR	2		
O00499	SPsPPDGsPAATPEIR	2	S3(Phospho); S8(Phospho)	S(1): 50.0; S(3): 50.0; S(8): 50.0; T(12): 50.0
P46821	GAEsPFEEK	2	S4(Phospho)	S(4): 100.0
Q8NFQ8	DKTEDENKQSFLDGGK	1		
Q13501	SRLtPVSPESSSTEEK	2	T4(Phospho)	S(1): 11.1; T(4): 88.9; S(7): 0.0; S(10): 0.0; S(11): 0.0; S(12): 0.0; T(13): 0.0
P04075	GILAADESTGsIAKR	3	S11(Phospho)	S(8): 8.6; T(9): 8.6; S(11): 82.9
Q8TDM6	ATHGSNsLPSSAR	1	S7(Phospho)	T(2): 0.0; S(5): 0.0; S(7): 1.3; S(10): 88.5; S(11): 10.2
O60333	GDsLILEHQWELEK	1	S3(Phospho)	S(3): 100.0
P10451	ISHELDSAsSEVN	1	S9(Phospho)	S(2): 0.0; S(7): 0.5; S(9): 93.1; S(10): 6.4
P42167	GPPDFssDEEREPTPVLGsGAAAAGR	2	S6(Phospho); S7(Phospho); S19(Phospho)	S(6): 100.0; S(7): 100.0; T(14): 100.0; S(19): 0.0
P40692	HREDSDVEMVEDDSRK	1		
Q7Z417	DYEIESQNPLAsPTNTLLGSAK	1	S12(Phospho)	Y(2): 0.0; S(6): 0.0; S(12): 99.5; T(14): 0.5; T(16): 0.1; S(20): 0.0
Q15811	SAFTPATATGSsPsPVLGQGEK	2	S12(Phospho); S14(Phospho)	S(1): 0.0; T(4): 0.0; T(7): 0.0; T(9): 0.7; S(11): 49.7; S(12): 49.7; S(14): 99.9
P51858	GNAEGSsDEEGKLVIDEPAK	1	S7(Phospho)	S(6): 87.0; S(7): 13.0
Q5VTL8	RRsQsIEQESQEK	1	S3(Phospho); S5(Phospho)	S(3): 100.0; S(5): 100.0; S(10): 0.0
O95905	ESESVSKEEKEQNYDLTEVSESMK	2		
P29966	AEDGATPSPsNETPKK	1	S10(Phospho)	T(6): 0.0; S(8): 0.1; S(10): 50.0; T(13): 50.0
P54920	KLLEAHEEQNVDSYTESVKEYDSISR	1		
P63267	YPIEHGIITNWDDMEK	1		
Q8WVC0	KLTsDEEGEPSGK	2	S4(Phospho)	T(3): 2.2; S(4): 97.8; S(11): 0.0
P84157	GPSSEGPEEEDGEGFSFKYsPGK	2	S20(Phospho)	S(3): 0.0; S(4): 0.4; S(16): 33.2; Y(19): 33.2; S(20): 33.2
O94851	sWNQGtPPLELLAERESLyR	1	N-Term(Acetyl); T6(Phospho); Y19(Phospho)	S(1): 3.3; T(6): 96.7; S(17): 14.9; Y(19): 85.1
O60333	SGLsLEELR	1	S4(Phospho)	S(1): 0.1; S(4): 99.9
P23497	GFENVIHDKLPLQEsEEEEREER	1	S15(Phospho)	S(15): 100.0
Q9H7L9	RPAsPsSPEHLPATPAESPAQR	1	S4(Phospho); S6(Phospho)	S(4): 99.9; S(6): 50.0; S(7): 50.0; T(14): 0.0; S(18): 0.0
Q09666	GDRsPEPGQTWTR	1	S4(Phospho)	S(4): 100.0; T(10): 0.0; T(12): 0.0
P01130	DWSDEPIKEcGTNEcLDNNGGcSHVcNDLK	1	C10(Carbamidomethyl); C15(Carbamidomethyl); C22(Carbamidomethyl); C26(Carbamidomethyl)	
Q86UU1	KNsITEISDNEDDLLEYHRR	1	S3(Phospho)	S(3): 89.2; T(5): 10.8; S(8): 0.0; Y(17): 0.0

Q03001	AMVDSQQKsPVK	1	S9(Phospho)	S(5): 0.1; S(9): 99.9
Q7Z5L9	RKPsPEPEGEVGPPK	3	S4(Phospho)	S(4): 100.0
P06753	YSQKEDKYEEEIK	2		
P15407	RRPcEQIsPEEEER	1	C4(Carbamidomethyl); S8(Phospho)	S(8): 100.0
Q92574	GGFDsPFYR	1	S5(Phospho)	S(5): 100.0; Y(8): 0.0
Q13439	TSsFTEQLDEGTPNRENASTHASK	6	S3(Phospho)	T(1): 13.9; S(2): 13.9; S(3): 69.2; T(5): 3.0; T(12): 0.0; S(19): 0.0; T(20): 0.0; S(23): 0.0
O60343	RHAsAPSHVQPSDSEK	1	S4(Phospho)	S(4): 87.7; S(7): 12.3; S(12): 0.0; S(14): 0.0
P07900	DQVANSAFVER	1		
Q96MW1	KAALLAQYADVtDEEDEADEKDDSGATTMNIGSDK	1	T12(Phospho)	Y(8): 12.5; T(12): 81.7; S(24): 1.3; T(27): 1.3; T(28): 2.5; S(33): 0.7
Q96PD2	AVDHEETPVRYSSsEVNHLSPR	3	S14(Phospho)	T(7): 0.4; Y(11): 8.4; S(12): 41.3; S(13): 41.3; S(14): 8.4; S(20): 0.0
P27824	EIEDPEDRKPEDWDERPK	1		
Q9GZY8	NDsLPVLR	1	S3(Phospho)	S(3): 100.0
Q8WVV9	LKTEEGEIDYsAEEGENRR	1	S11(Phospho)	T(3): 0.0; Y(10): 92.0; S(11): 8.0
Q14697	QGSKDPAEGDGAQPEETPRDGDKPEETQGK	1		
Q92974	LQDSSDPDtGSEEEGSSRLsPPHsPR	3	T9(Phospho); S20(Phospho); S24(Phospho)	S(4): 17.3; S(5): 82.4; T(9): 0.4; S(11): 0.8; S(16): 46.7; S(17): 46.6; S(20): 23.3; S(24): 82.5
Q8N6H7	DNPFSLGEsFGSR	2	S9(Phospho)	S(5): 0.0; S(9): 100.0; S(12): 0.0
Q13813	KNNHHEENISSK	3		
Q15293	DGKLDKDEIR	1		
Q9UNE7	LGAGGGsPEKsPSAQELK	1	S7(Phospho); S11(Phospho)	S(7): 99.8; S(11): 84.4; S(13): 15.8
Q9Y2D5	QAPPHIELSNSsPDPMAEAER	1	S12(Phospho)	S(9): 0.1; S(11): 12.4; S(12): 87.6
P43487	DTHEDHDTSTENTDEsNHDPQFEPIVSLPEQEIK	1	S16(Phospho)	T(2): 0.3; T(8): 0.1; S(9): 0.1; T(10): 0.3; T(13): 49.7; S(16): 49.7; S(27): 0.0
Q4V328	SLSSsPQAQPPRPAELSDEEVAELFQR	4	S5(Phospho)	S(1): 24.9; S(3): 24.9; S(4): 24.9; S(5): 24.9; S(17): 0.2
O75674	EATNTTSEPSAPSQDLLDLSPsPR	1	S22(Phospho)	T(3): 0.0; T(5): 0.0; T(6): 0.0; S(7): 0.0; S(10): 0.0; S(13): 0.0; S(20): 0.8; S(22): 99.2
Q92882	TLsNAEDYLDDEDsD	2	S3(Phospho); S14(Phospho)	T(1): 49.9; S(3): 49.9; Y(8): 0.1; S(14): 100.0
Q8TBE7	KQDYQEILDsPIK	1	S10(Phospho)	Y(4): 0.0; S(10): 100.0
Q86VP1	DQDKTDTLEHELRR	1		
Q8IYB3	RRsPsPAPPPR	3	S3(Phospho); S5(Phospho)	S(3): 100.0; S(5): 100.0
Q9H3Q1	AGPDLPSLPSHALEDEGWAAAAPsPGsAR	1	S24(Phospho); S27(Phospho)	S(7): 0.0; S(10): 0.0; S(24): 100.0; S(27): 100.0
P49411	DKPHVNVGTIGHVDHGK	2		
O00410	NLIDEDGNNQWPEGLK	1		
P98174	SLsLDPGQSLEPHPEGPQR	2	S3(Phospho)	S(1): 11.3; S(3): 88.7; S(9): 0.0
P27816	SKVGsTENIK	1	S5(Phospho)	S(1): 1.0; S(5): 98.1; T(6): 1.0
P04406	LISWYDNEFGYSNR	1		
P15311	RKEDEVEEWQHR	1		
Q96JC9	tSPLKDNPsPEPQLDDIKR	2	T1(Phospho); S9(Phospho)	T(1): 50.0; S(2): 50.0; S(9): 100.0
Q9H4L5	QDsWEVVEGLR	1	S3(Phospho)	S(3): 100.0
O14737	KVMDsDEDDDY	1	S5(Phospho)	S(5): 100.0; Y(11): 0.0
Q13813	NNHHEENISSK	4		
Q9UNY4	KQEEGAEIQcEAETGGTHKR	1	C10(Carbamidomethyl)	
O75962	KGAANASGsSPDAPAKDAR	2	S9(Phospho)	S(7): 0.3; S(9): 88.0; S(10): 11.8
Q9UQ35	ScFESsPDPELK	2	C2(Carbamidomethyl); S6(Phospho)	S(1): 0.0; S(5): 6.5; S(6): 93.5
Q9Y490	DHFGLEGDEESTMLEDSVSPK	1		
Q9NQT8	GRWEsQQDVSQTTVSR	1	S5(Phospho)	S(5): 100.0; S(10): 0.0; T(12): 0.0; T(13): 0.0; S(15): 0.0
Q8N350	HAsLDGASPYFK	1	S3(Phospho)	S(3): 99.9; S(8): 0.0; Y(10): 0.0
P78559	GLDsGAEtEEEKDTWEEKK	1	S4(Phospho); T8(Phospho)	S(4): 100.0; T(8): 100.0; T(14): 0.0
P78559	ADsKEsLKATTK	1	S3(Phospho); S6(Phospho)	S(3): 100.0; S(6): 100.0; T(10): 0.0; T(11): 0.0
Q8WWI1	EGFEsDTDSEFTFK	1	S5(Phospho)	S(5): 87.7; T(7): 12.0; S(9): 0.3; T(12): 0.0
P12268	GMGsLDAMDK	1	S4(Phospho)	S(4): 100.0
Q96CW6	EFGYDsPHDLDsD	1	S6(Phospho); S12(Phospho)	Y(4): 0.1; S(6): 99.9; S(12): 100.0
P46821	KESKEETPEVTK	1		
Q9NQT8	SIsSPNVNR	2	S3(Phospho)	S(1): 1.1; S(3): 89.4; S(4): 9.4
Q07157	ATLLNVPDLSDsIHsANASER	3	S12(Phospho); S15(Phospho)	T(2): 0.0; S(10): 27.3; S(12): 83.4; S(15): 85.6; S(19): 3.7
Q9UI30	GIPNMLLSEEETEs	5	S14(Phospho)	S(8): 0.8; T(12): 15.1; S(14): 84.1
Q15185	DWEDDsDEDMSNFDr	2	S6(Phospho); C-Term(Oxidation)	S(6): 99.1; S(11): 0.9
Q9UBB9	EEATYGVWAERDsDDERPSFGGK	1	S13(Phospho)	T(4): 0.0; Y(5): 0.0; S(13): 100.0; S(19): 0.0
Q8IWS0	TAHNSEAADLEESFNEHELEPsSPK	1	S22(Phospho)	T(1): 0.0; S(5): 0.0; S(13): 0.0; S(22): 15.3; S(23): 84.7
P21980	DLYLENPEIK	1		
P18124	TTHFVEGGDAGNREDQINR	2		
P23634	DAEGLDEIDHAEMELR	1		
P46821	RESVAsGDDRAEEDMDEAIEK	2	S6(Phospho)	S(3): 0.3; S(6): 99.7
P78559	ESSEKPPEKPAKPER	2		
P09497	VTEQEWR	1		
Q3V6T2	SSsQENLLDEVMk	5	S3(Phospho); C-Term(Oxidation)	S(1): 5.4; S(2): 47.3; S(3): 47.3
Q96AT1	IQPQPPDEDGDHsDKEDEQPQVVVLK	2	S13(Phospho)	S(13): 100.0
P67936	YSEKEDKYEEEIK	2		
Q9UQE7	NKKDELQSER	1		
P49023	QKsAEPsPTVMsTSLGSNLSELDR	1	S3(Phospho); S7(Phospho); S12(Phospho)	S(3): 97.9; S(7): 51.0; T(9): 51.0; S(12): 41.7; T(13): 41.7; S(14): 8.3; S(17): 8.3; S(20): 0.1
O94929	ASsPGYIDSPTYSR	1	S3(Phospho)	S(2): 9.4; S(3): 90.6; Y(6): 0.0; S(9): 0.0; T(11): 0.0; Y(12): 0.0; S(13): 0.0
Q13813	sLQQLAEER	1	S1(Phospho)	S(1): 100.0
Q8N6T3	VsGQPQSVTASSDK	1	S2(Phospho)	S(2): 100.0; S(7): 0.0; T(9): 0.0; S(11): 0.0; S(12): 0.0
P51858	AGDLLEDsPK	1	S8(Phospho)	S(8): 100.0
O00401	VIyDFIEK	1	Y3(Phospho)	Y(3): 100.0
Q92622	SHsDTSIASR	1	S3(Phospho)	S(1): 1.1; S(3): 32.6; T(5): 32.6; S(6): 32.6; S(9): 1.1
Q9NUJ3	VQRPHsSPPR	1	S6(Phospho)	S(6): 99.2; S(7): 0.8
P21333	DVDIIDHHDNTYTVK	1		
O14639	RSsGREEDDEELLR	1	S3(Phospho)	S(2): 0.8; S(3): 99.2
P08670	DNLAEDIMR	1		
P21333	DLAEDAPWK	1		
P27816	VGsTENIK	1	S3(Phospho)	S(3): 99.1; T(4): 0.9
Q8WXF7	DRNsWGGFSEK	2	S4(Phospho)	S(4): 100.0; S(9): 0.0
P26038	QRIDEFEsM	1	S8(Phospho)	S(8): 100.0
P27824	KDDTDDEIAKYDGK	1		
Q03001	QKSFsEDVISHK	1	S5(Phospho)	S(3): 50.0; S(5): 50.0; S(10): 0.0
Q12888	STPFIVPSsPTEQEGR	2	S9(Phospho)	S(1): 0.0; T(2): 0.0; S(8): 1.8; S(9): 86.4; T(11): 11.8
Q15149	TSsEDNLYLAVLR	1	S3(Phospho)	T(1): 1.9; S(2): 1.9; S(3): 12.0; Y(8): 84.2
P05787;P08670	LLEGEESR	1		
Q15121	YKDIIRQPsEEEIIK	1	S9(Phospho)	Y(1): 0.0; S(9): 100.0
Q0JRZ9	HsPVQMNR	1	S2(Phospho)	S(2): 100.0

Q6PKG0	sLPTTVPESPNYR	1	S1(Phospho)	S(1): 100.0; T(4): 0.0; T(5): 0.0; S(9): 0.0; Y(12): 0.0
P04083	SEIDMNDIK	1		
Q08AD1	SIsNEGLTLNNSHVSK	2	S3(Phospho)	S(1): 1.4; S(3): 98.6; T(8): 0.0; S(12): 0.0; S(15): 0.0
O43741	DLSSsPPGPYGQEMYAFR	3	S5(Phospho)	S(3): 7.7; S(4): 46.1; S(5): 46.1; Y(10): 0.0; Y(15): 0.0
P17677	QVEKNDDDQKIEQDGIKPEDK	1		
P46821	EEcPRPMsIsPPDFSPK	1	C3(Carbamidomethyl); S8(Phospho); S10(Phospho)	S(8): 100.0; S(10): 100.0; S(15): 0.0
Q9UBF8	tASNPKVENEDEPVR	2	T1(Phospho)	T(1): 50.0; S(3): 50.0
P18669	HGESAWNLENR	1		
P48634	LKFsDEEDGRDsDEEGAEGHR	2	S4(Phospho); S12(Phospho)	S(4): 100.0; S(12): 100.0
Q9ULU8	AGGGRPSsPSPSVVSEKEKEELER	1	S8(Phospho)	S(7): 32.5; S(8): 32.5; S(10): 32.5; S(12): 2.2; S(15): 0.2
Q9H2H9	RSLtNSHLEK	2	T4(Phospho)	S(2): 0.7; T(4): 99.2; S(6): 0.1
Q02952	QKEKEDAQEVELQEGK	1		
Q9P016	NNPHYDPSSKEDNPK	1		
P62753	RLssLRASTSK	1	S3(Phospho); S4(Phospho)	S(3): 99.8; S(4): 98.5; S(8): 1.5; T(9): 0.2; S(10): 0.0
Q14315	LGsFGSITR	1	S3(Phospho)	S(3): 99.1; S(6): 0.7; T(8): 0.2
Q9BST9	RPsDSGPPAER	2	S3(Phospho)	S(3): 99.7; S(5): 0.3
Q8IYB3	GTEKREsPsPAPKPR	1	S7(Phospho); S9(Phospho)	T(2): 1.0; S(7): 99.0; S(9): 100.0
Q8IYB3	RRsPsPPPTR	2	S3(Phospho); S5(Phospho)	S(3): 100.0; S(5): 100.0; T(9): 0.0
Q14195	GMYDGPVFDLTTtPK	2	T13(Phospho)	Y(3): 0.0; T(11): 0.2; T(12): 11.1; T(13): 88.7
P00558	DcVGPEVEK	1	C2(Carbamidomethyl)	
Q99460	DTSEDIEELVEPVAAHGPK	1		
Q9UQN3	ATIsDEEIER	1	S4(Phospho)	T(2): 0.0; S(4): 100.0
P51572	LDVGNAEVKLEEENR	1		
P53396	TAsFSESRADEVAPAK	3	S3(Phospho)	T(1): 0.2; S(3): 99.8; S(5): 0.0; S(7): 0.0
P06733	KLNVTEQEK	1		
P62328	NPLPSKETIEQEKQAGES	1		
P61978	DYDDMsPR	2	S6(Phospho)	Y(2): 0.0; S(6): 100.0
Q04637	SFsKEVEER	2	S3(Phospho)	S(1): 0.0; S(3): 100.0
Q6PKG0	sLPTTVPEsPNYR	3	S1(Phospho); S9(Phospho)	S(1): 99.9; T(4): 0.1; T(5): 0.3; S(9): 98.1; Y(12): 1.6
P07355	DLYDAGVK	1		
Q5T9C2	SSsLSDLTHR	1	S3(Phospho)	S(1): 0.0; S(2): 0.0; S(3): 100.0; S(5): 0.0; T(8): 0.0
Q96RU3	TVsDNsLSNSRGEGKPDLK	1	S3(Phospho); S6(Phospho)	T(1): 10.0; S(3): 90.0; S(6): 98.7; S(8): 1.3; S(10): 0.0
Q32MZ4	IDGATQSsPAEPK	1	S8(Phospho)	T(5): 0.3; S(7): 11.5; S(8): 88.3
Q96B23	RDSsEsQLASTESDKPTTGR	1	S4(Phospho); S6(Phospho)	S(3): 99.0; S(4): 50.5; S(6): 50.5; S(10): 0.0; T(11): 0.0; S(13): 0.0; T(17): 0.0; T(18): 0.0
Q8NC51	SEEAHAEDSVMDHHFR	1		
Q14152	ESRPSEEREWDREK	1		
Q05519	DYDEEEQGYDsEKEKK	1	S11(Phospho)	Y(2): 0.0; Y(9): 2.1; S(11): 97.9
O75347	ILENEKDLEEAEEYKEAR	1		
O95817	SsTPLHsPSPIR	1	S2(Phospho); S7(Phospho)	S(1): 7.8; S(2): 7.8; T(3): 84.3; S(7): 99.0; S(9): 1.0
P46821	EVPSKEEPsPVKAEVAEK	3	S9(Phospho)	S(4): 0.0; S(9): 100.0
O00571	DREEALHQFR	1		
P42677	DLLHPSPEEEK	1		
P36507	KLEELELDEQQKK	1		
P07900	KHLEINPDHSIIETLR	1		
A6ND36	AKsVDEIAK	1	S3(Phospho)	S(3): 100.0
Q96FS4	AHsHEEASRPAATSTR	1	S3(Phospho)	S(3): 100.0; S(8): 0.0; T(13): 0.0; S(14): 0.0; T(15): 0.0
O60565	GTAMPGEEVLESsQEALHVTER	2	S13(Phospho)	T(2): 0.0; S(12): 14.7; S(13): 84.8; T(20): 0.6
P27824	AEEDEILNRsPR	4	S10(Phospho)	S(10): 100.0
Q12874	DLYDDKDGLRK	1		
O15027	FTGsFDDDPDPHRDPYGEEVDRR	2	S4(Phospho)	T(2): 15.3; S(4): 84.7; Y(16): 0.0
Q9NSY0	EREDEsEDESDILEESPcGR	2	S6(Phospho); C18(Carbamidomethyl)	S(6): 90.5; S(10): 9.5; S(16): 0.0
P06493	IGEGtyGVVYK	1	T5(Phospho); Y6(Phospho)	T(5): 100.0; Y(6): 100.0; Y(10): 0.0
Q14671	RDsLTGSSDLYK	1	S3(Phospho)	S(3): 99.9; T(5): 0.1; S(7): 0.0; S(8): 0.0; Y(11): 0.0
Q9Y608	RGsGDTsSLIDPDTSLSELR	1	S3(Phospho); S7(Phospho)	S(3): 12.6; T(6): 82.4; S(7): 52.5; S(8): 52.5; T(14): 0.0; S(15): 0.0; S(17): 0.0
Q9NQX3	EVHDELEDLPsPPPPLsPPPTTSPHK	1	S11(Phospho); S17(Phospho)	S(11): 99.5; S(17): 97.0; T(21): 1.5; T(22): 1.5; S(23): 0.5
Q03001	LEEVKPVVEVHHQsEQETSVR	1	S14(Phospho)	S(14): 99.0; T(18): 0.8; S(19): 0.2
Q92597	sRTAsGsSVTSLDGTR	4	S1(Phospho); S5(Phospho); S7(Phospho)	S(1): 85.5; T(3): 17.1; S(5): 96.0; S(7): 3.3; S(8): 3.3; T(10): 14.0; S(11): 80.7; T(15): 0.0
P46821	SPSLsPsPPSPLEK	3	S5(Phospho); S7(Phospho)	S(1): 0.0; S(3): 0.0; S(5): 98.1; S(7): 2.1; S(10): 99.7
P11137	VDHGAEIItQSPGRSsVAsPR	3	T9(Phospho); S16(Phospho); S19(Phospho)	T(9): 15.3; S(11): 84.6; S(15): 15.4; S(16): 84.7; S(19): 100.0
Q13459	RTsFSTSDVSK	1	S3(Phospho)	T(2): 0.8; S(3): 76.8; S(5): 7.4; T(6): 7.4; S(7): 7.4; S(10): 0.0
Q13200	DAGDKDKEQELSEEDK	1		
P35579	NAEQYKDQADK	1		
Q86UU1	KNsITEIsDNEDDLLEYHR	1	S3(Phospho); S8(Phospho)	S(3): 99.9; T(5): 9.5; S(8): 90.6; Y(17): 0.0
O14617	NTETsKSPEKDVPMVEK	3	S5(Phospho)	T(2): 0.0; T(4): 0.0; S(5): 1.1; S(7): 98.9
P27816	KKPcSETSQIEDTPSSKPTLLANGGHGVEGSDTTGsPTEFLEEK	1	C4(Carbamidomethyl); S36(Phospho)	S(5): 0.1; T(7): 0.0; S(8): 0.0; T(13): 0.1; S(15): 0.6; S(16): 0.6; T(19): 0.2; S(31): 0.2; T(33): 1.3; T(34): 3.3; S(36): 69.6; T(38): 24.0
Q8NE71	GGNVFAALIQDQsEEEEEEEKHPPKPAKPEK	2	S13(Phospho)	S(13): 100.0
Q9UDY2	RAASsDQLRDNsPPPAFKPEPPK	3	S5(Phospho); S12(Phospho)	S(4): 54.9; S(5): 54.9; S(12): 90.2
Q96JG6	SAYQEYDsDSDVPEELKR	2	S8(Phospho)	S(1): 0.0; Y(3): 0.0; Y(6): 91.3; S(8): 8.6; S(10): 0.0
Q13501	sRLTPVsPESSSTEEK	2	S1(Phospho); S7(Phospho)	S(1): 1.8; T(4): 98.2; S(7): 97.9; S(10): 1.8; S(11): 0.3; S(12): 0.0; T(13): 0.0
O60313	GKEHDDIFDK	1		
Q9UPZ3	RGsQsDEDScSLHSQTLSEDER	1	S3(Phospho); S5(Phospho); C10(Carbamidomethyl)	S(3): 3.6; S(5): 50.8; S(9): 49.2; S(11): 96.0; S(14): 0.2; T(16): 0.2; S(18): 0.0
Q9H2G2	VDEDsAEDTQsNDGKEVVEVGQK	1	S5(Phospho); S11(Phospho)	S(5): 3.2; T(9): 97.0; S(11): 99.9
Q7L9B9	FctELNQPtLPNIRKWK	1	C2(Carbamidomethyl); T3(Phospho); T9(Phospho)	T(3): 100.0; T(9): 100.0
Q8IVF2	VDLKsPQVDIK	1	S5(Phospho)	S(5): 100.0
	VAPDEHPILLTEAPLNPK	1		
P02545	sVGGSGGGSFGDNLVTR	2	S1(Phospho)	S(1): 97.5; S(5): 2.5; S(9): 0.0; T(16): 0.0
Q96JM3	KPGPPLsPEIRsPAGsPELR	1	S7(Phospho); S12(Phospho); S16(Phospho)	S(7): 100.0; S(12): 100.0; S(16): 100.0
Q8TB03	LDSQEPGRQtPDR	1	T10(Phospho)	S(3): 1.9; T(10): 98.1
Q15029	DLDEMDDDDDDDDVGDHDDDHPGMEVVLHEDKK	1		
Q96EV2	VKPAsPVAQPK	1	S5(Phospho)	S(5): 100.0
P40818	RSYsSPDITQAIQEEEKR	3	S4(Phospho)	S(2): 1.9; Y(3): 11.4; S(4): 75.1; S(5): 11.4; T(9): 0.3
P14625	TDDEVVQREEEAIQLDGLNASQIR	1		
	HEERQDEHGFISr	1	C-Term(Oxidation)	
P23396	DEILPTtPISEQK	1	T7(Phospho)	T(6): 47.6; T(7): 47.6; S(10): 4.9
Q96C19	ADLNQGIGEPQsPSRR	1	S12(Phospho)	S(12): 89.0; S(14): 11.0
O95677	NNPsPPPDSDLER	2	S4(Phospho)	S(4): 100.0; S(9): 0.0
P00519	ScsAScVPHGAK	1	C2(Carbamidomethyl); S3(Phospho); C6(Carbamidomethyl)	S(1): 1.8; S(3): 86.6; S(5): 11.6
Q01082	RPPsPEPSTK	3	S4(Phospho)	S(4): 100.0; S(8): 0.0; T(9): 0.0

Q9UQ35	AQtPPGPSLSGSKsPcPQEK	1	T3(Phospho); S14(Phospho); C16(Carbamidomethyl)	T(3): 100.0; S(8): 0.0; S(10): 0.0; S(12): 0.2; S(14): 99.8
Q14247	AKTQtPPVSPAPQPTEER	2	T5(Phospho)	T(3): 0.8; T(5): 99.1; S(9): 0.1; T(15): 0.0
O43237	DFQDYMEPEEGcQGsPQRR	1	C12(Carbamidomethyl); S15(Phospho)	Y(5): 0.0; S(15): 100.0
Q9NTZ6	sRsPHEAGFcVYLK	1	S1(Phospho); S3(Phospho); C10(Carbamidomethyl)	S(1): 100.0; S(3): 100.0; Y(12): 0.0
Q9NX40	RSsPPGHYYQK	1	S3(Phospho)	S(2): 0.1; S(3): 99.9; Y(8): 0.0; Y(9): 0.0
Q9NPI6	KASsPsPLTIGTPESQR	1	S4(Phospho); S6(Phospho)	S(3): 76.3; S(4): 76.3; S(6): 32.5; T(9): 14.9; T(12): 0.0; S(15): 0.0
Q13177	YLsFTPPEK	1	S3(Phospho)	Y(1): 0.6; S(3): 99.4; T(5): 0.1
Q96KC8	DFDIAEQNESsDEEsLRK	2	S11(Phospho); S15(Phospho)	S(10): 10.8; S(11): 90.3; S(15): 98.9
P42566	sSPELLPSGVTDENEVTTAVTEK	2	S1(Phospho)	S(1): 50.0; S(2): 50.0; S(8): 0.1; T(11): 0.0; T(17): 0.0; T(18): 0.0; T(21): 0.0
P05386	KEEsEEsDDDMGFGLFd	1	S4(Phospho); S7(Phospho); C-Term(Oxidation)	S(4): 100.0; S(7): 100.0
P17812	SGSSsPDSEITELK	1	S5(Phospho)	S(1): 9.3; S(3): 9.3; S(4): 9.3; S(5): 62.9; S(8): 9.3; T(11): 0.1
P13693	DGLEMEKc	1	C8(Carbamidomethyl)	
P49585	AAAYDIsEDEED	1	S7(Phospho)	Y(4): 0.0; S(7): 100.0
Q92574	GGGGSSSSSSELsTPEKPPHQR	2	S13(Phospho)	S(5): 0.0; S(6): 0.1; S(7): 0.8; S(8): 32.7; S(9): 32.7; S(10): 32.7; S(13): 0.8; T(14): 0.1
Q8TER5	IQQHVGEEAsPR	1	S10(Phospho)	S(10): 100.0
P13639	AGETRFtDTR	1	T7(Phospho)	T(4): 0.0; T(7): 100.0; T(9): 0.0
Q8N3F8	KPsPAASPATK	2	S3(Phospho)	S(3): 87.8; S(7): 12.2; T(10): 0.1
Q0JRZ9	NLsNEELTK	1	S3(Phospho)	S(3): 100.0; T(8): 0.0
Q8WUY3	NLSLtDFVGDESPsPER	1	T5(Phospho); S14(Phospho)	S(3): 86.0; T(5): 14.0; S(12): 2.9; S(14): 97.1
P61244	NHTHQQDIDDLKR	1		
O95819	RPHPQHSQQPPPPQQER	1		
Q9Y490	GAAAHPDSEEQQQR	1		
O60343	LGsVDSFER	1	S3(Phospho)	S(3): 100.0; S(6): 0.0
Q8IU81	KAsPEPEGEAAGK	1	S3(Phospho)	S(3): 100.0
Q5VUA4	sSDPHRLESR	1	N-Term(Acetyl)	
Q92973	DNEEKDSAFR	1		
Q9UJU6	QLtQPETHFGR	1	T3(Phospho)	T(3): 99.9; T(7): 0.1
Q8IYB3	SRVsVsPGR	1	S4(Phospho); S6(Phospho)	S(1): 0.0; S(4): 100.0; S(6): 100.0
Q5VTL8	RRsQSIEQESQEK	1	S3(Phospho)	S(3): 99.8; S(5): 0.2; S(10): 0.0
O75688	DSELDKHLESR	1		
P38646	MEEFKDQLPADEcNK	1	C13(Carbamidomethyl)	
Q9H1E3	DDSHSAEDsEDEKEDHKNVR	2	S9(Phospho)	S(3): 1.0; S(5): 1.0; S(9): 97.9
Q5VT25	SLsLESTDR	1	S3(Phospho)	S(1): 0.6; S(3): 47.2; S(6): 47.2; T(7): 5.0
Q00587	AsWESLDEEWR	1	S2(Phospho)	S(2): 97.7; S(5): 2.3
P00338	DLADELALVDVIEDK	1		
Q9C0C2	DsLGAYASQDANEQGQDLGKR	2	S2(Phospho)	S(2): 99.6; Y(6): 0.3; S(8): 0.1
Q9NXR1	RPsSTSVPLGDK	2	S3(Phospho)	S(3): 77.3; S(4): 10.6; T(5): 10.6; S(6): 1.6
Q9H2K0	DHGNDKESNVLHQ	1		
Q8IX12	KLTDTSKDEENHEESESLQEDMLGNR	1		
Q16543	SMVNTKPEKTEEDSEEVREQK	2		
P16949	RKsHEAEVLK	1	S3(Phospho)	S(3): 100.0
Q9BVG9	RDAGGPRPEsPVPAGR	1	S10(Phospho)	S(10): 100.0
Q9Y4F5	GHKHEDGTQsDSEDPLAK	1	S10(Phospho)	T(8): 0.4; S(10): 97.4; S(12): 2.2
P46821	VSPSKSPSLsPsPPSPLEK	2	S10(Phospho); S12(Phospho)	S(2): 1.1; S(4): 46.0; S(6): 46.0; S(8): 6.9; S(10): 0.0; S(12): 2.4; S(15): 97.6
Q96JM3	LAPVPsPEPQKPAPVsPESVK	1	S6(Phospho); S16(Phospho)	S(6): 100.0; S(16): 96.3; S(19): 3.7
P09651	DYFEQYGK	1		
Q07666	SGsMDPSGAHPSVR	2	S3(Phospho)	S(1): 50.0; S(3): 50.0; S(7): 0.0; S(12): 0.0
Q8NHG8	DRPVGGsPGGPR	1	S7(Phospho)	S(7): 100.0
Q9P206	DQsPPPsPPPSYHPPPPPTK	3	S3(Phospho); S7(Phospho)	S(3): 100.0; S(7): 99.6; S(11): 0.3; Y(12): 0.1; T(19): 0.0
P27816	KVsYSHIQSK	3	S3(Phospho)	S(3): 4.5; Y(4): 47.5; S(5): 47.5; S(9): 0.5
P06703	LMEDLDR	1		
Q9H4G0	SLsPIIGK	1	S3(Phospho)	S(1): 7.7; S(3): 92.3
P63267;P60709	DLTDYLMK	1		
P16949	DKHIEEVRK	1		
Q8WWI1	GISsLPR	1	S4(Phospho)	S(3): 0.0; S(4): 100.0
P07814	FDDTNPEKEKEDFEK	2		
P26368	GAKEEHGGLIRsPR	1	S12(Phospho)	S(12): 100.0
Q9BW71	SLKEsEQEsEEEILAQK	1	S5(Phospho); S9(Phospho)	S(1): 4.2; S(5): 96.6; S(9): 99.2
P11717	ALSSLHGDDQDsEDEVLTIPEVK	1	S12(Phospho)	S(3): 2.2; S(4): 2.2; S(12): 95.6; T(18): 0.0
Q6PKG0	KNTFTAWsDEEsDYEIDDRDVNK	2	S8(Phospho); S12(Phospho)	T(3): 1.8; T(5): 3.0; S(8): 92.1; S(12): 93.3; Y(14): 9.8
P18887	AIGSTSKPQEsPK	1	S11(Phospho)	S(4): 0.0; T(5): 0.0; S(6): 0.0; S(11): 100.0
Q96QG7	RQLAELETEDGMQEsP	1	S15(Phospho)	T(8): 0.0; S(15): 100.0
O00178	LHGGFDsDcsEDGEALNGEPELDLTSK	2	S7(Phospho); C9(Carbamidomethyl); S10(Phospho)	S(7): 100.0; S(10): 100.0; T(25): 0.0; S(26): 0.0
Q96N67	SLSNSNPDISGtPTsPDDEVR	2	T12(Phospho); S15(Phospho)	S(1): 3.1; S(3): 3.1; S(5): 93.7; S(10): 0.2; T(12): 0.3; T(14): 49.8; S(15): 49.8
Q969V6	FGSTGsTPPVsPTPSER	1	S6(Phospho); S11(Phospho)	S(3): 0.1; T(4): 0.5; S(6): 13.9; T(7): 85.4; S(11): 46.2; T(13): 46.2; S(15): 7.6
O15085	sLENPtPPFTPK	2	S1(Phospho); T6(Phospho)	S(1): 100.0; T(6): 91.9; T(10): 8.1
P04049	RAsDDGKLTDPSK	2	S3(Phospho)	S(3): 99.6; T(9): 0.3; S(12): 0.0
Q00653	DKLPSTEVKEDSAYGSQSVEQEAEK	2		
Q99958	VETLSPEsALQGsPR	1	S8(Phospho); S13(Phospho)	T(3): 0.0; S(5): 99.9; S(8): 1.6; S(13): 98.5
Q6WCQ1	AEEQQLPPPLsPPsPSTPNHR	1	S11(Phospho); S14(Phospho)	S(11): 99.8; S(14): 85.1; S(16): 12.9; T(17): 2.1
Q86VQ1	GPsPsSPTPPAAAAPAEQAPR	2	S3(Phospho); S5(Phospho)	S(3): 79.9; S(5): 79.9; S(6): 29.8; T(8): 10.4
Q96F86	SQDVAVsPQQQQcsKSYVDR	1	S7(Phospho); C13(Carbamidomethyl); S14(Phospho)	S(1): 0.0; S(7): 100.0; S(14): 89.0; S(16): 9.8; Y(17): 1.2
Q9P2B4	DLsPTLIDNSAAK	1	S3(Phospho)	S(3): 99.9; T(5): 0.1; S(10): 0.0
Q16555	TVTPAssAKTsPAK	1	S6(Phospho); S7(Phospho); S11(Phospho)	T(1): 50.0; T(3): 50.0; S(6): 8.3; S(7): 91.6; T(10): 0.2; S(11): 99.9
P33176	SQEVEDKTKEYELLSDELNQK	1		
Q8N684	DLLHNEDRHDDYFQER	1		
P78559	VPSAPGQEsPIPDPK	1	S9(Phospho)	S(3): 0.0; S(9): 100.0
Q9BW71	KQAREEsEEsEAEPVQR	2	S7(Phospho); S10(Phospho)	S(7): 100.0; S(10): 100.0
Q9ULL5	NLETLPSFssDEEDSVAK	1	S9(Phospho); S10(Phospho)	T(4): 0.4; S(7): 23.7; S(9): 87.9; S(10): 87.9; S(15): 0.2
Q86UX6	RGSsAAAsPGSPPPGR	1	S4(Phospho); S8(Phospho)	S(3): 90.4; S(4): 9.6; S(8): 98.8; S(11): 1.2
Q8WWM7	EKEVDGLLTSEPMGsPVSSK	1	S15(Phospho)	T(9): 0.0; S(10): 0.0; S(15): 46.3; S(18): 46.3; S(19): 7.4
Q9UPU5	VSDQNsPVLPK	1	S6(Phospho)	S(2): 0.0; S(6): 100.0
Q765P7	AGsEEcVFYTDETASPLAPDLAK	1	S3(Phospho); C6(Carbamidomethyl)	S(3): 48.3; Y(9): 48.3; T(10): 1.6; T(13): 1.6; S(15): 0.1
P11717	LVsFHDDsDEDLLHI	1	S3(Phospho); S8(Phospho)	S(3): 100.0; S(8): 100.0
O75122	DKsFDDEESVDGNRPSSAASAFK	1	S3(Phospho)	S(3): 100.0; S(9): 0.0; S(16): 0.0; S(17): 0.0; S(20): 0.0
Q13404	LPQPPEGQcYsN	1	C9(Carbamidomethyl); S11(Phospho)	Y(10): 0.7; S(11): 99.3
Q8IXQ3	RRDsGDNSAPSGQER	1	S4(Phospho)	S(4): 100.0; S(8): 0.0; S(11): 0.0
Q05682	RGsIGENQIK	1	S3(Phospho)	S(3): 100.0

P08238	EREKEIsDDEAEEEK	1	S7(Phospho)	S(7): 100.0
Q07954	HsLASTDEKR	1	S2(Phospho)	S(2): 89.8; S(5): 10.0; T(6): 0.2
Q9H4G0	SLDGAEFSRPASVsENHDAGPDGDKR	1	S14(Phospho)	S(1): 3.2; S(8): 32.3; S(12): 32.3; S(14): 32.3
	DKRPEGYNLKDEEGR	1		
Q13443	HVsPVTPPR	1	S3(Phospho)	S(3): 99.9; T(6): 0.1
P04406	LEKPAKYDDIKK	1		
Q8N1F8	AKNsPPQAPSTR	1	S4(Phospho)	S(4): 100.0; S(10): 0.0; T(11): 0.0
Q3YEC7	LDRsFLEDTTPARDEK	1	S4(Phospho)	S(4): 100.0; T(9): 0.0; T(10): 0.0
P46379	AsPEPQRENAsPAPGTTAEEAMSR	1	S2(Phospho); S11(Phospho)	S(2): 99.9; S(11): 4.3; T(16): 4.2; T(17): 4.2; S(23): 87.4
P46821	SDIsPLtPR	2	S4(Phospho); T7(Phospho)	S(1): 0.0; S(4): 100.0; T(7): 100.0
Q9Y5A7	NYHSGNDVEAYEYLNK	1		
Q8IYB3	RRtAsPPPPPK	1	T3(Phospho); S5(Phospho)	T(3): 100.0; S(5): 100.0
Q9P2P5	SHsAGEVGEDSR	1	S3(Phospho)	S(1): 12.5; S(3): 87.5; S(11): 0.0
Q05682	NGQISSEEPKQEEEREQGSDEISHHEK	1		
Q9BVG4	GADsGEEKEEGINREDK	1	S4(Phospho)	S(4): 100.0
Q9H7D7	RLsQSDEDVIR	1	S3(Phospho)	S(3): 50.0; S(5): 50.0
Q9H2K0	DTLNKDHGNDKESNVLHQ	1		
Q92615	SPsPAHLPDDPK	3	S3(Phospho)	S(1): 0.7; S(3): 99.3
Q6PKG0	NTFTAWsDEEsDYEIDDRDVNK	1	S7(Phospho); S11(Phospho)	T(2): 0.0; T(4): 0.1; S(7): 99.2; S(11): 84.0; Y(13): 16.8
Q9BRS8	QAEMGTQEKsPGTsPLLSR	1	S10(Phospho); S14(Phospho)	T(6): 86.6; S(10): 13.4; T(13): 13.3; S(14): 86.2; S(18): 0.4
Q08AD1	SEsVEGFLsPSR	1	S3(Phospho); S9(Phospho)	S(1): 12.3; S(3): 87.7; S(9): 87.7; S(11): 12.3
Q14160	NsLESISSIDR	1	S2(Phospho)	S(2): 100.0; S(5): 0.0; S(7): 0.0; S(8): 0.0
Q16790	DKEGDDQSHWR	1		
Q8WWI1	GGREGFEsDTDSEFTFK	1	S8(Phospho)	S(8): 99.6; T(10): 0.4; S(12): 0.0; T(15): 0.0
Q12802	FLSHsTDSLNK	2	S5(Phospho)	S(3): 0.0; S(5): 49.9; T(6): 49.9; S(8): 0.2
P07237	NFEDVAFDEK	1		
Q02952	SPPsPVER	3	S4(Phospho)	S(1): 0.0; S(4): 100.0
Q6N075	HDAELRVPsPTEEPYAPEL	1	S9(Phospho)	S(9): 12.5; T(11): 87.5; Y(15): 0.0
Q9Y4B5	DASLHGLSQYNsL	1	S12(Phospho)	S(3): 0.0; S(8): 0.0; Y(10): 1.4; S(12): 98.6
Q8IYB3	REsPsPAPKPR	2	S3(Phospho); S5(Phospho)	S(3): 100.0; S(5): 100.0
Q13671	GsPATsPHLGR	1	S2(Phospho); S6(Phospho)	S(2): 98.2; T(5): 13.3; S(6): 88.4
O94804	LSEEAEcPNPStPSK	1	C7(Carbamidomethyl); T12(Phospho)	S(2): 0.0; S(11): 9.5; T(12): 89.4; S(14): 1.1
Q9H788	TLsSSAQEDIIR	1	S3(Phospho)	T(1): 0.1; S(3): 98.8; S(4): 1.0; S(5): 0.1
Q9NQC3	eTKLSAEPAPDFSDYSEMAK	1	N-Term(Acetyl)	
Q6P6C2	KSYESsEDcSEAAGSPAR	2	S6(Phospho); C9(Carbamidomethyl)	S(2): 1.6; Y(3): 10.1; S(5): 10.1; S(6): 68.0; S(10): 10.1; S(15): 0.0
Q09666	SNsFSDER	1	S3(Phospho)	S(1): 0.1; S(3): 92.3; S(5): 7.6
Q8TAQ2	KRSPsPsPTPEAK	1	S5(Phospho); S7(Phospho)	S(3): 93.9; S(5): 50.3; S(7): 49.7; T(9): 6.1
Q71U36	EIIDLVLDR	1		
Q8IVF2	LDLKsPK	1	S5(Phospho)	S(5): 100.0
Q86UU0	DRsVSVDSGEQR	1	S3(Phospho)	S(3): 100.0; S(5): 0.0; S(8): 0.0
Q06190	SSsVEEKPLSHR	1	S3(Phospho)	S(1): 0.1; S(2): 0.9; S(3): 99.0; S(10): 0.0
O94842	LSTTPsPTsSLHEDGVEDFRR	2	S6(Phospho); S9(Phospho)	S(2): 0.0; T(3): 0.0; T(4): 0.1; S(6): 15.2; T(8): 84.7; S(9): 84.7; S(10): 15.2
Q13263	LAsPsGSTSSGLEVVAPEGTSAPGGGPGTLDDSATIcR	4	S3(Phospho); S5(Phospho); C37(Carbamidomethyl)	S(3): 16.4; S(5): 16.4; S(7): 41.7; T(8): 41.7; S(9): 41.7; S(10): 41.7; T(20): 0.1; S(21): 0.1; T(29): 0.0; S(33): 0.0; T(35): 0.0
Q8IYB3	RRtPtPPPR	2	T3(Phospho); T5(Phospho)	T(3): 100.0; T(5): 100.0
Q15056	DKDTDKFK	2		
Q9Y217	ELLHSVHPEsPNLK	1	S10(Phospho)	S(5): 0.0; S(10): 100.0
Q9UPN6	DRDDRDFDFcR	1	C10(Carbamidomethyl)	
P33176	VHEMEKEHLNK	1		
P78559	ELVLssPEDLTQDFEEMKR	1	S5(Phospho); S6(Phospho)	S(5): 96.5; S(6): 84.2; T(11): 19.3
P51858	RAGDLLEDsPKRPK	2	S9(Phospho)	S(9): 100.0
Q04637	DKDDDEVFEKK	1		
Q6P996	GSDALSETsSVSHIEDLEK	2	S9(Phospho)	S(2): 0.1; S(6): 25.0; T(8): 25.0; S(9): 25.0; S(10): 25.0; S(12): 0.0
P06748	VDNDENEHQLSLR	1		
Q9BU76	RPAEATSSPTsPERPR	1	S11(Phospho)	T(6): 0.0; S(7): 0.0; S(8): 0.0; T(10): 1.1; S(11): 98.9
Q9BTU6	mDETsPLVsPER	1	N-Term(Acetyl); S5(Phospho); S9(Phospho)	T(4): 87.4; S(5): 12.6; S(9): 100.0
Q99613	QPLLLsEDEEDTKR	2	S6(Phospho)	S(6): 100.0; T(12): 0.0
P78559	RsPTPGKGPADR	2	S2(Phospho)	S(2): 90.5; T(4): 9.5
Q9Y4I1	tSSIADEGTYTLDSILR	2	T1(Phospho)	T(1): 33.3; S(2): 33.3; S(3): 33.3; T(9): 0.0; Y(10): 0.0; T(11): 0.0; S(14): 0.0
Q8IVF2	EKEDTDVADGcREtPTK	1	C11(Carbamidomethyl); T14(Phospho)	T(5): 0.0; T(14): 97.8; T(16): 2.2
Q9Y2V2	TFsATVR	1	S3(Phospho)	T(1): 0.9; S(3): 99.1; T(5): 0.0
P78559	sPFEIIsPPAsPPEMVGQr	1	S1(Phospho); S7(Phospho); S11(Phospho); C-Term(Oxidation)	S(1): 100.0; S(7): 100.0; S(11): 100.0
P49207	RLsYNTASNK	1	S3(Phospho)	S(3): 83.7; Y(4): 8.1; T(6): 8.1; S(8): 0.1
Q9Y2X3	HIKEEPLsEEEPcTsTAIASPEK	1	S8(Phospho); C13(Carbamidomethyl); S15(Phospho)	S(8): 100.0; T(14): 0.0; S(15): 0.0; T(16): 0.0; S(20): 100.0
P10451	QNLLAPQNAVssEETNDFKQETLPSK	1	S11(Phospho); S12(Phospho)	S(11): 97.6; S(12): 97.6; T(15): 3.1; T(22): 1.6; S(25): 0.2
Q9UQ35	THTTALAGRsPsPASGR	1	S10(Phospho); S12(Phospho)	T(1): 0.2; T(3): 0.1; T(4): 0.8; S(10): 83.7; S(12): 96.7; S(15): 18.5
P85037	SMVSPVPSPtGtISVPNScPAsPR	1	T10(Phospho); T12(Phospho); C19(Carbamidomethyl); S22(Phospho)	S(1): 0.7; S(4): 4.0; S(8): 93.7; T(10): 93.7; T(12): 5.9; S(14): 2.0; S(18): 0.4; S(22): 99.6
Q96JH7	GNsVEELEEMDSQDAEMTNTTEPMDHS	1	S3(Phospho)	S(3): 99.8; S(12): 0.1; T(18): 0.0; T(20): 0.0; T(21): 0.0; S(27): 0.0
Q96PD2	TDScssAQAQYDTPK	1	C4(Carbamidomethyl); S5(Phospho); S6(Phospho)	T(1): 51.0; S(3): 51.0; S(5): 49.0; S(6): 49.0; Y(11): 0.0; T(13): 0.0
Q9BUA3	NLDPDPEPPSPDsPTETFAAPAEVR	2	S13(Phospho)	S(10): 25.0; S(13): 25.0; T(15): 25.0; T(17): 25.0
P46821	DSISAVSSEKVSPSKsPsLSPSPPSPLEK	1	S16(Phospho); S18(Phospho)	S(2): 0.2; S(4): 0.9; S(7): 0.9; S(8): 3.8; S(12): 86.6; S(14): 3.8; S(16): 3.9; S(18): 3.0; S(20): 68.9; S(22): 14.0; S(25): 14.0
Q9NP74	SEASPHENTNHKsPHK	1	S13(Phospho)	S(1): 0.0; S(4): 0.0; T(9): 0.6; S(13): 99.4
P55040	SKscHDLSVL	1	S3(Phospho); C4(Carbamidomethyl)	S(1): 9.4; S(3): 90.6; S(8): 0.0
P46821	KEtPPKEVK	1	T3(Phospho)	T(3): 100.0
P23508	HIEQLTTAsEHcDLAIK	2	S9(Phospho); C12(Carbamidomethyl)	T(6): 0.0; T(7): 0.3; S(9): 99.7
Q96B23	SRsESETSTMAAK	1	S3(Phospho)	S(1): 0.3; S(3): 95.9; S(5): 0.3; T(7): 1.7; S(8): 1.7; T(9): 0.3
P49736	GNDPLTSsPGR	1	S8(Phospho)	T(6): 7.6; S(7): 0.7; S(8): 91.7
Q9P0K7	EAEADLSFDSYHSTQTDLGPSLGKPGETSPPDsK	1	S33(Phospho)	S(7): 0.0; S(10): 0.0; Y(11): 0.0; S(13): 0.0; T(14): 0.0; T(16): 0.0; S(21): 0.3; T(28): 6.5; S(29): 21.0; S(33): 72.1
P35579	DLQGRDEQSEEK	2		
Q99700	DSFIENSSSNcTSGSsKPNsPsISPSILSNTEHK	1	C11(Carbamidomethyl); S16(Phospho); S20(Phospho); S22(Phospho)	Too many isoforms
Q9H4G0	sLPELDRDKSDSDTEGLLFSR	1	S1(Phospho)	S(1): 98.5; S(10): 0.6; S(12): 0.3; T(14): 0.3; S(20): 0.2
O95786	DSQEKPKPVPDKENK	2		
Q8IYB3	RRtPsPPPR	3	T3(Phospho); S5(Phospho)	T(3): 100.0; S(5): 100.0
P35367	SGsNTGLDYIK	2	S3(Phospho)	S(1): 6.5; S(3): 93.5; T(5): 0.0; Y(9): 0.0
P61006	KLEGNSPQGsNQGVK	1	S10(Phospho)	S(6): 2.6; S(10): 97.4
Q15637	TGDLGIPPNPEDRsPsPEPIYNSEGKR	1	S14(Phospho); S16(Phospho)	T(1): 0.1; S(14): 97.6; S(16): 93.7; Y(21): 4.3; S(23): 4.3
Q9P227	SKscDDGLNTFRDEGR	1	S3(Phospho); C4(Carbamidomethyl)	S(1): 10.8; S(3): 89.0; T(10): 0.2
Q9BV36	SPQDPGDPVQYNRtTDEELsELEDR	1	T14(Phospho); S20(Phospho)	S(1): 0.0; Y(11): 70.0; T(14): 15.3; T(15): 15.3; S(20): 99.4
Q09666	KGDRsPEPGQTWTr	1	S5(Phospho); C-Term(Oxidation)	S(5): 100.0; T(11): 0.0; T(13): 0.0

Q96BY7	SYIsPHsSPSHTPTR	1	S4(Phospho); S7(Phospho)	S(1): 34.1; Y(2): 34.1; S(4): 35.9; S(7): 47.5; S(8): 47.5; S(10): 0.9; T(12): 0.0; T(14): 0.0
P46821	QGsPDQVsPVSEMTSTSLYQDKQEGK	1	S3(Phospho); S8(Phospho)	S(3): 99.9; S(8): 99.4; S(11): 0.2; T(14): 0.1; S(15): 0.1; T(16): 0.1; S(17): 0.1; Y(19): 0.1
P42167	AKtPVTLK	1	T3(Phospho)	T(3): 6.9; T(6): 93.1
Q9UPN3	RQGsFSEDVISHK	1	S4(Phospho)	S(4): 90.4; S(6): 9.6; S(11): 0.0
Q01813	sFAGNLNTYK	2	S1(Phospho)	S(1): 100.0; T(8): 0.0; Y(9): 0.0
Q3MII6	QAsLDGLQQLR	1	S3(Phospho)	S(3): 100.0
Q9UQ35	RRPsPQPsPR	1	S4(Phospho); S8(Phospho)	S(4): 100.0; S(8): 100.0
P53370	tENTTPITSRVARLLLyGyr	1	N-Term(Acetyl); Y17(Phospho); Y19(Phospho); C-Term(Oxidation)	T(1): 0.0; T(4): 0.0; T(5): 0.0; T(8): 0.0; S(9): 0.0; Y(17): 100.0; Y(19): 100.0
Q99549	GAEAFGDsEEDGEDVFEVEK	1	S8(Phospho)	S(8): 100.0
Q13247	ARsVsPPPKR	1	S3(Phospho); S5(Phospho)	S(3): 100.0; S(5): 100.0
Q9UJY1	TKDGYVEVSGKHEEK	1		
Q14204	TDsTSDGRPAWMR	1	S3(Phospho)	T(1): 0.1; S(3): 90.7; T(4): 9.0; S(5): 0.1
Q8NC51	SKsEEAHAEDSVMDHHFR	1	S3(Phospho)	S(1): 15.0; S(3): 84.9; S(11): 0.1
P78559	GELsPSFLNPPLPPSIDDR	3	S4(Phospho)	S(4): 96.7; S(6): 3.3; S(15): 0.0
Q5T5Y3	RPsEGPQPLVR	1	S3(Phospho)	S(3): 100.0
Q00613	GHTDTEGRPPsPPPTsTPEK	1	S11(Phospho); S16(Phospho)	T(3): 0.0; T(5): 10.7; S(11): 78.7; T(15): 11.4; S(16): 49.6; T(17): 49.6
Q5T5U3	SVsQERLEDSVLMK	2	S3(Phospho)	S(1): 50.0; S(3): 50.0; S(10): 0.0
Q9H1E3	DDsHsAEDsEDKEDHKNVR	1	S3(Phospho); S5(Phospho); S9(Phospho)	S(3): 100.0; S(5): 100.0; S(9): 100.0
Q5SW79	GNKHDDGTQsDSENAGAHR	1	S10(Phospho)	T(8): 15.6; S(10): 81.1; S(12): 3.3
P30622	TASESISNLsEAGsIKKGER	1	S10(Phospho); S14(Phospho)	T(1): 0.0; S(3): 0.0; S(5): 0.3; S(7): 12.5; S(10): 87.5; S(14): 99.7
Q2TAZ0	RLsAQAHPAGK	1	S3(Phospho)	S(3): 100.0
Q9P270	NSPRPsPKQsPRNsPR	1	S6(Phospho); S10(Phospho); S14(Phospho)	S(2): 3.5; S(6): 97.1; S(10): 99.4; S(14): 100.0
P11177	DAINQGMDEELERDEK	1		
P09525	DLLDDLK	2		
P02545;P02545	AQHEDQVEQYKK	1		
Q5T7B8	TSGNSSPKRIQSsPGALsEDK	1	S13(Phospho); S18(Phospho)	T(1): 35.7; S(2): 35.7; S(5): 8.9; S(6): 2.6; S(12): 9.0; S(13): 9.3; S(18): 98.7
Q08752	DGsGDSHPDFPEDADIDLKDVDK	1	S3(Phospho)	S(3): 96.2; S(6): 3.8
O95684	EKGPTTGEGALDLSDVHSPPKsPEGK	1	S22(Phospho)	T(5): 0.0; T(6): 0.0; S(14): 3.9; S(18): 16.9; S(22): 79.1
Q8IXF0	cYHFIHAEDVEGIR	1	N-Term(Acetyl); C1(Carbamidomethyl)	
P04920	RRPGASPTGETPTIEEGEEDEDEAsEAEGAR	1	S25(Phospho)	S(6): 0.0; T(8): 0.0; T(11): 0.0; T(13): 0.0; S(25): 100.0
Q6WCQ1	SKsVIEQVSWDT	1	S3(Phospho)	S(1): 0.6; S(3): 99.4; S(9): 0.0; T(12): 0.0
Q9Y450	DKPSVEPVEEYDYEDLKESSNSVSNHQLsGFDQAR	1	S29(Phospho)	S(4): 0.0; Y(11): 0.0; Y(13): 0.8; S(19): 3.4; S(20): 16.1; S(22): 78.9; S(24): 0.8; S(29): 0.0
Q7L4I2	KEQSEVSVsPR	1	S9(Phospho)	S(4): 83.6; S(7): 0.8; S(9): 15.5
Q96AQ6	ALQAPHsPSKTDGK	2	S7(Phospho)	S(7): 9.2; S(9): 1.0; T(11): 89.7
Q86W92	SQsTTFNPDDMSEPEFk	1	S3(Phospho); C-Term(Oxidation)	S(1): 12.0; S(3): 73.9; T(4): 12.0; T(5): 2.1; S(12): 0.0
Q6R327	GRNDsGEENVPLDLTR	1	S5(Phospho)	S(5): 99.0; T(15): 1.0
P08238	IEDVGsDEEDDsGKDKK	1	S6(Phospho); S12(Phospho)	S(6): 100.0; S(12): 100.0
Q14669	SEsPPAELPSLR	2	S3(Phospho)	S(1): 0.9; S(3): 99.1; S(10): 0.0
Q09666	EGAKDIDISsPEFK	1	S10(Phospho)	S(9): 1.4; S(10): 98.6
P78559	cLsPDDSTVK	2	C1(Carbamidomethyl); S3(Phospho)	S(3): 100.0; S(7): 0.0; T(8): 0.0
O14617	NTETSKsPEKDVPMVEk	4	S7(Phospho); C-Term(Oxidation)	T(2): 0.0; T(4): 9.1; S(5): 9.1; S(7): 81.9
Q9Y2V2	SRERsPsPLR	1	S5(Phospho); S7(Phospho)	S(1): 0.1; S(5): 99.9; S(7): 100.0
P11388	SVVsDLEADDVK	1	S4(Phospho)	S(1): 0.1; S(4): 99.9
Q9H6H4	SFsMQDLR	1	S3(Phospho)	S(1): 0.0; S(3): 100.0
Q08999	ADEIcIAGsPLtPR	1	C5(Carbamidomethyl); S9(Phospho); T12(Phospho)	S(9): 100.0; T(12): 100.0
Q969R2	VMNTHsDDsGDDDEATTPADKSELHHTLK	1	S6(Phospho); S9(Phospho)	T(4): 85.7; S(6): 16.9; S(9): 97.4; T(16): 0.0; T(17): 0.0; S(22): 0.0; T(27): 0.0
P46379	RVGDPPQPLPEEPMEVQGAERAsPEPQR	1	S23(Phospho)	S(23): 100.0
Q9Y548	GSGREEDDELLGNDDSDKTELLAGQK	1		
Q13207	AAGGNsREPsPLPELALR	1	S6(Phospho); S10(Phospho)	S(6): 100.0; S(10): 100.0
P46821	sVNFsLTPNEIK	1	S1(Phospho); S5(Phospho)	S(1): 100.0; S(5): 2.4; T(7): 97.6
P20645	GVGDDQLGEEsEERDDHLLPM	1	S11(Phospho)	S(11): 100.0
Q9H792	SHSsPSQIPK	1	S4(Phospho)	S(1): 0.0; S(3): 0.8; S(4): 91.2; S(6): 8.0
Q5T1B0	QyMEEIIKNIQKLyK	1	Y2(Phospho); Y14(Phospho)	Y(2): 100.0; Y(14): 100.0
Q7KZF4	DYVAPTANLDQK	1		
P07900;P08238	EDQTEYLEER	1		
Q969E4	EDEGEPGDEGQLEDEGsQEKQGR	1	S17(Phospho)	S(17): 100.0
Q8WWF8	VFRIMDDDNNR	1		
P49815	STsLNERPK	1	S3(Phospho)	S(1): 1.1; T(2): 1.1; S(3): 97.9
O60271	SASQSsLDKLDQELKEQQK	1	S6(Phospho)	S(1): 0.0; S(3): 0.3; S(5): 11.8; S(6): 87.9
Q5JSZ5	ADSHKEQAPKPSEQKDSEQGSGQSK	2		
Q07157	sVASSQPAKPTK	1	S1(Phospho)	S(1): 72.7; S(4): 9.1; S(5): 9.1; T(11): 9.1
P07602	DGGFcEVcKK	1	C5(Carbamidomethyl); C8(Carbamidomethyl)	
Q05209	NLsFEIK	1	S3(Phospho)	S(3): 100.0
Q8WWI1	MYsFDDVLEEGK	1	S3(Phospho)	Y(2): 1.7; S(3): 98.3
Q8IYB3	KSRVsVsPGR	1	S5(Phospho); S7(Phospho)	S(2): 0.2; S(5): 99.9; S(7): 99.9
P02545	SVTVVEDDEDEDGDDLLHHHHGSHcSSSGDPAEYNLR	1	C25(Carbamidomethyl)	
Q9NQ29	RsEEKEAGEI	1	S2(Phospho)	S(2): 100.0
Q9C073	ERGsPLLGDHAVR	1	S4(Phospho)	S(4): 100.0
P14618	LAPITSDPTEATAVGAVEASFK	1		
Q9UDY2	SSEPVQHEESIRKPsPEPR	1	S15(Phospho)	S(1): 0.0; S(2): 0.0; S(10): 3.7; S(15): 96.3
O75569	AEAPPLEREDSGtFSLGK	1	T13(Phospho)	S(11): 12.4; T(13): 85.6; S(15): 2.0
Q9UGV2	sRTHsTSSSLGSGESPFSR	1	S1(Phospho); S5(Phospho)	S(1): 2.7; T(3): 0.7; S(5): 2.7; T(6): 12.0; S(7): 12.0; S(8): 59.8; S(9): 14.8; S(12): 95.0; S(15): 0.3; S(18): 0.1
P25398	DVIEEYFK	1		
Q9NYF8	NTPSQHSHSIQHsPER	1	S13(Phospho)	T(2): 2.6; S(4): 61.8; S(7): 11.9; S(9): 11.9; S(13): 11.9
Q70EL1	KGDEPQASGYHsEGETLKEK	1	S12(Phospho)	S(8): 0.5; Y(10): 2.7; S(12): 94.0; T(16): 2.7
P78362	TVsAsSTGDLPK	1	S3(Phospho); S5(Phospho)	T(1): 0.8; S(3): 97.8; S(5): 1.5; S(6): 8.3; T(7): 91.7
O43493	DHSKPISNPSDNK	2		
O43164	NHGSsPEQVVRPK	2	S5(Phospho)	S(4): 8.1; S(5): 91.9
Q12986	NYSsPPPcHLSR	1	S4(Phospho); C8(Carbamidomethyl)	Y(2): 0.0; S(3): 10.6; S(4): 89.3; S(11): 0.0
O94854	EKTEsELKFEEDER	1	S5(Phospho)	T(3): 0.1; S(5): 99.9
Q96HB5	GQLIssPTFNAPAALFGEAAPQVk	1	S5(Phospho); S6(Phospho); C-Term(Oxidation)	S(5): 94.8; S(6): 94.8; T(8): 10.4
Q9NYV4	RRssSPFLSK	1	S3(Phospho); S4(Phospho)	S(3): 99.6; S(4): 6.0; S(5): 94.4; S(9): 0.0
Q13164	SQVEDPLPPVFsGtPK	1	S12(Phospho); T14(Phospho)	S(1): 0.0; S(12): 100.0; T(14): 100.0
Q8TD16	tSPGGRTsPEAR	2	T1(Phospho); S8(Phospho)	T(1): 47.1; S(2): 47.1; T(7): 17.2; S(8): 88.5
Q9UKA4	EDSGLFsPIR	1	S7(Phospho)	S(3): 0.0; S(7): 100.0
Q3KQU3	ESAAPAsPAPsPAPSPTPAPPQK	1	S7(Phospho); S11(Phospho)	S(2): 1.5; S(7): 98.6; S(11): 94.4; S(15): 5.2; T(17): 0.4
Q96JG6	SAYQEYDsDsDVPEELKR	1	S8(Phospho); S10(Phospho)	S(1): 0.5; Y(3): 0.8; Y(6): 86.8; S(8): 97.1; S(10): 14.8
P07355	GVDEVTIVNILTNR	1		

Q5T5U3	LRTsTSDLSR	1	S4(Phospho)	T(3): 4.5; S(4): 45.5; T(5): 4.5; S(6): 45.5; S(9): 0.0
Q8IZ21	RPLsSSHEASEGQAK	1	S4(Phospho)	S(4): 78.3; S(5): 10.8; S(6): 10.8; S(10): 0.1
Q8NE71	KLsVPtsDEEDEVPAPKPR	1	S3(Phospho); T6(Phospho); S7(Phospho)	S(3): 100.0; T(6): 100.0; S(7): 100.0
Q92598	IEsPKLER	1	S3(Phospho)	S(3): 100.0
Q12983	SSHcDsPPRSQTPQDTNR	1	C4(Carbamidomethyl); S6(Phospho)	S(1): 0.0; S(2): 0.0; S(6): 97.6; S(10): 0.3; T(12): 2.0; T(16): 0.0
P07900	ALLFVPR	2		
P85037	SGGLQTPEcLsREGsPIPHDPEFGSK	1	C9(Carbamidomethyl); S11(Phospho); S15(Phospho)	S(1): 0.8; T(6): 1.8; S(11): 98.7; S(15): 98.7; S(25): 0.0
O60763	DNQHQGSYSEGAQMNGIQPEEIGR	1		
Q15007	ETRQQLAQYQQQQSQASAPSTSRTTAsEPVEQSEATSk	2	S27(Phospho); C-Term(Oxidation)	T(2): 4.3; Y(9): 3.6; S(14): 3.6; S(17): 4.3; S(20): 4.3; T(21): 4.3; S(22): 4.3; T(24): 4.3; T(25): 4.3; S(27): 5.8; S(33): 27.4; T(36): 14.7; S(37): 14.7
Q9NVS9	NEELEQLYQDQEVPKPK	1		
Q6PKG0	tASISSSPSEGtPTVGSYGcTPQSLPK	1	T1(Phospho); T12(Phospho); C20(Carbamidomethyl)	T(1): 13.2; S(3): 13.2; S(5): 56.6; S(6): 15.5; S(7): 16.6; S(9): 16.6; T(12): 64.5; T(14): 3.8; S(17): 0.0; Y(18): 0.0; T(21): 0.0; S(24): 0.0
Q9BRS8	GHSGTTAsGGENEREDLEQEWKPPDEELIKK	1	S8(Phospho)	S(3): 2.4; T(5): 2.4; T(6): 7.4; S(8): 87.7
Q8IYB3	RLsPsAsPPR	1	S3(Phospho); S5(Phospho); S7(Phospho)	S(3): 100.0; S(5): 100.0; S(7): 100.0
Q5JSH3	RKsELEFETLK	1	S3(Phospho)	S(3): 100.0; T(9): 0.0
O75592	APLKDEQEMRAsPK	1	S12(Phospho)	S(12): 100.0
Q15149	SSsVGsSSSYPISPAVSR	1	S3(Phospho); S6(Phospho)	S(1): 21.9; S(2): 21.9; S(3): 21.9; S(6): 74.6; S(7): 56.7; S(8): 2.3; S(9): 0.5; Y(10): 0.1; S(13): 0.0; S(17): 0.0
Q5HYW2	SsLPPTsPMEKFPKsr	1	S2(Phospho); S7(Phospho); S15(Phospho); C-Term(Oxidation)	S(1): 13.0; S(2): 13.0; T(6): 75.3; S(7): 98.7; S(15): 100.0
Q9Y2I7	GTAGKsPDLSSQK	1	S6(Phospho)	T(2): 0.3; S(6): 99.7; S(10): 0.0; S(11): 0.0
Q9HAW4	NIDDASQMDLFHRDsDDDQTEEQLDESEAR	1	S15(Phospho)	S(6): 0.5; S(15): 99.0; T(20): 0.5; S(27): 0.0
Q7Z5K2	RPESPsEIsPIKGSVR	1	S6(Phospho); S9(Phospho)	S(4): 50.0; S(6): 50.0; S(9): 100.0; S(14): 0.0
P78318	AAQQQEEQEEKEEEDDEQTLHR	1		
Q8WW12	TLSVAAAFNEDEDsEPEEMPPEAk	1	S14(Phospho); C-Term(Oxidation)	T(1): 0.2; S(3): 0.2; S(14): 99.6
Q9UPT8	LtRHVEASGGSGPGDsGPsDPRLAR	1	T2(Phospho); S16(Phospho); S19(Phospho)	T(2): 97.1; S(8): 2.7; S(11): 0.8; S(16): 99.7; S(19): 99.7
Q08AD1	SEsVEGFLSPSR	1	S3(Phospho)	S(1): 0.7; S(3): 99.3; S(9): 0.0; S(11): 0.0
Q8TC71	SRsLsPlcPR	1	S3(Phospho); S5(Phospho); C8(Carbamidomethyl)	S(1): 1.6; S(3): 98.6; S(5): 99.8
Q13625	NQsSEDILR	1	S3(Phospho)	S(3): 99.0; S(4): 1.0
Q9H4G0	SLDGAEFSRPASVsENHDAGPDGDKRDEDGESGGQR	1	S14(Phospho)	S(1): 1.2; S(8): 3.3; S(12): 3.3; S(14): 92.0; S(32): 0.1
P41208	RMsPKPELTEEQKQEIR	1	S3(Phospho)	S(3): 99.9; T(9): 0.1
Q92934	HSsYPAGTEDDEGMGEEPSPFr	2	S3(Phospho); C-Term(Oxidation)	S(2): 36.3; S(3): 36.3; Y(4): 11.6; T(8): 4.1; S(19): 11.6
Q9UDY2	SRsWEDSPER	1	S3(Phospho)	S(1): 0.8; S(3): 99.2; S(7): 0.0
Q13972	eLDNNRSALsAAsAFAIATAGANEGTPNKEk	1	N-Term(Acetyl); S10(Phospho); S13(Phospho); C-Term(Oxidation)	S(7): 13.8; S(10): 28.6; S(13): 64.4; T(19): 85.6; T(26): 7.5
Q8IYB3	KVELsEsEEDKGGK	1	S5(Phospho); S7(Phospho)	S(5): 100.0; S(7): 100.0
Q5T5U3	FKsDSGSLGDAK	1	S3(Phospho)	S(3): 99.4; S(5): 0.3; S(7): 0.3
P25205	DGDSYDPYDFSDtEEMPQVHtPk	1	T13(Phospho); T22(Phospho); C-Term(Oxidation)	S(4): 0.0; Y(5): 0.0; Y(8): 2.8; S(11): 83.2; T(13): 16.8; T(22): 97.2
Q9NZD8	GSLGIsQEEQ	1	S6(Phospho)	S(2): 0.0; S(6): 100.0
Q7Z6Z7	DGGsGNsTIIVSR	1	S4(Phospho); S7(Phospho)	S(4): 91.8; S(7): 54.1; T(8): 54.1; S(12): 0.1
Q09666	GDLKSsKAsLGsLEGEAEAEASSPK	1	S6(Phospho); S9(Phospho); S12(Phospho)	S(5): 84.4; S(6): 84.4; S(9): 32.3; S(12): 99.0; S(22): 0.0; S(23): 0.0
Q8WXF7	DDHSFELDETALNR	1		
Q13136	RSsDGSLSHEEDLAK	1	S3(Phospho)	S(2): 0.2; S(3): 10.0; S(6): 79.8; S(8): 10.0
Q9H992	IKESLLLEDsEEEEGDLcR	1	S10(Phospho); C18(Carbamidomethyl)	S(4): 0.4; S(10): 99.6
P17812	SGSssPDSEITELK	1	S4(Phospho); S5(Phospho)	S(1): 95.5; S(3): 13.3; S(4): 4.2; S(5): 75.3; S(8): 11.7; T(11): 0.0
Q8IU81	AGGAsPAASSTAQPPTQHR	1	S5(Phospho)	S(5): 97.0; S(9): 1.0; S(10): 1.0; T(11): 1.0; T(16): 0.1
Q9Y2D5	DALGDSLQVPVSPSsTTsSR	1	S15(Phospho); S18(Phospho)	S(6): 0.1; S(12): 71.1; S(14): 14.3; S(15): 14.3; T(16): 8.9; T(17): 8.4; S(18): 41.5; S(19): 41.5
P20810	DTSQSDKDLDDALDK	1		
Q7Z2W4	AsLEDAPVDDLTR	1	S2(Phospho)	S(2): 100.0; T(12): 0.0
Q9NPR2	LGsEIRDSVV	1	S3(Phospho)	S(3): 98.0; S(8): 2.0
Q71RC2	EQYVPPRsPK	1	S8(Phospho)	Y(3): 0.0; S(8): 100.0
P13807	HSsPHQSEDEEDPR	2	S3(Phospho)	S(2): 11.4; S(3): 86.9; S(7): 1.7
Q15435	VEsEEsGDEEGKKHSSGIVADLSEQSLK	1	S3(Phospho); S6(Phospho)	S(3): 100.0; S(6): 100.0; S(15): 0.0; S(16): 0.0; S(23): 0.0; S(26): 0.0
Q13557	NFsAAKsLLK	1	S3(Phospho); S7(Phospho)	S(3): 100.0; S(7): 100.0
Q2M389	DQNDHKYPFDR	1		
Q15052	KPsEEEYVIRK	1	S3(Phospho)	S(3): 100.0; Y(7): 0.0
Q9C0C2	DsLGTYSSR	1	S2(Phospho)	S(2): 98.5; T(5): 1.3; Y(6): 0.2; S(7): 0.0; S(8): 0.0
Q99611	yGEGHQAWIVGIVEK	1	Y1(Phospho)	Y(1): 100.0
Q9H3Z4	sLSTSGESLYHVLGLDK	2	S1(Phospho)	S(1): 49.3; S(3): 49.3; T(4): 0.6; S(5): 0.6; S(8): 0.1; Y(10): 0.0
P35606	STAQQELDGKPASPtPVIVASHTANKEEK	2	T15(Phospho)	S(1): 0.0; T(2): 0.0; S(13): 74.3; T(15): 21.3; S(21): 2.2; T(23): 2.2
P55040	WsIPADGR	1	S2(Phospho)	S(2): 100.0
Q6ZRS2	iLRSSAPPsLAGPAVsHRGr	1	N-Term(Acetyl); S9(Phospho); S16(Phospho); C-Term(Oxidation)	S(4): 1.3; S(5): 1.3; S(9): 98.1; S(16): 99.4
P78559	ELSSPIsPK	1	S7(Phospho)	S(3): 0.0; S(4): 0.1; S(7): 99.9
Q8IYB3	EKtPELPEPSVK	1	T3(Phospho)	T(3): 99.9; S(10): 0.1
P15374	KFLEESVSMsPEER	1	S10(Phospho)	S(6): 2.8; S(8): 14.4; S(10): 82.8
Q92521	StLYFNTQEK	2	T2(Phospho)	S(1): 49.9; T(2): 49.9; Y(4): 0.2; T(7): 0.0
Q7Z591	YAEAENTIDQLr	1	C-Term(Oxidation)	
Q9Y5W7	GSLsLDDFR	1	S4(Phospho)	S(2): 1.0; S(4): 99.0
O60841	WDGsEEDEDNSKK	1	S4(Phospho)	S(4): 100.0; S(11): 0.0
Q5SW79	TPLTsADEHVHSK	1	S5(Phospho)	T(1): 0.0; T(4): 49.6; S(5): 49.6; S(12): 0.7
Q01082	KHYASEEIKEK	1		
P78536	sFEDLTDHPVTR	1	S1(Phospho)	S(1): 100.0; T(6): 0.0; T(11): 0.0
Q8NHQ9	TVDLGIsDLEDDc	1	S7(Phospho); C13(Carbamidomethyl)	T(1): 2.9; S(7): 97.1
Q7Z698	QVsFPDDDEEIVR	1	S3(Phospho)	S(3): 100.0
O75122	IPRPSVsQGcsR	1	S7(Phospho); C10(Carbamidomethyl); S11(Phospho)	S(5): 55.0; S(7): 55.0; S(11): 89.9
P49736	RGLLyDSDEEDEERPAR	1	Y5(Phospho)	Y(5): 89.3; S(7): 10.7
Q13017	RTHsDAsDDEAFTTSK	1	S4(Phospho); S7(Phospho)	T(2): 50.0; S(4): 50.0; S(7): 99.9; T(13): 0.0; T(14): 0.0; S(15): 0.0
Q02952	EGVTPWAsFK	1	S8(Phospho)	T(4): 0.0; S(8): 100.0
Q9P0V3	NSTLsDsGMIDNLPDSPDEVAK	1	S5(Phospho); S7(Phospho)	S(2): 50.0; T(3): 50.0; S(5): 50.0; S(7): 50.0; S(16): 0.0
Q96FT9	RKAsEEIEDFR	1	S4(Phospho)	S(4): 100.0
P09601	DQsPSRAPGLR	1	S3(Phospho)	S(3): 99.3; S(5): 0.7
Q9Y3T9	EAARsPDKPGGsPSASR	1	S5(Phospho); S12(Phospho)	S(5): 100.0; S(12): 97.6; S(14): 1.2; S(16): 1.2
P06733;P09104	YDLDFK	1		
Q9UBL0	SKsIEEREEEYQR	1	S3(Phospho)	S(1): 13.2; S(3): 86.7; Y(11): 0.1
Q5T5U3	AQPSSsEDELDNVFFK	1	S6(Phospho)	S(4): 2.7; S(5): 2.7; S(6): 94.6
P09104	DGKYDLDFK	2		
O43432	SPsPVLR	1	S3(Phospho)	S(1): 0.0; S(3): 100.0
P08670	DGQVINETSQHHDDLE	2		
Q8IUD2	VEPSSQSPGRsPR	1	S11(Phospho)	S(4): 0.0; S(5): 0.0; S(7): 8.4; S(11): 91.6
P09382	DSNNLcLHFNPR	1	C6(Carbamidomethyl)	
Q49MG5	MNDFHIsDDEEKNPSK	1	S7(Phospho)	S(7): 99.9; S(15): 0.1
Q92783	DPGTVANKKEEEDLAK	1		

Q96PU5	sLsSPTVTLSAPLEGAK	2	S1(Phospho); S3(Phospho)	S(1): 89.3; S(3): 89.3; S(4): 20.2; T(6): 0.6; T(8): 0.3; S(10): 0.3
P62753	RLssLR	2	S3(Phospho); S4(Phospho)	S(3): 100.0; S(4): 100.0
Q13541	NsPVtKTPPR	1	S2(Phospho); T5(Phospho)	S(2): 100.0; T(5): 91.3; T(7): 8.7
Q02952	RPsEsDKEDELDKVK	1	S3(Phospho); S5(Phospho)	S(3): 100.0; S(5): 100.0
Q96F86	RHNsWSSSSR	1	S4(Phospho)	S(4): 99.0; S(6): 0.9; S(7): 0.1; S(8): 0.0; S(9): 0.0
O94826	AsPAPGSGHPEGPGAHLDMNSLDr	1	S2(Phospho); C-Term(Oxidation)	S(2): 100.0; S(7): 0.0; S(21): 0.0
P49321	ATLVESStSGFtPGGGGSSVSMIASRKPtDGASSSNcVTDISHLVR	1	T8(Phospho); T12(Phospho); T29(Phospho); C37(Carbamidomethyl)	Too many isoforms
P24534	YGPADVEDTTGSGATDsKDDDDIDLFGsDDEEESEEAKR	1	S17(Phospho); S28(Phospho)	Y(1): 0.1; T(9): 1.5; T(10): 1.5; S(12): 17.5; T(15): 17.5; S(17): 63.5; S(28): 98.4; S(34): 0.0
O75122	SRsDIDVNAAAGAK	1	S3(Phospho)	S(1): 8.2; S(3): 91.8
Q3YEC7	NDSDLFGLGLEEAGPKEssEEGKEGK	1	S18(Phospho); S19(Phospho)	S(3): 0.0; S(18): 100.0; S(19): 100.0
Q3KQU3	RSsQPSPTAVPASDSPPTK	1	S3(Phospho)	S(2): 0.2; S(3): 6.9; S(6): 46.4; T(8): 46.4; S(13): 0.0; S(15): 0.0; T(18): 0.0
O14525	LNQVAIsQALsNALHSLDGATSr	1	S7(Phospho); S11(Phospho); C-Term(Oxidation)	S(7): 100.0; S(11): 100.0; S(16): 0.0; T(21): 0.0; S(22): 0.0
Q8WUY3	RAsDSVFQPK	1	S3(Phospho)	S(3): 99.0; S(5): 1.0
O00418	ESENSGDsGYPSEKRGELDDPEPR	2	S8(Phospho)	S(2): 0.1; S(5): 0.1; S(8): 0.6; Y(10): 0.6; S(12): 98.5
Q8IVH2	RDsSSHEETPGSHPLYGHGEcK	1	S3(Phospho); C21(Carbamidomethyl)	S(3): 46.8; S(4): 46.8; S(5): 6.4; T(9): 0.0; S(12): 0.0; Y(16): 0.0
Q16512	TDVSNFDEEFTGEAPTLsPPR	1	S18(Phospho)	T(1): 0.0; S(4): 0.0; T(11): 0.0; T(16): 4.3; S(18): 95.6
Q9Y3D6	yVRGLLQtEPQNNQAK	1	Y1(Phospho); T8(Phospho)	Y(1): 100.0; T(8): 100.0
Q8NHS3	MAGLRNESEQEPLLGDTPGSR	1		
Q9NRA8	ASEENLLSSSSVPSADRDsSPTTNSK	1	S19(Phospho)	S(2): 0.0; S(8): 0.0; S(9): 0.0; S(10): 0.0; S(11): 0.0; S(14): 0.2; S(19): 9.6; S(20): 9.6; T(22): 9.6; T(23): 69.5; S(25): 1.4
Q13625	KNQsSEDILR	1	S4(Phospho)	S(4): 92.7; S(5): 7.3
Q9Y4E8	lDERSFLALDWDPDLKK	1	N-Term(Acetyl)	
Q6WCQ1	SKsNPDFLKK	1	S3(Phospho)	S(1): 8.1; S(3): 91.9
Q8WWM7	GPHHLDNSsPGPGSEAR	1	S9(Phospho)	S(8): 49.8; S(9): 49.8; S(14): 0.4
Q5SW79	GNKHDDGTQSDSENAGAHR	1		
P46821	sPPLIGSESAYESFLSADDK	1	S1(Phospho)	S(1): 96.5; S(7): 2.9; S(9): 0.6; Y(11): 0.0; S(13): 0.0; S(16): 0.0
Q9UJD0	QGsREsTDGSTNSNSSDGTFIFPTTR	1	S3(Phospho); S6(Phospho)	S(3): 74.1; S(6): 8.4; T(7): 8.4; S(10): 17.2; T(11): 25.6; S(13): 65.4; S(15): 0.6; S(16): 0.2; T(19): 0.1; T(24): 0.0; T(25): 0.0
O43847	DAIDREVEAVDSEYQLARPSDANR	1		
P34932	DKLSGEYEK	1		
Q14315	LGsFGsITR	1	S3(Phospho); S6(Phospho)	S(3): 99.8; S(6): 91.0; T(8): 9.2
Q9UDY2	AASsDQLRDNsPPPAFKPEPPK	2	S4(Phospho); S11(Phospho)	S(3): 88.0; S(4): 23.9; S(11): 88.0
P98082	SsPNPFVGsPPK	1	S2(Phospho); S9(Phospho)	S(1): 50.2; S(2): 50.2; S(9): 99.7
P30533	REsGEEFR	1	S3(Phospho)	S(3): 100.0
P02768	YIcENQDSISSK	1	C3(Carbamidomethyl)	
Q9H0X4	KsQENLGNPSKNEDNVK	1	S2(Phospho)	S(2): 99.4; S(10): 0.6
P78559	sPESLPGPALEDIAIK	2	S1(Phospho)	S(1): 50.0; S(4): 50.0
Q9Y6K9	RsPPEEPPDFccPK	1	S2(Phospho); C11(Carbamidomethyl); C12(Carbamidomethyl)	S(2): 100.0
Q6Y7W6	SQsWEER	1	S3(Phospho)	S(1): 0.5; S(3): 99.5
Q8IWS0	DRsPHRsSPSDTRPK	1	S3(Phospho); S7(Phospho)	S(3): 100.0; S(7): 8.2; S(8): 91.7; S(10): 0.1; T(12): 0.0
Q16566	LGSASSSHGSIQESHKAsRDPsPIQDGNEDMK	1	S18(Phospho); S22(Phospho)	S(3): 5.2; S(5): 22.3; S(6): 22.3; S(7): 22.3; S(10): 22.3; S(14): 5.3; S(18): 0.8; S(22): 99.4
Q13322	SIQPQVsPR	1	S7(Phospho)	S(1): 0.0; S(7): 100.0
P08238	GEKEEEDKDDEEKPK	1		
P78559	SPFEIIsPPAsPPEMVGQr	1	S7(Phospho); S11(Phospho); C-Term(Oxidation)	S(1): 0.2; S(7): 99.8; S(11): 100.0
P23588	NRDSDKTDTDWR	1		
Q14498	YRsPYSGPK	1	S3(Phospho)	Y(1): 49.8; S(3): 49.8; Y(5): 0.4; S(6): 0.0
Q5T5U3	HNKsPTLScR	1	S4(Phospho); C9(Carbamidomethyl)	S(4): 99.9; T(6): 0.1; S(8): 0.0
P04233	SRscREDQKPVMDDQR	1	S3(Phospho); C4(Carbamidomethyl)	S(1): 12.4; S(3): 87.6
P49736	RGNDPLTSsPGR	1	S9(Phospho)	T(7): 7.6; S(8): 7.6; S(9): 84.9
Q96HE9	SPTPTLPLsTSSFDEQN	1	S9(Phospho)	S(1): 1.6; T(3): 1.6; T(5): 1.6; S(9): 42.8; T(10): 42.8; S(11): 1.6; S(12): 7.9
O60271	SsTLSQLPGDK	2	S2(Phospho)	S(1): 46.4; S(2): 46.4; T(3): 7.0; S(5): 0.3
P55072	DVDLEFLAK	1		
P29966	AEDGATPSPSNEtPKKK	1	T13(Phospho)	T(6): 0.0; S(8): 0.2; S(10): 1.6; T(13): 98.1
Q5T0F9	ASSSKEsPSPSVR	1	S7(Phospho)	S(2): 0.6; S(3): 0.6; S(4): 0.6; S(7): 94.8; S(9): 2.9; S(11): 0.6
Q9Y6M7	KHsDPHLLER	1	S3(Phospho)	S(3): 100.0
Q9UNL2	QQsEEDLLLQDFSR	1	S3(Phospho)	S(3): 99.7; S(13): 0.3
P52565	SIQEIQELDKDDESLRK	1		
P13693	GKLEEQRPER	1		
P78363	IAWRAAKPLLMGKILytPDsPAAr	1	Y16(Phospho); T17(Phospho); S20(Phospho); C-Term(Oxidation)	Y(16): 100.0; T(17): 100.0; S(20): 100.0
P16383	VLSGNcNHQEGTssDDELPSAEMIDFQK	1	C6(Carbamidomethyl); S13(Phospho); S14(Phospho)	S(3): 3.1; T(12): 65.6; S(13): 65.6; S(14): 65.6; S(20): 0.0
Q5TIA1	SAcRLAIEFQSEPSAQENPFTAPsAKk	1	C3(Carbamidomethyl); S24(Phospho); C-Term(Oxidation)	S(1): 0.0; S(11): 0.2; S(14): 0.2; T(21): 49.8; S(24): 49.8
Q13501	IALESEGRPEEQMESDNcSGGDDDWTHLSsk	1	C18(Carbamidomethyl); S30(Phospho); C-Term(Oxidation)	S(5): 0.0; S(15): 0.0; S(19): 0.1; T(26): 0.7; S(29): 49.6; S(30): 49.6
Q6R327	NDsGEENVPLDLTREPSDNLR	1	S3(Phospho)	S(3): 100.0; T(13): 0.0; S(17): 0.0
P16949	SKESVPEFPLsPPK	1	S11(Phospho)	S(1): 0.0; S(4): 0.0; S(11): 100.0
Q04917	NcNDFQYESK	1	C2(Carbamidomethyl)	
P06748	DELHIVEAEAMNYEGsPIK	1	S16(Phospho)	Y(13): 15.1; S(16): 84.9
Q9NY27	EANLQQNEEKNHSDSSTSESEVSSVsPLK	1	S26(Phospho)	S(13): 0.0; S(15): 0.0; S(16): 0.0; T(17): 0.0; S(18): 0.0; S(20): 0.0; S(23): 1.0; S(24): 4.3; S(26): 94.7
Q7L8J4	TVAsDLQKcDsVEHLR	1	S4(Phospho); C9(Carbamidomethyl); S11(Phospho)	T(1): 0.9; S(4): 99.4; S(11): 99.7
Q9H2P0	nTTWEDVGLWDPSLTK	1	N-Term(Acetyl)	
O43149	GDREEEVERPVSsPGDPEQK	1	S13(Phospho)	S(12): 50.0; S(13): 50.0
P78559	ELVPEVPEEQGsKDR	1	S12(Phospho)	S(12): 100.0
Q9GZT3	LPQTsDDEKKDF	1	S5(Phospho)	T(4): 0.6; S(5): 99.4
Q5SW79	cStGSPSKDVTK	1	C1(Carbamidomethyl); T3(Phospho)	S(2): 1.0; T(3): 89.2; S(5): 8.8; S(7): 1.0; T(11): 0.0
Q14152	NTDDDRPPRR	1		
O94876	QLsKIER	1	S3(Phospho)	S(3): 100.0
P62328	TETQEKNPLPSK	1		
Q8IUD2	VEPSSQsPGRsPR	1	S7(Phospho); S11(Phospho)	S(4): 11.8; S(5): 11.8; S(7): 76.6; S(11): 99.8
O15013	tKSLIAQDHR	1	T1(Phospho)	T(1): 8.0; S(3): 92.0

Sample: siPIM

Accessions	Sequence	PSM	Modifications	phosphoRS Site Probabilities
Q9UPN3	RGsDASDFDLLETQSAcSDTSESSAAGGQGNSR	4	S3(Phospho); C17(Carbamidomethyl)	S(3): 99.9; S(6): 0.1; T(13): 0.0; S(15): 0.0; S(18): 0.0; T(20): 0.0; S(21): 0.0; S(23): 0.0; S(24): 0.0; S(32): 0.0
O43934	sASPDDDLGSSNWEAADLGNEER	6	S13(Phospho)	S(4): 0.0; S(13): 88.3; S(14): 11.7; S(24): 0.0; T(30): 0.0
O00193	SKEGHSLEMENENLVENGADsDEDDNSFLK	2	S3(Phospho)	S(1): 50.0; S(3): 50.0; S(10): 0.0; S(11): 0.0
O95400	SQIDVALsQDSTYQGER	1	S5(Phospho)	S(2): 0.0; S(5): 100.0; S(16): 0.0; S(17): 0.0
Q92539	DHGFEVASTsPEDESPGSNPEPDATPFQEGLR	7	C14(Carbamidomethyl); S19(Phospho); S20(Phospho)	S(7): 0.0; T(13): 0.0; S(19): 100.0; S(20): 100.0
Q5T4S7	NHSDSSTSESEVSSVsPLK	2	T4(Phospho)	T(1): 0.4; S(3): 4.0; T(4): 45.8; S(5): 45.8; S(6): 4.0; Y(11): 0.0; S(13): 0.0; T(17): 0.0
O00264	LKDLGHPVEEEDELEsGDQEDEDDESEDPGKDLDHI	1	S13(Phospho)	S(10): 0.0; S(13): 100.0
Q5JSH3	MQVDQEEPHVEEQQQQTPAENKAESEEMETSQAGSK	2	S10(Phospho)	Y(2): 0.0; S(4): 0.0; S(10): 100.0; S(18): 0.0; T(21): 0.0; T(23): 0.0
O00193	HNDDEQYAWESSAGGSFTVR	7	S2(Phospho)	S(2): 50.0; S(4): 50.0; S(11): 0.0; S(12): 0.0
Q9Y2X7	SKDHFGLEGDEESTMLEDSVsPK	6	S3(Phospho)	S(3): 99.2; S(7): 0.7; Y(9): 0.1; T(12): 0.0; S(14): 0.0
Q15185	EVSsLEGsPPPcLGQEEAVcTK	3	S6(Phospho)	S(6): 100.0; S(11): 0.0
P05388	LQEEQDGGssDEDRAGPAPPGASDGVDIQDVK	2	S7(Phospho); S10(Phospho)	S(7): 100.0; S(10): 100.0
Q9C0C2	DNLTLWTSDsAGEEcDAAEGAEN	12	S3(Phospho); S4(Phospho)	S(3): 100.0; S(4): 100.0; S(14): 0.0
P46821	LLRDDEAHLQEDQGEEEcFHDcSASFEEEPGADKVENK	3	S14(Phospho)	S(4): 0.0; T(7): 0.0; T(12): 0.0; S(14): 99.2; S(16): 0.8; T(23): 0.0
O00505	KDsEEEVSLLGSQDIEEGNHQVEDGcR	2	S11(Phospho)	S(7): 0.0; S(11): 100.0
P35221	LPDsDDDEDEETAIQR	3	S8(Phospho)	T(1): 0.0; S(8): 100.0; T(12): 0.0
Q9UBB9	NADMSEEMQQDSVEcATQALEK	2	S11(Phospho)	S(11): 100.0
Q96K21	KLSsSDAPAQDTGSSAAAVETDASR	3	S4(Phospho)	S(4): 100.0; T(12): 0.0
Q9UGV2	DAHDVsPTsTDTEAQLTVER	6	S7(Phospho); C14(Carbamidomethyl)	S(1): 0.0; T(3): 0.4; S(4): 4.2; S(7): 47.7; T(10): 47.7; S(13): 0.0; S(16): 0.0
P13861	SFGTRPLSsGFSPEEAQQQDEEFEK	5	S3(Phospho); C5(Carbamidomethyl)	S(3): 99.9; T(8): 0.1; Y(9): 0.0; T(18): 0.0
Q8IXJ6	NHSVNEEEQEEQGEGsEDEWEQVGPR	1	S7(Phospho); S9(Phospho)	S(7): 90.8; S(9): 90.8; S(11): 18.4
P23588	VQGEAVSNIQENTQTPTVQEEsEEEEVDETGVEVK	1	S4(Phospho)	S(1): 0.0; S(3): 6.4; S(4): 93.6; T(6): 0.0; S(10): 0.0; T(12): 0.0; S(13): 0.0
O60271	RHSsDINHLVTQGREsPEGSYTDDANQEVR	1	S2(Phospho)	S(2): 87.7; S(4): 12.3; S(18): 0.0; S(21): 0.0; S(25): 0.0
P50502	DWEDDsDEDMSNFDR	7	S12(Phospho); S13(Phospho); S16(Phospho)	S(12): 100.0; S(13): 100.0; S(16): 100.0
P29692	KVEEEQEADEEDVsEEEAESK	1	S19(Phospho)	T(4): 0.0; S(19): 100.0
Q9BV36	SPLGEAPEPDsDAEVAEAAKPHLSEVTTEGYPSEPLGGLEQK	2	C14(Carbamidomethyl); S28(Phospho)	T(9): 0.0; S(12): 0.0; S(16): 0.0; T(23): 0.0; S(24): 1.0; S(26): 49.5; S(28): 49.5
Q9H3N1	EYVSNDAAQsDDEEKLQSQPTDTDGGR	3	S14(Phospho)	S(14): 100.0; S(20): 0.0; T(24): 0.0
P09603	SLDLDHSMDEAGAGASNSEPSEPDsPTREHAR	3	S10(Phospho)	S(1): 0.0; S(10): 100.0; S(18): 0.0; T(21): 0.0
Q70E73	cSMSSSADFsDEDDFSQK	1	N-Term(Acetyl); S5(Phospho); S17(Phospho)	S(5): 100.0; S(17): 100.0
Q8IVF2	LtVENSPKQEAGISEGQGTAGEEEEK	4	S6(Phospho)	S(6): 99.9; T(8): 0.0; S(9): 0.0; T(10): 0.0; T(12): 0.0; T(17): 0.0
P08240	FLESAAADFsDEDEDDDVDGREK	1	C13(Carbamidomethyl); S14(Phospho); S15(Phospho); S16(Phospho)	T(2): 0.0; S(4): 0.0; S(14): 100.0; S(15): 100.0; S(16): 100.0; S(25): 0.0; T(26): 0.0; S(29): 0.0; T(31): 0.0
Q9NY27	KVVDYSQFQEsDDADEDYGR	4	S16(Phospho)	S(3): 0.0; S(5): 0.0; S(6): 0.0; T(7): 0.0; S(8): 0.0; S(10): 0.0; S(13): 0.0; S(14): 0.1; S(16): 99.9
P61981	GNAEGssDEEGKLVIDEPAK	4		
Q08211	NHETDGGsAHGDDDDDGPHFEPVVPLPDKIEVK	1	C14(Carbamidomethyl); C18(Carbamidomethyl)	
O95365	cGSGPVHISGQHLVAVEEDAEsEDEEEEDVK	16	S13(Phospho)	S(13): 100.0
O75962	TLHcEGTEINsDDEQESKEVEETATAK	4	S11(Phospho)	S(2): 0.0; S(7): 0.1; S(11): 99.9; S(14): 0.0; T(17): 0.0; T(22): 0.0
Q13610	TAKDsDDDDDVAVTVDR	1	S10(Phospho)	S(10): 100.0; T(15): 0.0; S(17): 0.0; S(19): 0.0; S(25): 0.0
P48681	SLsPEDAESLSVLSVPSPDTANQEPTPK	7	S13(Phospho)	S(4): 0.0; S(13): 100.0
O75643	VSAEAEVAPVsPEVTQEVVEEHcAsPEDK	5	S4(Phospho)	S(4): 100.0
Q09666	SVTSNQSDGtQEScESPDVLDR	3	S3(Phospho)	S(3): 100.0; T(14): 0.0; S(16): 0.0
Q8NFC6	sLDGLSEAcGGAGSSGSAESGAGGGR	1	S6(Phospho); S7(Phospho)	Y(4): 21.7; S(6): 89.1; S(7): 89.1; S(20): 0.0
P78559	RGTGQsDDsDIWDDTALIK	7	S6(Phospho); S8(Phospho)	S(1): 0.0; S(6): 12.4; T(7): 93.8; S(8): 93.8; S(10): 0.1
Q9BXF6	SPEKIEEVLSPEGSPSKsPSK	4	S1(Phospho)	S(1): 46.0; S(3): 46.0; S(4): 4.0; S(5): 4.0; S(15): 0.0
P55010	KIAELEEEQSQGSTTNsDWMK	8	S6(Phospho); S7(Phospho)	S(6): 100.0; S(7): 100.0; Y(22): 0.0; S(23): 0.0
P48681	IALESEGRPEEQMESDNcsGGDDDWTHLSSK	3	S4(Phospho); S13(Phospho)	S(4): 100.0; S(13): 100.0
Q14160	SHQEPQRADsPLEQPEGSPLTQDDR	1	C5(Carbamidomethyl); C7(Carbamidomethyl); S13(Phospho)	S(2): 0.0; S(11): 0.0; S(13): 100.0
Q6PKG0	mEKPYNKNEGNLENEGKPEDEVEPDDEGKsDEEEKPDVEGK	1	T3(Phospho); S25(Phospho)	T(3): 79.7; S(10): 79.7; S(25): 40.7
P25788	KEEsEEsDDDMGFGLFD	5	S9(Phospho); C-Term(Oxidation)	S(2): 0.0; S(9): 100.0
Q9H1E3	NGILAIEGTGSDVDDDMsGDEKQDNESNVDPR	3	S10(Phospho)	Y(4): 0.0; S(5): 0.0; S(10): 100.0; Y(17): 0.0; S(21): 0.0; T(25): 0.0
P31323	RSsTVAPAQPDGAESEWTDVETR	3	S3(Phospho); C5(Carbamidomethyl)	S(3): 100.0; Y(9): 0.0; S(20): 0.0
O43719	KAEQGsEEEGEGEEEEEEGGESKADDPYAHLSK	1	S5(Phospho)	S(5): 100.0
P06748	ESPRPLQLPGAEGPAIsDGEEGGGEPGAGGGAAGAAGAGR	2	C1(Carbamidomethyl); S22(Phospho)	S(3): 0.0; S(9): 0.0; S(22): 100.0
P46821	SPEKIEEVLSPEGSPsKsPSK	1	S4(Phospho); S5(Phospho)	S(1): 0.0; S(4): 100.0; S(5): 100.0; T(10): 0.0
P55196	RDsLGAYASQDANEQGQDLGK	2	S6(Phospho); S15(Phospho)	S(5): 89.7; S(6): 10.3; S(15): 100.0
P35579	SHWDDsTsDSELEK	1	S6(Phospho)	S(6): 100.0
P46821	AAEAGGAEEQYGFLTtPTK	2	T4(Phospho)	T(1): 3.9; T(2): 48.0; T(4): 48.0; Y(10): 0.0; S(11): 0.0; Y(12): 0.0; S(15): 0.0
P23528	DKsPVREPIDNLTPEER	4	C7(Carbamidomethyl)	
P30622	KDDsDDDGGGWITPSNIK	3	S14(Phospho)	T(1): 0.0; S(3): 0.0; S(5): 0.0; S(7): 0.0; S(10): 0.0; S(14): 100.0
P63104	HGsGADsDYENTQSGDPLLGLEGK	4		
Q8IYB3	ASVSPMDEPVPDSEsPIEK	5	T3(Phospho); S5(Phospho)	T(3): 100.0; S(5): 100.0
Q9H3N1	KNTFTAWsDEESDYEIDDRDVNK	3	S14(Phospho)	S(14): 100.0; S(20): 0.0
Q13442	YMAENPTAGVVQEEEEDNLEYDsDGNPIAPTKK	3	S4(Phospho); S7(Phospho)	S(1): 6.5; S(4): 93.5; S(7): 100.0; Y(14): 0.0
Q14694	RNsEGSELScTEGSLTSSLDSR	5	S16(Phospho)	S(3): 0.0; S(16): 100.0
Q9UHB6	YTLENKEEGSLsDTEADAVSGQLPDPTTNPSAGK	2	S19(Phospho)	S(4): 0.0; S(19): 13.4; S(25): 86.6
Q63HN8	DNLTLWTSENQGDEGDAGEGEN	6	S20(Phospho)	S(7): 0.0; S(20): 100.0; S(26): 0.0
Q96F86	ELNSNHDGADETSEKEQQEAIEHIDEVQNEIDR	1	S7(Phospho); C13(Carbamidomethyl)	S(1): 0.0; S(7): 100.0; S(14): 0.0
Q8N2F6	VGDSTPVSEKPVSAAVDANASEsP	3	S4(Phospho)	S(1): 0.1; S(2): 0.1; S(4): 8.4; T(9): 8.4; S(12): 82.0; Y(13): 0.9
P46821	VSQGSKDPAEGDGAQPEETPRDGDKPEETQGK	2	S3(Phospho); S8(Phospho)	S(3): 100.0; S(8): 100.0; S(11): 0.0; T(14): 0.0; S(15): 0.0; T(16): 0.0; S(17): 0.0; Y(19): 0.0
P54725	DKDDDGGEDDDANcNLIcGDEYGPETR	1	S4(Phospho)	S(4): 97.7; S(6): 0.3; S(9): 2.0; T(12): 0.0; T(13): 0.0; S(14): 0.0; S(17): 0.0; S(19): 0.0; S(21): 0.0; S(24): 0.0; S(25): 0.0; S(27): 0.0; S(28): 0.0
Q96KC8	sFEVEEVETPNSTPPR	3	S16(Phospho)	S(16): 100.0; T(21): 0.0; T(24): 0.0
P22059	SSKAsLGsLEGEAEAEASSPK	8	S5(Phospho); S8(Phospho)	S(5): 100.0; S(8): 100.0; S(13): 0.0; S(15): 0.0; T(17): 0.0
Q3KQU3	SLSNsNPDISGTPTSPDDEVR	7	S3(Phospho); S6(Phospho)	S(2): 7.8; S(3): 92.2; S(6): 99.9; T(8): 0.1; S(13): 0.0; S(15): 0.0; T(18): 0.0
P49023	GQDVVQEWQETsPTREEPAGEQK	4	S5(Phospho); C-Term(Oxidation)	S(5): 50.0; S(6): 50.0
Q7Z6Z7	RSsQPsPTAVPASDSPPTK	5	S12(Phospho)	S(4): 0.0; S(12): 89.4; S(14): 10.6; T(18): 0.0; S(20): 0.0; S(23): 0.0; T(26): 0.0
P29966	KGAAEEAELEDsDDEEKPVKQDDFPK	1	S18(Phospho)	S(15): 1.1; S(16): 89.3; S(18): 9.6
O95218	HGsGADSDYENTQSGDPLLGLEGK	6	S11(Phospho)	Y(5): 0.0; S(11): 100.0; Y(15): 0.0
Q8IYB3	RADDFPVRDDPsDVtDEDEGPAEPPPPPK	2	S5(Phospho)	T(3): 0.0; S(5): 100.0
O95232	LSAEyEKVELPSEEQVSGSQGPSEEKPAPLATEVFDEK	2	S23(Phospho)	S(2): 0.0; T(4): 0.0; T(11): 0.0; S(12): 0.0; S(17): 0.0; T(21): 0.0; S(23): 100.0
P35251	DYEEVGVDSVEGEGEEEGEEY	1	S5(Phospho); S7(Phospho)	Y(3): 0.0; S(5): 100.0; S(7): 99.9; S(9): 0.1; T(12): 0.0
O43395	REDEGEPGDEGQLEDEGsQEKQGR	2	S16(Phospho)	T(5): 0.0; S(6): 0.0; T(8): 0.0; S(16): 100.0
Q96B36	DSLHEKFPDAGEDELLK	3	S7(Phospho); S15(Phospho)	T(2): 0.0; S(6): 50.0; S(7): 50.0; S(15): 50.0; S(16): 50.0
Q9UHD8	IGDEYAEDssDEEDIR	1	S1(Phospho)	S(1): 100.0; T(9): 0.0; S(12): 0.0; T(13): 0.0
Q9H6Y2	AsLGsLEGEAEAEASSPK	4	C2(Carbamidomethyl); S11(Phospho)	T(1): 0.0; S(11): 100.0; S(18): 0.0; T(23): 0.0
O15234	REESPMDVDQPsPSAQDTQSIASDGTPQGEK	1	S9(Phospho)	S(1): 0.1; T(2): 0.1; T(4): 8.9; S(9): 90.9; S(15): 0.0; T(16): 0.0
Q9Y2X7	DGEERGEEDPEEEHELPVDMETINLDRDAEDVDLNHYR	1	S16(Phospho)	S(1): 0.0; S(3): 0.0; Y(11): 0.0; S(13): 0.0; S(16): 100.0; T(20): 0.0
Q9C0C2	FEEESKEPVADEEEEDsDDDVEPITEFR	4	S3(Phospho); S9(Phospho)	S(3): 100.0; S(9): 99.0; S(11): 1.0

Q32MZ4	HELQANcYEEVKDR	4	C1(Carbamidomethyl); S8(Phospho); C17(Carbamidomethyl)	T(2): 0.0; S(8): 50.0; S(10): 50.0; S(14): 0.0; S(21): 0.0; T(22): 0.0
Q9H1E3	RAsVcAEAYNPDEEEDDAESR	1	S10(Phospho)	Y(4): 0.0; S(5): 0.0; S(10): 100.0; Y(17): 0.0
P23193	APSEEELHGDQTDFGQGSQsPQKQEEQR	1	S11(Phospho)	T(7): 0.0; S(8): 0.0; S(11): 100.0
P49792	KVEEDLKADEPssEEsDLEIDK	1	S7(Phospho)	Y(5): 0.0; S(7): 100.0; T(18): 0.0
Q09666	GPPDFssDEEREPTPVLGSGAAAAGR	5	S5(Phospho); S9(Phospho)	S(3): 98.9; S(5): 1.1; S(9): 90.6; T(11): 9.3; S(13): 0.1
Q5R372	KETKPEPMEEDLPENKK	4	T36(Phospho); C43(Carbamidomethyl)	S(2): 0.0; T(9): 0.0; T(11): 0.0; S(12): 0.0; S(18): 0.0; S(28): 0.9; S(33): 32.1; S(34): 32.1; T(36): 32.1; S(40): 2.8
P42167	EFITGDVEPTDAESEWHsENEEEEKLAGDMk	3	S6(Phospho); S7(Phospho)	S(6): 100.0; S(7): 100.0; T(14): 0.0; S(19): 0.0
Q8IWW6	SHILEDDENsVDISMLK	2	S7(Phospho)	S(5): 0.0; S(7): 100.0; S(12): 0.0; S(13): 0.0; S(14): 0.0; S(15): 0.0; T(16): 0.0
Q5JSH3	SLDsDEsEDEEDDYQQK	1	S12(Phospho)	T(1): 0.0; Y(4): 0.0; S(6): 0.0; S(12): 100.0; S(20): 0.0; T(23): 0.0; T(25): 0.0
Q09666	EGDELEDNGKNFYEsDDDQKEK	1	S5(Phospho); S8(Phospho)	S(1): 50.0; S(2): 50.0; S(5): 72.0; S(8): 28.0; S(18): 0.0; S(19): 0.0
Q9NPQ8	AQNEFKDEAQSLSHsPK	1	S13(Phospho)	Y(12): 50.0; S(13): 50.0; T(18): 0.0; T(20): 0.0; Y(23): 0.0
Q6ZRP7	LLEDsEEsSEETVSR	1	S14(Phospho)	T(6): 0.0; Y(7): 0.0; S(8): 0.0; S(14): 99.4; S(15): 0.6; T(19): 0.0
Q7Z460	HFKDEDEDEDVAsPDGLGR	2	S3(Phospho)	S(3): 99.5; S(4): 0.5; S(6): 0.0; T(8): 0.0; S(12): 0.0; T(13): 0.0
P46821	NQATSAtSEKDNDDDQSDKGTYTIELENPNSEEVEAR	7	S17(Phospho)	S(10): 0.0; S(13): 0.0; T(14): 0.1; T(15): 0.1; S(17): 99.9
Q9UH62	AKIDDPTDSKPEDWDKPEHIPDPDAK	14	S5(Phospho)	Y(1): 0.0; S(5): 100.0; S(11): 0.0; S(14): 0.0
Q99442	cDNDNDcGDNSDEAGcSHScSSTQFK	1	T7(Phospho); C11(Carbamidomethyl)	T(7): 100.0; T(23): 0.0
Q8TEQ0	KPDSENVLGEDESsDDQDMEVNESAQNNLTK	1	S5(Phospho)	S(5): 100.0; Y(19): 0.0; S(22): 0.0; S(23): 0.0
Q9NTJ3	AEsPESSAIESTQSTPQK	2	S3(Phospho)	T(1): 7.0; S(3): 93.0; T(6): 0.0; T(10): 0.0; S(12): 0.0
Q9H063	LFEEsDDKEDEDADGKEVEDADEK	1	S4(Phospho)	S(2): 6.5; S(4): 93.5; S(14): 0.0
Q05655	KETEsEAEDNLDDLEK	3	S6(Phospho)	S(2): 48.0; S(4): 2.0; S(6): 48.0; S(7): 2.0; Y(13): 0.0
P11274	RGPNYTSGYGTNSELSNPsETESER	1	S3(Phospho); C8(Carbamidomethyl)	S(3): 50.0; S(4): 50.0; S(6): 0.0; S(7): 0.0; Y(14): 0.0
O14976	DRWPEVSPEDTQSLSLSEEsPSKETSLDVSSK	13	S24(Phospho)	S(4): 0.0; T(5): 0.0; Y(8): 0.0; S(14): 0.0; T(20): 0.1; S(22): 13.7; S(24): 85.6; S(27): 0.4; S(28): 0.1; S(29): 0.1; S(33): 0.0
Q08J23	VEENPDADsDFDAKssADDEIEETR	2	S14(Phospho); C21(Carbamidomethyl)	S(6): 0.0; S(14): 99.9; T(18): 0.1
Q14498	VDGETAsDSESRAESAPLPVSADDTPEVLNR	5	S3(Phospho)	S(3): 100.0; T(13): 0.0
Q01082	LLKEGEEPTVYsDEEEPKDESAR	6	S30(Phospho)	S(2): 0.0; S(7): 0.0; T(13): 0.0; S(14): 0.0; S(20): 0.0; S(30): 100.0
Q9BWU0	NVRsDIsDQEEDEESEGcPVSINLSK	2	S6(Phospho)	S(6): 100.0; T(14): 0.0; S(15): 0.0
Q92882	DRGLDsGAETEEEKDTWEEK	1	S14(Phospho)	T(1): 0.0; S(3): 0.0; Y(8): 0.0; S(14): 100.0
Q02952	VVDYSQFQEsDDADEDYGRDSGPPTKK	2	S31(Phospho)	S(16): 0.0; T(21): 0.0; S(24): 0.4; T(28): 2.4; S(31): 83.2; T(34): 13.9
O43493	ESLKEEDEsDDDNm	1	S7(Phospho)	S(2): 9.7; S(4): 1.2; S(6): 9.7; S(7): 79.3; T(12): 0.2; T(16): 0.0; S(20): 0.0
P05386	DATNVGDEGGFAPNILENK	3	S7(Phospho)	S(4): 0.0; S(7): 100.0
O95361	LKsEDGVEGDLGETQSR	4	S7(Phospho)	T(2): 0.9; S(7): 90.3; S(9): 8.8
P06396	DSSKGEDSAEEtEAKPAVVAPAPVVEAVSTPSAAFPSDATAENVK	6		
Q8IWS0	LDVKsIDDEDVDENEDDVYGNSSGR	1	S22(Phospho)	T(1): 0.0; S(5): 0.0; S(12): 0.0; S(21): 11.9; S(22): 88.1
Q969E4	SNsSSEAVLGQEELSAQAK	5	S23(Phospho)	S(23): 100.0
Q9H1E3	DTHEDHDTSTENTDESNHDPQFEPIVSLPEQEIK	5	S6(Phospho)	S(2): 0.0; S(6): 100.0; S(9): 0.0
Q147X3	ESLKEEDEsDDDNM	2	C1(Carbamidomethyl); C11(Carbamidomethyl); C12(Carbamidomethyl); S13(Phospho)	S(13): 100.0; S(27): 0.0
Q9C0C2	NYQQNYQNsESGEKNEGSESAPEGQAQQR	4	S3(Phospho)	S(3): 100.0; Y(7): 0.0; S(9): 0.0
Q9NZ63	SQsPAASDcSSSSSSASLPSSGR	5	S5(Phospho); S7(Phospho)	S(5): 100.0; S(7): 100.0; S(14): 0.0
Q66PJ3	HIKEEPLsEEEPcTSTAIASPEK	1	S1(Phospho)	S(1): 100.0; T(12): 0.0
P28715	KEtEsEAEDNLDDLEK	3	S7(Phospho); S8(Phospho)	S(3): 0.0; S(4): 0.0; S(7): 100.0; S(8): 100.0; T(12): 0.0
P04083	RSAsPDDDLGSSNWEAADLGNEER	2		
O00264	SLENETLNKEEDcHsPTSKPPKPDQPLK	4	S12(Phospho)	T(9): 1.1; Y(11): 9.6; S(12): 89.3; S(21): 0.0
P06748	AELEEMEEVHPsDEEEEDTAKAEGFYQK	1		
P08648	sASPHDVDLcLVSPcEFEHR	4	S8(Phospho)	S(4): 0.0; S(5): 0.0; S(7): 0.0; S(8): 50.0; S(9): 50.0; Y(17): 0.0
Q9C0C2	YNDWsDDDDDSNESK	1	S3(Phospho)	S(3): 100.0; S(12): 0.0
Q9H1E3	SQDVAVsPQQQQcSK	1	S10(Phospho)	Y(4): 0.0; S(5): 0.0; S(10): 100.0; Y(17): 0.0; S(21): 0.0; T(25): 0.0
Q3YEC7	GAAEEAELEDsDDEEKPVKQDDFPK	1	S12(Phospho); T15(Phospho)	S(12): 100.0; T(15): 100.0
Q14147	HNIAGTTHSGAEKDVDVSEDsPPPLPER	2	S9(Phospho); S10(Phospho)	S(9): 100.0; S(10): 100.0; S(23): 0.0
Q8WU17	EAEEEssGGEEEDEDENIEVVYSK	2	T18(Phospho)	T(7): 0.0; T(18): 100.0
Q9UKA4	SEHQNSsPTcQEDEEDVR	5	S4(Phospho); C10(Carbamidomethyl); S15(Phospho)	S(4): 98.8; Y(6): 1.2; S(15): 100.0
O14745	SQsGTLDGESAAWSASGEDSR	3	S3(Phospho)	S(1): 49.8; S(3): 49.8; S(4): 0.4; T(6): 0.0; S(7): 0.0; S(12): 0.0; S(15): 0.0
Q6PKG0	HGGSPQPLAtTPLSQEPVNPPSEAsPTR	3	S8(Phospho); S12(Phospho)	T(3): 0.1; T(5): 0.2; S(8): 99.5; S(12): 99.1; Y(14): 1.1
Q99856	SQSDLDDQHDYDSVAsDEDTDQEPLR	2	S11(Phospho); S15(Phospho); S22(Phospho)	S(11): 100.0; S(15): 100.0; S(22): 100.0
P28482	VPssDEEVVEEPQSR	3	Y15(Phospho)	T(9): 0.0; T(13): 0.4; Y(15): 99.6; T(18): 0.0
O43719	EDAEGVAAEEEQEGDsGEQETGATDARPR	1	S7(Phospho); S15(Phospho)	S(7): 100.0; S(15): 100.0; T(24): 0.0; Y(25): 0.0
O94804	IEDsEPHIPLIDDTDAEDDAPTK	2	S10(Phospho)	S(10): 100.0
Q9Y2X7	AAsPPASASDLIEQQQK	1	S3(Phospho)	S(3): 100.0; S(7): 0.0; Y(9): 0.0; T(12): 0.0; S(14): 0.0
O60841	cTLPEHESPsQDISDAcEAESTER	2	S9(Phospho)	S(9): 100.0; S(17): 0.0
Q8NFC6	EAKPGAAEPEVGVPSSLSPSSPsSSWTETDVEER	9	S3(Phospho); S5(Phospho)	Y(1): 0.7; Y(2): 7.4; S(3): 92.0; S(5): 99.9; T(10): 0.0
Q9C0C2	GFKsPPcEDFSVTGESEK	2	S9(Phospho)	S(1): 0.1; T(4): 0.1; S(8): 9.2; S(9): 89.6; S(12): 1.0
Q92574	LAADEDDDDDDEEDDEDDDDDDFDDEEAEEKAPVKK	3	S3(Phospho); C7(Carbamidomethyl)	S(3): 50.0; S(5): 50.0; T(13): 0.0; S(14): 0.0; S(15): 0.0; S(17): 0.0; S(19): 0.0
Q7Z4S6	SSKSAEDLTDGsYDDVLNAEQLQK	5	S4(Phospho)	S(3): 46.5; S(4): 46.5; S(5): 6.9; T(12): 0.2; S(14): 0.0; S(15): 0.0; T(21): 0.0; S(24): 0.0
Q93009	EVEEELATSGGQsPTGEQIPQFQQR	1	S7(Phospho)	S(7): 100.0; T(19): 0.0
Q8IVF2	NEGNLENEGKPEDEVEPDDEGKsDEEEKPDVEGK	3	S6(Phospho); S9(Phospho)	S(6): 95.4; T(8): 52.0; S(9): 52.0; T(10): 0.6; T(12): 0.0; T(17): 0.0
O43583	FTDKDQQPsGsEGEDDDAEAALKK	2	S6(Phospho)	T(2): 0.9; S(6): 99.1; S(14): 0.0; T(19): 0.0
P82094	DAHDVsPTSTDTEAQLTVER	7	S7(Phospho)	S(1): 0.0; S(3): 0.0; S(7): 100.0; S(12): 0.0
Q86WR0	HIVSNDSSDsDDESHEPK	2	S13(Phospho)	S(5): 0.0; S(6): 0.0; S(13): 100.0
O95674	RAEDGSVIDYELIDQDAR	1	T5(Phospho)	T(5): 31.2; S(7): 31.2; S(9): 6.2; S(11): 31.2; S(15): 0.1; S(21): 0.0; T(25): 0.0
Q09666	KPATPAEDDEDDDIDLFGsDNEEEDKEAAQLREER	10	S13(Phospho)	T(10): 0.0; S(13): 100.0
Q9UNF1	TEATQGLDYVPSAGTIsPTSSLEEDKGFK	1	S9(Phospho)	S(9): 90.2; S(10): 9.8; S(13): 0.0; S(16): 0.0; T(18): 0.0; T(19): 0.0
P78559	GRsSESScGVDGDYEDAELNPR	4	S12(Phospho)	T(11): 0.5; S(12): 99.0; T(14): 0.5
Q9Y4K1	TcEERPAEDGsDEEDPDSMEAPTR	3	S3(Phospho); S6(Phospho)	S(3): 94.5; S(6): 52.7; T(7): 52.7; S(9): 0.0; S(19): 0.0
P46821	RSsDPALIGLSTSVSDSNFSSEEPSR	2	S20(Phospho)	S(19): 1.6; S(20): 87.1; T(22): 11.4; Y(25): 0.0
Q02241	LLESSLSsSEGEEPVEYK	5	S3(Phospho)	S(2): 4.9; S(3): 47.6; T(4): 47.6; S(15): 0.0; T(18): 0.0; T(22): 0.0
O60841	IALESEGRPEEQMEsDNcsGGDDDWTHLSSK	1	S4(Phospho)	S(4): 100.0; S(11): 0.0
Q658Y4	EGHSLEMENENLVENGADsDEDDNSFLK	2	S16(Phospho)	S(1): 0.0; S(7): 0.0; S(8): 0.1; T(10): 2.5; T(12): 13.1; T(14): 71.1; S(16): 13.1; T(23): 0.0; S(25): 0.0; S(27): 0.0; S(28): 0.0; S(30): 0.0; S(32): 0.0; T(33): 0.0; T(36): 0.0
Q9NYI0	GLDsGAEtEEEKDTWEEK	4	S6(Phospho); S12(Phospho)	S(1): 91.9; S(3): 8.0; S(4): 0.1; S(6): 0.0; T(11): 8.0; S(12): 91.9; T(15): 0.1
Q9UBB9	NPYLLsEEEDDDVDGDVNVEKNETEPPK	1	S12(Phospho)	S(12): 100.0
P27816	RFsEGVLQSPSQDQEK	3	C2(Carbamidomethyl); S3(Phospho)	S(3): 100.0; S(10): 0.0
Q5XUX1	GLAEVQQDGEAEEGATsDGEKKR	8	S6(Phospho)	T(1): 0.0; S(6): 99.9; S(10): 0.1; T(12): 0.0
Q9Y2X3	VIQWcTHHKDDPPPPEDDENKEK	3	S8(Phospho); C13(Carbamidomethyl)	S(8): 100.0; T(14): 0.0; S(15): 0.0; T(16): 0.0; S(20): 0.0
Q9UEY8	KVAELEEEQSQGsSSYSDWVK	6	S18(Phospho)	S(1): 0.0; S(10): 0.0; S(14): 0.0; S(16): 0.1; S(18): 50.0; S(20): 50.0
P51858	EQEESLGsPVHHSPFDAQTTGDGTEDPSLTALR	1	S6(Phospho); S7(Phospho)	S(6): 100.0; S(7): 100.0
Q9HB20	EAAEPLSEPKEDQEAAELLsEPEEESER	2	S3(Phospho); C8(Carbamidomethyl); C22(Carbamidomethyl)	T(1): 7.9; Y(2): 7.9; S(3): 83.4; T(5): 0.8; S(7): 0.0; S(9): 0.0; S(23): 0.0
Q8NC44	GQKsPGALETPSAAGSQGNTASQGK	2	S5(Phospho)	S(5): 100.0
Q6P158	FDSSLLssDDETK	2	S3(Phospho); C13(Carbamidomethyl); C14(Carbamidomethyl)	S(3): 100.0
P05386	VADPDHDHTGFLTEyVATR	4	S4(Phospho); S7(Phospho)	S(4): 100.0; S(7): 100.0
P78559	NATDLQNSsMSEEELTK	5	S4(Phospho); T8(Phospho)	S(4): 100.0; T(8): 100.0; T(14): 0.0

P24534	NGPLNEsQEDEEDSEHGTSLNR	1	S28(Phospho)	Y(1): 0.0; T(9): 0.0; T(10): 0.0; S(12): 0.0; T(15): 0.0; S(17): 0.3; S(28): 99.7; S(34): 0.0
Q9NZN4	QEEEQDLDGEKGPSsEGPEEEDGEGFSFK	2	S13(Phospho)	S(13): 100.0; T(22): 0.0
Q5JTV8	KPRPsEGDEDcLPASK	1	S5(Phospho); S7(Phospho)	S(5): 91.5; S(7): 91.5; S(8): 17.1; S(14): 0.0; S(15): 0.0; T(17): 0.0; S(20): 0.0; T(22): 0.0; S(24): 0.0
Q13442	EFITGDVEPTDAESEWHsENEEEEKLAGDMK	1	S5(Phospho); S8(Phospho)	S(2): 50.0; S(5): 50.0; S(8): 100.0; Y(15): 0.0
Q9H0D6	KVsVSESNVLLDEEVLTDPK	2	S5(Phospho); S7(Phospho)	S(5): 100.0; S(7): 100.0
Q8NEN9	DVMsDETNNEETESPSQEFVNITK	2	S15(Phospho)	S(11): 0.0; S(13): 50.0; S(15): 50.0
Q96BY6	IAQLEEELEEEQGNTELINDR	2	S5(Phospho); S8(Phospho)	S(2): 0.0; S(5): 100.0; S(8): 100.0; S(11): 0.0; T(12): 0.0
P41236	RGSsGsVDETLFALPAASEPVIR	6	S7(Phospho)	S(6): 9.8; S(7): 90.1; S(12): 0.0; S(15): 0.0
Q09666	TPLSFTNPLHsDDsDSDERNSDGAVTQNK	1	S8(Phospho)	T(6): 0.0; S(8): 99.9; S(12): 0.1; S(14): 0.0; S(16): 0.0
P51114	QGsPDQVsPVSEMTSTSLYQDK	3	S19(Phospho)	Y(5): 0.0; T(6): 0.0; S(7): 0.0; Y(9): 0.0; T(11): 0.0; S(13): 0.0; S(16): 0.0; S(19): 91.7; T(21): 8.2; S(23): 0.0
Q9BTU6	DVEDMELsDVEDDGSK	2	S10(Phospho); S14(Phospho)	S(7): 0.0; S(10): 100.0; S(14): 100.0
P55081	WDEQTSNTKGDDDEEsDEEAVKK	1	S6(Phospho); S7(Phospho)	S(6): 100.0; S(7): 100.0
P07355	NVPQEESLEDsDVDADFK	4		
Q9C0C2	HEVEKSEISENTDASGKIEK	1	S6(Phospho); S7(Phospho)	S(2): 0.0; S(6): 100.0; S(7): 100.0; S(17): 0.0
P78559	AMDNHsDsEEELAAFcPQLDDSTVAR	3	S13(Phospho)	T(2): 0.0; S(3): 0.0; S(9): 0.0; S(10): 0.0; S(13): 100.0
P24534	sLSEEVESSESGELPEVDVK	4	S17(Phospho); S28(Phospho)	Y(1): 0.0; T(9): 1.1; T(10): 2.7; S(12): 7.5; T(15): 21.9; S(17): 67.1; S(28): 98.9; S(34): 0.7
Q7Z5K2	sPSEARQDVDLcLVSScEYK	2	S14(Phospho)	S(5): 0.0; T(6): 0.0; S(14): 99.8; S(18): 0.2; S(22): 0.0; S(23): 0.0
Q9NTI5	NLGsINTELQDVQR	2	S3(Phospho)	S(3): 100.0; S(6): 0.0; S(7): 0.0; S(11): 0.0; T(12): 0.0; S(14): 0.0; T(15): 0.0
Q969E4	AEGLEEADTGASGcHSHPEEQPTsIsPSR	2	C3(Carbamidomethyl); S24(Phospho)	T(1): 0.0; S(24): 100.0
Q2M389	HPEPVPEEGsEDELPPQVHKV	4	S9(Phospho)	T(6): 95.4; T(8): 2.3; S(9): 2.3; S(14): 0.0; S(16): 0.0; T(17): 0.0; S(19): 0.0
O95359	DNLLDTYSADQGDSsEGGTLAR	5	S7(Phospho); S11(Phospho)	T(4): 0.0; S(7): 100.0; S(11): 100.0
Q02880	TEDGDWEWsDDEMDEKSEEGK	1	S7(Phospho); S9(Phospho)	S(7): 100.0; S(9): 100.0; S(11): 0.0
Q9Y4E8	NSSLLsFDNEDENE	2	S12(Phospho); C28(Carbamidomethyl)	S(3): 0.0; T(6): 0.0; S(12): 19.2; S(16): 80.8; T(31): 0.0
Q9BWU0	GLDsGAETEEEKDTWEEK	4	S7(Phospho)	S(7): 100.0; T(15): 0.0; S(16): 0.0
Q8NI22	SAsSDTSEELNSQDSPPK	3		
Q96TC7	EDKsPSEESAPTTSPESVSGSVPSSGSSGR	7	S3(Phospho)	S(1): 10.7; S(3): 89.1; S(7): 0.2; Y(10): 0.0; T(11): 0.0; T(13): 0.0; S(14): 0.0
Q9Y4B5	GLMAGGRPEGQYsEDEDtDTDEYKEAK	1	S9(Phospho)	S(9): 100.0; S(11): 0.0
Q96QR8	GKGGVTGsPEASISGSK	3	S11(Phospho)	S(5): 0.0; S(11): 100.0
O43815	DSDQVAQsDGEESPAAEEQLLGEHIK	3	S10(Phospho)	S(4): 0.0; S(10): 100.0
Q3KQU3	TDATDGKDYNASASTIsPPSSMEEDKFSR	3	S3(Phospho); S6(Phospho); S13(Phospho)	S(2): 9.3; S(3): 91.5; S(6): 90.7; T(8): 8.5; S(13): 0.9; S(15): 99.0; T(18): 0.1
Q9Y2X7	TGEEDKKINEELESQYQQSMDSK	3	S13(Phospho); S16(Phospho)	S(1): 0.0; S(3): 0.0; Y(11): 15.6; S(13): 84.9; S(16): 98.7; T(20): 0.7
P46821	KPDGVKEsTESSNTTIEDEDVK	6	S11(Phospho); C23(Carbamidomethyl); S25(Phospho)	S(2): 0.0; S(11): 99.9; T(15): 84.4; S(25): 15.7
Q86VM9	EGGGVHAVPPDPEDEGLEETGSKDKDQPPSPsPPPQSEALSSTSR	2	S16(Phospho)	S(2): 0.0; S(4): 0.0; S(16): 100.0; S(23): 0.0
Q9NTJ3	GPDEAMEDGEEGsDDEAEWVVTK	2	S10(Phospho); S15(Phospho)	S(10): 100.0; S(15): 91.2; S(16): 8.8; S(24): 0.0
Q9NSY0	GAGDGsDEEVDGKADGAEAKPAE	2	S8(Phospho); C20(Carbamidomethyl)	S(8): 100.0; S(12): 0.0; S(18): 0.0
Q02952	GDQPAASGDsDDDEPPPLPR	9	C5(Carbamidomethyl); S18(Phospho)	T(4): 0.0; S(7): 0.0; T(12): 0.0; S(18): 100.0
Q9Y490	NRsAEEGELAESK	2	S21(Phospho)	S(1): 0.0; S(13): 0.0; T(14): 0.0; S(19): 0.1; S(21): 99.9
Q5JTV8	ADHRSsPNVANQPPsPGGK	1	S4(Phospho)	S(4): 100.0; T(9): 0.0; S(16): 0.0; S(17): 0.0; S(19): 0.0; S(20): 0.0; T(22): 0.0; T(23): 0.0
Q5T8D3	LPSGsGAAsPTGSAVDIR	7	S6(Phospho); S10(Phospho)	S(3): 0.1; S(4): 0.4; S(6): 99.5; S(10): 100.0
Q92900	MLPHAPGVQMQAIPEDAVHEDsGDEDGEDPDKR	2	S8(Phospho)	S(1): 0.0; S(8): 88.4; S(11): 10.2; T(12): 1.3; Y(13): 0.2
P67809	HsTSGTDEGEDGDEPDDGSNDVVDLLPR	7	S11(Phospho)	Y(2): 0.0; Y(6): 0.0; S(9): 1.6; S(11): 86.4; S(18): 0.2; S(20): 11.7
O75382	HDSIPAADTFEDLsDVEGGGSEPTQR	2	S4(Phospho)	S(4): 100.0
Q9H3N1	EDGNEEDKENQGDETQGQQPPQR	1	S13(Phospho)	S(13): 100.0; S(19): 0.0
Q13439	VLDTSSLTQsAPAsPTNK	1	S6(Phospho)	S(6): 100.0; T(16): 0.0; S(17): 0.0
Q9NXG2	DIKPQLELIEDEEKLKETEPVEAYVIQK	2	S9(Phospho); S11(Phospho)	T(2): 0.0; S(9): 100.0; S(11): 100.0
Q86W92	VVDYSQFQEsDDADEDYGRDSGPPTK	1	S3(Phospho)	S(1): 0.5; S(3): 92.4; T(4): 6.6; T(5): 0.5; S(12): 0.0
Q92769	NWTEDMEGGISsPVKK	2	S22(Phospho)	S(22): 100.0
Q93008	GTEAPAVVtEEEDDDEETAPPVIAPRPDHTK	3	S18(Phospho)	T(9): 1.9; S(11): 0.6; S(18): 95.6; S(27): 1.9
Q96RK0	IEDVGsDEEDDSGK	1	T4(Phospho); S15(Phospho)	S(3): 99.0; T(4): 1.0; S(8): 0.0; T(14): 50.0; S(15): 50.0
Q6P6C2	NPDDITNEEYGEFYK	1	S5(Phospho)	Y(1): 3.0; S(5): 96.2; S(10): 0.6; Y(12): 0.1
Q9UPT8	NKDQGTYEDYVEGLR	2	T13(Phospho); S21(Phospho)	S(11): 49.9; T(13): 49.9; S(15): 0.2; S(17): 0.0; S(21): 100.0; T(25): 0.0; Y(28): 0.0
P42167	SRPTsEGsDIESTEPQK	1	S7(Phospho)	S(6): 1.4; S(7): 98.5; T(14): 0.2; S(19): 0.0
O75122	LSPHAFKTEsGEETDLISPPQEEVK	5	S3(Phospho)	S(1): 0.2; S(3): 99.8
P63104	NVPHEDIcEDsDIDGDYR	4		
O75396	KsPSEARQDVDLcLVSScEYK	2	S4(Phospho)	S(4): 99.5; T(7): 0.5
Q9BVS4	YGPADVEDTTGSGATDSKDDDDIDLFGsDDEEESEEAK	3	S8(Phospho)	S(3): 0.0; S(6): 0.2; S(8): 99.8
Q969E4	RQsSGSATNVASTPDNR	5	S5(Phospho)	T(2): 0.0; S(5): 92.5; S(9): 7.5
P46821	GSHQIsLDNPDYQQDFFPK	1	S6(Phospho)	T(1): 0.0; T(2): 0.0; S(4): 0.3; S(6): 99.7; S(8): 0.0; Y(10): 0.0; S(11): 0.0; Y(12): 0.0; T(14): 0.0
Q92882	KIQALQQQADEAEDR	1	S3(Phospho); S14(Phospho)	T(1): 7.6; S(3): 92.4; Y(8): 0.0; S(14): 100.0
Q6P2E9	RFsFccSPEPEAEAEAAAGPGPcER	2	S10(Phospho)	S(2): 0.0; S(6): 1.5; S(10): 98.5; S(18): 0.0; S(21): 0.0; S(23): 0.0; T(28): 0.0
P31946	GLRDsHsSEEDEASSQTDLSQTISK	2		
Q969E4	cPFPAGAALAccsEDEEDDEEHEGGGSR	1	S17(Phospho)	S(17): 100.0
P27348	NKEDQYDHLDAADMTK	3		
Q9C0C2	KREEGsDIEDEDMEELLNDTR	1	S1(Phospho)	S(1): 100.0
Q9UNZ2	DNLTLWTSDQQDDDGGEGNN	7		
Q08J23	TEPHDSDcSVDLGIsKSTEDLSPQK	3	S6(Phospho); S14(Phospho); C21(Carbamidomethyl)	S(6): 100.0; S(14): 100.0; T(18): 0.0
Q13283	QEEEQDLDGEKGPSSEGPEEEDGEGFSFKYsPGK	2	S3(Phospho)	S(1): 0.5; S(2): 0.5; S(3): 99.1; T(12): 0.0
P08651	AKsPQPPVEEEDEHFDDTVVcLDTYNcDLHFK	1	S12(Phospho)	T(3): 0.0; S(11): 9.4; S(12): 90.6
Q14008	DEFTNTcPSDKEVEIAYSDVAK	3	T3(Phospho)	S(2): 6.9; T(3): 31.0; S(4): 31.0; T(6): 31.0; S(19): 0.0
Q9H1E3	DHQEVETEGPESADTGDKSESPDEANVGKHPK	1	S6(Phospho); S9(Phospho)	S(2): 0.0; S(6): 100.0; S(9): 100.0
Q9NYF8	HPEPVPEEGsEDELPPQVHK	2	S4(Phospho)	S(4): 100.0; T(9): 0.0; T(12): 0.0; Y(15): 0.0
Q15154	DSIEKEHVEEISELFYDAK	4	S6(Phospho); S9(Phospho)	T(2): 0.0; S(6): 100.0; S(9): 92.6; S(10): 7.4
Q9BPX3	KLEEEQIILEDQNcK	1	C1(Carbamidomethyl); S8(Phospho); S10(Phospho)	T(3): 0.2; S(8): 99.9; S(10): 99.9; S(19): 0.0
O60231	GDsEsEEDEDLEVPVPSR	2	S5(Phospho); S8(Phospho)	S(5): 100.0; S(8): 92.3; S(9): 7.7; T(12): 0.0; S(14): 0.0
Q5JTV8	KAEQGsEEEGEGEEEEEEGGESK	1	S4(Phospho); S5(Phospho)	S(2): 87.5; S(4): 87.5; S(5): 25.0; S(11): 0.1; S(12): 0.0; T(14): 0.0; S(17): 0.0; T(19): 0.0; S(21): 0.0
P27348	MPLLELGGETtPPLStERSPEAVGSEcPSR	4	S10(Phospho); C15(Carbamidomethyl)	T(4): 0.0; T(7): 9.1; S(8): 1.0; S(10): 90.0
P46821	TASESISNLSEAGsIK	2	T4(Phospho)	T(1): 0.1; T(2): 0.7; T(4): 99.2; Y(10): 0.0; S(11): 0.0; Y(12): 0.0
P49815	EGQPSPADEKGNDsDGEGESDDPEKK	2	T5(Phospho)	S(1): 9.7; S(3): 9.7; T(5): 80.6; S(10): 0.0; S(14): 0.0; S(16): 0.0; S(20): 0.0
O95218	YSHSYLsDsDTEAKLTETNA	2	S7(Phospho)	S(7): 100.0; S(19): 0.0
Q9Y5X1	YEDKPEPEVDALGsPPALLK	1	S1(Phospho)	S(1): 100.0; S(6): 0.0; S(7): 0.0
Q86TN4	LPEGPVDsEDDEEEDEEIDRTDPLQGR	2	C13(Carbamidomethyl); S16(Phospho)	S(4): 0.0; T(11): 0.0; S(15): 0.4; S(16): 99.6
P35613	KGNAEGssDEEGKLVIDEPAK	2	S19(Phospho)	S(14): 0.1; S(19): 86.4; S(20): 13.6
Q8NE71	KKEPAITSQNsPEAR	5	S5(Phospho)	S(5): 100.0; S(21): 0.0; Y(27): 0.0; S(31): 0.0
Q9Y6G9	SSsPAPADIAQTVQEDLR	20	T9(Phospho); T11(Phospho)	T(4): 0.0; S(6): 0.9; T(8): 32.9; T(9): 95.1; T(11): 35.6; S(12): 35.6; T(14): 0.0; S(19): 0.0
P78346	VQENDGKEPPPVVNyEEDAR	2	S5(Phospho); C11(Carbamidomethyl)	S(5): 100.0; S(15): 0.0
P46821	mEQLsDEEIDHGAEEDsDKEDQDLDK	2	S5(Phospho); C19(Carbamidomethyl)	S(5): 99.9; T(11): 0.0; T(12): 0.0; T(13): 0.0; T(22): 0.0
Q8TE77	KcsLPAEEDSVLEK	1	S20(Phospho)	S(3): 0.0; T(12): 0.0; S(18): 2.2; S(20): 97.8
P18615	FGTEELFKDEATDGGGDNKEGEDSSVIHYDDK	1	S3(Phospho)	S(1): 0.5; S(3): 99.5; S(11): 0.0; S(12): 0.0

Q9BV36	SILKPSTPIPPQEGEEVGEsSEEQDNAPK	4	T9(Phospho); C14(Carbamidomethyl); S28(Phospho)	T(9): 0.6; S(12): 3.0; S(16): 96.5; T(23): 0.1; S(24): 2.5; S(26): 83.3; S(28): 14.1
Q13442	RSDSASsEPVGIYQGFEK	5	S4(Phospho); S7(Phospho)	S(1): 8.2; S(4): 91.8; S(7): 100.0; Y(14): 0.0
Q9HB90	DYHVEcYHcEDcGLELNDEDGHR	2	S11(Phospho)	S(2): 0.0; T(6): 0.0; S(11): 93.7; T(12): 6.3
O94804	SVTEQGAELSNEER	1	S4(Phospho); S8(Phospho)	S(2): 50.0; S(4): 50.0; S(8): 91.0; S(9): 9.0; T(13): 0.0
Q15019	DGSGDSHPDFPEDADIDLKDVDK	5	S9(Phospho)	Y(2): 0.0; S(9): 100.0; T(19): 0.0
Q9NP74	YKDLDEDELLGNLsETELK	4	S7(Phospho); C10(Carbamidomethyl)	S(1): 0.0; S(6): 49.8; S(7): 49.8; T(9): 0.3
Q15311	NRPDYVsEEEEDDEDFETAVK	2	S11(Phospho); S12(Phospho)	T(1): 0.1; Y(4): 0.1; S(11): 99.8; S(12): 99.8; S(18): 0.1; S(20): 0.1
O95817	AVsDSFGPGEWDDR	1	S3(Phospho); C9(Carbamidomethyl)	S(1): 50.0; S(3): 50.0; S(7): 0.0; S(10): 0.0; S(11): 0.0; S(12): 0.0; S(13): 0.0; S(14): 0.0; S(15): 0.0; S(17): 0.0; S(20): 0.0; S(21): 0.0
Q05209	NQTAEKEEFEHQQK	4	S11(Phospho); S14(Phospho)	T(1): 0.0; S(4): 0.0; T(6): 0.4; S(11): 99.6; S(14): 99.9; S(16): 0.1; S(21): 0.0; T(26): 0.0
Q7Z2K8	DcSYGAVTsPTSTLESR	1	S5(Phospho); C13(Carbamidomethyl)	S(3): 0.5; S(5): 99.5; S(12): 0.0; S(14): 0.0
P25788	IYHLPDAEsDEDEDFKEQTR	7	S9(Phospho)	S(2): 0.0; S(9): 100.0
Q9NX63	KAAQQQEEQEEKEEEDDEQTLHR	2	S5(Phospho)	S(1): 0.4; Y(4): 49.8; S(5): 49.8; Y(8): 0.0; S(11): 0.0; S(13): 0.0
Q6WCQ1	HRsPSGAGEGAScSDGPR	2	S9(Phospho); S13(Phospho)	S(6): 0.0; S(7): 1.6; S(9): 98.4; S(13): 98.4; S(15): 1.6; S(17): 0.0; S(23): 0.0; S(24): 0.0
O43852	ALVEFESNPEETREPGsPPSVQR	2	S7(Phospho)	S(7): 100.0; Y(10): 0.0
O95155	ALDSNsLENDDLSAPGREPGHFNPESR	3	S3(Phospho); C11(Carbamidomethyl)	S(1): 0.1; S(3): 99.9; S(10): 0.0
P08238	RDsLGAYASQDANEQGQDLGKR	1	S6(Phospho)	S(6): 100.0; S(12): 0.0
Q12959	GSGTAsDDEFENLR	1		
Q9ULT8	NKDQSEQETSDADQHVTSNAsDSESSYR	2	S9(Phospho)	S(2): 0.0; T(3): 0.0; S(8): 12.2; S(9): 87.8; S(17): 0.0
Q96N67	IQQFDDGGsDEEDIWEEK	3	S5(Phospho)	S(1): 7.0; S(3): 7.0; S(5): 86.0; S(10): 0.0; T(12): 0.0; T(14): 0.0; S(15): 0.0
P35568	TDLEKDIISDTSGDFRK	1	S3(Phospho); S7(Phospho)	T(1): 0.9; S(3): 99.1; T(6): 8.3; S(7): 90.7; T(9): 0.8; T(11): 0.1
P46821	EKEEETKtSNGDLSDSTVSADPVVK	3	S4(Phospho); S6(Phospho)	T(1): 0.5; T(2): 0.5; S(4): 47.2; S(6): 47.2; S(8): 5.5; Y(10): 89.2; S(11): 8.9; Y(12): 0.9; T(14): 0.0
Q7Z6Z7	TKEYVSNDAAQsDDEEKLQSQPTDTDGGR	2	S4(Phospho); T5(Phospho)	S(3): 52.7; S(4): 52.7; T(5): 94.7
P24534	GTDDsPKDSQEDLQER	6	S28(Phospho)	Y(1): 0.0; T(9): 0.0; T(10): 0.0; S(12): 0.0; T(15): 0.0; S(17): 0.0; S(28): 100.0; S(34): 0.0
P78559	AGEPNsPDAEEANSPDVTAGcDPAGVHPPR	5	S12(Phospho)	T(11): 1.2; S(12): 98.7; T(14): 0.1
Q02878	DMQGLsLDAASQPSK	1		
O94979	SSEPVKETVQTTQsPTPVEK	1	S8(Phospho)	S(2): 0.0; S(8): 99.8; S(13): 0.2
Q9UPN3	DHSSQSEEEVVEGEKEVEALKK	4	S3(Phospho); S6(Phospho); C17(Carbamidomethyl)	S(3): 100.0; S(6): 100.0; T(13): 0.0; S(15): 0.0; S(18): 0.0; T(20): 0.0; S(21): 0.0; S(23): 0.0; S(24): 0.0; S(32): 0.0
Q13043	REEDEPEERsGDETPGSEVPGDK	1	S10(Phospho)	S(10): 100.0; S(17): 0.0; T(19): 0.0
P29692	NKHEAMITDLEER	4	T4(Phospho); S19(Phospho)	T(4): 100.0; S(19): 100.0
P48634	EKPELSEPSHLNGPSsDPEAAFLSR	2	T1(Phospho); S7(Phospho); S10(Phospho)	T(1): 90.0; S(3): 10.0; T(5): 0.2; S(7): 99.8; S(10): 100.0; Y(12): 0.0
Q9Y6X9	DsLGAYASQDANEQGQDLGK	2	S5(Phospho)	S(1): 0.0; S(5): 100.0
O95684	TTKtPEDGDYSYEIIEK	2	S18(Phospho); S22(Phospho)	T(5): 0.0; T(6): 0.0; S(14): 16.2; S(18): 86.2; S(22): 97.6
Q13435	VENMSSNQDGNDsDEFM	2	S9(Phospho); S13(Phospho); S14(Phospho)	S(9): 100.0; S(13): 100.0; S(14): 100.0
P78559	AGAAAGDsDEESRADDKGVMDYYLK	1	T17(Phospho)	T(17): 50.0; S(18): 50.0
P67809	KSEDSLLRNsDEEESASESELWK	2		
Q9BTC0	SRsLsNSNPDISGTPTSPDDEVR	5	S10(Phospho); S14(Phospho)	S(10): 100.0; S(14): 100.0; S(16): 0.0; S(22): 0.0
P84157	GPPDFsSDEEREPTPVLGSGAAAAGR	4	S15(Phospho)	S(14): 2.0; S(15): 98.0; S(27): 0.0
Q7Z460	SNNWsLEDVTASDK	1	S3(Phospho)	S(1): 0.3; S(3): 99.7; S(12): 0.0
Q96B23	sSKASLGSLEGEAEAEASSPK	4	S3(Phospho)	S(3): 88.0; S(4): 10.6; S(6): 1.4; S(10): 0.0; T(11): 0.0; S(13): 0.0; T(17): 0.0; T(18): 0.0
Q5VTR2	KKDQVTAQEIFQDNHEDGPTAK	1	S14(Phospho)	S(12): 0.5; S(14): 99.5
Q13547	IQEQEssGEEDSDLSPEER	3	C3(Carbamidomethyl); S8(Phospho); S10(Phospho)	S(8): 100.0; S(10): 100.0
Q8TDB6	VSTLAGPSsDDENEEESKPEKEDEPQEDAK	2	S13(Phospho)	T(7): 0.0; S(13): 100.0; S(16): 0.0
Q9NXG2	TPGVGKEDAAEETVKPGPEEGTLEKEEK	1	S9(Phospho); S11(Phospho)	T(2): 0.0; S(9): 100.0; S(11): 100.0
Q8N573	SVTVVEDDEDEDGDDLLHHHHVSGSR	6	S3(Phospho); S4(Phospho)	S(3): 92.6; S(4): 50.0; T(5): 50.0; S(6): 7.4; T(13): 0.0
P78559	KGGSYSQAASSDSAQGsDVsLTAcKV	1	T8(Phospho)	S(3): 0.0; S(4): 0.0; T(8): 100.0
Q32MZ4	SQsTTFNPDDMSEPEFK	8	C7(Carbamidomethyl); S14(Phospho); C23(Carbamidomethyl)	S(1): 0.0; T(8): 0.2; S(14): 98.8; S(16): 1.0; S(20): 0.0; S(27): 0.0; T(28): 0.0
Q9C0C9	SRDEDNDEDEERLEEEEQNEEEEVDN	2	S7(Phospho); S9(Phospho)	S(7): 100.0; S(9): 100.0
P24534	scETLEGPQTVDTWPR	1	S10(Phospho)	S(10): 100.0; S(16): 0.0
P49023	HRPsEADEEELAR	1	S13(Phospho); C15(Carbamidomethyl)	T(1): 0.0; S(2): 0.0; S(3): 0.0; S(5): 0.0; S(10): 0.0; S(13): 92.7; S(16): 7.3
Q15678	KLGDVSPtQIDVSQFGsFKEDTK	1	S5(Phospho); S6(Phospho)	Y(1): 0.0; S(3): 0.0; S(5): 100.0; S(6): 100.0; T(11): 0.0
Q9H1E3	APsRKDsLESDSSTAIIPHELIR	1	S11(Phospho)	Y(5): 0.0; S(6): 0.0; S(11): 100.0; Y(18): 0.0
Q9Y487	VEAAEIVEGEDSAYSVR	2	S3(Phospho); C26(Carbamidomethyl)	S(3): 100.0; S(8): 0.0; S(12): 0.0
O94929	RREEGPPPPsPDGAsSDAEPEPPSGR	4	S3(Phospho); S4(Phospho)	S(3): 100.0; S(4): 100.0
P05388	DIKPQLELIEDEEK	1	S7(Phospho); S10(Phospho); C-Term(Oxidation)	S(7): 100.0; S(10): 100.0
P60660	DSNPEDSGEGKKESLESEAELEGLQDAPAGPQVSEE	2		
Q9Y5S2	TEATQGLDYVPSAGTIsPTsSLEEDKGFK	1	S15(Phospho); S18(Phospho)	S(2): 0.0; T(3): 0.0; S(5): 0.0; S(7): 0.0; S(8): 0.0; S(11): 0.0; S(15): 100.0; S(18): 100.0
Q9Y3P9	VDPSLMEDsDDGPSLPTK	1	T7(Phospho)	T(7): 100.0; T(13): 0.0
O60292	KTsLVIVESADNQPETcER	2	S3(Phospho); C10(Carbamidomethyl)	S(1): 33.3; S(2): 33.3; S(3): 33.3; T(6): 0.1; S(8): 0.0
P35606	GDSKKDDEENYLDLFSHK	2	S13(Phospho)	S(1): 0.5; T(2): 0.5; S(13): 84.2; T(15): 14.8; S(21): 0.0; T(23): 0.0
Q06210	TYsDTDScSDIPLEDPDRPVHcSK	6	S3(Phospho); C6(Carbamidomethyl)	S(3): 99.6; T(4): 0.4; T(5): 0.0
Q4G0J3	KLEKEEEEGISQEssEEEQ	2	S5(Phospho); S8(Phospho)	S(1): 7.6; T(4): 99.4; S(5): 93.0; S(8): 0.0; S(12): 0.0; T(13): 0.0
Q3YEC7	QALDsEEEEEDVAAK	1	S11(Phospho); T14(Phospho)	S(11): 100.0; T(14): 100.0
P06733	RKPDYEPVENTDEAQKK	3		
Q15637	SLMssPEDLTK	3	S14(Phospho); S16(Phospho)	T(1): 0.0; S(14): 99.8; S(16): 99.8; Y(21): 0.3; S(23): 0.2
P07900	LKEGHETPMDIDsDDSK	1		
Q03001	EVEDKEsEGEEEDEDEDLSK	2	C1(Carbamidomethyl); S10(Phospho)	S(2): 0.0; S(4): 0.0; S(5): 0.0; S(6): 0.0; S(10): 100.0; S(16): 0.0
Q12872	QVAEQGGDLsPAANR	2	S5(Phospho)	S(1): 2.7; S(4): 14.5; S(5): 82.8; Y(18): 0.0; S(22): 0.0; S(26): 0.0
Q09666	AEGLEEADTGASGcHSHPEEQPTSIsPSR	5	S8(Phospho)	Y(3): 0.2; T(6): 11.0; S(8): 88.8; T(12): 0.0; S(18): 0.0; S(21): 0.0; S(24): 0.0
Q3KQU3	GASAATGIPLEsDEDSNDNDNDIENENcMHTN	4	S12(Phospho); S16(Phospho); S20(Phospho)	S(2): 0.0; S(7): 0.0; S(12): 100.0; S(16): 100.0; S(20): 50.0; T(22): 50.0
Q15751	TQtPPVSPAPQPTEER	1	S1(Phospho)	S(1): 33.3; S(3): 33.3; S(5): 33.3; S(8): 0.0; S(12): 0.0; Y(17): 0.0; S(20): 0.0
P13861	SGSssPDSEITELKFPSINHD	2	S8(Phospho); S10(Phospho)	S(8): 100.0; S(10): 100.0; S(22): 0.0
Q9NRH2	KGsDALRPPVPQGEDEVPK	1	S3(Phospho)	S(3): 85.7; S(4): 14.3; S(10): 0.0; S(18): 0.0; S(21): 0.0; S(24): 0.0
P46821	AFyGSEEDEAKGAGSEETAAAAAPSR	2	S16(Phospho)	S(9): 0.0; S(12): 0.0; T(13): 1.7; T(14): 11.9; S(16): 86.4
Q9UHI6	DLIHDQDEDEEEEEGQR	1	S7(Phospho)	S(1): 0.0; Y(2): 0.0; S(6): 0.8; S(7): 99.2; S(14): 0.0; S(16): 0.0; T(17): 0.0
P46821	NSQEDsEDSEDKDVK	1	T16(Phospho)	Y(11): 0.0; T(15): 1.0; T(16): 89.9; T(18): 9.1
Q9Y4E8	GFEEEHKDsDDDssDDEQEKKPEAPK	1	S12(Phospho); S16(Phospho); C28(Carbamidomethyl)	S(3): 0.0; T(6): 0.2; S(12): 99.7; S(16): 98.8; T(31): 1.2
Q02952	GILAADEsTGSIAK	1	S17(Phospho)	T(16): 0.1; S(17): 99.9
Q13435	ARQEEGADAsEEDPTPAGEEDVKDAR	1	S6(Phospho); S8(Phospho)	S(1): 0.3; S(2): 0.3; S(6): 99.4; S(8): 98.4; T(10): 1.7; T(14): 0.0; S(16): 0.0; S(18): 0.0
Q13557	DVMSDETNNEETESPSQEFVNITK	1	S8(Phospho)	S(8): 94.1; T(9): 5.9; S(11): 0.0; S(12): 0.0; T(14): 0.0; T(15): 0.0
Q5UIP0	WPEVSPEDTQSLSLSEESPsKETSLDVSSK	3	S4(Phospho)	S(1): 0.0; S(4): 100.0
O15013	KNsITEISDNEDDLLEYHR	2	S18(Phospho)	Y(1): 0.0; T(3): 0.0; S(18): 100.0
O00203	SVSEINsDDELSGK	1	S15(Phospho)	Y(13): 0.3; S(15): 99.7
Q12982	QEKPAEKPAETPVATsPTATDSTSGDSSR	15	S3(Phospho)	S(3): 99.3; T(5): 0.7; Y(7): 0.0; T(8): 0.0
Q96JM3	TVAISDAAQLPHDYcTtPGGtLFSTTPGGTR	1	S6(Phospho)	S(5): 8.9; S(6): 91.0; S(11): 0.1; S(12): 0.0; S(13): 0.0; Y(15): 0.0
P23588	IADPEHDHTGFLTEyVATR	2	S3(Phospho)	T(1): 50.0; S(3): 50.0; S(5): 0.0; S(6): 0.0; T(8): 0.0; T(10): 0.0; S(11): 0.0; T(12): 0.0; T(13): 0.0; S(14): 0.0; S(15): 0.0
P49585	DGHDQHVLELEAK	1	S6(Phospho); S10(Phospho); S12(Phospho)	S(6): 100.0; S(10): 99.2; S(12): 0.9; S(13): 7.9; S(14): 91.2; T(16): 0.7
Q15052	GGIDNPAITsDQELDDKK	1	T2(Phospho)	S(1): 50.0; T(2): 50.0
P30533	tASGSSVTsLDGTR	5	S7(Phospho)	S(2): 0.0; Y(6): 0.9; S(7): 90.6; T(8): 8.5

Q9BQ70	TESPAtAAETASEELDNR	9	T3(Phospho)	T(3): 0.3; S(5): 1.8; T(7): 97.7; T(12): 0.3; T(14): 0.0; S(17): 0.0; S(20): 0.0; S(23): 0.0
P78317	AVSETPAVPPVsEDEDDDDDATPPPVIAPRPEHTK	1	C10(Carbamidomethyl); S13(Phospho); S14(Phospho)	S(9): 0.0; S(13): 100.0; S(14): 100.0; S(20): 0.0
P04075	RTEARSsDEENGPPsSPDLDR	1	S8(Phospho)	S(8): 93.0; T(9): 6.9; S(11): 0.0
Q9NPQ8	RPQFsPTGDcQAEAAAAANGTGGEEDDGPAAELLEK	2	S13(Phospho); T18(Phospho)	Y(12): 83.2; S(13): 17.5; T(18): 82.4; T(20): 16.8; Y(23): 0.2
O00203	RGsIGENQVEVMVEEK	2	S5(Phospho)	Y(3): 0.0; S(5): 100.0
P41236	NYAGEEEEEGSGsSEGFDPPATDR	2		
Q8N3D4	ADALQAGASQFETsAAK	1	S3(Phospho)	S(3): 100.0
Q13557	DSQEEEKTEALTSAK	3	T9(Phospho); S12(Phospho)	S(8): 99.2; T(9): 8.1; S(11): 84.5; S(12): 7.4; T(14): 0.7; T(15): 0.1
Q07954	MSPNETLFLEsTNK	2	C1(Carbamidomethyl); C7(Carbamidomethyl); C16(Carbamidomethyl); C20(Carbamidomethyl)	
Q7Z417	sTAALEEDAQILK	2	S7(Phospho)	S(7): 89.8; S(9): 9.1; S(11): 1.0; Y(13): 0.1; T(14): 0.0; T(15): 0.0
Q5JSZ5	STSAPQMSPGsSDNQSSSPQPAQQK	2	S4(Phospho)	S(4): 100.0
P17096	DKAPVQPQQsPAAAPGGTDEKPSGK	2	S14(Phospho); S15(Phospho)	S(11): 0.0; S(14): 100.0; S(15): 100.0
Q6P5R6	VAHEPVAPPEDKESESEAKVDGETAsDSESR	5	S11(Phospho)	Y(1): 0.0; S(5): 0.0; S(11): 100.0; S(13): 0.0
P04083	DSDQVAQsDGEESPAAEEQLLGEHIKEEK	2		
P01891	GPAEsPDEGITTTEGEGEcEQTPEELEPVEK	1	S13(Phospho); S17(Phospho); S20(Phospho); C24(Carbamidomethyl)	S(4): 0.0; Y(5): 0.0; S(6): 0.0; S(10): 10.6; S(11): 10.6; S(13): 78.8; S(17): 100.0; S(20): 100.0; T(22): 0.0
Q92614	GQsQLsNPTDDSWK	1	S3(Phospho)	S(1): 8.3; T(2): 8.3; S(3): 83.3; S(11): 0.0; T(17): 0.0
Q86WR0	GsDEENLDSETSASTESLLEER	1	S13(Phospho); C-Term(Oxidation)	S(5): 0.0; S(6): 0.0; S(13): 100.0
Q7Z3C6	MPQDGsDDEDEEWPTLEK	3	S10(Phospho)	S(10): 100.0
O00264	IEEVLSPEGSPSKsPSK	1	S10(Phospho)	S(7): 0.0; S(10): 100.0
Q6VMQ6	TAHNSEADLEESFNEHELEPSsPK	4	C9(Carbamidomethyl); S13(Phospho)	S(13): 14.8; T(18): 84.6; S(23): 0.6
P01891	DLHDDDEDEEMLETADGESMNTEESNQGSTPSDQQQNK	1	S17(Phospho); S20(Phospho); C24(Carbamidomethyl)	S(4): 0.0; Y(5): 0.0; S(6): 0.0; S(10): 0.0; S(11): 0.0; S(13): 0.0; S(17): 99.9; S(20): 97.7; T(22): 2.4
P04920	EVSSLEGsPPPcLGQEEAVcTK	3	S6(Phospho)	S(6): 42.6; T(8): 7.4; T(11): 42.6; T(13): 7.4; S(25): 0.0
Q15276	TPSPKEEDEPEsPPEKK	3	S4(Phospho); S7(Phospho)	S(4): 87.7; T(5): 12.3; S(7): 12.2; T(10): 87.1; S(11): 0.3; S(13): 0.3; S(16): 0.0
Q9H1E3	SDKsPDLAPTPAPQSTPR	3	S13(Phospho)	T(1): 0.0; S(3): 0.0; S(13): 100.0
Q9Y2B0	KGsITEYTAAEEK	1	S4(Phospho)	S(4): 92.7; S(5): 7.3
Q86WC4	NKQDDDLNcEPLsPHNITPEPVSK	1	S12(Phospho)	S(1): 0.0; S(2): 0.0; T(3): 0.0; S(4): 0.0; S(12): 100.0
Q9Y6D5	GEAAAERPGEAAVAsSPSK	2	S3(Phospho); S6(Phospho)	S(3): 100.0; S(6): 92.4; T(9): 7.6; S(12): 0.0
O60504	SHGKDEEcVLEAENKK	7	C2(Carbamidomethyl); S11(Phospho)	T(10): 0.5; S(11): 99.5
Q00613	STETSDFENIEsPLNER	1	S11(Phospho)	T(3): 0.0; T(5): 0.0; S(11): 47.4; T(15): 0.1; S(16): 47.4; T(17): 5.1
Q8N573	ADDFPVRDDPsDVtDEDEGPAEPPPPPK	2	S4(Phospho)	S(3): 0.4; S(4): 92.9; T(5): 6.2; S(6): 0.4; T(13): 0.0
Q96RT1	GDRSEDFGVNEDLADSDAR	1	C8(Carbamidomethyl); S17(Phospho)	T(1): 0.0; S(6): 0.0; S(9): 0.0; S(15): 96.5; S(17): 3.3; T(18): 0.1; S(22): 0.0
Q9Y520	RVsVcAETYNPDEEEEDTDPR	2	T10(Phospho); C18(Carbamidomethyl)	S(1): 0.0; T(10): 98.7; S(16): 1.2
Q9H4I2	DGGRsSPGGQDEGGFMAQGK	6	S7(Phospho)	S(7): 100.0; S(25): 0.0
Q7Z6Z7	sIQDLTVTGTEPGQVSSR	1	S6(Phospho)	S(2): 0.0; T(4): 0.0; S(6): 100.0
Q9UKJ3	sQEADVQDWEFR	1	S3(Phospho)	S(3): 87.6; S(5): 12.3; T(11): 0.0
P34932	DDDKNNDGYIDYAEFAK	3		
Q8WVT3	REDsPGPEVQPMDK	2	S22(Phospho); S24(Phospho); C31(Carbamidomethyl)	T(20): 82.9; S(22): 20.9; S(24): 96.2; S(39): 0.0; S(40): 0.0
Q5T1M5	KGAGDGsDEEVDGK	3	S2(Phospho); S4(Phospho)	S(1): 50.0; S(2): 50.0; S(4): 100.0
Q92614	IAAPELHKGDsDsEEDEPTK	1	S7(Phospho)	S(7): 100.0; S(11): 0.0
Q9H1C4	KPVKENQSEGKGSDsDSEGDNPEK	1	S7(Phospho); S10(Phospho)	Y(1): 5.7; S(7): 95.4; S(10): 98.9
Q15276	SsLsGDEEDELFK	1	S3(Phospho); S6(Phospho)	S(3): 90.4; T(4): 9.6; S(6): 98.7; T(9): 1.2; S(10): 0.0; S(12): 0.0; S(15): 0.0
Q99856	SHWDDSTsDSELEK	6	S11(Phospho); S15(Phospho)	S(11): 100.0; S(15): 100.0; S(22): 0.0
O15440	KNsAIPSELNEK	1	S12(Phospho); S16(Phospho)	T(3): 0.0; S(8): 0.2; S(9): 1.2; S(11): 49.3; S(12): 49.3; S(16): 100.0
P27540	HQEGEIFDTEKEKYEITEQR	7	S4(Phospho)	S(4): 100.0; S(9): 0.0; S(10): 0.0
O15021	RPNEDsDEDEEKGAVVPPVHDIYR	1	S3(Phospho)	S(3): 100.0; T(7): 0.0
Q13136	SYLEGSsDNQLKDSESTPVDDR	1	S3(Phospho); S6(Phospho)	S(2): 50.3; S(3): 50.3; S(6): 97.9; S(8): 1.5
O14974	ESESALMEDRDEsEVsDEGGSPISSEGQEPR	2	T3(Phospho)	S(2): 8.9; T(3): 90.2; T(7): 0.9; T(9): 0.0
Q14161	DDGVFVQEVTQNsPAAR	2	T4(Phospho)	S(3): 50.0; T(4): 50.0; S(7): 0.1; Y(9): 0.0; T(12): 0.0; S(23): 0.0; S(24): 0.0
Q96AT1	TITLEVEPSDTIENVK	1	S6(Phospho)	S(2): 0.0; S(3): 0.0; S(6): 100.0
Q13286	SHyADVDPENQNFLLESNLGK	2	S3(Phospho); S5(Phospho)	S(3): 100.0; S(5): 100.0; T(10): 0.0
Q13424	HGsGADSDYENTQSGDPLLGLEGKR	1	S12(Phospho); S16(Phospho)	S(2): 0.0; T(3): 0.0; T(6): 0.0; S(7): 0.0; S(12): 100.0; S(16): 100.0
Q6PKG0	ASRVPssDEEVVEEPQSR	2	S17(Phospho)	S(2): 0.0; S(17): 100.0
Q9UJD0	HKKEEEDEELDLNK	2	S5(Phospho); S6(Phospho); C15(Carbamidomethyl)	S(3): 99.0; S(5): 1.9; S(6): 98.9; S(9): 0.1; T(11): 0.0; S(12): 0.0; S(14): 0.0; S(16): 0.0
Q6VMQ6	sAGEEEDGPVLTDEQK	1	S3(Phospho)	S(1): 50.0; S(3): 50.0; S(11): 0.0
O43432	SRsDIDVNAAASAK	3	S3(Phospho)	S(3): 93.3; S(4): 6.7; S(12): 0.0
P51858	STVTGERQsGDGQESTEPVENK	1	S6(Phospho); S7(Phospho)	S(6): 100.0; S(7): 100.0
P16333	RSAsPHDVDLcLVSPcEFEHR	2	S4(Phospho)	S(4): 99.9; S(8): 0.1; S(10): 0.0; S(15): 0.0
Q5T8D3	TEPAAEAEAASGPSESPsPPAAEELPGSHAEPPVPAQGEAPGEQAR	7	S6(Phospho); S10(Phospho)	S(3): 1.0; S(4): 1.0; S(6): 98.1; S(10): 100.0; S(25): 0.0
Q9ULX3	IEDsEPHIPLIDDTDAEDDAPTKR	4	S4(Phospho)	S(4): 100.0; T(13): 0.0; S(15): 0.0
Q9BY44	KQSFDDNDsEELEDKDSK	3	S4(Phospho)	S(1): 0.9; S(4): 99.1; T(10): 0.0; S(15): 0.0; T(16): 0.0
Q96AT1	KQEtAAVcGETDEEAGEsGGEGIFR	1	S14(Phospho)	S(14): 100.0
Q7RTP6	NAPAAVDEGSIsPR	1	T3(Phospho)	T(3): 6.7; S(4): 0.5; S(6): 92.8
P35579	EEGSLsDTEADAVSGQLPDPTTNPSAGK	3		
P35658	EKGPTTGEGALDLSDVHsPPKsPEGK	5	S4(Phospho); S11(Phospho)	S(1): 8.5; S(4): 83.1; T(5): 8.5; T(7): 1.0; T(8): 9.1; T(10): 88.8; S(11): 1.0; S(12): 0.1
Q92538	NKPGPNIEsGNEDDDASFK	4	S12(Phospho)	S(10): 14.7; S(12): 85.3; S(16): 0.0; Y(17): 0.0; S(23): 0.0
Q09472	KHEAFESDLAAHQDR	5	S18(Phospho)	T(1): 0.0; S(11): 0.0; T(12): 0.0; S(13): 0.0; T(15): 1.5; S(17): 11.0; S(18): 87.3; S(24): 0.2
Q6QNY0	KAsPEPPDSAEGALK	2	T8(Phospho); S10(Phospho)	T(8): 100.0; S(10): 100.0; T(19): 0.0
P54105	DKYEPAAVSEQGDKK	3	S17(Phospho)	S(5): 0.0; S(17): 100.0; T(25): 0.0
Q96B36	ASLGSLEGEAEAEASsPK	7	S6(Phospho); S14(Phospho)	T(1): 0.0; S(5): 50.0; S(6): 50.0; S(14): 91.5; S(15): 8.5
P06493	VVsSTSEEEEAFTEK	4	T5(Phospho); Y6(Phospho)	T(5): 100.0; Y(6): 99.9; Y(10): 0.1
Q9H6Z4	REGPVGGEsDSEEMFEK	2	S7(Phospho)	S(7): 46.3; S(9): 46.3; S(11): 3.2; S(12): 3.2; S(13): 0.9; T(17): 0.0; S(22): 0.0; S(26): 0.0; Y(30): 0.0; T(31): 0.0
Q09666	SEDFGVNEDLADSDAR	1	S2(Phospho); S5(Phospho)	S(2): 100.0; S(5): 100.0; S(15): 0.0; S(16): 0.0
Q27J81	DEALEQNIQALEENHQTQEQESLVQEDK	1	S29(Phospho)	T(20): 0.0; S(24): 0.0; T(28): 18.1; S(29): 81.9
Q5ZPR3	YGPADVEDTTGSGATDSKDDDDIDLFGsDDEEESEEAKR	11	S4(Phospho)	S(2): 0.0; S(4): 100.0
O95671	KGAANAsGSSPDAPAK	5	S14(Phospho)	S(3): 2.4; T(9): 0.0; S(14): 97.6; S(21): 0.0; T(24): 0.0
P78559	NsPEDLGLSLTGDScK	2	S10(Phospho)	T(9): 5.8; S(10): 94.2
Q09666	NPYLLsEEEDDDVDGDVNVEK	1	S8(Phospho)	S(1): 48.9; S(2): 48.9; S(5): 1.1; S(8): 1.1; S(18): 0.0; S(19): 0.0
P0DJ93	YQDEVFGGFVTEPQEEsEEEVEEPEER	2	S16(Phospho); S18(Phospho)	T(6): 0.0; S(8): 0.0; S(16): 99.5; S(18): 97.4; T(20): 3.1; T(24): 0.0; S(25): 0.0; S(26): 0.0; S(27): 0.0
Q92614	ASLAsLDsNPSTNEK	1	S7(Phospho); S9(Phospho)	Y(1): 0.0; S(2): 0.0; S(4): 0.0; Y(5): 0.1; S(7): 99.8; S(9): 98.9; T(11): 1.1
Q6ZSR9	KPEsPYGNLcDAPDsPRPVK	2	Y2(Phospho)	Y(2): 99.2; S(3): 0.8; S(9): 0.1; Y(18): 0.0
Q70Z53	DMsPLSETEMALGK	5	S10(Phospho)	S(2): 11.9; S(5): 87.9; S(10): 0.3; S(15): 0.0; S(17): 0.0; S(19): 0.0
Q15811	KVDVDEYDENKFVDEEDGGDGQAGPDEGEVDScLR	1	S12(Phospho); S14(Phospho)	S(1): 0.0; T(4): 0.0; T(7): 0.0; T(9): 0.0; S(11): 10.2; S(12): 90.0; S(14): 99.8
Q14157	RLPQDHADScVVssDDEELSR	2	S8(Phospho)	S(1): 0.0; T(2): 0.0; S(3): 0.0; S(8): 83.8; S(11): 8.1; S(12): 8.1; S(16): 0.0; S(17): 0.0; S(18): 0.0
P46821	tASGSsVTsLDGTR	1	S12(Phospho)	S(11): 0.7; S(12): 98.6; T(14): 0.7; Y(17): 0.0
Q9UGV2	DEREDITHTYKYPEGSSEER	2	T3(Phospho); T6(Phospho)	S(1): 0.1; T(3): 0.4; S(5): 14.6; T(6): 77.6; S(7): 77.6; S(8): 21.2; S(9): 8.5; S(12): 0.0; S(15): 0.0; S(18): 0.0
P16591	QAQQERDELADEIANSSGK	1	Y15(Phospho)	Y(15): 100.0
Q9C0C2	KPATPAEDDEDDDIDLFGsDNEEEDKEAAQLR	10	C7(Carbamidomethyl); S10(Phospho)	T(1): 0.0; S(10): 100.0; S(18): 0.0; S(19): 0.0; S(20): 0.0
Q12983	LGADESEEEGRRGsLSNAGDPEIVK	4	S8(Phospho)	S(2): 0.0; T(4): 0.0; T(6): 7.9; S(8): 92.1

P35579	RIQPQPPDEDGDHsDKEDEQPQVVVLK	1	S7(Phospho)	S(7): 100.0
P02765	KYTLENKEEGSLsDTEADAVSGQLPDPTTNPSAGK	2	C1(Carbamidomethyl); S7(Phospho)	S(3): 0.0; S(4): 0.0; S(7): 100.0
Q9UEW8	DHSsQSEEEVVEGEKEVEALKK	5	S9(Phospho)	T(1): 0.0; S(9): 100.0
P48634	GSTIETEQKEDKGEDSEPVTSK	1	S4(Phospho); S12(Phospho)	S(4): 100.0; S(12): 100.0
Q9Y4E1	RSsQPsPTAVPASDSPPTKQEVK	4	S4(Phospho)	T(2): 7.2; S(4): 92.8
P27708	NKLEGDsDVDSELEDRVDGVK	2	S5(Phospho)	S(5): 100.0
Q92597	AsSVTTFTGEPNTcPR	2	S1(Phospho); S3(Phospho)	S(1): 93.7; S(3): 52.7; T(5): 52.6; S(6): 1.0; T(14): 0.0; S(17): 0.0; S(23): 0.0
Q8TBB5	GTLDEEDEEADsDTDDIDHR	2	S14(Phospho); S19(Phospho); C31(Carbamidomethyl)	T(4): 0.0; S(8): 0.1; S(14): 99.9; S(19): 99.0; S(25): 1.0
Q9NY27	LTQTSsTEQLNVLETETEVLNK	8		
Q96C19	HSsETFSSTPSATR	1		
Q92466	GHYEVtGSDDETGKLQGSGVSLASK	5	S3(Phospho)	S(1): 4.4; S(3): 95.6
Q8TC07	RKPsVPDSASPADDSFVDPGER	1	S3(Phospho); C14(Carbamidomethyl)	S(3): 100.0; T(5): 0.0; S(10): 0.0; S(11): 0.0
Q9UDY2	GKGGVtGSPEASISGsKGDLK	2	S5(Phospho)	S(2): 0.0; Y(3): 0.1; S(5): 99.9; Y(12): 0.0
Q96PD2	KTEAPAAPAAQETKsDGAPASDSKPGSSEAAPSSK	1	T1(Phospho); C4(Carbamidomethyl); S6(Phospho)	T(1): 50.0; S(3): 50.0; S(5): 8.5; S(6): 91.5; Y(11): 0.0; T(13): 0.0
Q14247	VVDYSQFQEsDDADEDYGR	4	T3(Phospho); T13(Phospho)	T(1): 19.1; T(3): 81.8; S(7): 97.2; T(13): 1.5; S(19): 0.1; S(20): 0.1; Y(23): 0.1; S(28): 0.0
O00499	GTGQsDDSDIWDDTALIK	3	S6(Phospho); S11(Phospho)	S(4): 50.0; S(6): 50.0; S(11): 99.9; T(15): 0.1
Q9UHX1	NSPVtKtPPRDLPTIPGVTSPSSDEPPMEASQSHLR	2	C14(Carbamidomethyl)	
O60711	NAsAsFQELEDK	1	S6(Phospho)	S(1): 0.0; T(2): 0.0; S(6): 100.0; Y(9): 0.0; S(10): 0.0; S(21): 0.0
Q86VR2	DNLTLWTSDQQDEEAGEGN	1	S6(Phospho); S8(Phospho); C16(Carbamidomethyl)	S(6): 100.0; S(8): 100.0; S(22): 0.0; T(23): 0.0
Q5THJ4	SQsMDIDGVScEK	1	T10(Phospho); S14(Phospho)	Y(7): 0.0; T(10): 100.0; S(14): 99.1; T(16): 0.9
O43493	ERsESLDPDSSMDTTLILK	3	T1(Phospho)	T(1): 98.5; S(3): 1.4; T(7): 0.2; S(11): 0.0
Q13017	KEESEEsDDDMGFGLFD	5	S3(Phospho); S6(Phospho)	T(1): 11.2; S(3): 89.0; S(6): 99.8; T(12): 0.0; T(13): 0.0; S(14): 0.0
Q9BTU6	SGsESEEAELSLSLAR	2	S3(Phospho)	S(1): 5.6; S(2): 5.6; S(3): 83.3; S(5): 5.6; Y(6): 0.0; T(7): 0.0; S(9): 0.0; S(12): 0.0
Q9BRS8	TTRtPEEGGYSYDISEK	1	S8(Phospho); S10(Phospho)	Y(1): 1.7; S(3): 1.7; S(8): 98.3; S(10): 98.3; S(15): 0.0
Q09666	GLSDHVsLDGQELGTR	3	S6(Phospho)	T(4): 0.7; S(6): 99.3; S(10): 0.0; S(12): 0.0; S(14): 0.0
Q3KQU3	DFAARSPsAsITDEDSNV	2	S3(Phospho); S6(Phospho)	S(2): 1.3; S(3): 98.6; S(6): 90.1; T(8): 10.0; S(13): 0.0; S(15): 0.0; T(18): 0.0
Q9P1Y6	QKQEEESLGQVTDQVEVNAQNSVPDEEAK	1	T11(Phospho)	S(9): 0.1; T(11): 99.9; T(17): 0.0; S(19): 0.0
Q92805	RStQGVTLTDLQEAEK	2	S9(Phospho); S14(Phospho)	S(1): 50.0; S(3): 50.0; S(6): 0.0; S(9): 0.1; S(14): 99.9; S(20): 0.0; S(23): 0.0; S(24): 0.0; S(25): 0.0
P78559	TETQEKNPLPSKETIEQEK	1	S6(Phospho)	S(6): 99.9; T(10): 0.1; T(16): 0.0
Q86W92	LcDDGPQLPTsPR	2	S8(Phospho); S10(Phospho)	S(6): 99.9; S(8): 0.1; S(10): 91.7; T(12): 8.3; S(16): 0.0
Q15637	GSIsEDELITAIK	2	S14(Phospho); S16(Phospho)	T(1): 0.0; S(14): 100.0; S(16): 99.6; Y(21): 0.3; S(23): 0.1
Q9UEY8	NTVDLVTTcHIHSsSDDEIDFK	2	S12(Phospho); S14(Phospho)	S(6): 0.0; S(10): 99.0; S(12): 1.9; S(14): 90.3; S(16): 8.8
P02765	KAVAEEDNGSIGEETDSsPGRK	2	C1(Carbamidomethyl); S7(Phospho)	S(3): 0.0; S(4): 0.0; S(7): 100.0
P31749	GLAAEDsGGDSKDLSEVSETTESTDVK	3	S5(Phospho)	S(1): 6.0; S(3): 46.6; S(5): 46.6; S(8): 0.8; S(16): 0.0
Q14697	TLEVVsPSQSVTGSAGHTPYYQSPTDEK	3		
Q14008	KFQEQEcPPsPEPTRK	2	S2(Phospho); S4(Phospho)	S(2): 82.7; T(3): 17.3; S(4): 17.3; T(6): 82.7; S(19): 0.0
Q14247	EENPEsDGEPVVEDGTSVK	1	T1(Phospho); S7(Phospho)	T(1): 50.0; T(3): 50.0; S(7): 100.0; T(13): 0.0
Q5VSL9	RSsLPLDHGSPAQENPESEK	1	S3(Phospho)	S(3): 100.0; S(7): 0.0; S(9): 0.0
Q9C0C2	GLMAGGRPEGQYsEDEDTDTDEYKEAK	2	S2(Phospho)	S(2): 100.0; Y(6): 0.0; S(8): 0.0
Q92597	SSsESYTQSFQSR	3	T3(Phospho); T12(Phospho)	S(1): 12.3; T(3): 75.4; S(4): 12.4; T(12): 99.4; S(15): 0.5; S(21): 0.0
Q09666	DKNHDEEPTASIHLNK	1	C6(Carbamidomethyl); S13(Phospho)	S(4): 0.0; S(5): 0.0; S(7): 0.0; S(8): 0.0; S(13): 100.0; Y(19): 0.0
Q92974	EGTGQQERESPRPLQLPGAEGPAIsDGEEGGGEPGAGGGAAGAAGAGR	1	S20(Phospho); S24(Phospho)	S(4): 0.0; S(5): 0.0; T(9): 0.0; S(11): 0.0; S(16): 0.3; S(17): 12.7; S(20): 88.6; S(24): 98.5
Q5JRA6	DRDLEQTDKAPEQK	2	S9(Phospho); S10(Phospho)	T(2): 0.5; S(7): 13.7; S(8): 61.9; S(9): 61.9; S(10): 61.9; S(25): 0.0
Q9NZT2	DHFGLEGDEESTMLEDSVsPKK	2	S7(Phospho)	S(7): 100.0; S(16): 0.0; T(17): 0.0
Q99733	DSQDASAEQsDHDDEVASLASASGGFGTK	5	S18(Phospho)	T(4): 0.0; T(10): 0.0; S(14): 0.0; S(18): 100.0
Q13439	sDSASSEPVGIYQGFEK	6	S3(Phospho)	T(1): 6.5; S(2): 6.5; S(3): 80.4; T(5): 6.5; T(12): 0.0
O14936	KWDGsEEDEDNSK	1	S5(Phospho); C6(Carbamidomethyl)	T(1): 0.0; S(3): 0.0; S(4): 0.1; S(5): 99.9; S(11): 0.0; T(12): 0.0; T(13): 0.0
O15127	DVYKEHFQDDVFNEK	2	S3(Phospho)	S(3): 99.5; S(4): 0.5
O95365	KSLDsDEsEDEEDDYQQK	1	S8(Phospho)	S(8): 99.8; S(12): 0.2; Y(22): 0.0; Y(23): 0.0
P46821	SPsDSGYSYETIGK	3	S1(Phospho)	S(1): 98.9; S(3): 1.1; S(5): 0.0; Y(7): 0.0; S(8): 0.0; Y(9): 0.0; T(11): 0.0
Q9H2P0	DLQEQDADAGsER	1	S6(Phospho); S8(Phospho)	S(6): 100.0; S(8): 100.0
Q14137	VAENVADKNEEPSsNHIPHGK	2	S9(Phospho); S10(Phospho)	Y(5): 0.0; S(9): 100.0; S(10): 100.0
P51858	HySPEDEPSPEAQPIAAYK	3	S7(Phospho)	S(6): 10.9; S(7): 89.1
O60271	GVAGPGPIGREPDPDsDWEPEER	3	S13(Phospho)	T(2): 0.0; Y(4): 0.0; S(11): 89.0; S(13): 11.0
Q92785	EGTGQQERESPRPLQLPGAEGPAIsDGEEGGGEPGAGGGAAGAAGAGR	1	S5(Phospho)	S(5): 100.0; T(12): 0.0; S(14): 0.0
P18858	IHGVNSGSSEGAQPNTENGVPEITDAATDQGPAEsPPTsPSSASR	2	S4(Phospho); S14(Phospho)	S(4): 100.0; S(14): 100.0
O60271	RGSDAsDFDLLETQsAcSDTSESSAAGGQGNSR	3	S3(Phospho); S5(Phospho)	S(1): 8.4; S(3): 90.8; S(5): 1.8; S(6): 99.1
P35579	DGGRSsPGGQDEGGFMAQGk	2		
Q13017	EGALSRVsDEsLSKVQEAESPVFK	1	S10(Phospho)	T(9): 7.9; S(10): 92.1
P07437	VESDLLDQEEMVDKPDIGsYER	2		
Q9BS91	KAEGEPQEEsPLKSK	1	S6(Phospho); S9(Phospho)	T(2): 0.0; S(6): 100.0; S(9): 99.8; T(13): 0.2
Q16512	NTFTAWsDEESDYEIDDRDVNK	3	S10(Phospho)	T(5): 0.0; S(7): 0.0; S(9): 7.1; S(10): 92.9
Q6P158	SGsAAQAEGLcK	2	S11(Phospho)	S(11): 100.0
P46940	RGTGQsDDSDIWDDTALIK	2		
O43237	GNAEGssDEEGKLVIDEPAKEK	2	C12(Carbamidomethyl); S15(Phospho)	Y(5): 0.0; S(15): 100.0
Q96RU3	KKDAsDDLDDLNFFNQK	1	S3(Phospho)	T(1): 0.0; S(3): 100.0; S(6): 0.0; S(8): 0.0; S(10): 0.0
O95674	DSENLAsPSEYPENGER	1	S26(Phospho)	S(14): 0.0; S(16): 0.0; T(24): 13.4; S(26): 85.8; S(28): 0.4; S(30): 0.4
P08240	AGNsDsEEDDANGRVELILEPK	2	C13(Carbamidomethyl); S15(Phospho); S16(Phospho)	T(2): 0.4; S(4): 0.4; S(14): 25.0; S(15): 87.0; S(16): 87.0; S(25): 0.0; T(26): 0.0; S(29): 0.0; T(31): 0.0
Q5JSZ5	sRsHTSEGAHLDITPNSGAAGNSAGPK	5	S4(Phospho)	S(4): 100.0
C9JLW8	AtTPPNQGRPDsPVYANLQELK	2	S3(Phospho); S6(Phospho)	T(1): 36.1; S(2): 36.1; S(3): 36.1; S(6): 64.4; S(9): 9.1; S(10): 9.1; S(11): 9.1; T(15): 0.0
Q9Y4E1	RSsDGsLSHEEDLAK	1	S3(Phospho)	T(1): 0.5; S(3): 99.5
Q8WVC0	GHYEVTGsDDETGK	2	S4(Phospho)	T(3): 9.4; S(4): 90.6; S(11): 0.0
P20020	AKTQtPPVSPAPQPtEERLPSSPVyEDAASFK	1	S4(Phospho)	S(4): 100.0; T(14): 0.0; T(22): 0.0
Q8WWI1	EPALPLEPDSGGNTsPGVTANGEAR	1	T5(Phospho)	S(1): 0.0; S(3): 0.0; T(4): 3.4; T(5): 96.6; Y(10): 0.0; S(11): 0.0; T(12): 0.0
P46821	IEEVLSPEGSPsKsPSK	1	S10(Phospho); S13(Phospho); S16(Phospho)	S(3): 0.0; S(10): 89.5; S(13): 89.5; S(16): 20.9; T(19): 100.0
Q8N3X1	YLEEDNsDEsDAEGEHGDGAEEEAPPAGPRPGPEPAGLGR	1	C6(Carbamidomethyl); S14(Phospho)	T(2): 0.0; Y(11): 0.0; S(14): 99.9; S(22): 0.1
Q5H9R7	RRPsDENTIAPSEVQK	1	S9(Phospho)	S(9): 100.0
Q9UHI6	ELAPAWEDTsPEQDNR	7	S6(Phospho); S7(Phospho)	S(1): 0.0; Y(2): 0.0; S(6): 99.1; S(7): 99.9; S(14): 1.0; S(16): 0.0; T(17): 0.0
Q99543	GNKsPSPPDGSPAATPEIR	4	S3(Phospho)	S(3): 99.8; S(5): 0.2
Q9BVS4	RFssGGEEDDFDR	1	S3(Phospho); S6(Phospho)	S(3): 2.4; S(6): 98.8; S(8): 98.8
Q9H788	SHsPSSPDPDTPSPVGDSR	2	S3(Phospho)	T(1): 0.8; S(3): 98.3; S(4): 0.8; S(5): 0.1
P54727	tSDSDQQAYLVQR	2	T20(Phospho)	T(11): 0.0; T(15): 0.0; S(16): 0.1; T(18): 0.3; T(20): 99.0; S(22): 0.3; T(23): 0.3; S(24): 0.1; S(27): 0.0; S(28): 0.0
Q9H2G2	TNSsDsERSPDLGHSTQIPR	1	S11(Phospho)	S(5): 0.0; T(9): 0.0; S(11): 100.0
Q4L180	KGGSYSQAASSDsAQGsDVsLTAcKV	1	S3(Phospho)	S(1): 6.7; S(3): 92.7; S(5): 0.5; S(6): 0.0; S(7): 0.0; T(10): 0.0; T(11): 0.0
Q9C0D5	IAELEEEQSQGSTTNsDWMK	1	C5(Carbamidomethyl); S6(Phospho)	S(6): 100.0; T(13): 0.0; T(14): 0.0; S(16): 0.0; S(19): 0.0; T(20): 0.0
Q16637	DMEsPTKLDVTLAK	4	S5(Phospho); S8(Phospho)	T(2): 50.0; S(5): 50.0; S(8): 99.8; T(14): 0.2
P13861	KDsIPQVLLPEEEK	2	S3(Phospho); S5(Phospho)	S(3): 100.0; S(5): 100.0; S(17): 0.0
P48307	KDDEENYLDLFSHK	1	C5(Carbamidomethyl); S6(Phospho)	S(6): 100.0; T(10): 0.0

O60841	KDEGEGAAGAGDHKDPSLGAGEAASK	4	S5(Phospho)	S(5): 100.0; S(12): 0.0
Q96B36	DAcRDHMEGYHYR	1	S2(Phospho); S10(Phospho)	S(1): 50.0; S(2): 50.0; S(10): 9.4; S(11): 90.6
P27816	YFQISQDEDEsESED	4	S4(Phospho)	S(4): 99.5; T(6): 0.5; T(11): 0.0
O75592	KGAGDGsDEEVDGKADGAEAKPAE	9	S3(Phospho)	S(1): 50.0; S(3): 50.0; S(5): 0.0; Y(6): 0.0; T(7): 0.0; T(12): 0.0
Q9H1E3	HRGsEEDPLLSPVETWK	1	S5(Phospho); S9(Phospho); S12(Phospho)	S(1): 0.1; S(5): 99.9; S(9): 100.0; S(12): 100.0
O00418	DNLTLWTSDMQGDGEEQNKEALQDVEDENQ	2	S4(Phospho); S8(Phospho)	Y(2): 0.0; S(4): 100.0; S(8): 100.0; S(11): 0.0; S(12): 0.0
Q9P0P8	NKsESQcDEDGMTSSLSESLK	1	S10(Phospho); S16(Phospho)	S(6): 98.2; S(10): 79.3; S(16): 22.5; T(27): 0.0
P84157	LSEIDVSSEGVK	1	S31(Phospho)	S(14): 0.2; S(15): 0.4; S(27): 7.1; Y(30): 21.7; S(31): 70.6
Q8NDI1	RKPEDVLDDDDAGsAPLK	2	S5(Phospho); S9(Phospho); S13(Phospho)	S(3): 80.0; T(4): 10.0; S(5): 10.0; S(9): 99.9; S(13): 100.0
Q5JTV8	EAEHLSSPWGEsSPEELR	2	S6(Phospho); S8(Phospho); S9(Phospho)	S(6): 100.0; S(8): 100.0; S(9): 100.0; S(15): 0.0; S(16): 0.0; T(18): 0.0; S(21): 0.0; T(23): 0.0; S(25): 0.0
Q969E4	DRWPEVsPEDTQSLSLSEESPSK	3	T2(Phospho); S9(Phospho)	T(2): 50.0; S(5): 50.0; S(9): 100.0
Q96K76	RWsAEVTSSTYSDEDRPPK	1	S12(Phospho)	S(1): 0.0; T(2): 0.0; T(4): 0.0; S(5): 0.0; S(12): 100.0
O60238	TLSNAEDYLDDEDsD	4		
O43847	HVsFQDEDEIVR	1	S3(Phospho)	S(3): 50.0; S(5): 50.0
Q14247	RDHALLEEQSK	1	T3(Phospho); S9(Phospho)	T(3): 0.5; T(5): 99.5; S(9): 100.0; T(15): 0.0
Q09666	NLSLTDFVGDESPsPER	4	T6(Phospho); S16(Phospho)	T(6): 50.0; S(8): 50.0; S(12): 0.0; S(14): 12.9; S(16): 87.1
Q99543	ASLGSLEGEAEAEASsPKGK	4	S3(Phospho); S5(Phospho)	S(3): 100.0; S(5): 100.0
Q8NFA0	ASsESQIFELNK	2	S4(Phospho)	S(2): 0.4; S(3): 49.8; S(4): 49.8; S(11): 0.0
Q5JRA6	EDSRGsLIPEGATGFPDQGNTGENTR	3	S10(Phospho)	T(2): 4.5; S(7): 4.5; S(8): 30.3; S(9): 30.3; S(10): 30.3; S(25): 0.0
P35613	DHFGLEGDEESTMLEDSVsPK	4	S14(Phospho)	S(14): 100.0
Q92974	DQPDGSSLsPAQsPSQSQPPAASSLR	2	S20(Phospho); S24(Phospho)	S(4): 0.0; S(5): 0.0; T(9): 0.0; S(11): 0.0; S(16): 0.7; S(17): 0.1; S(20): 99.1; S(24): 50.0; T(29): 50.0
Q6UN15	KGESQTDIEITREEDFTR	1	T5(Phospho)	S(3): 49.9; T(5): 49.9; S(7): 0.3; S(11): 0.0
P14618	DMsPLsETEMALGK	2	C6(Carbamidomethyl)	
Q9UFC0	VHHEPQLSDKVHNDAQSFDYDHDAFLGAEEAK	1	S3(Phospho)	S(3): 100.0
Q6GQQ9	VDsTTcLFPVEEK	3	S8(Phospho)	S(1): 0.0; T(2): 0.0; S(5): 0.2; S(8): 99.5; S(12): 0.2; S(15): 0.0; S(16): 0.0; S(17): 0.0; T(18): 0.0; S(19): 0.0
P27824	SGSsSPDSEITELKFPSINHD	21		
O15027	GNKsPsPPDGSPAATPEIR	3	S5(Phospho)	S(4): 50.0; S(5): 50.0; T(11): 0.0; T(14): 0.0; T(15): 0.0; S(17): 0.0; Y(20): 0.0
P35611	DFQEYVEPGEDFPAsPQR	2	S5(Phospho)	S(5): 100.0
A7KAX9	NLLLSTsEEQIEK	1	S4(Phospho)	S(1): 1.7; S(4): 96.3; S(7): 1.7; T(9): 0.3; S(10): 0.0; S(18): 0.0
Q8TB72	AVPIAVADEGEsEsEDDDLKPR	1	S4(Phospho)	S(4): 100.0
P17096	SHsANDSEEFFREDDGGADLHNATNLR	1	S11(Phospho); S14(Phospho); S15(Phospho)	S(11): 100.0; S(14): 100.0; S(15): 100.0
P82094	SEGEGKPQGEGKPASQAKPESQPR	2	S3(Phospho); S7(Phospho)	S(1): 100.0; S(3): 0.0; S(7): 100.0; S(12): 0.0
Q92597	FLESAAADFsDEDEDDDVDGR	1	T3(Phospho)	S(1): 8.0; T(3): 91.3; S(4): 0.7; T(12): 0.0; S(15): 0.0; S(21): 0.0
Q9BTU6	GGVTGsPEASISGSK	1	S10(Phospho); S14(Phospho)	S(7): 0.0; S(10): 100.0; S(14): 100.0
Q9H6H4	DKPEEQWWNAEDSEGKR	2	S7(Phospho); C13(Carbamidomethyl); S15(Phospho)	S(7): 97.9; T(9): 2.1; S(15): 99.7; T(17): 0.3
Q6PKG0	RNSLtGEEGQLAR	3	S3(Phospho)	S(3): 100.0
O43765	STsFRQGPEESGLGDGTGPK	3	S5(Phospho)	S(1): 0.0; T(3): 0.0; S(5): 99.6; S(7): 0.4
P78559	DAYRPTTDADKIEDEVTR	2	S12(Phospho)	S(12): 99.9; T(20): 0.0; Y(26): 0.0
P11142	ELSSEPQtPPAQK	1		
O75376	SHtSEGAHLDITPNSGAAGNSAGPK	12	S5(Phospho); C13(Carbamidomethyl)	S(5): 90.1; S(6): 9.8; S(9): 0.0; S(20): 0.0; S(22): 0.0
Q6P6C2	NLEELEEKSTtPPPAEPVSLPQEPPKPR	3	S2(Phospho); C9(Carbamidomethyl)	S(2): 93.0; Y(3): 7.0; S(5): 0.0; S(6): 0.0; S(10): 0.0; S(15): 0.0
P27816	DAQHYGGWEHR	2	S13(Phospho)	T(4): 0.0; S(7): 0.0; T(12): 0.4; S(13): 99.6
P41236	DGTAPPPQSPGSPGTGQDEEWsDEESPR	1	S6(Phospho); S7(Phospho)	S(6): 99.4; S(7): 100.0; S(12): 0.6; S(15): 0.0
Q6VY07	sAsPHDVDLcLVSPcEFEHR	2	S4(Phospho); S6(Phospho)	T(1): 49.8; S(3): 49.8; S(4): 29.0; S(6): 70.4; S(9): 1.0; S(15): 0.0; T(16): 0.0
Q5T5U3	KPKEDEVEASEK	2	S3(Phospho); C10(Carbamidomethyl)	S(3): 46.0; S(6): 46.0; S(9): 7.8; T(11): 0.3; S(14): 0.0; T(16): 0.0; S(17): 0.0; S(18): 0.0; S(21): 0.0
Q08AD1	AIEPQKEEADENYNsVNTR	2	S5(Phospho)	S(4): 7.7; S(5): 92.3; T(10): 0.0; T(12): 0.0
P67809	DAHDVSPTSTDTEAQLTVER	1		
Q92597	SPsFASEWDEIEK	1	S3(Phospho)	T(1): 0.0; S(3): 99.9; S(5): 0.0; S(6): 0.0; T(8): 0.0; S(9): 0.0; T(13): 0.0
Q96N67	IcTEQSNsPPPIR	2	S3(Phospho); S5(Phospho)	S(1): 12.2; S(3): 87.8; S(5): 87.8; S(7): 12.2; S(12): 0.0; T(14): 0.0; T(16): 0.0; S(17): 0.0
Q7Z417	KLEKEEEEGIsQEssEEEQ	2	S7(Phospho); S9(Phospho)	S(7): 99.8; S(9): 98.6; S(11): 1.5; Y(13): 0.0; T(14): 0.0; T(15): 0.0
Q09666	TEAQDLcRAsPEPPGPESSSR	1	S16(Phospho)	S(2): 0.0; S(5): 0.0; S(15): 1.1; S(16): 98.9
Q5VTR2	SAAsREDLVGPEVGASPQSGR	3	S13(Phospho)	S(11): 0.4; S(13): 99.6
Q86VR2	DIKPQLELIEDEEKLK	1	S9(Phospho); S16(Phospho)	T(3): 0.0; T(6): 0.0; S(9): 100.0; S(16): 100.0; S(21): 0.0
P18669	STGVSFWTQDSDENEQEQQsDTEEGSNKK	4	S4(Phospho)	S(4): 100.0
Q14247	WLAEsPVGLPPEEEDKLTR	4	T3(Phospho)	T(1): 0.9; T(3): 99.1; S(7): 0.0; T(13): 0.0
Q96G46	SHSSPsLNPDTsPITAK	1	C4(Carbamidomethyl); S16(Phospho); S20(Phospho)	T(15): 0.2; S(16): 10.8; T(17): 89.0; S(20): 89.2; S(21): 10.8
Q04917	sRTAsGSSVTSLDGTR	4		
Q16623	DAYKPITDADKIEDEVTR	2	S5(Phospho)	T(1): 0.1; S(5): 99.9; T(14): 0.0
Q8WVC0	GLYDGPVcEVSVtPK	1	S5(Phospho)	Y(2): 0.0; S(5): 100.0
P13798	AGEPNsPDAEEANsPDVTAGcDPAGVHPPR	4	S7(Phospho)	S(5): 0.0; S(7): 100.0
Q5T1M5	DYNASASTIsPPSSMEEDKFSR	7	S4(Phospho)	S(1): 0.1; S(2): 0.1; S(4): 99.8
O43719	sASPDDDLGSSNWEAADLGNEERK	2	S7(Phospho)	S(7): 100.0; S(18): 0.0; S(19): 0.0
Q5JTV8	TRNsGIWESPELDR	1	S2(Phospho); S4(Phospho)	S(2): 98.9; S(4): 90.2; S(5): 11.0; S(11): 0.0; S(12): 0.0; T(14): 0.0; S(17): 0.0; T(19): 0.0; S(21): 0.0
Q9BPX3	TSSVSNPQDSVGsPcSR	2	C4(Carbamidomethyl); S11(Phospho)	T(1): 0.0; T(7): 9.6; S(11): 90.4; S(17): 0.0; T(23): 0.0; T(25): 0.0
O94885	EESREPAPAsPAPAGVEIR	1	S3(Phospho)	T(2): 49.9; S(3): 49.9; Y(10): 0.1; S(13): 0.0; T(16): 0.0; S(18): 0.0
P80723	IHRAsDPGLPAEEPK	1		
Q66K14	DIEREDIEFIcK	2	T9(Phospho); S31(Phospho)	S(3): 99.8; S(8): 0.1; T(9): 0.1; S(11): 0.0; S(12): 0.0; S(19): 0.0; S(24): 0.1; S(27): 99.7; S(30): 0.1; S(31): 0.1
Q9NPE2	DSLLQDGEFSMDLR	1	S16(Phospho)	S(16): 100.0
Q13586	KEsKEETPEVTK	2	S3(Phospho)	S(1): 8.6; S(3): 91.3; S(5): 0.1; S(6): 0.0; T(11): 0.0; S(13): 0.0; S(18): 0.0
P78559	DLAEDLYDGQVLQK	1	S8(Phospho)	S(1): 0.0; S(6): 0.0; T(7): 5.0; S(8): 94.7; S(10): 0.3
Q9Y2X7	RGSGDTsISIDTEASIR	9	S3(Phospho); S7(Phospho)	S(3): 89.9; S(7): 10.1; Y(9): 17.7; T(12): 82.1; S(14): 0.2
O60271	SGSLDDsFSDFQELPASSK	5	S6(Phospho)	S(1): 0.6; S(3): 0.6; S(5): 6.9; S(6): 92.0
Q8IYB7	NEKGEVEQEKLDK	2	S17(Phospho)	T(4): 0.0; S(17): 100.0; S(24): 0.0; S(25): 0.0; T(26): 0.0; S(27): 0.0
Q13459	AVQQHQHGYDsDEEVDSELGTWEHQLR	1	S7(Phospho)	S(7): 100.0; S(11): 0.0; T(12): 0.0
O43493	MDKEETKDWILPSDYDHAEAEAR	4	S11(Phospho)	S(2): 0.0; S(10): 49.6; S(11): 49.6; T(14): 0.9
P17181	GKLsAEENPDDSEVPSSSGINSTK	1	S7(Phospho)	S(5): 0.0; T(6): 0.5; S(7): 99.5
Q96N67	AEDGSVIDYELIDQDAR	2	S1(Phospho)	S(1): 87.2; S(3): 12.7; S(5): 0.1; S(7): 0.1; S(12): 0.0; T(14): 0.0; T(16): 0.0; S(17): 0.0
Q9BW71	LDNTPAsPPRsPAEPNDIPIAK	3	S3(Phospho); S6(Phospho)	S(3): 100.0; S(6): 100.0
Q8IYB3	SVsPTFLNPSDENLK	4	S14(Phospho)	S(14): 100.0
Q15052	GLLYDsDEEDEERPAR	4	S3(Phospho)	S(3): 100.0
Q9UN37	QScLRPFTEDDAADPNDsDIDPESR	2	S15(Phospho)	S(8): 0.0; S(13): 0.2; S(15): 99.8; S(17): 0.0
P23588	VQSLEGEKLsPKsDIsPLTPR	2	C13(Carbamidomethyl); S15(Phospho)	S(1): 0.0; T(6): 0.2; S(15): 33.3; T(17): 33.3; S(18): 33.3
Q8IVF2	RNscNVGGGGGGFK	2	S3(Phospho)	S(1): 0.3; S(3): 99.7; S(4): 0.0; S(5): 0.0; Y(8): 0.0
Q92597	KPLSLAGDEETEcQSsPK	5	T1(Phospho); S6(Phospho); S9(Phospho)	T(1): 8.8; S(3): 91.1; S(5): 1.1; S(6): 98.9; T(8): 8.9; S(9): 91.2; T(13): 0.0
P27824	EEDEPEERsGDETPGSEVPGDK	1	S3(Phospho); S13(Phospho)	S(3): 100.0; T(11): 0.0; S(13): 100.0
Q6PKG0	TGSTsSKEDDYESDAATIVQK	5	S7(Phospho)	T(2): 0.0; T(4): 0.0; S(7): 79.5; S(11): 10.3; Y(13): 10.3
Q1KMD3	ALDVSAsDDEIAR	1	S10(Phospho)	S(10): 100.0; T(14): 0.0; S(17): 0.0
P07355	NTGIIcTIGPASR	1		

P29692	TASESISNLsEAGsIK	1	S19(Phospho)	T(4): 0.0; S(19): 100.0
Q92597	NsSEASSGDFLDLK	1	S6(Phospho); S9(Phospho)	T(1): 0.0; S(3): 0.0; S(5): 1.3; S(6): 88.6; T(8): 11.4; S(9): 98.7; T(13): 0.0
Q9BPX3	DKLESEMEDAYHEHQANLLR	3	C1(Carbamidomethyl); S8(Phospho); S10(Phospho); C-Term(Oxidation)	T(3): 0.1; S(8): 100.0; S(10): 99.9; S(19): 0.0
P78559	ADKDYHFKVDNDENEHQLSLR	2	T20(Phospho)	S(12): 50.0; T(20): 50.0
Q8N5D0	SQDATFSPGsEQAEKsPGPIVSR	1	S3(Phospho)	S(3): 100.0; S(5): 0.0
Q9H6Z4	TSsFTEQLDEGTPNR	3	S5(Phospho); S6(Phospho); S13(Phospho); C21(Carbamidomethyl)	S(1): 15.8; S(5): 86.5; S(6): 97.7; S(13): 100.0; Y(20): 0.0
Q13895	SREsLNVDVVK	2	S6(Phospho)	S(6): 100.0; T(15): 0.0
Q86W92	EFITGDVEPTDAESEWHsENEEEEK	1	S3(Phospho)	S(1): 0.7; S(3): 98.6; T(4): 0.7; T(5): 0.0; S(12): 0.0
Q7Z3C6	EVFSScSSEVVLsGDDEEYQR	3	S10(Phospho)	S(10): 100.0
Q9Y485	HGEsAWNLENR	1	S3(Phospho)	S(3): 100.0; S(15): 0.0
O14639	YFDsGDYNMAK	1	T2(Phospho)	S(1): 33.3; T(2): 33.3; S(3): 33.3; S(6): 0.0; S(9): 0.0; Y(12): 0.0; S(13): 0.0
P13807	VSPSKSPSLsPsPPsPLEK	10	S3(Phospho); S7(Phospho)	S(2): 8.3; S(3): 91.7; S(7): 100.0
P17987	RNsSEASSGDFLDLK	5		
P27797	SSLsGDEEDELFK	2		
Q9H1E3	LQDSSDPDTGSEEEGSSRLsPPHsPR	6	S11(Phospho)	Y(5): 0.0; S(6): 0.0; S(11): 100.0; Y(18): 0.0; S(22): 0.0; T(26): 0.0
Q05209	VDHGAEIITQsPGR	1	S21(Phospho)	T(6): 0.2; T(7): 0.2; S(9): 0.5; S(18): 5.9; S(21): 93.3
Q13501	ETSFGsSENITMTSLSK	2	S1(Phospho); S7(Phospho)	S(1): 0.1; T(4): 99.9; S(7): 100.0; S(10): 0.0; S(11): 0.0; S(12): 0.0; T(13): 0.0
P23588	TEIKEEEDQPSTSATQsSPAPGQSK	2	S5(Phospho); S7(Phospho)	S(1): 1.0; T(3): 9.1; S(5): 89.9; S(7): 1.1; S(8): 97.8; T(10): 1.1; T(12): 0.0; S(13): 0.0; T(14): 0.0; T(15): 0.0; S(16): 0.0; S(17): 0.0
Q9UGV2	DHANEELDELKR	1	S3(Phospho); S5(Phospho); S6(Phospho)	T(1): 7.5; S(3): 91.8; T(4): 0.7; S(5): 7.0; S(6): 86.0; S(7): 7.1; S(10): 99.9; S(13): 0.0; S(16): 0.0
Q15648	KEsIIKSEGELLER	1	S12(Phospho)	S(2): 0.0; S(3): 0.0; S(5): 0.0; S(11): 90.0; S(12): 9.9; T(15): 0.0
Q08945	TGsESSQTGTSTTSSR	2	S13(Phospho)	S(6): 0.0; Y(7): 0.0; Y(10): 0.0; S(13): 97.8; Y(21): 2.2
Q12846	STAGDTHLGGEDFDNR	2	S15(Phospho)	Y(13): 0.0; S(15): 100.0; T(18): 0.0
Q16637	DAIKENPEFDLHFEK	1	S6(Phospho); S9(Phospho)	T(3): 14.8; S(6): 92.6; S(9): 92.6; T(15): 0.0
Q1KMD3	KIVEPEVVGEsDsEVEGDAWR	2	S9(Phospho)	S(9): 100.0; T(13): 0.0; S(16): 0.0
P27797	DFQDYMEPEEGcQGsPQR	6		
Q92609	LHsSNPNLSTLDFGEEK	7	S7(Phospho); S10(Phospho)	S(3): 0.6; S(4): 6.5; S(5): 6.2; S(7): 93.4; S(10): 93.4
A1X283	APVQPQQsPAAAPGGTDEKPSGK	1	S3(Phospho)	S(3): 100.0; S(7): 0.0
P27361	EKTPSPKEEDEEPEsPPEK	3	T13(Phospho); T18(Phospho)	T(9): 0.0; T(13): 100.0; Y(15): 100.0; T(18): 0.0
Q9UQ35	LDSEDKDKEGKPLLK	6	T11(Phospho); T27(Phospho)	S(4): 100.0; T(10): 0.0; T(11): 0.0; S(14): 0.0; S(22): 2.4; S(25): 13.8; T(27): 83.7
Q01831	ASDPDEAGGPEGSEAVQSGtPEEPEPELEAEASK	1	S15(Phospho); S16(Phospho)	S(1): 0.0; T(8): 0.0; S(15): 100.0; S(16): 100.0; T(22): 0.0; S(23): 0.0; S(24): 0.0
P07339	HIEDTGsTPsIGENDLK	1		
Q8WUY3	IEDVGsDEEDDSGKDK	5	S8(Phospho)	S(2): 0.0; S(6): 0.6; S(8): 95.9; Y(12): 2.9; T(13): 0.6
O00629	DDDDIDLFGsDDEEESEEAK	3	C8(Carbamidomethyl); S11(Phospho)	S(11): 100.0; Y(17): 0.0
Q14699	KALVVPEPEPDSDsNQER	2	S5(Phospho); C17(Carbamidomethyl)	S(5): 100.0; T(13): 0.0; S(18): 0.0
Q96K21	ESsPLYsPTFsDSTSAVK	4	S15(Phospho)	T(6): 2.8; Y(10): 48.5; S(15): 48.5; T(23): 0.2
Q13200	KPAtPAEDDEDDDIDLFGsDNEEEDKEAAQLR	2	S10(Phospho)	S(10): 100.0; T(18): 0.0; S(23): 0.0
Q969R2	GGsSKDLLDNQSQEEQR	2	S6(Phospho); S9(Phospho)	T(4): 50.0; S(6): 50.0; S(9): 100.0; T(16): 0.0; T(17): 0.0; S(22): 0.0; T(27): 0.0
O43318	KANDEsNEHSDVIDSQELSK	1	S1(Phospho); S16(Phospho)	S(1): 99.8; T(6): 0.2; T(8): 0.6; T(10): 0.1; S(16): 2.9; S(17): 96.4
Q96K76	RPPsPDVIVLsDNEQPsSPR	2	S9(Phospho)	T(8): 9.6; S(9): 90.4; S(16): 0.0
P54259	EDDSIRPFKVETSDEEIHDLHQR	3	S12(Phospho); S19(Phospho); S21(Phospho)	Y(7): 0.7; T(9): 0.7; S(12): 98.6; S(19): 100.0; S(21): 100.0
O60238	DFQESsPQKGLEVER	1	S4(Phospho)	S(3): 12.1; S(4): 75.7; S(6): 12.1
O94885	DQGTYEDYVEGLR	1	S1(Phospho); C2(Carbamidomethyl)	S(1): 100.0; T(4): 0.0; T(10): 0.0; T(13): 0.0
Q8WZ73	NDADEKHPEHAEQNIR	1	S9(Phospho); S12(Phospho)	T(7): 12.8; S(9): 87.3; S(12): 99.9; S(15): 0.1
P27448	NKHPDEDAVEAEGHEVKR	2	S2(Phospho)	S(2): 82.0; T(4): 0.9; S(5): 8.5; T(6): 8.5; S(9): 0.1; S(17): 0.0; S(18): 0.0
Q86W92	TLHcEGTEINsDDEQESK	1	S4(Phospho)	S(4): 100.0; T(8): 0.0; S(12): 0.0
Q92538	RGEGDAPFSEPGTTSTQRPssPETATK	1	Y2(Phospho)	Y(2): 91.0; T(3): 0.8; S(4): 8.2; S(6): 0.0; Y(9): 0.0; T(10): 0.0
O43491	VATLNsEEESDPPTYK	1	S29(Phospho)	S(10): 0.0; S(11): 0.0; S(19): 0.6; S(27): 13.5; S(29): 42.9; S(30): 42.9
P35269	GLESTEAEMHIPSALEPSTSSsPR	4	S3(Phospho); S9(Phospho)	S(3): 100.0; T(7): 88.8; S(9): 11.2; S(14): 0.0; T(15): 0.0; S(16): 0.0; S(17): 0.0; T(18): 0.0
O60343	GTGSGGQLQDLDcsssDDEGAAQNSTKPSATK	1	S3(Phospho)	S(3): 100.0; S(6): 0.0; S(11): 0.0; S(13): 0.0
P46821	NFYEsDDDQKEK	1	T1(Phospho)	T(1): 100.0; Y(7): 0.0; S(8): 0.0; Y(9): 0.0; S(12): 0.0
Q9P2E9	HLVYESDKNKDEK	13	S15(Phospho)	S(1): 0.0; S(14): 16.2; S(15): 83.8
Q92597	RPPtPcsDPER	3	S1(Phospho); S5(Phospho); S7(Phospho)	S(1): 99.4; T(3): 0.7; S(5): 93.3; S(7): 13.2; S(8): 93.4; T(10): 0.0; S(11): 0.0; T(15): 0.0
P41227	DTSQSDKDLDDALDKLsDSLGQR	2	S7(Phospho)	S(7): 80.9; S(11): 15.8; S(15): 3.3; S(18): 0.0; T(20): 0.0; T(21): 0.0; S(23): 0.0; T(24): 0.0
Q9NQT8	TAsGSSVTSLDGTR	1	S3(Phospho)	S(3): 100.0; S(8): 0.0; T(10): 0.0; T(11): 0.0; S(13): 0.0
Q16643	DFQEETVKDYEAGQLYGLEK	1	S3(Phospho)	S(1): 11.7; S(3): 88.0; S(5): 0.3; S(6): 0.0; T(7): 0.0; S(9): 0.0; T(10): 0.0
P34932	cQTAEADsEsDHEVPEPESEMK	2		
O95425	NGDLDEVKDYVAKGEDVNR	1	S5(Phospho)	S(5): 100.0
Q9UEY8	GGVTGSPEASISGsKGDLK	1	S16(Phospho); S18(Phospho)	S(1): 0.0; S(10): 0.0; S(14): 48.6; S(16): 48.6; S(18): 51.4; S(20): 51.4
P57081	DALGDSLQVPVsPSSTTSSR	2	S3(Phospho)	S(3): 100.0
P11137	NKESKDPADETEAD	1	S11(Phospho)	T(9): 0.0; S(11): 100.0
P49736	DVEcGEGHFcHDNQTccR	1	S6(Phospho)	Y(4): 0.0; S(6): 100.0
Q5JTD0	RGsGDTSSLIDPDTSLSELR	3	S3(Phospho)	S(3): 99.3; T(5): 0.7
Q5VT52	DKRPEGYNLKEEDGR	4	S8(Phospho)	S(8): 100.0; S(15): 0.0
Q02952	FcGVNETENEDNTNRDsPIFDYSPR	1	S8(Phospho); C12(Carbamidomethyl); C20(Carbamidomethyl)	S(3): 0.1; S(4): 0.1; S(8): 99.8; T(21): 0.0
P27708	DPVQEAWAEDVDLR	1	S5(Phospho)	S(5): 100.0
Q9NXG2	VLGsEGEEEDEALsPAK	1	S11(Phospho)	T(2): 0.0; S(9): 11.3; S(11): 88.7
Q86VQ1	DLLHPSPEEEKRK	2	S3(Phospho); S5(Phospho)	S(3): 95.0; S(5): 51.8; S(6): 52.2; T(8): 0.9
Q9UK76	TDLEKDIISDTSGDFR	1	S3(Phospho)	S(3): 50.0; S(4): 50.0; S(7): 0.0; S(8): 0.0
Q92614	TEMDKsPFNSPSPQDSPR	1	S7(Phospho); S9(Phospho)	Y(1): 0.1; S(2): 0.1; S(4): 0.1; Y(5): 0.1; S(7): 14.6; S(9): 87.1; T(11): 97.9; T(16): 0.0; T(18): 0.0
Q5ZPR3	ENVEYIEREEsDGEYDEFGR	1	C3(Carbamidomethyl); S20(Phospho)	S(2): 0.0; S(20): 81.4; T(22): 18.6
O15173	DAEDAMDAMDGAVLDGR	2	T12(Phospho)	S(9): 0.0; Y(11): 50.0; T(12): 50.0; T(17): 0.0
Q9ULL5	QKSDAEEDGGTVSQEEEDRKPK	2	T4(Phospho); C8(Carbamidomethyl); S18(Phospho)	T(4): 0.3; T(11): 99.7; S(18): 100.0
O00499	RAVSEGcAsEDEVEGEA	1	S4(Phospho)	S(4): 87.7; S(6): 12.3; S(11): 0.0; T(15): 0.0
Q15149	KGSSGNAsEVSVAcLTER	1	S3(Phospho); S6(Phospho)	S(1): 8.9; S(2): 8.9; S(3): 81.2; S(6): 89.9; S(7): 10.7; S(8): 0.2; S(9): 0.2; Y(10): 0.0; S(13): 0.0; S(17): 0.0
Q96PD2	LEGDsDVDsELEDRVDGVK	2	T1(Phospho); C4(Carbamidomethyl)	T(1): 90.7; S(3): 9.3; S(5): 0.0; S(6): 0.0; Y(11): 0.0; T(13): 0.0
P19634	DDKHGSYEDAVHSGALND	4	S20(Phospho)	S(1): 0.0; T(4): 0.0; S(5): 0.0; S(6): 0.0; T(9): 0.0; T(14): 0.0; S(18): 0.1; S(20): 47.4; S(22): 47.4; S(23): 5.1
P31943	KVtNIISFDDEEDEQNSGDVFK	1	S6(Phospho)	T(2): 0.0; S(6): 90.3; T(9): 9.7
Q9Y2K5	LDQPVsAPPsPR	3	T4(Phospho)	S(1): 33.3; S(3): 33.3; T(4): 33.3; T(8): 0.0
Q7RTP6	EKPDsDDDLDIASLVTAK	4	S3(Phospho)	T(2): 0.5; S(3): 99.5; S(5): 0.0
P08651	STLQDsDEYSNPAPLPLDQHSR	4	S10(Phospho); S12(Phospho)	T(1): 0.0; S(6): 6.9; S(10): 93.1; S(12): 100.0; S(16): 0.0
P55884	KTsDANETEDHLESLIcK	2	S3(Phospho)	S(1): 50.0; S(3): 50.0; S(9): 0.0
Q15742	sFSEDVFQSVK	1	S3(Phospho)	S(3): 98.4; T(5): 1.6; S(15): 0.0
P78559	TIGGGDDsFNTFFSETGAGK	2	S4(Phospho)	S(4): 100.0; T(8): 0.0; T(14): 0.0
O14976	sASTDLGTADVVLGR	2	S16(Phospho)	S(2): 0.0; S(4): 0.0; S(13): 0.1; S(16): 89.0; S(21): 4.8; S(24): 4.8; S(25): 1.2
Q16204	IDDPTDSKPEDWDKPEHIPDPDAK	4	S6(Phospho); S10(Phospho)	S(6): 100.0; S(10): 100.0
Q32MZ4	ETVSEESNVLcLSK	1	S6(Phospho)	S(4): 1.7; S(6): 98.3; S(13): 0.0; S(26): 0.0
O60610	mEDLDQsPLVsSSDSPPRPQPAFK	4	S4(Phospho)	S(4): 100.0; S(10): 0.0
Q8IWE2	NDDDQKIEQDGIKPEDK	1	S35(Phospho); S39(Phospho)	S(6): 0.0; S(8): 0.0; S(9): 0.0; T(16): 0.0; T(24): 0.0; T(28): 0.0; S(35): 99.9; T(38): 81.0; S(39): 15.3; S(41): 3.0; S(42): 0.6; S(44): 0.1

Q13615	GKAsPFEEDQNR	1	C2(Carbamidomethyl); S3(Phospho)	S(3): 99.9; S(6): 0.1
Q14161	HHEEEIVHHK	5	T4(Phospho); C-Term(Oxidation)	S(3): 13.8; T(4): 83.6; S(7): 2.4; Y(9): 0.1; T(12): 0.1; S(23): 0.0; S(24): 0.0
P46821	cSsLGDLLGEGPR	1	S6(Phospho); S8(Phospho); S10(Phospho)	T(1): 0.0; S(6): 91.3; S(8): 6.2; S(10): 2.6; T(12): 2.5; S(14): 6.1; T(18): 90.3; Y(20): 18.4; Y(21): 77.1; S(23): 4.3; T(25): 1.1
Q5JTH9	HYNGEAYEDDEHHPR	1	S11(Phospho)	S(3): 0.0; S(11): 100.0
P46821	SYELPDGQVITIGNER	1	S6(Phospho); S11(Phospho)	S(1): 0.4; S(6): 99.7; S(11): 100.0; S(14): 0.0; T(17): 0.0; S(18): 0.0; T(19): 0.0; S(20): 0.0; Y(22): 0.0
Q7Z2X4	DHsPTPSVFNSDEER	3	S16(Phospho)	S(3): 0.0; S(5): 0.0; S(6): 0.0; S(7): 0.0; S(11): 0.0; S(16): 100.0
P55884	WLNSGRGDEASEEGQNGSsPK	1	S16(Phospho)	T(1): 1.2; S(11): 41.3; S(14): 12.2; S(16): 41.3; S(18): 3.8; S(28): 0.2
Q92733	DKYEPAAVSEQGDK	1	S11(Phospho); S13(Phospho)	S(11): 100.0; S(13): 100.0; T(19): 0.0
Q96N67	NKLEGDsDVDsELEDRVDGVK	3	S3(Phospho); S5(Phospho)	S(1): 88.6; S(3): 11.6; S(5): 99.8; S(10): 0.0; T(12): 0.0; T(14): 0.0; S(15): 0.0
Q9H2K8	GGEFDEFVNDDtDDDLPISK	1	S7(Phospho)	S(7): 100.0; S(14): 0.0; T(18): 0.0; S(19): 0.0
Q9UPU7	NINMDNDLEVLTSSPtR	2	T19(Phospho)	T(4): 0.0; S(5): 0.0; S(13): 97.8; T(19): 2.2
Q9H3K6	DGEDQTQDTELVETRPAGDGTFQK	1		
P49023	VADAKGDsEsEEDEDLEVPVPSR	4	S5(Phospho)	S(5): 50.0; S(6): 50.0
Q9Y2W1	QRSDDESPsTSSGSSDADQRDPAAPEPEEQEER	5	S13(Phospho)	S(2): 0.0; S(13): 95.3; T(15): 4.6; T(27): 0.0; T(28): 0.0
P00519	AAVQELSSsILAGEDPEER	1	C2(Carbamidomethyl); S3(Phospho); C6(Carbamidomethyl)	S(1): 0.1; S(3): 99.8; S(5): 0.1
Q86VM9	DDDRTPGLHGDcDDDKYR	3	S11(Phospho)	S(3): 0.0; S(11): 100.0; S(18): 0.0
Q09666	DELTDLDQSNVTEETPEGEEHHPVADTENKENEVEEVKEEGPK	2	T4(Phospho); S14(Phospho)	T(4): 2.0; S(6): 98.0; S(10): 1.7; S(12): 86.5; S(14): 11.8
O95817	DSISAVSSEKVSPsKsPsLSPSPPSPLEK	4	S8(Phospho); S12(Phospho)	S(1): 0.0; S(2): 0.0; S(7): 0.0; S(8): 100.0; S(12): 100.0
Q05655	ENPPVEDssDEDDKR	2	S3(Phospho)	S(1): 49.4; S(3): 49.4; S(5): 1.1; S(6): 0.0; Y(12): 0.0
Q76I76	ADAPDAGAQsDSELPSYHQNDVSLDR	1	S5(Phospho)	T(3): 0.0; T(4): 5.7; S(5): 94.3
P46821	NKLEGDsDVDSELEDR	1	S17(Phospho)	T(1): 0.0; T(4): 0.0; Y(9): 8.4; S(12): 40.7; S(14): 40.7; T(15): 1.8; S(17): 8.4; S(20): 0.0; S(21): 0.0; S(28): 0.0
Q9H2G2	KQELEEIcHDLEAR	2	S5(Phospho); S11(Phospho)	S(5): 87.3; T(9): 13.0; S(11): 99.7
Q8NEY1	NTDKNGEELHGGK	2	S5(Phospho)	S(1): 0.0; Y(3): 0.1; S(5): 99.1; S(7): 0.8
O14976	VDDDsLGEFPVTNSR	1	S13(Phospho); S16(Phospho)	S(2): 0.3; S(4): 1.0; S(13): 98.8; S(16): 99.9; S(21): 0.0; S(24): 0.0; S(25): 0.0
Q86W56	HFSQSEEtGNEVFGALNEEQPLPR	3	S8(Phospho)	S(1): 0.0; S(8): 83.4; T(10): 14.1; S(12): 2.5; T(23): 0.0; T(24): 0.0
A2RRP1	TLSSsSMDLSR	1	S11(Phospho)	S(11): 100.0; S(13): 0.0; Y(15): 0.0; S(18): 0.0
O60343	GHDLNEDGLVSWEEYK	1	S3(Phospho); C5(Carbamidomethyl); S6(Phospho); S9(Phospho)	T(1): 10.5; S(2): 10.5; S(3): 70.9; T(4): 20.0; S(6): 87.1; S(9): 50.5; S(11): 50.5; T(15): 0.0; S(16): 0.0; T(18): 0.0
Q9NWV8	KGGEFDEFVNDDtDDDLPISK	1	N-Term(Acetyl); S8(Phospho)	S(7): 11.4; S(8): 87.1; T(10): 1.6; S(20): 0.0
Q5SW79	AGGGRPSsPSPSVVSEK	3	T7(Phospho)	T(4): 9.1; S(5): 9.1; T(7): 39.6; S(8): 39.6; S(17): 2.2; T(21): 0.1; Y(22): 0.1; T(23): 0.1; S(31): 0.0
Q13045	IIYDsDsESEETLQVK	2	S8(Phospho)	S(8): 100.0; S(12): 0.0
P78559	SAGGSSPEGGEDsDREDGNYcPPVKR	1	S4(Phospho); C7(Carbamidomethyl)	S(4): 100.0; S(11): 0.0; T(13): 0.0; S(16): 0.0
Q92538	RSStSSEPTPTVK	1	C3(Carbamidomethyl); S4(Phospho)	S(1): 0.2; S(4): 99.8; S(12): 0.0
Q9UJU6	QGsPDQVSPVSEMTSTSLYQDK	3		
Q69YU3	ESEDKPEIEDVGsDEEEEKK	3	S3(Phospho)	S(3): 95.4; T(4): 4.6
P22314	AsGQAFELILsPR	2	S3(Phospho)	S(3): 100.0; Y(12): 0.0; S(13): 0.0
P11717	QNALLFAEEEDGEAGAEDKRsQEETPGHR	1	S12(Phospho)	S(3): 2.7; S(4): 14.5; S(12): 82.8; T(18): 0.0
Q86VX9	DGKYDLDFKsPTDPSR	1	S1(Phospho)	S(1): 91.4; Y(2): 8.6; T(6): 0.0; S(8): 0.0; S(14): 0.0; S(17): 0.0
Q92769	RAsLQASTAAPEAR	2	C3(Carbamidomethyl); S8(Phospho); S10(Phospho)	S(8): 100.0; S(10): 100.0
Q13428	IsDAELEAELEK	3	S14(Phospho)	T(1): 0.0; S(2): 0.0; S(8): 0.0; S(14): 100.0
P80723	VSPPAPGsAPEtPEDK	2	S15(Phospho)	T(2): 0.0; T(13): 17.3; S(15): 82.7; S(21): 0.0; S(23): 0.0; S(27): 0.0; S(28): 0.0; S(33): 0.0; S(34): 0.0
O15085	VSAEAEVAPVSPEVTQEVVEEHcAsPEDK	6	T1(Phospho)	T(1): 50.0; S(4): 50.0; S(9): 0.0
Q12906	SQPsETERLTDDY	3	S11(Phospho)	S(11): 98.9; S(13): 1.1
Q15154	EYIPGQPPLSQSSDSsPTR	2	S7(Phospho)	S(7): 100.0; T(22): 0.0; T(24): 0.0
Q6PD62	LSKsQGGEEEGPLSDK	1	S5(Phospho)	S(5): 100.0; T(10): 0.0
Q15648	DREEGHPEPEDEEEEREHEVMTAGK	1		
P23634	DDGSWEVIEGYR	1	S14(Phospho)	T(1): 0.0; S(14): 100.0
Q8NFG4	KEsAPQVLLPEEEK	1	S3(Phospho)	S(3): 100.0; S(9): 0.0; S(12): 0.0; S(13): 0.0; S(14): 0.0
Q93008	IVRGDQPAASGDsDDDEPPPLPR	1	S18(Phospho); C-Term(Oxidation)	T(9): 0.0; S(11): 1.1; S(18): 94.4; S(27): 4.5
Q9UPU7	DsSTSPGDYVLSVSENSR	7	S11(Phospho)	T(2): 0.0; S(3): 0.0; S(11): 100.0; T(17): 0.0
P13489	NFSDNQLQEGK	2	C4(Carbamidomethyl)	
O75122	EGQPSPADEKGNDsDGEGESDDPEK	1	S3(Phospho)	S(3): 100.0; S(9): 0.0; S(16): 0.0; S(17): 0.0; S(20): 0.0
P29692	TPQASTYSYETSDLcYTAEKKsPSEAR	2	T4(Phospho); S19(Phospho)	T(4): 100.0; S(19): 100.0
Q9C0C2	QKsDAEEDGGTVsQEEEDR	1	S3(Phospho)	S(3): 100.0; S(9): 0.0; S(11): 0.0
Q14699	KLSVPtsDEEDEVPAPKPR	1		
Q8WXE0	SAGGssPEGGEDsDREDGNYcPPVKR	2	S19(Phospho)	S(2): 0.0; S(5): 0.0; S(14): 0.0; S(19): 100.0
Q96N67	tFcGTPDYIAPEIIAYQPYGK	1	S3(Phospho)	S(1): 0.6; S(3): 99.4; T(7): 0.0
P62191	SASQSsLDKLDQELK	2		
Q9C0C2	cDSSPDsAEDVRK	4	S3(Phospho)	S(3): 90.3; Y(7): 9.6; S(9): 0.1
Q9H6S0	NKDQSEQETSDADQHVTSNASDSESSYR	1	T6(Phospho); C12(Carbamidomethyl)	S(2): 0.1; S(3): 0.1; T(6): 7.9; S(9): 91.9; T(13): 0.0; T(14): 0.0
P52948	TKFAsDDEHDEHDENGATGPVK	1	S7(Phospho)	S(2): 0.0; S(7): 91.3; S(9): 7.9; Y(11): 0.7
P40818	TSFSVGsDDELGPIR	1	S3(Phospho)	S(1): 4.3; Y(2): 4.3; S(3): 45.6; S(4): 45.6; T(8): 0.1
P78559	KDEEGQKEEDKPR	7		
Q8IY81	SKsMDLGIADETK	1	S5(Phospho); S7(Phospho)	S(5): 2.5; S(7): 97.9; S(8): 99.7; S(19): 0.0; T(20): 0.0; T(23): 0.0; S(24): 0.0
Q96KR1	AAPEASsPPAsPLQHLLPGK	5	S4(Phospho)	S(4): 100.0
Q13409	SDAEEDGGTVsQEEEDRKPK	2		
Q6PKG0	NIDEHANEDVER	1	S7(Phospho); S11(Phospho)	T(2): 0.0; T(4): 0.0; S(7): 100.0; S(11): 99.6; Y(13): 0.4
Q96B23	QITQEEDDsDEEVAPENFFSLPEK	3	S4(Phospho)	S(2): 0.0; S(4): 99.0; S(6): 1.0; T(8): 0.0; S(9): 0.0; T(10): 0.0
Q8IWS0	WRsLQQLAEER	1		
P08670	HSGASAEVQKEEEKK	3	S9(Phospho)	T(8): 7.7; S(9): 92.3
O95477	VQIPVSRPDPEPVsDNEEDsYDEEIHDPR	3	C3(Carbamidomethyl); S18(Phospho)	S(2): 0.0; T(8): 0.0; S(18): 100.0; S(24): 0.0
Q5TH69	KAsPEAASTPRDPIDVDLPEEAER	1	C13(Carbamidomethyl); S16(Phospho)	T(2): 0.0; S(4): 0.0; S(16): 50.0; S(21): 50.0
P67936	RsQSTTFNPDDMSEPEFK	3		
Q9Y4E8	SQGDEAGGHGEDRPEPLsPK	3	S12(Phospho); C28(Carbamidomethyl); C-Term(Oxidation)	S(3): 0.8; T(6): 2.3; S(12): 74.8; S(16): 22.1; T(31): 0.0
Q8TEA8	KLSVPTsDEEDEVPAPKPR	2	S4(Phospho)	S(1): 7.8; S(3): 7.8; S(4): 84.4; S(11): 0.0; S(12): 0.0
Q05519	DSQDAsAEQsDHDDEVASLASASGGFGTK	4	S14(Phospho); C-Term(Oxidation)	S(14): 100.0
P22059	KIALESEGRPEEQMESDNcsGGDDDWTHLSSK	4	S5(Phospho); S8(Phospho)	S(5): 100.0; S(8): 100.0; S(13): 0.0; S(15): 0.0; T(17): 0.0; T(20): 0.0; T(25): 0.0
Q8TDM6	DNNQFASASLDR	1	S7(Phospho)	T(2): 0.0; S(5): 9.0; S(7): 81.0; S(10): 9.0; S(11): 1.1
Q13501	DHYEATAMHR	1	C19(Carbamidomethyl); S20(Phospho)	S(6): 0.1; S(16): 5.4; S(20): 94.5; T(27): 0.0; S(30): 0.0; S(31): 0.0
Q9Y608	SKsMDKSDEELQFPK	2	S10(Phospho)	S(2): 0.0; S(4): 0.0; T(6): 0.0; T(7): 0.2; S(10): 97.0; S(13): 1.4; S(14): 1.4
Q6VY07	TNQELQEINR	1	S3(Phospho)	T(1): 1.5; S(3): 76.3; S(4): 10.3; S(6): 1.5; S(9): 10.3; S(15): 0.0; T(16): 0.0
Q8NHZ8	RVGEQDSAPTQEKPtSPGK	2	S11(Phospho)	S(11): 100.0; S(20): 0.0; S(21): 0.0
O95831	NKEDQYDHLDAADMTk	2		
Q92614	RSSDsWEVWGSASTNR	2	S7(Phospho); S11(Phospho)	S(7): 100.0; S(11): 100.0
Q14247	DGQVINETsQHHDDLE	2	T5(Phospho); T15(Phospho); Y25(Phospho)	T(3): 83.8; T(5): 16.4; S(9): 99.7; T(15): 0.2; S(21): 16.1; S(22): 83.2; Y(25): 0.7; S(30): 0.0
O75717	DKKAEGAATEEEGTPK	1	S10(Phospho)	S(10): 100.0; S(28): 0.0; S(30): 0.0
Q9BYX2	VEAKEEsEEsDEDMGFGLFD	1	S4(Phospho); C7(Carbamidomethyl); S9(Phospho)	S(4): 100.0; S(9): 100.0
O60343	LENEGsDEDIETDVLYSPQMALK	4	S3(Phospho); S6(Phospho)	S(3): 100.0; S(6): 100.0
Q9Y613	APsDsSLGTPSDGRPELR	3	S1(Phospho); C7(Carbamidomethyl)	S(1): 100.0

Q9UM82	KVPQVSTPTLVEVSR	1	S1(Phospho)	S(1): 100.0; Y(5): 0.0; S(7): 0.0; Y(8): 0.0; S(11): 0.0
Q9UPZ3	GLAEVQQDGEAEEGATsDGEK	4	S3(Phospho); S5(Phospho); C10(Carbamidomethyl)	S(3): 73.9; S(5): 84.7; S(9): 13.4; S(11): 27.0; S(14): 1.0; T(16): 0.1; S(18): 0.0
Q5JSL3	VTPQEEADsDVGEEPDSENTPQK	2	T13(Phospho)	S(3): 0.0; S(6): 0.1; T(13): 97.7; T(21): 0.5; T(25): 1.7
Q8IYB3	VQSLEGEKLsPK	1	T4(Phospho)	T(4): 100.0; S(11): 0.0
P52948	VNFSEEGEtEEDDQDSSHSSVTTVK	2	S6(Phospho)	Y(1): 0.0; S(6): 100.0; S(15): 0.0
Q96ST2	EKTEAEPTHVNEEKLEHETAVTVSEEVSK	5	S5(Phospho); S7(Phospho)	S(5): 100.0; S(7): 100.0; S(14): 0.0
P35613	SVEDVRPHHtDANNQSAcFEAPDQK	1	S13(Phospho)	S(13): 100.0
O15439	SAKsEESLTSLHAVDGDSK	3	T13(Phospho)	S(5): 0.0; T(13): 98.6; T(15): 1.4
Q96JG6	IAAEEEEENDGHYQEEEEGGAHsLKDVcDLR	1	S8(Phospho)	S(1): 0.0; Y(3): 0.0; Y(6): 0.1; S(8): 99.7; S(10): 0.1
Q02952	DGDKsPMSSLQISNEK	2		
O60343	SRTPsASNDDQQE	1	S3(Phospho); C5(Carbamidomethyl); S6(Phospho)	T(1): 49.6; S(2): 49.6; S(3): 33.3; T(4): 33.3; S(6): 33.3; S(9): 0.8; S(11): 0.1; T(15): 0.0; S(16): 0.0; T(18): 0.0
Q14697	AKSEENTKEEKPDSK	7		
P28715	GDHAsLENEKPGTGDVcSAPAGR	1	S10(Phospho); S12(Phospho)	S(10): 100.0; S(12): 100.0
P26006	DDSHsAEDsEDEKEDHKNVR	2	S4(Phospho)	S(1): 0.0; S(4): 99.6; T(6): 0.4; T(10): 0.0; Y(13): 0.0
Q13017	VQEAQDsDsDSEGGAAGGEADMDFLR	6	S4(Phospho); S7(Phospho)	T(2): 0.1; S(4): 99.9; S(7): 100.0; T(13): 0.0; T(14): 0.0; S(15): 0.0
Q32MZ4	KGSsGNAsEVSVAcLTER	3	S7(Phospho)	S(3): 0.1; T(6): 7.6; S(7): 84.8; S(9): 7.6; T(12): 0.0; S(15): 0.0
P11047	NRDNDPNDYVEQDDILIVK	1	C8(Carbamidomethyl); C11(Carbamidomethyl); C20(Carbamidomethyl)	
Q14697	DKGDEEEEGEEKLEEK	1		
O94808	NAEQNGPVDGQGDNPGPQAAEQGTDTAVPSDSDK	1	S3(Phospho); C6(Carbamidomethyl)	S(3): 100.0; S(4): 0.0
Q8TEW0	VAAAAGSGPsPPGsPGHDR	4	S3(Phospho)	S(1): 50.0; S(3): 50.0; T(12): 0.0
P51812	TEAEPTHVNEEKLEHETAVTVSEEVSK	1	S3(Phospho)	S(3): 100.0
P50502	DLEAEHVEVEDTTLNR	2	S13(Phospho); S16(Phospho)	S(12): 0.8; S(13): 99.2; S(16): 100.0
O94979	SsDEENGPPsSPDLDR	1	S8(Phospho)	S(2): 0.0; S(8): 100.0; S(13): 0.0
P01891	SLSSsLDDTEVKK	3	S17(Phospho); S20(Phospho); C24(Carbamidomethyl)	S(4): 0.0; Y(5): 0.0; S(6): 0.0; S(10): 0.0; S(11): 0.0; S(13): 0.0; S(17): 99.3; S(20): 96.7; T(22): 4.0
Q9C0C2	NVPHEDIcEDSDIDGDYR	1	C5(Carbamidomethyl); T12(Phospho)	S(1): 0.0; S(10): 1.0; T(12): 99.0
Q9UKS6	DSYVGDEAQSK	1	S22(Phospho)	T(3): 0.0; S(9): 0.0; S(12): 0.0; T(15): 0.0; S(22): 99.9; S(26): 0.1
P50995	GGVtGSPEASISGsKGDLK	2		
Q86WB0	YAALsVDGEDENEGEDYAE	2	S10(Phospho); S16(Phospho)	S(1): 0.0; T(5): 0.4; S(7): 97.3; S(10): 2.7; S(16): 99.5; S(22): 0.1
Q9H6Z4	SQsTTFNPDDMSEPEFKR	5	S13(Phospho); C21(Carbamidomethyl)	S(1): 0.1; S(5): 0.1; S(6): 0.2; S(13): 99.6; Y(20): 0.1
Q16555	VFDDEsDEKEDEEYADEKGLEAADK	4	C8(Carbamidomethyl); T13(Phospho)	Y(3): 0.0; S(11): 0.1; T(13): 99.9
Q08AE8	KVVDYSQFQEsDDADEDYGRDSGPPTK	4	S3(Phospho)	S(1): 0.7; S(3): 99.3; S(7): 0.0
P43490	KKNEELEQLYQDQEVPKPK	2		
Q9BX95	APsDSSLGTPSDGRPELR	2	T5(Phospho)	S(3): 0.0; T(5): 100.0
Q13428	TAKDsDDDDDVAVTVDRDR	2	S13(Phospho)	S(11): 0.9; S(13): 99.1; T(17): 0.0; S(18): 0.0; T(19): 0.0; T(20): 0.0; S(21): 0.0
Q6R327	EREEsEDELEEANGNNPIDIEVDQNK	1	S7(Phospho); S10(Phospho)	T(5): 96.3; S(7): 3.7; T(8): 3.7; S(10): 96.3
P53618	TKFAsDDEHDEHDENGATGPVKR	2		
Q69YQ0	GKEEELQDVRDQLEQAQEER	2	S3(Phospho)	S(2): 0.5; S(3): 47.1; T(4): 47.1; S(5): 4.8; S(6): 0.5; T(9): 0.0; T(11): 0.0
O43493	NDFTEEEEAQVR	1	S10(Phospho)	S(2): 0.0; T(8): 3.8; S(10): 92.4; T(14): 3.8; S(18): 0.0
O00178	mDSAGQDINLNsPNK	1	S7(Phospho); C9(Carbamidomethyl); S10(Phospho)	S(7): 100.0; S(10): 100.0; T(25): 0.0; S(26): 0.0
Q13131	SINKLDsPDPFK	1	S11(Phospho)	S(1): 0.0; T(7): 0.1; S(11): 99.9; T(13): 0.1; T(15): 0.0
Q01433	LEASYSDsPPGEEDLLVHVAEGSK	1	S3(Phospho)	S(3): 100.0; S(7): 0.0; T(8): 0.0
Q8N8S7	SGVSsDNEDDDDEEDGNYLHPSLFASK	2		
Q15154	SRTGsESSQTGTSTTSSR	3	S6(Phospho)	T(2): 0.2; S(6): 89.6; S(9): 10.1; S(10): 0.2
Q9UDY2	KIKDPDASKPEDWDER	5	S5(Phospho); S12(Phospho)	S(4): 50.1; S(5): 50.1; S(12): 99.8
Q9C0C2	IHQDsESGDELSSSSTEQIR	2		
P40855	ESsPLYsPTFSDSTSAVK	6	S9(Phospho)	T(3): 0.0; S(8): 5.3; S(9): 47.0; S(11): 47.0; T(16): 0.6
Q8TCJ2	SEDSKKPADDQDPIDALSGDLDScPSTTETSQNTAK	1	S8(Phospho); S9(Phospho)	S(8): 100.0; S(9): 100.0
Q9UK76	STsQGSINSPVYSR	3	S2(Phospho)	S(2): 92.5; S(3): 7.5; S(6): 0.0; S(7): 0.0
O14617	RsQTSTADSDLKEDGISSR	4	S4(Phospho)	S(4): 100.0
Q9NS91	DSAIPVEsDTDDEGAPR	2	S9(Phospho)	T(6): 0.0; S(9): 100.0
Q6P1N0	TLsPTPSAEGYQDVR	2	S21(Phospho)	S(21): 99.2; T(26): 0.8
Q9HCD6	DKTEDDADREAQLLEMR	2	C2(Carbamidomethyl); S9(Phospho)	S(3): 0.0; Y(4): 0.0; T(8): 5.5; S(9): 44.1; T(11): 44.1; S(12): 5.5; T(13): 0.7; S(16): 0.0
Q92685	DQLIYNLLKEEQTPQNK	1	S3(Phospho); C11(Carbamidomethyl)	S(1): 50.0; S(3): 50.0
Q969E4	LPSsPVYEDAASFK	2	S18(Phospho)	S(18): 100.0
P52292	DLEEDHAcIPIKK	4	T10(Phospho)	S(3): 0.0; S(4): 0.0; T(10): 90.8; S(11): 9.2
Q14247	LEFQQQLGEAPSDAsP	2	S4(Phospho)	S(3): 50.0; S(4): 50.0; Y(7): 0.0; S(12): 0.0
Q9NRY4	KQKGsEENLDEAR	2	S7(Phospho)	T(1): 0.0; S(2): 0.0; S(4): 0.0; S(7): 99.9
Q3V6T2	GRsIDQDYER	1	S3(Phospho)	S(1): 33.3; S(2): 33.3; S(3): 33.3
Q9BYT3	SRTsVQTEDDQLIAGQSAR	4	C6(Carbamidomethyl); S9(Phospho)	S(7): 0.5; S(8): 6.6; S(9): 85.7; T(11): 6.6; S(12): 0.5; S(13): 0.1
P46821	DVAEAKPELSLLGDGDH	2	S3(Phospho); S7(Phospho); S11(Phospho)	S(2): 50.0; S(3): 50.0; Y(6): 1.0; S(7): 98.8; T(9): 0.2; S(11): 99.9; S(13): 0.0; T(14): 0.0; S(15): 0.0
Q9H0D6	VELPsEEQVSGSQGPSEEKPAPLATEVFDEK	2	S4(Phospho); S6(Phospho)	S(4): 100.0; S(6): 100.0
P05023	KsTAALEEDAQILK	1		
O43847	NsIQFTDGYEVK	2	S14(Phospho)	S(6): 2.2; S(14): 84.4; S(16): 13.4
Q9P227	REPEEHQVEEEHRK	4	S1(Phospho); S12(Phospho)	S(1): 100.0; S(12): 100.0
P27361	SQsLPNSLDYTQTSDPGR	4	Y15(Phospho)	T(9): 0.0; T(13): 0.9; Y(15): 98.9; T(18): 0.1
P28290	RHDEDEDDSLKDR	2	S3(Phospho); C9(Carbamidomethyl)	S(1): 49.9; S(3): 49.9; T(11): 0.2; T(15): 0.0; T(17): 0.0
Q13557	SHsSSEAYEPR	2	T9(Phospho); T14(Phospho)	S(8): 45.3; T(9): 45.3; S(11): 13.5; S(12): 13.5; T(14): 73.0; T(15): 9.4
Q96CV9	ENPPVEDsSDEDDKR	2	S6(Phospho)	T(5): 6.2; S(6): 93.8; S(8): 0.0; Y(13): 0.0
Q9HCN4	KSEDSKKPADDQDPIDALSGDLDScPSTTETSQNTAK	1	S12(Phospho)	T(2): 0.0; S(12): 99.9; T(14): 0.1
O75410	RAEPHTPSSHDAEEDEPLKESR	1	T5(Phospho); C15(Carbamidomethyl)	T(5): 100.0; S(12): 0.0; T(13): 0.0; S(14): 0.0
Q9BPX3	VVEAVNSDsDsEFGIPK	2	C4(Carbamidomethyl); S11(Phospho)	T(1): 0.0; T(7): 0.0; S(11): 100.0; S(17): 0.0
P46821	DNLTLWTSDMQGDGEEQNK	1	S3(Phospho); S7(Phospho)	S(2): 11.2; S(3): 88.8; Y(6): 1.5; S(7): 87.4; T(9): 10.9; S(11): 0.2; S(13): 0.0; T(14): 0.0; S(15): 0.0
Q8TCJ2	YTGEEDGAGGHsPAPPQTEEcLR	1	S8(Phospho)	S(8): 90.3; S(9): 9.7
P78559	EVDEQMLNVQNK	1	S17(Phospho)	S(10): 0.0; S(13): 0.0; S(14): 1.5; S(15): 10.5; Y(16): 10.5; S(17): 77.5
Q96ST2	RQAQQERDELADEIANSSGK	2	S4(Phospho); S6(Phospho)	S(4): 100.0; S(6): 100.0
O75351	YDTNNNEEEEGEQFDFDsGDEIPEADR	2	S14(Phospho)	S(5): 0.0; S(14): 100.0; S(20): 0.0
Q08AD1	EVDGLLTSEPMGsPVSSK	1	S12(Phospho)	Y(8): 0.4; S(12): 99.6
P78559	SRDsGDENEPIQER	1	S4(Phospho)	S(4): 100.0; T(8): 0.0; T(14): 0.0
P05386	QENcGAQQVPAGPGtSTPPSsPVR	2	S4(Phospho); S7(Phospho); C-Term(Oxidation)	S(4): 100.0; S(7): 100.0
Q9H6Z4	DSEGDSLGARPGLPYGLsDDESGGGR	2	S6(Phospho); S13(Phospho); C21(Carbamidomethyl)	S(1): 2.6; S(5): 14.3; S(6): 83.8; S(13): 99.4; Y(20): 0.0
Q16513	DAGGPRPEsPVPAGR	1	S2(Phospho)	S(2): 50.0; S(3): 50.0; S(10): 0.0; S(11): 0.0
Q92900	VQEKPDsPGGSTQIQR	2	S8(Phospho); S11(Phospho)	S(1): 0.0; S(8): 1.6; S(11): 89.9; T(12): 88.8; Y(13): 19.7
P42575	LDsSAcLHAVGDK	2	S5(Phospho); C8(Carbamidomethyl)	S(5): 100.0; S(11): 0.0
Q14680	EGKPEIEGKPEsEGEPGSETR	1	S5(Phospho)	S(1): 0.0; S(5): 100.0; T(10): 0.0; S(12): 0.0
Q86W92	KVEEEGsPGDPDHEASTQGR	1	S1(Phospho)	S(1): 9.1; S(3): 9.1; S(5): 1.1; T(7): 80.8; S(11): 0.0
P11137	LGAGGGsPEKSPSAQELK	2	T9(Phospho); S16(Phospho); S19(Phospho)	T(9): 0.1; S(11): 99.9; S(15): 50.0; S(16): 50.0; S(19): 100.0
O43934	AQsYPDNHQEFSDYDNPIFEK	4	S13(Phospho); C-Term(Oxidation)	S(4): 0.0; S(13): 0.5; S(14): 0.5; S(24): 0.5; T(30): 98.5
O00499	TTKSPsDSGYSYETIGK	5	S3(Phospho); S8(Phospho)	S(1): 8.3; S(3): 91.7; S(8): 100.0; T(12): 0.0

Q15742	SPFEIIsPPAsPPEMVGQR	5	S3(Phospho); S6(Phospho)	S(1): 6.7; S(3): 93.3; S(6): 100.0
Q9Y4E1	RSsTLSQLPGDK	1	T2(Phospho); S4(Phospho)	T(2): 100.0; S(4): 100.0
P16949	tSStcSNESLSVGGTSVTPR	4		
Q70EL1	DNFDMcSSsFTSSK	3	S12(Phospho)	S(8): 0.0; Y(10): 1.9; S(12): 96.3; T(16): 1.9
Q12983	QKEDVEVVGGsDGEGAIGLSSDPK	1	S5(Phospho)	S(2): 0.0; S(3): 0.0; S(5): 100.0
P46379	SRsQPcDLDAR	2	S2(Phospho); S11(Phospho)	S(2): 100.0; S(11): 95.1; T(16): 4.0; T(17): 0.9; S(23): 0.0
P62328	HSTPSNSSNPSGPPsPNSPHR	1	T3(Phospho)	T(1): 11.4; T(3): 88.6; S(11): 0.0; T(14): 0.0
Q96QE2	HVDSLSQRsPK	2	S12(Phospho)	S(2): 0.0; Y(4): 0.0; S(7): 0.0; S(12): 100.0
Q5VZ89	SPQNDDHsDTDSEENRDNQQFLTTVK	1	S5(Phospho); S10(Phospho)	S(2): 50.0; S(5): 50.0; S(10): 97.1; T(12): 2.9; S(19): 0.0; S(20): 0.0
P35579	RDsFDDRGPSLNPVLDYDHGSR	1	S7(Phospho)	S(7): 100.0
Q8N122	SsGFAFDPSVNYSK	2	S10(Phospho); S14(Phospho)	T(4): 0.0; S(5): 0.0; S(6): 0.0; T(8): 0.9; S(10): 97.2; S(14): 50.9; T(16): 50.9
Q9Y2D5	KQNsPVAPTAQPK	1	S12(Phospho)	S(9): 0.8; S(11): 3.6; S(12): 95.6
P55196	AGGLQDsDTEDEcWsDTEAVPR	3	S6(Phospho)	S(1): 1.4; S(6): 86.6; S(9): 10.6; S(10): 1.4; T(17): 0.0
P29966	KAEENAsQEEEEAAEDEGGEEDLASELR	1	S15(Phospho)	S(15): 90.1; S(16): 9.0; S(18): 0.9
Q07157	tQTPPVsPAPQPTEER	3	S1(Phospho)	S(1): 94.3; S(6): 5.7; T(12): 0.0
Q92574	mEDLDQSPLVSSSDsPPRPQPAFK	3	S5(Phospho)	S(5): 100.0; Y(8): 0.0
P02545	DHSSQSEEEVVEGEKEVEALK	1	T8(Phospho); S11(Phospho)	S(1): 0.0; S(6): 0.0; S(7): 1.1; T(8): 98.9; S(11): 91.0; T(13): 9.0
Q15459	SRsGEGEVSGLMR	3	S13(Phospho)	S(4): 0.0; S(13): 100.0
Q9UJM3	DGEDQTQDTELVETRPAGDR	1	S3(Phospho)	S(3): 100.0; T(7): 0.0; S(8): 0.0; S(9): 0.0; T(10): 0.0; Y(11): 0.0; S(12): 0.0
Q02818	DRPGDEDWVHR	1	S5(Phospho)	S(5): 100.0; T(8): 0.0
P35579	HAsAPSHVQPSDSEK	6		
P28290	DLFSLDSEDPSPAsPPLR	1	S6(Phospho)	T(1): 0.3; S(6): 99.4; S(11): 0.3; S(12): 0.0; S(13): 0.0; T(14): 0.0
Q96CV9	GLsGEEEDDEPDccNDER	1	S4(Phospho)	T(1): 33.3; S(2): 33.3; S(4): 33.3; Y(9): 0.0
P55081	RKtSDANETEDHLESLIcK	6	S11(Phospho); S13(Phospho)	S(11): 100.0; S(13): 100.0
Q15185	ESDKEDGREsPSYDTPSQR	2	S6(Phospho); C-Term(Oxidation)	S(6): 100.0; S(11): 0.0
O60716	DASDDLDDLNFFNQK	2	Y7(Phospho)	S(1): 0.0; S(3): 1.6; S(4): 1.6; S(6): 1.6; Y(7): 82.7; S(10): 1.6; T(11): 10.9
Q13439	LKFsDDEEEEEVVK	1	S4(Phospho); S5(Phospho)	S(1): 6.5; T(3): 48.4; S(4): 48.4; S(5): 48.4; T(7): 48.4; T(14): 0.0
P46821	GEKELSSEPQtPPAQK	1	T1(Phospho); C11(Carbamidomethyl)	T(1): 91.2; T(2): 8.1; T(4): 0.8; T(7): 0.0; S(8): 0.0; T(9): 0.0; Y(10): 0.0; Y(12): 0.0; T(14): 0.0
Q09666	HSPTEDEESAKAEADAYIR	1	S8(Phospho)	Y(3): 0.0; T(6): 0.0; S(8): 94.8; T(12): 5.2
P46821	sADSVMAEQVALSR	1	S10(Phospho)	Y(2): 0.0; S(5): 0.1; S(7): 5.9; T(8): 43.6; S(10): 43.6; S(13): 0.8; S(14): 5.9; S(21): 0.0
Q01082	ISQDADLKtPTKPK	1	S3(Phospho); S6(Phospho); S7(Phospho)	T(1): 7.3; S(2): 7.3; S(3): 85.5; S(6): 7.9; S(7): 92.1; S(11): 99.9; T(13): 0.1; S(14): 0.0
O43707	GHTDTEGRPPsPPPTSTPEK	4		
Q96JM3	KEKtPELPEPSVK	1	S7(Phospho); S12(Phospho); S16(Phospho)	S(7): 100.0; S(12): 100.0; S(16): 100.0
O75382	EDLRLPEGDLGKEIEQK	2	S4(Phospho); C-Term(Oxidation)	S(4): 100.0
P28715	SPSLSPSPPsPLEK	1	S12(Phospho)	S(10): 0.1; S(12): 99.9
Q5TH69	NIIHGSDSVESAEK	1	S7(Phospho)	Y(1): 0.0; S(2): 0.0; S(4): 0.2; S(7): 99.7; S(14): 0.0
Q96B97	ESKEEETsIDVAGKPNEVTK	1	S1(Phospho)	S(1): 100.0
P35579	KDsLTQAQEQGNLLN	1	C14(Carbamidomethyl)	
Q96D46	DDDDIDLFGsDDEEESEEAKR	2	S8(Phospho); T10(Phospho)	S(2): 0.0; S(8): 100.0; T(10): 100.0
Q5VW32	TSLDIEEYsDTEVQK	1		
Q9H7L9	NLDPDPEPPsPDsPTETFAAPAEVR	7	S4(Phospho); S6(Phospho)	S(4): 99.0; S(6): 50.5; S(7): 50.5; T(14): 0.0; S(18): 0.0
Q9Y478	RNAEQYKDQADK	1	C1(Carbamidomethyl); S10(Phospho); C22(Carbamidomethyl)	S(2): 0.0; S(5): 0.0; S(8): 0.7; S(9): 16.1; S(10): 82.4; Y(15): 0.7; Y(20): 0.0
Q7Z417	ESEDKPEIEDVGsDEEEEKKDGDK	2	S3(Phospho)	S(3): 100.0; S(6): 0.0
Q9P0K7	HNsTTSSTSSGGYR	2	S1(Phospho)	S(1): 100.0; T(6): 0.0; Y(9): 0.0
Q16623	SASsGAEGDVSSEREP	3	S5(Phospho)	T(1): 0.0; S(5): 100.0; T(14): 0.0
Q8IWW6	SPWASDFKDFQESsPQK	3	S5(Phospho); S7(Phospho)	S(5): 100.0; S(7): 100.0; S(12): 0.0; S(13): 0.0; S(14): 0.0; S(15): 0.0; T(16): 0.0
Q9Y2K6	AAVPSGASTGIYEALELR	4	S3(Phospho); C5(Carbamidomethyl); T7(Phospho)	S(1): 6.9; S(2): 6.9; S(3): 86.2; T(7): 100.0
Q3KQU3	VEMYsGsDDDDDFNKLPK	5	S12(Phospho); S16(Phospho)	S(2): 0.0; S(7): 0.0; S(12): 100.0; S(16): 0.0; S(20): 0.2; T(22): 99.8
Q32MZ4	DRNENEIGHSR	1		
P78559	LDIDsPPITAR	1	S5(Phospho)	S(5): 92.7; S(6): 7.3
P37802	SKETSSPGTDDVFTPAPSDsPSSQR	3		
O43823	DKEVsDDEAEEK	1	S3(Phospho); S8(Phospho)	S(3): 100.0; S(8): 100.0
Q05682	DEREDITHTYK	2	S3(Phospho)	S(3): 100.0
Q6PKG0	ENPPVEDssDEDDKRNQGNLYDK	1	S3(Phospho); S6(Phospho); C16(Carbamidomethyl)	T(1): 10.5; S(3): 89.5; S(6): 100.0
Q9H1E3	IADPEHDHTGFLtEYVAtR	3	S9(Phospho)	S(3): 0.0; S(5): 50.0; S(9): 50.0
Q7L8J4	STGHGGHcTNcQDNTDGAHcER	2	S7(Phospho)	S(3): 50.0; S(7): 50.0; T(15): 0.0
P51812	DITSDTSGDFR	1	S2(Phospho)	S(2): 100.0; T(6): 0.0; Y(9): 0.0
Q15628	NRNEQESAVHPR	2		
O94808	RDsLTGSSDLYKR	1	S4(Phospho); C7(Carbamidomethyl)	S(4): 91.2; S(5): 8.8
Q8TEW0	ESsPLYSPTFSDSTSAVK	4	S1(Phospho)	S(1): 100.0; T(10): 0.0
P35579	HSQYHVDGSLEKDR	3		
Q76N89	KESKEEEtSIDVAGKPNEVTK	7	S3(Phospho)	S(1): 7.0; T(2): 7.0; S(3): 86.0; T(5): 0.1; T(9): 0.0; S(10): 0.0; S(12): 0.0
Q13905	EIQNGNLHEsDSESVPR	2	S4(Phospho)	S(2): 8.6; S(4): 91.4; S(9): 0.0; S(10): 0.0
Q13557	SRPTSEGsDIESTEPQK	2	S8(Phospho)	S(8): 74.4; T(9): 10.9; S(11): 1.7; S(12): 1.7; T(14): 10.9; T(15): 0.3
P30533	HMDPPPAPVQDRsPsPR	6	S8(Phospho)	S(3): 0.0; Y(7): 0.5; S(8): 98.9; T(9): 0.5
P07900	DEGLcsANVTR	1	S13(Phospho)	S(2): 0.0; S(13): 100.0
P04792	TTsPPEVSGYSYEK	2		
Q96ST2	IHRAsDPGLPAEEPKEK	4	S4(Phospho); S6(Phospho)	S(4): 100.0; S(6): 100.0
Q8NBU5	SEDYVDIVQGNR	1	S10(Phospho)	Y(2): 2.3; S(5): 7.9; T(6): 29.9; S(7): 29.9; S(10): 29.9
Q9Y4E1	EMPQDLRsPARTPPsEEDSAEAER	1	S4(Phospho); S6(Phospho)	S(4): 100.0; S(6): 100.0
P16070	GDLGIEIPAEK	1	S1(Phospho)	S(1): 100.0
Q15678	TPsDDEEDNLFAPPK	1	C2(Carbamidomethyl); S8(Phospho)	T(3): 0.0; S(6): 9.3; S(8): 90.6
Q69YQ0	HDGKEVDEGAWETK	1	S3(Phospho); S4(Phospho); C14(Carbamidomethyl)	S(3): 98.5; S(4): 12.6; S(8): 88.9; S(11): 0.0; T(16): 0.0
Q7Z5L9	KDsSSVVEWTQAPK	3	S4(Phospho)	S(4): 100.0
P18887	QEAIPDLEDsPPVsDSEEQQESAR	1	T4(Phospho); T17(Phospho)	T(1): 0.0; T(4): 0.0; S(10): 50.0; S(11): 50.0; T(17): 100.0; T(21): 0.0
O94826	NQEQMKPLEEKQEEER	1		
P09104	RWsEDcRLEGGGGPAGGFEDEGEDK	1	S10(Phospho)	Y(4): 0.0; S(10): 100.0; T(12): 0.0; S(15): 0.0
O15234	KGGSYSQAASSDSAQGsDVSLTAcK	1	T11(Phospho)	S(1): 0.0; S(9): 10.3; S(10): 10.3; T(11): 79.3; T(22): 0.0
P19105	RAsISEPSDTDPEPR	3		
Q9NQT8	DLLHPSLEEEKKK	2		
Q9NSY0	ASQSRPNSsALETLGGEK	3	S6(Phospho); C18(Carbamidomethyl)	S(6): 86.7; S(10): 13.3; S(16): 0.0
Q3KQU3	NsLTGEEGQLAR	1	S10(Phospho); S24(Phospho)	S(8): 50.7; S(10): 50.7; S(24): 18.2; S(26): 63.1; S(27): 17.4
Q92609	GLDsGAETEEEKDTWEEKK	1	T2(Phospho)	T(2): 98.0; S(3): 0.9; S(4): 0.1; T(5): 0.9; S(8): 0.0; T(11): 0.0; S(14): 0.0; Y(15): 0.0
Q96AJ1	WLDEsDAEMELr	2	S8(Phospho)	S(2): 0.0; S(8): 100.0
P62857	DDSTKPKPEEEVKPVK	1	S3(Phospho); C7(Carbamidomethyl)	T(1): 8.8; S(3): 91.2; T(8): 0.0
P62070	GTEDELDKYSEALKDAQEK	1	C7(Carbamidomethyl); S10(Phospho)	S(10): 100.0; T(14): 0.0
Q9UEW8	GALQNIIPASTGAAK	1	S9(Phospho)	T(1): 0.0; S(9): 100.0; S(17): 0.0
Q9H0G5	DSIKLDDDSERK	1	S9(Phospho); S15(Phospho); S16(Phospho)	S(9): 99.9; S(15): 99.7; S(16): 99.7; T(24): 0.7

P49023	DLGLSESGEDVNAAILDEsGKK	1	S1(Phospho)	S(1): 50.0; S(2): 50.0
Q13185	QEPLGsDSEGVNcLAYDEAIMAQQDR	3	S3(Phospho); S5(Phospho)	S(3): 100.0; S(5): 92.2; S(7): 7.7; S(9): 0.1; S(12): 0.0
P49840	DGSNKsGAEEQGPIDGPSK	2	T1(Phospho)	T(1): 45.8; S(2): 45.8; S(3): 8.4; S(21): 0.0; S(23): 0.0; T(28): 0.0
P04083	HGsPEFcGILGER	1		
P42858	SKDHFGLEGDEESTMLEDSVsPKK	2	S15(Phospho)	S(11): 0.2; S(15): 99.8
P17812	LKGNKHDDGTQsDSENAGAHR	2	S7(Phospho); S9(Phospho)	T(2): 0.0; Y(3): 0.0; S(4): 0.0; S(7): 98.8; S(9): 10.6; S(10): 89.4; S(11): 1.1; S(14): 0.0; T(17): 0.0
P63010	SNsWVNTGGPK	1	C9(Carbamidomethyl)	
O60841	LNLGTDSDSsPQK	2	S9(Phospho)	S(3): 0.0; S(9): 100.0; S(17): 0.0
Q9H8M9	AsRRsDSASSEPVGIYQGFEK	1	S5(Phospho)	T(4): 0.3; S(5): 99.7
Q92903	SVGDGETVEFDVVEGEK	1	S15(Phospho)	T(11): 0.0; S(13): 1.9; T(14): 1.9; S(15): 96.2; T(19): 0.0
Q96T60	AVTKDEDEWKELEQK	1	T1(Phospho); T8(Phospho)	T(1): 5.4; S(4): 95.6; T(8): 98.9; T(12): 0.1; S(16): 0.0
Q14671	HQDGLPYIDDsPSSSPHLSSK	1	S3(Phospho)	S(3): 92.4; T(5): 7.6; S(7): 0.0; S(8): 0.0; Y(11): 0.0
Q15642	sQEMVHLVNK	1	S3(Phospho); S5(Phospho)	S(3): 100.0; S(5): 7.3; S(6): 92.7; T(9): 0.0; S(11): 0.0
O14545	TTRtPDTSTYcYETAEK	3	S9(Phospho)	S(3): 0.0; T(8): 0.5; S(9): 99.5
P05455	GESAEDKHEEGRDsEEGPR	2	S5(Phospho)	T(1): 0.3; S(5): 99.7; T(18): 0.0
P28482	VPAEDETQsIDsEDSFVPGR	1	T13(Phospho); T18(Phospho)	T(9): 0.0; T(13): 100.0; Y(15): 100.0; T(18): 0.0
O00264	sVENLPEcGITHEQR	2	S9(Phospho)	T(6): 0.0; Y(8): 10.6; S(9): 89.3; S(18): 0.0
Q8WVC0	sVDAYDSYWESR	1	S4(Phospho)	T(3): 7.4; S(4): 92.6; S(11): 0.0
Q15293	RDSsDDWEIPDGQITVGQR	7		
Q9UBF8	KHHEEEIVHHK	1	S4(Phospho); C11(Carbamidomethyl)	S(1): 5.8; T(2): 47.1; S(4): 47.1; T(14): 0.0
Q8NFQ8	DLEAHIDSANK	3		
P46821	HsPTEDEESAKAEADAYIR	5	S10(Phospho)	S(3): 0.0; S(10): 100.0
P24534	KSLDsDEsEDEEDDYQQKR	2	S10(Phospho)	S(10): 100.0; S(16): 0.0
P78559	DGGNPFAEPSELDNPFQDPAVIQHRPsR	3	S17(Phospho); S20(Phospho)	T(1): 0.0; T(4): 0.0; Y(9): 0.0; S(12): 0.6; T(15): 0.6; S(17): 98.7; T(19): 96.7; S(20): 3.2; S(21): 0.1
Q9NYA4	VHNDAQsFDYDHDAFLGAEEAK	4	S3(Phospho); C11(Carbamidomethyl)	T(1): 6.4; S(2): 6.4; S(3): 87.2
P46108	NQVEEDAEDsGEADDEEKPEIHKPGQNSFSK	1	S2(Phospho)	S(2): 13.2; S(3): 2.4; T(4): 2.4; S(5): 2.4; Y(9): 79.4; S(12): 0.1; S(14): 0.0; S(17): 0.0
Q7KZF4	VEIIANDQGNR	2		
O43493	QsPDHPTVGAGVLHITENGPTEVDYSPSDMQDSSLSHk	3	S6(Phospho)	S(2): 0.3; S(4): 48.1; S(6): 48.1; S(7): 3.6; T(12): 0.0; T(16): 0.0
Q9BQE9	RHsTEGPEDPPPWAEK	3	S10(Phospho); S14(Phospho)	S(3): 0.0; S(4): 0.0; T(5): 0.0; S(7): 0.5; S(8): 2.8; S(10): 96.1; S(14): 0.5; S(16): 0.1; S(18): 0.1; T(22): 2.8; S(23): 97.1
P08238	RGGGDEESGEHTQVPADSPDSQEEQKGESSASSPEEPEEITcLEK	1	S6(Phospho)	S(6): 100.0; S(12): 0.0
P78559	VGHMSsVELDDELLDPDMDPPHPFPK	2	S4(Phospho); T8(Phospho)	S(4): 100.0; T(8): 100.0; T(14): 0.0
Q02952	TKEDEKDDKPIR	3	S4(Phospho); S8(Phospho); C12(Carbamidomethyl); C20(Carbamidomethyl)	S(3): 23.7; S(4): 81.3; S(8): 95.0; T(21): 0.0
Q5VT06	SATLSsTESTASEMQEEMK	2	S7(Phospho)	S(7): 100.0; S(12): 0.0
Q7Z5L9	KPsPEPEGEVGPPK	1	S3(Phospho)	S(3): 100.0
Q7Z699	SRsSDIVSSVR	1	S3(Phospho)	S(3): 100.0
Q07866	RDsSESQLASTESDKPTTGR	1	S5(Phospho)	S(3): 0.0; S(5): 99.4; T(7): 0.6
P46821	DDKHGsYEDAVHSGALND	1	S5(Phospho); S7(Phospho)	S(1): 0.1; S(3): 0.1; S(5): 99.8; S(7): 0.1; S(10): 100.0
P46821	DRGHDSEMIGDLQAR	2	C4(Carbamidomethyl); S8(Phospho); S9(Phospho)	T(1): 6.5; T(3): 46.6; S(6): 46.6; S(7): 7.2; S(8): 46.6; S(9): 46.6; S(16): 0.0; Y(20): 0.0
P08670	DFQYNEEEMKADKEEMNR	1		
Q03135	sDSRAQAVSEDAGGNEGR	2	S7(Phospho)	S(7): 100.0; Y(12): 0.0; T(16): 0.0
Q16799	AEtEEAEEPEEDGEEHVcVSASK	1	S4(Phospho)	S(2): 0.1; S(4): 99.9; T(10): 0.0
Q86XR7	QNsATESADSIEIYVPEAQTR	4	S3(Phospho)	S(3): 90.7; T(6): 8.4; S(7): 0.8; Y(10): 0.0; S(13): 0.0; S(15): 0.0
O43719	NFEDVAFDEKK	2	S6(Phospho)	S(6): 100.0; Y(14): 0.0
Q9NRY4	KNDKEAAGEGPALYEDPPDQK	2	S5(Phospho)	S(5): 99.5; S(7): 0.5
Q71U36	EGMNPSYDEYADsDEDQHDAYLER	1		
Q9UGP4	AKAEASSGDHPTDTEMKEEQK	1	C6(Carbamidomethyl); C9(Carbamidomethyl); C12(Carbamidomethyl)	
Q13459	GSLAsLDSLR	1	S8(Phospho); T12(Phospho)	S(2): 0.0; S(8): 100.0; T(12): 100.0
Q92466	LDSQPQETsPELPR	2	S3(Phospho)	S(1): 7.5; S(3): 92.5
Q96EY5	NSASATTPLsGNSSR	1	S6(Phospho)	S(6): 99.8; S(11): 0.2; S(14): 0.0
Q96ST2	cAsLEEILSQR	1	S4(Phospho); S6(Phospho)	T(1): 2.7; S(4): 98.6; S(6): 98.6; S(15): 0.0
Q53EL6	LKFsDDEEEEEVVKDGRPK	1	S7(Phospho)	S(2): 0.0; S(7): 90.3; S(9): 9.7; S(11): 0.0; S(13): 0.0
Q14669	RPGTQGHLGPEKEEEEsDGEPEDSSTS	3	S7(Phospho)	S(3): 0.0; S(7): 100.0
Q5T5C0	RGsIGENQGEEK	1	S3(Phospho); S6(Phospho)	S(1): 0.6; S(2): 0.6; S(3): 98.8; T(5): 6.8; S(6): 93.2; S(11): 0.0
Q7L2J0	DLIHTGVANDHEEDFELRK	2	T1(Phospho); S10(Phospho)	T(1): 0.0; T(6): 99.2; S(9): 9.0; S(10): 91.8
Q9Y2X7	HQEQHHPELEDKDLDFK	4	S3(Phospho); S7(Phospho)	S(3): 100.0; S(7): 92.5; Y(9): 3.4; T(12): 3.4; S(14): 0.7
Q4G0J3	TEMDKSPFNsPsPQDSPR	1	S5(Phospho); T6(Phospho)	S(5): 100.0; T(6): 100.0; T(12): 0.0
P43487	KEKPELSEPSHLNGPSsDPEAAFLSR	2	T13(Phospho)	T(2): 1.1; T(8): 1.1; S(9): 1.1; T(10): 4.6; T(13): 91.7; S(16): 0.3; S(27): 0.0
Q9H1E3	GAVAAEGASDtEREEPTESQGLAAR	2	S10(Phospho); S21(Phospho)	Y(4): 14.4; S(5): 14.4; S(10): 71.3; Y(17): 82.5; S(21): 16.7; T(25): 0.8
Q04637	SIDDEITEAKSGtATPQR	3		
P78559	EGEEPTVYsDEEEPKDESARK	4	T11(Phospho)	S(6): 0.0; S(7): 0.0; T(11): 100.0
Q96B23	KRsSSEDAESLAPR	3	S3(Phospho); S5(Phospho)	S(1): 15.1; S(3): 81.9; S(5): 9.5; T(7): 42.9; S(8): 42.9; T(9): 7.7
Q96QB1	DRVHHEPQLSDK	7	S3(Phospho); C12(Carbamidomethyl)	S(3): 50.0; S(6): 50.0; S(15): 0.0
Q12888	DIISDTSGDFR	1	S2(Phospho); C15(Carbamidomethyl)	S(2): 100.0; S(9): 0.0; T(11): 0.0; S(14): 0.0
Q86TC9	NSRPEANEALER	2	S6(Phospho)	T(1): 0.0; S(6): 100.0; T(17): 0.0
Q96EI5	SRSLsNSNPDISGTPTSPDDEVR	2	S12(Phospho)	S(12): 33.3; S(18): 33.3; T(20): 33.3
P35611	APEsDTGDEDQDQERDTVFLK	1	S4(Phospho)	S(1): 0.1; S(4): 99.9; T(10): 0.0; S(12): 0.0
P02786	TSFsVGsDDELGPIR	5		
P52926	GAsPDMAPILEPVDR	2	S11(Phospho)	T(7): 0.0; S(11): 100.0
P23634	KEsAPQVLLPEEEKIIVEETK	2		
Q5TCZ1	FARsDDEQSSADKER	1	S7(Phospho)	S(2): 9.6; S(4): 9.6; S(7): 80.8; T(13): 0.0; S(15): 0.0; S(19): 0.0; S(22): 0.0
Q13367	RGSLsNAGDPEIVK	3	S5(Phospho)	Y(3): 50.0; S(5): 50.0; S(15): 0.0; T(18): 0.0; S(25): 0.0
Q5SW79	RADNcsPVAEEETTGSAESTLPK	2		
O14980	DMTSEQLDDILK	1		
P30622	AQsPGAVEEILDR	1		
Q86TB9	DSHssEEDEASSQTDLSQTISK	1	S3(Phospho); S8(Phospho)	S(1): 1.7; T(2): 11.3; S(3): 87.0; S(8): 100.0
Q9Y2X3	EGEEPTVYsDEEEPKDESAR	3	C13(Carbamidomethyl); S15(Phospho)	S(8): 99.6; T(14): 0.3; S(15): 0.1; T(16): 0.0; S(20): 0.0
P46940	DSDYLKNDNPEEHLK	1	S3(Phospho)	S(1): 0.7; S(3): 99.3; S(8): 0.0; T(11): 0.0
P08651	DSLLQDGEFSMDLr	1	S6(Phospho)	T(1): 1.2; S(6): 98.8; S(10): 0.0; S(12): 0.0; S(16): 0.0
Q92934	SKSDsYTLDPDTLR	3	Y4(Phospho)	S(2): 9.7; S(3): 9.7; Y(4): 70.9; T(8): 9.7; S(19): 0.0
Q9H832	NSPTFKsFEEK	1	S10(Phospho); S12(Phospho)	S(10): 99.4; S(12): 97.1; S(13): 3.3; S(14): 0.1; S(15): 0.0; T(17): 0.0; T(19): 0.0; S(24): 0.0
Q13428	NQTAEKEEFEHQQKELEK	3	S12(Phospho)	S(10): 85.5; S(12): 13.8; T(16): 0.5; S(17): 0.0; T(18): 0.1; T(19): 0.1; S(20): 0.1
Q9NYF3	TGsQGQcTQVR	1	S3(Phospho); C6(Carbamidomethyl)	S(1): 7.0; S(3): 93.0
Q8NE71	sAEALGPGALVsPR	1	S6(Phospho)	S(2): 0.0; T(5): 0.8; S(6): 99.2
Q92609	EGsEFsFSDGEVAEK	2	S6(Phospho)	S(5): 1.6; S(6): 86.9; S(10): 11.3; S(12): 0.2
O43150	DQHSFELDEK	1	S9(Phospho)	S(9): 100.0; S(20): 0.0
P41236	DGDSYDPYDFsDTEEEMPQVHTPK	2	S6(Phospho); S7(Phospho)	S(6): 100.0; S(7): 100.0; S(12): 0.0; S(15): 0.0
P30622	VAAAAGSGPsPPGsPGHDRER	4	S10(Phospho); S14(Phospho)	T(1): 0.0; S(3): 0.0; S(5): 0.2; S(7): 3.6; S(10): 96.2; S(14): 99.9
Q86TB9	LDTGPQSLsGKsTPQPPSGK	4	T3(Phospho); S9(Phospho)	S(2): 1.3; T(3): 50.0; S(4): 50.0; S(9): 98.7

P12694	cDSSPDsAEDVR	1	S5(Phospho)	S(5): 48.6; T(6): 48.6; S(7): 2.9; S(10): 0.0; S(11): 0.0; Y(13): 0.0
P11274	GyTSDSEVYTDHGRPGK	5	S11(Phospho)	Y(7): 0.1; S(11): 11.5; S(13): 2.8; S(14): 11.5; S(15): 51.1; S(19): 11.5; S(20): 11.5
P05023	DIDISsPEFK	2		
Q9H0G5	HLDGEEDGsSDQSQASGTTGGR	2	S15(Phospho); S16(Phospho)	S(9): 24.4; S(15): 87.8; S(16): 87.8; T(24): 0.0
P50548	VSQsPSKDSEENPATEERPEK	3	S2(Phospho); C6(Carbamidomethyl)	S(2): 44.2; S(4): 44.2; S(7): 9.1; T(10): 0.5; S(11): 2.0
Q8IYB3	DAGTIAGLNVLR	1	S3(Phospho); S5(Phospho); S7(Phospho)	S(3): 100.0; S(5): 100.0; S(7): 100.0
O95292	DPDAQPGGELMLGGTDSK	1	S5(Phospho)	S(1): 0.0; S(3): 0.0; S(4): 7.6; S(5): 92.4; T(9): 0.0
P16383	IDASKNEEDEGHSNsSPR	6	S3(Phospho); S9(Phospho)	S(3): 100.0; S(9): 100.0
Q96D46	cDGDHDcADGSDEKDcTPR	1	T10(Phospho)	S(2): 0.0; S(8): 0.0; T(10): 100.0
Q9Y2W2	QDVDLcLVsScEYK	2	S9(Phospho)	Y(8): 0.5; S(9): 99.5
P35606	VADPDHDHTGFLtEYVAtR	6		
O94885	GGDAKsPVLQEK	1	S4(Phospho)	S(4): 98.9; S(7): 1.1
Q9NZT2	DLGSTEDGDGtDDFLTDKEDEK	1	S18(Phospho)	S(1): 0.0; S(18): 100.0
Q9BY89	DSDKTDTDWR	1	S4(Phospho)	S(4): 100.0; T(9): 0.0; S(11): 0.0
P07900	GNAEGSsDEEGKLVIDEPAKEK	2	S13(Phospho)	S(2): 0.0; S(13): 100.0
P49411	VHVGDEDFVHLR	1	C5(Carbamidomethyl)	
Q3KQU3	VGVTEGcDPTR	1	S7(Phospho); S11(Phospho); S15(Phospho)	S(2): 0.0; S(7): 100.0; S(11): 100.0; S(15): 99.9; T(17): 0.1
P46821	NDsPTQIPVSSDVcR	1	T3(Phospho); C4(Carbamidomethyl)	T(1): 9.7; T(3): 85.6; S(6): 1.2; S(7): 1.2; S(8): 1.2; S(9): 1.2; S(16): 0.0; Y(20): 0.0
P62158	GDDGIFDDNFIEER	1		
P46821	NSNPALNDNLEK	1	S13(Phospho); S17(Phospho)	S(10): 0.0; S(13): 88.0; T(14): 20.0; T(15): 12.0; S(17): 80.0
P35221	HQEGEIFDTEKEK	1	S4(Phospho)	S(1): 0.4; T(3): 49.6; S(4): 49.6; T(7): 0.4; S(17): 0.0
Q9Y5Q9	SEAPAEVTHFsPK	1	S4(Phospho)	S(4): 100.0; S(12): 0.0; S(16): 0.0; S(17): 0.0; S(18): 0.0; S(22): 0.0; T(23): 0.0
Q9H6Z4	EVQDKDYPLtPPPSPtVDEPK	2	S13(Phospho); C21(Carbamidomethyl)	S(1): 0.0; S(5): 0.0; S(6): 0.0; S(13): 96.0; Y(20): 4.0
Q13428	QSFDDNDsEELEDKDSK	2	S3(Phospho)	S(3): 100.0
O00461	TTWGDGGENsPcNVVSK	1		
P34932	GALSSsLRDLSDAGVcY	3	C-Term(Oxidation)	
Q15052	EAQTLDSQIQETsI	2	S2(Phospho)	S(2): 50.0; T(3): 50.0
Q07157	DDTDDEIAKYDGKWEVEEMK	1	S14(Phospho); S20(Phospho)	S(6): 0.0; S(14): 100.0; S(20): 83.0; Y(21): 17.0
Q9HCH0	KAAsPSPQSVR	1	S4(Phospho)	S(2): 48.0; S(4): 48.0; S(7): 2.0; S(9): 2.0
Q00613	NVFTsAEELER	2	S11(Phospho); S16(Phospho)	T(3): 0.0; T(5): 0.2; S(11): 99.8; T(15): 0.7; S(16): 49.7; T(17): 49.7
O00499	SKsVKEDSNLTLQEK	1	S3(Phospho)	S(1): 0.9; S(3): 99.1; S(8): 0.0; T(12): 0.0
P23284	RMsDEFVDSFK	1	C12(Carbamidomethyl)	
Q05682	HGARtSDSDQQAYLVQR	2	S3(Phospho)	S(3): 100.0
Q9UPN3	STsPIIGsPPVR	2	S3(Phospho)	S(3): 99.5; S(5): 0.5; S(10): 0.0
P50552	ANEAGGQVGPEAPRPPEtSPEMR	2	S3(Phospho)	S(3): 99.8; S(9): 0.2; T(13): 0.0
O14617	yHGHSMSDPGVSYR	3	S5(Phospho)	T(2): 0.0; T(4): 0.7; S(5): 49.7; S(7): 49.7
P55327	EMEKsFDEQNVPK	2	S7(Phospho)	S(2): 0.0; T(4): 92.5; S(7): 7.5
P51580	GLSEDTTEETLKESFDGSVR	2	S9(Phospho)	T(1): 0.0; S(2): 0.0; Y(8): 1.2; S(9): 88.7; T(11): 10.1
Q02952	LLKPGEEPSEYtDEEDTKDHNKQD	4	S17(Phospho)	S(17): 100.0; S(25): 0.0
O14974	AFLAELEQNsPK	1	S11(Phospho); S20(Phospho)	S(1): 1.4; T(2): 1.4; S(5): 0.4; T(8): 19.2; S(11): 77.5; S(20): 98.1; T(22): 1.9; S(26): 0.0
Q8IU81	LHDNPLTDENKEHEADTANMSDk	1	S3(Phospho)	S(3): 100.0
Q7Z5R6	HTGPNsPDTANDGFVR	1	S3(Phospho)	S(2): 0.3; S(3): 99.7; T(5): 0.0; S(6): 0.0; S(8): 0.0; T(11): 0.0
P60660	DLEDKEGEIQAGAK	2		
Q9NYL9	SPGsPVGEGTGSPPK	1	S14(Phospho)	Y(1): 0.0; S(14): 100.0; T(16): 0.0
Q9H4A3	ESEDKPEIEDVGsDEEEEK	3	S11(Phospho)	S(6): 0.0; S(8): 1.1; S(11): 98.9; S(16): 0.0; S(17): 0.0
Q9Y608	SQsSHSYDDSTLPLIDR	2	S3(Phospho)	S(3): 45.0; T(6): 45.0; S(7): 5.0; S(8): 5.0; T(14): 0.0; S(15): 0.0; S(17): 0.0
Q8N350	QLsSGVSEIR	2	S3(Phospho)	S(3): 100.0; S(8): 0.0; Y(10): 0.0
Q07866	GADsGEEKEEGINR	1	S4(Phospho)	S(1): 95.2; S(4): 4.8
P78559	DSPSKsSAEAQTPEDTPNK	8	S10(Phospho); S13(Phospho)	T(2): 0.0; S(3): 0.0; S(9): 0.2; S(10): 99.8; S(13): 100.0
Q96I99	DSVLsYTSVR	1		
Q4G0J3	TRHsPTPQQSNR	4	S3(Phospho)	S(3): 33.3; S(4): 33.3; S(5): 33.3; S(10): 0.0
Q02952	IYVDDGLISLQVK	1	S17(Phospho)	T(16): 9.8; S(17): 90.2
P08581	NLsPGAVESDVR	1	S3(Phospho)	S(1): 49.9; S(3): 49.9; T(5): 0.1; T(6): 0.0; S(10): 0.0; S(13): 0.0; Y(16): 0.0
Q7Z3G6	DSPSVWAAVPGK	1	S3(Phospho)	S(1): 50.0; S(3): 50.0; S(11): 0.0
Q9ULQ0	KSAsPEVSEGHENQHGQESEAK	4	S3(Phospho)	S(3): 100.0; Y(7): 0.0
Q9Y696	SLAPDRsDDEHDPLDNTSRPR	2		
Q8WWI1	IEDVGsDEEDDSGKDKK	8	S4(Phospho)	S(4): 0.8; S(7): 99.2; S(10): 0.0; S(13): 0.0
O95425	QEKPAEKPAETPVATSPTATDSTSGDSSR	1	S10(Phospho)	S(2): 0.0; S(3): 0.0; T(5): 0.0; S(10): 100.0
Q99584	QKSDAEEDGGTVsQEEEDRKPK	8	S3(Phospho)	S(3): 99.2; S(5): 0.8
P46821	ERsPsPLRGNVVPsPLPTR	3	T3(Phospho); C18(Carbamidomethyl)	T(3): 100.0; S(20): 0.0; S(22): 0.0
Q9HC35	HQEHKGEIDAHEDSFK	5	T14(Phospho); T16(Phospho)	S(4): 0.0; S(5): 0.0; T(6): 0.0; S(8): 0.0; S(12): 19.2; T(14): 80.6; T(16): 93.7; S(20): 5.8; T(26): 0.4; S(31): 0.1
Q4G0J3	SSsQENLLDEVMK	3	S8(Phospho)	S(1): 0.0; T(4): 0.0; S(5): 0.1; S(8): 99.8; S(12): 0.0; T(13): 0.0
Q9Y520	DLFAFQEsPPR	2	S5(Phospho)	S(5): 100.0
P46821	QQNQEITDQLEEEKKEcHQK	1	S6(Phospho)	T(1): 0.0; S(6): 97.1; S(8): 2.4; S(10): 0.4; T(12): 0.0; S(14): 0.1; T(18): 0.0; Y(20): 0.0; Y(21): 0.0; S(23): 0.0; T(25): 0.0
P51858	HRPsPPAtPPPK	6	S7(Phospho); S8(Phospho)	S(7): 100.0; S(8): 100.0
Q9BQE3	MGAPESGLAEYLFDKHTLGDSDNEs	3	S9(Phospho)	Y(2): 0.0; S(9): 100.0; Y(19): 0.0
Q8WWI1	tATcHSSSSPPIDAASAEPYGFR	4	S3(Phospho)	S(3): 99.7; S(6): 0.3; S(9): 0.0; S(12): 0.0
P53985	GSEDsPPKHAGNNESHSSR	4	S9(Phospho)	S(3): 0.0; T(8): 50.0; S(9): 50.0; T(20): 0.0
Q58WW2	DKLGERDDTIPPEYR	1	T7(Phospho); S10(Phospho)	S(2): 92.1; T(7): 15.8; S(10): 92.1
O75832	AGDNIPEEQPVASTPTTVsDGENKKDK	3		
P0CG48	RPMEEDGEEKsPSK	3		
Q5VZK9	SSsMSSIDLVSASDDVHR	6	S4(Phospho)	S(1): 50.0; S(4): 50.0
P07814	RREEGPPPPSPDGAsSDAEPEPPSGR	8	S15(Phospho); S16(Phospho)	Y(2): 0.0; S(10): 0.6; S(12): 92.0; S(13): 7.3; S(15): 0.7; S(16): 98.6; T(18): 0.7
Q96EV2	TATESFASDPILYRPVAVALDTK	1	S6(Phospho)	S(6): 100.0; S(16): 0.0
Q9Y4H2	KDNEESEQPPVPGtPTLR	2	S3(Phospho)	S(3): 100.0
Q04726	MLEEKsPEKVK	2	S3(Phospho); S8(Phospho)	Y(1): 66.7; S(3): 66.7; S(8): 66.7; S(16): 0.0; T(22): 0.0
Q86VP6	KIAELEEEQSQGSTTNsDWMk	3		
P10644	KPSGsPDLWKLsPDQR	2	S3(Phospho); S9(Phospho)	T(1): 50.0; S(3): 50.0; S(9): 100.0
P10451	NAGVEGSLIVEK	1	S8(Phospho); S14(Phospho)	S(4): 91.8; Y(5): 8.2; T(7): 0.0; S(8): 0.0; S(14): 100.0; T(17): 0.0; S(19): 0.0
O00193	DGHAtDEEKLASTScGQK	1	S4(Phospho)	S(2): 49.7; S(4): 49.7; S(11): 0.3; S(12): 0.3
Q9UPP1	SKGHYEVTGsDDETGK	1	S11(Phospho)	Y(4): 0.0; Y(6): 0.4; S(8): 13.4; S(11): 86.2
Q9H1B7	DKQQAEENEKNLEDQSSK	1	S3(Phospho)	S(3): 100.0; S(9): 0.0
P63151	ScSIDRsPGAGsLGSPASQR	1		
P78559	FLEDDTsDPTYTSALGGK	3	C1(Carbamidomethyl); S3(Phospho)	S(3): 100.0; S(7): 0.0; T(8): 0.0
Q92609	SDIsPLTPR	2	S2(Phospho)	T(1): 48.1; S(2): 48.1; S(3): 3.6; T(4): 0.3; S(7): 0.0; T(10): 0.0; S(13): 0.0; Y(14): 0.0
Q8IZP0	sTELVLSPDMPR	2	S3(Phospho)	S(3): 87.1; T(4): 11.0; T(5): 1.5; S(6): 0.2; S(7): 0.0; T(8): 0.0; S(9): 0.0; S(10): 0.0; Y(13): 0.0
Q86YP4	NQsPVLEPVGR	1	S4(Phospho); S11(Phospho); S17(Phospho)	S(4): 100.0; S(11): 100.0; S(17): 8.9; S(18): 91.1
P60709	TQtPPVSPAPQPtEERLPSSPVYEDAASFK	4		

Q9ULU8	RPEGPGAQAPSsPR	5	S8(Phospho)	S(7): 13.9; S(8): 72.0; S(10): 13.9; S(12): 0.2; S(15): 0.0
Q9UQ35	AQGEPVAGHEsPKIPYEK	3	S10(Phospho); S12(Phospho)	T(1): 30.4; T(3): 19.1; T(4): 19.1; S(10): 62.2; S(12): 60.9; S(15): 8.3
Q9H1E3	AGQAVDDFIEK	1	S5(Phospho); S9(Phospho)	S(3): 0.2; S(5): 99.8; S(9): 100.0
Q8NE71	AEDGsVIDYELIDQDAR	1	T6(Phospho); S7(Phospho)	S(3): 0.0; T(6): 100.0; S(7): 100.0
O43896	RSPtSsPTPQR	2	S5(Phospho); S7(Phospho)	Y(2): 0.0; S(5): 99.9; S(7): 92.4; S(11): 7.6
Q13263	DKDDKISWEEYK	1	S1(Phospho)	S(1): 100.0; S(7): 0.0
P07900	LEGDsDVDSELEDRVDGVK	1	S13(Phospho)	S(2): 0.0; S(13): 100.0
O60841	VAGEAAEtDsEPEPEPEPTAAPR	2	S8(Phospho)	S(2): 0.0; S(8): 100.0; S(16): 0.0
Q9NRY4	KHGGPKDEER	5	T1(Phospho); S7(Phospho)	T(1): 52.5; S(2): 52.5; S(4): 82.5; S(7): 12.6
P24386	GNsRPGTPsAEGGSTSSTLR	3	S11(Phospho)	S(2): 0.0; T(3): 0.0; S(11): 99.1; S(12): 0.9
P15531	DcDVQGLEHDMEEINAR	1		
P55287	DKPLKDVIIADcGK	1	S7(Phospho)	Y(5): 0.1; S(7): 99.9; T(10): 0.0; S(15): 0.0
Q9UDY2	VLsPLRsPPLIGSESAYESFLSADDK	1	S3(Phospho)	S(3): 100.0; Y(8): 0.0
O60238	LNtSDFQK	1	S4(Phospho)	S(3): 33.3; S(4): 33.3; S(6): 33.3
P27824	KDNDFIYHDR	1	S11(Phospho)	S(1): 0.0; T(9): 1.3; S(11): 98.7
Q8IYB3	SKSEEAHAEDSVMDHHFR	3	S4(Phospho); S6(Phospho)	S(4): 100.0; S(6): 99.9; S(9): 0.1
O60271	AEGEWEDQEALDYFsDKESGK	1	S3(Phospho); S5(Phospho)	S(1): 8.9; S(3): 83.2; S(5): 16.8; S(6): 91.1
P33176	AKtQTPPVsPAPQPTEER	4		
O94967	DKSDEKPSYcNSR	2	T7(Phospho)	S(6): 50.0; T(7): 50.0; Y(13): 0.0; Y(14): 0.0
P00338	TYsDEANQMR	1		
Q9UPN6	NTSAEKELSSPIsPK	4	T16(Phospho)	S(1): 0.0; S(2): 0.0; T(8): 0.0; T(11): 0.0; T(12): 0.0; S(14): 10.2; T(16): 89.8
Q01082	AGKEPAKPsPSR	2	S6(Phospho); S7(Phospho); S11(Phospho)	T(1): 33.3; S(2): 33.3; S(3): 33.3; S(6): 8.8; S(7): 91.2; S(11): 100.0; T(13): 0.0; S(14): 0.0
Q16555	AMADELsEKQVYDAHTK	1	S7(Phospho); S11(Phospho)	T(1): 0.0; T(3): 0.0; S(6): 8.1; S(7): 8.1; T(10): 83.9; S(11): 100.0
P14618	EQFLDGDGWTSR	1		
Q09666	SRSPGsPVGEGTGSPPK	1	S6(Phospho)	S(5): 0.2; S(6): 99.8
Q8TC07	VQGGVPAGsDEYEDEcPHLIALSSLNR	1	S4(Phospho)	S(3): 33.3; S(4): 33.3; S(5): 33.3; T(10): 0.0
P25205	DSSFTEVPRsPK	1	S5(Phospho)	S(1): 0.0; S(5): 100.0; T(7): 0.0; S(14): 0.0
Q09666	KDEGEGAAGAGDHKDPsLGAGEAASK	2	S5(Phospho); S15(Phospho)	S(2): 0.4; S(5): 99.6; S(15): 50.0; S(16): 50.0
P23396	LHDNPLTDENKEHEADTANMSDK	2	T7(Phospho)	T(6): 6.7; T(7): 93.3; S(10): 0.0
Q09666	KHEEEEAKAER	5	S5(Phospho)	S(5): 100.0; T(11): 0.0; T(13): 0.0
Q8TAQ2	RLDsSAcLHAVGDK	5	S3(Phospho); S5(Phospho)	S(3): 99.4; S(5): 47.9; S(7): 47.9; T(9): 4.7
P60709	KLNVTEQEKIDK	1		
Q8NE71	KNsLKEDHEETK	3	T5(Phospho); S6(Phospho)	S(2): 0.0; T(5): 100.0; S(6): 100.0
P23588	ATAPQTQHVsPMR	2	S5(Phospho)	Y(1): 10.7; S(5): 89.3; Y(17): 0.0
P51858	KKPNEDEVNQDSVKK	1	S8(Phospho)	S(8): 100.0
Q86X27	KLGAGEGGEASVsPEKTSTTSK	1	S1(Phospho)	S(1): 50.0; S(4): 50.0; S(16): 0.0; S(19): 0.0
Q9UK76	DSVFLScSEDNR	2	S4(Phospho); S7(Phospho)	S(3): 90.1; S(4): 10.5; S(7): 49.7; S(8): 49.7
Q96D71	KQQQEPTGEPsPK	3	S3(Phospho)	S(3): 98.7; S(4): 0.6; S(5): 0.6; Y(6): 0.1
Q5TAQ9	QRSDDESPStSSGSSDADQR	2	S5(Phospho)	S(5): 100.0
P50991	sVDFDSLTVR	1	C11(Carbamidomethyl)	
Q96ST2	SDGsLEDGDDVHR	1	S3(Phospho)	S(3): 100.0
Q9BYX2	NDsGEENVPLDLTR	1	C7(Carbamidomethyl); S9(Phospho)	S(4): 0.0; S(9): 100.0
Q9H4L5	TNSsDSERSPDLGHSTQIPR	4	S3(Phospho)	S(3): 50.0; S(4): 50.0; S(9): 0.0; T(10): 0.0
Q92597	AGDLLEDsPKRPK	4	S6(Phospho)	S(1): 1.8; S(3): 11.4; T(5): 11.4; S(6): 75.2; T(14): 0.1; S(17): 0.0; S(23): 0.0
P47712	RTsSTLDSEGTFNSYR	4	S10(Phospho)	S(4): 0.0; S(7): 0.0; S(8): 0.0; S(10): 100.0; S(14): 0.0
Q9P270	RSsDTSGSPATPLK	5	S6(Phospho); S10(Phospho); S14(Phospho)	S(2): 0.0; S(6): 100.0; S(10): 100.0; S(14): 100.0
Q92934	ALVVPEPEPDSDsNQER	2	S3(Phospho)	S(3): 100.0; S(9): 0.0
P07900	RMsDEFVDSFKK	1		
P40222	AELEEMEEVHPsDEEEEDATK	4	S12(Phospho)	S(11): 8.2; S(12): 91.8
Q9UQN3	RADsDRIQPSADR	1	S4(Phospho)	T(2): 0.0; S(4): 100.0
Q5TH69	KPEDWDERPK	1	S3(Phospho); S6(Phospho)	S(1): 49.7; S(3): 49.7; T(4): 0.6; S(6): 1.0; S(7): 98.9; S(9): 0.1; S(11): 0.0
Q9H1H9	AAVLsDsEDEEKASAK	1	S3(Phospho); C4(Carbamidomethyl); C10(Carbamidomethyl)	T(1): 49.6; S(3): 49.6; S(5): 0.9; S(12): 0.0
Q9H4G0	NSTGGTSVGWDsPPAsPLQR	2	S14(Phospho)	S(1): 40.7; S(8): 4.9; S(12): 13.6; S(14): 40.7; S(32): 0.0
O75976	RPMEEDGEEKsPSKK	2	T11(Phospho); T13(Phospho)	S(1): 94.6; S(4): 96.8; T(11): 5.8; T(13): 2.8; T(17): 0.0; Y(19): 0.0; S(20): 0.0; S(21): 0.0
O00461	KTAHNSEADLEESFNEHELEPsSPK	2		
Q13442	VFDDEsDEKEDEEYADEK	2	S5(Phospho); S8(Phospho)	S(2): 0.6; S(5): 99.4; S(8): 100.0; Y(15): 0.0
O94929	ETVSEAPPLLFsDEEEKEAQLGVK	1	S3(Phospho)	S(3): 92.1; S(4): 7.9
P17987	NHDEESLEcLcR	1	S6(Phospho)	S(6): 98.8; Y(7): 1.2; S(13): 0.0
Q13435	ASVsPMDEPVPDsESPIEK	3	S6(Phospho); S8(Phospho)	S(1): 14.2; S(2): 14.2; S(6): 72.5; S(8): 83.0; T(10): 16.0; T(14): 0.1; S(16): 0.0; S(18): 0.0
O00264	TRsNPEGAEDR	1	S9(Phospho)	T(6): 0.0; Y(8): 10.0; S(9): 90.0; S(18): 0.0
P49736	IGHHsTSDDSSAYR	2	S8(Phospho)	T(6): 0.4; S(7): 0.0; S(8): 99.6
Q8TEU7	KGDRsPEPGQTWTR	4	S8(Phospho)	S(8): 100.0; T(21): 0.0
O43399	LEGDSDDLLEDsDSEEHSR	3	N-Term(Acetyl); S12(Phospho)	S(3): 0.0; S(12): 100.0
P21980	EKAEGDVAALNR	1		
Q9H694	SFLGTsGELSVK	2	S3(Phospho)	S(1): 9.0; S(3): 91.0; S(11): 0.0; S(13): 0.0
Q53LP3	RtPSDDEEDNLFAPPK	4	S7(Phospho)	S(6): 5.1; S(7): 94.9
Q9NRY5	RMsADMSEIEAR	1		
Q9BVG4	cSDVSELSSsPPGPYHQEPYVcKPEER	2	S4(Phospho)	S(4): 100.0
Q6PD62	NSATFKsFEDR	4	T13(Phospho)	T(13): 100.0; S(20): 0.0
P35611	QGsFSEDVISHK	1	S6(Phospho)	S(1): 0.0; S(3): 0.0; S(6): 100.0; T(12): 0.0; S(14): 0.0
Q16555	GHREEEQEDLTK	1	T1(Phospho); S7(Phospho); S11(Phospho)	T(1): 8.8; T(3): 91.2; S(6): 8.2; S(7): 83.9; T(10): 8.2; S(11): 99.8
P13639	cNEIINWLDKNQTAEKEEFEHQQK	1	C11(Carbamidomethyl)	
P27824	IGGIGTVPVGR	1		
O60841	HEEAPGHRPTTNPNASK	1	S5(Phospho); S7(Phospho)	Y(4): 1.1; S(5): 99.0; S(7): 99.9
Q8NG27	VNGDDHHEEDMDMsD	1	T5(Phospho)	T(5): 100.0; S(14): 0.0; T(15): 0.0; S(16): 0.0
Q96ST2	RKsLsDSESDDSK	3	S3(Phospho)	S(3): 100.0
Q08999	QQPPEPEWIGDGESTsPSDK	1	C5(Carbamidomethyl); S9(Phospho); T12(Phospho)	S(9): 100.0; T(12): 100.0
P29966	HIAEEADRKYEEVAR	4	T15(Phospho)	S(13): 17.4; T(15): 78.4; S(23): 4.1; S(26): 0.0; S(27): 0.0; T(28): 0.0; S(29): 0.0; S(30): 0.0
Q9Y4E1	ETEEQDsDSAEQGDPAGEGKEVLcDFcLDDTRR	4	T4(Phospho); S6(Phospho)	T(4): 100.0; S(6): 100.0
P10451	SLDsDEsEDEEDDYQQKR	2	S9(Phospho)	S(2): 0.0; S(7): 0.0; S(9): 88.9; S(10): 11.0
Q9NYM9	mEVAEPSsPTEEEEEEEEHSAEPRPR	3	S3(Phospho)	S(3): 100.0
O94915	HGsVSADEAAR	1	T3(Phospho)	S(1): 7.5; T(3): 92.5
P54259	RHsVDTSPGYHESDSK	1	S4(Phospho)	S(4): 100.0; S(9): 0.0; T(10): 0.0; S(11): 0.0; S(12): 0.0; S(13): 0.0
Q8WX93	ASLGsLEGEAEAEAsSPKGK	2	S3(Phospho)	S(3): 100.0; T(10): 0.0
Q9UER7	SNSGRELtDEEILASVMIK	3	S5(Phospho)	S(5): 85.7; S(8): 12.4; S(9): 2.0; S(13): 0.0
O14617	DGDKPEETQGKAEKDEPGAWEETFK	1	S7(Phospho); C-Term(Oxidation)	T(2): 0.0; T(4): 0.0; S(5): 0.9; S(7): 99.1
P33527	sLYESFVSSSDR	2	S4(Phospho); S5(Phospho)	S(3): 98.9; S(4): 1.0; S(5): 0.2; S(6): 7.4; S(7): 7.4; Y(8): 77.6; S(9): 7.4; S(13): 0.0
Q9H1E3	LSsSKENLDASK	1	S8(Phospho)	S(8): 100.0

Q8NE71	GELsPSFLNPPLPPSIDDRDLSTEEVR	4	S16(Phospho)	S(14): 1.6; T(15): 1.6; S(16): 95.3; S(18): 1.6
Q96ST2	DLDKELDEYMHGGK	1	S4(Phospho); S6(Phospho)	S(4): 100.0; S(6): 100.0
Q86W56	DKDRNWDDIESK	1	S8(Phospho); S12(Phospho)	S(1): 0.0; S(8): 88.2; T(10): 88.2; S(12): 23.7; T(23): 0.0; T(24): 0.0
Q92597	ESAAPAsPAPsPAPsPTPAPPQK	1	S5(Phospho); S7(Phospho)	S(1): 0.3; T(3): 51.5; S(5): 51.5; S(7): 95.7; S(8): 0.9; T(10): 0.0; S(11): 0.0; T(15): 0.0
O75351	EADDDEEVDDNIPEMPsPK	1	S14(Phospho)	S(5): 0.6; S(14): 99.4; S(20): 0.0
P60709	DQHLNNSSSsPQR	1		
Q92614	NTSAEKELSsPIsPK	4	S3(Phospho); S7(Phospho); S10(Phospho)	S(3): 100.0; S(7): 100.0; S(10): 0.0; S(11): 0.2; S(12): 1.2; T(14): 97.3; S(15): 1.2; Y(16): 0.0
P27824	IQDKEGIPPDQQR	7		
Q05519	SGsTGsSLSVSVR	1	S14(Phospho)	S(14): 100.0
Q13185	tSSTLDSEGTFNSYR	2	S3(Phospho)	S(1): 1.2; S(3): 98.7; S(5): 0.0; S(7): 0.0; S(10): 0.0
O95292	MLAEsDEsGDEESVSQTDKTELQNTLR	2	S3(Phospho); S4(Phospho)	S(1): 99.3; S(3): 0.7; S(4): 0.7; S(5): 99.3; T(9): 0.0
Q9UKK3	LSVPtsDEEDEVPAPKPR	2	T11(Phospho)	Y(2): 0.0; Y(5): 0.0; T(11): 100.0
Q9Y6M5	sREDLSAQPVQTK	3	S10(Phospho)	S(9): 0.7; S(10): 99.3
P15056	NQDEcVIALHDcNGDVNR	1	S4(Phospho)	S(3): 7.5; S(4): 92.5; T(15): 0.0
Q02952	RPKsNIAVEGR	1		
Q15121	SRsDNALHLASER	1	S7(Phospho)	S(7): 100.0
Q15831	VTNDIsPEsSPGVGR	2	S3(Phospho)	S(3): 8.2; T(4): 91.0; Y(8): 0.8
P78559	HEERQDEHGYISR	4	S9(Phospho)	S(3): 0.0; S(9): 100.0
Q08AD1	SEGDNYSATLLEPAASSLsPDHK	1	S3(Phospho)	S(1): 50.0; S(3): 50.0; S(9): 0.0; S(11): 0.0
Q8NE71	RKsQMEEVQDELIHR	1	T6(Phospho)	S(3): 0.0; T(6): 92.6; S(7): 7.4
P08238	RFsSGGEEDDFDR	1	S6(Phospho); S12(Phospho)	S(6): 100.0; S(12): 100.0
Q86X53	TIGGGDDSFNTFFSETGAGK	1	S10(Phospho)	S(10): 100.0; T(15): 0.0
Q8IYB3	TsSFAEPGGGGGGGGGPGGSASGPGGTGGGK	3	T3(Phospho); S5(Phospho)	T(3): 100.0; S(5): 100.0
Q99958	SEVQQPVHPKPLsPDSR	1	S8(Phospho); S13(Phospho)	T(3): 0.0; S(5): 100.0; S(8): 0.0; S(13): 100.0
P04083	IKDPDASKPEDWDER	2		
Q92614	FAsDDEHDEHDENGATGPVK	1	S7(Phospho); S11(Phospho)	S(7): 100.0; S(11): 100.0
Q969X1	DAEAWFNEK	1	S3(Phospho)	S(3): 99.9; S(5): 0.1
Q14315	ILEDHGsPAGEIDDEDKDKDETETVK	1	C7(Carbamidomethyl)	
Q9UHR5	DKEVsDDEAEEKEDK	1	S5(Phospho)	S(5): 89.8; Y(8): 10.2; S(14): 0.0
P46821	NHSGNDERDEEDEERESK	2	S3(Phospho); S8(Phospho)	S(3): 100.0; S(8): 97.0; S(11): 2.5; T(14): 0.5; S(15): 0.0; T(16): 0.0; S(17): 0.0; Y(19): 0.0
P27824	DTSPDKGELVsDEEEDT	2	S3(Phospho)	S(3): 100.0; T(11): 0.0; S(13): 0.0
Q9UPN3	DVtPPPETEVVLIK	4	S10(Phospho)	S(10): 100.0
P14314	VGDTEKPEPERsPPNR	4		
Q9H4G0	SSsPVQVEEEPVR	3	S4(Phospho)	S(4): 50.0; T(6): 50.0; T(17): 0.0
P43487	sSDSWEVWGSASTNR	2		
Q92521	DVDDGSGsPHsPHQLSSK	2	S1(Phospho)	S(1): 49.8; T(2): 49.8; Y(4): 0.4; T(7): 0.0
P37173	ANsPEKPPEAGAAHKPR	2	C2(Carbamidomethyl); S3(Phospho)	S(1): 1.0; S(3): 99.0; S(12): 0.0; T(15): 0.0; T(16): 0.0
Q13459	DHAEQQHIAAQQK	1	S3(Phospho)	T(2): 1.0; S(3): 81.4; S(5): 8.4; T(6): 8.4; S(7): 1.0; S(10): 0.0
P78559	SPLEHsSPEKEAPSPEK	2	S16(Phospho)	S(1): 0.0; T(2): 0.0; T(3): 0.0; S(4): 0.0; T(7): 0.0; S(16): 89.4; S(19): 10.6
Q9BV36	TDGsIsGDRQPVTVADYISR	2	S3(Phospho)	S(2): 50.0; S(3): 50.0; S(5): 0.1
Q2TAA2	MDsDEDEKEGEEEKVAK	1		
Q7Z698	sSPGGQDEGGFMAQGK	2	S3(Phospho)	S(3): 100.0
Q92974	sLEDVTAEYIHK	1	S15(Phospho)	S(10): 0.0; T(14): 0.3; S(15): 99.7; T(19): 0.0
Q03001	SSsMAAGLER	2	S10(Phospho); S11(Phospho)	S(10): 100.0; S(11): 100.0
Q16204	VGSLtPPSsPK	4	S3(Phospho)	S(3): 99.9; S(5): 0.1; S(7): 0.0; S(8): 0.0
Q96F63	NHEGDEDDSHVR	2	S17(Phospho)	Y(6): 0.0; S(17): 100.0
Q13200	EKKEESHSNDQSPQIR	1	S8(Phospho)	S(8): 100.0; T(16): 0.0; S(21): 0.0
Q96QB1	KVsKQEEASGGPTAPK	2	S3(Phospho); S6(Phospho); C12(Carbamidomethyl)	S(3): 100.0; S(6): 100.0; S(15): 0.0
P46821	VPKPEPIPEPKEPsPEK	1	S4(Phospho); S13(Phospho)	S(2): 92.2; S(4): 7.8; S(13): 7.8; S(15): 92.2
P11274	STTSQVTPAEEKDGHsPMSK	2	S31(Phospho)	S(2): 0.0; S(4): 0.0; S(31): 100.0
P56211	DSDLSHVQNK	1	S4(Phospho)	Y(1): 0.0; S(4): 91.2; Y(7): 8.8
O95786	RRDsDGVDGFEAEGK	1		
Q04637	KPsVSEEVQATPNK	1	C9(Carbamidomethyl); C11(Carbamidomethyl)	
Q07960	DADSQNPDAPEGK	1	S3(Phospho)	S(1): 0.0; S(2): 0.3; S(3): 99.7; T(8): 0.0
P27824	SIDDEITEAKSGtAtPQR	2	S3(Phospho); S13(Phospho)	S(3): 100.0; T(11): 13.3; S(13): 86.7
Q9H1E3	LDIDSPPITAR	1	S15(Phospho)	T(3): 0.0; S(5): 0.0; S(15): 100.0
Q86W92	NEQLPLQYLADVDTsDEESIR	4	S5(Phospho)	S(5): 50.0; S(7): 50.0
P14618	HLVYESDQNKDGK	2		
Q2M2I8	RIDFTPVsPAPsPTR	1	T5(Phospho); S9(Phospho)	S(3): 0.0; T(5): 96.1; S(8): 51.9; S(9): 51.9
Q13671	SSKAsLGsLEGEAEAEAsSPK	2	C8(Carbamidomethyl); S19(Phospho)	S(1): 0.0; S(19): 100.0
Q99638	DSIVAELDR	1	S13(Phospho)	S(1): 0.0; S(6): 0.0; S(13): 100.0
Q13625	DQDHLDKEIEK	1	S3(Phospho)	S(3): 99.0; S(4): 1.0
Q9UHB6	NSLDcEIVSAK	1	S4(Phospho)	S(4): 7.4; S(6): 92.5; S(9): 0.1; S(12): 0.0
Q8IYB3	RNLLEDDsDEEEDFFLR	2	S3(Phospho); S5(Phospho)	S(3): 100.0; S(5): 100.0
Q96A57	KRsEGFSMDR	1	S3(Phospho)	S(2): 7.7; S(3): 84.5; T(4): 7.7; Y(8): 0.0
Q5VZK9	SNDsGEEAEKEFIFV	1	S3(Phospho)	S(2): 49.8; S(3): 49.8; S(7): 0.4; S(11): 0.0
P05787	LRLsPsPTSQR	2		
P27816	SPsPPDGSPAATPEIR	4	S3(Phospho); S6(Phospho)	S(3): 100.0; S(6): 91.0; T(8): 9.0
Q5JSH3	GGLVsDAYGEDDFSR	1	S3(Phospho)	S(3): 100.0; T(9): 0.0
Q9P1Y6	DADETKEWIEEK	1	S6(Phospho); S17(Phospho)	S(5): 19.8; S(6): 83.6; S(17): 95.1; S(19): 1.1; S(21): 0.3
Q9NQT8	KPEEEsPRKDDAK	4	S3(Phospho)	S(1): 0.7; S(3): 98.6; S(4): 0.7
Q9UNE7	tAtcHSSSSPPIDAASAEPYGFR	1	S7(Phospho)	S(7): 100.0; S(11): 0.0; S(13): 0.0
P51858	DRHESVGHGEDFSK	2	S6(Phospho)	S(6): 89.7; S(7): 10.3
P53621	DQVTAQEIFQDNHEDGPTAK	1	S3(Phospho)	S(3): 100.0; S(9): 0.0
Q9BW71	ASsLEDLVLK	1	S5(Phospho); S9(Phospho)	S(1): 0.0; S(5): 100.0; S(9): 100.0
Q9NZ63	TEAQGEEDDAEGQDQDKKsPKPK	2	S12(Phospho)	T(4): 0.0; S(12): 100.0
Q86UP2	TKPtQAAGPSSPQKPPtPEETK	2	S10(Phospho)	S(10): 89.9; S(12): 10.1; S(14): 0.0
Q15293	TRsPsPTLGESLAPHK	1		
P14618	HHEEEIVHHKK	2	S5(Phospho)	S(5): 99.9; T(9): 0.1
P46821	GYSFTTTAER	1	S4(Phospho)	S(4): 100.0
Q9Y653	HVTLPSsPR	1	S9(Phospho); S10(Phospho)	S(4): 0.0; S(5): 1.1; S(7): 51.8; T(8): 51.3; S(9): 85.5; S(10): 9.2; S(11): 1.1
O95218	LPSHSFEIDHEDADKDEDTTSHSSSK	1	S1(Phospho); S3(Phospho)	S(1): 99.9; S(3): 99.7; S(6): 0.4; T(13): 0.0
Q969E4	SPAEcREEHSKtPEER	3		
Q969T4	DGGAWGTEQR	1	S9(Phospho)	S(3): 2.6; S(7): 11.0; S(9): 50.0; T(10): 11.0; S(11): 11.0; S(12): 11.0; S(14): 2.6; S(15): 0.6
Q09666	TGDLGIPPNPEDRsPsPEPIYNSEGK	12	S3(Phospho)	S(1): 0.0; S(3): 93.2; S(5): 6.8
Q86YS7	AGNsDSEEDDANGRVELILEPK	2	S3(Phospho); S5(Phospho)	S(1): 16.9; S(3): 79.8; S(5): 17.4; S(6): 70.8; T(10): 15.0; S(15): 0.2
Q7Z6Z7	SSsPELVTHLK	2	S4(Phospho); S7(Phospho)	S(4): 99.9; S(7): 50.0; T(8): 50.0; S(12): 0.0
Q9Y606	EKYIDQEELNK	1	T14(Phospho)	S(3): 0.0; S(8): 0.0; T(14): 100.0

Q9H4I2	ELISNASDALDK	1	S13(Phospho)	S(5): 0.1; S(6): 0.5; T(11): 33.1; S(12): 33.1; S(13): 33.1
Q02880	AKPPADPAAAAsPcR	1	S8(Phospho); S10(Phospho)	S(8): 89.5; S(10): 98.6; S(12): 11.9
P48651	GLFSDEEDsEDLFSSQSASK	2	S3(Phospho); C5(Carbamidomethyl)	T(1): 1.0; Y(2): 1.0; S(3): 98.1; T(9): 0.0; Y(10): 0.0; S(11): 0.0; S(15): 0.0
P35367	TFVNITPAEVGVLVGK	3	S3(Phospho)	S(1): 46.8; S(3): 46.8; T(5): 6.3; Y(9): 0.2
Q9UHB6	DNWEELYNR	1	S13(Phospho)	S(1): 0.0; S(13): 99.9; S(16): 0.1
Q9NVM6	DISTNYYASQK	1	S7(Phospho)	S(7): 100.0; S(13): 0.0
Q92922	DAYREFDRK	1	S3(Phospho); S5(Phospho)	S(3): 100.0; S(5): 100.0; T(10): 0.0; T(12): 0.0; S(14): 0.0
P46821	LQEKEDLQELNDR	1	S3(Phospho)	S(3): 100.0; S(8): 0.0; S(11): 0.0; T(14): 0.0; S(15): 0.0; T(16): 0.0; S(17): 0.0; Y(19): 0.0
P46821	ERRtPsDDEEDNLFAPPK	1	S3(Phospho)	T(1): 6.5; T(2): 6.5; S(3): 87.1; S(8): 0.0; Y(10): 0.0; S(11): 0.0; Y(12): 0.0
P05455	AGFAGDDAPR	1	S5(Phospho)	T(1): 2.0; S(5): 98.0; T(18): 0.0
Q9GZT3	VHNDAQSFDYDHDAFLGAEEAK	1	S5(Phospho)	T(4): 0.4; S(5): 99.6
Q9UHB6	SASQssLDKLDQELKEQQK	4	S5(Phospho)	T(2): 13.1; S(5): 86.9
Q8IYM9	DSYVGDEAQSKR	2	S1(Phospho)	S(1): 50.0; S(2): 50.0; S(9): 0.0; Y(12): 0.0; S(13): 0.0
Q9UGV2	HNsWSSSSR	1	S3(Phospho); S5(Phospho)	T(1): 6.7; S(3): 6.7; T(4): 85.9; S(5): 1.2; S(6): 7.3; S(7): 92.1; S(10): 0.0; S(13): 0.0; S(16): 0.0
O15164	RDsVLAASR	1	S9(Phospho); S12(Phospho)	S(3): 12.4; S(9): 87.6; S(12): 100.0
O15119	TESVSDKEDKPPLAPSGGTEGPEQPPPPcPSQTGsPPVGLIK	3	C3(Carbamidomethyl); S5(Phospho); S9(Phospho); C24(Carbamidomethyl)	S(5): 100.0; S(9): 99.8; S(15): 0.2
Q9P2B4	TNNVSEHEDTDKYR	1	S3(Phospho)	S(3): 100.0; T(5): 0.0; S(10): 0.0
P51116	RNsRDGDPLPSSLScK	1	S4(Phospho); S6(Phospho)	T(1): 0.3; S(4): 99.7; S(6): 99.9; T(13): 0.0; Y(17): 0.0; S(19): 0.0
Q8WUM9	YEWDVAEAR	1	C4(Carbamidomethyl); S5(Phospho)	S(5): 33.3; S(7): 33.3; S(9): 33.3
Q7Z5K2	IAcDEEFsDsEDEGEGGRR	4		
P78559	RDGPGLERsPGEPGGAAPER	1	S3(Phospho)	S(1): 49.4; S(3): 49.4; S(9): 1.2; S(11): 0.0; S(14): 0.0; S(17): 0.0; T(20): 0.0; T(26): 0.0
Q96FT9	LIEDNEyTAR	1	S3(Phospho)	S(3): 100.0
P19532	SDsLLSFR	1	S3(Phospho); S8(Phospho)	S(3): 100.0; S(8): 0.8; S(9): 8.2; S(11): 91.0; S(15): 0.0
Q96B23	TDEERPPVEHsPEK	1	S3(Phospho)	S(1): 0.0; S(3): 2.0; S(5): 2.0; T(7): 2.0; S(8): 12.0; T(9): 82.0
P78559	sFEEEGEHLGSR	1	S2(Phospho)	S(2): 50.0; T(4): 50.0
Q9H0B6	KLTsDEEGEPSGKR	1	S5(Phospho)	T(1): 0.0; S(3): 0.0; S(4): 0.6; S(5): 92.5; S(6): 6.9; S(10): 0.0
Q8NC51	DGTAPPPQSPGSPGTGQDEEWsDEESPRK	4	T1(Phospho)	T(1): 60.0; S(4): 13.3; S(5): 13.3; S(7): 13.3
Q96TA1	ADDLGKGGNEESTK	1	S7(Phospho); S11(Phospho)	S(6): 3.6; S(7): 96.4; S(11): 100.0
P07237	ASQsRPNsSALETLGGEK	3		
Q8IWW6	VTLTSEEEAR	1	T2(Phospho); S12(Phospho)	T(2): 50.0; T(3): 50.0; S(12): 100.0; Y(15): 0.0
O75190	QEYDESGPSIVHR	3	C5(Carbamidomethyl); S7(Phospho)	S(7): 100.0
O14737	ATAGDTHLGGEDFDNR	1	S5(Phospho)	S(5): 100.0; Y(11): 0.0
Q9NQC3	TYsEcEDGTYSPEISWHHR	1	S5(Phospho); S7(Phospho)	S(4): 53.5; S(5): 53.5; S(7): 90.4; T(11): 2.6; S(19): 0.0
P55040	SQEPIPDDQKVsDDDKEK	1	S3(Phospho); C4(Carbamidomethyl)	S(1): 94.0; S(3): 6.0; S(8): 0.0
P07900	SSsLGSYDDEQEDLTPAQLTR	3		
Q5JRA6	DREVGIPPEQSLETAK	1	S8(Phospho)	Y(6): 14.8; S(8): 85.2
O75368	FAsDDEHDEHDENGATGPVKR	2		
P28290	sPcGLTEQYLHK	1	S5(Phospho); S6(Phospho); S9(Phospho)	S(3): 92.5; S(5): 51.8; S(6): 51.8; T(8): 51.3; S(9): 51.3; T(13): 1.3; T(20): 0.0
Q9Y2K6	HTHIDKPDcSGPPMDISNK	1	S3(Phospho); C5(Carbamidomethyl)	S(1): 0.6; S(2): 7.4; S(3): 91.9; T(7): 0.1
Q13263	TDsREDEIsPPPPNPVVK	6	S3(Phospho)	S(1): 0.9; S(3): 99.1; S(9): 0.0
Q8TD55	RYsPsPPPK	3	C1(Carbamidomethyl); S3(Phospho)	S(2): 10.4; S(3): 89.6
Q9UQ35	NLQEAEEWYK	1	C2(Carbamidomethyl); S5(Phospho); S6(Phospho)	S(1): 0.2; S(5): 99.8; S(6): 100.0
Q9UPU5	RADLNQGIGEPQsPSR	2	S6(Phospho)	S(2): 0.0; S(6): 100.0
Q7L2J0	KREDcSPADKPYIDEAR	2	S3(Phospho); C4(Carbamidomethyl)	S(3): 100.0
P29966	MGPSGGEGMEPERRDsQDGSSYR	2	T13(Phospho)	T(6): 0.0; S(8): 1.1; S(10): 9.5; T(13): 89.4
P13639	REDsFESLDSLGSR	3		
Q09666	DRYDSFIELQEK	2	S6(Phospho); S9(Phospho); S12(Phospho)	S(5): 54.6; S(6): 54.6; S(9): 92.3; S(12): 98.6; S(22): 0.0; S(23): 0.0
P04083	LAADDFR	1		
Q9Y2H0	DSAIPVEsDtDDEGAPR	2	T5(Phospho)	S(3): 11.0; T(5): 76.3; S(7): 11.0; S(10): 1.7; Y(14): 0.0; T(20): 0.0
P27797	YISPDQLADLYK	1		
Q1KMD3	DLEGLSQR	1	S11(Phospho)	S(11): 100.0; S(14): 0.0; S(19): 0.0
P46821	NHEEEMKDLR	1	S13(Phospho); S15(Phospho)	S(13): 100.0; S(15): 100.0
O14639	QLHQScQTDDGEDDLKK	1	S3(Phospho)	S(2): 0.8; S(3): 99.2
Q02952	NKYEDEINKR	2	S6(Phospho)	S(1): 0.1; T(3): 0.1; S(5): 5.2; S(6): 47.3; T(7): 47.3; S(9): 0.1; T(10): 0.0; S(12): 0.0
Q13459	LQDAEIAR	1	S6(Phospho)	S(1): 0.0; S(6): 50.0; S(7): 50.0; S(14): 0.0
Q92615	EHQHEEIQNVR	1	S3(Phospho)	S(1): 1.1; S(3): 98.9
O14737	EDsFESLDSLGSR	1	S4(Phospho)	S(4): 100.0; Y(10): 0.0
Q8N1F8	SNsREHLGGGSESDNWR	1	S3(Phospho)	S(3): 49.8; S(5): 49.8; S(8): 0.3; T(10): 0.0
P10451	DAVLLVFANK	1	S6(Phospho)	S(6): 15.5; S(10): 84.4; S(15): 0.0; S(19): 0.0
Q9Y6X9	ATAPQTQHVsPMr	1	S7(Phospho); S9(Phospho)	S(3): 0.0; S(7): 100.0; S(9): 100.0; S(15): 0.0
Q8IXJ6	KADKESRPENEEERPK	4	S9(Phospho); S11(Phospho); C-Term(Oxidation)	S(7): 90.7; S(9): 54.7; S(11): 54.7
P27824	MsGFIYQGK	1		
P09651	LSsPVLHR	1	S3(Phospho)	S(1): 0.9; S(3): 99.1
P48741	DLEEDHAcIPIK	2		
Q96F86	QKsDAEEDGGTVSQEEEDR	2	S3(Phospho)	S(3): 33.3; S(5): 33.3; S(6): 33.3; S(7): 0.0; S(8): 0.0
O43399	EsSPEKEAEEGcPEKESEEGcPK	2	S7(Phospho)	S(2): 0.0; T(4): 0.0; S(7): 100.0
Q9BV36	HHNsTAELQK	2	T1(Phospho)	T(1): 49.9; T(2): 49.9; S(7): 0.2
O75400	QNsDPTSENPPLPTR	1	S5(Phospho); S8(Phospho)	S(3): 8.9; S(5): 91.1; S(8): 100.0
O95677	DRPHASGTDGDEsEEDPPEHKPSK	3	S4(Phospho)	S(4): 100.0; S(9): 0.0
Q92614	DAENHEAQLK	1	S5(Phospho); S9(Phospho)	S(5): 100.0; S(9): 100.0
Q5SW79	SLSsPTDNLELSLR	2	S12(Phospho)	T(10): 14.6; S(12): 82.6; S(14): 2.8
Q14155	DGDSVMVLPTIPEEEAK	1	S3(Phospho)	S(3): 100.0
P17936	sRPTsFADELAAR	1	S19(Phospho)	Y(1): 0.0; Y(5): 0.0; S(7): 0.0; S(9): 0.0; T(10): 0.0; T(12): 0.3; S(16): 85.9; S(17): 12.0; S(19): 1.8
P46821	LQPHPGLEKKEEEEEEEYDEGSNLK	3	S4(Phospho); T7(Phospho)	S(1): 0.0; S(4): 100.0; T(7): 100.0
Q9UKE5	RGsIGENQIKDEK	4	S3(Phospho)	S(3): 100.0; T(6): 0.0; S(7): 0.0; T(14): 0.0
Q9BUA3	RPsESDKEDELDKVK	5	S5(Phospho); S9(Phospho)	S(5): 96.6; S(9): 6.7; S(11): 96.6
Q16637	RHsMQTEQIR	1	T3(Phospho)	T(3): 84.4; S(6): 13.3; S(9): 2.3; T(15): 0.0
Q13427	GDQccYSHsPPTPR	1	S7(Phospho)	S(7): 99.9; S(10): 0.1
Q9NRF8	KLsGDQPAAR	2	S8(Phospho); S11(Phospho)	S(2): 0.0; S(3): 0.0; S(4): 0.0; Y(7): 1.1; S(8): 98.9; S(11): 100.0; S(14): 0.0; S(16): 0.0
Q04727	RGEsLDNLDSPR	1	S5(Phospho)	S(1): 0.0; S(2): 0.0; S(3): 0.1; S(5): 78.8; S(7): 10.6; S(9): 10.6
Q07960	NGEsSELDLQGIR	2		
P28715	VKEEPPsPPQsPR	1	S13(Phospho)	T(3): 0.0; T(10): 0.0; S(13): 100.0
Q8WWI1	McKDEDEcEEGKHDcTEK	1	S3(Phospho)	S(3): 99.4; Y(4): 0.5; T(5): 0.0; S(6): 0.0
O60930	SMsIDDTPR	2	S4(Phospho)	S(2): 12.7; S(4): 87.3; S(8): 0.1; S(19): 0.0
Q96JC9	SASQssLDKLDQELK	3	T1(Phospho); S9(Phospho)	T(1): 50.0; S(2): 50.0; S(9): 100.0
P31948	REsPSPAPKPR	3		
P13639	SLMSsPEDLTK	1		
Q8IYB3	QGsPDQVSPVSEMTSTSLYQDKQEGK	1	S4(Phospho); T8(Phospho)	S(4): 100.0; T(8): 100.0

Q8WXF7	DAHSQGEVVScLEK	1	S4(Phospho)	S(4): 100.0; S(9): 0.0
P67936	NEFGEIQGDK	1		
O15541	LVIITAGAR	1	S7(Phospho); S8(Phospho)	Y(3): 92.6; S(7): 53.7; S(8): 53.7; S(18): 0.0; Y(23): 0.0
Q6WCQ1	KEESEEsDDDMGFGLFd	1	S3(Phospho)	S(1): 0.5; S(3): 99.5
P08559	NADVELQQR	1	S5(Phospho)	Y(1): 5.0; S(5): 90.1; S(7): 5.0; S(12): 0.0; Y(13): 0.0
P41229	TLHPDLGTDKDKEQWK	3	S12(Phospho); C16(Carbamidomethyl)	T(1): 0.0; S(5): 1.3; S(10): 1.3; S(12): 97.3; T(17): 0.0
P08670	EHASIDAQSGAGVPNPStSASPK	4		
Q9BW71	SSStSDILEPFTVER	10	S7(Phospho); S10(Phospho)	S(7): 100.0; S(10): 100.0
Q03001	ESsPIPsPTSDR	1	S3(Phospho)	S(3): 92.5; S(5): 7.5; S(10): 0.0
P35269	DLEIERPILGQNDNK	1	T10(Phospho); T11(Phospho)	S(1): 0.0; T(2): 0.0; S(7): 98.2; T(10): 13.7; T(11): 88.0; S(14): 0.0; T(20): 0.0
Q92945	GAAsPVLQEDHcDSLPSVLQVEEK	1		
Q9Y2V2	DHRPPcAQEAPR	1	S3(Phospho); S5(Phospho); S14(Phospho)	S(3): 100.0; S(5): 100.0; S(14): 98.5; T(18): 1.5
O60271	EKtYSEcEDGTYSPEISWHHR	2	S2(Phospho)	S(1): 49.7; S(2): 49.7; T(3): 0.6; S(5): 0.0
P16070	GVDEATIIDILTK	1	S22(Phospho)	T(1): 0.0; Y(7): 0.0; S(9): 0.1; T(12): 0.4; S(17): 7.8; S(18): 7.8; S(20): 7.8; S(21): 38.0; S(22): 38.0
Q96N67	DFQEYVEPGEDFPAsPQRR	5	S1(Phospho)	S(1): 99.9; S(3): 0.1; T(7): 0.0
Q09666	TsPPcsPANLSR	1	S10(Phospho)	S(9): 8.3; S(10): 91.7
P78559	DYNVTANSK	1	S7(Phospho)	S(7): 68.1; T(11): 14.2; S(13): 14.2; S(15): 3.2; S(17): 0.2; S(20): 0.0; S(22): 0.0
O00299	DLGLSESGEDVNAAILDESGKK	1		
P26358	ELVLssPEDLTQDFEEMKr	1	S17(Phospho)	S(17): 100.0
Q9NSD9	KADGPPGPHDGGDRPSAEAR	2		
P09497	DLTHsDSESSLHMSDR	1		
Q8TCU6	NDHQEDFWK	1	S5(Phospho)	S(2): 0.0; S(5): 32.5; Y(6): 32.5; T(7): 32.5; S(8): 2.4
Q09161	RPDPDsDEDEDYERER	2	S3(Phospho); C17(Carbamidomethyl)	T(2): 1.8; S(3): 96.3; T(8): 1.8; S(14): 0.0
P62070	FEDENFILK	1	C6(Carbamidomethyl); S9(Phospho)	S(9): 100.0; T(13): 0.0
Q8TDY2	DYLLcDYNR	1	S1(Phospho); S7(Phospho)	S(1): 50.0; T(2): 50.0; S(7): 100.0
Q6WCQ1	TEEARPSPAPGPGtPTGTPTR	2	S11(Phospho); S14(Phospho)	S(11): 98.5; S(14): 45.9; S(16): 47.1; T(17): 8.5
Q9BXF6	LSVPTsDEEDEVPAPKPR	7	S3(Phospho)	T(1): 0.3; Y(2): 0.3; S(3): 99.5
Q04637	NNFEGEVTK	1	S2(Phospho)	S(2): 99.5; S(4): 0.5
Q15293	TDSREDEIsPPPPNPVVK	9		
Q9NWW5	SPsPAHLPDDPKVAEK	2	S3(Phospho)	S(3): 100.0; S(5): 0.0
Q9UKA4	TAsFSESRADEVAPAKK	1	T3(Phospho); C6(Carbamidomethyl)	S(1): 0.9; T(3): 99.0; S(5): 0.1
Q09666	YDERPGPsPLPHR	2	S4(Phospho)	S(4): 100.0; T(10): 0.0; T(12): 0.0
Q8NHQ9	DALNIETAIK	1	S7(Phospho); C13(Carbamidomethyl)	T(1): 0.0; S(7): 100.0
O75379	AYtHQVVTR	1	S7(Phospho)	S(7): 100.0
Q52LW3	QKsDAEEDGGTVsQEEEDRKPK	5	S4(Phospho)	T(2): 1.4; S(4): 98.6; S(9): 0.0; S(12): 0.0; S(15): 0.0; S(16): 0.0; S(17): 0.0
Q9UKS6	sIDAALFR	1	S22(Phospho)	T(3): 0.0; S(9): 0.0; S(12): 0.0; T(15): 0.1; S(22): 99.8; S(26): 0.0
P55196	DSNLHSSTDKEQAER	1	S3(Phospho)	T(1): 1.2; S(2): 1.2; S(3): 97.6; T(6): 0.0
P55786	SSSISEEKGDsDDEKPR	1		
Q9BQE3	VNsGDTEVGSSLLR	1		
P43243	SEsPKEPEQLR	4	S3(Phospho)	S(1): 0.1; Y(2): 1.0; S(3): 98.9; S(9): 0.0; S(11): 0.0
P04083	ASALLFsSDEEDQWNIPASQTHLASDSR	3	S4(Phospho)	T(3): 0.6; S(4): 6.8; T(6): 6.8; S(7): 85.8
Q2VPK5	NRVPsAGDVEK	1	C2(Carbamidomethyl); S7(Phospho)	S(7): 100.0; S(14): 0.0
P07900	sLQLMDNR	1	S5(Phospho)	S(5): 100.0
P10644	DTDSEEEIREAFR	1	S9(Phospho)	T(1): 0.0; S(3): 0.0; S(9): 100.0
Q07866	sRsRDsGDENEPIQER	1	S1(Phospho); S4(Phospho)	S(1): 100.0; S(4): 100.0
Q15154	ETPHsPGVEDAPIAK	2	S9(Phospho)	S(7): 0.5; S(8): 4.5; S(9): 47.5; S(10): 47.5
P08670	SSKAsLGsLEGEAEAEAsSPKGK	2		
Q9NZT2	LTASEQAHPQEPAESAHEPRLSAEyEK	2	S1(Phospho)	S(1): 100.0
Q9NP61	GQGESDPLDHEPAVsPLLPR	2		
P17302	GGsPIIQEPEEPSEHREESSPR	1	S3(Phospho)	S(3): 84.5; Y(10): 13.2; S(11): 2.3
Q96ST2	ETERAsPIKMDLAPSK	2	S4(Phospho)	S(2): 3.7; S(4): 96.3; S(15): 0.0; S(17): 0.0; T(19): 0.0; S(21): 0.0
Q9Y6R9	EDKEKEEEEEEKPK	1	S3(Phospho); S6(Phospho)	S(3): 99.8; S(6): 96.8; S(11): 3.3; S(13): 0.0; S(15): 0.0
Q9GZY8	IQLVEEELDR	1	S3(Phospho)	S(3): 100.0
Q15084	RSsRDMAGGAGPR	1		
Q5FWE3	APIIAVTR	1	S1(Phospho)	S(1): 100.0
P48634	NRPEDYQGGR	1	S5(Phospho)	S(3): 0.0; S(5): 47.4; S(8): 47.4; S(9): 4.7; S(10): 0.5
O75962	FNGGHsPTHSPEK	1	S7(Phospho)	S(7): 97.9; S(9): 1.0; S(10): 1.0
Q96CW6	YHPDKNKEPGAEEK	2	S6(Phospho); S12(Phospho)	Y(4): 0.3; S(6): 99.7; S(12): 100.0
Q9BXW6	KVVVsPTK	3	S1(Phospho)	S(1): 100.0; S(11): 0.0
O75592	EAELKEAEKELHEK	2	S3(Phospho)	S(3): 100.0; T(6): 0.0; S(10): 0.0; S(11): 0.0
Q14643	SDEDKDKEGEALEVK	2	S3(Phospho)	S(3): 100.0; S(8): 0.0
Q99607	STIGVMVTAsHNPEEDNGVK	2	T4(Phospho)	T(4): 92.7; S(5): 7.3; S(11): 0.0
P67809	SSsSDSIHSVR	3		
Q9Y2H5	GEPNVSyIcSR	5	S3(Phospho)	T(1): 8.4; S(3): 90.8; T(5): 0.8; T(10): 0.0; S(12): 0.0
P01889	ITGDDSMR	1		
P01891	SWsPPPEVSR	3		
Q9Y5K6	ADEGISFR	1	S1(Phospho)	S(1): 100.0; S(6): 0.0; T(8): 0.0
P46108	DLKPHEDQQDINKDVGVK	2		
Q8ND76	SYsSPDITQAIQEEEKR	4	S3(Phospho)	S(1): 1.1; S(3): 98.9; T(8): 0.0
Q9UQE7	DENQSINHQMAQEDAQR	1		
P27816	KKEEEATEWQHK	1	S5(Phospho)	S(1): 0.0; S(5): 99.5; T(6): 0.5
P63208	WRPHsPDGPR	3		
O95394	QKGsEENLDEAR	1	S10(Phospho)	S(1): 0.0; T(2): 0.0; T(8): 2.0; S(10): 98.0
Q12906	AALAPAKEsPR	1	S11(Phospho)	S(11): 1.2; S(13): 98.8
Q13009	RVEsEEsGDEEGKK	1	S3(Phospho)	S(3): 100.0; Y(8): 0.0
Q9NRA8	GQLTKsPLAQMEEERR	1	S3(Phospho)	S(1): 0.1; S(2): 0.9; S(3): 99.0
P14618	NLLSVAYK	1		
P18887	KGPGQPsSPQR	2	S11(Phospho)	S(4): 0.0; T(5): 0.0; S(6): 1.2; S(11): 98.7
Q7Z2W4	cTsHSETPTVDDEEKVDER	4	S1(Phospho); C2(Carbamidomethyl); S5(Phospho)	S(1): 0.0; T(3): 100.0; S(5): 100.0; S(10): 0.0
O95159	RTDALTSsPGR	3	C12(Carbamidomethyl)	
P08648	SPTPKsPPSR	4	S7(Phospho); S8(Phospho)	S(4): 86.6; S(5): 13.3; S(7): 13.1; S(8): 85.2; S(9): 1.8; Y(17): 0.0
Q8TDY2	RPsQGPPAR	1	T2(Phospho)	S(1): 50.0; T(2): 50.0; S(7): 0.0
Q9UHB7	NSQEDsEDsEDKDVK	1	S3(Phospho)	S(1): 50.0; S(3): 50.0; S(13): 0.0; S(15): 0.0
P05386	GYSSLDQSPDEKPLVALDTDsDDDFDMSr	2	S7(Phospho); C-Term(Oxidation)	S(4): 0.0; S(7): 100.0
P48651	EESEPEVKEDVIEKAELEEMEEVHPsDEEEEDATK	2	S5(Phospho)	S(2): 0.1; S(5): 99.9; S(15): 0.0; S(17): 0.0; S(18): 0.0
Q14697	RSsQPsPTAVPAsDSPPTKQEVK	1		
Q14103	AGSISTLDSLDFARYsDDGNRETDEK	2	S16(Phospho)	S(4): 0.0; S(13): 0.1; S(15): 50.0; S(16): 50.0
Q5VTL8	TLEVVsPsQsVTGSAGHTPYYQSPTDEK	4	S3(Phospho); S5(Phospho)	S(3): 100.0; S(5): 100.0; S(10): 0.0

P51858	ASETDTHsIGEK	1	S8(Phospho)	S(8): 100.0
Q5TH69	KDsISEDEMVLR	1	S4(Phospho); S7(Phospho)	Y(1): 46.5; S(2): 46.5; S(4): 21.9; S(7): 85.0; S(14): 0.1
O60333	NQATSAtSEKDNDDDQSDKGtYTIELENPNSEEVEAR	1	S1(Phospho); S4(Phospho)	S(1): 100.0; S(4): 100.0
Q02952	LLHEDLDEsDDDMDEKLQPSPNR	2	S3(Phospho); S5(Phospho)	S(3): 100.0; S(5): 100.0
Q12802	HGEVcPAGWKPGSDTIKPDVQK	2	S5(Phospho)	S(3): 0.0; S(5): 48.2; T(6): 48.2; S(8): 3.5
Q9P035	RRDEDMLYsPELAQR	1	S5(Phospho)	S(5): 100.0
Q9BZL4	TLEVVSPSQSVtGSAGHtPYYQSPTDEK	1	S3(Phospho)	S(1): 41.7; S(3): 41.7; S(4): 8.3; S(5): 8.3; T(10): 0.0; S(11): 0.0; T(12): 0.0
Q9NQG7	SRsPLELEPEAKK	1	S9(Phospho)	S(7): 4.4; S(8): 47.8; S(9): 47.8
Q9H8Y8	DLStSPKPsPIPsPVLGR	5	S23(Phospho)	S(4): 0.0; T(5): 0.0; S(8): 0.0; S(13): 0.0; S(21): 0.1; S(23): 99.9
O00193	SPGsTPTTPTsSQAPQK	1	S3(Phospho)	S(1): 50.0; S(3): 50.0; S(10): 0.0; S(11): 0.0
Q9NWL6	TSsTcsNEsLSVGGTSVTPR	2	N-Term(Acetyl); C1(Carbamidomethyl); S9(Phospho)	S(5): 18.9; S(9): 81.1; S(19): 0.0
P09651	LLRDDEAHLQEDQGEEEcFHDcSAsFEEEPGADKVENK	2	S3(Phospho)	S(1): 0.3; S(3): 99.7
O14972	VKEVHDELEDLPsPPPPLsPPPTTSPHK	2	C16(Carbamidomethyl); C-Term(Oxidation)	
Q9BW71	QGEDLAHVQHPTGAGPHAQEEDsQEEEEEDEEAASR	2	T4(Phospho); C6(Carbamidomethyl); S7(Phospho)	T(4): 100.0; S(7): 100.0
P46821	ANsPSLFGTEGKPK	1	S2(Phospho)	S(2): 49.9; T(4): 49.9; S(9): 0.2; Y(17): 0.0
Q9Y4B5	VSPAsPAGsPSADFAVHGESLGDR	1	T4(Phospho); S8(Phospho)	T(4): 100.0; S(8): 100.0
P46821	VVRPDSELGERPPEDNQSFQYDHEAFLGKEDSK	1	S3(Phospho)	S(3): 100.0; T(7): 0.0; T(11): 0.0
Q8TEH3	AVFVDLEPTVIDEVR	1	S3(Phospho); S6(Phospho)	T(2): 50.0; S(3): 50.0; S(6): 100.0; Y(13): 0.0
Q9Y6M7	EVLDEDtDEEKETLK	1	S7(Phospho)	S(4): 13.1; S(7): 73.3; S(11): 13.1; T(12): 0.5; S(16): 0.0
P23528	HSDsKEDDGQEIA	1		
Q09666	TQDGVALEIQPLNsQEGIDNEEKDKK	1	S5(Phospho); C-Term(Oxidation)	S(5): 100.0; T(11): 0.0; T(13): 0.0
Q9NWQ8	DQPsPcLSSAEDSGVDEGQGSPSEMVHSSEFR	1	Y4(Phospho)	Y(4): 99.8; S(6): 0.1; S(8): 0.1
Q9NR09	NYDPYKPLDItPPPDQK	1	S12(Phospho)	S(5): 0.0; S(12): 13.5; S(14): 86.4; S(18): 0.0
Q7L4I2	RASEDTTSGsPPKK	2	S8(Phospho)	S(3): 0.0; S(6): 0.2; S(8): 99.8
Q9H1E3	AVVVsPKEENK	1	S3(Phospho); S5(Phospho); S9(Phospho)	S(3): 100.0; S(5): 100.0; S(9): 100.0
Q86YD1	EIKcSPsEsPLMEK	2	S3(Phospho)	S(3): 100.0
O60832	GRAsSHSSQTQGGGSVTK	2	S4(Phospho); S6(Phospho)	S(4): 91.2; S(6): 99.1; S(8): 9.7; T(11): 0.0
Q9NX55	GRNDsGEENVPLDLTREPSDNLR	2		
P27824	EEKDKDDQEWEsPSPPKPTVFISGVIAR	1	S13(Phospho)	S(3): 0.0; T(11): 0.2; S(13): 99.8
P60709	RGEGDAPFSEPGTTSTQRPSsPETATK	5		
P08238	SQDATFSPGSEQAEKsPGPIVSR	1		
Q15052	NRPtSISWDGLDSGK	2	S3(Phospho)	S(3): 100.0; Y(7): 0.0
O95208	KPDGVKEStESSNtTIEDEDVK	1	S3(Phospho)	S(2): 8.4; S(3): 91.6; S(8): 0.0; T(9): 0.0; S(10): 0.0; S(12): 0.0; Y(16): 0.0
Q9BZE4	QIDSsPVGGETDETTVSQNYR	2	S10(Phospho)	S(5): 5.4; S(9): 47.3; S(10): 47.3
Q9UBL6	SLLSHEFQDEtDtEEETLYSSK	2	S10(Phospho); C13(Carbamidomethyl); T14(Phospho)	S(1): 0.8; S(10): 99.2; T(14): 100.0
Q86UX6	MLQAIsPKQsPsSSPTR	1	S4(Phospho); S8(Phospho)	S(3): 92.0; S(4): 8.0; S(8): 0.8; S(11): 99.2
Q15052	KVsSAEGAAKEEPK	1	S3(Phospho)	S(3): 99.9; Y(7): 0.1
P35241	ALSAsHTDLAH	1		
Q9H7D0	RVEsEESGDEEGKK	1	S1(Phospho)	S(1): 99.6; T(5): 0.3; S(6): 0.0; S(10): 0.0
Q9UHQ4	DKsPSSLLEDAK	1	C8(Carbamidomethyl)	
P78559	TQFEDLVPsPTSEK	1	S5(Phospho)	S(5): 100.0; T(18): 0.0
P09382	DIIRQPsEEEIIK	3		
O60716	DAELQDQEFGKRDsLGTYSSR	2	S5(Phospho)	S(2): 0.0; S(5): 100.0; S(8): 0.0
Q05682	VVTQRsEIGEKQDTELQEK	2		
P49761	ENPPVEDsSDEDDKRNQGNLYDK	2	C1(Carbamidomethyl); S5(Phospho); S7(Phospho)	S(5): 98.7; S(7): 98.7; Y(12): 2.4; Y(13): 0.3; S(16): 0.1
O94929	VAPEEHPVLLTEAPLNPK	1	S2(Phospho)	S(2): 50.0; S(3): 50.0; Y(6): 0.0; S(9): 0.0; T(11): 0.0; Y(12): 0.0; S(13): 0.0
Q8WUY3	KDsLSQLEEYLR	1	S16(Phospho)	S(2): 0.0; S(16): 100.0
P47756	DAEYIYPSLEsDDDDPALK	1	C5(Carbamidomethyl)	
P13693	IKEEVLsESEAENQQAGAAALAPEIVIK	2	C8(Carbamidomethyl)	
Q8IWB9	RKTsQSEEEEAPR	2	S5(Phospho); S9(Phospho)	T(1): 1.3; S(4): 15.4; S(5): 15.4; T(8): 61.0; S(9): 76.2; S(11): 26.7; T(13): 4.1
Q9NTJ3	RAEDGsVIDYELIDQDAR	3	S15(Phospho)	S(10): 11.6; S(15): 76.7; S(16): 11.6; S(24): 0.0
Q9ULG6	YVSGssPDLVTR	1	S3(Phospho)	T(1): 12.7; S(3): 61.8; S(5): 12.7; S(7): 12.7; T(17): 0.2; S(20): 0.0
Q9H1B7	EIKcsPSESPLMEK	3	S4(Phospho)	S(4): 97.6; S(6): 0.2; S(7): 1.1; T(8): 1.1
Q14669	DIHDDQDYLHSLGK	1	S3(Phospho)	S(1): 1.1; S(3): 98.9; S(10): 0.0
P48741	sLEGGGcPAR	1		
Q86U06	DVDDGSGSPHsPHQLSSK	2	S3(Phospho)	S(3): 100.0; S(13): 0.0
Q9UDY2	YLSPGWGsAsEEEPSR	1	S5(Phospho)	S(1): 0.0; S(5): 100.0
P46821	IDEDGENTQIEDTEPMsPVLNSK	1	S3(Phospho)	S(3): 100.0; S(8): 0.0; S(11): 0.0; T(14): 0.0; S(15): 0.0; T(16): 0.0; S(17): 0.0; Y(19): 0.0
Q99501	AcAsPSAQVEGSPVAGSDGSQPAVK	2	S3(Phospho); S5(Phospho)	S(3): 100.0; S(5): 100.0
Q8IYB3	TPVDEsDDEIQHDEIPTGK	1	T3(Phospho); S5(Phospho)	T(3): 100.0; S(5): 100.0
Q6UXH1	DNQHQDEAEGDPGNRHEPR	2	S7(Phospho)	T(1): 0.0; S(3): 0.0; Y(5): 3.7; S(7): 48.1; S(8): 48.1
Q53GL0	SPsPPDGsPAATPEIR	2	C1(Carbamidomethyl); S3(Phospho)	S(3): 100.0; S(9): 0.0
P04792	GAEsPFEEK	2	S3(Phospho)	S(3): 99.2; S(4): 0.8; S(7): 0.0
P00338	DKTEDENKQSFLDGGK	1		
Q9Y3P9	SRLtPVSPESSSTEEK	2	S12(Phospho)	T(5): 0.2; S(7): 6.8; T(8): 46.5; S(12): 46.5; T(17): 0.0
P08559	GILAADESTGsIAKR	3	S6(Phospho)	Y(1): 0.0; T(5): 50.0; S(6): 50.0
P05787	ATHGSNsLPSSAR	1		
O43852	GDsLILEHQWELEK	1		
Q9NRA8	ISHELDSAsSEVN	1	S20(Phospho)	S(2): 0.0; S(8): 13.3; S(9): 60.6; S(10): 3.1; S(11): 3.1; S(14): 13.3; S(19): 3.1; S(20): 3.1; T(22): 0.2; T(23): 0.1; S(25): 0.1
Q15751	GPPDFssDEEREPTPVLGsGAAAAGR	2	S8(Phospho)	S(8): 100.0; T(10): 0.0; Y(12): 0.0
P00441	HREDSDVEMVEDDSRK	1		
P62328	DYEIESQNPLAsPTNTLLGSAK	1		
P25205	SAFTPATATGSsPsPVLGQGEK	2	S11(Phospho); T13(Phospho); C-Term(Oxidation)	S(4): 0.0; Y(5): 0.0; Y(8): 0.1; S(11): 85.3; T(13): 14.7; T(22): 99.9
Q8TER5	GNAEGSsDEEGKLVIDEPAK	1	S10(Phospho)	S(10): 100.0
P00367	RRsQsIEQESQEK	1		
Q15642	ESESVSKEEKEQNYDLTEVSESMK	2	S3(Phospho)	S(3): 100.0; S(5): 0.0; S(6): 0.0; T(9): 0.0; S(11): 0.0
P12268	AEDGATPSPsNETPKK	1	S4(Phospho)	S(4): 100.0
Q9BW71	KLLEAHEEQNVDSYTESVKEYDSISR	1	S10(Phospho); S11(Phospho)	S(10): 100.0; S(11): 100.0; Y(18): 0.0
Q9ULX3	YPIEHGIITNWDDMEK	1	S6(Phospho)	S(6): 100.0
Q8N6T3	KLTsDEEGEPSGK	2	S2(Phospho)	S(2): 100.0; S(7): 0.0; T(9): 0.0; S(11): 0.0; S(12): 0.0
Q9UDY2	GPSSEGPEEEDGEGFSFKYsPGK	2	S5(Phospho)	S(2): 29.7; Y(3): 29.7; S(5): 29.7; Y(12): 5.2; S(17): 2.8; S(20): 2.8
Q01082	sWNQGtPPLELLAERESLyR	1	S4(Phospho)	S(4): 100.0; S(8): 0.0; T(9): 0.0
Q53GL0	SGLsLEELR	1	S4(Phospho)	S(4): 100.0; S(10): 0.0
Q66K74	GFENVIHDKLPLQEsEEEEREER	1	S1(Phospho); C10(Carbamidomethyl); C15(Carbamidomethyl)	S(1): 50.0; S(3): 50.0; S(13): 0.0
O43310	RPAsPsSPEHLPATPAESPAQR	1	S1(Phospho)	S(1): 100.0
Q14103	GDRsPEPGQTWTR	1	S15(Phospho); S16(Phospho)	S(4): 0.0; S(13): 99.9; S(15): 8.8; S(16): 91.3
Q96N67	DWSDEPIKEcGTNEcLDNNGGcSHVcNDLK	1	S1(Phospho); S3(Phospho)	S(1): 100.0; S(3): 100.0; T(7): 0.0
P41227	KNsITEISDNEDDLLEYHRR	1	S3(Phospho); C11(Carbamidomethyl)	S(3): 99.7; S(6): 0.2; S(7): 0.0

O43852	AMVDSQQKsPVK	1		
Q12907	RKPsPEPEGEVGPPK	3		
P53396	YSQKEDKYEEEIK	2	S3(Phospho)	T(1): 0.2; S(3): 99.8; S(5): 0.0; S(7): 0.0
Q05519	RRPcEQIsPEEEER	1	C4(Carbamidomethyl); S5(Phospho)	T(1): 1.7; S(5): 98.0; T(10): 0.3; S(13): 0.0
P21333	GGFDsPFYR	1		
Q12982	TSsFTEQLDEGTPNRENASTHASK	6	S3(Phospho)	S(3): 99.9; T(5): 0.1; Y(7): 0.0; T(8): 0.0
P46821	RHAsAPSHVQPSDSEK	1	C23(Carbamidomethyl); S25(Phospho)	S(2): 0.0; S(11): 0.0; T(15): 0.0; S(25): 100.0
O00423	DQVANSAFVER	1	T1(Phospho); S8(Phospho)	T(1): 33.2; S(2): 33.2; S(3): 33.2; S(4): 1.2; S(8): 99.1
Q9Y2D5	KAALLAQYADVtDEEDEADEKDDSGATTMNIGSDK	1	S12(Phospho)	S(6): 0.0; S(12): 0.4; S(14): 7.7; S(15): 7.7; T(16): 41.1; T(17): 41.1; S(18): 1.6; S(19): 0.4
P25205	AVDHEETPVRYSSsEVNHLSPR	3	T13(Phospho); T22(Phospho)	S(4): 0.0; Y(5): 0.0; Y(8): 2.5; S(11): 14.0; T(13): 83.5; T(22): 100.0
A0FGR8	EIEDPEDRKPEDWDERPK	1		
Q09666	NDsLPVLR	1	S16(Phospho)	S(2): 0.0; S(5): 0.0; S(15): 50.0; S(16): 50.0
O60343	LKTEEGEIDYsAEEGENRR	1	S3(Phospho)	S(3): 100.0; S(6): 0.0
P46821	QGSKDPAEGDGAQPEETPRDGDKPEETQGK	1	C6(Carbamidomethyl); S10(Phospho); C11(Carbamidomethyl)	S(9): 87.3; S(10): 12.7; Y(13): 0.0
P16989	LQDSSDPDtGSEEEGSSRLsPPHsPR	3	S11(Phospho); S13(Phospho)	Y(2): 0.2; S(11): 96.6; S(13): 18.8; S(14): 84.3; T(22): 0.0
Q15435	DNPFSLGEsFGSR	2	S4(Phospho); S7(Phospho)	S(4): 100.0; S(7): 100.0
O00257	KNNHHEENISSK	3	S11(Phospho)	S(1): 0.0; S(11): 90.8; S(13): 9.2
P78559	DGKLDKDEIR	1	S6(Phospho)	S(6): 100.0
P27348	LGAGGGsPEKsPSAQELK	1	C15(Carbamidomethyl)	
Q13813	QAPPHIELSNSsPDPMAEAER	1		
A6NFI3	DTHEDHDTSTENTDEsNHDPQFEPIVSLPEQEIK	1	S6(Phospho)	S(6): 100.0
O94763	SLSSsPQAQPPRPAELSDEEVAELFQR	4	S3(Phospho); C4(Carbamidomethyl); C9(Carbamidomethyl)	S(1): 50.0; S(3): 50.0; T(8): 0.0; S(10): 0.0; T(12): 0.0; S(13): 0.0; S(15): 0.0
P60709	EATNTTSEPSAPSQDLLDLSPsPR	1		
Q01484	TLsNAEDYLDDEDsD	2	S3(Phospho)	S(3): 100.0; S(13): 0.0; S(19): 0.0; S(20): 0.0
Q86VQ1	KQDYQEILDsPIK	1	S3(Phospho)	S(1): 0.8; S(3): 99.2; S(6): 0.0
P67936	DQDKTDTLEHELRR	1		
Q9H4M9	RRsPsPAPPPR	3	S13(Phospho)	T(4): 0.1; Y(5): 0.0; Y(10): 0.8; T(11): 3.6; S(13): 95.6
Q9Y4E1	AGPDLPSLPSHALEDEGWAAAAPsPGsAR	1	S1(Phospho); S5(Phospho)	S(1): 99.4; T(4): 7.6; S(5): 93.0
Q15052	DKPHVNVGTIGHVDHGK	2	S2(Phospho)	S(2): 100.0; Y(6): 0.0
Q07954	NLIDEDGNNQWPEGLK	1	C10(Carbamidomethyl)	
P06241	SLsLDPGQSLEPHPEGPQR	2	Y7(Phospho)	Y(7): 99.4; T(8): 0.6
A6ND36	SKVGsTENIK	1	S3(Phospho)	S(3): 100.0
Q9H1E3	LISWYDNEFGYSNR	1	T3(Phospho); S5(Phospho); S15(Phospho)	T(3): 100.0; S(5): 100.0; S(15): 100.0
O94988	RKEDEVEEWQHR	1	S1(Phospho)	S(1): 45.4; S(2): 45.4; S(3): 6.9; S(6): 1.1; Y(7): 1.1; T(15): 0.0; T(20): 0.0
Q69YU3	tSPLKDNPsPEPQLDDIKR	2	S3(Phospho)	S(3): 100.0; T(6): 0.0
Q6P1N0	QDsWEVVEGLR	1	S4(Phospho)	S(4): 99.8; T(9): 0.2
P08238	KVMDsDEDDDY	1	S6(Phospho)	S(6): 100.0; S(12): 0.0
P49840	NNHHEENISSK	4	Y7(Phospho); C9(Carbamidomethyl)	S(6): 50.0; Y(7): 50.0; S(10): 0.0
Q6Y7W6	KQEEGAEIQcEAETGGTHKR	1	S3(Phospho)	S(1): 0.3; S(3): 99.7
Q86V48	KGAANASGsSPDAPAKDAR	2	S5(Phospho)	S(5): 100.0; S(13): 0.0; T(16): 0.0
P18615	ScFESsPDPELK	2	S1(Phospho)	S(1): 98.6; Y(3): 1.4; S(5): 0.0; S(8): 0.0; S(9): 0.0; S(10): 0.0
P07814	DHFGLEGDEESTMLEDSVSPK	1	T18(Phospho)	Y(2): 0.0; S(10): 0.0; S(12): 0.0; S(13): 0.0; S(15): 1.3; S(16): 10.2; T(18): 88.5
O95817	GRWEsQQDVSQTTVSR	1	S2(Phospho); S7(Phospho)	S(1): 0.7; S(2): 0.7; T(3): 98.6; S(7): 99.3; S(9): 0.7
P18669	HAsLDGASPYFK	1		
P46821	GLDsGAEtEEEKDTWEEKK	1	S3(Phospho); S8(Phospho); C-Term(Oxidation)	S(3): 100.0; S(8): 97.0; S(11): 0.7; T(14): 0.7; S(15): 0.7; T(16): 0.7; S(17): 0.2; Y(19): 0.0
Q86UE4	ADsKEsLKATTK	1	S12(Phospho)	S(1): 0.0; S(12): 100.0
Q7L4I2	EGFEsDTDSEFTFK	1	S10(Phospho)	S(5): 0.0; S(8): 8.4; S(10): 91.6
P02545	GMGsLDAMDK	1	S4(Phospho); S6(Phospho)	S(4): 50.0; S(6): 50.0; T(8): 4.5; S(9): 95.5
Q8IYB3	EFGYDsPHDLDsD	1	S4(Phospho); S6(Phospho)	S(1): 0.0; S(4): 100.0; S(6): 100.0
Q9NR19	KESKEETPEVTK	1	S3(Phospho)	S(1): 0.1; S(3): 99.9; S(9): 0.1
O95684	SIsSPNVNR	2	S16(Phospho); S20(Phospho)	T(3): 0.0; T(4): 0.0; S(12): 0.1; S(16): 99.9; S(20): 100.0
Q96RS0	ATLLNVPDLSDsIHsANASER	3	S13(Phospho)	S(6): 1.3; T(8): 49.2; S(13): 49.2; S(23): 0.2
P46821	GIPNMLLSEEETEs	5	S10(Phospho); S12(Phospho)	S(2): 1.4; S(4): 98.1; S(6): 0.2; S(8): 0.0; S(10): 0.2; S(12): 0.2; S(15): 99.8
Q92625	DWEDDsDEDMSNFDr	2	S3(Phospho)	S(1): 8.5; S(3): 91.5; S(6): 0.0
Q14160	EEATYGVWAERDsDDERPSFGGK	1	S2(Phospho)	S(2): 100.0; S(5): 0.0; S(7): 0.0; S(8): 0.0
Q13439	TAHNSEAADLEESFNEHELEPsSPK	1	S3(Phospho)	T(1): 12.5; S(2): 12.5; S(3): 72.8; T(5): 2.3; T(12): 0.0; S(19): 0.0; T(20): 0.0; S(23): 0.0
Q15435	DLYLENPEIK	1	S4(Phospho)	S(4): 99.9; S(7): 0.1
P50613	TTHFVEGGDAGNREDQINR	2	T3(Phospho)	Y(2): 6.3; T(3): 93.7; T(8): 0.0
Q7Z4V5	DAEGLDEIDHAEMELR	1	S3(Phospho)	S(3): 100.0; S(7): 0.0
P06703	RESVAsGDDRAEEDMDEAIEK	2		
O60506	ESSEKPPEKPAKPER	2		
Q9P2Q2	VTEQEWR	1	S6(Phospho)	Y(3): 8.6; S(6): 91.4
Q9ULU8	SSsQENLLDEVMk	5	S8(Phospho)	S(7): 49.8; S(8): 49.8; S(10): 0.3; S(12): 0.0; S(15): 0.0
Q96NE9	IQPQPPDEDGDHsDKEDEQPQVVVLK	2	S4(Phospho)	S(2): 8.4; S(4): 91.6
O43432	YSEKEDKYEEEIK	2		
P00338	NKKDELQSER	1		
Q00613	QKsAEPsPTVMsTSLGSNLSELDR	1	S7(Phospho); S11(Phospho)	S(7): 100.0; S(11): 100.0
O60841	ASsPGYIDSPTYSR	1	S4(Phospho)	S(4): 100.0; S(11): 0.0
P31948	sLQQLAEER	1		
Q9BST9	VsGQPQSVTASSDK	1	S3(Phospho)	S(3): 99.9; S(5): 0.1
P14618	AGDLLEDsPK	1		
Q96EB6	VIyDFIEK	1	S9(Phospho)	S(9): 100.0
P61313	SHsDTSIASR	1		
Q70E73	VQRPHsSPPR	1	S3(Phospho)	S(2): 9.8; S(3): 90.1; S(6): 0.1; S(7): 0.0; S(8): 0.0
Q12888	DVDIIDHHDNTYTVK	1	S9(Phospho)	S(1): 0.0; T(2): 0.0; S(8): 10.9; S(9): 88.9; T(11): 0.2
P14618	RSsGREEDDEELLR	1		
Q6R327	DNLAEDIMR	1	S3(Phospho)	S(3): 100.0; T(13): 0.0
P27824	DLAEDAPWK	1	N-Term(Acetyl); S3(Phospho)	S(3): 100.0; T(11): 0.0; S(13): 0.0
P35237	VGsTENIK	1		
P49736	DRNsWGGFSEK	2	S6(Phospho)	Y(4): 0.2; S(6): 99.8
P21333	QRIDEFEsM	1		
P07900	KDDTDDEIAKYDGK	1		
O94913	QKSFsEDVISHK	1	S3(Phospho); S8(Phospho)	S(1): 4.7; S(3): 95.3; S(8): 100.0
O15013	STPFIVPSsPTEQEGR	2	S3(Phospho)	T(1): 0.5; S(3): 99.5
O94979	TSsEDNLYLAVLR	1	S11(Phospho)	S(11): 100.0; Y(16): 0.0
Q15057	LLEGEESR	1	S3(Phospho)	S(2): 8.6; S(3): 81.8; S(5): 8.6; T(6): 1.0; S(8): 0.0; S(11): 0.0; S(15): 0.0
P29323	YKDIIRQPsEEEIIK	1	S7(Phospho)	T(6): 13.4; S(7): 81.1; T(10): 2.4; Y(11): 2.4; T(12): 0.5; S(13): 0.1
Q9NXH9	HsPVQMNR	1	C4(Carbamidomethyl); C5(Carbamidomethyl); S9(Phospho)	Y(6): 0.0; S(7): 0.0; S(9): 91.8; T(12): 8.2

P16070	sLPTTVPESPNYR	1	S3(Phospho)	S(3): 100.0; S(10): 0.0
O00193	SEIDMNDIK	1	S1(Phospho); S3(Phospho)	S(1): 69.0; S(3): 69.0; S(10): 31.0; S(11): 31.0
Q9BWH2	SIsNEGLTLNNSHVSK	2	S1(Phospho)	S(1): 100.0; T(6): 0.0
P49761	DLSSsPPGPYGQEMYAFR	3	Y1(Phospho)	Y(1): 94.1; S(3): 5.9; Y(9): 0.0; S(11): 0.0; Y(12): 0.0
P25205	QVEKNDDDQKIEQDGIKPEDK	1	S1(Phospho); S5(Phospho)	S(1): 0.0; S(5): 100.0; T(7): 100.0; S(14): 0.0
Q9BU76	EEcPRPMsIsPPDFSPK	1	S8(Phospho); S11(Phospho)	T(6): 0.3; S(7): 0.3; S(8): 99.4; T(10): 98.3; S(11): 1.7
P46821	tASNPKVENEDEPVR	2	T7(Phospho)	S(1): 0.0; S(4): 0.0; T(7): 100.0
Q86W92	HGESAWNLENR	1	S1(Phospho); C-Term(Oxidation)	S(1): 49.4; S(3): 49.4; T(4): 1.1; T(5): 0.2; S(12): 0.0
Q6UN15	LKFsDEEDGRDsDEEGAEGHR	2	S5(Phospho)	S(5): 90.6; T(7): 9.3; S(9): 0.1; S(13): 0.0
O60303	AGGGRPSsPSPSVVSEKEKEELER	1	S3(Phospho)	S(3): 98.6; T(5): 1.4; S(10): 0.0; T(12): 0.0
Q14498	RSLtNSHLEK	2	S3(Phospho)	Y(1): 6.4; S(3): 93.5; Y(5): 0.0; S(6): 0.0
Q8WWI1	QKEKEDAQEVELQEGK	1	S1(Phospho)	S(1): 48.6; S(3): 48.6; S(5): 2.8; S(7): 0.0; S(9): 0.0; S(11): 0.0
Q8NHG8	NNPHYDPSSKEDNPK	1	S7(Phospho)	S(7): 100.0
P61978	RLssLRASTSK	1	S6(Phospho)	Y(2): 0.0; S(6): 100.0
P35579	LGsFGSITR	1		
Q9NX40	RPsDSGPPAER	2	S3(Phospho)	S(2): 1.0; S(3): 99.0; Y(8): 0.0; Y(9): 0.0
P41091	GTEKREsPsPAPKPR	1		
P80723	RRsPsPPPTR	2		
Q96B36	GMYDGPVFDLTTtPK	2	T3(Phospho)	T(3): 100.0; S(4): 0.0
O75592	DcVGPEVEK	1	S3(Phospho)	S(1): 0.8; S(2): 0.8; S(3): 98.4
Q92973	DTSEDIEELVEPVAAHGPK	1		
Q7Z6B7	ATIsDEEIER	1	S4(Phospho)	S(4): 99.0; T(6): 1.0; Y(13): 0.0
P46937	LDVGNAEVKLEEENR	1	S1(Phospho); S3(Phospho)	S(1): 91.3; S(3): 50.0; S(7): 50.0; T(8): 8.7; T(12): 0.1; T(17): 0.0
Q96FS4	TAsFSESRADEVAPAK	3	S3(Phospho)	S(3): 100.0; S(8): 0.0; T(13): 0.0; S(14): 0.0; T(15): 0.0
Q9BRS8	KLNVTEQEK	1	S8(Phospho)	Y(1): 0.0; S(3): 0.0; S(8): 11.3; S(10): 88.6; S(15): 0.0
Q2M2I8	NPLPSKETIEQEKQAGES	1	T5(Phospho); S9(Phospho)	S(3): 2.0; T(5): 91.6; S(8): 53.1; S(9): 53.1; T(12): 0.2
Q5VTL8	DYDDMsPR	2	S4(Phospho)	S(2): 0.0; S(4): 100.0; S(9): 0.0
P27816	SFsKEVEER	2	S6(Phospho)	T(2): 0.0; S(6): 100.0; S(15): 0.0
P11142	sLPTTVPEsPNYR	3		
P62158	DLYDAGVK	1		
Q06830	SSsLSDLTHR	1		
P07737	TVsDNsLSNSRGEGKPDLK	1		
P46821	IDGATQSsPAEPK	1	S8(Phospho); S10(Phospho); S12(Phospho)	S(2): 0.0; S(4): 0.0; S(6): 0.6; S(8): 99.4; S(10): 99.4; S(12): 0.6; S(15): 100.0
P48634	RDSsEsQLASTESDKPTTGR	1	S5(Phospho); S8(Phospho)	S(3): 1.7; S(5): 80.8; S(8): 19.0; S(9): 11.0; S(10): 87.6
P25789	SEEAHAEDSVMDHHFR	1		
P27695	ESRPSEEREWDREK	1		
P42167	DYDEEEQGYDsEKEKK	1	S7(Phospho)	S(4): 0.4; S(7): 0.4; Y(10): 13.1; S(11): 86.2; S(17): 0.0
P49768	ILENEKDLEEAEEYKEAR	1	S7(Phospho); S8(Phospho)	S(7): 94.7; S(8): 52.6; S(9): 52.6
P40818	SsTPLHsPSPIR	1	S3(Phospho)	S(1): 8.0; Y(2): 8.0; S(3): 83.0; S(4): 0.8; T(8): 0.1
Q86YS7	EVPSKEEPsPVKAEVAEK	3	S7(Phospho)	T(3): 1.1; Y(4): 8.4; S(5): 8.4; S(7): 73.6; S(9): 8.4
Q13586	DREEALHQFR	1	S5(Phospho); C-Term(Oxidation)	T(3): 0.0; S(5): 99.2; S(7): 0.8; S(9): 0.0; S(10): 0.0; S(14): 0.0
Q9UMZ2	DLLHPSPEEEK	1	S7(Phospho)	S(1): 2.5; S(3): 2.5; S(7): 81.3; S(9): 13.7; S(17): 0.0; S(18): 0.0
Q9Y485	KLEELELDEQQKK	1	S15(Phospho)	S(2): 0.0; Y(12): 8.3; S(13): 8.3; S(14): 8.3; S(15): 75.0
Q8IWB9	KHLEINPDHSIIETLR	1		
Q9UQ35	AKsVDEIAK	1	C2(Carbamidomethyl); S6(Phospho)	S(1): 0.0; S(5): 0.6; S(6): 99.4
Q8IZ21	AHsHEEASRPAATSTR	1	S3(Phospho)	S(1): 0.6; S(2): 0.6; S(3): 98.8
Q8WYP3	GTAMPGEEVLESsQEALHVTER	2	S3(Phospho)	S(3): 100.0
Q92934	AEEDEILNRsPR	4	S3(Phospho)	S(3): 100.0; S(9): 0.0
Q9NZD8	DLYDDKDGLRK	1	S6(Phospho)	S(2): 0.0; S(6): 100.0
Q7L8J4	FTGsFDDDPDPHRDPYGEEVDRR	2	S4(Phospho); C9(Carbamidomethyl); S11(Phospho)	T(1): 0.2; S(4): 99.8; S(11): 100.0
P05455	EREDEsEDESDILEESPcGR	2	S3(Phospho)	S(3): 100.0; T(16): 0.0
Q5T8P6	IGEGtyGVVYK	1	S4(Phospho)	S(4): 100.0; S(8): 0.0; S(9): 0.0; S(10): 0.0
Q8NE71	RDsLTGSSDLYK	1	S3(Phospho); T6(Phospho); S7(Phospho)	S(3): 100.0; T(6): 100.0; S(7): 100.0
Q9Y6D5	RGsGDTsSLIDPDTSLSELR	1	S4(Phospho); S5(Phospho)	S(2): 2.8; S(4): 97.6; S(5): 99.6; S(11): 0.0
O14617	EVHDELEDLPsPPPPLsPPPTTSPHK	1	S19(Phospho)	T(1): 0.0; S(19): 100.0
Q16543	LEEVKPVVEVHHQsEQETSVR	1		
Q96D71	sRTAsGsSVTSLDGTR	4	S3(Phospho)	S(1): 0.4; S(2): 0.4; S(3): 99.2
P35579	SPSLsPsPPSPLEK	3		
Q09666	VDHGAEIItQSPGRSsVAsPR	3	S14(Phospho)	T(4): 0.0; S(6): 0.5; S(10): 12.4; S(12): 12.4; S(14): 74.7
Q92598	RTsFSTSDVSK	1	S3(Phospho)	S(3): 100.0
P68104	DAGDKDKEQELSEEDK	1		
O43719	NAEQYKDQADK	1	S2(Phospho); C12(Carbamidomethyl); C21(Carbamidomethyl)	S(2): 50.0; S(3): 50.0; S(17): 0.0
P00338	KNsITEIsDNEDDLLEYHR	1		
Q13362	NTETsKSPEKDVPMVEK	3	S3(Phospho)	S(3): 100.0; T(11): 0.0
O15085	KKPcSETSQIEDTPSSKPTLLANGGHGVEGSDTTGsPTEFLEEK	1	T10(Phospho); T11(Phospho)	S(1): 0.0; S(6): 12.4; S(7): 87.6; T(10): 87.6; T(11): 12.4; S(15): 0.0; T(18): 0.0
Q06190	GGNVFAALIQDQsEEEEEEEKHPPKPAKPEK	2	S3(Phospho)	S(1): 0.1; S(2): 0.9; S(3): 99.0; S(10): 0.0
Q96I25	RAASsDQLRDNsPPPAFKPEPPK	3	S6(Phospho)	S(6): 100.0; Y(12): 0.0
Q9Y6X9	SAYQEYDsDSDVPEELKR	2	S3(Phospho); S7(Phospho)	S(3): 100.0; S(7): 100.0
Q16790	sRLTPVsPESSSTEEK	2		
P60709	GKEHDDIFDK	1	S10(Phospho)	S(2): 0.0; Y(3): 0.0; S(10): 100.0
Q13098	RGsQsDEDScSLHSQTLSEDER	1	S7(Phospho)	S(1): 13.6; S(7): 86.4; T(12): 0.0; S(16): 0.0; S(18): 0.0
P27816	VDEDsAEDTQsNDGKEVVEVGQK	1	S3(Phospho)	S(3): 90.0; T(4): 10.0
Q05655	FctELNQPtLPNIRKWK	1	S2(Phospho); S5(Phospho)	S(2): 99.0; S(5): 1.9; S(7): 98.0; S(9): 1.0; S(10): 0.1; Y(16): 0.0
Q8IYB3	VDLKsPQVDIK	1	S3(Phospho); S5(Phospho)	S(3): 100.0; S(5): 100.0; T(9): 0.0
Q03252	VAPDEHPILLTEAPLNPK	1		
O76021	sVGGSGGGSFGDNLVTR	2	S10(Phospho)	S(4): 0.1; T(6): 2.4; S(10): 97.5
O60271	KPGPPLsPEIRsPAGsPELR	1	S5(Phospho); S7(Phospho)	S(5): 100.0; S(7): 100.0; S(12): 0.0; T(13): 0.0; S(16): 0.0
Q68DK2	LDSQEPGRQtPDR	1	S8(Phospho)	T(2): 0.0; S(4): 1.9; S(8): 98.1
O43237	DLDEMDDDDDDDVGDHDDDHPGMEVVLHEDKK	1	C12(Carbamidomethyl); S15(Phospho)	Y(5): 0.0; S(15): 100.0
Q8NEM2	VKPAsPVAQPK	1	S8(Phospho); C9(Carbamidomethyl); C12(Carbamidomethyl)	S(8): 50.0; S(10): 50.0; S(13): 0.1; Y(14): 0.0
Q06830	RSYsSPDITQAIQEEEKR	3		
Q5VZK9	TDDEVVQREEEAIQLDGLNASQIR	1	S1(Phospho)	S(1): 50.0; S(2): 50.0; S(6): 0.0; S(10): 0.0
P05455	HEERQDEHGFISr	1	S3(Phospho)	S(3): 100.0; T(16): 0.0
Q68DQ2	DEILPTtPISEQK	1	S3(Phospho)	S(1): 50.0; S(3): 50.0; T(16): 0.0; S(17): 0.0
O15014	ADLNQGIGEPQsPSRR	1	C2(Carbamidomethyl); S4(Phospho); S7(Phospho)	S(4): 94.5; T(6): 52.7; S(7): 52.7
P78559	NNPsPPPDSDLER	2	S1(Phospho); C4(Carbamidomethyl)	S(1): 99.8; S(6): 0.1; S(10): 0.0
P17677	ScsAScVPHGAK	1		
Q02952	RPPsPEPSTK	3	S22(Phospho)	T(2): 0.0; S(4): 0.0; S(15): 0.0; S(22): 84.3; Y(25): 15.7

P12814	AQtPPGPSLSGSKsPcPQEK	1		
P46821	AKTQtPPVSPAPQPTEER	2	S15(Phospho)	S(2): 0.0; S(4): 0.0; S(13): 87.9; S(15): 12.1
P35221	DFQDYMEPEEGcQGsPQRR	1	S1(Phospho); S4(Phospho)	S(1): 82.0; T(3): 82.0; S(4): 25.5; T(7): 10.6; S(17): 0.0
Q15170	sRsPHEAGFcVYLK	1	S11(Phospho)	T(1): 0.0; S(11): 100.0
P27824	RSsPPGHYYQK	1	S3(Phospho); T11(Phospho); S13(Phospho)	S(3): 100.0; T(11): 100.0; S(13): 100.0
Q6PD62	KASsPsPLTIGTPESQR	1	S8(Phospho)	S(8): 100.0; T(13): 0.0
Q9NRX5	YLsFTPPEK	1	S4(Phospho)	S(1): 0.0; S(4): 100.0
O43617	DFDIAEQNESsDEEsLRK	2		
Q13813	sSPELLPSGVTDENEVTTAVTEK	2	S1(Phospho)	S(1): 100.0
Q96ST2	KEEsEEsDDDMGFGLFd	1	S6(Phospho); S8(Phospho)	S(6): 100.0; S(8): 100.0; S(15): 0.0
Q15527	SGSSsPDSEITELK	1	T11(Phospho)	S(4): 0.0; T(5): 0.1; T(11): 50.0; T(16): 50.0
Q8TCJ2	DGLEMEKc	1	S8(Phospho)	S(8): 98.2; S(9): 1.8; Y(21): 0.0
Q8IUD2	AAAYDIsEDEED	1	S7(Phospho); S11(Phospho)	S(4): 2.3; S(5): 13.3; S(7): 86.7; S(11): 97.7
Q9H0X4	GGGGSSSSSSELsTPEKPPHQR	2	S2(Phospho)	S(2): 100.0; S(10): 0.0
Q9BVG4	IQQHVGEEAsPR	1	S4(Phospho)	S(4): 100.0
P13639	AGETRFtDTR	1	C8(Carbamidomethyl)	
Q9Y2D5	KPsPAASPATK	2	S8(Phospho)	S(5): 1.1; T(6): 9.2; S(8): 89.7
P13646	NLsNEELTK	1		
P49736	NLSLtDFVGDESPsPER	1	S8(Phospho)	T(2): 0.0; T(6): 0.1; S(7): 0.6; S(8): 99.3
P78559	NHTHQQDIDDLKR	1	S16(Phospho); C-Term(Oxidation)	S(1): 0.0; T(2): 0.0; T(3): 0.0; S(4): 0.0; T(7): 0.0; S(16): 86.9; S(19): 13.1
P62258	RPHPQHSQQPPPPQQER	1		
Q9UDY2	GAAAHPDSEEQQQR	1	S4(Phospho); S11(Phospho)	S(3): 99.0; S(4): 1.0; S(11): 100.0
P04049	LGsVDSFER	1	S3(Phospho)	S(3): 100.0; T(9): 0.0; S(12): 0.0
Q8IZ21	KAsPEPEGEAAGK	1	S4(Phospho)	S(4): 47.4; S(5): 47.4; S(6): 5.1; S(10): 0.0
Q96B97	sSDPHRLESR	1	S3(Phospho)	S(3): 88.1; S(5): 1.4; T(9): 10.5
Q02952	DNEEKDSAFR	1	S3(Phospho)	S(3): 100.0; S(5): 0.0
P11142	QLtQPETHFGR	1		
Q86VP6	SRVsVsPGR	1		
Q8WYL5	RRsQSIEQESQEK	1	S3(Phospho)	S(1): 0.0; S(2): 0.0; S(3): 99.6; S(4): 0.3; S(6): 0.0; S(9): 0.0
Q6PKG0	DSELDKHLESR	1	S1(Phospho); S9(Phospho)	S(1): 81.7; T(4): 9.2; T(5): 9.2; S(9): 99.7; Y(12): 0.2
P46821	MEEFKDQLPADEcNK	1	S5(Phospho)	S(1): 0.0; S(4): 7.0; S(5): 93.0; T(10): 0.0
Q9Y2V2	DDSHSAEDsEDEKEDHKNVR	2	S3(Phospho)	T(1): 0.0; S(3): 99.9; T(5): 0.0
Q13625	SLsLESTDR	1	S5(Phospho)	S(4): 0.0; S(5): 100.0
P61204	AsWESLDEEWR	1		
Q15121	DLADELALVDVIEDK	1	S9(Phospho)	Y(1): 0.0; S(9): 100.0
Q71U36	DsLGAYASQDANEQGQDLGKR	2		
Q8IYB3	RPsSTSVPLGDK	2	S5(Phospho); S7(Phospho)	S(5): 100.0; S(7): 100.0
P17812	DHGNDKESNVLHQ	1	S4(Phospho); S5(Phospho)	S(1): 11.9; S(3): 79.7; S(4): 18.9; S(5): 78.7; S(8): 10.9; T(11): 0.0
P06748	KLTDTSKDEENHEESESLQEDMLGNR	1		
P08238	SMVNTKPEKTEEDSEEVREQK	2	S5(Phospho)	S(5): 100.0
Q96AQ6	RKsHEAEVLK	1	C5(Carbamidomethyl); S6(Phospho); S7(Phospho); S8(Phospho)	S(6): 100.0; S(7): 100.0; S(8): 99.9; T(12): 0.2
P09601	RDAGGPRPEsPVPAGR	1	S3(Phospho)	S(3): 100.0; S(5): 0.0
Q96C92	GHKHEDGTQsDSEDPLAK	1	S5(Phospho); S9(Phospho)	S(2): 0.1; S(5): 99.9; S(9): 99.9; S(11): 0.1; S(20): 0.0
P14618	VSPSKSPSLsPsPPSPLEK	2		
P11142	LAPVPsPEPQKPAPVsPESVK	1		
Q8WWI1	DYFEQYGK	1	S4(Phospho)	S(4): 100.0; S(10): 0.0
Q9Y2W1	SGsMDPSGAHPSVR	2	S4(Phospho)	S(2): 1.7; S(4): 86.9; T(6): 11.4
Q05519	DRPVGGsPGGPR	1	S11(Phospho)	Y(2): 0.0; Y(9): 14.6; S(11): 85.4
Q5T9C2	DQsPPPsPPPSYHPPPPPTK	3	S3(Phospho)	S(1): 0.0; S(2): 0.0; S(3): 100.0; S(5): 0.0; T(8): 0.0
Q00587	KVsYSHIQSK	3	S3(Phospho)	S(1): 0.4; S(3): 99.6; S(6): 0.0
P09382	LMEDLDR	1	C6(Carbamidomethyl)	
Q9BTA9	SLsPIIGK	1	S3(Phospho)	S(1): 0.2; S(3): 99.7; Y(8): 0.0; S(9): 0.0; S(11): 0.0; T(12): 0.0; S(15): 0.0
Q8IWC1	DLTDYLMK	1	S3(Phospho)	S(3): 100.0; S(7): 0.0; S(8): 0.0; T(9): 0.0
Q5SW79	DKHIEEVRK	1	S5(Phospho); S8(Phospho)	S(3): 99.9; S(5): 1.2; S(8): 98.8; S(10): 0.1; T(13): 0.0; S(15): 0.0
Q14697	GISsLPR	1		
Q01484	FDDTNPEKEKEDFEK	2	S3(Phospho)	S(1): 0.6; S(3): 98.9; S(6): 0.6
Q9H0B6	GAKEEHGGLIRsPR	1	S3(Phospho)	S(2): 0.5; S(3): 99.5
Q8IWS0	SLKEsEQEsEEEILAQK	1	S3(Phospho); S7(Phospho)	S(3): 100.0; S(7): 7.3; S(8): 92.7; S(10): 0.1; T(12): 0.0
Q9Y2K6	ALSSLHGDDQDsEDEVLTIPEVK	1	S12(Phospho)	S(12): 50.0; S(14): 50.0
Q9P206	KNTFTAWsDEEsDYEIDDRDVNK	2	S3(Phospho); S7(Phospho)	S(3): 100.0; S(7): 88.7; S(11): 11.1; Y(12): 0.2; T(19): 0.0
P78559	AIGSTSKPQEsPK	1	S5(Phospho)	T(3): 0.8; S(5): 98.3; S(6): 0.8
Q01082	RQLAELETEDGMQEsP	1	S3(Phospho); S7(Phospho)	S(2): 10.4; S(3): 89.7; S(7): 98.5; T(9): 1.5; S(10): 0.0
Q8IYB3	LHGGFDsDcsEDGEALNGEPELDLTSK	2	S3(Phospho); S5(Phospho)	Y(2): 0.4; S(3): 99.6; S(5): 100.0
Q07352	SLSNSNPDISGtPTsPDDEVR	2	S3(Phospho)	S(1): 0.0; S(3): 100.0
Q92614	FGSTGsTPPVsPTPSER	1	S7(Phospho)	S(1): 0.0; S(7): 100.0; T(17): 0.0; S(18): 0.0
Q9Y2K6	sLENPtPPFTPK	2	S12(Phospho); S14(Phospho)	S(12): 100.0; S(14): 100.0
P51858	RAsDDGKLTDPSK	2	S5(Phospho)	S(2): 0.0; T(3): 0.0; S(5): 99.8; S(9): 0.1
P27797	DKLPSTEVKEDSAYGSQSVEQEAEK	2		
O14639	VETLSPEsALQGsPR	1	S3(Phospho)	T(1): 0.5; S(3): 99.5; Y(7): 0.0
Q9GZY8	AEEQQLPPPLsPPsPSTPNHR	1	S3(Phospho)	S(3): 97.9; T(6): 2.0; S(8): 0.1
Q969E4	GPsPsSPTPPAAAAPAEQAPR	2	S17(Phospho)	S(17): 100.0
Q9BXF6	SQDVAVsPQQQQcsKSYVDR	1	S3(Phospho); S5(Phospho)	S(1): 0.8; S(3): 99.2; S(5): 0.8; S(6): 99.2; S(9): 0.0; T(10): 0.0
P42677	DLsPTLIDNSAAK	1	S6(Phospho)	S(6): 100.0
O60716	TVTPAssAKTsPAK	1	S2(Phospho); S5(Phospho)	S(2): 100.0; S(5): 100.0; S(8): 0.0
P46821	SQEVEDKTKEYELLSDELNQK	1	S9(Phospho)	S(4): 0.0; S(9): 100.0
P30622	DLLHNEDRHDDYFQER	1	S10(Phospho); S14(Phospho)	T(1): 0.0; S(3): 0.0; S(5): 0.0; S(7): 0.1; S(10): 99.9; S(14): 100.0
P60174	VPSAPGQEsPIPDPK	1		
P63104	KQAREEsEEsEAEPVQR	2		
P53985	NLETLPSFssDEEDSVAK	1	S8(Phospho)	S(2): 0.0; T(7): 1.7; S(8): 98.3; T(19): 0.0
P53396	RGSsAAAsPGSPPPGR	1	S3(Phospho)	T(1): 1.0; S(3): 98.9; S(5): 0.1; S(7): 0.0
	EKEVDGLLTSEPMGsPVSSK	1		
P27824	VSDQNsPVLPK	1	S10(Phospho)	S(10): 100.0
Q01813	AGsEEcVFYTDETASPLAPDLAK	1	S1(Phospho)	S(1): 100.0; T(8): 0.0; Y(9): 0.0
P23588	LVsFHDDsDEDLLHI	1	S5(Phospho)	S(1): 0.0; T(3): 0.1; S(5): 99.9; S(7): 0.0; S(8): 0.0; T(10): 0.0; T(12): 0.0; S(13): 0.0; T(14): 0.0; T(15): 0.0; S(16): 0.0; S(17): 0.0
P35568	DKsFDDEESVDGNRPSSAASAFK	1	S3(Phospho)	S(2): 13.7; S(3): 83.4; T(5): 2.6; S(7): 0.1; S(8): 0.1; T(9): 0.0; S(11): 0.0; T(13): 0.0
Q9BVG9	LPQPPEGQcYsN	1	S9(Phospho)	S(9): 100.0
O95425	RRDsGDNSAPSGQER	1	S3(Phospho); S10(Phospho)	S(1): 90.4; S(3): 9.6; S(10): 100.0
Q9C0C2	RGsIGENQIK	1	S3(Phospho)	S(3): 100.0

O43583	EREKEIsDDEAEEEK	1	T11(Phospho)	S(6): 3.7; T(11): 96.3
Q99613	HsLASTDEKR	1	S6(Phospho)	S(6): 100.0; T(12): 0.0
Q15477	SLDGAEFSRPASVsENHDAGPDGDKR	1	S3(Phospho)	S(2): 7.2; S(3): 92.8
Q96AT1	DKRPEGYNLKDEEGR	1	S13(Phospho)	S(13): 100.0
Q9Y6D5	HVsPVTPPR	1	S5(Phospho)	S(2): 0.0; S(4): 0.8; S(5): 99.2; S(11): 0.0
O60231	LEKPAKYDDIKK	1	S8(Phospho)	T(3): 0.0; S(8): 100.0
P07355	AKNsPPQAPSTR	1		
Q9P2G1	LDRsFLEDTTPARDEK	1	S7(Phospho)	S(7): 100.0
Q96ST2	AsPEPQRENAsPAPGTTAEEAMSR	1	S5(Phospho); S7(Phospho)	S(5): 100.0; S(7): 100.0
P62491	SDIsPLtPR	2		
Q8WWM7	NYHSGNDVEAYEYLNK	1	S7(Phospho)	S(3): 0.3; S(7): 99.3; S(9): 0.3; S(12): 0.0
Q13428	RRtAsPPPPPK	1	S9(Phospho)	S(9): 100.0
Q8IYB3	SHsAGEVGEDSR	1	S3(Phospho)	S(3): 100.0; S(5): 0.0
P26599	NGQISSEEPKQEEEREQGSDEISHHEK	1	S7(Phospho)	T(4): 0.0; S(6): 8.5; S(7): 91.5
P78559	GADsGEEKEEGINREDK	1	S3(Phospho); S6(Phospho)	S(3): 100.0; S(6): 100.0; T(10): 0.0; T(11): 0.0
P00558	RLsQSDEDVIR	1	C2(Carbamidomethyl)	
Q9UNE7	DTLNKDHGNDKESNVLHQ	1	S7(Phospho); S11(Phospho)	S(7): 99.5; S(11): 97.2; S(13): 3.3
Q6DD88	SPsPAHLPDDPK	3		
Q9H1B7	NTFTAWsDEEsDYEIDDRDVNK	1	S4(Phospho); S5(Phospho)	S(3): 21.5; S(4): 86.9; S(5): 86.9; S(8): 3.9; S(11): 0.9
Q6Y7W6	QAEMGTQEKsPGTsPLLSR	1	S5(Phospho)	S(5): 100.0
Q6PKG0	SEsVEGFLsPSR	1	S9(Phospho); S13(Phospho)	Y(3): 0.0; T(7): 1.9; S(9): 98.1; S(13): 100.0
Q05682	NsLESISSIDR	1		
Q8IYB3	DKEGDDQSHWR	1	T3(Phospho); T5(Phospho)	T(3): 100.0; T(5): 100.0
P11940	GGREGFEsDTDSEFTFK	1		
P62701	FLSHsTDSLNK	2		
Q8NBJ4	NFEDVAFDEK	1	S8(Phospho)	S(8): 99.9; S(12): 0.1
Q9UBF8	SPPsPVER	3	S1(Phospho); C8(Carbamidomethyl)	S(1): 100.0; T(11): 0.0
P36915	HDAELRVPsPTEEPYAPEL	1	S7(Phospho)	T(4): 0.0; T(6): 10.5; S(7): 89.3; S(11): 0.2; T(13): 0.0
Q9UHD8	DASLHGLSQYNsL	1	S9(Phospho)	S(4): 0.0; S(6): 0.1; S(9): 99.9
O95817	REsPsPAPKPR	2	S8(Phospho)	S(1): 0.0; S(2): 0.0; S(7): 7.5; S(8): 92.5; S(12): 0.0
Q9Y2V2	GsPATsPHLGR	1	S5(Phospho); S7(Phospho)	S(1): 0.0; S(5): 100.0; S(7): 100.0
Q147X3	LSEEAEcPNPStPSK	1	C1(Carbamidomethyl); C11(Carbamidomethyl); C12(Carbamidomethyl); S13(Phospho); S27(Phospho)	S(13): 82.5; S(27): 42.3; S(29): 42.3; S(35): 16.5; T(37): 16.5
Q13765	TLsSSAQEDIIR	1	S22(Phospho)	S(7): 0.0; T(13): 0.0; T(15): 0.2; T(17): 2.2; S(22): 90.3; T(30): 7.2
Q969E4	eTKLSAEPAPDFSDYSEMAK	1	S8(Phospho)	T(1): 0.0; T(5): 14.9; S(8): 85.0; S(12): 0.0
Q71UM5	KSYESsEDcSEAAGSPAR	2		
Q9NY27	SNsFSDER	1	S24(Phospho)	S(13): 0.0; S(15): 0.0; S(16): 0.0; T(17): 0.2; S(18): 0.2; S(20): 0.8; S(23): 15.0; S(24): 68.8; S(26): 15.0
P46821	KRSPsPsPTPEAK	1	S14(Phospho); S23(Phospho)	T(1): 0.0; S(6): 99.5; S(8): 0.5; S(10): 0.0; T(12): 0.0; S(14): 0.0; T(18): 0.1; Y(20): 10.6; Y(21): 10.6; S(23): 68.2; T(25): 10.6
Q9UGV2	EIIDLVLDR	1	S1(Phospho); S5(Phospho); S7(Phospho)	S(1): 2.7; T(3): 13.5; S(5): 72.9; T(6): 38.0; S(7): 38.0; S(8): 38.0; S(9): 96.7; S(12): 0.2; S(15): 0.0; S(18): 0.0
Q8WX93	LDLKsPK	1	S1(Phospho); S3(Phospho); S6(Phospho)	S(1): 100.0; S(3): 100.0; S(6): 100.0
Q9BUI4	DRsVSVDSGEQR	1	S3(Phospho)	S(3): 99.2; S(4): 0.8
Q5VTR2	SSsVEEKPLSHR	1	S10(Phospho)	S(1): 0.0; S(3): 0.0; S(8): 0.0; S(10): 0.0; S(11): 0.1; T(12): 0.1; S(26): 1.7; S(30): 5.1; S(31): 5.1; S(33): 16.3; S(34): 16.3; S(36): 55.1
Q8IY57	LSTTPsPTsSLHEDGVEDFRR	2	S3(Phospho)	S(1): 33.2; S(2): 33.2; S(3): 33.2; S(9): 0.0; S(10): 0.3; S(15): 0.0
Q12929	LAsPsGSTSSGLEVVAPEGTSAPGGGPGTLDDSATIcR	4	S5(Phospho); S6(Phospho)	S(3): 32.3; S(4): 32.3; S(5): 31.5; S(6): 8.5; S(8): 95.1; S(11): 0.2
P42167	RRtPtPPPR	2	S6(Phospho); S7(Phospho); S19(Phospho)	S(6): 100.0; S(7): 100.0; T(14): 100.0; S(19): 0.0
Q13884	DKDTDKFK	2	T12(Phospho); S17(Phospho)	S(3): 0.3; S(6): 0.1; T(12): 99.6; S(17): 100.0
O15320	ELLHSVHPEsPNLK	1	S3(Phospho); S8(Phospho)	S(3): 55.1; Y(5): 55.1; S(8): 89.6; S(14): 0.0; S(15): 0.0; T(17): 0.0
P38159	DRDDRDFDFcR	1	S5(Phospho)	Y(3): 11.1; S(5): 85.4; Y(11): 0.3; S(12): 1.6; T(13): 1.6
P21980	VHEMEKEHLNK	1		
Q12770	ELVLssPEDLTQDFEEMKR	1	S3(Phospho)	S(3): 99.9; S(7): 0.1; S(14): 0.0
Q14157	RAGDLLEDsPKRPK	2	T2(Phospho); S18(Phospho)	S(1): 0.0; T(2): 0.0; S(3): 0.0; S(8): 9.2; S(11): 89.9; S(12): 1.0; S(16): 0.0; S(17): 0.1; S(18): 99.9
Q8TC71	DKDDDEVFEKK	1	S3(Phospho); S5(Phospho); C8(Carbamidomethyl)	S(1): 0.8; S(3): 99.2; S(5): 100.0
Q9BXF6	GSDALSETsSVSHIEDLEK	2	S1(Phospho); S3(Phospho)	S(1): 83.6; S(3): 83.6; S(4): 26.0; S(5): 6.8; S(15): 0.0
P06756	VDNDENEHQLSLR	1	S13(Phospho)	S(13): 50.0; T(15): 50.0
Q9UK58	RPAEATSSPTsPERPR	1	S5(Phospho)	S(5): 90.6; S(8): 9.4
Q96HR8	mDETsPLVsPER	1	S13(Phospho)	S(13): 100.0
O75061	QPLLLsEDEEDTKR	2	T3(Phospho); S8(Phospho)	S(1): 1.4; T(3): 10.2; S(4): 85.9; T(5): 2.7; S(6): 11.6; S(8): 88.0; T(10): 0.2
O43164	RsPTPGKGPADR	2	S5(Phospho)	S(4): 50.0; S(5): 50.0
Q9P2R6	tSSIADEGTYTLDSILR	2	T7(Phospho)	S(5): 0.9; T(7): 99.1
Q8WUY3	EKEDTDVADGcREtPTK	1	S3(Phospho)	S(3): 100.0; S(5): 0.0
Q9NYV6	TFsATVR	1	S7(Phospho); S9(Phospho); C23(Carbamidomethyl)	S(7): 100.0; S(9): 100.0; T(22): 0.0
Q09666	sPFEIIsPPAsPPEMVGQr	1	S5(Phospho); S8(Phospho); S18(Phospho)	S(1): 0.1; S(2): 0.1; S(5): 99.8; S(8): 100.0; S(18): 14.2; S(19): 85.8
Q06190	RLsYNTASNK	1	S5(Phospho)	S(1): 0.0; S(4): 0.0; S(5): 100.0
Q13136	HIKEEPLsEEEPcTsTAIASPEK	1	S3(Phospho)	S(2): 0.1; S(3): 7.8; S(6): 92.0; S(8): 0.1
Q9UFC0	QNLLAPQNAVssEETNDFKQETLPSK	1	C2(Carbamidomethyl); S6(Phospho); S12(Phospho)	S(4): 50.0; S(6): 50.0; S(12): 99.9; S(17): 0.0; S(20): 0.0
Q9NTI5	THTTALAGRsPsPASGR	1	S14(Phospho)	S(14): 100.0
Q9NTI5	SMVSPVPSPtGtISVPNScPAsPR	1	S8(Phospho); S10(Phospho)	S(4): 3.0; T(6): 89.5; S(8): 53.8; S(10): 53.8
Q9NQT8	GNsVEELEEMDSQDAEMTNTTEPMDHS	1	S3(Phospho); C9(Carbamidomethyl)	S(3): 100.0
Q01804	TDScssAQAQYDTPK	1	S3(Phospho); S4(Phospho)	S(3): 100.0; S(4): 100.0; S(11): 0.0
Q13177	NLDPDPEPPSPDsPTETFAAPAEVR	2	S3(Phospho)	Y(1): 0.0; S(3): 99.6; T(5): 0.3
P55196	DSISAVSSEKVSPSKsPsLSPSPPSPLEK	1	S3(Phospho)	S(3): 100.0; S(9): 0.0
Q9BTA9	SEASPHENTNHKsPHK	1	S4(Phospho)	S(2): 2.8; S(4): 96.9; Y(9): 0.1; S(10): 0.1; S(12): 0.0; T(13): 0.0; S(16): 0.0
O75962	SKscHDLSVL	1	S10(Phospho)	S(7): 1.9; S(9): 85.9; S(10): 12.2
Q92504	KEtPPKEVK	1		
P09488	HIEQLTTAsEHcDLAIK	2	C5(Carbamidomethyl)	
Q5T5U3	SRsESETSTMAAK	1	S7(Phospho)	S(3): 82.1; S(5): 9.0; S(7): 9.0
Q9Y3M8	GNDPLTSsPGR	1	S6(Phospho); C12(Carbamidomethyl)	S(6): 100.0; S(14): 0.0
P13639	EAEADLSFDSYHSTQTDLGPSLGKPGETSPPDsK	1	T7(Phospho)	T(4): 5.0; T(7): 94.7; T(9): 0.3
P26038	DLQGRDEQSEEK	2	S8(Phospho)	S(8): 100.0
Q13813	DSFIENSSSNcTSGSsKPNsPsISPSILSNTEHK	1		
P08559	sLPELDRDKSDSDTEGLLFSR	1	Y1(Phospho); S12(Phospho)	Y(1): 0.1; S(5): 99.9; S(7): 0.0; S(12): 50.0; Y(13): 50.0
Q8IYB3	DSQEKPKPVPDKENK	2	S7(Phospho); S9(Phospho)	T(2): 0.0; S(7): 100.0; S(9): 100.0
Q9Y5K6	RRtPsPPPR	3	S6(Phospho)	S(6): 100.0; T(8): 0.0; S(10): 0.0
Q05209	SGsNTGLDYIK	2	S8(Phospho)	S(5): 13.3; S(8): 86.7
Q02952	KLEGNSPQGsNQGVK	1	S4(Phospho)	S(1): 0.0; S(4): 100.0
P25205	TGDLGIPPNPEDRsPsPEPIYNSEGKR	1	S11(Phospho)	S(4): 0.0; Y(5): 0.0; Y(8): 81.8; S(11): 15.1; T(13): 3.0; T(22): 0.0
P61204	SKscDDGLNTFRDEGR	1		
Q9Y2U8	SPQDPGDPVQYNRtTDEELsELEDR	1	S7(Phospho); S10(Phospho)	S(6): 18.2; S(7): 84.9; S(10): 96.8; S(15): 0.1
Q9H4A3	KGDRsPEPGQTWTr	1	S6(Phospho); S8(Phospho); S11(Phospho)	S(6): 100.0; S(8): 100.0; S(11): 100.0; S(16): 0.0; S(17): 0.0

Q9NPI6	SYIsPHsSPSHTPTR	1	S3(Phospho); S4(Phospho)	S(3): 19.6; S(4): 15.3; S(6): 82.5; T(9): 82.5; T(12): 0.1; S(15): 0.0
P15311	QGsPDQVsPVSEMTSTSLYQDKQEGK	1		
O15234	AKtPVTLK	1	S3(Phospho)	Y(1): 99.0; S(3): 1.0
Q8NC51	RQGsFSEDVISHK	1	S3(Phospho)	S(1): 9.1; S(3): 45.4; S(11): 45.4
Q9BRS8	sFAGNLNTYK	2	S10(Phospho); S14(Phospho)	T(6): 10.1; S(10): 89.9; T(13): 1.4; S(14): 98.6; S(18): 0.0
P21333	QAsLDGLQQLR	1		
O43432	RRPsPQPsPR	1	S3(Phospho)	S(1): 0.0; S(3): 100.0
Q14155	tENTTPITSRVARLLLyGyr	1	S3(Phospho)	S(3): 100.0; S(9): 0.0
Q14699	GAEAFGDsEEDGEDVFEVEK	1	S3(Phospho)	S(3): 100.0; S(7): 0.0; S(8): 0.0
Q9Y6G9	ARsVsPPPKR	1	S15(Phospho)	Y(5): 0.0; S(15): 100.0
Q8TB72	TKDGYVEVSGKHEEK	1	S3(Phospho)	S(3): 100.0; T(5): 0.0
Q13185	TDsTSDGRPAWMR	1	S3(Phospho)	S(3): 59.4; S(5): 12.5; S(7): 12.5; S(9): 12.5; S(12): 3.1
Q8TDY2	SKsEEAHAEDSVMDHHFR	1	S2(Phospho); C6(Carbamidomethyl)	S(2): 99.2; Y(3): 0.8
Q13951	GELsPSFLNPPLPPSIDDR	3	S6(Phospho)	S(6): 98.2; S(9): 1.8
P78559	RPsEGPQPLVR	1	S8(Phospho)	S(1): 0.0; S(6): 0.8; T(7): 8.2; S(8): 91.0; S(10): 0.0
Q9C0C2	GHTDTEGRPPsPPPTsTPEK	1	S2(Phospho)	S(2): 99.8; T(5): 0.1; Y(6): 0.0; S(7): 0.0; S(8): 0.0
P78559	SVsQERLEDSVLMK	2	S6(Phospho); T10(Phospho)	S(6): 100.0; T(10): 100.0; T(16): 0.0
Q04637	DDsHsAEDsEDEKEDHKNVR	1	S4(Phospho)	S(4): 50.0; T(6): 50.0
Q8TD16	GNKHDDGTQsDSENAGAHR	1	S2(Phospho); S8(Phospho)	T(1): 2.6; S(2): 14.6; T(7): 85.4; S(8): 97.4
Q2NKX8	TASESISNLsEAGsIKKGER	1	S4(Phospho)	S(4): 100.0
Q5T9S5	RLsAQAHPAGK	1		
Q8IYB3	NSPRPsPKQsPRNsPR	1	S3(Phospho); S5(Phospho)	S(3): 100.0; S(5): 100.0
P25685	DAINQGMDEELERDEK	1		
P27797	DLLDDLK	2		
Q9Y520	AQHEDQVEQYKK	1	S3(Phospho)	S(1): 49.9; S(3): 49.9; S(6): 0.1; T(9): 0.0
P29966	TSGNSSPKRIQSsPGALsEDK	1	S10(Phospho)	T(6): 0.0; S(8): 0.3; S(10): 1.9; T(13): 97.7
Q8TD16	DGsGDSHPDFPEDADIDLKDVDK	1	S10(Phospho); S16(Phospho)	T(9): 0.8; S(10): 99.0; T(15): 15.8; S(16): 84.4
Q8IYB3	EKGPTTGEGALDLSDVHSPPKsPEGK	1	S5(Phospho)	S(5): 100.0; S(7): 0.0
Q8IYB3	cYHFIHAEDVEGIR	1	S5(Phospho); S7(Phospho)	S(2): 0.0; S(5): 100.0; S(7): 100.0
Q96KC8	RRPGASPTGETPTIEEGEEDEDEAsEAEGAR	1	S12(Phospho); S13(Phospho)	S(12): 98.1; S(13): 99.7; S(17): 2.2
Q9UQ35	SKsVIEQVSWDT	1	S4(Phospho); S8(Phospho)	S(4): 100.0; S(8): 100.0
Q12874	DKPSVEPVEEYDYEDLKESSNSVSNHQLsGFDQAR	1		
Q9Y3E1	KEQSEVSVsPR	1	S26(Phospho); S27(Phospho)	T(2): 0.0; Y(4): 0.0; S(11): 0.2; S(12): 0.2; S(13): 0.2; T(15): 0.3; T(22): 66.3; S(26): 66.4; S(27): 66.4
Q969E4	ALQAPHsPSKTDGK	2	N-Term(Acetyl); S30(Phospho)	Y(5): 0.4; S(30): 99.6
O14974	SQsTTFNPDDMSEPEFk	1	S20(Phospho)	S(1): 0.0; T(2): 0.0; S(5): 0.0; T(8): 0.0; S(11): 0.0; S(20): 96.4; T(22): 3.6; S(26): 0.0
Q9BV36	GRNDsGEENVPLDLTR	1	C19(Carbamidomethyl); S21(Phospho); S29(Phospho)	T(14): 2.0; S(17): 76.5; S(21): 21.6; T(28): 5.1; S(29): 16.9; S(31): 61.1; S(33): 16.9
Q6P6C2	IEDVGsDEEDDsGKDKK	1	S5(Phospho); S10(Phospho)	Y(1): 92.7; S(5): 14.6; S(10): 46.4; Y(12): 46.4
P10451	SEsPPAELPSLR	2	S6(Phospho); S10(Phospho)	S(6): 97.1; S(10): 2.9; S(15): 99.4; S(19): 0.6
Q9H6Z4	EGAKDIDISsPEFK	1	S5(Phospho); S6(Phospho); S13(Phospho); C21(Carbamidomethyl)	S(1): 85.5; S(5): 17.2; S(6): 97.3; S(13): 85.0; Y(20): 15.0
O14730	cLsPDDSTVK	2	S8(Phospho); S10(Phospho); S11(Phospho)	Y(5): 99.6; S(8): 1.2; S(10): 99.6; S(11): 99.6; T(20): 0.0
Q9BTC0	NTETSKsPEKDVPMVEk	4	T10(Phospho); S13(Phospho); S15(Phospho)	T(10): 99.2; S(11): 97.1; S(13): 50.0; S(15): 50.5; T(19): 3.1
Q92945	SRERsPsPLR	1	S4(Phospho); S7(Phospho)	S(4): 100.0; S(7): 100.0
Q5TCQ9	SVVsDLEADDVK	1	S3(Phospho); S5(Phospho)	S(3): 100.0; S(5): 90.9; S(7): 9.1
Q5JTV8	SFsMQDLR	1	S5(Phospho); S7(Phospho); S8(Phospho)	S(5): 100.0; S(7): 100.0; S(8): 100.0; S(14): 0.0; S(15): 0.0; T(17): 0.0; S(20): 0.0; T(22): 0.0; S(24): 0.0
Q86SF2	ADEIcIAGsPLtPR	1	S13(Phospho)	S(13): 100.0
O60343	VMNTHsDDsGDDDEATTPADKSELHHTLK	1	S4(Phospho)	S(4): 99.4; S(7): 0.6; S(12): 0.0; S(14): 0.0
Q86SF2	RVGDPPQPLPEEPMEVQGAERAsPEPQR	1	S13(Phospho); C-Term(Oxidation)	S(13): 100.0
O75909	GSGREEDDELLGNDDSDKTELLAGQK	1	S5(Phospho)	S(5): 100.0
Q13131	AAGGNsREPsPLPELALR	1	T7(Phospho); T13(Phospho)	S(1): 0.0; T(7): 1.3; S(11): 98.7; T(13): 0.2; T(15): 99.8
Q9C0C2	sVNFsLTPNEIK	1	S14(Phospho)	S(14): 98.4; T(17): 0.5; Y(18): 0.1; S(19): 0.5; S(20): 0.5
Q86TB9	GVGDDQLGEEsEERDDHLLPM	1	S2(Phospho); S4(Phospho); S9(Phospho)	S(2): 50.4; T(3): 50.4; S(4): 99.2; S(9): 100.0
P19532	SHSsPSQIPK	1	S3(Phospho)	S(2): 7.0; S(3): 92.4; S(5): 0.6; S(10): 0.0
O43493	QyMEEIIKNIQKLyK	1	S2(Phospho)	S(1): 49.9; S(2): 49.9; T(7): 0.1; T(11): 0.0
Q9UQ35	DYVAPTANLDQK	1	S1(Phospho); S3(Phospho); C5(Carbamidomethyl)	S(1): 100.0; S(3): 100.0; S(6): 0.0; S(10): 0.0
P13861	EDQTEYLEER	1	S10(Phospho)	S(8): 50.0; S(10): 50.0; S(22): 0.0
O15085	EDEGEPGDEGQLEDEGsQEKQGR	1	S1(Phospho); T6(Phospho)	S(1): 100.0; T(6): 99.4; T(10): 0.6
Q96KC8	VFRIMDDDNNR	1	S10(Phospho); S11(Phospho)	S(10): 100.0; S(11): 99.8; S(15): 0.2
P21283	STsLNERPK	1		
Q96F86	SASQSsLDKLDQELKEQQK	1	S7(Phospho); C13(Carbamidomethyl); S14(Phospho)	S(1): 0.0; S(7): 100.0; S(14): 1.0; S(16): 49.5; Y(17): 49.5
Q5JTD0	ADSHKEQAPKPSEQKDSEQGSGQSK	2	S2(Phospho)	S(2): 100.0
O15027	sVASSQPAKPTK	1	S4(Phospho)	T(2): 79.7; S(4): 20.2; Y(16): 0.1
P62753	DGGFcEVcKK	1	S9(Phospho); S11(Phospho); S13(Phospho)	S(2): 0.0; S(3): 0.0; S(7): 10.7; T(8): 10.7; S(9): 78.8; S(11): 99.5; S(13): 2.3; S(14): 98.0
P35269	NLsFEIK	1	S7(Phospho); S11(Phospho); S17(Phospho)	S(3): 1.6; S(6): 21.2; S(7): 81.8; S(11): 95.4; T(15): 89.5; S(17): 4.6; S(22): 1.2; T(23): 1.2; S(24): 1.2; S(25): 1.2; T(26): 1.2
Q96C19	MYsFDDVLEEGK	1	S12(Phospho)	S(12): 98.7; S(14): 1.3
Q9UDY2	KSRVsVsPGR	1	S3(Phospho)	S(1): 8.1; S(3): 91.9; S(7): 0.0
O94979	SVTVVEDDEDEDGDDLLHHHHGSHcSSSGDPAEYNLR	1	S8(Phospho); S13(Phospho)	S(2): 0.4; S(8): 99.7; S(13): 99.9
Q8WWI1	RsEEKEAGEI	1	S5(Phospho)	S(5): 87.9; T(7): 11.8; S(9): 0.3; T(12): 0.0
Q07157	ERGsPLLGDHAVR	1	S12(Phospho); S15(Phospho)	T(2): 0.0; S(10): 50.0; S(12): 50.0; S(15): 50.0; S(19): 50.0
P08238	LAPITSDPTEATAVGAVEASFK	1	S7(Phospho)	S(7): 100.0
O43237	SSEPVQHEESIRKPsPEPR	1	C12(Carbamidomethyl); S15(Phospho); C-Term(Oxidation)	Y(5): 13.6; S(15): 86.4
O60841	AEAPPLEREDSGtFSLGK	1	S5(Phospho); S7(Phospho)	Y(4): 88.4; S(5): 88.4; S(7): 23.2
Q9H6Z4	sRTHsTSSSLGSGESPFSR	1	S17(Phospho)	S(11): 28.0; S(17): 69.5; S(26): 0.8; T(29): 0.8; T(41): 0.2; T(42): 0.2; S(44): 0.2; T(45): 0.2
Q96PU5	DVIEEYFK	1	S8(Phospho); S12(Phospho); S16(Phospho)	T(2): 0.0; S(4): 0.4; S(8): 99.6; S(12): 97.8; Y(14): 2.3; S(16): 99.9
	NTPSQHSHSIQHsPER	1	N-Term(Acetyl); S12(Phospho); C-Term(Oxidation)	S(1): 0.0; S(5): 0.0; S(8): 0.1; S(11): 9.1; S(12): 90.8
Q9NP74	KGDEPQASGYHsEGETLKEK	1	S13(Phospho)	S(1): 3.3; S(4): 11.1; T(9): 42.8; S(13): 42.8
O60841	TVsAsSTGDLPK	1	S5(Phospho); S6(Phospho); S9(Phospho)	S(1): 50.0; S(5): 50.0; S(6): 5.9; S(9): 98.1; S(13): 95.9; S(19): 0.0
Q9H1E3	DHSKPISNPSDNK	2	S13(Phospho)	T(1): 0.0; S(5): 0.0; S(13): 100.0
Q03001	NHGSsPEQVVRPK	2	S9(Phospho)	S(5): 0.6; S(9): 99.4
Q9Y2X3	NYSsPPPcHLSR	1	S8(Phospho); C13(Carbamidomethyl); S15(Phospho)	S(8): 100.0; T(14): 3.1; S(15): 0.6; T(16): 0.6; S(20): 95.7
P42684	EKTEsELKFEEDER	1	S3(Phospho)	S(3): 99.9; S(5): 0.1; S(6): 0.0
Q6R327	GQLIssPTFNAPAALFGEAAPQVk	1	S5(Phospho)	S(5): 99.4; T(15): 0.6
P18615	RRssSPFLSK	1	S3(Phospho)	S(1): 17.2; S(3): 82.8; T(10): 0.0; T(12): 0.0; T(14): 0.0
Q9NPH3	SQVEDPLPPVFsGtPK	1	S3(Phospho)	S(2): 33.3; S(3): 33.3; S(4): 33.3; S(10): 0.0; Y(11): 0.0; S(12): 0.0; S(13): 0.0
Q92597	tSPGGRTsPEAR	2	S9(Phospho); S15(Phospho)	T(1): 0.0; S(3): 0.0; S(5): 0.0; S(6): 0.1; T(8): 49.5; S(9): 49.5; T(13): 2.5; S(15): 98.4
Q15154	EDSGLFsPIR	1	S4(Phospho); S7(Phospho); C18(Carbamidomethyl)	S(4): 100.0; S(7): 100.0; S(15): 0.0; S(21): 0.0; S(25): 0.0
P46821	ESAAPAsPAPsPAPSPTPAPPQK	1	S3(Phospho); S5(Phospho); S7(Phospho)	S(1): 99.2; S(3): 9.2; S(5): 91.6; S(7): 99.1; S(10): 0.9
Q07960	SAYQEYDsDsDVPEELKR	1	S7(Phospho); S8(Phospho)	S(1): 0.0; S(4): 0.1; S(6): 52.3; S(7): 52.3; S(8): 95.4; T(13): 0.0
P12882	GVDEVTIVNILTNR	1	N-Term(Acetyl)	

Q9H7D0	LRTsTSDLSR	1	S1(Phospho)	S(1): 100.0
P23588	RPLsSSHEASEGQAK	1	S4(Phospho); S10(Phospho)	S(1): 2.9; S(3): 2.9; S(4): 94.0; T(6): 12.0; S(10): 74.0; T(12): 12.0; S(13): 2.2
P48960	KLsVPtsDEEDEVPAPKPR	1	S5(Phospho)	S(5): 99.9; S(7): 0.1
Q9Y4J8	IEsPKLER	1	S9(Phospho)	T(1): 0.0; S(9): 85.7; T(11): 12.3; S(12): 2.0
Q9UDY2	SSHcDsPPRSQTPQDTNR	1	S3(Phospho); S7(Phospho)	S(1): 0.1; S(3): 99.9; S(7): 100.0
P51159	ALLFVPR	2	T7(Phospho)	S(1): 33.3; S(6): 33.3; T(7): 33.3; S(11): 0.1
Q05193	SGGLQTPEcLsREGsPIPHDPEFGSK	1	S1(Phospho)	S(1): 99.9; T(3): 0.1; S(4): 0.0; S(5): 0.0; T(7): 0.0
Q9BX95	DNQHQGSYSEGAQMNGIQPEEIGR	1	S2(Phospho)	S(2): 99.9; T(4): 0.1
Q92945	ETRQQLAQYQQQQSQASAPSTSRTTAsEPVEQSEATSk	2	S4(Phospho)	S(4): 92.2; S(7): 7.8
P05204	NEELEQLYQDQEVPKPK	1		
Q9Y653	tASISSSPSEGtPTVGSYGcTPQSLPK	1	S5(Phospho); S11(Phospho)	S(1): 7.4; S(3): 46.3; S(5): 46.3; S(11): 0.0; S(12): 0.0; S(14): 20.0; T(15): 20.0; S(16): 20.0; S(17): 20.0; S(18): 20.0
Q13191	GHSGTTAsGGENEREDLEQEWKPPDEELIKK	1	S3(Phospho); S7(Phospho); S10(Phospho)	S(3): 100.0; T(6): 10.8; S(7): 89.2; S(10): 50.0; S(11): 50.0
P02545	RLsPsAsPPR	1	S4(Phospho)	S(4): 86.1; S(5): 12.0; S(7): 1.8; S(8): 0.1; T(10): 0.0; S(15): 0.0; T(17): 0.0
O60841	RKsELEFETLK	1	Y4(Phospho)	Y(4): 33.3; S(5): 33.3; S(7): 33.3
Q9BZF1	APLKDEQEMRAsPK	1	S23(Phospho); C-Term(Oxidation)	Y(2): 0.3; S(3): 0.2; S(4): 0.3; S(12): 5.5; S(13): 2.0; S(15): 2.0; S(19): 16.7; S(23): 56.3; T(24): 16.7
Q5JSZ5	SSsVGsSSSYPISPAVSR	1		
Q8NI08	SsLPPTsPMEKFPKsr	1	S3(Phospho); S4(Phospho)	S(3): 99.7; S(4): 1.7; T(5): 10.8; S(6): 87.7
O43719	GTAGKsPDLSSQK	1	S7(Phospho)	S(7): 100.0
Q9NY27	NIDDASQMDLFHRDsDDDQTEEQLDESEAR	1		
O43815	RPESPsEIsPIKGSVR	1	S10(Phospho)	S(4): 0.0; S(10): 100.0
Q96FQ6	AAQQQEEQEEKEEEDDEQTLHR	1	S10(Phospho)	S(9): 0.0; S(10): 50.0; S(11): 50.0
Q9NYF8	TLSVAAAFNEDEDsEPEEMPPEAk	1	S10(Phospho)	S(10): 100.0; S(14): 0.0
P08238	LtRHVEASGGSGPGDsGPsDPRLAR	1		
Q9Y608	SEsVEGFLSPSR	1	S3(Phospho); S7(Phospho)	S(3): 99.6; T(6): 46.3; S(7): 46.3; S(8): 7.7; T(14): 0.0; S(15): 0.0; S(17): 0.0
Q14847	SRsLsPIcPR	1	S16(Phospho)	S(4): 0.0; S(16): 96.4; S(20): 1.2; S(21): 1.2; Y(22): 1.2
Q9UHY1	NQsSEDILR	1	T3(Phospho)	T(1): 0.0; T(3): 100.0; T(11): 0.0
O43765	SLDGAEFSRPASVsENHDAGPDGDKRDEDGESGGQR	1	S8(Phospho); S15(Phospho)	S(8): 6.2; T(12): 94.3; S(15): 99.4; S(19): 0.2
Q9NS87	RMsPKPELTEEQKQEIR	1	S9(Phospho)	S(1): 1.1; S(9): 98.9
Q9H6H4	HSsYPAGTEDDEGMGEEPSPFr	2	S3(Phospho)	S(1): 0.0; S(3): 100.0
P46821	SRsWEDSPER	1	S5(Phospho); C-Term(Oxidation)	S(2): 11.4; S(5): 88.6
Q96N67	eLDNNRSALsAAsAFAIATAGANEGTPNKEk	1	S5(Phospho); S7(Phospho); S12(Phospho)	S(1): 47.8; S(3): 47.8; S(5): 15.2; S(7): 89.2; S(12): 0.0; T(14): 0.0; T(16): 89.2; S(17): 10.8
Q99961	KVELsEsEEDKGGK	1	S6(Phospho)	S(4): 0.1; S(5): 7.5; S(6): 92.4
P31749	FKsDSGSLGDAK	1	S5(Phospho); S8(Phospho)	S(1): 8.2; S(3): 46.7; S(5): 47.0; S(8): 98.0; S(16): 0.2
Q9H6F5	DGDSYDPYDFSDtEEEMPQVHtPk	1	S8(Phospho); S16(Phospho)	S(8): 100.0; Y(15): 7.9; S(16): 46.0; S(19): 46.0
O00418	GSLGIsQEEQ	1	S9(Phospho)	S(9): 100.0
Q9Y2I7	DGGsGNsTIIVSR	1	S6(Phospho)	T(2): 0.0; S(6): 100.0; S(10): 0.0; S(11): 0.0
P08651	GDLKSsKAsLGsLEGEAEAEASSPK	1	S7(Phospho); S11(Phospho)	T(2): 0.0; S(7): 100.0; S(11): 0.0; S(13): 98.2; S(17): 1.8
P04233	DDHSFELDETALNR	1	S1(Phospho); C2(Carbamidomethyl)	S(1): 100.0
P21980	RSsDGSLSHEEDLAK	1		
Q9P206	IKESLLLEDsEEEEGDLcR	1	S8(Phospho)	S(8): 100.0
Q9UDY2	SGSssPDSEITELK	1	S1(Phospho)	S(1): 88.5; Y(6): 11.5
Q7KZI7	AGGAsPAASSTAQPPTQHR	1	S4(Phospho)	S(1): 9.6; S(4): 90.4
Q8IU81	DALGDSLQVPVSPSsTTsSR	1	S4(Phospho)	S(4): 100.0
Q96JG6	DTSQSDKDLDDALDK	1	S8(Phospho); S10(Phospho)	S(1): 0.0; Y(3): 0.0; Y(6): 3.1; S(8): 98.4; S(10): 98.4
O60504	AsLEDAPVDDLTR	1	S4(Phospho)	S(3): 47.6; S(4): 47.6; S(6): 4.8
Q93100	LGsEIRDSVV	1	S3(Phospho)	S(1): 0.1; S(3): 99.8; Y(5): 0.1
Q8N3F8	EQYVPPRsPK	1	S3(Phospho); S5(Phospho)	S(3): 100.0; S(5): 98.6; S(12): 1.4
Q96N67	HSsPHQSEDEEDPR	2	S1(Phospho)	S(1): 90.5; S(3): 9.3; S(5): 0.1; S(9): 0.0
P40763	VEsEEsGDEEGKKHSSGIVADLSEQSLK	1	C2(Carbamidomethyl); Y20(Phospho)	Y(1): 0.0; S(6): 0.0; S(16): 0.0; Y(20): 100.0
Q5SW79	NFsAAKsLLK	1	S10(Phospho)	T(8): 43.1; S(10): 43.1; S(12): 13.8
Q96PY6	DQNDHKYPFDR	1	C2(Carbamidomethyl); S3(Phospho)	T(1): 8.1; S(3): 91.9; S(8): 0.0
O95365	KPsEEEYVIRK	1	S8(Phospho)	S(8): 99.7; S(12): 0.3
Q96MK3	DsLGTYSSR	1	S13(Phospho); S15(Phospho); S16(Phospho); C-Term(Oxidation)	T(1): 0.0; T(5): 0.0; S(11): 2.6; S(13): 95.0; S(15): 7.5; S(16): 97.5; S(17): 97.5
Q9UQB8	yGEGHQAWIVGIVEK	1	S3(Phospho)	S(1): 6.9; S(2): 6.9; S(3): 86.2
O00418	sLSTSGESLYHVLGLDK	2	S3(Phospho); S4(Phospho)	Y(1): 21.9; S(2): 8.8; S(3): 77.1; S(4): 77.1; S(6): 15.0; S(10): 0.0
Q9Y5P4	STAQQELDGKPASPtPVIVASHTANKEEK	2	S3(Phospho); S5(Phospho)	S(1): 33.3; S(2): 33.3; S(3): 33.3; S(5): 12.1; S(6): 87.9; S(11): 0.0; S(13): 0.0
Q9Y490	WsIPADGR	1	S19(Phospho)	S(11): 2.7; T(12): 2.7; S(17): 24.7; S(19): 69.9
Q8NFQ8	iLRSSAPPsLAGPAVsHRGr	1		
Q9NVS9	ELSSPIsPK	1		
Q66K74	EKtPELPEPSVK	1	S1(Phospho); S3(Phospho); C10(Carbamidomethyl); C15(Carbamidomethyl)	S(1): 100.0; S(3): 100.0; S(13): 0.0
Q7Z5L9	KFLEESVSMsPEER	1	S5(Phospho); S8(Phospho)	S(3): 10.7; S(5): 89.4; S(8): 98.2; S(10): 1.6; S(11): 0.2
P36915	StLYFNTQEK	2	S8(Phospho)	T(5): 0.0; T(7): 1.5; S(8): 95.6; S(12): 1.5; T(14): 1.5
Q92615	YAEAENTIDQLr	1	S3(Phospho)	S(1): 8.6; S(3): 91.4
Q02952	GSLsLDDFR	1	S3(Phospho)	S(3): 92.5; S(5): 7.5
P78559	WDGsEEDEDNSKK	1	S7(Phospho)	S(3): 0.0; S(4): 0.1; S(7): 99.9
P07900	TPLTsADEHVHSK	1	S7(Phospho)	S(7): 100.0
O60841	KHYASEEIKEK	1	S5(Phospho); S7(Phospho)	Y(4): 0.3; S(5): 99.7; S(7): 99.9
Q96AQ6	sFEDLTDHPVTR	1	S7(Phospho)	S(7): 85.4; S(9): 14.1; T(11): 0.6
P26368	TVDLGIsDLEDDc	1	S12(Phospho)	S(12): 100.0
O94929	QVsFPDDDEEIVR	1	S3(Phospho); S6(Phospho)	T(2): 68.0; S(3): 68.0; T(5): 28.4; S(6): 28.4; S(8): 7.1; S(12): 0.0; S(13): 0.0; S(16): 0.0
P18206	IPRPSVsQGcsR	1		
P11413	RGLLyDSDEEDEERPAR	1	S1(Phospho)	S(1): 85.9; S(4): 14.1; S(13): 0.0
P61764	RTHsDAsDDEAFTTSK	1	S10(Phospho)	T(4): 0.0; S(9): 6.0; S(10): 94.0
P04406	EGVTPWAsFK	1		
Q99741	NSTLsDsGMIDNLPDSPDEVAK	1	T14(Phospho); T16(Phospho); C17(Carbamidomethyl)	S(2): 0.0; S(3): 0.0; T(10): 0.0; T(14): 1.3; T(16): 99.4; S(18): 99.4
Q9GZN8	RKAsEEIEDFR	1	S12(Phospho); C15(Carbamidomethyl); S18(Phospho); C-Term(Oxidation)	S(3): 62.7; Y(11): 20.6; S(12): 20.6; Y(17): 48.1; S(18): 48.1
Q9H4A3	DQsPSRAPGLR	1	S8(Phospho); S11(Phospho)	S(6): 99.2; S(8): 50.4; S(11): 50.4; S(16): 0.0; S(17): 0.0
P21333	EAARsPDKPGGsPSASR	1	C3(Carbamidomethyl); C11(Carbamidomethyl)	
Q15418	YDLDFK	1	T5(Phospho)	S(4): 50.0; T(5): 50.0; T(6): 0.1
Q05682	SKsIEEREEEYQR	1	S3(Phospho)	S(3): 100.0
Q9NW75	AQPSSsEDELDNVFFK	1	S7(Phospho)	S(7): 50.0; Y(11): 50.0
P27816	DGKYDLDFK	2	S3(Phospho)	S(3): 100.0; S(6): 0.0; T(8): 0.0
O95477	SPsPVLR	1	S2(Phospho)	S(2): 96.9; S(3): 2.4; S(4): 0.5; T(5): 0.1; S(7): 0.1; Y(8): 0.0
Q9BTU6	DGQVINETSQHHDDLE	2	N-Term(Acetyl); S5(Phospho); S9(Phospho)	T(4): 50.0; S(5): 50.0; S(9): 100.0
P28290	VEPSSQSPGRsPR	1	S4(Phospho)	S(1): 0.0; S(4): 100.0
P09104	DSNNLcLHFNPR	1		
P51957	MNDFHIsDDEEKNPSK	1	S3(Phospho); C6(Carbamidomethyl)	S(3): 99.9; S(4): 0.1; S(7): 0.0; T(9): 0.0
Q9H4G0	DPGTVANKKEEEDLAK	1	S4(Phospho); T6(Phospho)	S(4): 100.0; T(6): 100.0; T(17): 0.0

P16949	sLsSPTVTLSAPLEGAK	2	S3(Phospho)	S(3): 100.0
P35606	RLssLR	2		
P53396	NsPVtKTPPR	1	S3(Phospho)	T(1): 0.1; S(3): 99.8; S(5): 0.1; S(7): 0.0
Q9BVG9	RPsEsDKEDELDKVK	1	S10(Phospho)	S(10): 100.0
P49585	RHNsWSSSSR	1	S7(Phospho)	Y(4): 0.0; S(7): 100.0
Q9BTA9	AsPAPGSGHPEGPGAHLDMNSLDr	1	S6(Phospho)	S(6): 100.0
P07814	ATLVESStSGFtPGGGGSSVSMIASRKPtDGASSSNcVTDISHLVR	1	S13(Phospho); S15(Phospho)	Y(2): 0.0; S(10): 97.4; S(12): 69.0; S(13): 16.2; S(15): 16.2; S(16): 1.0; T(18): 0.3; S(21): 0.0; T(28): 0.0
P18206	YGPADVEDTTGSGATDsKDDDDIDLFGsDDEEESEEAKR	1		
Q8IYB3	SRsDIDVNAAAGAK	1	T3(Phospho)	T(3): 100.0; S(10): 0.0
P21333	NDSDLFGLGLEEAGPKEssEEGKEGK	1		
Q92538	RSsQPSPTAVPASDSPPTK	1	S5(Phospho); S6(Phospho)	S(3): 1.3; S(4): 10.3; S(5): 87.1; S(6): 2.8; S(9): 98.4; S(13): 0.0; S(14): 0.0; S(16): 0.0
Q7Z7L8	LNQVAIsQALsNALHSLDGATSr	1	T2(Phospho)	S(1): 50.0; T(2): 50.0; S(4): 0.0; S(6): 0.0
O60271	RAsDSVFQPK	1	S6(Phospho)	S(1): 0.9; S(3): 6.1; S(5): 46.5; S(6): 46.5
O00303	ESENSGDsGYPSEKRGELDDPEPR	2	C2(Carbamidomethyl); S4(Phospho)	T(1): 0.0; S(4): 100.0
Q9UPT8	RDsSSHEETPGSHPLYGHGEcK	1	S5(Phospho); S11(Phospho)	T(1): 3.9; S(3): 3.9; S(5): 92.4; S(11): 99.9
Q3MII6	TDVSNFDEEFTGEAPTLsPPR	1	S3(Phospho)	S(3): 100.0
Q9P2B2	yVRGLLQtEPQNNQAK	1		
Q8WUX9	MAGLRNESEQEPLLGDTPGSR	1	S2(Phospho)	S(2): 100.0
Q9H4L5	ASEENLLSSSSVPSADRDsSPTTNSK	1	S3(Phospho)	S(3): 100.0
P11137	KNQsSEDILR	1	S8(Phospho)	S(2): 0.0; T(6): 0.1; S(8): 99.9
Q13555	lDERSFLALDWDPDLKK	1	T3(Phospho); C6(Carbamidomethyl)	T(3): 100.0
Q13439	SKsNPDFLKK	1	S3(Phospho); S6(Phospho)	S(1): 0.7; S(2): 0.7; S(3): 98.6; S(6): 100.0
P46821	GPHHLDNSsPGPGSEAR	1		
Q13247	GNKHDDGTQSDSENAGAHR	1	S3(Phospho); S5(Phospho)	S(3): 100.0; S(5): 100.0
Q9NYL2	sPPLIGSESAYESFLSADDK	1	S3(Phospho)	S(1): 0.2; S(2): 1.2; S(3): 97.4; T(5): 1.2; Y(7): 0.0; T(10): 0.0
O75475	QGsREsTDGSTNSNSSDGTFIFPTTR	1	S6(Phospho)	S(2): 33.3; S(5): 33.3; S(6): 33.3; T(15): 0.0; S(16): 0.1; S(18): 0.0; T(22): 0.0
Q5VT25	DAIDREVEAVDSEYQLARPSDANR	1	S1(Phospho)	S(1): 91.6; S(3): 8.4; S(6): 0.0; T(7): 0.0
P60981	DKLSGEYEK	1	C3(Carbamidomethyl)	
Q49MG5	LGsFGsITR	1	S7(Phospho)	S(7): 100.0; S(15): 0.0
Q96JM3	AASsDQLRDNsPPPAFKPEPPK	2	S6(Phospho); S16(Phospho)	S(6): 100.0; S(16): 96.9; S(19): 3.1
Q9UKA4	SsPNPFVGsPPK	1	S7(Phospho)	S(3): 0.5; S(7): 99.5
Q9UDY2	REsGEEFR	1	S3(Phospho)	Y(2): 0.5; S(3): 99.5; Y(6): 0.0
Q96E09	YIcENQDSISSK	1	S3(Phospho)	S(3): 88.8; T(4): 9.8; T(5): 1.3; S(8): 0.0
P49815	KsQENLGNPSKNEDNVK	1	S3(Phospho)	S(1): 0.7; S(3): 99.3
P04080	sPESLPGPALEDIAIK	2		
P49810	RsPPEEPPDFccPK	1	S5(Phospho); S8(Phospho)	T(1): 0.0; S(2): 0.0; S(5): 100.0; S(8): 99.9; T(10): 0.1
Q14315	SQsWEER	1		
O75122	DRsPHRsSPSDTRPK	1	S4(Phospho)	S(1): 40.4; S(2): 40.4; S(3): 6.3; S(4): 6.3; S(5): 6.3; S(8): 0.2; S(14): 0.0; S(15): 0.0
P02545	LGSASSSHGSIQESHKAsRDPsPIQDGNEDMK	1	S3(Phospho); S5(Phospho)	S(2): 89.8; S(3): 10.2; S(5): 0.2; S(6): 1.4; T(8): 98.2; S(13): 0.2; T(15): 0.0
P49736	SIQPQVsPR	1	S9(Phospho)	T(7): 33.3; S(8): 33.3; S(9): 33.3
Q7Z5L9	GEKEEEDKDDEEKPK	1	S4(Phospho); S7(Phospho)	S(2): 0.2; S(4): 99.8; S(7): 88.3; S(9): 10.4; S(10): 1.3
Q9UMX1	SPFEIIsPPAsPPEMVGQr	1	S3(Phospho)	S(3): 99.9; T(7): 0.1
P62937	NRDSDKTDTDWR	1		
Q8NE71	YRsPYSGPK	1		
P35611	HNKsPTLScR	1	S4(Phospho)	S(4): 100.0
O60504	SRscREDQKPVMDDQR	1	S10(Phospho)	S(3): 0.0; S(5): 0.5; S(10): 99.5
O15344	RGNDPLTSsPGR	1	S8(Phospho)	S(2): 0.1; S(4): 0.6; S(8): 84.6; S(10): 14.5; T(12): 0.1
Q14004	SPTPTLPLsTSSFDEQN	1	S5(Phospho); S7(Phospho)	S(5): 100.0; S(7): 99.0; Y(9): 0.9; S(10): 0.1
O14578	SsTLSQLPGDK	2	S3(Phospho)	S(3): 95.1; S(11): 2.4; S(12): 2.4
Q9UDY2	DVDLEFLAK	1	S3(Phospho)	S(1): 0.0; S(3): 100.0
Q04637	AEDGATPSPSNEtPKKK	1	S3(Phospho)	S(1): 0.0; S(3): 100.0
O94854	ASSSKEsPSPSVR	1	S5(Phospho)	T(3): 8.0; S(5): 92.0
P17858	KHsDPHLLER	1	S3(Phospho)	T(1): 1.4; S(3): 98.6
Q9Y248	QQsEEDLLLQDFSR	1	S11(Phospho)	T(1): 0.0; S(8): 0.2; T(9): 1.5; S(11): 98.3
Q5SW79	SIQEIQELDKDDESLRK	1	T5(Phospho)	T(5): 98.4; S(7): 1.4; S(9): 0.2
Q13439	GKLEEQRPER	1	S3(Phospho)	S(1): 0.0; S(2): 0.0; S(3): 99.9; S(6): 0.0
P38159	IAWRAAKPLLMGKILytPDsPAAr	1	S5(Phospho)	Y(3): 0.0; S(5): 100.0
O43567	VLSGNcNHQEGTssDDELPSAEMIDFQK	1	T7(Phospho)	Y(2): 0.0; T(7): 100.0
P06733	SAcRLAIEFQSEPSAQENPFTAPsAKk	1		
Q07960	IALESEGRPEEQMESDNcSGGDDDWTHLSsk	1	S8(Phospho)	S(1): 0.0; S(4): 0.2; S(6): 1.5; S(7): 11.1; S(8): 87.2; T(13): 0.0
P28290	NDsGEENVPLDLTREPSDNLR	1	S1(Phospho); S4(Phospho)	S(1): 100.0; S(4): 100.0
O94929	SKESVPEFPLsPPK	1	S4(Phospho)	S(4): 89.5; T(6): 10.3; S(8): 0.2
P07237	NcNDFQYESK	1		
Q6AI12	DELHIVEAEAMNYEGsPIK	1	S3(Phospho)	S(3): 6.8; T(6): 93.1; Y(7): 0.1
Q9Y6M7	EANLQQNEEKNHSDSSTSESEVSSVsPLK	1	S3(Phospho)	S(3): 100.0
Q96PY5	TVAsDLQKcDsVEHLR	1	S1(Phospho)	S(1): 100.0
Q03111	nTTWEDVGLWDPSLTK	1	S6(Phospho); C10(Carbamidomethyl)	S(2): 0.6; S(6): 95.9; S(9): 0.6; S(11): 2.9
Q92974	GDREEEVERPVSsPGDPEQK	1	T9(Phospho); S20(Phospho); S24(Phospho)	S(4): 13.5; S(5): 86.5; T(9): 0.0; S(11): 0.0; S(16): 0.5; S(17): 15.3; S(20): 86.5; S(24): 97.8
P05114	ELVPEVPEEQGsKDR	1	S3(Phospho)	S(3): 50.0; S(4): 50.0
Q9H1E3	LPQTsDDEKKDF	1	S5(Phospho); S9(Phospho)	S(1): 99.8; S(5): 99.8; S(9): 0.3; S(12): 0.0
Q9BV36	cStGSPSKDVTK	1	T14(Phospho); S20(Phospho)	S(1): 0.0; Y(11): 0.8; T(14): 17.3; T(15): 84.9; S(20): 97.0
P36915	NTDDDRPPRR	1	T5(Phospho); T7(Phospho)	T(5): 99.4; T(7): 50.3; S(8): 50.3; S(12): 0.0; T(14): 0.0
Q05193	QLsKIER	1	S2(Phospho); S5(Phospho)	S(2): 88.9; T(4): 9.8; S(5): 2.5; S(6): 1.5; T(8): 97.3
P61764	TETQEKNPLPSK	1	S11(Phospho)	T(5): 0.0; S(10): 8.2; S(11): 91.8
Q8WYL5	VEPSSQsPGRsPR	1	S3(Phospho)	S(1): 49.9; S(3): 49.9; T(10): 0.1
Q53SF7	tKSLIAQDHR	1	S5(Phospho)	T(3): 2.5; S(5): 95.1; T(9): 2.5
